# Development of a Diastereoselective Csp^2^–Csp^3^ Cross-Coupling Reaction Inspired by Macrocyclic RiPP Natural Products

**DOI:** 10.1021/acs.orglett.5c02198

**Published:** 2025-07-09

**Authors:** Eleda V. Plouch, Eliott Le Du, Melanie Deville, John Bacsa, Hans Renata, Simon B. Blakey

**Affiliations:** ‡ Department of Chemistry, 1371 Emory University, 1515 Dickey Drive, Atlanta, Georgia 30322, United States; # Department of Chemistry, BioScience Research Collaborative, 3990 Rice University, Houston, Texas 77005, United States

## Abstract

Macrocyclic peptides containing C­(sp^2^)–C­(sp^3^) side chain cross-links are a rapidly growing subclass of ribosomally synthesized and post-translationally modified peptides (RiPPs), with significant potential in the development of new pharmaceuticals. This report presents a method for the efficient synthesis of derivatives of this class using a diastereoselective cross-electrophile coupling for the formation of the key β-aryl-alkyl cross-link.

## Graphical abstract

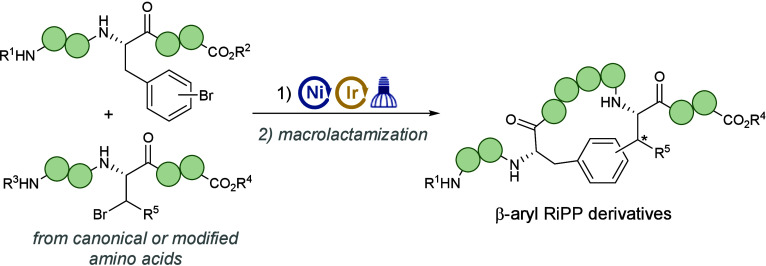

Ribosomally synthesized and post-translationally modified peptides (RiPPs) are a growing class of natural products exhibiting diverse and pharmaceutically interesting biological activities. In particular, one subclass of these RiPP natural products contains a post-translationally installed macrocyclic C–C bond linking an aryl residue to an alkyl residue at the β-position.^1^ This subclass includes natural products, such as streptide,^2,3^ celogentin C,^4–7^ xenorceptide,^8^ and the daropeptides,^9–12^ featuring darobactin A^13,14^ and dynobactin A,^15,16^ among others (Figure 1).^17–20^ Total syntheses of some of these natural products have been completed in recent years, including the seminal synthesis of streptide by Boger,^3^ and more recently, the syntheses of darobactin A by the Baran^21^ and Sarlah^22^ groups and the synthesis of dynobactin A by the Baran group.^16^ The biological synthesis of this subclass has also been explored in recent years.^23^ In the biosynthetic strategy, the linear peptide precursor is first assembled; then the macrocycle is constructed by tailoring enzymes that engage in selective C–H functionalization.

**Figure 1:**
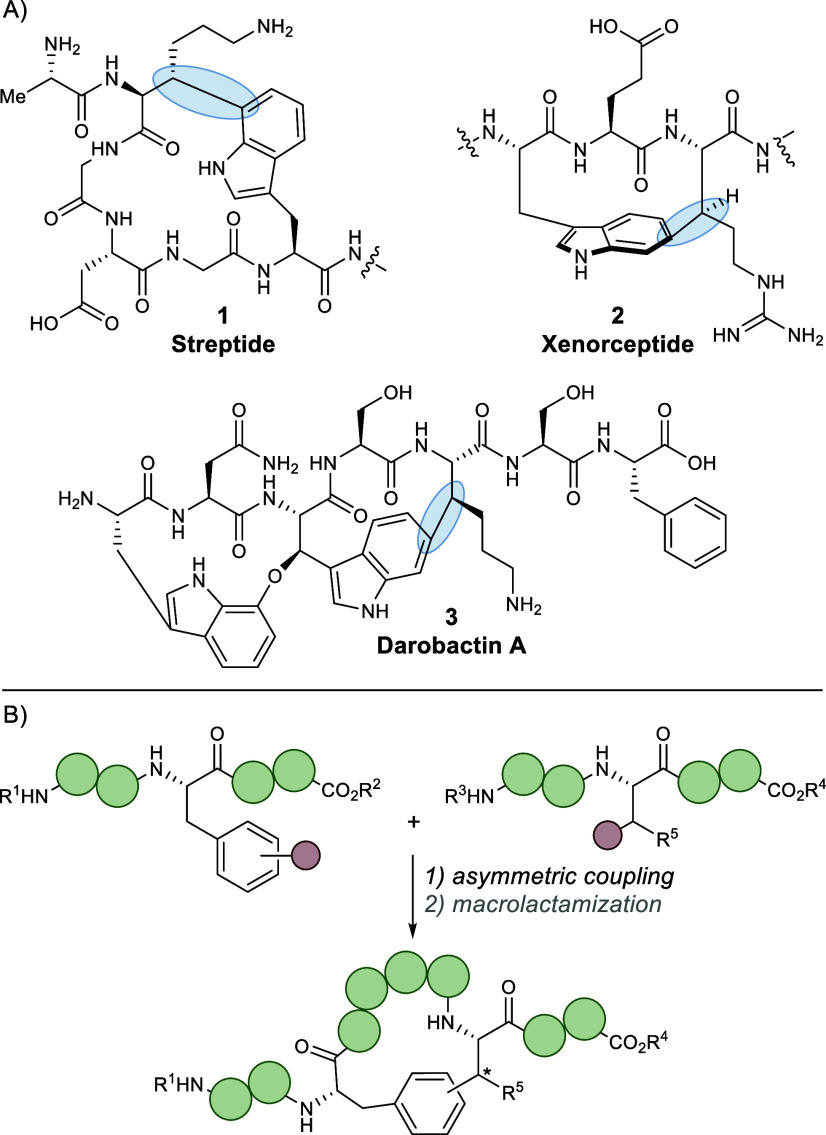
(A) Representative macrocycles from RiPP natural products of interest. (B) A convergent strategy for the synthesis of RiPP derivatives.

A primary challenge presented in the synthesis of this subclass is the stereoselective construction of the key aryl-alkyl C–C bond. In most syntheses, this bond is built with various directing or protecting group installations and removals, chiral auxiliaries, or challenging stereoselective hydrogenations. Other strategies are unable to access the β-position of alkyl amino acids.^24^ With current methods, emerging natural products in this rapidly growing subclass must each still be synthesized with a *de novo* strategy, limiting diversification and thus the structures available for biological testing.

Inspired by the biosynthesis of this subclass, we envisioned a more general synthetic strategy that circumvents the use of directing groups. This strategy would involve the straightforward construction of linear peptides containing preinstalled functional group handles on both aryl and alkyl amino acid residues, then, leveraging recent advances in asymmetric catalysis, would build the key β-aryl stereocenter, with subsequent macrocyclization producing the desired product (Figure 1). While this strategy does not perfectly mimic the biosynthetic strategy, it presents a more generalizable approach for the convergent synthesis of this subclass of RiPP natural products and their analogues.

Advances in metallaphotoredox^25–28^ and cross-electrophile^29,30^ couplings have cemented these reactions as powerful tools for the synthesis of complex molecules.^25,26,31,32^ The known compatibility of these reactions with peptide functionality and heterocycles, easily accessible functional group handles, and potential for asymmetric variants with chiral ligands made it an attractive reaction class for the implementation of our proposed strategy. At the start of our study, few stereoselective couplings involving preradical species on unactivated or otherwise unbiased secondary alkyl carbons were known.^33,34^ Furthermore, the effect of the adjacent chiral amine was unknown. In previous reports, cross-couplings of threonine derivatives had led to 1.5:1 mixtures of diastereomers.^35^ Thus, we set out to develop conditions for stereoselective cross-electrophile coupling at the β-position of α-amino acids.

Based on the prevalence of tryptophan residues as cross-linking sites in this subclass of RiPP natural products, we initiated our study by implementing nickel-catalyzed, silane-mediated reaction conditions, first pioneered by MacMillan and co-workers,^35,36^ in an amino acid system to couple 6-bromo-1-tosyl-1H-indole **4** with a series of brominated threonine derivatives (Figure 2). Using achiral ligand **L2** in 10 mol %, along with NiCl_2_·glyme, Ir­[(dF­(CF_3_)­ppy)_2_dtbbpy]­PF_6_, tris­(trimethylsilyl)­silane, Cs_2_CO_3_, and alkyl bromide (R^1^ = Cbz, R^2^ = Me), with DME (0.1 M), in a Penn photoreactor (450 nm LED light, 10% light intensity), the desired product was observed in 18% yield and 1:1.2 dr (Figure 2). Induction of stereochemistry was approached by screening a variety of chiral ligands. Bi-oxazoline (BiOx) ligands were found to be optimal under the standard reaction conditions, with **L1** providing the coupled product in 77% yield and 15:1 dr (Entry 1). Notably, these chiral ligands favored the minor diastereomer in the reaction with **L2**. The identities of the protecting groups on the threonine derivatives were found to be important for both reactivity and selectivity. An *N*-Cbz, *C*-OMe protecting group strategy was found to be optimal with chiral ligand **L1** (Entry 1), while *N*-Boc, *C*-OMe and *N*-Boc, *C*-OPh both provided the product in both lower yield and dr (51% yield, 12:1 dr; 34% yield, 4:1 dr; Entries 7–8). Notably, an *N*-Boc, *C*-Gly-OMe protecting strategy gave a lower yield and diastereoselectivity (59% yield, 1:1 dr, Entry 9). Other factors impacting yield were the choice of base, with Na_2_CO_3_ and 2,6-lutidine giving lower yields (42% yield, 17:1 dr; 20% yield, 7:1 dr, Entries 14 and 15), and light intensity, with 10% light intensity being optimal. The major byproducts observed were dehalogenated starting materials. The stereochemistry of both the major and minor diastereomers was determined by X-ray crystallography (Supporting Information). As the reaction proceeds through radical intermediates, the stereochemistry of the alkyl bromide starting materials is not expected to impact the product of these reactions.

**Figure 2:**
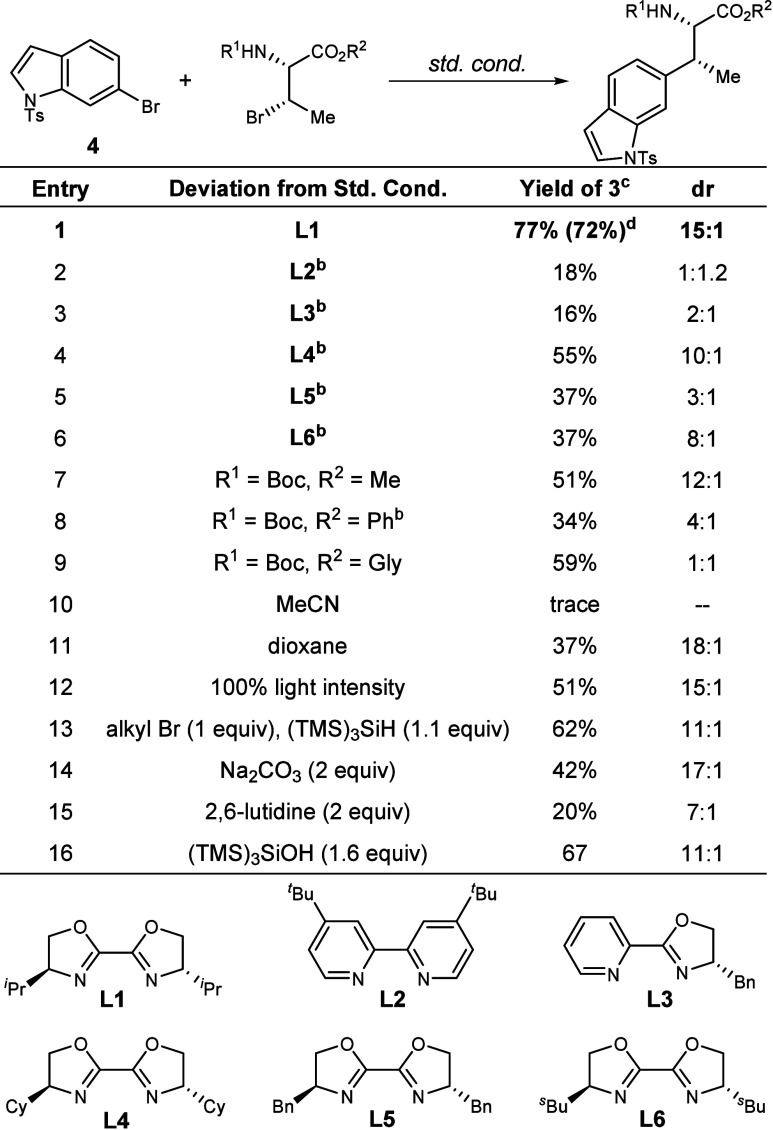
Optimization of the reaction conditions. ^a^Std. Cond.: NiCl_2_·glyme (10 mol %), Ir­[(dF­(CF_3_)­ppy)_2_dtbbpy]­PF_6_ (1 mol %), **L1** (10 mol %) tris­(trimethylsilyl)­silane (1.6 equiv), Cs_2_CO_3_ (2.0 equiv), **4** (1.0 equiv; 0.1 mmol), alkyl bromide (R^1^ = Cbz, R^2^ = Me; 1.5 equiv), DME (0.1 M), 16 h, 450 nm LED Penn photoreactor, 10% light intensity, 6800 rpm fan, 1000 rpm stir rate; dr determined from crude NMR; ^b^Run with 1 equiv alkyl bromide and 1.1 equiv tris­(trimethylsilyl)­silane; ^c^NMR yields with 0.1 mmol dibromomethane external standard; ^d^Isolated yield.

With the optimal conditions in hand, this method was applied to a variety of aryl amino acids with preinstalled aryl bromides, including phenylalanine (**6**–**8b**), tyrosine (**9a** and **9b**), and tryptophan (**10–11**) derivatives (Figure 3). *Para*- and *meta*-substituted phenylalanine products **6** and **7** were isolated in 66% and 67% yield, respectively, both in 6:1 dr. *Ortho*-substituted phenylalanine did not provide the desired product **8a** when reacted with a β-Br threonine derivative, but did yield the desired product **8b** when reacted with β-Br serine derivative (29% yield). Similarly, tyrosine-derived product **9a** was isolated in a lower yield (11% yield, 9:1 isolated dr) than **9b** (28% yield). Tryptophan-derived products **10** and **11** were successfully isolated in 47% yield (7:1 dr) and 66% yield (6:1 dr), respectively. However, the tryptophan derivative brominated at the indole C4 position did not provide a productive coupling reaction (only starting material and debrominated byproducts were isolated). These results suggest that the reaction is sensitive to steric congestion around the cross-coupling site.

**Figure 3:**
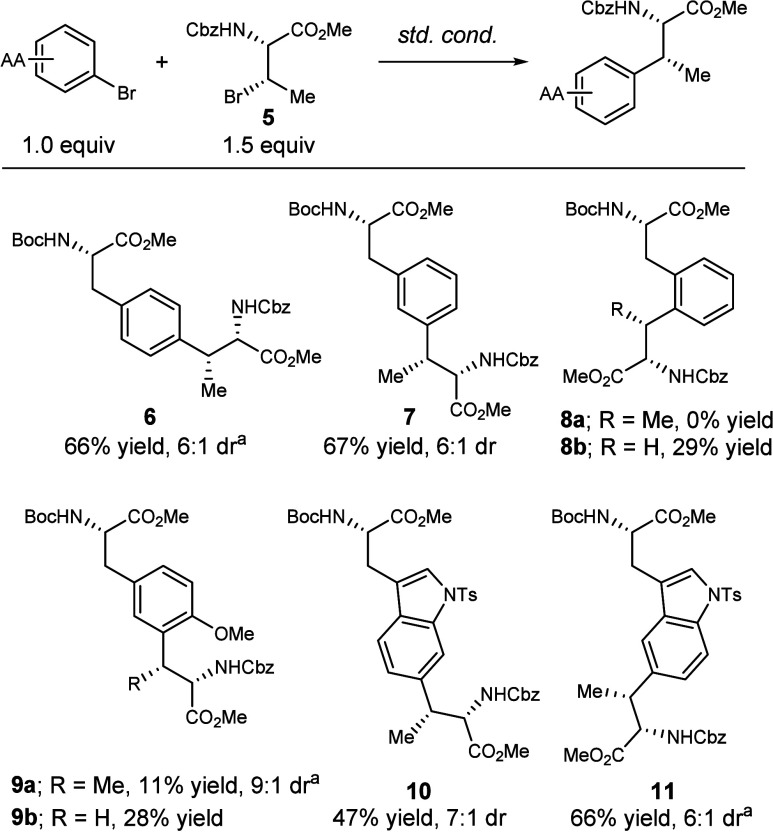
Scope of aryl amino acids. Standard conditions: NiCl_2_·glyme (10 mol %), Ir­[(dF­(CF_3_)­ppy)_2_dtbbpy]­PF_6_ (1 mol %), **L1** (10 mol %) tris­(trimethylsilyl)­silane (1.6 equiv), Cs_2_CO_3_ (2.0 equiv), aryl bromide (1.0 equiv; 0.1 mmol), **5** (1.5 equiv); dr determined from crude NMR, DME (0.1 M), 16 h, 450 nm LED Penn photoreactor, 10% light intensity, 6800 rpm fan, 1000 rpm stir rate; ^a^dr determined from NMR of purified sample.

To further understand the scope of the reaction, the reactivity of various alkyl amino acids was tested under these reaction conditions. Biocatalytic amino acid β-hydroxylation provided amino acid derivatives that were readily converted into the required brominated coupling partners for this study (see Supporting Information).

*N*-Cbz, O-Me protected amino acids were initially tested in the coupling reaction (Figure 4). Extending the alkyl chain lengths of the threonine derived starting material, derivatives of 2-aminopentanoic acid **12** and 2-aminohexanoic acid **13** were isolated in 53% and 58% yield, and 19:1 and >20:1 dr. *N*-Cbz-protected lysine derivative **14** was isolated in 48% yield with 6:1 dr. Consistent with sensitivity to steric congestion observed in the aryl scope, leucine derivative **15** was observed in only trace amounts. Similarly, a β-Br phenylalanine derivative also did not undergo the desired transformation.

**Figure 4:**
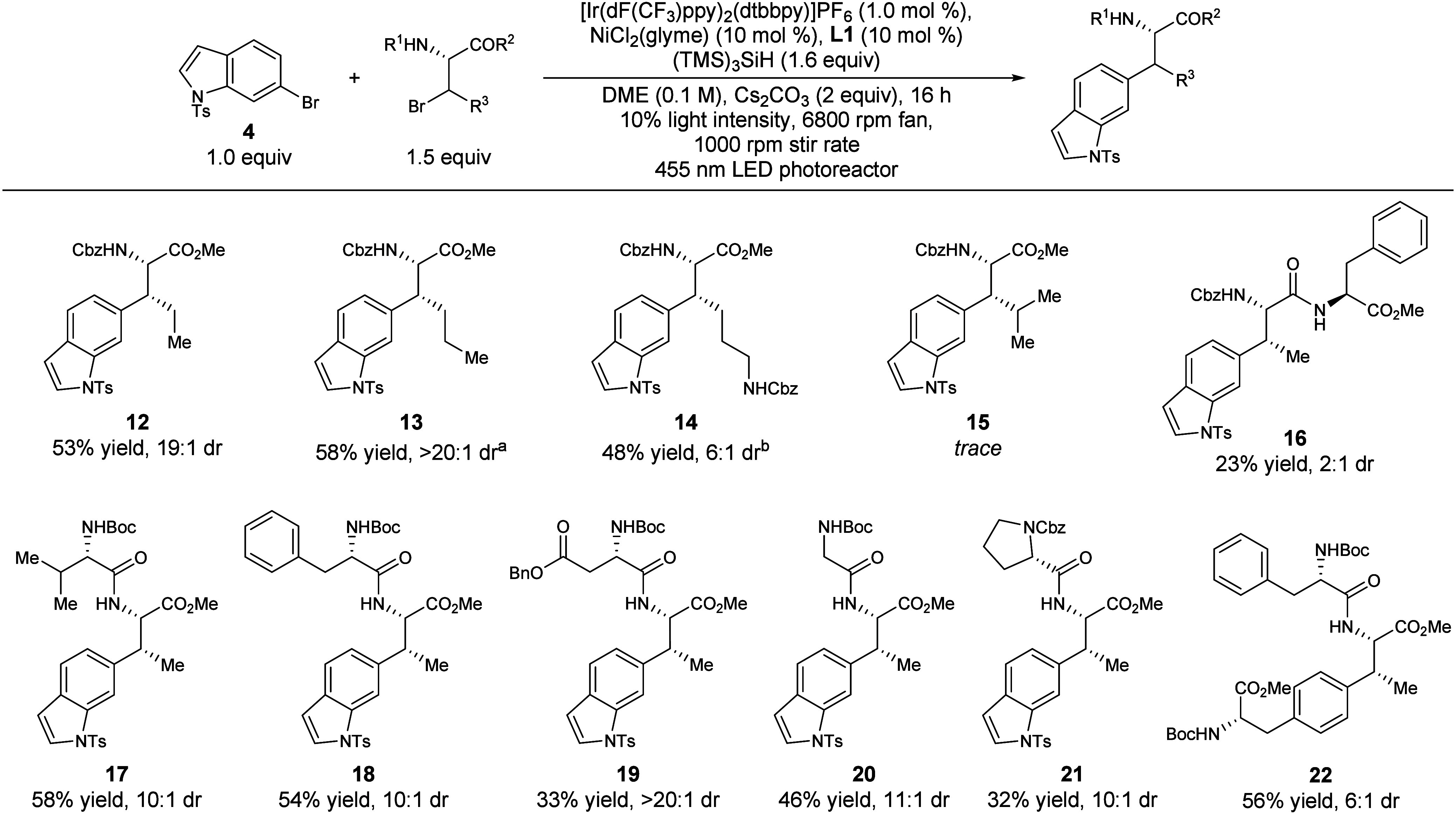
Scope of the alkyl amino acids. Standard conditions: NiCl_2_·glyme (10 mol %), Ir­[(dF­(CF_3_)­ppy)_2_dtbbpy]­PF_6_ (1 mol %), **L1** (10 mol %) tris­(trimethylsilyl)­silane (1.6 equiv), Cs_2_CO_3_ (2.0 equiv), **4** (1.0 equiv; 0.1 mmol), alkyl bromide (1.5 equiv), DME (0.1 M), 16 h, 450 nm LED Penn photoreactor, 10% light intensity, 6800 rpm fan, 1000 rpm stir rate; dr determined from crude NMR; ^a^0.05 mmol scale. ^b^1.0 equiv of alkyl bromide.

Various small peptides were next tested under these reaction conditions to evaluate how the identity of the dipeptide impacts the yield and diastereoselectivity of the reaction and the suitability of the conditions in the context of a convergent synthetic strategy. Threonine dipeptides wherein the alkyl bromide derivative is located at the C-terminus were all tolerated in moderate to good yields and good diastereoselectivities (**17**–**22**). Hydrophobic side chain dipeptides including valine-containing **17** and phenylalanine-containing **18** were isolated in 58% yield and 10:1 dr and 54% yield and 10:1 dr, respectively. The reaction also tolerated carbonyl-containing aspartic acid derivative dipeptide **19**, which was isolated in 33% yield and >20:1 dr. Additionally, glycine-containing dipeptide **20** was isolated in 46% yield and 11:1 dr, and proline-containing dipeptide **21** was isolated in 32% yield and 10:1 dr. However, the threonine-phenylalanine dipeptide with phenylalanine on the *C*-terminus significantly impacted diastereoselectivity, and **16** was isolated in only a 23% yield with 2:1 dr. Finally, when a phenylalanine dipeptide was reacted with a brominated phenylalanine derivative, product **22** was isolated in 56% yield and 5:1 dr, exhibiting the feasibility of this reaction in a peptide–peptide coupling setting.

To further exhibit the robustness of this method, reactants **4** and **5** were subjected to reaction conditions on a 1 mmol scale. Product **23** was isolated in 41% yield with a 15:1 dr (Figure 5).

**Figure 5:**
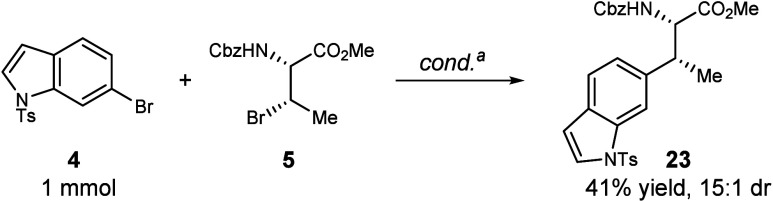
Reaction performed on a 1 mmol scale. ^a^NiCl_2_·glyme (10 mol %), Ir­[(dF­(CF_3_)­ppy)_2_dtbbpy]­PF_6_ (1 mol %), **L1** (10 mol %) tris­(trimethylsilyl)­silane (1.6 equiv), Cs_2_CO_3_ (2.0 equiv), **4** (1.0 equiv; 1 mmol), **5** (1.5 equiv), DME (0.1 M), 16 h, 450 nm LED Penn photoreactor, 10% light intensity, 6800 rpm fan, 1000 rpm stir rate, run in scintillation vial; dr determined from crude NMR.

To demonstrate the feasibility of this as a convergent strategy for the synthesis of macrocyclic peptides, we subjected dipeptide **24** and tripeptide **25** to the standard conditions and obtained coupled product **26** in 18% yield and 15:1 dr (Figure 6). Deprotection with TFA removed the *N*-Boc and O-^*t*^Bu protecting groups and the product was used immediately without further purification in the macrolactamization step,^37^ providing macrocyclic peptide **27** in 63% yield over two steps.

**Figure 6:**
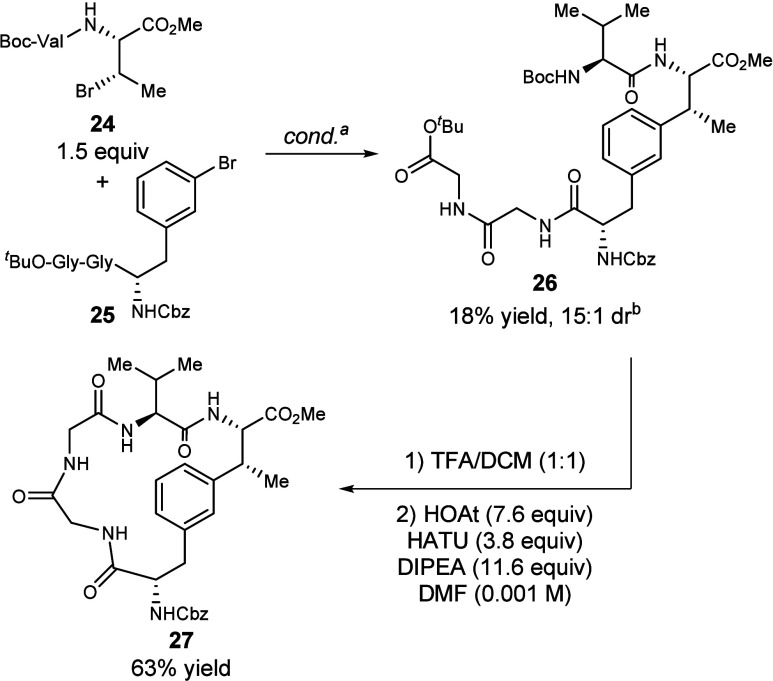
Synthesis of a macrocyclic peptide by convergent reductive coupling and subsequent macrocyclization. ^a^NiCl_2_•glyme (10 mol %), Ir­[(dF­(CF_3_)­ppy)_2_dtbbpy]­PF_6_ (1 mol %), **L1** (10 mol %) tris­(trimethylsilyl)­silane (1.6 equiv), Cs_2_CO_3_ (2.0 equiv), **24** (1.5 equiv), **25** (1.0 equiv; 0.1 mmol), DME (0.1 M), 16 h, 450 nm LED Penn photoreactor, 10% light intensity, 6800 rpm fan, 1000 rpm stir rate; ^b^dr determined from NMR of purified sample.

In summary, we report a new strategy for the synthesis of macrocyclic peptides containing β-aryl C–C linkages, involving incorporation of noncanonical amino acids into peptide chains, nickel-catalyzed asymmetric coupling to stereoselectively generate the key β-aryl C–C bonds, then macrolactamization to generate peptide macrocycles. Importantly, this strategy does not rely on directing groups or chiral auxiliaries to induce stereocontrol. Instead, the introduction of a chiral BiOx ligand enhances substrate control of diastereoselectivity. This study opens new doors to address the challenges associated with generating peptide libraries resembling this emerging subclass of RiPP natural products.

## Supplementary Material



## Data Availability

The data underlying this study are available in the published article and its Supporting Information.

## Notes

Experimental protocols, characterization data, NMR spectra, and single crystal X-ray data (PDF)
