# Supplementary material for: Development of a Diastereoselective Csp2–Csp3 Cross-Coupling Reaction Inspired by Macrocyclic RiPP Natural Products
Source: Org Lett. 2025 Jul 9;27(28):7627–32. doi: 10.1021/acs.orglett.5c02198 (PMC12281569; doi:10.1021/acs.orglett.5c02198)

Supporting Information

For

Development of a Diastereoselective Csp²-Csp³ Cross-Coupling Reaction Inspired by Macrocyclic RiPP Natural Products

Eleda V. Plouch‡, Eliott Le Du^, Melanie Deville‡, John Bacsa‡, Hans Renata^*, Simon B. Blakey‡*

‡ Department of Chemistry, Emory University, 1515 Dickey Drive, Atlanta, Georgia 30322, United States

^ Department of Chemistry, BioScience Research Collaborative, Rice University, Houston, Texas 77005, United States

*email: sblakey@emory.edu, hr28@rice.edu

Table of Contents

1. General Information 2
2. Preparation of Starting Materials 3
2.1 Preparation of Hydroxylated Amino Acids 4
2.2 Preparation of Aryl Bromides 15
2.3 Preparation of Brominated Amino Acids 21
2.4 Preparation of Dipeptides 27
2.5 Preparation of Brominated Dipeptides 31
3. Preparation of Cross-Coupled Products 36
4. Preparation of Cyclized Peptide 58
5. Crystal Structure Data 59
6. References 84
7. NMR Spectra 87

1. General Information

All reactions were conducted under nitrogen atmosphere with anhydrous solvents in oven- or flame-dried glassware using standard Schlenk technique, unless otherwise stated. Anhydrous dimethoxyethane (DME), dichloromethane (DCM), toluene, tetrahydrofuran (THF), were obtained by passage through activated alumina using a *Glass Contours* solvent purification system. Diisopropylethylamine (DIPEA, or Hünig's base) was distill over ninhydrin and KOH and stored over activated 4Å molecular sieves. Solvents for workup, extraction, and column chromatography were used as received from commercial suppliers without further purification. All catalysts were stored and weighed out in a nitrogen-filled glovebox. All other chemicals were purchased from Millipore Sigma, Strem Chemicals, Oakwood Chemicals, Alfa Aesar, TCI, Comi Blocks, Ambeed, or Aapptec and used as received without further purification, unless otherwise stated. Ligands L1-L6 were obtained from Ambeed. All photochemical reactions were run in a Penn PhD Photoreactor M2 with 450 nm light using a Multi-Vial Holder 4 mL and a 2.5" tall stand allowing for complete (360°) irradiation of the vial. The 2.5" stand was used as it held the vial just above (< 0.5") a 450 nm lamp attachment. The 1 mmol reaction was run using a Single Vial Holder 20 mL and a 2.5" tall stand. Additional information regarding the Penn PhD Photoreactor M2 can be found at https://www.acceledbio.com/photocatalysis-technology.

Unless otherwise noted, all yields refer to chromatographically and spectroscopically (1H NMR) homogenous materials. New compounds were characterized by NMR and HRMS. 1H and 13C NMR spectra were obtained from the Emory University NMR facility and recorded on a Bruker 400 (400MHz), Bruker 600 (600 MHz), INOVA 600 (600 mHz), INOVA 500 (500 MHz), VNMR 400 (400 MHz), or Bruker 800 (800MHz), and are internal referenced to residual protio solvent signals. Data for 1H NMR are reported as follows: chemical shift (ppm), multiplicity (s = singlet, d = doublet, t = triplet, q = quartet, p = pentet, m = multiplet, dd = doublet of doublets, dt = doublet of triplets, ddd = doublet of doublet of doublets, dtd = doublet of triplet of doublets, br s = broad singlet) coupling constant (Hz), integration, and assignment, when applicable. Data for decoupled 13C NMR are reported in terms of chemical shift and multiplicity when applicable. High Resolution mass spectra were obtained from the Emory University Mass Spectral facility using a Thermo Scientific Extractive Plus with an orbitrap mass analyzer. High Pressure Liquid Chromatography (HPLC) was performed on an Agilent 1100 series HPLC as indicated. Optical rotation were measured on a PerkinElmer 341 polarimeter. Analytical thin layer chromatography (TLC) was performed on precoated glass-backed Silicycle SiliaPureR 0.25 mm silica gel 60 plates and visualized with UV light. Silica gel column chromatography was performed using Silicycle SiliaFlashR F60 silica gel (40-63 μm). Flash column chromatography was performed using Silicycle SiliaFlashR F60 silica gel (40-63 μm) on a Biotage Isolera One system. Preparatory TLC was performed on precoated glass backed Silicycle SiliaPureR 1.0 mm silica gel 60 plates. Unless otherwise noted, all heating was performed with heating blocks.

Abbreviations include dichloromethane (DCM), dimethoxyethane (DME), dimethylformamide (DMF), ethyl acetate (EtOAc), methanol (MeOH), round bottom flask (RBF), thin layer chromatography (TLC).

The diastereomeric ratios (dr's) were determined via 1H NMR analysis of the unpurified reaction mixture unless otherwise indicated.

Structural assignments were made with additional information from gCOSY, gHSQC, and gHMBC experiments.

2. Preparation of Starting Materials

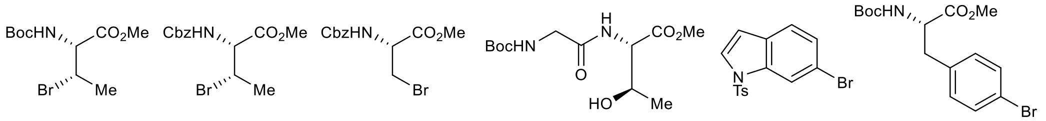

S1 **S2** **S3** **S4** **4** **S5**

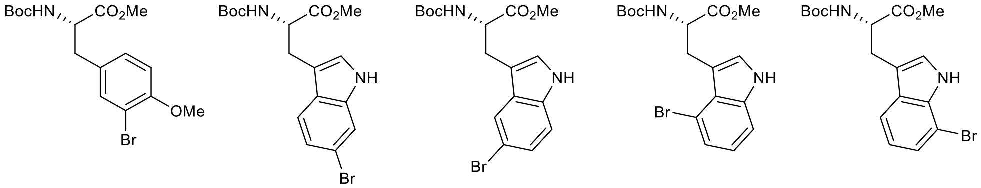

S6 **S7** **S8** **S9** **S10**

S1[1], **S2**[1], **S3**[2], **S4**[3], **4**[4], **S5**[5], **S6**[6], **S7**[7], **S8**[7], **S9**[7], **and S10**[7] were synthesized following reported literature procedures.

2.1 Preparation of Hydroxylated Amino Acids

General Materials and Methods

Unless otherwise noted, all chemicals and reagents for chemical reactions were purchased at the highest commercial quality and used without further purification. Reactions were monitored by thin layer chromatography (TLC). TLC was performed with 0.25 mm E. Merck silica plates (60F-254) using short-wave UV light as the visualizing agent and KMnO4, ninhydrin, vanillin, bromocresol green, iodine and heat as developing agents. LC/HRMS was performed on a Thermo Vanquish UHPLC coupled to an Orbitrap Exploris 120 (HRESI) equipped with an Accucore C18 column (100 mm × 2.1 mm), or on an Agilent 1260 Infinity system equipped with Poroshell 120 EC-C18 column (4.6 x 50 mm, 2.7 µm) and an Agilent G6230B TOF LC/MS with water and acetonitrile buffered with 0.1% formic acid as mobile phases. LCMS analysis was performed on an Agilent 1260 Infinity System coupled to an Agilent 6100 Series Single Quadrupole LCMS equipped with Poroshell 120 EC-C18 column (3.0 x 50 mm, 2.7 µm). Preparative HPLC purification was performed on an Agilent 1260 Infinity system equipped with a Zorbax Eclipse XDB-C18 PrepHT column (21.2 x 250 mm, 7 µm) with water and acetonitrile buffered with 0.1% formic acid as mobile phases. NMR spectra were recorded on a Bruker AVANCE AV400 (400 MHz and 101 MHz) or Bruker AVANCE AV600 (600 MHz and 151 MHz) at 23 °C unless otherwise noted. Optical rotations were measured on Autopol IV polarimeter (Rudolph Research Analytical). Sonication was performed using a Qsonica Q500 sonicator. Biochemicals and media components were purchased from standard commercial sources. Expression vectors were obtained via DNA synthesis from Twist Bioscience and were used directly with electrocompetent E. coli BL21(DE3). Electrocompetent *E. coli* BL21(DE3) strains were purchased from Lucigen. All *E. coli* strains generated in this work are stored as glycerol stocks at –80 °C.

Proteins and DNA sequences

Expression vectors for SadA/LasA[8], KDO1[9] and VioC[10,11] were constructed as described in previously reported procedures. For the construction of expression vectors, each of the above sequences was inserted between the NdeI and BamHI restriction sites within the commercial pET28a(+) vector. The resulting expression vector was used directly to transform electrocompetent *E. coli* strain BL21(DE3). Variants were stored as glycerol stocks at –80 °C.

Protein sequence of SadA (NCBI accession code: WP_011660927.1)

MQHTYPAQLMRFGTAARAEHMTIAAAIHALDADEADAIVMDIVPDGERDAWWDDEG
FSSSPFTKNAHHAGIVATSVTLGQLQREQGDKLVSKAAEYFGIACRVNDGLRTTRFVRL
FSDALDAKPLTIGHDYEVEFLLATRRVYEPFEAPFNFAPHCDDVSYGRDTVNWPLKRSF
PRQLGGFLTIQGADNDAGMVMWDNRPESRAALDEMHAEYRETGAIAALERAAKIML
KPQPGQLTLFQSKNLHAIERCTSTRRTMGLFLIHTEDGWRMFD*

DNA sequence of SadA (codon optimized by Genscript)

ATGCAGCACACCTACCCGGCGCAACTGATGCGTTTTGGTACCGCGGCGCGTGCGGA
GCACATGACCATTGCGGCGGCGATTCATGCGCTGGATGCGGATGAAGCGGATGCGA
TCGTGATGGACATTGTTCCGGATGGCGAGCGTGACGCGTGGTGGGACGATGAAGGC
TTCAGCAGCAGCCCGTTTACCAAGAACGCGCACCATGCGGGTATTGTGGCGACCAG
CGTTACCCTGGGTCAGCTGCAACGTGAGCAGGGCGATAAGCTGGTTAGCAAAGCGG
CGGAATATTTTGGTATTGCGTGCCGTGTGAACGACGGCCTGCGTACCACCCGTTTCG
TTCGTCTGTTTAGCGACGCGCTGGATGCGAAGCCGCTGACCATCGGTCACGATTACG
AGGTGGAATTCCTGCTGGCGACCCGTCGTGTTTATGAGCCGTTTGAAGCGCCGTTCA
ACTTTGCGCCGCACTGCGACGATGTGAGCTACGGCCGTGACACCGTTAACTGGCCG
CTGAAACGTAGCTTCCCGCGTCAGCTGGGTGGCTTTCTGACCATTCAGGGTGCGGA
CAACGATGCGGGCATGGTTATGTGGGATAACCGTCCGGAGAGCCGTGCGGCGCTGG
ATGAAATGCATGCGGAGTATCGTGAAACCGGTGCGATTGCGGCGCTGGAGCGTGCG
GCGAAGATTATGCTGAAACCGCAGCCGGGCCAACTGACCCTGTTCCAAAGCAAAA
ACCTGCACGCGATCGAGCGTTGCACCAGCACCCGTCGTACCATGGGTCTGTTCCTG
ATTCACACCGAAGATGGCTGGCGTATGTTTGACTGA

Protein sequence of LasA (NCBI accession code: WP_011660928.1)

MTAIALTEGNDPARSRLEAEIAAFERFAESSTIGRARVERVERFGFVIHPPHANAAHEPRF
DLLFIALTHGNEYAGLPVLNALCRYVEAGVLAPSVSIGFLLGNVDAARRGTRFVERDL
NRTFGRAGRDTADDRRAREMEPVMRRARFCVDLHQTIEPSEAPFFVFNYHPTSLQLAH
AIAPDTPVVTHWGRPFSTASAGGCTSDEFIQQGDGVALSIELGQKGFGLYQIAAGVRVC
LDAITTVSAVVHGASLPPVDACVNPIYTWAHIVPYPDGEVRLDAGLRNFQPIRAGDRLG
TRDGAPLVAGTSGLLLFPKYRRSESEPKPAELYRLLRQPALSELGKGDCLMPQS*

DNA sequence of LasA (codon optimized by Genscript)

ATGACCGCGATTGCGCTGACCGAGGGTAACGACCCGGCGCGTAGCCGTCTGGAGGC
GGAAATCGCGGCGTTCGAGCGTTTTGCGGAAAGCAGCACCATTGGCCGTGCGCGTG
TGGAGCGTGTTGAACGTTTCGGTTTTGTGATCCACCCGCCGCACGCGAACGCGGCG
CATGAGCCGCGTTTTGACCTGCTGTTTATTGCGCTGACCCACGGCAACGAATACGCG
GGTCTGCCGGTTCTGAACGCGCTGTGCCGTTATGTTGAGGCGGGCGTTCTGGCGCC

GAGCGTGAGCATTGGTTTCCTGCTGGGCAACGTTGATGCGGCGCGTCGTGGTACCC
GTTTCGTGGAGCGTGACCTGAACCGTACCTTTGGTCGTGCGGGCCGTGATACCGCG
GACGATCGTCGTGCGCGTGAGATGGAACCGGTGATGCGTCGTGCGCGTTTCTGCGT
TGACCTGCACCAGACCATCGAGCCGAGCGAAGCGCCGTTCTTTGTTTTTAACTATCA
TCCGACCAGCCTGCAACTGGCGCATGCGATTGCGCCGGATACCCCGGTGGTTACCC
ACTGGGGTCGTCCGTTCAGCACCGCGAGCGCGGGTGGCTGCACCAGCGACGAGTT
TATCCAGCAAGGTGATGGCGTGGCGCTGAGCATTGAACTGGGCCAGAAGGGTTTTG
GCCTGTATCAAATTGCGGCGGGTGTGCGTGTTTGCCTGGACGCGATTACCACCGTTA
GCGCGGTGGTTCATGGTGCGAGCCTGCCGCCGGTGGATGCGTGCGTTAACCCGATC
TACACCTGGGCGCACATTGTGCCGTATCCGGACGGCGAAGTTCGTCTGGATGCGGG
TCTGCGTAACTTTCAACCGATTCGTGCGGGTGATCGTCTGGGTACCCGTGATGGTGC
GCCGCTGGTTGCGGGTACCAGCGGTCTGCTGCTGTTTCCGAAGTACCGTCGTAGCG
AGAGCGAACCGAAACCGGCGGAGCTGTATCGTCTGCTGCGTCAGCCGGCGCTGAG
CGAACTGGGTAAAGGCGATTGCCTGATGCCGCAAAGCTAA

Protein sequence of VioC (Uniprot ID: Q6WZB0)

MTESPTTHHGAAPPDSVATPVRPWSEFRLTPAEAAAAAALAARCAQRYDETDGPEFLL
DAPVIAHELPRRLRTFMARARLDAWPHALVVRGNPVDDAALGSTPVHWRTARTPGSR
PLSFLLMLYAGLLGDVFGWATQQDGRVVTDVLPIKGGEHTLVSSSSRQELGWHTEDAF
SPYRADYVGLLSLRNPDGVATTLAGVPLDDLDERTLDVLFQERFLIRPDDSHLQVNNST
AQQGRVEFEGIAQAADRPEPVAILTGHRAAPHLRVDGDFSAPAEGDEEAAAALGTLRK
LIDASLYELVLDQGDVAFIDNRRAVHGRRAFQPRYDGRDRWLKRINITRDLHRSRKAW
AGDSRVLGQR

DNA sequence of VioC (codon optimized by Genscript)

ATGACCGAAAGCCCGACCACCCATCATGGCGCGGCGCCGCCGGATAGCGTGGCGAC
CCCGGTGCGCCCGTGGAGCGAATTTCGCCTGACCCCGGCGGAAGCGGCGGCGGCG
GCGGCGCTGGCGGCGCGCTGCGCGCAGCGCTATGATGAAACCGATGGCCCGGAATT
TCTGCTGGATGCGCCGGTGATTGCGCATGAACTGCCGCGCCGCCTGCGCACCTTTAT
GGCGCGCGCGCGCCTGGATGCGTGGCCGCATGCGCTGGTGGTGCGCGGCAACCCG
GTGGATGATGCGGCGCTGGGCAGCACCCCGGTGCATTGGCGCACCGCGCGCACCCC
GGGCAGCCGCCCGCTGAGCTTTCTGCTGATGCTGTATGCGGGCCTGCTGGGCGATGT
GTTTGGCTGGGCGACCCAGCAGGATGGCCGCGTGGTGACCGATGTGCTGCCGATTA
AAGGCGGCGAACATACCCTGGTGAGCAGCAGCAGCCGCCAGGAACTGGGCTGGCA
TACCGAAGATGCGTTTAGCCCGTATCGCGCGGATTATGTGGGCCTGCTGAGCCTGCG
CAACCCGGATGGCGTGGCGACCACCCTGGCGGGCGTGCCGCTGGATGATCTGGATG
AACGCACCCTGGATGTGCTGTTTCAGGAACGCTTTCTGATTCGCCCGGATGATAGCC
ATCTGCAGGTGAACAACAGCACCGCGCAGCAGGGCCGCGTGGAATTTGAAGGCAT
TGCGCAGGCGGCGGATCGCCCGGAACCGGTGGCGATTCTGACCGGCCATCGCGCGG
CGCCGCATCTGCGCGTGGATGGCGATTTTAGCGCGCCGGCGGAAGGCGATGAAGAA
GCGGCGGCGGCGCTGGGCACCCTGCGCAAACTGATTGATGCGAGCCTGTATGAACT
GGTGCTGGATCAGGGCGATGTGGCGTTTATTGATAACCGCCGCGCGGTGCATGGCCG
CCGCGCGTTTCAGCCGCGCTATGATGGCCGCGATCGCTGGCTGAAACGCATTAACAT
TACCCGCGATCTGCATCGCAGCCGCAAAGCGTGGGCGGGCGATAGCCGCGTGCTGG
GCCAGCGC

Protein sequence of KDO1 (Uniprot accession code: C7QJ42)

MKNLSAYEVYESPKTSGESRTEAVSEAAFESDPEVSAILVLTSSEASTLERVADLVTAHA
LYAAHDFCAQAQLAAAELPSRVVARLQEFAWGDMNEGHLLIKGLPQVRSLPPTPTSNV
HAVAATTPMSRYQALINECVGRMIAYEAEGHGHTFQDMVPSAMSAHSQTSLGSAVELE
LHTEQAFSPLRPDFVSLACLRGDPRALTYLFSARQLVATLTTQEIAMLREPMWTTTVDE
SFLAEGRTFLLGFERGPIPILSGADDDPFIVFDQDLMRGISAPAQELQQTVIRAYYAERVS
HCLAPGEMLLIDNRRAVHGRSIFAPRFDGADRFLSRSFIVADGSRSRHARSSFGRVVSAR
FS

DNA sequence of KDO1 (codon optimized using IDT Codon Optimization Tool)

ATGAAGAACTTATCGGCGTATGAGGTGTACGAATCCCCCAAAACTTCCGGTGAGTCC
CGCACGGAGGCTGTTAGTGAGGCAGCCTTCGAGTCTGACCCCGAAGTAAGCGCTAT
CCTGGTCCTGACGTCGTCGGAAGCGTCAACATTGGAACGTGTTGCCGATTTGGTGA
CAGCACACGCCTTGTATGCTGCCCACGACTTCTGCGCGCAAGCGCAATTGGCCGCC
GCGGAATTGCCGTCACGTGTCGTCGCCCGTTTACAGGAGTTCGCTTGGGGTGACAT
GAACGAAGGTCATTTACTTATTAAGGGGCTGCCTCAGGTACGCAGTTTGCCCCCTAC
GCCTACATCCAACGTTCATGCAGTTGCGGCTACGACGCCAATGTCCCGCTATCAAGC
GTTAATTAACGAGTGTGTCGGCCGTATGATTGCCTATGAAGCTGAGGGCCACGGCCA
CACATTCCAAGATATGGTGCCATCTGCAATGTCCGCTCATTCTCAAACATCGTTAGGC
AGCGCTGTTGAGCTTGAACTTCACACCGAACAAGCTTTTAGCCCACTTCGCCCAGA
TTTCGTTTCGCTTGCCTGTTTGCGCGGCGATCCACGTGCACTTACTTATCTTTTTTCG
GCTCGTCAGTTGGTTGCGACATTAACAACACAGGAGATTGCCATGTTACGTGAGCC
CATGTGGACGACCACAGTTGACGAGAGCTTCTTAGCGGAGGGACGCACTTTTTTGC
TTGGGTTCGAACGCGGGCCTATCCCTATTTATCAGGTGCTGATGATGATCCTTTCAT
CGTCTTTGACCAAGACTTAATGCGTGGAATTTCTGCACCGGCTCAAGAACTGCAAC
AAACGGTCATTCGTGCGTATTACGCCGAGCGTGTAAGCCACTGCTTAGCGCCAGGC
GAGATGCTTTTGATCGACAACCGTCGTGCCGTCCACGGCCGTTCTATCTTTGCGCCT
CGCTTCGACGGCGCTGACCGTTTCCTGTCCCGTTCTTTCATTGTGGCCGACGGATCT
CGCTCGCGTCACGCCCGCTCCTCTTTCGGGCGCGTGGTATCAGCACGCTTTTCA

Synthesis of Starting Material

(*2S,3R*)-2-((tert-butoxycarbonyl)amino)-3-hydroxypentanoic acid (S11)

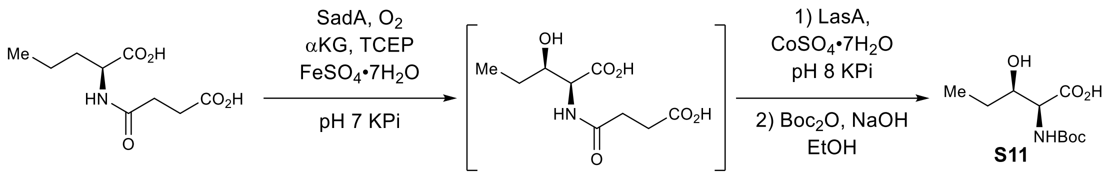

(*2S,3R*)-2-((tert-butoxycarbonyl)amino)-3-hydroxypentanoic acid

L-Succinyl-norvaline (**S11**) was synthesized from *L*-norvaline following a previously published procedure.[12] Following a reported procedure,[13] a glycerol stock of *E. coli* BL21(DE3) cells harboring pET-28a(+)-SadA plasmid was used to inoculate an overnight culture of LB media (5 mL) containing 50 µg/mL kanamycin. 2.0 mL of this culture was used to inoculate 400 mL TB media containing 50 µg/mL kanamycin in a 2 L non-beveled Erlenmeyer flask. The cultures were shaken at 250 rpm/37 °C for 2.5 h or until an OD_{600} = 0.6 was reached. The culture was cooled on ice (15 min), induced by adding IPTG to final concentration of 25 µM, then allowed to continue shaking at 250 rpm/23 °C for another 21 h. Cells were harvested by centrifugation (4 °C, 15 min, 4200 rpm), then resuspended in 50 mM pH = 7 KPi buffer (ca. 120 mL) to a final OD_{600} = 30. The cell suspension was lysed at 50% amplitude for 5 min (1 s on, 4 s off) in an ice bath. The cell debris was pelleted by centrifugation (4 °C, 15 min, 4200 rpm), and the clarified lysate supernatant was diluted 1:1 with 50 mM pH = 7 KPi and added to 3X500 mL non-beveled Erlenmeyer flask (≥80% headspace) each containing 80 mL of diluted lysate, *L*-norvaline (174 mg, 0.8 mmol, 1.00 equiv., 10 mM final concentration), α-ketoglutaric acid (disodium salt dihydrate, 271 mg, 1.20 mmol, 1.50 equiv.), TCEP (46.0 mg, 0.160 mmol, 0.20 equiv.) and $FeSO_4$•$7H_2O$ (44.0 mg, 0.160 mmol, 0.20 equiv.). The reaction mixture was then shaken at 250 rpm/30 °C in open Erlenmeyer flasks overnight or until completion by LCMS. The reaction was then quenched by addition of 6 M HCl to final pH = 2 and centrifuged (4 °C, 15 min, 4200 RPM). The supernatant was concentrated under a gentle stream of compressed air to a final volume of 5 mL and centrifuged. The resulting pellet was resuspended in 1 mL H_2O, centrifuged, and the supernatants were combined and purified by C18 flash chromatography (100% H_2O for 1 CV then 0-25% MeOH over 10 CV, then 5 CV to 100% MeOH). The product-containing fractions were combined and concentrated under a gentle stream of compressed air to afford 3-OH-*L*-succinyl-norvaline that was used directly in the next step.

Following a reported procedure,[13] a glycerol stock of *E. coli* BL21(DE3) cells harboring pET-28a(+)-LasA plasmid was used to inoculate an overnight culture of LB media (4 mL) containing 50 µg/mL kanamycin. 2 mL of this culture was used to inoculate 400 mL TB media containing 50 µg/mL kanamycin in a 1 L non-beveled Erlenmeyer flasks. The cultures were shaken at 250 rpm/37 °C for 2.5 h or until an OD_{600} = 0.6 was reached. The culture was cooled on ice (15 min), induced by adding IPTG to final concentration of 25 µM, then allowed to continue shaking at 250 rpm/23 °C for another 20 h. Cells were harvested by centrifugation (4 °C, 15 min, 4200 rpm), then resuspended in 50 mM pH = 8 KPi buffer (ca. 195 mL) to a final OD_{600} = 20. The combined cell suspensions were lysed by sonication at 50% amplitude for 2 x 3 min (1 s on, 4 s off) in an ice bath. The cell debris was pelleted by centrifugation (4 °C, 15 min, 4200 rpm), and 167 mL of the clarified lysate supernatant was added to a non-beveled 1 L Erlenmeyer flask containing 3-OH-*L*-succinyl-norvaline (390 mg, 1.67 mmol, 1.00 equiv, 10

mM final concentration) and $CoSO_4 \cdot 7H_2O$ (4.70 mg, 0.017 mmol, 0.01 equiv). The mixture was shaken at 200 rpm/20 °C overnight or until completion by LCMS. The reaction mixture was basified to pH = 11 with 6M NaOH and a solution of Boc_2O (1.46 g, 6.68 mmol, 4.00 equiv.) in EtOH (84 mL, 2:1 v/v) was added slowly at room temperature. After 30 minutes, the pH was adjusted to pH = 10 with 6M NaOH and the reaction was allowed to stir 4 hours. At this point pH was adjusted to pH = 10 with 6M NaOH and a solution of Boc_2O (730 mg, 3.34 mmol, 2.00 equiv.) in 1 mL of EtOH was slowly added. The reaction was then allowed to stir overnight at room temperature. Ethanol was removed from the reaction mixture under vacuum and the aqueous phase was adjusted to pH = 1 with 6M HCl and diluted with EtOAc (80 mL). The aqueous layer was separated and extracted with an additional 3X50 mL EtOAc. The combined organic layers were washed with brine and dried over $MgSO_4$, filtered and concentrated under vacuum. (*2S,3R*)-2-((tert-butoxycarbonyl)amino)-3-hydroxypentanoic acid (**S11**) (250 mg, 1.07 mmol, 45% yield over the three steps) was obtained as a colorless sticky oil after purification by FCC on silica gel (DCM/MeOH/AcOH 20:1:0.1).

$[\alpha]_D^{20}$ = +4.9 (c = 0.48, MeOH).

NMR spectroscopic data was consistent with the values reported in the literature for the racemic compound.[14]

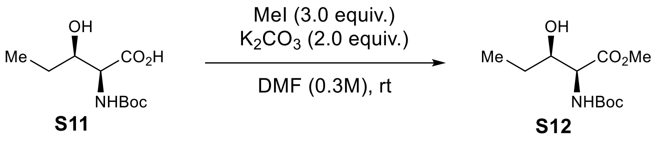

Methyl (*2S,3R*)-2-((tert-butoxycarbonyl)amino)-3-hydroxypentanoate

Following a reported procedure,[15] an oven-dried round bottom flask was charged with (*2S,3R*)-2-((tert-butoxycarbonyl)amino)-3-hydroxypentanoic acid (**S11**) (230 mg, 0.99 mmol, 1.0 equiv.), K_2CO_3 (273 mg, 1.97 mmol, 2.0 equiv.) and dry DMF (3.2 mL). MeI (184 µL, 2.96 mmol, 3.0 equiv.) was then added dropwise and the reaction mixture was let stirring at room temperature overnight. The crude mixture was diluted with 20 mL of sat. $NaHCO_3$, extracted with EtOAc, washed with brine, LiCl (5% w), dried over $MgSO_4$, filtered and concentrated under reduced pressure. Methyl (*2S,3R*)-2-((tert-butoxycarbonyl)amino)-3-hydroxypentanoate (**S12**) (156 mg, 0.63 mmol, 64% yield) was obtained as a colorless oil after purification by FCC on silica gel (Hexane/EtOAc 4:1 to 7:3).

Rf (Hexane/EtOAc 4:1): 0.1.

^{1}H NMR (600 MHz, $CDCl_3$) δ 5.31-5.26 (m, 1H), 4.34 (d, *J* = 9.0 Hz, 1H), 3.99 (d, *J* = 7.2 Hz, 1H), 3.77 (s, 3H), 1.88 (br s, 1H), 1.61-1.50 (m, 2H), 1.45 (s, 9H), 0.99 (t, *J* = 7.4 Hz, 3H).

^{13}C NMR (151 MHz, $CDCl_3$) δ 172.4, 156.2, 80.2, 73.7, 57.2, 52.7, 28.4, 26.9, 10.2.

HRMS (ESI/QTOF) m/z: $[M + Na]^+$ Calcd for $C_{11}H_{21}NNaO_5^+$ Calcd for 270.1317; Found 270.1321.

$[\alpha]_D^{20}$ = -8.8 (c = 0.36, MeOH).

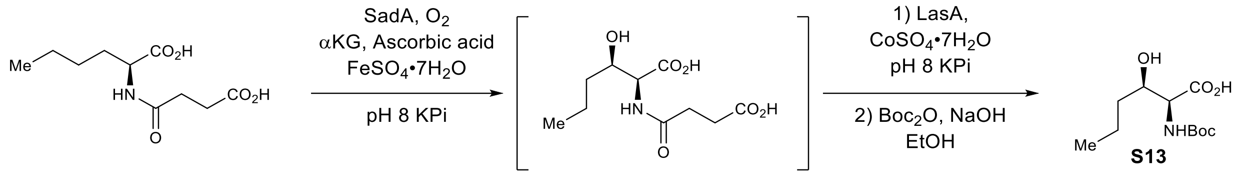

(*2S,3R*)-2-((tert-butoxycarbonyl)amino)-3-hydroxyhexanoic acid

L-Succinyl-norleucine (**S13**) was synthesized from *L*-norleucine following a previously published procedure.[12] Following a reported procedure,[8] a glycerol stock of *E. coli* BL21(DE3) cells harboring pET-28a(+)-SadA plasmid was used to inoculate an overnight culture of LB media (5 mL) containing 50 µg/mL kanamycin. 4.0 mL of this culture was used to inoculate 2X400 mL TB media containing 50 µg/mL kanamycin in two 2 L non-beveled Erlenmeyer flask. The cultures were shaken at 250 rpm/37 °C for 2.5 h or until an OD_{600} = 0.6 was reached. The cultures were cooled on ice (15 min), induced by adding IPTG to final concentration of 25 µM, then allowed to continue shaking at 250 rpm/23 °C for another 21 h. Cells were harvested by centrifugation (4 °C, 15 min, 4200 rpm), then resuspended in 50 mM pH = 8 KPi buffer (ca. 180 mL) to a final OD_{600} = 30. The cell suspension was lysed at 50% amplitude for 5 min (1 s on, 4 s off) in an ice bath. The cell debris was pelleted by centrifugation (4 °C, 15 min, 4200 rpm), and the clarified lysate supernatant was divided in 3X60 mL and added to three non-beveled Erlenmeyer flask (≥80% headspace) containing *L*-norleucine (139 mg, 0.60 mmol, 1.00 equiv., 10 mM final concentration), α-ketoglutaric acid (disodium salt dihydrate, 204 mg, 0.90 mmol, 1.50 equiv.), ascorbic acid (106 mg, 0.6 mmol, 1.00 equiv.) and $FeSO_4•7H_2O$ (17 mg, 0.06 mmol, 0.10 equiv.). The reaction mixture was then shaken at 200 rpm/23 °C in an open Erlenmeyer flask overnight or until completion by LCMS. The reaction was then quenched by addition of 6 M HCl to final pH = 2 and centrifuged (4 °C, 15 min, 4200 RPM). The supernatant was concentrated under a gentle stream of compressed air to a final volume of 5 mL and centrifuged. The resulting pellet was resuspended in 1 mL H_2O, centrifuged, and the supernatants were combined and purified by C18 flash chromatography (100% H_2O for 1 CV then 0-25% MeOH over 10 CV, then 5 CV to 100% MeOH). The product-containing fractions were combined and concentrated under a gentle stream of compressed air to afford 3-OH-*L*-succinyl-norleucine that was used directly in the next step.

Following a reported procedure,[8] a glycerol stock of *E. coli* BL21(DE3) cells harboring pET-28a(+)-LasA plasmid was used to inoculate an overnight culture of LB media (4 mL) containing 50 µg/mL kanamycin. 2 mL of this culture was used to inoculate 400 mL TB media containing 50 µg/mL kanamycin in a 2 L non-beveled Erlenmeyer flasks. The cultures were shaken at 250 rpm/37 °C for 2.5 h or until an OD_{600} = 0.6 was reached. The culture was cooled on ice (15 min), induced by adding IPTG to final concentration of 25 µM, then allowed to continue shaking at 250 rpm/23 °C for another 20 h. Cells were harvested by centrifugation (4 °C, 15 min, 4200 rpm), then resuspended in 50 mM pH = 8 KPi buffer (ca. 100 mL) to a final OD_{600} = 10. The combined cell suspensions were lysed by sonication at 50% amplitude for 2 x 3 min (1 s on, 4 s off) in an ice bath. The cell debris was pelleted by centrifugation (4 °C, 15 min, 4200 rpm), and 60 mL of the diluted clarified lysate supernatant (1:1 dilution with 50 mM pH = 8 KPi buffer) was added to a non-beveled 500 mL Erlenmeyer flask containing the crude material (150 mg, 0.61 mmol, 1.00 equiv, 10 mM final concentration) and CoSO4•7H2O (1.70 mg, 0.006 mmol, 0.01 equiv). The mixture was shaken at 200 rpm/20 °C overnight or until completion by LCMS. The reaction mixture was basified to pH = 11 with 6M NaOH and a

solution of Boc_2O (530 mg, 2.4 mmol, 4.00 equiv.) in EtOH (30 mL, 2:1 v/v) was added slowly at room temperature. After 30 minutes, the pH was adjusted to pH = 10 with 6M NaOH and the reaction was allowed to stir 4 hours. At this point pH was adjusted to pH = 10 with 6M NaOH and a solution of Boc_2O (265 mg, 1.2 mmol, 2.00 equiv.) in 1 mL of EtOH was slowly added. The reaction was then allowed to stir overnight at room temperature. Ethanol was removed from the reaction mixture under vacuum and the aqueous phase was adjusted to pH = 1 with 6M HCl and diluted with EtOAc (80 mL). The aqueous layer was separated and extracted with an additional 3X50 mL EtOAc. The combined organic layers were washed with brine and dried over $MgSO_4$, filtered and concentrated under vacuum. (*2S,3R*)-2-((tert-butoxycarbonyl)amino)-3-hydroxyhexanoic acid (**S13**) (99 mg, 0.40 mmol, 43% yield over the three steps) was obtained as a clear hygroscopic foam after purification by FCC on silica gel (DCM/MeOH/AcOH 20:1:0.1).

NMR spectroscopic data was consistent with the values reported in the literature.[8]

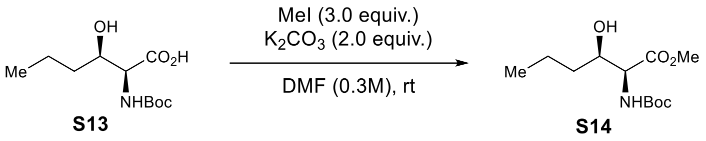

Methyl (*2S,3R*)-2-((tert-butoxycarbonyl)amino)-3-hydroxyhexanoate

Following a reported procedure,[15] an oven-dried round bottom flask was charged with (*2S,3R*)-2-((tert-butoxycarbonyl)amino)-3-hydroxyhexanoic acid (**S13**) (190 mg, 0.77 mmol, 1.0 equiv.), K_2CO_3 (212 mg, 1.54 mmol, 2.0 equiv.) and dry DMF (2.6 mL). MeI (143 µL, 2.3 mmol, 3.0 equiv.) was then added dropwise and the reaction mixture was let stirring at room temperature overnight. The crude mixture was diluted with 20 mL of sat. $NaHCO_3$, extracted with EtOAc, washed with brine, LiCl (5% w), dried over $MgSO_4$, filtered and concentrated under reduced pressure. Methyl (*2S,3R*)-2-((tert-butoxycarbonyl)amino)-3-hydroxyhexanoate (**S14**) (166 mg, 0.64 mmol, 82% yield) was obtained as a colorless oil after purification by FCC on silica gel (Hexane/EtOAc 5:1 to 3:1).

Rf (Hexane/EtOAc 7:3): 0.21.

^{1}H NMR (600 MHz, $CDCl_3$) δ 5.30 (d, *J* = 9.3 Hz, 1H), 4.33-4.28 (m, 1H), 4.09 (d, *J* = 7.1 Hz, 1H), 3.77 (s, 3H), 1.93 (br s, 1H), 1.53-1.46 (m, 3H), 1.45 (s, 9H), 1.42-1.34 (m, 1H), 0.94 (t, *J* = 7.1 Hz, 3H).

^{13}C NMR (151 MHz, $CDCl_3$) δ 172.4, 156.2, 80.2, 71.9, 57.6, 52.6, 35.9, 28.4, 18.9, 14.0.

HRMS (ESI/QTOF) m/z: $[M + Na]^+$ Calcd for $C_{12}H_{23}NNaO_5^+$ 284.1474; Found 284.1475.

$[\alpha]_D^{20}$ = -1.3 (c = 0.55, MeOH).

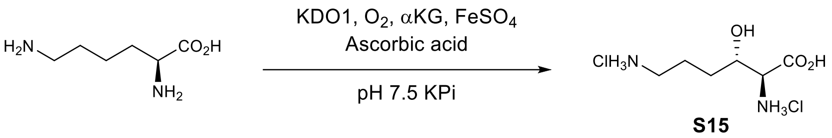

(*1S,2S*)-1-carboxy-2-hydroxypentane-1,5-diaminium chloride

Following a reported procedure,[16] an overnight culture of recombinant *E. coli* BL21(DE3) cells harboring pET-28a(+)-KDO1 and pGro7 plasmids was used to inoculate 200 mL TB media (in a 1 L non-beveled Erlenmeyer flask) containing 25 μg/mL chloroamphenicol and 50 μg/mL kanamycin. The cultures were shaken at 250 rpm at 37 °C until an optical density of approximately 1.5 was reached (~3.3 h). The cultures were cooled on ice (20 min) and then induced by adding IPTG and L-arabinose to final concentrations of 0.025 mM and 1 mg/mL, respectively. The cultures were allowed to continue for another 20 hours at 23 °C and shaking at 250 rpm. Cell were harvested by centrifugation (4 °C, 15 min, 3,000xg), and the cell pellets were resuspended to OD_{600} = 20 in 50 mM kPi (pH = 7.50). This procedure yielded ca. 150 mL of cell suspension. Cells were disrupted by sonication (3x1 min, 50% duty cycle) and pelleted by centrifugation at 4,000 rpm for 15 min at 4 °C. Two 500 mL Erlenmeyer flasks were each charged with *L*-lysine (385 mg, 2.63 mmol, 1.00 equiv), *L*-ascorbic acid (462 mg, 2.63 mmol, 1.00 equiv), α-ketoglutaric acid (disodium salt dihydrate, 1.19 g, 5.25 mmol, 2.0 equiv), and $FeSO_4$ (heptahydrate, 37 mg, 1.32 μmol, 0.05 equiv), giving a total of 5.25 mmol of *L*-lysine for hydroxylation. After addition of clarified KDO1 lysate (75 mL to each flask), the flasks were shaken at 20 °C for 18 h at 250 rpm. The reaction was quenched by addition of 1 M HCl (10 mL to each flask) and centrifuged at 4,000 rpm for 15 min at 4 °C. The combined supernatant was collected, concentrated to dryness and was used for the next step without further purification.

NMR spectroscopic data was consistent with the values reported in the literature.[16]

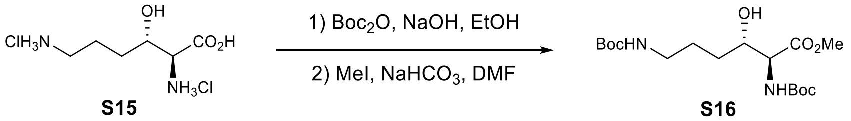

Methyl (*2S,3S*)-2,6-bis((tert-butoxycarbonyl)amino)-3-hydroxyhexanoate

Following a reported procedure,[16] The crude material from the previous biotransformation (5.25 mmol, 1.0 equiv.) was resuspended in 1 M NaOH (26.3 mL) and treated dropwise with a solution of Boc_2O (4.58 g, 21 mmol, 4.0 equiv.) in ethanol (5.3 mL) at 0 °C. The reaction was stirred at 0 °C for 3 hours and then acidified to pH = 2–3 with 1 N HCl. The reaction was diluted with water (25 mL) and the aqueous phase was extracted with EtOAc (3X25 mL). The organic layer was washed with brine, dried over anhydrous $MgSO_4$, and concentrated in vacuo to give a yellow oil, which was further dissolved in dry DMF (26.3 mL). Solid $NaHCO_3$ (2.21 g, 26.3 mmol, 5.0 equiv.) and methyl iodide (0.49 mL, 7.9 mmol, 1.5 equiv.) were subsequently added and the reaction mixture was stirred overnight. The reaction was diluted with water (150 mL) and the aqueous phase was extracted with EtOAc (3X50 mL). The combined organic phase was washed with water, followed by brine, and dried over anhydrous $MgSO_4$. Methyl (*2S,3S*)-2,6-bis((tert-butoxycarbonyl)amino)-3-hydroxyhexanoate (**S16**) (206 mg, 0.55 mmol,

10% yield over the three steps) was obtained as a yellowish sticky oil after purification by FCC on silica gel (Hexane/EtOAc 3:1 to 1:1).

Rf (Hexane/EtOAc 1:1): 0.21.

^{1}H NMR (600 MHz, $CDCl_3$) δ 5.47 (d, J = 7.8 Hz, 1H), 4.62 (s, 1H), 4.39-4.32 (m, 1H), 3.95-3.86 (m, 1H), 3.77 (s, 3H), 3.22-3.06 (m, 2H), 1.72-1.64 (m, 1H), 1.63-1.53 (m, 1H), 1.44 (m, 20H).[1]

^{13}C NMR (151 MHz, $CDCl_3$) δ 171.2, 156.4, 156.2, 80.7, 79.4, 73.0, 58.7, 52.7, 40.3, 30.2, 28.6, 28.4, 26.8.

HRMS (ESI/QTOF) m/z: $[M + Na]^+$ Calcd for $C_{17}H_{32}N_2NaO_7^+$ Calcd for 399.2107; Found 399.2118.

$[\alpha]_D^{20}$ = -7.5 (c = 0.36, MeOH).

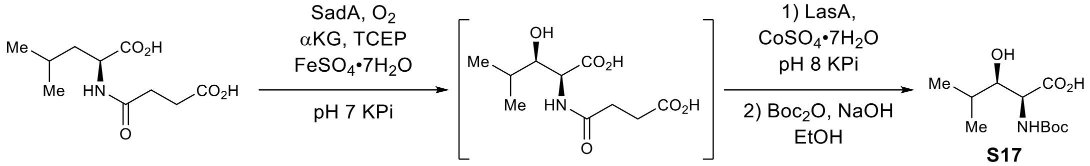

(*2S,3R*)-2-(3-carboxypropanamido)-3-hydroxy-4-methylpentanoic acid

L-Succinyl-leucine (**S17**) was synthesized from *L*-leucine following a previously published procedure.[12] Following a reported procedure,[13] a glycerol stock of *E. coli* BL21(DE3) cells harboring pET-28a(+)-SadA plasmid was used to inoculate an overnight culture of LB media (5 mL) containing 50 μg/mL kanamycin. 1.0 mL of this culture was used to inoculate 200 mL TB media containing 50 μg/mL kanamycin in a 1 L non-beveled Erlenmeyer flask. The cultures were shaken at 250 rpm/37 °C for 2.5 h or until an OD_{600} = 0.6 was reached. The culture was cooled on ice (15 min), induced by adding IPTG to final concentration of 25 μM, then allowed to continue shaking at 250 rpm/23 °C for another 21 h. Cells were harvested by centrifugation (4 °C, 15 min, 4200 rpm), then resuspended in 50 mM pH = 7 KPi buffer (ca. 45 mL) to a final OD_{600} = 30. The cell suspension was lysed at 50% amplitude for 5 min (1 s on, 4 s off) in an ice bath. The cell debris was pelleted by centrifugation (4 °C, 15 min, 4200 rpm), and the clarified lysate supernatant was diluted 1:1 with 50 mM pH = 7 KPi and added to a non-beveled Erlenmeyer flask (≥80% headspace) containing *L*-leucine (208 mg, 0.90 mmol, 1.00 equiv., 10 mM final concentration), α-ketoglutaric acid (disodium salt dihydrate, 305 mg, 1.35 mmol, 1.50 equiv.), TCEP (52.0 mg, 0.180 mmol, 0.20 equiv.) and $FeSO_4•7H_2O$ (50.0 mg, 0.180 mmol, 0.20 equiv.). The reaction mixture was then shaken at 250 rpm/30 °C in an open Erlenmeyer flask overnight or until completion by LCMS. The reaction was then quenched by addition of 6 M HCl to final pH = 2 and centrifuged (4 °C, 15 min, 4200 RPM). The supernatant was concentrated under a gentle stream of compressed air to a final volume of 5 mL and centrifuged. The resulting pellet was resuspended in 1 mL H_2O, centrifuged, and the

supernatants were combined and purified by C18 flash chromatography (100% H_2O for 1 CV then 0-25% MeOH over 10 CV, then 5 CV to 100% MeOH). The product-containing fractions were combined and concentrated under a gentle stream of compressed air to afford 3-OH-*L*-succinyl-leucine that was used directly in the next step.

Following a reported procedure,[13] a glycerol stock of *E. coli* BL21(DE3) cells harboring pET-28a(+)-LasA plasmid was used to inoculate an overnight culture of LB media (4 mL) containing 50 µg/mL kanamycin. 1 mL of this culture was used to inoculate 200 mL TB media containing 50 µg/mL kanamycin in a 1 L non-beveled Erlenmeyer flasks. The cultures were shaken at 250 rpm/37 °C for 2.5 h or until an OD_{600} = 0.6 was reached. The culture was cooled on ice (15 min), induced by adding IPTG to final concentration of 25 µM, then allowed to continue shaking at 250 rpm/23 °C for another 20 h. Cells were harvested by centrifugation (4 °C, 15 min, 4200 rpm), then resuspended in 50 mM pH = 8 KPi buffer (ca. 100 mL) to a final OD_{600} = 20. The combined cell suspensions were lysed by sonication at 50% amplitude for 2 x 3 min (1 s on, 4 s off) in an ice bath. The cell debris was pelleted by centrifugation (4 °C, 15 min, 4200 rpm), and 81 mL of the clarified lysate supernatant was added to a non-beveled 500 mL Erlenmeyer flask containing the crude residue (201 mg, 0.81 mmol, 1.00 equiv, 10 mM final concentration) and CoSO4•7H2O (2.30 mg, 0.008 mmol, 0.01 equiv). The mixture was shaken at 200 rpm/20 °C overnight or until completion by LCMS. The reaction mixture was basified to pH = 11 with 6M NaOH and a solution of Boc_2O (707 mg, 3.24 mmol, 4.00 equiv.) in EtOH (40.5 mL, 2:1 v/v) was added slowly at room temperature. After 30 minutes, the pH was adjusted to pH = 10 with 6M NaOH and the reaction was allowed to stir 4 hours. At this point pH was adjusted to pH = 10 with 6M NaOH and a solution of Boc_2O (354 mg, 1.62 mmol, 2.00 equiv.) in 1 mL of EtOH was slowly added. The reaction was then allowed to stir overnight at room temperature. Ethanol was removed from the reaction mixture under vacuum and the aqueous phase was adjusted to pH = 1 with 6M HCl and diluted with EtOAc (80 mL). The aqueous layer was separated and extracted with an additional 3X50 mL EtOAc. The combined organic layers were washed with brine and dried over $MgSO_4$, filtered and concentrated under vacuum. (2S,3R)-2-(3-carboxypropanamido)-3-hydroxy-4-methylpentanoic acid (**S17**) (153 mg, 0.62 mmol, 68% yield over the three steps) was obtained as a colorless sticky oil after purification by FCC on silica gel (DCM/MeOH/AcOH 20:1:0.1).

NMR spectroscopic data was consistent with the values reported in the literature.[17]

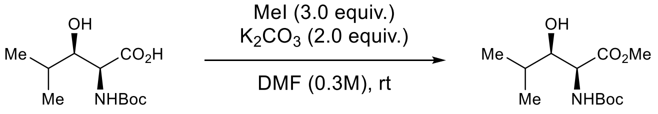

S17 **S18**

Methyl (*2S,3R*)-2-((tert-butoxycarbonyl)amino)-3-hydroxy-4-methylpentanoate

Following a reported procedure,[15] an oven-dried round bottom flask was charged with (2S,3R)-2-(3-carboxypropanamido)-3-hydroxy-4-methylpentanoic acid (**S17**) (200 mg, 0.81 mmol, 1.0 equiv.), K_2CO_3 (224 mg, 1.62 mmol, 2.0 equiv.) and dry DMF (2.6 mL). MeI (151 µL, 2.4 mmol, 3.0 equiv.) was then added dropwise and the reaction mixture was let stirring at room temperature overnight. The crude mixture was diluted with 20 mL of sat. $NaHCO_3$, extracted with EtOAc, washed with brine, LiCl (5% w), dried over $MgSO_4$, filtered and concentrated

under reduced pressure. Methyl (2S,3R)-2-((tert-butoxycarbonyl)amino)-3-hydroxy-4-methylpentanoate (**S18**) (161 mg, 0.62 mmol, 76% yield) was obtained as a colorless oil after purification by FCC on silica gel (Hexane/EtOAc 8:2).

Rf (Hexane/EtOAc 8:2): 0.19.

^{1}H NMR (600 MHz, $CDCl_3$) δ 5.30-5.25 (m, 1H), 4.47 (d, J = 9.1 Hz, 1H), 3.76 (s, 3H), 3.68 (d, J = 8.9 Hz, 1H), 1.99 (br s, 1H), 1.78-1.72 (m, 1H), 1.45 (s, 9H), 1.02 (d, J = 6.7 Hz, 3H), 0.96 (d, J = 6.7 Hz, 3H).

^{13}C NMR (151 MHz, $CDCl_3$) δ 172.9, 156.2, 80.2, 55.8, 52.6, 30.9, 29.8, 28.4, 19.1, 19.0.

HRMS (ESI/QTOF) m/z: $[M + Na]^+$ Calcd for $C_{12}H_{23}NNaO_5^+$ 284.1474; Found 284.1476.

$[\alpha]_D^{20}$ = -5.3 (c = 0.52, MeOH).

2.2 Preparation of Aryl Bromides

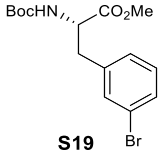

S19

methyl (*S*)-3-(3-bromophenyl)-2-((tert-butoxycarbonyl)amino)propanoate

Following a modified procedure,[18] (*S*)-2-Amino-3-(3-bromophenyl)propanoic acid (20 mmol, 5.16 g, 1.0 equiv) was dissolved in anhydrous methanol (20 mL, 1 M) and cooled to 0 °C. Thionyl chloride (25 mmol, 1.8 mL, 1.2 equiv) was added dropwise. The reaction mixture was warmed to room temperature, then refluxed for 1.5 hours. The crude reaction mixture was concentrated to yield the crude methyl ester. This was dissolved in MeCN:H_2O (85:15, 44 mL, 0.5 M), and triethylamine (44 mmol, 6 mL, 2.2 equiv) was added. The reaction mixture was cooled to 0 °C. Then, $(Boc)_2O$ (24 mmol, 5.2 g, 1.2 equiv) was added in three portions. This was warmed to room temperature and stirred for 12 hours. Upon reaction completion, the reaction mixture was concentrated, then partitioned between EtOAc and water. The aqueous layer was extracted thrice with EtOAc, then the combined extracts were washed with brine, dried over sodium sulfate, and concentrated *in vacuo*. The resulting crude oil was purified by silica gel column chromatography (10% EtOAc in hexanes) to yield the title compound as a white powder (4.95 g, 69% yield, two steps). The physical properties and ^{1}H NMR data were consistent with reported values.[19]

R_f = 0.15 in 10% EtOAc in hexanes

^{1}H NMR (400 MHz, $CDCl_3$) δ 7.37 (d, J = 7.8 Hz, 1H), 7.27 (s, 1H), 7.16 (dd, J = 7.8, 7.8 Hz, 1H), 7.06 (d, J = 7.6 Hz, 1H), 5.01 (d, J = 8.2 Hz, 1H), 4.62 – 4.49 (m, 1H), 3.73 (s, 3H), 3.11 (dd, J = 13.8, 5.8 Hz, 1H), 2.99 (dd, J = 13.8, 6.3 Hz, 1H), 1.42 (s, 9H).

^{13}C NMR (101 MHz, $CDCl_3$) δ 172.1, 155.1, 138.6, 132.6, 130.3, 130.2, 128.0, 122.6, 80.3, 54.4, 52.5, 38.1, 28.4.

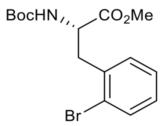

S20

methyl (*S*)-3-(2-bromophenyl)-2-((tert-butoxycarbonyl)amino)propanoate

Following a modified procedure,[18] (*S*)-2-Amino-3-(2-bromophenyl)propanoic acid (20 mmol, 5.0 g, 1.0 equiv) was dissolved in anhydrous methanol (13 mL) and cooled to 0 °C. Thionyl chloride (25 mmol, 1.8 mL, 1.2 equiv) was added dropwise. The reaction mixture was warmed to room temperature, then refluxed until consumption of starting material was observed. The crude reaction mixture was concentrated to yield the crude methyl ester. This was dissolved in MeCN:H_2O (85:15, 44 mL, 0.5 M), and triethylamine (43 mmol, 6 mL, 2.2 equiv) was added. The reaction mixture was cooled to 0 °C. Then, $(Boc)_2O$ (23 mmol, 5.1 g, 1.2 equiv) was added in three portions. This was warmed to room temperature and stirred for 12 hours. Upon reaction completion, the reaction mixture was concentrated, then partitioned between EtOAc and water. The aqueous layer was extracted thrice with EtOAc, then the combined extracts were washed with brine, dried over sodium sulfate, and concentrated *in vacuo*. The resulting crude oil was purified by silica gel column chromatography (10% EtOAc in hexanes) to yield the title compound as a white powder (4.37 g, 63% yield, two steps). The physical properties and ^{1}H NMR data were consistent with reported values.[20]

R_f = 0.6 in 25% EtOAc in hexanes

^{1}H NMR (400 MHz, $CDCl_3$) δ 7.55 (dd, *J* = 8.0, 1.3 Hz, 1H), 7.25 – 7.17 (m, 2H), 7.11 (dd, *J* = 7.6, 7.6 Hz, 1H), 5.05 (d, *J* = 8.6 Hz, 1H), 4.73 – 4.58 (m, 1H), 3.72 (s, 3H), 3.30 (dd, *J* = 13.8, 5.8 Hz, 1H), 3.11 (dd, *J* = 13.8, 8.1 Hz, 1H), 1.38 (s, 9H).

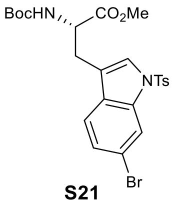

S21

methyl (S)-3-(6-bromo-1-tosyl-1H-indol-3-yl)-2-((tert-butoxycarbonyl)amino)propanoate

Following a modified procedure,[4] a 100-mL rbf was charged with a stir bar, methyl (S)-3-(6-bromo-1H-indol-3-yl)-2-((tert-butoxycarbonyl)amino)propanoate (1.5 mmol, 592 mg, 1.0 equiv), hydrogen tetra(but-1-yl)ammonium sulphate (1.5 mmol, 506 mg, 1.0 equiv), and NaOH (7.45 mmol, 298 mg, 5.0 equiv). This was fitted with a condenser, and anhydrous DCM (6 mL, 0.2 M) was added. This was warmed to reflux and stirred for 20 minutes. This was allowed to cool to room temperature, then p-tosyl-Cl (4.47 mmol, 852 mg, 3.0 equiv) was added. This was heated to reflux and stirred for an additional 3 hours. Upon consumption of starting material, the reaction mixture was diluted with EtOAc, then washed with saturated sodium bicarbonate. This was purified by silica gel column chromatography (25% EtOAc in hexanes) to yield the title substrate as a white powder (183 mg, 22% yield).

R_f = 0.45 in 25% EtOAc in hexanes

^{1}H NMR (400 MHz, $CDCl_3$) δ 8.13 (s, 1H), 7.72 (d, *J* = 8.3 Hz, 2H), 7.37 – 7.27 (m, 4H), 7.25 (d, *J* = 7.7 Hz, 3H), 5.05 (d, *J* = 7.8 Hz, 1H), 4.65 – 4.54 (m, 1H), 3.63 (s, 3H), 3.21 (dd, *J* = 14.8, 5.8 Hz, 1H), 3.11 (dd, *J* = 15.2, 5.3 Hz, 1H), 2.36 (s, 3H), 1.44 (s, 9H).

^{13}C NMR (101 MHz, $CDCl_3$) δ 172.0, 155.1, 145.4, 135.8, 135.1, 130.2, 129.9, 126.9, 126.7, 125.0, 120.8, 118.7, 117.3, 116.9, 80.4, 53.7, 52.5, 28.4, 27.9, 21.7.

HRMS (+ APCI) calculated for $C_{24}H_{27}O_6N_2{}^{79}Br^{32}S$ [M+H] 550.0768, found 550.0765.

$[\alpha]_D^{22}$ = +41.1 (c = 0.78, MeOH).

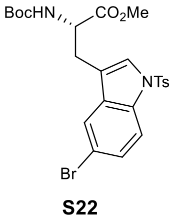

S22

methyl (*S*)-3-(5-bromo-1-tosyl-1H-indol-3-yl)-2-((tert-butoxycarbonyl)amino)propanoate

Following a modified procedure,[4] a 25-mL rbf was charged with a stir bar, methyl (*S*)-3-(4-bromo-1H-indol-3-yl)-2-((tert-butoxycarbonyl)amino)propanoate (0.8 mmol, 333 mg, 1.0 equiv), hydrogen tetra(but-1-yl)ammonium sulphate (0.8 mmol, 285 mg, 1.0 equiv), and NaOH (4.2 mmol, 168 mg, 5.0 equiv). This was fitted with a condenser, and anhydrous DCM (4 mL, 0.2 M) was added. This was warmed to reflux and stirred for 20 minutes. This was allowed to cool to room temperature, then p-tosyl-Cl (2.5 mmol, 479 mg, 3.0 equiv) was added. This was heated to reflux and stirred for an additional 3 hours. Upon consumption of starting material, the reaction mixture was diluted with EtOAc, then washed with saturated sodium bicarbonate. This was purified by silica gel column chromatography (25% EtOAc in hexanes) to yield the title substrate as a white powder (254 mg, 52% yield).

R_f = 0.45 in 25% EtOAc in hexanes

^{1}H NMR (400 MHz, $CDCl_3$) δ 7.82 (d, *J* = 8.8 Hz, 1H), 7.69 (d, *J* = 8.3 Hz, 2H), 7.56 (d, *J* = 1.9 Hz, 1H), 7.38 (dd, *J* = 8.8, 1.9 Hz, 1H), 7.34 (s, 1H), 7.23 (d, *J* = 8.1 Hz, 2H), 5.07 (d, *J* = 7.6 Hz, 1H), 4.62 (dd, *J* = 6.5, 4.8 Hz, 1H), 3.68 (s, 3H), 3.22 (dd, *J* = 14.6, 5.3 Hz, 1H), 3.11 (dd, *J* = 14.9, 5.2 Hz, 1H), 2.35 (s, 3H), 1.46 (s, 9H).

^{13}C NMR (101 MHz, $CDCl_3$) δ 171.8, 145.4, 135.0, 133.9, 132.8, 130.1, 127.9, 126.9, 125.9, 122.5, 117.0, 115.3, 80.4, 53.7, 52.6, 28.5, 27.9, 21.7.

HRMS (+ APCI) calculated for $C_{24}H_{27}O_6N_2{}^{79}Br^{32}S$ [M+H] 550.0768, found 550.0765.

$[\alpha]_D^{23}$= +43.9 (c = 0.67, MeOH).

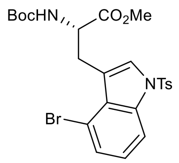

S23

methyl (*S*)-3-(4-bromo-1-tosyl-1H-indol-3-yl)-2-((tert-butoxycarbonyl)amino)propanoate

Following a modified procedure,[4] a 25-mL rbf was charged with a stir bar, methyl (*S*)-3-(4-bromo-1H-indol-3-yl)-2-((tert-butoxycarbonyl)amino)propanoate (0.6 mmol, 273 mg, 1.0 equiv), hydrogen tetra(but-1-yl)ammonium sulphate (0.6 mmol, 216 mg, 1.0 equiv), and NaOH (3.2 mmol, 127 mg, 5.0 equiv). This was fitted with a condenser, and anhydrous DCM (3 mL, 0.2 M) was added. This was warmed to reflux and stirred for 20 minutes. This was allowed to cool to room temperature, then p-tosyl-Cl (1.9 mmol, 364 mg, 3.0 equiv) was added. This was heated to reflux and stirred for an additional 3 hours. Upon consumption of starting material, the reaction mixture was diluted with EtOAc, then washed with saturated sodium bicarbonate. This was purified by silica gel column chromatography (25% EtOAc in hexanes) to yield the title substrate as a white powder (183 mg, 40% yield). The physical properties and ^{1}H NMR data were consistent with reported values.[21]

R_f = 0.35 in 25% EtOAc in hexanes

^{1}H NMR (400 MHz, $CDCl_3$) δ 7.94 (d, *J* = 8.4 Hz, 1H), 7.73 (d, *J* = 8.3 Hz, 2H), 7.48 (s, 1H), 7.38 (d, *J* = 7.8 Hz, 1H), 7.24 (d, *J* = 8.0 Hz, 2H), 7.12 (dd, *J* = 8.1, 8.1 Hz, 1H), 5.06 (d, *J* = 8.4 Hz, 1H), 4.69 (td, *J* = 8.7, 5.3 Hz, 1H), 3.70 (s, 3H), 3.60 (dd, *J* = 15.0, 5.2 Hz, 1H), 3.26 (dd, *J* = 14.9, 8.7 Hz, 1H), 2.35 (s, 3H), 1.38 (s, 7H).

^{13}C NMR (101 MHz, $CDCl_3$) δ 172.7, 155.2, 145.4, 136.4, 135.0, 130.1, 128.8, 128.1, 127.0, 126.4, 125.6, 118.0, 114.5, 113.0, 80.2, 54.1, 52.5, 29.3, 28.4, 21.7.

HRMS (+ APCI) calculated for $C_{24}H_{27}O_6N_2{}^{79}Br^{32}S$ [M+H] 550.0768, found 550.0764.

$[\alpha]_D^{22}$ = +22.3 (c = 0.63, MeOH).

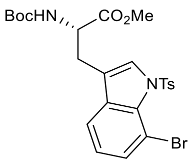

S24

methyl (*S*)-3-(7-bromo-1-tosyl-1H-indol-3-yl)-2-((tert-butoxycarbonyl)amino)propanoate

Following a modified procedure,[4] a 25-mL rbf was charged with a stir bar, methyl (*S*)-3-(4-bromo-1H-indol-3-yl)-2-((tert-butoxycarbonyl)amino)propanoate (0.6 mmol, 273 mg, 1.0 equiv), hydrogen tetra(but-1-yl)ammonium sulphate (0.6 mmol, 216 mg, 1.0 equiv), and NaOH (3.2 mmol, 127 mg, 5.0 equiv). This was fitted with a condenser, and anhydrous DCM (3 mL, 0.2 M) was added. This was warmed to reflux and stirred for 20 minutes. This was allowed to cool to room temperature, then p-tosyl-Cl (1.9 mmol, 364 mg, 3.0 equiv) was added. This was heated to reflux and stirred for an additional 3 hours. Upon consumption of starting material,

the reaction mixture was diluted with EtOAc, then washed with saturated sodium bicarbonate. This was purified by silica gel column chromatography (25% EtOAc in hexanes) to yield the title substrate as a white powder (70 mg, 20% yield).

R_f = 0.45 in 25% EtOAc in hexanes

^{1}H NMR (400 MHz, $CDCl_3$) δ 7.69 (s, 1H), 7.65 (d, *J* = 8.3 Hz, 2H), 7.49 – 7.42 (m, 2H), 7.26 (d, *J* = 8.2 Hz, 3H), 7.06 (dd, *J* = 7.8, 7.8 Hz, 1H), 5.13 (d, *J* = 7.8 Hz, 1H), 4.71 – 4.60 (m, 1H), 3.72 (s, 3H), 3.29 (dd, *J* = 14.6, 5.6 Hz, 1H), 3.17 (dd, *J* = 14.7, 5.7 Hz, 1H), 2.39 (s, 3H), 1.44 (s, 9H).

^{13}C NMR (101 MHz, $CDCl_3$) δ 172.1, 155.1, 144.8, 137.4, 135.1, 133.9, 131.1, 129.8, 129.0, 127.2, 124.5, 118.8, 115.6, 106.4, 80.3, 53.8, 52.6, 28.5, 27.9, 21.8.

HRMS (+ APCI) calculated for $C_{24}H_{27}O_6N_2{}^{79}Br^{32}S$ [M+H] 550.0768, found 550.0766.

$[\alpha]_D^{22}$ = +78.0 (c = 0.35, MeOH).

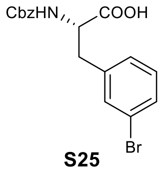

S25

(*S*)-2-(((benzyloxy)carbonyl)amino)-3-(3-bromophenyl)propanoic acid

A 100-mL round bottom flask equipped with stir bar was charged with (*S*)-2-Amino-3-(3-bromophenyl)propanoic acid (5.0 mmol, 1.22 g, 1.0 equiv) and sodium carbonate (12.5 mmol, 1.32 g, 2.5 equiv). To this was added THF and DI water (1:1, 25 mL, 0.2M). This was cooled to 0 °C in an ice bath for 10 minutes. Then, benzyl chloroformate (7.5 mmol, 1.1 mL, 1.5 equiv) was added dropwise. This was allowed to warm to room temperature, and was stirred for 16 hours. The reaction mixture was diluted with water, then extracted with ether. The aqueous phase was acidified with 1 N HCl to pH = 1-2. This was extracted with EtOAc thrice and washed with brine. This was dried over sodium sulfate and concentrated *in vacuo* to provide the title compound as a white powder (1.77 g, 94% yield). This was used without further purification.

^{1}H NMR (400 MHz, DMSO) δ 12.81 (s, 1H), 7.69 (d, *J* = 8.5 Hz, 1H), 7.48 (s, 1H), 7.42 (dt, *J* = 7.2, 1.9 Hz, 1H), 7.37 – 7.16 (m, 7H), 4.97 (s, 2H), 4.18 (ddd, *J* = 10.8, 8.5, 4.4 Hz, 1H), 3.08 (dd, *J* = 13.8, 4.4 Hz, 1H), 2.82 (dd, *J* = 13.8, 10.8 Hz, 1H).

^{13}C NMR (101 MHz, DMSO) δ 173.0, 156.0, 140.8, 137.0, 131.9, 130.3, 129.3, 128.3, 128.2, 127.7, 127.4, 121.4, 65.3, 55.2, 35.9.

HRMS (+ ESI) calculated for $C_{17}H_{16}O_4N^{79}Br^{23}Na$ [M+Na] 400.0155, found 400.0158.

$[\alpha]_D^{22}$ = +2.4 (c = 0.093, MeOH).

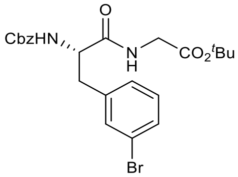

S26

tert-butyl (*S*)-(2-(((benzyloxy)carbonyl)amino)-3-(3-bromophenyl)propanoyl)glycinate

Prepared according to GP3 using **S25** (5.0 mmol, 1.89 g, 1.0 equiv), glycine *tert*-butyl ester hydrochloride (5.0 mmol, 640 mg, 1.0 equiv), HOBt (6.0 mmol, 919 mg, 1.2 equiv), EDC (6.5 mmol, 1.37 g, 1.3 equiv), DIPEA (19 mmol, 3.3 mL, 3.8 equiv), and DCM (23 mL, 0.25 M). After 15 hours, the reaction was worked up and purified by silica gel column chromatography (25% EtOAc in hexanes) to afford the title compound as a white solid (527 mg, 21% yield). **Note: upon workup, large emulsion formed.

R_f = 0.25 in 25% EtOAc in hexanes

^{1}H NMR (400 MHz, $CDCl_3$) δ 7.41 – 7.28 (m, 7H), 7.19 – 7.11 (m, 2H), 6.27 (s, 1H), 5.28 (d, *J* = 7.6 Hz, 1H), 5.09 (dd, *J* = 14.9, 12.1 Hz, 2H), 4.48 – 4.39 (m, 1H), 3.91 (dd, *J* = 18.3, 5.1 Hz, 1H), 3.84 (dd, *J* = 18.3, 5.0 Hz, 1H), 3.14 – 2.99 (m, 2H), 1.46 (s, 9H).

^{13}C NMR (101 MHz, $CDCl_3$) δ 170.6, 168.5, 156.0, 138.8, 136.2, 132.5, 130.4, 130.4, 128.7, 128.4, 128.2, 128.1, 122.8, 82.7, 67.4, 56.1, 42.1, 38.2, 28.2.

HRMS (+ ESI) calculated for $C_{23}H_{27}O_5N_2{}^{79}Br^{23}Na$ [M+Na] 513.0996, found 513.0999.

$[\alpha]_D^{22}$ = -0.5 (c = 1.03, $CHCl_3$).

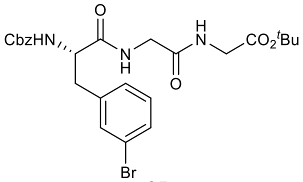

25

tert-butyl (*S*)-(*S*)-(2-(((benzyloxy)carbonyl)amino)-3-(3-bromophenyl)propanoyl)glycylglycinate

To a 100-mL round bottom flask was added **S26** (0.92 mmol, 450 mg, 1.0 equiv) and stir bar. To this was added a mixture of DCM and TFA (1:1, 7.2 mL, 0.1 M). This was stirred for 2 hours, until starting material consumption was observed by TLC. Then, the reaction mixture was concentrated to provide (*S*)-(*S*)-(2-(((benzyloxy)carbonyl)amino)-3-(3-bromophenyl)propanoyl)glycylglycine, which used immediately in the next step without further purification.

Prepared according to GP3 using the crude material from the previous step (0.92 mmol, 1.0 equiv), glycine *tert*-butyl ester hydrochloride (0.92 mmol, 154 mg, 1.0 equiv), HOBt (1.1 mmol, 169 mg, 1.2 equiv), EDC (1.6 mmol, 288 mg, 1.3 equiv), DIPEA (3.5 mmol, 0.6 mL, 3.8 equiv), and DCM (4.2 mL, 0.25 M). After 15 hours, the reaction was worked up and

purified by silica gel column chromatography (50% EtOAc in hexanes) to afford the title compound as a white solid (117 mg, 23% yield). **Note: upon workup, large emulsion formed.

R$_f$ = 0.25 in 50% EtOAc in hexanes

^{1}H NMR (400 MHz, CDCl$_3$) δ 7.40 – 7.28 (m, 7H), 7.19 – 7.06 (m, 2H), 6.62 – 6.55 (m, 1H), 6.55 – 6.47 (m, 1H), 5.32 (d, *J* = 7.5 Hz, 1H), 5.13 – 5.02 (m, 2H), 4.40 (dt, *J* = 7.1, 7.1 Hz, 1H), 4.03 – 3.83 (m, 4H), 3.11 (dd, *J* = 13.9, 6.6 Hz, 1H), 3.02 (dd, *J* = 13.9, 7.3 Hz, 1H), 1.46 (d, *J* = 0.8 Hz, 9H).

^{13}C NMR (151 MHz, CDCl$_3$) δ 171.1, 168.8, 168.4, 156.3, 138.7, 136.0, 132.4, 130.5, 130.5, 128.8, 128.5, 128.3, 128.0, 122.9, 82.7, 67.5, 56.4, 43.0, 42.1, 37.9, 28.2.

HRMS (+ ESI) calculated for $C_{25}H_{30}O_6N_3{}^{79}Br^{23}Na$ [M+Na] 570.1210, found 570.1215.

$[\alpha]_D^{22}$ = +1.8 (c = 0.32, CHCl$_3$).

2.3 Preparation of Brominated Amino Acids

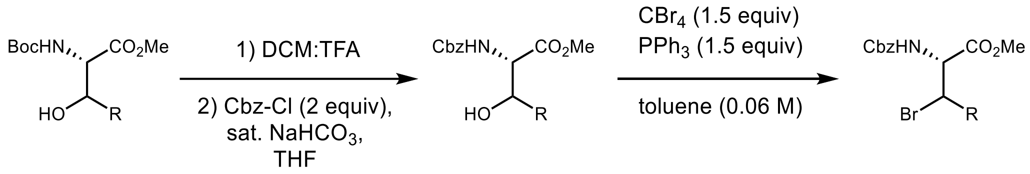

General Procedure 1: Boc deprotection/Cbz protection

Following a reported modified procedure,[22] the Boc-protected amino acid derivative (1.0 equiv) was taken up in DCM. TFA was added slowly while stirring (DCM:TFA 1:1, 0.1 M). This was stirred for 1-2 hours, or until consumption of starting material was observed, then concentrated to yield the crude amino acid derivative, which was used immediately in the next reaction.

Following a reported modified procedure,[23] the crude amino acid derivative was taken up in THF and sat. NaHCO$_3$ (1:1, 0.05 M). This mixture was cooled to 0 °C in an ice bath. Then, a solution of Cbz-Cl in THF (0.06 M) was added slowly to the reaction mixture (bringing the concentration of the reaction to 0.014 M). This was stirred at 0 °C for 1-3 hours, or until starting material consumption was observed. The reaction mixture was diluted with DCM, then quenched at 0 °C with 3M HCl. The layers were separated, and the organic layer was washed with brine, then dried over sodium sulfate and concentrated *in vacuo* to provide a crude mixture. This was purified by silica gel column chromatography to afford the corresponding Cbz-protected amino acid derivative.

General Procedure 2: Bromination

Following a modified procedure,[1] to an oven-dried round bottom flask equipped with a stir bar was added PPh$_3$ (1.5 equiv). To this was added anhydrous toluene (0.1 M) and CBr$_4$ (1.5 equiv). This was stirred for 1 hour at room temperature. To this was added the amino acid derivative (1.0 equiv). This was stirred for 12 hours or until starting material consumption was observed by TLC. The reaction mixture was filtered through celite, rinsing with toluene or EtOAc, and concentrated to yield a crude oil, which was purified by silica gel column chromatography unless otherwise stated.

Note: Beginning with Boc-protected enzymatically synthesized amino acids instead of directly introducing the Cbz group consistently affords better product isolation following enzymatic hydroxylation. For example, in the case of 3-OH-Lys (**S16**), attempts to Cbz-protect the crude hydroxylation product resulted in failure to isolate the protected product. This strategy improves both yield and reliability across multiple substrates.

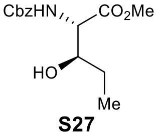

S27

methyl (2*S*,3*R*)-2-(((benzyloxy)carbonyl)amino)-3-hydroxypentanoate

Prepared according to GP1 with the following alterations: A 1-dram vial containing **S12** (0.49 mmol, 121.9 mg, 1.0 equiv) was charged with cooled 4M HCl in dioxane (2.5 mL, 0.2 M). This was stirred at 0 °C for 4.5 hours, then used immediately following GP1, using Cbz-Cl (0.99 mmol, 141 µL, 2.0 equiv), THF:sat. $NaHCO_3$ (1:1, 12.2 mL, 0.04 M), and THF for Cbz-Cl solution (18.5 mL, 0.014 M). After 1 hour and 40 mintues, the reaction was worked up (extracting with EtOAc), and purified by silica gel column chromatography (40% EtOAc in hexanes) to afford the title compound as a colorless oil (113 mg, 86% yield over two steps).

R_f = 0.35 (40% EtOAc in hexanes)

"#" indicates rotamer

^{1}H NMR (800 MHz, $CDCl_3$) δ 7.42 – 7.27 (m, 4.61H), 5.63 (d, *J* = 9.3 Hz, 0.86H), 5.48# (br s, 0.15H), 5.12 (s, 1.95H), 4.41 (dd, *J* = 9.4, 2.2 Hz, 0.86H), 4.27# (br s, 0.13H), 4.06 – 4.00 (m, 0.86H), 3.96# (s, 0.15H), 3.76 (s, 2.57H), 3.68# (br s, 0.37H), 2.27# (br s, 0.14H), 2.21 (d, *J* = 5.3 Hz, 0.85H), 1.60 – 1.49 (m, 2.00H), 0.97 (t, *J* = 7.5 Hz, 2.65H), 0.92# (br s, 0.37H).

^{13}C NMR (201 MHz, $CDCl_3$) δ 172.1, 156.8, 136.3, 128.6, 128.3, 128.2, 73.6, 67.3, 57.6, 52.7, 26.9, 10.2, 0.1.

HRMS (+ p APCI) calculated for $C_{14}H_{20}O_5N$ [M+H] 282.1336, found 282.1332.

$[\alpha]_D^{23}$ = +0.38 (c = 0.80, MeOH).

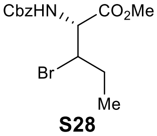

S28

methyl (2*R*,3*S*)-2-(((benzyloxy)carbonyl)amino)-3-bromopentanoate

Prepared according to GP2 using **S27** (1.67 mmol, 470 mg, 1.0 equiv), CBr_4 (2.5 mmol, 829 mg, 1.5 equiv), PPh_3 (2.5 mmol, 829 mg, 1.5 equiv), toluene (17 mL, 0.1 M). After 24 hours, the reaction was worked up and purified by column chromatography (15% EtOAc in hexanes), to afford the title compound as a colorless oil in a 1:1.4 mixture of diastereomers (271 mg, 47% yield).

R_f = 0.22 (10% EtOAc in hexanes)

"^" indicates major diastereomer

"*" indicates minor diastereomer

^{1}H NMR (400 MHz, $CDCl_3$) δ 7.42 – 7.29^* (m, 5H), 5.68* (d, J = 8.6 Hz, 0.41H), 5.51^ (d, J = 9.7 Hz, 0.56H), 5.18 – 5.07^* (m, 2H), 4.78^ (dd, J = 9.7, 2.3 Hz, 0.56H), 4.69* (dd, J = 8.6, 3.5 Hz, 0.42H), 4.44^ (ddd, J = 8.5, 6.1, 2.3 Hz, 0.50H), 4.11* (ddd, J = 8.9, 5.0, 3.5 Hz, 0.42H), 3.79^* (s, 3H), 2.08 – 1.82^* (m, 2H), 1.14 – 1.02^* (m, 3H).

^{13}C NMR (101 MHz, $CDCl_3$) δ 170.1, 169.2, 156.5, 155.6, 136.1, 128.7, 128.5, 128.3, 128.3, 67.6, 67.5, 58.7, 58.5, 58.4, 58.0, 53.2, 52.9, 29.6, 29.3, 12.8, 12.6.

HRMS (+ ESI) calculated for $C_{14}H_{19}O_4N^{79}Br$ [M+H] 344.0492, found 344.0493.

$[\alpha]_D^{21}$ = +0.018 (c = 1.120, MeOH).

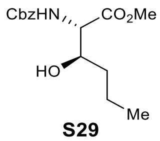

S29

methyl (2*S*,3*R*)-2-(((benzyloxy)carbonyl)amino)-3-hydroxyhexanoate

Prepared according to GP1 using **S14** (0.37 mmol, 96.3 mg, 1.0 equiv) and DCM:TFA (1:1, 3 mL, 0.1 M). After 2 hours, the reaction was concentrated and used immediately in the next step, with Cbz-Cl (0.74 mmol, 105 µL, 2.0 equiv), and THF:sat. $NaHCO_3$ (1:1, 7.4 mL, 0.04 M), and THF for Cbz-Cl solution (18.5 mL, 0.014 M). After 1 hour and 40 mintues, the reaction was worked up (extracting with EtOAc), and purified by silica gel column chromatography (25% EtOAc in hexanes) to afford the title compound as a colorless oil (86 mg, 78% yield over two steps).

R_f = 0.2 (25% EtOAc in hexanes)

^{1}H NMR (400 MHz, $CDCl_3$) δ 7.39 – 7.31 (m, 5H), 5.58 (d, J = 9.3 Hz, 1H), 5.17 – 5.07 (m, 2H), 4.39 (dd, J = 9.3, 2.1 Hz, 1H), 4.18 – 4.10 (m, 1H), 3.77 (s, 3H), 2.02 (s, 1H), 1.54 – 1.36 (m, 4H), 0.93 (t, J = 6.9 Hz, 3H).

^{13}C NMR (101 MHz, $CDCl_3$) δ 172.1, 156.8, 136.3, 128.7, 128.3, 128.2, 71.8, 67.3, 58.0, 52.8, 35.9, 18.9, 14.0.

HRMS (+ APCI) calculated for $C_{15}H_{22}O_5N$ [M+H] 296.1493, found 296.1490.

$[\alpha]_D^{23}$ = -3.7 (c = 0.32, MeOH).

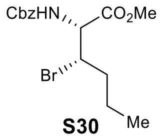

S30

methyl (2*R*,3*S*)-2-(((benzyloxy)carbonyl)amino)-3-bromohexanoate

Prepared according to GP2 using **S29** (0.22 mmol, 65.1 mg, 1.0 equiv), CBr_4 (0.33 mmol, 87.8 mg, 1.5 equiv), PPh_3 (0.33 mmol, 112 mg, 1.5 equiv), toluene (2.2 mL, 0.1 M). After 24 hours, the reaction was worked up and purified by column chromatography (10% EtOAc and hexanes), to afford the title compound as a colorless oil (35.6 mg, 46% yield, one diastereomer observed).

$\mathbf{R_f}$ = 0.2 (10% EtOAc in hexanes)

^{1}H NMR (400 MHz, $CDCl_3$) δ 7.41 – 7.29 (m, 5H), 5.69 (d, *J* = 8.6 Hz, 1H), 5.18 – 5.07 (m, 2H), 4.68 (dd, *J* = 8.7, 3.4 Hz, 1H), 4.20 (ddd, *J* = 9.0, 5.2, 3.3 Hz, 1H), 3.80 (s, 3H), 1.93 (dtd, *J* = 14.1, 8.9, 5.4 Hz, 2H), 1.70 – 1.56 (m, 1H), 1.45 (ddt, *J* = 13.4, 9.0, 6.8 Hz, 1H), 0.95 (t, *J* = 7.4 Hz, 3H).

^{13}C NMR (101 MHz, $CDCl_3$) δ 169.2, 155.6, 136.1, 128.7, 128.5, 128.3, 67.5, 58.9, 56.3, 52.9, 37.8, 21.2, 13.4.

HRMS (+ ESI) calculated for $C_{15}H_{21}O_4N^{79}Br$ [M+H] 358.0649, found 358.0652.

$[\alpha]_D^{21}$ = -18.3 (c = 1.108, MeOH).

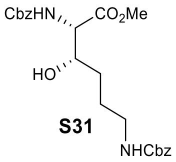

S31

methyl (2*S*,3*S*)-2,6-bis(((benzyloxy)carbonyl)amino)-3-hydroxyhexanoate

Prepared according to GP1 using **S16** (0.455 mmol, 171.3 mg, 1.0 equiv) and DCM:TFA (1:1, 3.6 mL, 0.1 M). After 1 hour, the reaction was concentrated and used immediately in the next step, with Cbz-Cl (1.365 mmol, 0.2 mL, 2.0 equiv), and THF:sat. $NaHCO_3$ (1:1, 9.2 mL, 0.04 M), and THF for Cbz-Cl solution (23 mL, 0.014 M). After 1 hour and 30 mintues, the reaction was worked up and purified by silica gel column chromatography (25% EtOAc in hexanes) to afford the title compound as a colorless oil (182.9 mg, 90% yield over two steps).

$\mathbf{R_f}$ = 0.4 (50% EtOAc in hexanes)

^{1}H NMR (400 MHz, $CDCl_3$) δ 7.39 – 7.28 (m, 10H), 5.71 (d, *J* = 7.6 Hz, 1H), 5.16 – 5.01 (m, 4H), 4.91 – 4.80 (m, 1H), 4.42 (dd, *J* = 7.7, 3.9 Hz, 1H), 3.99 – 3.88 (m, 1H), 3.76 (s, 3H), 3.31 – 3.13 (m, 2H), 1.80 – 1.56 (m, 2H), 1.56 – 1.42 (m, 2H).

^{13}C NMR (101 MHz, $CDCl_3$) δ 170.8, 156.8, 156.6, 136.7, 136.1, 128.7, 128.7, 128.5, 128.3, 128.3, 72.8, 67.6, 66.9, 58.9, 52.8, 40.7, 30.1, 26.6.

HRMS (+ APCI) calculated for $C_{23}H_{29}O_7N_2$ [M+H] 445.1969, found 445.1973.

$[\alpha]_D^{23}$ = +11.2 (c = 0.69, MeOH).

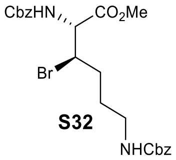

S32

methyl (2*R*,3*R*)-2,6-bis(((benzyloxy)carbonyl)amino)-3-bromohexanoate

Prepared according to GP2 using **S31** (0.37 mmol, 165 mg, 1.0 equiv), CBr_4 (0.56 mmol, 147 mg, 1.5 equiv), PPh_3 (0.56 mmol, 195 mg, 1.5 equiv), toluene (3.7 mL, 0.1 M). After 24 hours, the reaction was worked up and purified by column chromatography (25% EtOAc and hexanes), to afford the title compound as a colorless oil (92.5 mg, 49% yield, one diastereomer observed).

R_f = 0.65 (50% EtOAc in hexanes)

^{1}H NMR (400 MHz, $CDCl_3$) δ 7.42 – 7.27 (m, 10H), 5.57 (d, *J* = 9.6 Hz, 1H), 5.19 – 5.04 (m, 4H), 4.99 – 4.88 (m, 1H), 4.76 (dd, *J* = 9.6, 2.3 Hz, 1H), 4.51 (d, *J* = 6.4 Hz, 1H), 3.78 (s, 3H), 3.21 (q, *J* = 6.5 Hz, 2H), 1.97 – 1.81 (m, 2H), 1.81 – 1.58 (m, 2H).

^{13}C NMR (101 MHz, $CDCl_3$) δ 169.8, 156.6, 156.5, 136.6, 136.0, 128.7, 128.7, 128.5, 128.3, 77.5, 77.2, 76.8, 67.7, 66.9, 58.0, 55.7, 53.3, 40.3, 33.2, 1.2.

HRMS (+ ESI) calculated for $C_{23}H_{28}O_6N_2{}^{79}Br$ [M+H] 507.1125, found 507.1107.

$[\alpha]_D^{23}$ = +20.5 (c = 1.02, MeOH).

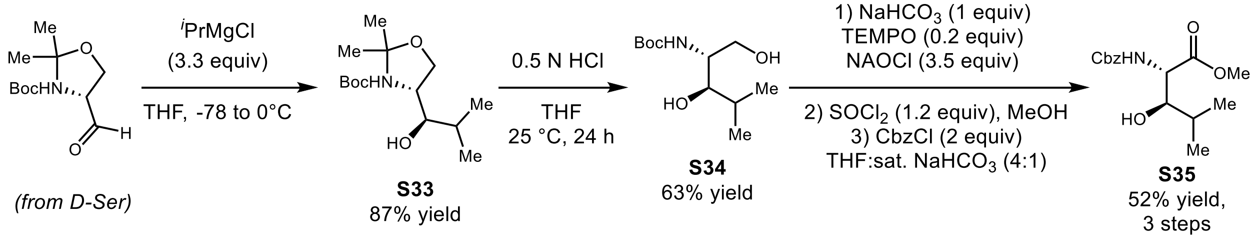

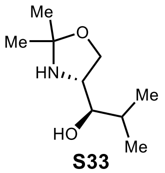

S33

(*R*)-1-((*R*)-2,2-dimethyloxazolidin-4-yl)-2-methylpropan-1-ol

To a flame-dried round bottom flask with stir bar under an inert atmosphere was added THF (66 mL, 0.15 M), then Garner's aldehyde (1 equiv, 10 mmol, 2.29 g). This was cooled to -78 °C in a dry ice/acetone bath. To this was added a 2.0 M solution of isopropylmagnesium chloride in THF (3.3 equiv, 33 mmol, 16.5 mL) over the course of 30 minutes. This was stirred for two hours at -78 °C, then warmed to 0 °C in an ice bath. The reaction mixture was diluted with ether (60 mL), then quenched with a saturated ammonium chloride solution. EtOAc was added, and the organic layer was washed with brine, dried over sodium sulfate, filtered, and concentrated *in vacuo*. This was purified by column chromatography (5% EtOAc in Hex) to

provide the product as a clear yellow viscous oil (1.50 g, 87% yield). These properties and spectra matched literature precedent.[24]

^{1}H NMR (400 MHz, CDCl$_3$) δ 4.05 (s, 2H), 3.93 (dd, *J* = 9.3, 5.7 Hz, 1H), 3.76 (d, *J* = 9.3 Hz, 1H), 3.51 (s, 1H), 1.71 – 1.64 (m, 1H), 1.60 (dd, *J* = 2.1, 1.2 Hz, 6H), 1.49 (s, 9H), 1.02 (d, *J* = 6.8 Hz, 3H), 0.90 (d, *J* = 6.7 Hz, 3H).

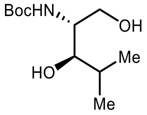

S34

tert-butyl ((2*R*,3*R*)-1,3-dihydroxy-4-methylpentan-2-yl)carbamate

This product was synthesized in 63% yield following a reported literature procedure.[24]

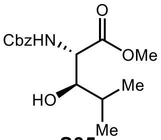

S35

methyl (2*S*,3*R*)-2-(((benzyloxy)carbonyl)amino)-3-hydroxy-4-methylpentanoate

To a round bottom flask was added **S34** (1 equiv, 3.4 mmol, 803 mg) and acetone (17 mL, 0.2 M). To this was added TEMPO (0.2 equiv, 0.68 mmol, 107 mg) followed by a 5% aqueous solution of NaHCO3 (17 mL). This mixture was cooled to 0 °C and sodium hypochlorite (3.5 equiv, 12 mmol, 7.43 mL, 10% by weight) was added in 3 portions over 30 minutes. This mixture was then warmed to room temperature and stirred for 3 hours, when TLC showed complete consumption of the starting material. The reaction mixture was diluted with water, washed with ether twice, then acidified to a pH of 2 with 1 M HCl. The aqueous phase was extracted with ethyl acetate three times, then the combined organic layers were dried over sodium sulfate, filtered, and concentrated *in vacuo*.

This crude was dried under high vacuum in a round bottom flask, then placed under inert atmosphere with stir bar. To this residue was added MeOH (3 mL). Then, thionyl chloride was added (1.2 equiv, 3.7 mmol, 0.27 mL), and the reaction mixture was heated to 65 °C for 3.5 hours. At this point, the reaction mixture was concentrated *in vacuo*.

This crude residue was immediately taken up in THF:sat. $NaHCO_3$ (1:1, 75 mL, 0.04 M). This mixture was cooled to 0 °C, then to this was added a solution of benzyl chloroformate (2 equiv, 6 mmol, 0.86 mL) in THF (125 mL, 0.05 M). This mixture was stirred at 0 °C for 1 hour, then the reaction was quenched with 3 N HCl. This mixture was extracted with EtOAc three times, then dried over sodium sulfate and concentrated. The product was purified by column chromatography (25% EtOAc in Hex) as a clear and colorless oil (525.5 mg, 59% yield).
R_f = 0.2 (25% EtOAc in hexanes)

^{1}H NMR (400 MHz, CDCl$_3$) δ 7.40 – 7.28 (m, 5H), 5.52 (s, 1H), 5.13 (s, 2H), 4.56 (dd, *J* = 9.5, 2.0 Hz, 1H), 3.77 (s, 3H), 3.75 – 3.64 (m, 1H), 2.01 (s, 1H), 1.82 – 1.69 (m, 1H), 1.01 (d, *J* = 6.8 Hz, 3H), 0.96 (d, *J* = 6.7 Hz, 3H).

^{13}C NMR (101 MHz, CDCl$_3$) δ 170.5, 169.8, 156.4, 155.5, 136.1, 136.1, 128.7, 128.5, 128.4, 128.3, 128.2, 77.5, 77.2, 76.8, 67.5, 67.5, 64.4, 63.9, 56.9, 53.1, 52.7, 33.2, 31.9, 22.1, 21.1, 20.9, 20.3.

HRMS (+ APCI) calculated for $C_{15}H_{22}O_5N$ [M+H] 296.1493, found 296.1489.

$[\alpha]_D^{23}$ = -9.9 (c = 0.83, MeOH).

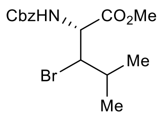

S36

methyl (2*R*,3*S*)-2-(((benzyloxy)carbonyl)amino)-3-bromo-4-methylpentanoate

Prepared according to GP2 using **S35** (1.60 mmol, 471 mg, 1.0 equiv), CBr_4 (2.4 mmol, 799 mg, 1.5 equiv), PPh_3 (2.4 mmol, 631 mg, 1.5 equiv), toluene (16 mL, 0.1 M). After 17 hours, the reaction was worked up and purified by column chromatography (10% EtOAc and hexanes), to afford the title compound as a colorless oil in a 7:1 mixture of diastereomers (91.2 mg, 19% yield).

R_f = 0.25 (10% EtOAc in hexanes)

"^" indicates major diastereomer

"*" indicates minor diastereomer

^{1}H NMR (400 MHz, $CDCl_3$) δ 7.42 – 7.28^* (m, 5H), 5.62^ (d, *J* = 8.9 Hz, 0.70H), 5.47* (d, *J* = 10.1 Hz, 0.10H), 5.19 – 5.06^* (m, 2H), 4.89* (dd, *J* = 10.1, 2.4 Hz, 0.09H), 4.84^ (dd, *J* = 8.9, 5.0 Hz, 0.76H), 4.27* (dd, *J* = 9.3, 2.5 Hz, 0.11H), 3.92^ (dd, *J* = 7.8, 5.0 Hz, 0.82H), 3.78^* (s, 3H), 2.18^ (dp, *J* = 13.4, 6.7 Hz, 0.83H), 2.03* (dp, *J* = 9.3, 6.6 Hz, 0.09H), 1.20 – 1.00^* (m, 6H).

^{13}C NMR (101 MHz, $CDCl_3$) δ 170.5, 169.8, 156.4, 155.5, 136.1, 136.1, 128.7, 128.5, 128.4, 128.3, 128.2, 67.5, 67.5, 64.4, 63.9, 56.9, 53.1, 52.7, 33.2, 31.9, 22.1, 21.1, 20.9, 20.3.

HRMS (+ ESI) calculated for $C_{15}H_{21}O_4N^{79}Br$ [M+H] 358.0649, found 358.0649.

$[\alpha]_D^{23}$ = +9.5 (c = 0.90, MeOH).

2.4 Preparation of Dipeptides

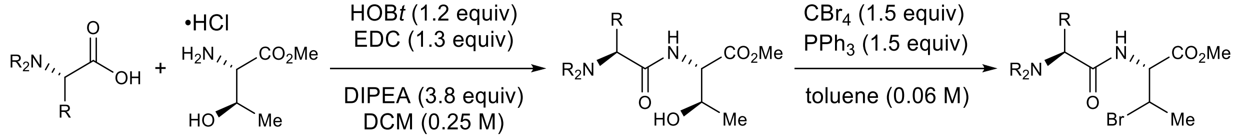

General Procedure 3: Amidation

Following a modified procedure,[25] an oven-dried round bottom flask with stir bar was charged with the N-unprotected amino acid and evacuated and backfilled 3x. To this was added anhydrous DCM (0.25 M). To this stirred suspension was added distilled DIPEA (3.8 equiv) at room temperature. Upon dissolution, the solution was cooled to 0 °C in an ice bath, and the O-unprotected amino acid (1 equiv) and HOBt (1.2 equiv) were added in succession. This was stirred at 0 °C for 15 minutes, then EDC (1.3 equiv) was added. This mixture was warmed to room temperature and stirred for 12 h or until reaction completion. Upon observed completion by TLC, the mixture was diluted with DCM and washed with 0.1 N HCl, then saturated sodium bicarbonate. The organic layer was dried over sodium sulfate, filtered, and concentrated *in vacuo* to provide a crude oil. This was purified by silica gel column chromatography to afford the corresponding peptide.

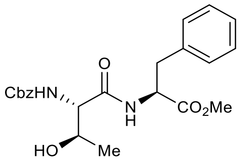

S37

methyl ((benzyloxy)carbonyl)-*L*-threonyl-*L*-phenylalaninate

Prepared according to GP3 using ((benzyloxy)carbonyl)-*L*-threonine (20 mmol, 4.4 g, 1.0 equiv), methyl *L*-phenylalaninate hydrochloride (20 mmol, 4.3 g, 1.0 equiv), HOBt (24 mmol, 4.6 g, 1.2 equiv), EDC (26 mmol, 5.0 g, 1.3 equiv), DIPEA (76 mmol, 13.2 mL, 3.8 equiv), and DCM (80 mL, 0.25 M). After 24 hours, the reaction was worked up and purified by silica gel column chromatography (50% EtOAc in hexanes) to afford the title compound as a white solid (5.2 g, 68% yield). The physical properties and ^{1}H NMR data were consistent with reported values.[26]

$\mathbf{R_f}$ = 0.4 (50% EtOAc in hexanes)

^{1}H NMR (400 MHz, CDCl$_3$) δ 7.40 – 7.31 (m, 5H), 7.30 – 7.27 (m, 1H), 7.25 – 7.20 (m, 2H), 7.12 – 7.06 (m, 2H), 6.89 (d, *J* = 8.2 Hz, 1H), 5.61 (d, *J* = 8.0 Hz, 1H), 5.10 (dd, *J* = 3.2 Hz, 2H), 4.85 (td, *J* = 7.5, 5.3 Hz, 1H), 4.35 – 4.27 (m, 1H), 4.10 (dd, *J* = 8.0, 2.3 Hz, 1H), 3.75 (s, 3H), 3.17 (dd, *J* = 14.0, 5.4 Hz, 1H), 3.00 (dd, *J* = 13.9, 7.2 Hz, 1H), 2.83 (s, 1H), 1.14 (d, *J* = 6.5 Hz, 3H).

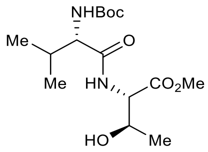

S38

methyl (tert-butoxycarbonyl)-*L*-valyl-*L*-threoninate

Prepared according to GP3 using (tert-butoxycarbonyl)-*L*-valine (20.0 mmol, 4.35 g, 1.0 equiv), methyl (2*S*)-2-amino-3-hydroxybutanoate hydrochloride (20.0 mmol, 3.39 g, 1.0 equiv), HOBt (24 mmol, 4.59 g, 1.2 equiv), EDC (26 mmol, 4.98 g, 1.3 equiv), DIPEA (76 mmol, 13.2 mL, 3.8 equiv), and DCM (80 mL, 0.25 M). After 24 hours, the reaction was worked up and purified by silica gel column chromatography (50% EtOAc in hexanes) to afford the title compound as a white foam (3.86 g, 50% yield). The physical properties and ^{1}H NMR data were consistent with reported values.[27]

$\mathbf{R_f}$ = 0.25 in 50% EtOAc in hexanes

^{1}H NMR (800 MHz, CDCl$_3$) δ 6.72 (d, *J* = 9.5 Hz, 1H), 5.12 (d, *J* = 7.8 Hz, 1H), 4.62 (dd, *J* = 8.9, 2.6 Hz, 1H), 4.39 – 4.33 (m, 1H), 3.94 (dd, *J* = 8.8, 6.8 Hz, 1H), 3.77 (s, 3H), 2.73 (s, 1H), 2.15 – 2.07 (m, 1H), 1.43 (s, 9H), 1.21 (d, *J* = 6.5 Hz, 3H), 0.99 (d, *J* = 6.8 Hz, 3H), 0.96 (d, *J* = 6.8 Hz, 3H).

^{13}C NMR (201 MHz, CDCl$_3$) δ 172.4, 171.4, 156.2, 80.2, 68.3, 60.4, 57.3, 52.7, 30.9, 28.4, 20.1, 19.3, 18.2.

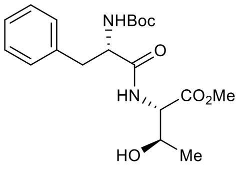

S39

methyl (tert-butoxycarbonyl)-*L*-phenylalanyl-*L*-threoninate

Prepared according to GP3 using (tert-butoxycarbonyl)-*L*-phenylalanine (24.0 mmol, 6.37 g, 1.0 equiv), methyl (2*S*)-2-amino-3-hydroxybutanoate hydrochloride (24.0 mmol, 4.10 g, 1.0 equiv), HOBt (28.8 mmol, 5.513 g, 1.2 equiv), EDC (31.2 mmol, 5.98 g, 1.3 equiv), DIPEA (90 mmol, 15.8 mL, 3.78 equiv), and DCM (96 mL, 0.25 M). After 24 hours, the reaction was worked up and purified by silica gel column chromatography (50% EtOAc in hexanes) to afford the title compound as a white foam (3.23 g, 35% yield). The physical properties and ^{1}H NMR data were consistent with reported values.[28]

R_f = 0.3 in 50% EtOAc in hexanes

^{1}H NMR (400 MHz, $CDCl_3$) δ 7.33 – 7.27 (m, 2H), 7.25 – 7.19 (m, 3H), 6.62 (d, *J* = 8.8 Hz, 1H), 5.00 (d, *J* = 7.8 Hz, 1H), 4.56 (dd, *J* = 8.9, 2.8 Hz, 1H), 4.41 – 4.33 (m, 1H), 4.31 – 4.22 (m, 1H), 3.73 (s, 3H), 3.13 (dd, *J* = 13.9, 6.6 Hz, 1H), 3.06 (dd, *J* = 13.9, 7.2 Hz, 1H), 1.56 (s, 1H), 1.41 (s, 9H), 1.16 (d, *J* = 6.4 Hz, 3H).

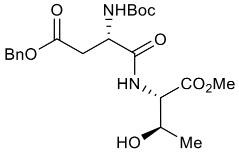

S40

benzyl (*S*)-3-((tert-butoxycarbonyl)amino)-4-(((2*S*,3*R*)-3-hydroxy-1-methoxy-1-oxobutan-2-yl)amino)-4-oxobutanoate

Prepared according to GP3 using (*S*)-4-(benzyloxy)-2-((tert-butoxycarbonyl)amino)-4-oxobutanoic acid (10 mmol, 1.57 g, 1.0 equiv), methyl (2*S*)-2-amino-3-hydroxybutanoate hydrochloride (10 mmol, 3.26 g, 1.0 equiv), HOBt (12 mmol, 1.85 g, 1.2 equiv), EDC (13 mmol, 2.51 g, 1.3 equiv), DIPEA (38 mmol, 6.6 mL, 3.8 equiv), and DCM (40 mL, 0.25 M). After 20 hours, the reaction was worked up and purified by silica gel column chromatography (50% EtOAc in hexanes) to afford the title compound as a white solid (3.0 g, 68% yield). The physical properties and ^{1}H NMR data were consistent with reported values.[29]

R_f = 0.3 in 50% EtOAc in hexanes.

^{1}H NMR (400 MHz, $CDCl_3$) δ 7.40 – 7.28 (m, 5H), 7.21 (d, *J* = 9.0 Hz, 1H), 5.71 (d, *J* = 8.8 Hz, 1H), 5.19 – 5.08 (m, 2H), 4.63 – 4.52 (m, 2H), 4.36 – 4.29 (m, 1H), 3.74 (s, 3H), 3.08 (dd, *J* = 17.2, 4.8 Hz, 1H), 2.77 (dd, *J* = 17.2, 5.8 Hz, 1H), 2.44 (s, 1H), 1.45 (s, 10H), 1.20 (d, *J* = 6.3 Hz, 3H).

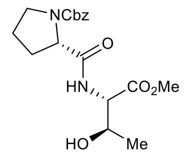

S41

benzyl (*S*)-2-(((2*S*,3*R*)-3-hydroxy-1-methoxy-1-oxobutan-2-yl)carbamoyl)pyrrolidine-1-carboxylate

Prepared according to GP3 using ((benzyloxy)carbonyl)-*L*-proline (10 mmol, 1.60 g, 1.0 equiv), methyl (2*S*)-2-amino-3-hydroxybutanoate hydrochloride (10 mmol, 2.49 g, 1.0 equiv), HOBt (12 mmol, 1.86 g, 1.2 equiv), EDC (13 mmol, 2.63 g, 1.3 equiv), DIPEA (38 mmol, 6.6 mL, 3.8 equiv), and DCM (40 mL, 0.25 M). After 20 hours, the reaction was worked up and purified by silica gel column chromatography (50% EtOAc in hexanes to 100% EtOAc) to afford the title compound as a white solid (3.30 g, 91% yield). The physical properties and ^{1}H NMR data were consistent with reported values.[30,31]

R_f = 0.4 in EtOAc

^{1}H NMR (400 MHz, DMSO) δ 8.04 – 7.94 (m, 1H), 7.41 – 7.21 (m, 5H), 5.12 – 4.93 (m, 3H), 4.50 – 4.37 (m, 1H), 4.28 (dt, *J* = 7.1, 3.3 Hz, 1H), 4.11 (dd, *J* = 6.3, 3.1 Hz, 1H), 3.62 (d, *J* = 6.0 Hz, 3H), 3.49 – 3.34 (m, 2H), 2.24 – 2.03 (m, 1H), 1.93 – 1.72 (m, 3H), 1.08 (d, *J* = 6.4 Hz, 1.34H (rotamer)), 0.95 (d, *J* = 6.3 Hz, 1.75H (rotamer)).

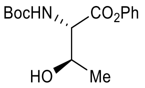

S42

phenyl (tert-butoxycarbonyl)-*L*-threoninate

A 100-mL flame-dried flask with stir bar was charged with (2S)-2-((tert-butoxycarbonyl)amino)-3-hydroxybutanoic acid (16 mmol, 3.5 g, 1.0 equiv), Ph-OH (16 mmol, 1.5 g, 1.0 equiv), EDC (17 mmol, 3.3 g, 1.1 equiv), and DMAP (17 mmol, 2.1 g, 1.1 equiv). This mixture was dissolved in anhydrous DCM (27 mL, 0.59 M). This mixture was stirred vigorously under N2 for 16 hours. After the reaction was complete via TLC, the reaction mixture was concentrated, then the crude oil taken up in EtOAc. This was washed with 0.1 M HCl, then the organic layer was washed with saturated sodium bicarbonate, dried over sodium sulfate, and concentrated. The resulting crude oil was purified by silica gel column chromatography (50% EtOAc in hexanes) to provide the title compound as a white powder (1.17 g, 25% yield).

R_f = 0.4 (50% EtOAc in hexanes)

^{1}H NMR (400 MHz, $CDCl_3$) δ 7.40 – 7.32 (m, 2H), 7.25 – 7.20 (m, 1H), 7.13 – 7.08 (m, 2H), 5.47 (d, *J* = 9.1 Hz, 1H), 4.55 – 4.45 (m, 2H), 1.47 (s, 9H), 1.33 (d, *J* = 6.4 Hz, 3H).

^{13}C NMR (101 MHz, $CDCl_3$) δ 170.3, 156.2, 150.5, 129.5, 126.2, 121.4, 80.3, 68.3, 59.0, 28.3, 20.2.

HRMS (+ ESI) calculated for $C_{15}H_{21}O_5N^{23}Na$ [M+Na] 318.1312, found 318.1310.

$[\alpha]_D^{22}$ = -28.1 (c = 1.03, $CHCl_3$).

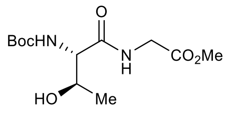

S43

methyl (tert-butoxycarbonyl)-L-threonylglycinate

Prepared according to GP3 using 2-((tert-butoxycarbonyl)amino)-3-hydroxybutanoic acid (36.8 mmol, 8.06 g, 1.0 equiv), glycine methyl ester hydrochloride (40.4 mmol, 5.08 g, 1.0 equiv), HOBt (44.1 mmol, 6.76 g, 1.2 equiv), EDC (47.8 mmol, 9.16 g, 1.3 equiv), DIPEA (140 mmol, 24 mL, 3.8 equiv), and DCM (28 mL, 0.25 M). After 14 hours, the reaction was worked up and purified by silica gel column chromatography (50% EtOAc in hexanes) to afford the title compound as a white solid (3.98 g, 37% yield).

R_f = 0.1 (50% EtOAc in hexanes)

^{1}H NMR (400 MHz, $CDCl_3$) δ 7.02 (s, 1H), 5.45 (d, *J* = 8.1 Hz, 1H), 4.41 (d, *J* = 3.2 Hz, 1H), 4.17 – 4.05 (m, 2H), 3.99 (dd, *J* = 18.2, 5.4 Hz, 1H), 3.76 (s, 3H), 3.04 (s, 1H), 1.46 (s, 9H), 1.21 (d, *J* = 6.5 Hz, 3H).

^{13}C NMR (101 MHz, $CDCl_3$) δ 172.1, 170.3, 156.6, 80.6, 67.1, 58.5, 52.6, 41.3, 28.4, 18.4.

HRMS (+ ESI) calculated for $C_{12}H_{22}O_6N_2{}^{23}Na$ [M+Na] 313.1370, found 313.1366.

$[\alpha]_D^{22}$ = -38.4 (c = 1.24, $CHCl_3$).

2.5 Preparation of Brominated Dipeptides

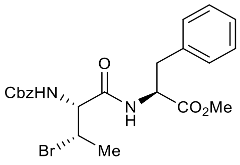

S44

methyl ((2*R*,3*S*)-2-(((benzyloxy)carbonyl)amino)-3-bromobutanoyl)-*L*-phenylalaninate

Prepared according to GP2 using **S37** (4.8 mmol, 2.3 g, 1.0 equiv), CBr_4 (12.7 mmol, 4.22 g, 2.6 equiv), PPh_3 (12.6 mmol, 3.31 g, 2.6 equiv), toluene (140 mL, 0.03 M). After 20 hours, the reaction was worked up and purified by column chromatography (50% EtOAc and hexanes), then trituration from cold EtOAc to afford the title compound as a white solid (78.8 mg, 3% yield, one diastereomer observed).

R_f = 0.5 in 50% EtOAc in hexanes

^{1}H NMR (600 MHz, $CDCl_3$) δ 7.41 – 7.32 (m, 5H), 7.25 – 7.19 (m, 3H), 7.10 – 7.06 (m, 2H), 6.45 (d, *J* = 7.8 Hz, 1H), 5.41 (d, *J* = 6.8 Hz, 1H), 5.14 (s, 2H), 4.89 – 4.83 (m, 1H), 4.45 – 4.38 (m, 1H), 4.30 (dq, *J* = 6.8, 6.7 Hz, 1H), 3.73 (s, 3H), 3.16 (dd, *J* = 13.9, 5.7 Hz, 1H), 3.07 (dd, *J* = 13.9, 6.2 Hz, 1H), 1.67 (d, *J* = 6.6 Hz, 3H).

^{13}C NMR (151 MHz, CDCl$_3$) δ 171.4, 168.2, 156.0, 136.0, 135.6, 129.4, 128.8, 128.8, 128.5, 128.3, 127.4, 67.7, 60.7, 53.4, 52.6, 47.8, 38.0, 21.9.

HRMS (+ p APCI) calculated for $C_{22}H_{26}O_5N_2{}^{79}Br$ [M+H] 477.1020, found 477.1023.

$[\alpha]_D^{22}$ = +18.1 (c = 0.33, CHCl$_3$).

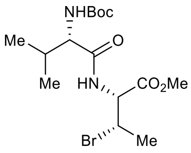

24

methyl (2*R*)-3-bromo-2-((*S*)-2-((tert-butoxycarbonyl)amino)-3-methylbutanamido)butanoate

Prepared according to GP2 using **S38** (10.5 mmol, 3.5 g, 1.0 equiv), CBr_4 (15.7 mmol, 5.22 g, 1.5 equiv), PPh_3 (15.7 mmol, 4.12 g, 1.5 equiv), toluene (105 mL, 0.1 M). After 16 hours, the reaction was worked up and purified by column chromatography (25% EtOAc and hexanes) to afford the title compound as a white solid in a 6:1 mixture of diastereomers (1.39 g, 33% yield).

R_f = 0.3 in 25% EtOAc in hexanes

"^" indicates major diastereomer

"*" indicates minor diastereomer

^{1}H NMR (400 MHz, CDCl$_3$) δ 6.80^ (d, *J* = 7.9 Hz, 0.75H), 6.52* (d, *J* = 9.3 Hz, 0.13H), 5.04* (d, *J* = 7.3 Hz, 0.10H), 5.02 – 4.92^* (m, 0.77H), 4.81^* (dd, *J* = 7.9, 3.5 Hz, 0.83H), 4.72* (qd, *J* = 7.1, 2.6 Hz, 0.15H), 4.36^ (qd, *J* = 7.1, 3.5 Hz, 0.86H), 4.08 – 3.93^* (m, 0.86H), 3.81^ (s, 2.54H), 3.78* (s, 0.50H), 2.29 – 2.13^* (m, 1.01H), 1.82^ (d, *J* = 7.0 Hz, 2.52H), 1.69* (d, *J* = 6.9 Hz, 0.48H), 1.46^* (s, 9.17H), 1.04 – 0.89^* (m, 6.06H).

One diastereomer visible in ^{13}C NMR

^{13}C NMR (201 MHz, CDCl$_3$) δ 172.4, 171.5, 169.5, 169.0, 155.9, 80.4, 80.2, 60.3, 60.1, 58.0, 57.2, 53.1, 52.9, 50.3, 49.0, 30.9, 30.7, 28.4, 23.3, 23.1, 19.4, 19.4, 17.9, 17.7.

HRMS (+ ESI) calculated for $C_{15}H_{27}O_5N_2{}^{79}Br^{23}Na$ [M+Na] 417.0996, found 417.0999.

$[\alpha]_D^{22}$ = +12.5 (c = 0.71, CHCl$_3$).

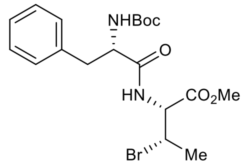

S45

methyl (2*R*)-3-bromo-2-((*S*)-2-((tert-butoxycarbonyl)amino)-3-phenylpropanamido)butanoate

Prepared according to GP2 using **S39** (7.2 mmol, 2.8 g, 1.0 equiv), CBr_4 (10.9 mmol, 3.6 g, 1.5 equiv), PPh_3 (10.9 mmol, 2.85 g, 1.5 equiv), toluene (72 mL, 0.1 M). After 16 hours, the reaction was worked up and purified by column chromatography (25% EtOAc and hexanes) to afford the title compound as a white solid in an 8:1 mixture of diastereomers (772 mg, 24% yield).

$\mathbf{R_f}$ = 0.4 in 25% EtOAc in hexanes

"^" indicates major diastereomer

"*" indicates minor diastereomer

^{1}H NMR (800 MHz, $CDCl_3$) δ 7.26 – 7.22^* (m, 2H), 7.17^* (td, J = 7.9, 6.2 Hz, 1H), 7.15 – 7.12^* (m, 2H), 6.71^ (s, 0.97H), 6.62* (s, 0.12H), 4.88^* (s, 1H), 4.84* (dd, J = 9.1, 2.5 Hz, 0.13H), 4.68^ (dd, J = 7.9, 3.9 Hz, 0.95H), 4.61* (qd, J = 6.9, 2.5 Hz, 0.13H), 4.41 – 4.35* (m, 0.10H), 4.35 – 4.28^ (m, 0.88H), 4.24^* (qd, J = 7.0, 3.8 Hz, 1.02H), 3.69^* (s, 3H), 3.11* (dd, J = 14.1, 6.3 Hz, 0.14H), 3.06 – 2.98^* (m, 1.97H), 1.70^ (d, J = 7.0 Hz, 2.56H), 1.58* (d, J = 6.9 Hz, 0.39H), 1.36^* (s, 9H).

^{13}C data includes signals from both diastereomers (8:1 dr)

^{13}C NMR (201 MHz, $CDCl_3$) δ 172.0, 171.2, 169.4, 168.7, 155.5, 136.4, 129.5, 129.4, 128.9, 128.9, 127.2, 80.6, 58.1, 57.3, 55.8, 53.1, 52.8, 50.3, 48.8, 38.0, 28.4, 23.3, 22.9.

HRMS (- APCI) calculated for $C_{19}H_{27}O_5N_2{}^{79}Br^{35}Cl$ [M+Cl-] 477.0797, found 477.0801.

$[\alpha]_D^{22}$ = +8.8 (c = 1.04, $CHCl_3$).

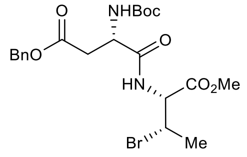

S46

benzyl (*S*)-4-(((2*R*,3*S*)-3-bromo-1-methoxy-1-oxobutan-2-yl)amino)-3-((tert-butoxycarbonyl)amino)-4-oxobutanoate

Prepared according to GP2 using **S40** (6.8 mmol, 3.00 g, 1.0 equiv), CBr_4 (8.1 mmol, 2.67 g, 1.2 equiv), PPh_3 (12.9 mmol, 3.38 g, 1.9 equiv), toluene (68 mL, 0.10 M). After 16 hours, the reaction was worked up and purified by column chromatography (25% EtOAc and hexanes) to

afford the title compound as a yellow/white solid in a 11:1 mixture of diastereomers (650 mg, 19% yield).

"^" indicates major diastereomer

"*" indicates minor diastereomer

R_f = 0.25 in 25% EtOAc in hexanes

^{1}H NMR (400 MHz, $CDCl_3$) δ 7.38 – 7.32^* (m, 5.49H), 5.71^* (d, J = 8.9 Hz, 0.80H), 5.20 – 5.10^* (m, 1.98H), 4.89* (dd, J = 9.3, 2.5 Hz, 0.08H), 4.78^ (dd, J = 8.4, 3.9 Hz, 0.851H), 4.70* (qd, J = 6.9, 2.4 Hz, 0.08H), 4.65 – 4.52^* (m, 0.83H), 4.30^ (qd, J = 7.0, 3.9 Hz, 0.87H), 3.78^ (s, 2.62H), 3.76* (s, 0.26H), 3.06^* (dd, (major) J = 17.1, 4.6 Hz, 0.96H), 2.75^* (dd, (major) J = 17.2, 5.9 Hz, 0.94H), 1.78^ (d, J = 7.0 Hz, 2.63H), 1.67* (d, J = 6.9 Hz, 0.23H), 1.46^* (s, 8.75H).

^{13}C data includes signals from both diastereomers (11:1 dr)

^{13}C NMR (151 MHz, $CDCl_3$) δ 171.7, 171.4, 170.6, 169.2, 168.7, 155.6, 135.4, 128.6, 128.4, 128.4, 128.3, 80.8, 67.0, 58.1, 57.3, 53.4, 53.0, 52.7, 50.6, 50.1, 48.4, 35.7, 28.3, 23.1, 22.7.

HRMS (+ ESI) calculated for $C_{21}H_{29}O_7N_2{}^{79}Br^{23}Na$ [M+Na] 523.1050, found 523.1054.

$[\alpha]_D^{22}$ = +7.8 (c = 0.74, $CHCl_3$).

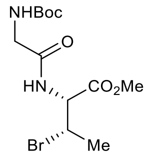

S47

methyl (2*R*)-3-bromo-2-(2-((tert-butoxycarbonyl)amino)acetamido)butanoate

Prepared according to GP2 using **S4** (1.76 mmol, 512 mg, 1.0 equiv), CBr_4 (2.64 mmol, 876 mg, 1.5 equiv), PPh_3 (2.64 mmol, 692 mg, 1.5 equiv), toluene (17 mL, 0.1 M). After 16 hours, the reaction was worked up and purified by column chromatography (50% EtOAc and hexanes) to afford the title compound as a colorless oil in a 5:1 mixture of diastereomers (230 mg, 37% yield).

R_f = 0.3 in 50% EtOAc in hexanes

"^" indicates major diastereomer

"*" indicates minor diastereomer

^{1}H NMR (400 MHz, $CDCl_3$) δ 6.98^ (d, J = 7.6 Hz, 0.68H), 6.83* (d, J = 9.3 Hz, 0.12H), 5.12^* (br s, 1H), 4.95* (dd, J = 9.4, 2.4 Hz, 0.15H), 4.83^ (dd, J = 8.2, 3.6 Hz, 0.75H), 4.72* (qd, J = 6.9, 2.5 Hz, 0.14H), 4.35^ (qd, J = 7.0, 3.6 Hz, 0.78H), 4.01 – 3.91* (m, 0.21H), 3.90 – 3.82^* (m, 1.81H), 3.81^ (s, 2.43H), 3.79* (s, 0.47H), 1.81^ (d, J = 7.0 Hz, 2.37H), 1.70* (d, J = 6.9 Hz, 0.59H), 1.47^* (s, 9H).

^{13}C data includes signals from both diastereomers (5:1 dr)

^{13}C NMR (151 MHz, CDCl$_3$) δ 170.2, 169.6, 169.4, 169.0, 156.2, 80.7, 57.9, 57.0, 53.2, 52.9, 50.5, 49.1, 44.6, 28.5, 28.4, 23.3, 23.1.

HRMS (+ ESI) calculated for $C_{12}H_{21}O_5N_2{}^{79}Br^{23}Na$ [M+Na] 375.0526, found 375.0528.

$[\alpha]_D^{23}$ = +33.6 (c = 0.25, MeOH).

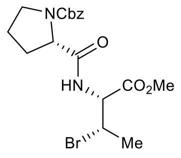

S48

benzyl (2*S*)-2-(((2*R*)-3-bromo-1-methoxy-1-oxobutan-2-yl)carbamoyl)pyrrolidine-1-carboxylate

Prepared according to GP2 using **S41** (5.0 mmol, 1.82 g, 1.0 equiv), CBr_4 (5.9 mmol, 1.97 g, 1.2 equiv), PPh_3 (9.5 mmol, 2.48 g, 1.9 equiv), toluene (50 mL, 0.10 M). After 16 hours, the reaction was worked up and purified by column chromatography (25-50% EtOAc and hexanes) to afford the title compound as a white solid (629 mg, 29% yield).

Reported as a mixture of rotamers and diastereomers

^{1}H NMR (400 MHz, CDCl$_3$) δ 7.60 – 7.27 (m, 5H), 6.88 – 6.55 (m, 0H), 5.31 – 5.04 (m, 2H), 4.96 – 4.62 (m, 1H), 4.51 – 4.17 (m, 2H), 3.86 – 3.34 (m, 5H), 2.45 – 2.07 (m, 1H), 2.07 – 1.82 (m, 3H), 1.73 (d, J = 7.0 Hz, 2H), 1.68 – 1.53 (m, 1H).

^{13}C NMR (101 MHz, CDCl$_3$) δ 171.5, 171.1, 170.9, 167.4, 167.1, 166.3, 156.4, 155.1, 154.6, 136.6, 136.5, 128.6, 128.3, 128.3, 128.2, 128.1, 128.1, 128.0, 127.8, 127.8, 68.9, 68.6, 67.5, 67.4, 67.3, 60.6, 59.9, 59.1, 58.9, 57.3, 57.1, 56.8, 54.0, 53.6, 53.1, 47.3, 46.8, 31.3, 30.1, 29.8, 28.8, 24.7, 24.5, 23.6, 23.4, 18.1, 17.4, 17.1.

R_f = 0.2 in 25% EtOAc in hexanes

HRMS (+ APCI) calculated for $C_{18}H_{24}O_5N_2{}^{79}Br$ [M+H] 427.0863, found 427.0864.

$[\alpha]_D^{22}$ = -15.3 (c = 2.49, CHCl$_3$).

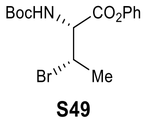

S49

phenyl (2R,3S)-3-bromo-2-((tert-butoxycarbonyl)amino)butanoate

Prepared according to GP2 using **S42** (4.0 mmol, 1.17 g, 1.0 equiv), CBr_4 (5.9 mmol, 1.97 g, 1.5 equiv), PPh_3 (5.9 mmol, 1.56 g, 1.5 equiv), toluene (50 mL, 0.08 M). After 15 hours, the reaction was worked up and purified by column chromatography (10% EtOAc and hexanes) to afford the title compound as a white solid (410 mg, 29% yield, one diastereomer observed).

R_f = 0.4 in 15% EtOAc in hexanes.

^{1}**H NMR (400 MHz, CDCl$_3$)** δ 7.45 – 7.36 (m, 2H), 7.30 – 7.23 (m, 2H, *includes chloroform peak*), 7.19 – 7.09 (m, 2H), 5.49 (d, *J* = 8.7 Hz, 1H), 4.78 (dd, *J* = 8.7, 3.5 Hz, 1H), 4.45 (dt, *J* = 10.5, 5.2 Hz, 1H), 1.94 (d, *J* = 7.1 Hz, 3H), 1.48 (s, 9H).

^{13}**C NMR (101 MHz, CDCl$_3$)** δ 167.7, 150.2, 129.6, 126.5, 121.4, 80.8, 77.4, 77.0, 76.7, 59.5, 50.0, 28.3, 23.3.

HRMS (+ APCI) calculated for $C_{10}H_{13}O_2N^{79}Br$ [M+H -Boc] 258.0124, found 258.0124.

$[\alpha]_D^{22}$ = -4.7 (c = 0.30, CHCl$_3$).

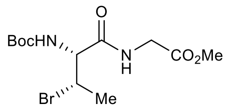

S50

methyl ((2R,3S)-3-bromo-2-((tert-butoxycarbonyl)amino)butanoyl)glycinate

Prepared according to GP2 using **S43** (8.3 mmol, 2.4 g, 1.0 equiv), CBr$_4$ (12.5 mmol, 4.1 g, 1.5 equiv), PPh$_3$ (12.5 mmol, 3.3 g, 1.5 equiv), toluene (150 mL, 0.05 M). After 20 hours, the reaction was worked up and purified by column chromatography (25% EtOAc and hexanes) to afford the title compound as a white solid (1.445 g, 49% yield, one diastereomer observed).

$\mathbf{R_f}$ = 0.5 in 50% EtOAc in hexanes.

^{1}**H NMR (400 MHz, CDCl$_3$)** δ 6.59 (s, 1H), 5.19 (s, 1H), 4.46 – 4.35 (m, 2H), 4.12 (dd, *J* = 18.4, 5.4 Hz, 1H), 4.03 (dd, *J* = 18.4, 5.0 Hz, 1H), 3.77 (s, 3H), 1.76 (d, *J* = 6.7 Hz, 3H), 1.47 (s, 9H).

^{13}**C NMR (101 MHz, CDCl$_3$)** δ 170.0, 169.3, 155.5, 81.0, 60.1, 52.6, 48.3, 41.3, 28.4, 21.8.

HRMS (+ ESI) calculated for $C_{12}H_{21}O_5N_2{}^{79}Br^{23}Na$ [M+Na+] 375.0526, found 375.0523.

$[\alpha]_D^{22}$ = -8.9 (c = 1.13, CHCl$_3$).

3. Preparation of Cross-Coupled Products

General Procedure 4: Light-mediated cross-coupling

In an N$_2$-filled glovebox, NiCl$_2$•glyme (10 mol%), (4S,4'S)-4,4'-diisopropyl-4,4',5,5'-tetrahydro-2,2'-bioxazole (10 mol%), Cs$_2$CO$_3$ (2 equiv), and DME were added to a 1-dram vial with magnetic stir bar. This pre-catalyst mixture was stirred for 10 minutes. To a separate 1-dram vial was added Ir[(dF(CF$_3$)ppy)$_2$dtbbpy]PF$_6$ (1 mol%), the aryl bromide (1.0 equiv), and the alkyl bromide (1.5 equiv). This was solvated in DME and transferred to the pre-catalyst mixture, rinsing the vial with an equivalent amount of DME, to reach a total concentration of 0.1 M. To this was added 1,1,1,3,3,3-hexamethyl-2-(trimethylsilyl)trisilane (1.6 equiv). The vial was capped, sealed with electrical tape, and transported out of the glovebox and to a Penn photoreactor. The reaction mixture was irradiated at 10% light intensity with 1000 rpm stirring and 6800 rpm fan for 16 hours. Upon completion, the reaction was filtered through a plug of celite, rinsing with EtOAc 3x, and purified via silica gel column chromatography.

Diastereoselectivity determination

The diastereomeric ratio for the products described herein were obtained through crude ^{1}H NMR, unless otherwise specified. The dr of compound **7** was determined by crude ^{1}H NMR comparison of protons giving aryl singlet signals of the two diastereomers (minor 6.90 ppm, major 6.95 ppm).

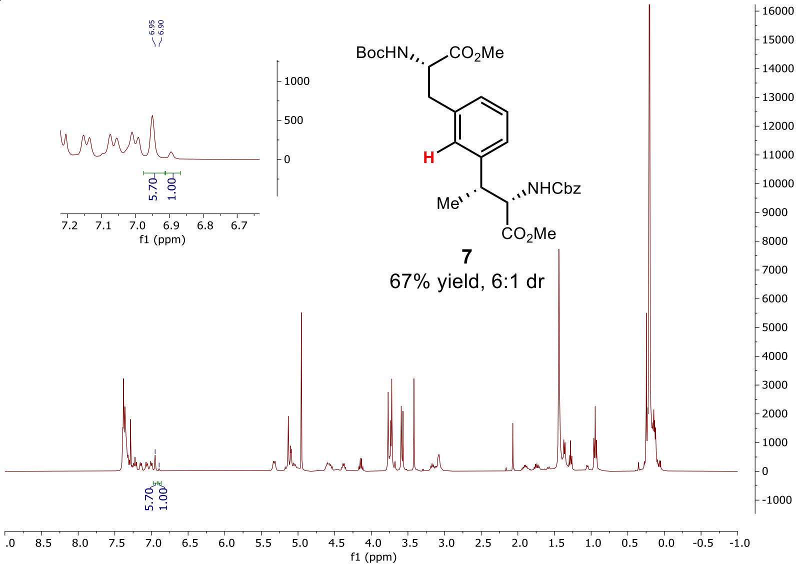

The dr of compounds **S52**, **23**, **10**, and **16** were determined by crude ^{1}H NMR comparison of protons giving aryl singlet signals of the two diastereomers (for **10**, minor 7.77 ppm, major 7.81 ppm). An example of this is provided below for **10**.

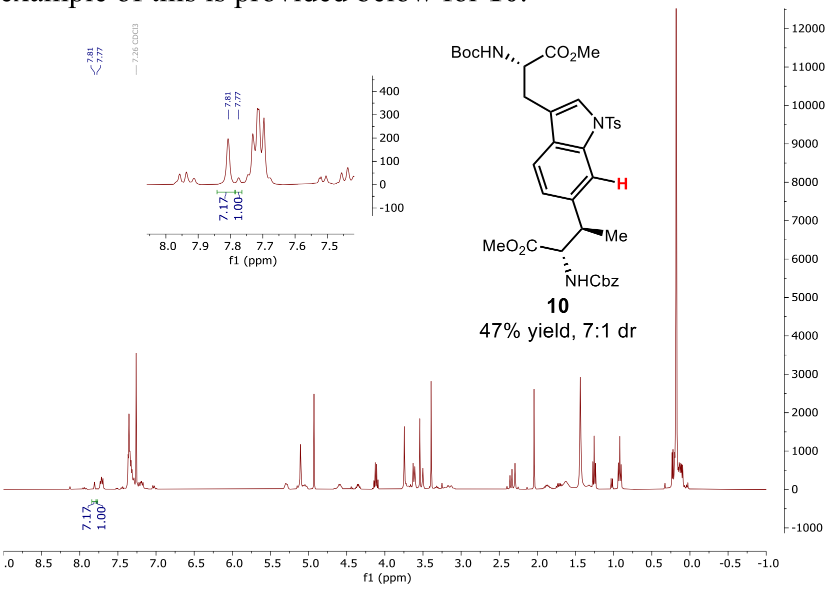

The dr of compounds **13**, **18**, and **21** were determined by crude ^{1}H NMR comparison of the alpha amine protons of the two diastereomers (minor 4.75 ppm, major 4.61 ppm for **13**). An example of this is provided below for **13**.

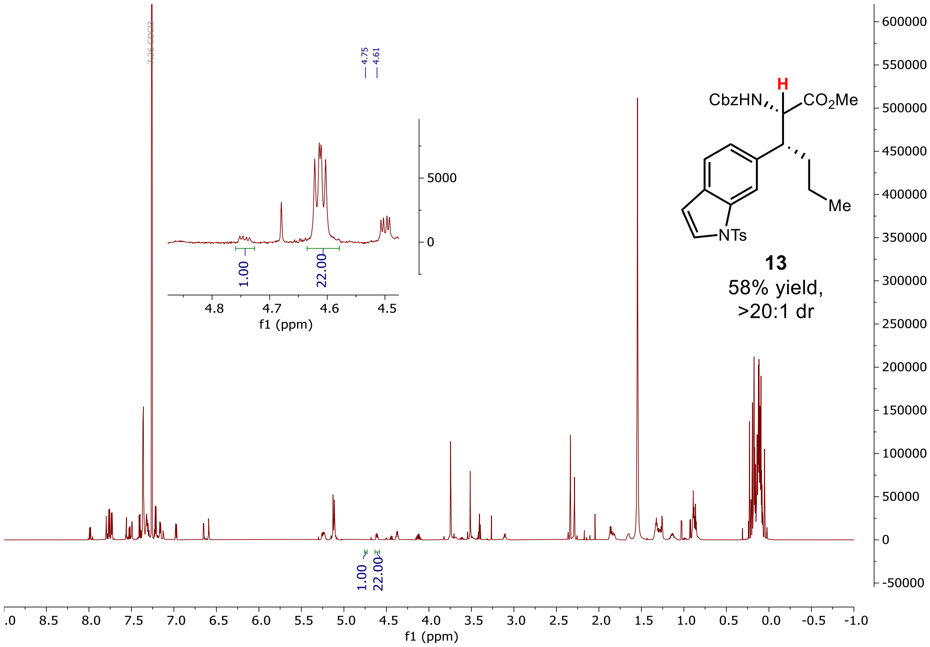

The dr of compounds **12** and **14** were determined by crude ^{1}H NMR comparison of the tosyl methyl protons giving alkyl singlet signals of the two diastereomers (minor ~2.25 ppm, major ~2.28 ppm). An example of this is provided below for **14**.

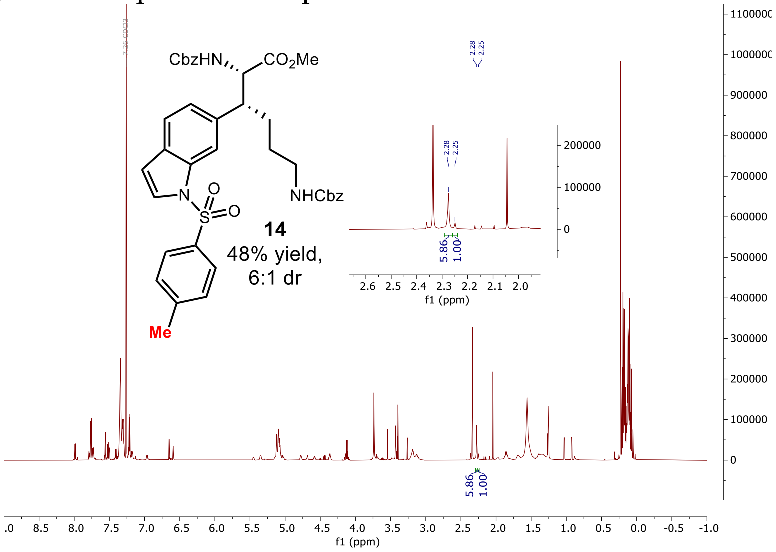

The dr of compounds **17**, **19**, and **22** were determined by crude ^{1}H NMR comparison of the NH doublet signals of the two diastereomers (major 6.39 ppm, minor 6.24 ppm for **17**). An example of this is provided below for **17**.

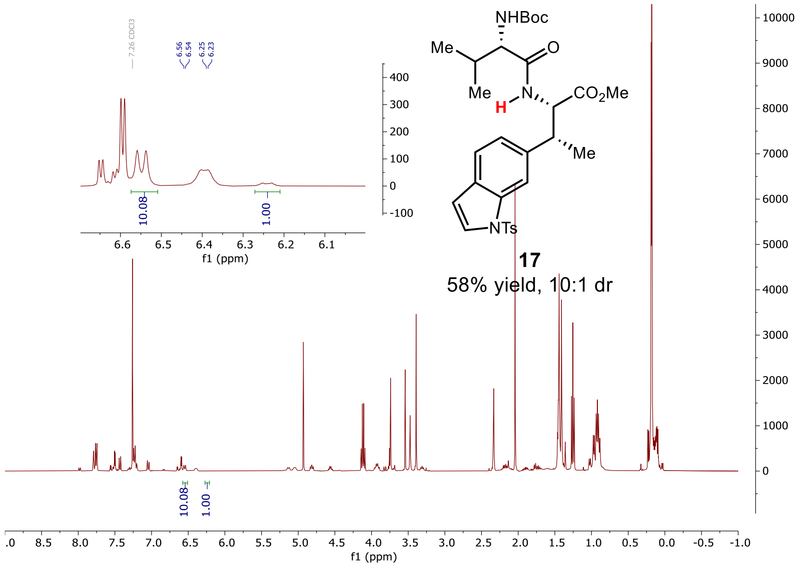

The dr of compound **20** was determined by crude ^{1}H NMR comparison of the aryl doublet signals of the two diastereomers (major 7.44 ppm, minor 7.47 ppm). An example of this is provided below for **20**.

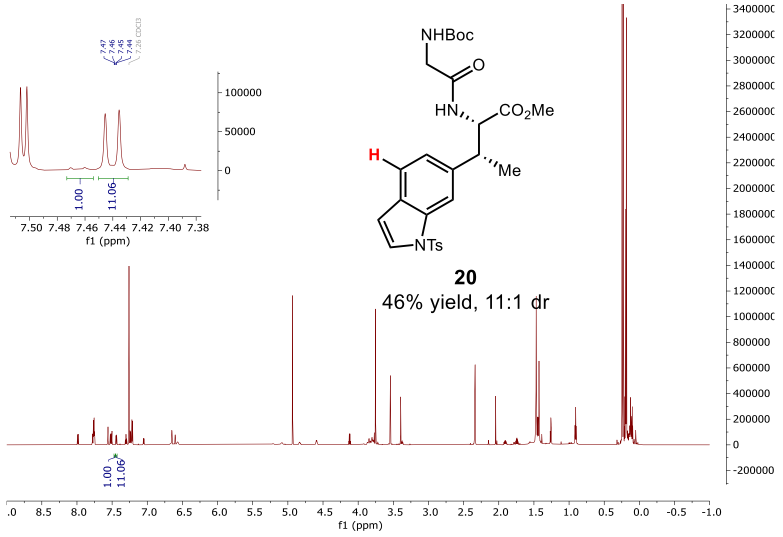

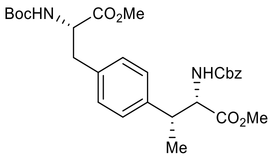

6

methyl (2*S*,3*R*)-2-(((benzyloxy)carbonyl)amino)-3-(4-((*S*)-2-((tert-butoxycarbonyl)amino)-3-methoxy-3-oxopropyl)phenyl)butanoate

Prepared according to GP4 using $NiCl_2$•glyme (10 mol%, 2.2 mg), (4S,4'S)-4,4'-diisopropyl-4,4',5,5'-tetrahydro-2,2'-bioxazole (10 mol%, 2.2 mg), Cs_2CO_3 (2 equiv, 64.3 mg), Ir[(dF(CF_3)ppy)$_2$dtbbpy]PF_6 (1 mol%, 1.0 mg), ArBr **S5** (1.0 equiv, 35.6 mg), alkyl bromide **S2** (1.5 equiv, 50.1 mg), and 1,1,1,3,3,3-hexamethyl-2-(trimethylsilyl)trisilane (1.6 equiv, 49.4 µL). After 16 hours, the reaction was worked up and purified by silica gel column

chromatography (15-20% acetone in pentane) to afford the title compound as a white powder with 6:1 dr (37.4 mg, 66% yield, 12:1 isolated dr).

R$_f$ = 0.25 (20% acetone in pentane)

"^" indicates major diastereomer

"*" indicates minor diastereomer

^{1}H NMR (800 MHz, CDCl$_3$) δ 7.38 – 7.28^* (m, 5H), 7.10^* (d, J = 7.8 Hz, 2H), 7.04^* (d, J = 7.8 Hz, 2H), 5.25^* (d, J = 9.2 Hz, 1H), 5.08^* (q, J = 12.2 Hz, 2H), 4.94^* (d, J = 8.6 Hz, 1H), 4.55^* (q, J = 6.8 Hz, 1H), 4.53 – 4.48^* (m, 1H), 3.74* (d, J = 2.5 Hz, 0.33H), 3.70^ (s, 3.02H), 3.68* (s, 0.38H), 3.55^ (s, 2.88H), 3.36* (p, J = 6.9 Hz, 0.09H), 3.15^ (p, J = 6.7 Hz, 1.02H), 3.07^* (dd, J = 14.0, 5.9 Hz, 1H), 3.01^* (dd, J = 14.4, 6.2 Hz, 1H), 1.44 – 1.36^* (m, 9H), 1.34^* (d, J = 7.2 Hz, 3H).

Only major diastereomer visible in ^{13}C NMR

^{13}C NMR (201 MHz, CDCl$_3$) δ 172.3, 171.9, 155.8, 155.1, 139.9, 136.2, 134.9, 129.4, 128.6, 128.2, 128.2, 127.8, 80.0, 67.1, 59.6, 54.4, 52.2, 52.0, 42.6, 38.0, 28.3, 16.7.

HRMS (+ p APCI) calculated for $C_{28}H_{36}O_8N_2{}^{23}Na$ [M+Na] 551.2364, found 551.2370.

$[\alpha]_D^{22}$ = +2.9 (c = 0.17, CHCl$_3$).

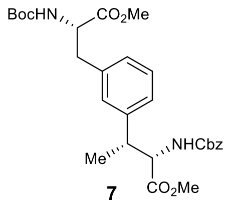

7

methyl (2*S*,3*R*)-2-(((benzyloxy)carbonyl)amino)-3-(3-((*S*)-2-((tert-butoxycarbonyl)amino)-3-methoxy-3-oxopropyl)phenyl)butanoate

Prepared according to GP4 using NiCl$_2$•glyme (10 mol%, 2.6 mg), (4S,4'S)-4,4'-diisopropyl-4,4',5,5'-tetrahydro-2,2'-bioxazole (10 mol%, 2.0 mg), Cs$_2$CO$_3$ (2 equiv, 69.8 mg), Ir[(dF(CF$_3$)ppy)$_2$dtbbpy]PF$_6$ (1 mol%, 1.6 mg), ArBr **S19** (1.0 equiv, 37.0 mg), alkyl bromide **S2** (1.5 equiv, 53.7 mg), and 1,1,1,3,3,3-hexamethyl-2-(trimethylsilyl)trisilane (1.6 equiv, 49.4 μL). After 16 hours, the reaction was worked up and purified by silica gel column chromatography (10-20% acetone in hexanes) to afford the title compound as a white foam with 6:1 dr (36.5 mg, 67% yield, 5:1 isolated dr).

R$_f$ = 0.2 (20% acetone in hexanes)

"^" indicates major diastereomer

"*" indicates minor diastereomer

^{1}H NMR (600 MHz, CDCl$_3$) δ 7.39 – 7.28^* (m, 5H), 7.20^* (t, J = 7.6 Hz, 1H), 7.04^* (d, J = 7.7 Hz, 1H), 6.98^* (d, J = 7.6 Hz, 1H), 6.93^ (s, 0.84H), 6.88* (s, 0.18H), 5.28^* (d, J = 9.3 Hz, 1H), 5.08^* (t, J = 10.3 Hz, 2H), 5.03 – 4.96^* (m, 1H), 4.61 – 4.50^* (m, 2H), 3.72* (s,

0.22H), 3.70^ (s, 2.91H), 3.65* (s, 0.43H), 3.57^ (s, 2.50H), 3.38 – 3.31* (m, 0.18H), 3.15^ (p, J = 7.1 Hz, 0.95H), 3.06^* (t, J = 6.1 Hz, 2H), 1.42^* (d, J = 3.4 Hz, 9H), 1.35^* (t, J = 7.5 Hz, 3H).

^{13}C data includes signals from both diastereomers (5:1 dr)

^{13}C NMR (201 MHz, $CDCl_3$) δ 172.4, 171.9, 155.9, 155.3, 141.5, 136.4, 136.3, 128.8, 128.8, 128.7, 128.7, 128.4, 128.3, 128.3, 126.6, 80.1, 67.2, 59.7, 54.5, 52.4, 52.3, 43.0, 38.2, 28.5, 16.8.

HRMS (+ p APCI) calculated for $C_{28}H_{36}O_8N_2{}^{23}Na$ [M+Na] 551.2364, found 551.2373.

$[\alpha]_D^{22}$ = +44.0 (c = 1.57, $CHCl_3$).

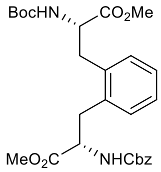

8b

methyl (*S*)-2-(((benzyloxy)carbonyl)amino)-3-(2-((*S*)-2-((tert-butoxycarbonyl)amino)-3-methoxy-3-oxopropyl)phenyl)propanoate

Prepared according to GP4 using $NiCl_2$•glyme (10 mol%, 2.8 mg), (4S,4'S)-4,4'-diisopropyl-4,4',5,5'-tetrahydro-2,2'-bioxazole (10 mol%, 2.2 mg), Cs_2CO_3 (2 equiv, 67.7 mg), Ir[(dF(CF_3)ppy)$_2$dtbbpy]PF_6 (1 mol%, 1.2 mg), ArBr **S20** (1.0 equiv, 43.8 mg), alkyl bromide **S2** (1.5 equiv, 48.8 mg), and 1,1,1,3,3,3-hexamethyl-2-(trimethylsilyl)trisilane (1.6 equiv, 49.4 µL). After 16 hours, the reaction was worked up and purified by silica gel column chromatography (20% acetone in pentane) to afford the title compound as a white foam (16.5 mg, 29% yield).

R_f = 0.25 (20% acetone in pentane)

^{1}H NMR (400 MHz, $CDCl_3$) δ 7.38 – 7.26 (m, 5H), 7.20 – 7.13 (m, 2H), 7.13 – 7.04 (m, 2H), 5.53 (d, J = 8.1 Hz, 1H), 5.13 – 4.98 (m, 3H), 4.67 (q, J = 7.6 Hz, 1H), 4.59 (q, J = 7.9 Hz, 1H), 3.76 – 3.61 (m, 6H), 3.26 – 3.13 (m, 2H), 3.06 (dd, J = 14.6, 8.2 Hz, 1H), 2.92 (dd, J = 14.2, 8.0 Hz, 1H), 1.31 (s, 9H).

^{13}C NMR (201 MHz, $CDCl_3$) δ 172.7, 172.6, 156.0, 155.2, 136.4, 135.0, 134.9, 130.6, 129.8, 128.6, 128.2, 127.4, 127.3, 80.2, 67.1, 54.3, 53.9, 52.5, 52.4, 35.7, 34.8, 29.8, 28.3.

HRMS (+ p APCI) calculated for $C_{27}H_{34}O_8N_2{}^{23}Na$ [M+Na] 537.2207, found 537.2209.

$[\alpha]_D^{21}$ = +13.8 (c = 0.63, $CHCl_3$).

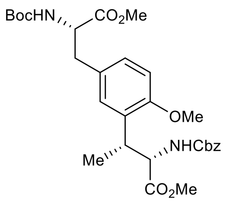

9a

methyl (2*S*,3*R*)-2-(((benzyloxy)carbonyl)amino)-3-(5-((*S*)-2-((tert-butoxycarbonyl)amino)-3-methoxy-3-oxopropyl)-2-methoxyphenyl)butanoate

Prepared according to GP4 using $NiCl_2$•glyme (20 mol%, 3.1 mg), (4S,4'S)-4,4'-diisopropyl-4,4',5,5'-tetrahydro-2,2'-bioxazole (20 mol%, 4.55 mg), Cs_2CO_3 (2 equiv, 65.9 mg), Ir[(dF(CF_3)ppy)$_2$dtbbpy]PF_6 (1 mol%, 1.4 mg), ArBr **S6** (1.0 equiv, 42.84 mg), alkyl bromide **S2** (1.5 equiv, 50.13 mg), and 1,1,1,3,3,3-hexamethyl-2-(trimethylsilyl)trisilane (1.6 equiv, 49.4 μL). After 16 hours, the reaction was worked up and purified by silica gel column chromatography (15-20% acetone in pentane, then 5% acetone in toluene) to afford the title compound as a white foam with 9:1 isolated dr (7.6 mg, 11% yield).

R_f = 0.3 (15% acetone in pentane)

"^" indicates major diastereomer

"*" indicates minor diastereomer

^{1}H NMR (800 MHz, $CDCl_3$, 50 °C) δ 7.37 – 7.27^* (m, 5H), 6.95^* (dd, J = 8.3, 2.2 Hz, 1H), 6.90* (s, 0.10H), 6.84^ (s, 0.86H), 6.80 – 6.73^* (m, 1H), 5.33^ (br s, 0.71H), 5.25* (br s, 0.16H), 5.12* (q, J = 2.0 Hz, 0.31H), 5.04^ (br s, 1.76H), 4.98^* (br s, 1H), 4.71^* (m, 1H), 4.53^ (br s, 0.74H), 4.48* (br s, 0.17H), 3.79^* (d, J = 38.0 Hz, 3H), 3.70^* (d, J = 1.4 Hz, 3H), 3.68 – 3.56^* (m, 4H), 3.07 – 2.92^* (m, 2H), 1.43^* (s, 9H), 1.32* (d, J = 7.2 Hz, 0.60H), 1.27^ (br s, 3.43H).

^{13}C data includes signals from both diastereomers (9:1 dr)

^{13}C NMR (201 MHz, $CDCl_3$) δ 172.3, 172.3, 156.4, 156.0, 155.2, 136.5, 129.5, 129.3, 129.0, 128.9, 128.7, 128.6, 128.6, 128.4, 128.3, 128.2, 128.2, 127.6, 110.7, 80.0, 67.0, 59.0, 57.8, 55.6, 55.6, 54.6, 52.3, 52.3, 52.2, 52.1, 37.6, 37.4, 35.7, 35.0, 29.8, 28.5, 17.4, 15.3.

HRMS (+ p APCI) calculated for $C_{29}H_{38}O_9N_2{}^{23}Na$ [M+Na] 581.2470, found 581.2475.

$[\alpha]_D^{22}$ = -254.1 (c = 0.60, $CHCl_3$).

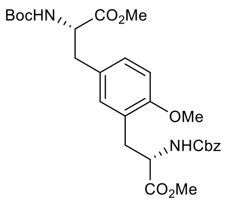

9b

methyl (*S*)-2-(((benzyloxy)carbonyl)amino)-3-(5-((*S*)-2-((tert-butoxycarbonyl)amino)-3-methoxy-3-oxopropyl)-2-methoxyphenyl)propanoate

Prepared according to GP4 using $NiCl_2$•glyme (10 mol%, 2.4 mg), (4S,4'S)-4,4'-diisopropyl-4,4',5,5'-tetrahydro-2,2'-bioxazole (10 mol%, 2.2 mg), Cs_2CO_3 (2 equiv, 66.2 mg), $Ir[(dF(CF_3)ppy)_2dtbbpy]PF_6$ (1 mol%, 1.1 mg), ArBr **S6** (1.0 equiv, 41.5 mg), alkyl bromide **S2** (1.5 equiv, 47.7 mg), and 1,1,1,3,3,3-hexamethyl-2-(trimethylsilyl)trisilane (1.6 equiv, 49.4 µL). After 16 hours, the reaction was worked up and purified by silica gel column chromatography (20% acetone in pentane, then 5-10% acetone in toluene) to afford the title compound as a white powder (16.5 mg, 28% yield).

R_f = 0.25 (20% acetone in pentane)

"#" indicates rotamer

^{1}H NMR (600 MHz, $CDCl_3$, 25 °C) δ 7.36 – 7.27 (m, 5H), 6.97 (dd, J = 8.3, 2.2 Hz, 1H), 6.81 (s, 1H), 6.76 (d, J = 8.4 Hz, 1H), 5.46 (d, J = 7.9 Hz, 0.81H), 5.26# (s, 0.13H), 5.06 (s, 2H), 4.97 (d, J = 8.4 Hz, 0.67H), 4.91# (d, J = 12.5 Hz, 0.19H), 4.64# (s, 0.11H), 4.60 – 4.54 (m, 0.85H), 4.52 (q, J = 6.5 Hz, 1H), 3.78 (s, 3H), 3.72 (s, 3H), 3.68 (s, 3H), 3.64# (s, 0.46H), 3.12 – 2.88 (m, 4H), 1.42 (s, 9H).

^{1}H NMR (600 MHz, $CDCl_3$, 50 °C) δ 7.35 – 7.27 (m, 5H), 6.98 (dd, J = 8.3, 2.3 Hz, 1H), 6.83 (d, J = 2.3 Hz, 1H), 6.76 (d, J = 8.3 Hz, 1H), 5.41 (s, 1H), 5.07 (s, 2H), 4.95 (s, 0H), 4.63 – 4.42 (m, 2H), 3.77 (s, 3H), 3.71 (s, 3H), 3.68 (s, 3H), 3.09 (dd, J = 13.6, 5.6 Hz, 1H), 3.06 – 2.86 (m, 3H), 1.43 (s, 9H).

^{13}C NMR (201 MHz, $CDCl_3$) δ 172.6, 172.4, 156.8, 155.9, 155.2, 136.6, 132.4, 129.4, 128.6, 128.2, 128.1, 128.0, 124.6, 110.7, 80.1, 66.9, 55.6, 54.7, 54.6, 52.4, 37.5, 33.0, 28.5.

HRMS (+ p APCI) calculated for $C_{28}H_{36}O_9N_2{}^{23}Na$ [M+Na] 567.2313, found 567.2319.

$[\alpha]_D^{21}$ = +15.7 (c = 0.31, $CHCl_3$).

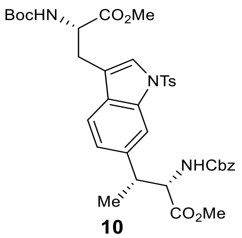

10

methyl (2*S*,3*R*)-2-(((benzyloxy)carbonyl)amino)-3-(3-((*S*)-2-((tert-butoxycarbonyl)amino)-3-methoxy-3-oxopropyl)-1-tosyl-1H-indol-6-yl)butanoate

Prepared according to GP4 using $NiCl_2$•glyme (10 mol%, 2.6 mg), (4S,4'S)-4,4'-diisopropyl-4,4',5,5'-tetrahydro-2,2'-bioxazole (10 mol%, 2.8 mg), Cs_2CO_3 (2 equiv, 66.3 mg), Ir[(dF(CF$_3$)ppy)$_2$dtbbpy]PF$_6$ (1 mol%, 1.1 mg), ArBr **S21** (1.0 equiv, 55.2 mg), alkyl bromide **S2** (1.5 equiv, 51.1 mg), and 1,1,1,3,3,3-hexamethyl-2-(trimethylsilyl)trisilane (1.6 equiv, 49.4 µL). After 16 hours, the reaction was worked up and purified by silica gel column chromatography (20% acetone in hexanes) to afford the title compound as a white foam with 7:1 dr (34.3 mg, 47% yield, 15:1 isolated dr).

R_f = 0.2 (25% acetone in hexanes)

"^" indicates major diastereomer

"*" indicates minor diastereomer

^{1}H NMR (800 MHz, CDCl$_3$) δ 7.81 (s, ^0.88H), 7.78* (s, 0.06H), 7.71^* (d, J = 8.3 Hz, 2H), 7.38 – 7.27^* (m, 7H), 7.18^* (d, J = 8.1 Hz, 2H), 7.04^ (d, J = 8.1 Hz, 0.93H), 7.01* (d, 0.08H), 5.27^* (d, J = 9.1 Hz, 1H), 5.10^* (d, J = 1.8 Hz, 2H), 5.02^ (d, J = 8.2 Hz, 0.96H), 4.94* (d, 0.06H), 4.67* (dd, J = 9.1, 4.5 Hz, 0.09H), 4.63 – 4.55^* (m, 1.71H, only major of 1H, major and minor of 1H), 3.72* (s, 0.19H), 3.61^ (s, 3.10H), 3.51^* (s, 3H), 3.32^ (p, J = 6.8 Hz, 1.00H), 3.19^* (dd, J = 15.1, 5.8 Hz, 1H), 3.12^* (dd, J = 15.2, 5.4 Hz, 1H), 2.30^ (s, 2.81H), 2.26* (s, 0.16H), 1.47 – 1.36^* (m, 12H).

^{13}C data includes signals from both diastereomers (15:1 dr)

^{13}C NMR (201 MHz, CDCl$_3$) δ 172.1, 171.8, 155.9, 155.1, 145.0, 138.4, 136.3, 135.4, 135.3, 130.2, 130.0, 128.7, 128.6, 128.4, 128.3, 127.8, 126.9, 126.8, 124.7, 123.4, 119.5, 117.4, 113.0, 80.3, 67.4, 67.3, 59.8, 59.4, 53.6, 52.5, 52.2, 43.3, 42.3, 29.8, 28.5, 28.0, 21.7, 17.6.

HRMS (- ESI) calculated for $C_{37}H_{43}O_{10}N_3{}^{35}Cl^{32}S$ [M+Cl] 756.2363, found 756.2370.

$[\alpha]_D^{21}$ = +32.7 (c = 0.25, CHCl$_3$).

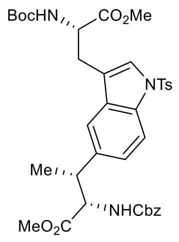

11

methyl (2*S*,3*R*)-2-(((benzyloxy)carbonyl)amino)-3-(3-((*S*)-2-((tert-butoxycarbonyl)amino)-3-methoxy-3-oxopropyl)-1-tosyl-1H-indol-5-yl)butanoate

Prepared according to GP4 using $NiCl_2$•glyme (10 mol%, 2.6 mg), (4S,4'S)-4,4'-diisopropyl-4,4',5,5'-tetrahydro-2,2'-bioxazole (10 mol%, 2.6 mg), Cs_2CO_3 (2 equiv, 63.6 mg), $Ir[(dF(CF_3)ppy)_2dtbbpy]PF_6$ (1 mol%, 1.8 mg), ArBr **S22** (1.0 equiv, 56.1 mg), alkyl bromide **S2** (1.5 equiv, 49.0 mg), and 1,1,1,3,3,3-hexamethyl-2-(trimethylsilyl)trisilane (1.6 equiv, 49.4 μL). After 16 hours, the reaction was worked up and purified by silica gel column chromatography (21% acetone in hexanes) to afford the title compound as a white foam with (48.8 mg, 66% yield, 6:1 isolated dr).

R_f = 0.2 (25% acetone in hexanes)

^{1}H NMR (600 MHz, $CDCl_3$, 50 °C) δ 7.86^* (d, J = 8.6 Hz, 1H), 7.74 – 7.68^* (m, 2H), 7.39 – 7.26^* (m, 7H), 7.24 – 7.17^* (m, 2H), 7.14^* (dd, J = 8.6, 1.7 Hz, 1H), 7.11 – 7.08* (m, 0.17H), 5.25^ (s, 1H), 5.04^* (s, 3H), 4.67 – 4.44^* (m, 2H), 3.67* (s, 0.36H), 3.62^ (s, 2.79H), 3.59* (s, 0.30H), 3.50^ (s, 2.65H), 3.34 – 3.25^ (m, 0.94H), 3.18^* (dd, J = 14.8, 5.4 Hz, 1H), 3.11^* (d, J = 11.7 Hz, 1H), 2.32^* (s, 3H), 1.44^* (s, 9H), 1.37^* (d, J = 7.1 Hz, 3H).

One diastereomer visible in ^{13}C NMR

^{13}C NMR (201 MHz, $CDCl_3$) δ 172.1, 172.0, 155.9, 155.2, 145.0, 136.5, 136.3, 135.4, 134.4, 131.0, 130.0, 128.7, 128.4, 128.3, 126.9, 125.0, 124.7, 118.6, 117.4, 113.8, 80.4, 67.2, 59.7, 53.6, 52.5, 52.1, 42.9, 28.5, 28.1, 21.7, 17.1.

HRMS (+ p APCI) calculated for $C_{37}H_{43}O_{10}N_3{}^{23}Na^{32}S$ [M+Na] 744.2561, found 744.2569.

$[\alpha]_D^{21}$ = +26.9 (c = 0.28, $CHCl_3$).

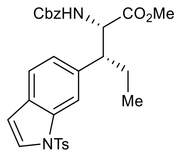

12

methyl (2*S*,3*R*)-2-(((benzyloxy)carbonyl)amino)-3-(1-tosyl-1H-indol-6-yl)pentanoate

Prepared according to GP4 using $NiCl_2$•glyme (10 mol%, 2.5 mg), (4S,4'S)-4,4'-diisopropyl-4,4',5,5'-tetrahydro-2,2'-bioxazole (10 mol%, 3.0 mg), Cs_2CO_3 (2 equiv, 74.1 mg), $Ir[(dF(CF_3)ppy)_2dtbbpy]PF_6$ (1 mol%, 1.0 mg), ArBr **4** (1.0 equiv, 37.0 mg), alkyl bromide **S28** (1.5 equiv, 54.5 mg), and 1,1,1,3,3,3-hexamethyl-2-(trimethylsilyl)trisilane (1.6 equiv, 49.4 μL). After 16 hours, the reaction was worked up and purified by silica gel column

chromatography (20% acetone in hexanes, then 5% acetone in toluene) to afford the title compound as a white foam with 19:1 dr (29.7 mg, 53% yield, 14:1 isolated dr).

R$_f$ = 0.45 (30% acetone in hexanes)

"^" indicates major diastereomer

"*" indicates minor diastereomer

^{1}H NMR (400 MHz, CDCl$_3$) δ 7.80^* (s, 1H), 7.74^* (d, J = 8.2 Hz, 2H), 7.49^* (d, J = 3.6 Hz, 1H), 7.41^* (d, J = 8.1 Hz, 1H), 7.39 – 7.28^* (m, 5H), 7.16^ (d, J = 8.0 Hz, 2.34H, includes Cbz rotamer peak), 7.13 – 7.09* (m, 0.09H), 6.97^* (dd, J = 8.1, 1.5 Hz, 1H), 6.59^* (d, J = 3.7 Hz, 1H), 5.26^ (d, J = 9.0 Hz, 0.86H), 5.13^* (s, 2H, includes rotamer), 4.96* (d, J = 10.1 Hz, 0.06H), 4.78* (dd, J = 9.4, 4.1 Hz, 0.06H), 4.63^ (dd, J = 9.1, 6.1 Hz, 0.89H), 4.52 (s, 0.12H, rotamer), 3.70* (s, 0.13H), 3.51^ (s, 2.89H), 3.30 – 3.23* (m, 0.07H), 3.06 – 2.94 (m, ^1.01H), 2.36* (s, 0.16H), 2.28^ (s, 2.98H), 2.06 – 1.80^* (m, 2H), 0.88* (t, J = 7.4 Hz, 0.24H), 0.79^ (t, J = 7.3 Hz, 3.00H).

One diastereomer visible in ^{13}C NMR

^{13}C NMR (201 MHz, CDCl$_3$) δ 171.7, 155.9, 145.0, 136.3, 136.1, 135.3, 135.3, 130.2, 130.0, 128.7, 128.4, 128.4, 126.9, 126.6, 124.2, 121.4, 113.6, 109.2, 67.3, 58.9, 52.1, 51.6, 25.0, 21.6, 12.3.

HRMS (+ p APCI) calculated for $C_{29}H_{31}O_6N_2{}^{32}S$ [M+H] 535.1897, found 535.1903.

$[\alpha]_D^{22}$ = +65.0 (c = 0.31, $CHCl_3$).

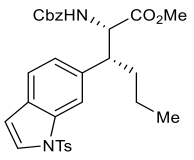

13

methyl (2*S*,3*R*)-2-(((benzyloxy)carbonyl)amino)-3-(1-tosyl-1H-indol-6-yl)hexanoate

Prepared according to GP4 on a 005 mmol scale using $NiCl_2$•glyme (10 mol%, 2.0 mg), (4S,4'S)-4,4'-diisopropyl-4,4',5,5'-tetrahydro-2,2'-bioxazole (10 mol%, 1.3 mg), Cs_2CO_3 (2 equiv), Ir[(dF(CF$_3$)ppy)$_2$dtbbpy]PF$_6$ (1 mol%, 1.3 mg), ArBr **4** (1.0 equiv, 17.8 mg), alkyl bromide **S30** (1.5 equiv, 23.9 mg), and 1,1,1,3,3,3-hexamethyl-2-(trimethylsilyl)trisilane (1.6 equiv, 24.7 μL). After 16 hours, the reaction was worked up and purified by silica gel column chromatography (15% acetone in pentane, then 4% acetone in toluene) to afford the title compound as a white foam with >20:1 dr (16.0 mg, 58% yield, 14:1 isolated dr).

R$_f$ = 0.25 (25% acetone in toluene)

"^" indicates major diastereomer

"*" indicates minor diastereomer

^{1}H NMR (600 MHz, CDCl$_3$) δ 7.80^ (s, 1.03H), 7.79* (s, 0.09H), 7.76 – 7.69^* (m, 2H), 7.49^* (d, J = 3.7 Hz, 1H), 7.42* (d, J = 8.1 Hz, 0.10H), 7.41^ (d, J = 8.0 Hz, 1.06H), 7.39 –

7.29^* (m, 5H), 7.16^ (d, J = 8.1 Hz, 1.86H), 7.12* (d, J = 8.3 Hz, 0.13H), 6.98^* (dd, J = 8.1, 1.6 Hz, 1H), 6.59^* (dd, J = 3.7, 0.8 Hz, 1H), 5.26^ (d, J = 9.1 Hz, 0.84H), 5.13^* (s, 2H), 4.98* (d, J = 9.7 Hz, 0.08H), 4.75* (dd, J = 9.3, 4.4 Hz, 0.06H), 4.61^ (dd, J = 9.1, 6.1 Hz, 0.85H), 3.71* (s, 0.15H), 3.51^ (s, 2.84H), 3.37* (dd, J = 11.9, 7.1 Hz, 0.08H), 3.11^ (q, J = 7.2 Hz, 0.98H), 2.29^* (s, 3H), 1.86^* (q, J = 7.7 Hz, 2H), 1.20 – 1.05^* (m, 2H), 0.86^* (t, J = 7.4 Hz, 3H).

One diastereomer visible in ^{13}C NMR

^{13}C NMR (151 MHz, $CDCl_3$) δ 171.8, 155.9, 145.0, 136.4, 136.3, 135.3, 135.3, 130.2, 130.0, 128.7, 128.4, 128.4, 126.9, 126.6, 124.2, 121.4, 113.5, 109.2, 77.4, 77.2, 76.9, 67.3, 59.1, 52.2, 49.5, 34.0, 21.7, 20.8, 14.1.

HRMS (+ p APCI) calculated for $C_{30}H_{33}O_6N_2{}^{32}S$ [M+H] 549.2054, found 549.2063.

$[\alpha]_D^{22}$ = +51.5 (c = 0.27, $CHCl_3$).

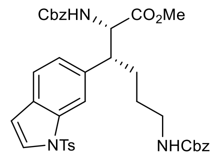

14

methyl (2*S*,3*R*)-2,6-bis(((benzyloxy)carbonyl)amino)-3-(1-tosyl-1H-indol-6-yl)hexanoate

Prepared according to GP4 using $NiCl_2$•glyme (10 mol%, 2.5 mg), (4S,4'S)-4,4'-diisopropyl-4,4',5,5'-tetrahydro-2,2'-bioxazole (10 mol%, 2.1 mg), Cs_2CO_3 (2 equiv, 66.7 mg), Ir[(dF(CF_3)ppy)$_2$dtbbpy]PF_6 (1 mol%, 1.9 mg), ArBr **4** (1.0 equiv, 37.1 mg), alkyl bromide **S32** (1.0 equiv, 67.0 mg), and 1,1,1,3,3,3-hexamethyl-2-(trimethylsilyl)trisilane (1.6 equiv, 49.4 µL). After 16 hours, the reaction was worked up and purified by silica gel column chromatography (22% acetone in pentane) to afford the title compound as a white foam with 6:1 dr (33.7 mg, 48% yield, 7:1 isolated dr).

R_f = 0.2 (25% acetone in toluene)

"^" indicates major diastereomer

"*" indicates minor diastereomer

^{1}H NMR (800 MHz, $CDCl_3$) δ 7.79^ (s, 0.90H), 7.76* (d, J = 1.4 Hz, 0.17H), 7.72^* (dd, J = 22.0, 8.1 Hz, 2H), 7.50^* (d, J = 3.7 Hz, 1H), 7.41^* (d, J = 8.1 Hz, 1H), 7.39 – 7.27^* (m, 10H), 7.18^ (d, J = 8.1 Hz, 1.51H), 7.13* (d, J = 8.2 Hz, 0.34H), 6.97^ (d, J = 7.9 Hz, 0.74H), 6.94* (d, J = 8.3 Hz, 0.33H), 6.59^* (d, J = 3.6 Hz, 1H), 5.47^ (d, J = 8.7 Hz, 0.63H), 5.13 – 5.01^* (m, 4H), 4.90* (d, J = 5.8 Hz, 0.09H), 4.78* (dd, J = 9.3, 4.0 Hz, 0.14H), 4.70^ (t, J = 6.2 Hz, 0.67H), 4.58^ (t, J = 7.8 Hz, 0.75H), 3.70* (s, 0.37H), 3.42^ (s, 2.38H), 3.21^* (dq, J = 14.5, 7.1 Hz, 1H), 3.13^* (m, 2H), 2.28^ (s, 2.72H), 2.25* (s, 0.38H), 2.01 – 1.95^* (m, 1H), 1.91 – 1.83^* (m, 1H), 1.42 – 1.34^* (m, 1H), 1.34 – 1.27^* (m, 1H).

^{13}C data includes signals from both diastereomers (7:1 dr)

^{13}C NMR (201 MHz, CDCl$_3$) δ 171.8, 171.6, 156.7, 156.6, 156.0, 145.1, 136.7, 136.3, 136.2, 135.7, 135.3, 135.3, 135.2, 130.3, 130.1, 130.0, 128.7, 128.7, 128.7, 128.6, 128.4, 128.4, 128.2, 128.2, 128.0, 126.9, 126.8, 126.8, 126.7, 123.9, 123.5, 121.8, 121.6, 113.5, 113.2, 109.2, 109.1, 67.5, 67.3, 66.8, 66.7, 59.1, 57.7, 52.5, 52.1, 49.0, 48.0, 40.9, 40.5, 29.8, 28.9, 28.9, 28.0, 27.7, 22.8, 21.6.

HRMS (+ p APCI) calculated for $C_{38}H_{40}O_8N_3{}^{32}S$ [M+H] 698.2531, found 698.2542.

$[\alpha]_D^{22}$ = +37.1 (c = 0.55, CHCl$_3$).

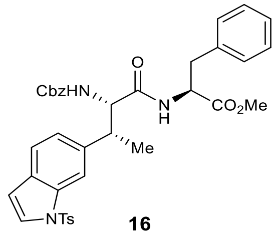

16

methyl ((2*S*,3*R*)-2-(((benzyloxy)carbonyl)amino)-3-(1-tosyl-1H-indol-6-yl)butanoyl)-*L*-phenylalaninate

Prepared according to GP4 using NiCl$_2$•glyme (20 mol%, 3.5 mg), (4S,4'S)-4,4'-diisopropyl-4,4',5,5'-tetrahydro-2,2'-bioxazole (20 mol%, 4.7 mg), Cs_2CO_3 (2 equiv, 68.2 mg), Ir[(dF(CF$_3$)ppy)$_2$dtbbpy]PF$_6$ (1 mol%, 1.1 mg), ArBr **4** (1.0 equiv, 34.2 mg), alkyl bromide **S44** (1.5 equiv, 68.9 mg), and 1,1,1,3,3,3-hexamethyl-2-(trimethylsilyl)trisilane (1.6 equiv, 49.4 µL). After 16 hours, the reaction was worked up and purified by column chromatography, then preparative TLC (25 to 40% EtOAc in hexanes, then 50% EtOAc in hexanes) to afford the title compound as a white foam with 2:1 dr (15.6 mg, 23% yield, 1.4:1 isolated dr).

R$_f$ = 0.15 (25% EtOAc in hexanes)

"^" indicates major diastereomer

"*" indicates minor diastereomer

^{1}H NMR (400 MHz, CDCl$_3$) δ 7.87* (s, 0.43H), 7.80^ (s, 0.56H), 7.78 – 7.71^* (m, 2H), 7.55* (d, J = 3.7 Hz, 0.44H), 7.50^ (d, J = 3.7 Hz, 0.58H), 7.43* (d, J = 8.0 Hz, 0.44H), 7.40^ (d, J = 8.1 Hz, 0.59H), 7.38 – 7.26^* (m, 5H), 7.23 – 7.11^* (m, 4H), 7.11 – 6.99^* (m, 2H), 6.98 – 6.90^* (m, 1H), 6.77^* (d, J = 7.4 Hz, 1H), 6.61* (dd, J = 3.7, 0.7 Hz, 0.43H), 6.58^ (dd, J = 3.6, 0.8 Hz, 0.59H), 6.11* (d, J = 7.5 Hz, 0.34H), 5.83^ (d, J = 7.5 Hz, 0.46H), 5.40^ (d, J = 9.1 Hz, 0.50H), 5.21* (d, J = 8.3 Hz, 0.37H), 5.12^ (s, 1.16H), 5.10 – 5.04* (m, 0.93H), 4.79* (q, J = 6.1 Hz, 0.44H), 4.51 – 4.43^ (m, 0.50H), 4.43 – 4.36* (m, 0.39H), 4.29^ (t, J = 8.5 Hz, 0.60H), 3.71* (s, 1.28H), 3.62 – 3.51* (m, 0.45H), 3.39^ (s, 1.53H), 3.32 – 3.20^ (m, 0.58H), 2.99^* (dd, J = 14.2, 5.8 Hz, 1.38H), 2.89^ (dd, J = 13.8, 5.9 Hz, 0.60H), 2.31^ (s, 1.85H), 2.26* (s, 1.21H), 1.37^* (d, J = 7.2 Hz, 3H).

^{13}C data includes signals from both diastereomers (1.4:1 dr)

^{13}C NMR (201 MHz, CDCl$_3$) δ 171.5, 170.9, 170.1, 169.9, 156.3, 156.2, 145.1, 145.1, 138.8, 138.0, 136.4, 136.3, 135.7, 135.5, 135.3, 135.3, 135.2, 135.1, 130.2, 130.1, 130.0, 129.9, 129.3,

129.2, 128.7, 128.7, 128.6, 128.6, 128.3, 128.3, 128.2, 128.0, 127.2, 127.2, 127.0, 127.0, 126.6, 126.5, 123.7, 123.0, 121.8, 121.6, 112.8, 112.7, 108.9, 108.8, 67.3, 67.2, 61.1, 60.5, 53.3, 53.2, 52.5, 52.2, 43.1, 41.8, 38.1, 37.8, 21.7, 21.7, 17.6, 17.3.

HRMS (+ p APCI) calculated for $C_{37}H_{38}O_7N_3{}^{32}S$ [M+H] 668.2425, found 668.2421.

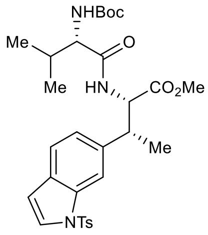

17

methyl (2*S*,3*R*)-2-((*S*)-2-((tert-butoxycarbonyl)amino)-3-methylbutanamido)-3-(1-tosyl-1H-indol-6-yl)butanoate

Prepared according to GP4 using $NiCl_2$•glyme (10 mol%, 2.4 mg), (4S,4'S)-4,4'-diisopropyl-4,4',5,5'-tetrahydro-2,2'-bioxazole (10 mol%, 2.1 mg), Cs_2CO_3 (2 equiv, 67.0 mg), Ir[(dF(CF_3)ppy)$_2$dtbbpy]PF_6 (1 mol%, 1.0 mg), ArBr **4** (1.0 equiv, 35.9 mg), alkyl bromide **24** (1.5 equiv, 63.6 mg), and 1,1,1,3,3,3-hexamethyl-2-(trimethylsilyl)trisilane (1.6 equiv, 49.4 μL). After 16 hours, the reaction was worked up and purified by silica gel column chromatography (23% acetone in pentane) to afford the title compound as a white powder with 10:1 dr (35.0 mg, 58% yield, 6:1 isolated dr).

$\mathbf{R_f}$ = 0.2 (25% acetone in hexanes)

"^" indicates major diastereomer

"*" indicates minor diastereomer

^{1}H NMR (800 MHz, $CDCl_3$) δ 7.80^* (s, 1H), 7.76^* (d, J = 8.4 Hz, 2H), 7.52* (d, J = 3.7 Hz, 0.17H), 7.50^ (d, J = 3.6 Hz, 0.80H), 7.47* (d, J = 8.0 Hz, 0.17H), 7.43^ (d, J = 8.0 Hz, 0.83H), 7.24^* (d, J = 8.2 Hz, 2H), 7.07* (d, J = 8.0 Hz, 0.16H), 7.05^ (d, J = 8.3 Hz, 0.89H), 6.61* (d, J = 3.7 Hz, 0.17H), 6.60^ (d, J = 3.6 Hz, 0.82H), 6.51^ (d, J = 8.6 Hz, 0.82H), 6.23* (d, J = 9.1 Hz, 0.12H), 5.16* (d, J = 8.9 Hz, 0.16H), 5.12^ (d, J = 8.6 Hz, 0.69H), 4.93* (dd, J = 9.3, 4.4 Hz, 0.15H), 4.82^ (dd, J = 8.6, 6.3 Hz, 0.84H), 3.99 – 3.89^* (m, 1H), 3.69* (s, 0.46H), 3.62 – 3.56* (m, 0.17H), 3.48^ (s, 2.58H), 3.32^ (p, J = 7.2 Hz, 0.84H), 2.35* (s, 0.55H), 2.34^ (s, 2.53H), 2.22 – 2.15^* (m, 1H), 1.46^ (d, J = 7.3 Hz, 2.69H), 1.41^ (s, 7.64H), 1.39* (d, J = 7.1 Hz, 0.67H), 1.37* (s, 1.49H), 0.96 – 0.82^* (m, 6H).

^{13}C data includes signals from both diastereomers (6:1)

^{13}C NMR (201 MHz, $CDCl_3$) δ 171.4, 171.4, 156.1, 145.2, 145.1, 138.0, 135.5, 135.5, 135.2, 130.1, 130.1, 130.1, 130.1, 130.0, 127.0, 126.9, 126.6, 123.5, 123.2, 121.7, 121.4, 112.6, 112.6, 109.1, 108.9, 80.1, 60.3, 60.2, 57.9, 57.1, 52.4, 52.1, 43.4, 42.4, 30.4, 30.4, 28.4, 28.3, 21.7, 21.7, 19.4, 19.3, 18.0, 17.8, 17.8, 17.4.

HRMS (+ p APCI) calculated for $C_{30}H_{40}O_7N_3{}^{32}S$ [M+H] 586.2582, found 586.2591.

$[\alpha]_D^{22}$ = +2.2 (c = 0.64, $CHCl_3$).

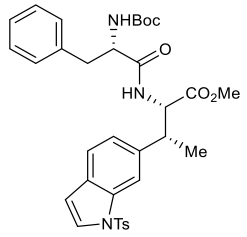

18

methyl (2*S*,3*R*)-2-((*S*)-2-((tert-butoxycarbonyl)amino)-3-phenylpropanamido)-3-(1-tosyl-1H-indol-6-yl)butanoate

Prepared according to GP4 using $NiCl_2$•glyme (10 mol%, 2.1 mg), (4S,4'S)-4,4'-diisopropyl-4,4',5,5'-tetrahydro-2,2'-bioxazole (10 mol%, 2.3 mg), Cs_2CO_3 (2 equiv, 65.0 mg), Ir[(dF(CF_3)ppy)$_2$dtbbpy]PF_6 (1 mol%, 1.0 mg), ArBr **4** (1.0 equiv, 35.7 mg), alkyl bromide **S45** (1.5 equiv, 69.5 mg), and 1,1,1,3,3,3-hexamethyl-2-(trimethylsilyl)trisilane (1.6 equiv, 49.4 µL). After 16 hours, the reaction was worked up and purified by silica gel column chromatography (20% acetone in hexanes, then 3% acetone in toluene) to afford the title compound as a white foam with 10:1 dr (35.0 mg, 54% yield, 9:1 isolated dr).

R_f = 0.25 (25% acetone in hexanes)

"^" indicates major diastereomer

"*" indicates minor diastereomer

^{1}H NMR (400 MHz, $CDCl_3$) δ 7.71 – 7.63^* (m, 3H), 7.43^* (d, J = 3.7 Hz, 1H), 7.33^* (d, J = 8.1 Hz, 1H), 7.25 – 7.10^* (m, 8H, includes chloroform peak), 6.89^* (dd, J = 8.2, 1.5 Hz, 1H), 6.52^* (dd, J = 3.6, 0.8 Hz, 1H), 6.45^ (d, J = 8.5 Hz, 0.91H), 6.16* (s, 0.03H), 5.04^* (s, 1H), 4.80* (dd, J = 8.9, 4.7 Hz, 0.12H), 4.69^ (dd, J = 8.5, 6.2 Hz, 1.02H), 4.37 – 4.20^* (m, 1H), 3.59* (s, 0.19H), 3.41^ (s, 2.94H), 3.22^ (p, J = 7.1 Hz, 1H, minor is under 3.41 singlet), 3.06^* (dd, J = 14.0, 6.4 Hz, 1H), 3.00 – 2.86^* (m, 1H), 2.26^* (s, 3H), 1.32^ (d, J = 7.2 Hz, 3H, minor is under 1.29 singlet), 1.29^ (s, 8.45H), 1.23* (s, 0.48H).

One diastereomer visible in ^{13}C NMR

^{13}C NMR (201 MHz, $CDCl_3$) δ 171.1, 171.0, 155.8, 145.1, 138.0, 136.9, 135.4, 135.1, 130.1, 130.0, 129.5, 128.8, 127.0, 127.0, 126.6, 123.4, 121.3, 112.6, 109.0, 80.3, 58.0, 56.1, 52.1, 43.0, 38.0, 28.3, 21.7, 17.5.

HRMS (+ p APCI) calculated for $C_{34}H_{40}O_7N_3{}^{32}S$ [M+H] 634.2582, found 634.2595.

$[\alpha]_D^{22}$ = +3.4 (c = 0.59, $CHCl_3$).

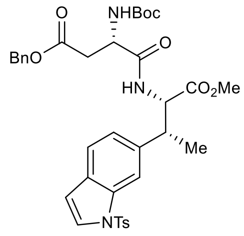

19

benzyl (*S*)-3-((tert-butoxycarbonyl)amino)-4-(((2*S*,3*R*)-1-methoxy-1-oxo-3-(1-tosyl-1H-indol-6-yl)butan-2-yl)amino)-4-oxobutanoate

Prepared according to GP4 using $NiCl_2$•glyme (10 mol%, 2.6 mg), (4S,4'S)-4,4'-diisopropyl-4,4',5,5'-tetrahydro-2,2'-bioxazole (10 mol%, 3.1 mg), Cs_2CO_3 (2 equiv, 73.3 mg), Ir[(dF(CF$_3$)ppy)$_2$dtbbpy]PF$_6$ (1 mol%, 1.1 mg), ArBr **4** (1.0 equiv, 34.5 mg), alkyl bromide **S46** (1.5 equiv, 78.4 mg), and 1,1,1,3,3,3-hexamethyl-2-(trimethylsilyl)trisilane (1.6 equiv, 49.4 μL). After 16 hours, the reaction was worked up and purified by silica gel column chromatography (15-20% acetone in pentane, then 5% acetone in toluene) to afford the title compound as a white powder with >20:1 dr (22.6 mg, 33% yield, >20:1 isolated dr).

R_f = 0.4 (25% acetone in pentane)

"^" indicates major diastereomer

"*" indicates minor diastereomer

^{1}H NMR (600 MHz, CDCl$_3$) δ 7.88^* (s, 1H), 7.82^* (d, J = 8.1 Hz, 2H), 7.51^* (d, J = 3.7 Hz, 1H), 7.44^* (dd, J = 8.1, 0.6 Hz, 1H), 7.39 – 7.29^* (m, 5H), 7.25 – 7.20^* (m, 2H), 7.10^* (d, J = 8.5 Hz, 1H), 7.07^* (dd, J = 8.1, 1.5 Hz, 1H), 6.60^* (dd, J = 3.7, 0.8 Hz, 1H), 6.06* (d, J = 9.2 Hz, 0.25H), 5.88^ (d, J = 9.0 Hz, 0.79H), 5.20 – 5.08^* (m, 2H), 4.95* (dd, J = 9.3, 3.8 Hz, 0.03H), 4.78^ (dd, J = 8.6, 6.0 Hz, 0.95H), 4.58^* (s, 1H), 3.65* (s, 0.11H), 3.48^ (s, 2.77H), 3.38 – 3.30^* (m, 1H), 3.12^* (dd, J = 16.5, 4.5 Hz, 1H), 2.76^* (dd, J = 17.3, 6.0 Hz, 1H), 2.33^* (s, 3H), 1.45^* (d, J = 7.2 Hz, 3H), 1.37^ (s, 8.89H), 1.30* (s, 0.62H).

One diastereomer visible in ^{13}C NMR

^{13}C NMR (151 MHz, CDCl$_3$) δ 172.0, 171.0, 170.6, 155.9, 145.1, 138.0, 135.5, 135.4, 135.1, 130.0, 130.0, 128.7, 128.5, 128.4, 127.1, 126.5, 123.2, 121.3, 112.9, 109.0, 80.6, 77.4, 77.2, 76.9, 67.0, 58.2, 52.1, 50.7, 43.1, 36.1, 28.3, 21.7, 17.5.

HRMS (+ p APCI) calculated for $C_{36}H_{42}O_9N_3{}^{32}S$ [M+H] 692.2636, found 692.2630.

$[\alpha]_D^{22}$ = -20.4 (c = 0.32, $CHCl_3$).

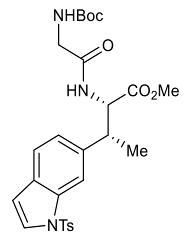

20

methyl (2*S*,3*R*)-2-(2-((tert-butoxycarbonyl)amino)acetamido)-3-(1-tosyl-1H-indol-6-yl)butanoate

Prepared according to GP4 using $NiCl_2$•glyme (10 mol%, 3.2 mg), (4S,4'S)-4,4'-diisopropyl-4,4',5,5'-tetrahydro-2,2'-bioxazole (10 mol%, 2.7 mg), Cs_2CO_3 (2 equiv, 65.0 mg), Ir[(dF(CF_3)ppy)$_2$dtbbpy]PF_6 (1 mol%, 1.1 mg), ArBr **4** (1.0 equiv, 36.6 mg), alkyl bromide **S47** (1.5 equiv54.55 mg), and 1,1,1,3,3,3-hexamethyl-2-(trimethylsilyl)trisilane (1.6 equiv, 49.4 µL). After 16 hours, the reaction was worked up and purified by silica gel column chromatography (21% acetone in pentane) to afford the title compound as a white foam with 11:1 dr (26.0 mg, 46% yield, 12:1 isolated dr).

$\mathbf{R_f}$ = 0.2 (25% acetone in pentane)

"^" indicates major diastereomer

"*" indicates minor diastereomer

^{1}H NMR (600 MHz, $CDCl_3$) δ 7.78^* (s, 1H), 7.75^* (d, J = 8.4 Hz, 2H), 7.51* (d, J = 3.7 Hz, 0.09H), 7.50^ (d, J = 3.7 Hz, 0.85H), 7.46* (d, J = 8.1 Hz, 0.10H), 7.44^ (dd, J = 8.1, 0.7 Hz, 0.91H), 7.24^* (d, J = 8.0 Hz, 2H), 7.05^* (dd, J = 8.1, 1.6 Hz, 1H), 6.70^ (d, J = 8.6 Hz, 0.83H), 6.60^* (dd, J = 3.7, 0.8 Hz, 1.00H), 6.28* (d, J = 9.0 Hz, 0.04H), 5.24^* (s, 1H), 4.92* (dd, J = 9.0, 4.7 Hz, 0.08H), 4.83^ (dd, J = 8.6, 5.9 Hz, 0.96H), 3.87 – 3.73^* (m, 3H), 3.72* (s, 0.33H), 3.53^ (s, 2.65H), 3.40 – 3.33^ (m, 0.97H), 2.34^* (s, 3H), 1.42^* (s, 12H).

One diastereomer visible in ^{13}C NMR

^{13}C NMR (151 MHz, $CDCl_3$) δ 171.6, 171.3, 169.6, 169.4, 156.3, 145.1, 137.9, 135.4, 135.1, 130.1, 130.1, 130.0, 126.9, 126.9, 126.7, 126.6, 123.4, 121.7, 121.4, 112.9, 112.7, 109.0, 108.9, 80.5, 57.7, 57.2, 52.5, 52.5, 52.2, 44.6, 42.9, 42.5, 28.4, 28.3, 21.7, 17.4.

HRMS (+ ESI) calculated for $C_{27}H_{33}O_7N_3{}^{23}Na^{32}S$ [M+Na] 566.1931, found 566.1932.

$[\alpha]_D^{21}$ = +6.8 (c = 1.26, $CHCl_3$).

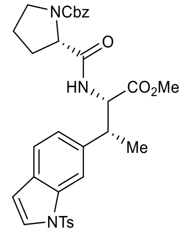

21

benzyl (*S*)-2-(((2*S*,3*R*)-1-methoxy-1-oxo-3-(1-tosyl-1H-indol-6-yl)butan-2-yl)carbamoyl)pyrrolidine-1-carboxylate

Prepared according to GP4 using $NiCl_2$•glyme (10 mol%, 3.3 mg), (4S,4'S)-4,4'-diisopropyl-4,4',5,5'-tetrahydro-2,2'-bioxazole (10 mol%, 2.2 mg), Cs_2CO_3 (2 equiv, 64.7 mg), Ir[(dF(CF_3)ppy)$_2$dtbbpy]PF_6 (1 mol%, 1.1 mg), ArBr **4** (1.0 equiv, 34.4 mg), alkyl bromide **S48** (1.5 equiv, 63.9 mg), and 1,1,1,3,3,3-hexamethyl-2-(trimethylsilyl)trisilane (1.6 equiv, 49.4 μL). After 16 hours, the reaction was worked up and purified by silica gel column chromatography (22-25% acetone in pentane, then 25% acetone in toluene) to afford the title compound as a white foam with 10:1 dr (19.6 mg, 32% yield, 13:1 isolated dr).

R_f = 0.2 (25% acetone in pentane)

"^" indicates major diastereomer

"*" indicates minor diastereomer

^{1}H NMR reported as a mixture of diastereomers and rotamers

^{1}H NMR (400 MHz, $CDCl_3$) δ 7.88 – 7.68^* (m, 3H), 7.48^* (d, J = 3.7 Hz, 1H), 7.44 – 7.27^* (m, 6H), 7.22^* (d, J = 8.2 Hz, 2H), 7.00^* (d, J = 8.0 Hz, 1H), 6.57^* (s, 1H), 5.14^* (s, 2H), 4.88* (dd, J = 8.7, 6.1 Hz, 0.07H), 4.77^ (dd, J = 8.5, 6.5 Hz, 0.93H), 4.37^* (s, 1H), 3.46^* (s, 6H), 2.32^* (s, 3H), 2.23 – 1.97^* (m, 1H), 1.96 – 1.78^* (m, 3H), 1.36^* (d, J = 7.2 Hz, 3H).

^{13}C data includes signals from both diastereomers (13:1 dr)

^{13}C NMR (151 MHz, $CDCl_3$) δ 171.9, 171.6, 171.4, 156.4, 145.0, 138.5, 136.6, 135.4, 135.2, 130.2, 130.0, 128.6, 128.6, 128.2, 128.1, 128.0, 127.9, 127.0, 126.6, 123.5, 121.1, 112.7, 109.1, 67.5, 61.1, 60.5, 58.3, 57.7, 52.4, 52.1, 47.7, 47.1, 43.0, 31.1, 29.8, 29.4, 28.0, 24.7, 23.8, 21.7, 17.5.

HRMS (+ ESI) calculated for $C_{33}H_{35}O_7N_3{}^{23}Na^{32}S$ [M+Na] 640.2088, found 640.2089.

$[\alpha]_D^{22}$ = -7.03 (c = 0.68, $CHCl_3$).

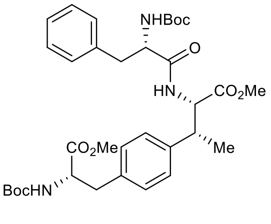

22

methyl (2*S*,3*R*)-3-(4-((*S*)-1-((tert-butoxycarbonyl)amino)-2-methoxy-2-oxoethyl)phenyl)-2-((*S*)-2-((tert-butoxycarbonyl)amino)-3-phenylpropanamido)butanoate

Prepared according to GP4 using $NiCl_2$•glyme (10 mol%, 2.1 mg), (4S,4'S)-4,4'-diisopropyl-4,4',5,5'-tetrahydro-2,2'-bioxazole (10 mol%, 2.3 mg), Cs_2CO_3 (2 equiv, 64.4 mg), Ir[(dF(CF_3)ppy)$_2$dtbbpy]PF_6 (1 mol%, 1.2 mg), ArBr **S19** (1.0 equiv, 40.7 mg), alkyl bromide **S45** (1.5 equiv, 67.0 mg), and 1,1,1,3,3,3-hexamethyl-2-(trimethylsilyl)trisilane (1.6 equiv, 49.4

μL). After 16 hours, the reaction was worked up and purified by silica gel column chromatography (20-30% acetone in hexanes, then 5% acetone in toluene) to afford the title compound as a white foam with 6:1 dr (41.2 mg, 56% yield, 7:1 isolated dr).

R_f = 0.2 (20% acetone in hexanes)

"^" indicates major diastereomer

"*" indicates minor diastereomer

^{1}H NMR (600 MHz, $CDCl_3$, 50 °C) δ 7.30 – 7.24^* (m, 3H, includes $CDCl_3$ reference), 7.24 – 7.16^* (m, 3H), 7.05 – 7.00^* (m, 4H), 6.30^ (d, J = 8.7 Hz, 0.90H), 6.07* (d, J = 8.3 Hz, 0.12H), 4.96^* (s, 2H), 4.68^ (dd, J = 8.7, 6.9 Hz, 4.68H), 4.55^* (s, 1H), 4.30^* (q, J = 7.3 Hz, 1H), 3.70^* (s, 3H), 3.64* (s, 0.35H), 3.49^ (s, 2.65H), 3.24* (p, J = 6.7 Hz, 0.11H), 3.15 – 2.89^* (m, 4.40H), 1.45 – 1.39^* (m, 18H), 1.28^* (d, J = 7.2 Hz, 3H).

^{13}C data includes signals from both diastereomers (7:1 dr)

^{13}C NMR (201 MHz, $CDCl_3$) δ 172.4, 171.3, 171.0, 155.6, 155.2, 140.0, 136.9, 136.8, 135.0, 129.4, 128.8, 127.9, 127.0, 80.4, 80.1, 57.8, 56.0, 54.5, 52.4, 52.1, 42.5, 38.1, 38.0, 38.0, 28.5, 16.7.

HRMS (+ p APCI) calculated for $C_{34}H_{48}O_9N_3$ [M+H] 642.3385, found 642.3395.

$[\alpha]_D^{21}$ = +25.9 (c = 1.05, $CHCl_3$).

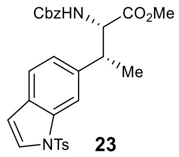

23

methyl (2*S*,3*R*)-2-(((benzyloxy)carbonyl)amino)-3-(1-tosyl-1H-indol-6-yl)butanoate

Prepared according to GP4 using $NiCl_2$•glyme (10 mol%, 2.7 mg), (4S,4'S)-4,4'-diisopropyl-4,4',5,5'-tetrahydro-2,2'-bioxazole (10 mol%, 2.7 mg), Cs_2CO_3 (2 equiv, 66.4 mg), Ir[(dF(CF_3)ppy)$_2$dtbbpy]PF_6 (1 mol%, 1.1 mg), 5-bromo-1-tosyl-1H-indole (1.0 equiv, 36.5 mg), alkyl bromide **S2** (1.5 equiv, 52.2 mg), and 1,1,1,3,3,3-hexamethyl-2-(trimethylsilyl)trisilane (1.6 equiv, 49.4 μL). After 16 hours, the reaction was worked up and purified by silica gel column chromatography (20% acetone in pentane) to afford the title compound as a white foam with 15:1 dr (39.2 mg, 72% yield, 12:1 isolated dr).

1 mmol scale:

Prepared according to GP4 in a scintillation vial using $NiCl_2$•glyme (10 mol%, 2.7 mg), (4S,4'S)-4,4'-diisopropyl-4,4',5,5'-tetrahydro-2,2'-bioxazole (10 mol%, 2.7 mg), Cs_2CO_3 (2 equiv, 66.4 mg), Ir[(dF(CF_3)ppy)$_2$dtbbpy]PF_6 (1 mol%, 1.1 mg), 5-bromo-1-tosyl-1H-indole (1.0 equiv, 36.5 mg), alkyl bromide **S2** (1.5 equiv, 52.2 mg), and 1,1,1,3,3,3-hexamethyl-2-(trimethylsilyl)trisilane (1.6 equiv, 49.4 μL). After 16 hours, the reaction was worked up and purified by silica gel column chromatography (13% acetone in pentane) to afford the title compound as a white foam with 15:1 diastereoselectivity (214 mg, 41% yield, 17:1 isolated dr).

"^" = indicates major diastereomer

"*" = indicates minor diastereomer

^{1}H NMR (600 MHz, $CDCl_3$, 50 °C) δ 7.86^ (dd, *J* = 1.5, 0.8 Hz, 0.96H), 7.83* (dd, *J* = 1.5, 0.8 Hz, 0.08H), 7.77 – 7.71^* (m, 2H), 7.50^* (d, *J* = 3.6 Hz, 1H), 7.41^* (dd, *J* = 8.1, 0.6 Hz, 1H), 7.37 – 7.26^* (m, 5H), 7.19^* (d, *J* = 8.2 Hz, 2H), 7.05^* (dd, *J* = 8.1, 1.6 Hz, 1H), 6.59^* (dd, *J* = 3.7, 0.8 Hz, 1H), 5.24^ (s, 0.65H), 5.11^* (d, *J* = 6.4 Hz, 2H), 4.97* (s, 0.10H), 4.60^* (s, 1H), 3.71* (s, 0.24H), 3.53^ (s, 2.96H), 3.39 – 3.31^* (m, 1H), 2.30^ (s, 2.93H), 2.28* (s, 0.24H), 1.45^* (d, *J* = 7.2 Hz, 3H).

^{13}C data includes signals from both diastereomers (12:1 dr)

^{13}C NMR (151 MHz, $CDCl_3$) δ 172.0, 171.8, 156.5, 155.9, 145.1, 145.0, 138.0, 137.2, 136.3, 136.3, 135.4, 135.3, 135.2, 130.1, 130.0, 130.0, 128.7, 128.3, 128.3, 126.9, 126.8, 126.7, 126.6, 123.4, 123.1, 121.6, 121.4, 112.8, 109.1, 109.0, 67.3, 67.3, 59.8, 59.5, 52.4, 52.2, 43.3, 42.4, 21.7, 18.1, 17.5.

HRMS (+ p APCI) calculated for $C_{28}H_{28}O_6N_2{}^{23}Na^{32}S$ [M+Na] 543.1560, found 543.1568.

$[\alpha]_D^{22}$ = +58.6 (c = 0.52, $CHCl_3$).

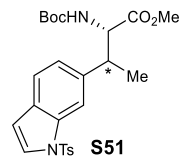

S51

methyl (2*S*,3*R*)-2-((tert-butoxycarbonyl)amino)-3-(1-tosyl-1H-indol-6-yl)butanoate

Prepared according to GP4 using $NiCl_2$•glyme (10 mol%), (4S,4'S)-4,4'-diisopropyl-4,4',5,5'-tetrahydro-2,2'-bioxazole (10 mol%), Cs_2CO_3 (2 equiv), Ir[(dF(CF_3)ppy)$_2$dtbbpy]PF_6 (1 mol%), 6-bromo-1-tosyl-1H-indole (1.0 equiv), alkyl bromide **2.14b** (1.5 equiv), and 1,1,1,3,3,3-hexamethyl-2-(trimethylsilyl)trisilane (1.6 equiv). After 16 hours, the reaction was worked up and purified by silica gel column chromatography (20% acetone in hexanes) to afford the title compound as a white foam with 12:1 dr (51% yield).

The diastereomers were further separated by preparative HPLC (Poroshell 120 C18, 1.0 ml/min, 65-75% MeCN in H_2O, t_R= 4.9 min and t_R= 5.1 min).

S51 (Diastereomer 1) *(*R*):

^{1}H NMR (400 MHz, $CDCl_3$) δ 7.84 (d, J = 1.5 Hz, 1H), 7.80 – 7.73 (m, 2H), 7.50 (d, J = 3.7 Hz, 1H), 7.43 (d, J = 8.1 Hz, 1H), 7.23 (d, J = 8.1 Hz, 2H), 7.06 (dd, J = 8.2, 1.5 Hz, 1H), 6.59 (dd, J = 3.6, 0.8 Hz, 1H), 5.08 (d, J = 9.1 Hz, 1H), 4.52 (dd, J = 9.1, 6.4 Hz, 1H), 3.50 (s, 3H), 3.31 (p, J = 7.1 Hz, 1H), 2.33 (s, 3H), 1.44 (s, 12H).

^{13}C NMR (151 MHz, $CDCl_3$) δ 172.1, 155.3, 145.0, 138.3, 135.4, 135.2, 130.0, 130.0, 127.0, 126.6, 123.5, 121.3, 112.9, 109.1, 80.2, 59.4, 52.1, 43.4, 28.4, 21.7, 17.6.

HRMS (- p APCI) calculated for $C_{25}H_{29}O_6N_2{}^{32}S$ [M-H] 485.1752, found 485.1759.

$[\alpha]_D^{22}$ = +76.2 (c = 1.01, $CHCl_3$).

S51 (Diastereomer 2) *(*S*):

^{1}H NMR (600 MHz, $CDCl_3$) δ 7.81 (s, 1H), 7.76 (d, J = 8.3 Hz, 2H), 7.50 (d, J = 3.7 Hz, 1H), 7.45 (d, J = 8.1 Hz, 1H), 7.24 (d, J = 8.1 Hz, 2H), 7.05 (dd, J = 8.1, 1.5 Hz, 1H), 6.60 (dd, J = 3.7, 0.8 Hz, 1H), 4.79 (d, J = 9.1 Hz, 1H), 4.59 (dd, J = 9.0, 4.9 Hz, 1H), 3.72 (s, 3H), 3.52 (p, J = 6.1 Hz, 1H), 2.34 (s, 3H), 1.42 (s, 12H).

^{13}C NMR (151 MHz, $CDCl_3$) δ 172.4, 155.8, 145.1, 137.6, 135.5, 135.3, 130.1, 127.0, 126.9, 126.7, 123.2, 121.5, 113.0, 109.1, 80.2, 59.0, 52.3, 42.5, 28.4, 21.7, 18.1.

HRMS (- p APCI) calculated for $C_{25}H_{29}O_6N_2{}^{32}S$ [M-H] 485.1752, found 485.1761.

$[\alpha]_D^{22}$ = +22.2 (c = 0.31, $CHCl_3$).

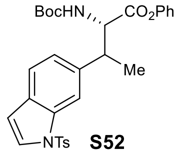

S52

phenyl (2*S*)-2-((tert-butoxycarbonyl)amino)-3-(1-tosyl-1H-indol-6-yl)butanoate

Prepared according to GP4 using $NiCl_2$•glyme (10 mol%), (4S,4'S)-4,4'-diisopropyl-4,4',5,5'-tetrahydro-2,2'-bioxazole (10 mol%), Cs_2CO_3 (2 equiv), Ir[(dF(CF_3)ppy)$_2$dtbbpy]PF_6 (1 mol%), 5-bromo-1-tosyl-1H-indole (1.0 equiv), alkyl bromide **S42** (1.0 equiv), and 1,1,1,3,3,3-hexamethyl-2-(trimethylsilyl)trisilane (1.1 equiv). After 16 hours, the reaction was worked up and purified by silica gel column chromatography (15% acetone in pentane) to afford the title compound as a white foam with 3.9:1 dr (10.4 mg, 19% yield, 3:1 isolated dr).

R_f = 0.2 (15% acetone in pentane)

"^" indicates major diastereomer

"*" indicates minor diastereomer

^{1}H NMR (800 MHz, $CDCl_3$) δ 8.02^ (s, 0.79H), 7.97* (s, 0.25H), 7.72^* (dd, *J* = 8.3, 6.4 Hz, 2H), 7.55^* (d, *J* = 3.7 Hz, 1H), 7.50^* (d, *J* = 8.0 Hz, 1H), 7.37* (t, *J* = 7.7 Hz, 0.51H), 7.23 – 7.02^* (m, 6.42H), 6.66 – 6.63^* (m, 1H), 6.52^* (d, *J* = 7.8 Hz, 1H), 5.17^ (d, *J* = 9.1 Hz, 0.67H), 4.92* (d, *J* = 9.0 Hz, 0.20H), 4.85* (dd, *J* = 9.0, 4.8 Hz, 0.28H), 4.76^ (t, *J* = 8.4 Hz, 0.65H), 3.74* (p, *J* = 6.3 Hz, 0.20H), 3.40^ (p, *J* = 7.3 Hz, 0.82H), 2.29* (s, 0.70H), 2.20^ (s, 2.47H), 1.59^* (d, *J* = 7.1 Hz, 3H), 1.49^* (s, 9H).

^{13}C data includes signals from both diastereomers (3:1 dr)

^{13}C NMR (201 MHz, $CDCl_3$) δ 170.7, 170.5, 155.5, 150.5, 150.1, 145.0, 138.1, 135.33, 135.28, 135.1, 130.2, 130.0, 129.9, 129.6, 129.3, 126.9, 126.83, 126.77, 126.2, 126.0, 123.8, 123.4, 121.6, 121.5, 121.4, 113.3, 113.11, 109.1, 80.4, 77.3, 77.2, 77.0, 59.6, 59.1, 44.1, 42.6, 29.9, 28.5, 21.7, 21.6, 18.6, 18.2.

HRMS (- APCI) calculated for $C_{27}H_{33}O_7N_3{}^{35}Cl^{32}S$ [M+Cl-] 578.1733, found 578.1734.

$[\alpha]_D^{21}$ = -1.35 (c = 0.21, $CHCl_3$).

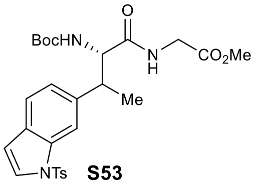

S53

methyl ((2*S*)-2-((tert-butoxycarbonyl)amino)-3-(1-tosyl-1H-indol-6-yl)butanoyl)glycinate

Prepared according to GP4 using $NiCl_2$•glyme (10 mol%, 3.3 mg), (4S,4'S)-4,4'-diisopropyl-4,4',5,5'-tetrahydro-2,2'-bioxazole (10 mol%, 4.0 mg), Cs_2CO_3 (2 equiv, 68.4 mg), Ir[(dF(CF_3)ppy)$_2$dtbbpy]PF_6 (1 mol%, 1.2 mg), 5-bromo-1-tosyl-1H-indole (1.0 equiv, 35.3 mg), alkyl bromide **S43** (1.5 equiv, 52.3 mg), and 1,1,1,3,3,3-hexamethyl-2-(trimethylsilyl)trisilane (1.6 equiv, 49.4 µL). After 16 hours, the reaction was worked up and purified by silica gel column chromatography (20% acetone in pentane, then 5-10% acetone in toluene) to afford the title compound as a white powder with 1.3:1 dr (18.9 mg, 35% yield, 1:1 isolated dr).

"^" indicates major diastereomer

"*" indicates minor diastereomer

^{1}H NMR (800 MHz, $CDCl_3$) δ 7.86^* (s, 1H), 7.83 – 7.78^* (m, 2H), 7.56^ (d, J = 3.6 Hz, 0.54H), 7.54* (d, J = 3.7 Hz, 0.50H), 7.50^ (dd, J = 8.1, 0.6 Hz, 0.74H), 7.48* (d, J = 8.1 Hz, 0.48H), 7.28 – 7.26^* (m, 2H), 7.22 – 7.15^* (m, 2H), 6.64^ (dd, J = 3.7, 0.8 Hz, 0.52H), 6.62* (dd, J = 3.7, 0.8 Hz, 0.48H), 6.23* (s, 0.35H), 5.97^ (s, 0.38H), 5.17^ (s, 0.41H), 4.97* (d, J = 7.8 Hz, 0.38H), 4.40* (s, 0.43H), 4.37 – 4.32^ (m, 0.51H), 4.05^ (dd, J = 18.3, 5.3 Hz, 0.56H), 3.98^ (dd, J = 18.3, 5.1 Hz, 0.57H), 3.88* (d, J = 18.1 Hz, 0.43H), 3.74* (s, 1.58H), 3.63^* (s, 2.33H), 3.41 – 3.35* (m, 0.43H), 2.38* (s, 1.01H), 2.37^ (s, 2.37H), 1.44 – 1.41^* (m, 12H).

^{13}C data includes signals from both diastereomers (1:1 dr)

^{13}C NMR (201 MHz, $CDCl_3$) δ 171.1, 171.1, 170.0, 169.6, 155.7, 145.2, 145.1, 139.2, 138.0, 135.4, 135.4, 135.2, 135.1, 130.1, 130.1, 129.9, 129.9, 129.2, 128.4, 127.1, 126.6, 126.5, 125.4, 123.5, 123.1, 121.7, 121.6, 112.8, 108.9, 108.8, 80.4, 60.5, 60.2, 52.5, 52.4, 42.9, 41.7, 41.3, 41.1, 29.9, 28.4, 21.7, 21.7, 21.6, 17.5, 17.3.

HRMS (- ESI) calculated for $C_{27}H_{33}O_7N_3{}^{35}Cl^{32}S$ [M+Cl$^-$] 578.1733, found 578.1738.

$[\alpha]_D^{22}$ = +29.5 (c = 0.37, $CHCl_3$).

4. Preparation of Cyclized Peptide

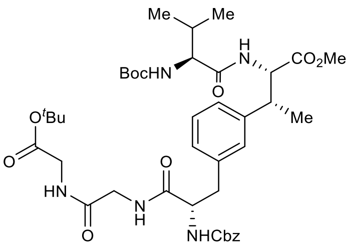

26

tert-butyl (*S*)-5-(3-((2*R*,3*S*)-3-((*S*)-2-((tert-butoxycarbonyl)amino)-3-methylbutanamido)-4-methoxy-4-oxobutan-2-yl)benzyl)-3,6,9-trioxo-1-phenyl-2-oxa-4,7,10-triazadodecan-12-oate

Prepared according to GP4 using $NiCl_2$•glyme (10 mol%, 2.8), (4S,4'S)-4,4'-diisopropyl-4,4',5,5'-tetrahydro-2,2'-bioxazole (10 mol%, 2.8 mg), Cs_2CO_3 (2 equiv, 65.2 mg), Ir[(dF(CF_3)ppy)$_2$dtbbpy]PF_6 (1 mol%, 2.0 mg), aryl bromide **25** (1.0 equiv, 54.6 mg), alkyl bromide **S38** (1.5 equiv, 60.0 mg), and 1,1,1,3,3,3-hexamethyl-2-(trimethylsilyl)trisilane (1.6 equiv, 49.4 µL). After 16 hours, the reaction was worked up and purified by silica gel column chromatography (30% acetone in pentane), then preparative HPLC(Pursuit 5 C18 250x21.2mm, 50-95% MeCN in H_2O, 10 min gradient, t_R = 8.830 min) to afford the title compound as a white powder with 15:1 isolated dr (14.4 mg, 18% yield).

Rotamers extreme in $CDCl_3$, mostly resolved in MeOD, though still visible at 25 °C

“^” indicates major diastereomer

“*” indicates minor diastereomer

^{1}H NMR (800 MHz, MeOD, 50 °C) δ 7.33 – 7.25^* (m, 5H), 7.19^* (t, J = 7.6 Hz, 1H), 7.16^* (s, 1H), 7.09^* (d, J = 7.7 Hz, 1H), 7.07^* (d, J = 7.6 Hz, 1H), 5.05^* (s, 2H), 4.68^* (d, J = 7.8 Hz, 1H), 4.36^* (s, 1H), 3.94^* (d, J = 17.0 Hz, 1H), 3.90 – 3.75^* (m, 4H), 3.68* (s, 0.18H), 3.45^ (s, 2.82H), 3.19^* (p, J = 7.2 Hz, 1H), 3.15^* (dd, J = 14.2, 5.7 Hz, 1H), 2.93^* (s, 1H), 2.01 – 1.92^* (m, 1H), 1.46^* (s, 9H), 1.45^* (s, 9H), 1.32^* (d, J = 5.8 Hz, 3H), 0.95 – 0.84^* (m, 6H).

One diastereomer observed in ^{13}C NMR

^{13}C NMR (201 MHz, MeOD) δ 174.6, 174.6, 172.9, 171.9, 170.4, 158.5, 157.9, 143.4, 138.6, 138.1, 133.4, 130.0, 129.6, 129.5, 129.0, 128.8, 127.1, 83.0, 80.6, 67.8, 61.6, 59.5, 58.3, 52.4, 43.5, 43.3, 42.7, 38.5, 32.0, 28.8, 28.3, 19.7, 18.7, 17.6.

HRMS (+ ESI) calculated for $C_{40}H_{58}O_{11}N_5$ [M+H] 784.4127, found 784.4128.

$[\alpha]_D^{22}$ = +16.5 (c = 0.17, $CHCl_3$).

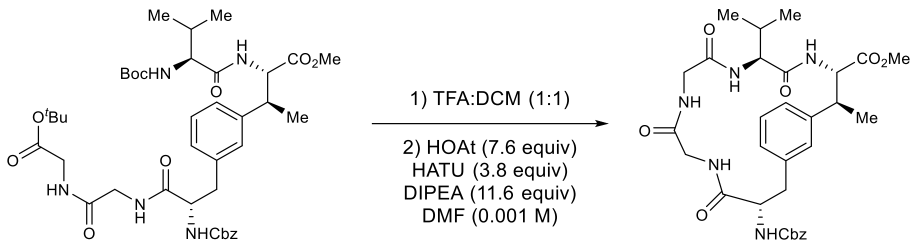

methyl (2*S*,3*S*,6*S*,15*S*)-15-(((benzyloxy)carbonyl)amino)-6-isopropyl-2-methyl-5,8,11,14-tetraoxo-4,7,10,13-tetraaza-1(1,3)-benzenacyclohexadecaphane-3-carboxylate

Following a reported modified procedure,[22] **26** (1.0 equiv, 7.1 mg, 0.00906 mmol) was taken up in DCM. TFA was added slowly while stirring (DCM:TFA 1:1, 0.03 M, 0.28 mL). This was stirred for 1-2 hours, then concentrated to yield the crude amino acid derivative, which was used immediately in the next reaction.

Following a modified procedure,[32] an oven-dried round bottom flask with stir bar was charged with the N-unprotected amino acid and evacuated and backfilled 3x. To this was added anhydrous DMF (0.001 M, 9 mL). To this stirred suspension was added freshly distilled DIPEA (11.6 equiv, 18 μL, 0.105 mmol) at room temperature. Upon dissolution, the solution was cooled to 0 °C in an ice bath, and and HOAt (7.6 equiv, 9.1 mg, 0.069 mmol) and HATU (3.8 equiv, 13.5 mg, 0.0344 mmol) were added. This mixture was warmed to room temperature and stirred for 64 h. Upon observed completion by TLC, the mixture was diluted with DCM and washed with 1 N HCl, then saturated sodium bicarbonate. The organic layer was dried over sodium sulfate, filtered, and concentrated *in vacuo* to provide a crude material. This was purified by preparative HPLC (Pursuit 5 C18 250x21.2mm, 85-89% MeOH in H_2O, 10 min gradient, t_R = 4.301 min) to afford the corresponding peptide as a white amorphous solid (62% yield, 3.4 mg).

^{1}H NMR (800 MHz, $CDCl_3$) δ 7.40 – 7.32 (m, 5H), 7.24 (t, J = 7.6 Hz, 1H), 7.20 (s, 1H), 7.09 (d, J = 7.5 Hz, 1H), 7.04 (d, J = 7.6 Hz, 1H), 6.38 – 6.33 (m, 2H), 6.31 (s, 1H), 5.93 (s, 1H), 5.70 – 5.66 (m, 1H), 5.15 (d, J = 3.0 Hz, 2H), 4.92 (dd, J = 9.2, 3.5 Hz, 1H), 4.41 (ddd, J = 37.2, 16.2, 9.1 Hz, 3H), 4.09 (dd, J = 9.5, 6.7 Hz, 1H), 3.81 (s, 3H), 3.57 (dd, J = 16.9, 3.3 Hz, 1H), 3.51 (dt, J = 9.0, 5.5 Hz, 1H), 3.35 (dd, J = 15.6, 4.0 Hz, 1H), 3.27 (dd, J = 13.3, 3.6 Hz, 1H), 2.85 (t, J = 12.2 Hz, 1H), 2.03 – 1.95 (m, 1H), 1.27 (d, *J* = 7.3 Hz, 3H), 0.92 (d, J = 6.7 Hz, 3H), 0.89 (d, J = 6.9 Hz, 3H).

^{13}C NMR (201 MHz, $CDCl_3$) δ 171.3, 171.0, 170.5, 170.2, 155.8, 142.6, 136.9, 136.2, 129.8, 128.8, 128.6, 128.5, 128.3, 127.4, 127.3, 67.3, 59.4, 57.3, 57.3, 52.9, 44.1, 43.6, 41.4, 39.7, 30.0, 29.9, 19.5, 18.2.

HRMS (+ ESI) calculated for $C_{31}H_{39}O_8N_5{}^{23}Na$ [M+Na] 632.2691, found 632.2691.

$[\alpha]_D^{22}$ = -3.4 (c = 0.23, MeOH).

5. Crystal Structure Data

Crystal Structure Data was obtained of the two isolated diastereomers of **S52**. Synthetic manipulations, described below, were carried out to provide evidence for the stereochemical assignment of the structures presented herein by analogy.

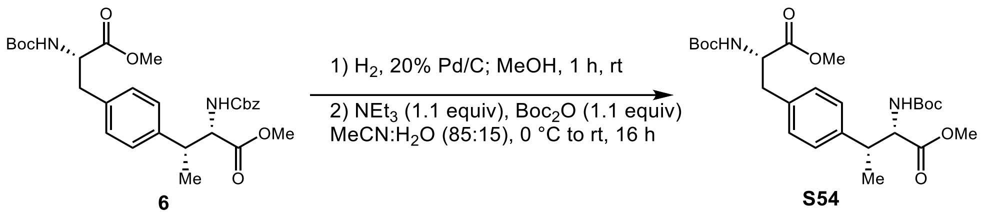

6
66% yield, 1:6 dr

S54
41% yield over 2 steps[a]

A 50-mL round bottom flask was evacuated and backfilled twice with N_2 and H_2 and charged with 20% Pd/C (.07 mmol, 7.4 mg, 0.2 equiv). Anhydrous MeOH (3 mL) and **6** (0.36 mmol, 187.7 mg, 1 equiv) were added and stirred overnight at 60° C until starting material consumption was observed by TLC. The reaction was filtered twice on celite and concentrated to yield a yellow oil. The reaction was purified by silica gel column chromatography (35% acetone in hexanes) to afford a colorless oil (102.5 mg, 74% yield), which was used in the next reaction step. $\mathbf{R_f}$ = 0.20 in 35% acetone in hexanes.

To a 25-mL round bottom flask was added a stir bar and the colorless oil from the last step (.235 mmol, 92.5 mg, 1 equiv) in H_2O (0.3 mL) and acetonitrile (1.5 mL). The reaction was cooled to 0° C in an ice bath before the addition of $(Boc)_2O$ (0.259 mmol, 56.53 mg, 1.1 equiv) and triethylamine (0.517 mmol, 0.07 mL, 2.2 equiv). This reaction was left to stir overnight before being acidified to a pH of 4 using half-saturated citric acid at 0° C. The organic layer was extracted with DCM and washed with brine before being dried over sodium sulfate. The reaction was purified by silica gel column chromatography (20% EtOAc in hexanes) to afford **S54** as a colorless solid (64.6 mg, 56% yield). 41% yield over 2 steps.

$\mathbf{R_f}$ = 0.18 in 20% EtOAc in hexanes.

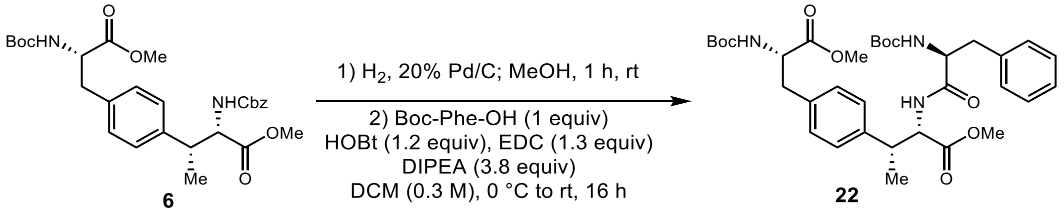

6
66% yield, 1:6 dr

22
28% yield over 2 steps[a]

A 50-mL round bottom flask was evacuated and backfilled twice with N_2 and H_2 and charged with 20% Pd/C (0.056 mmol, 5.95 mg, 0.2 equiv). Anhydrous MeOH (2.5 mL) and **6** (0.28 mmol, 148 mg, 1 equiv) were added and stirred overnight at 60° C until starting material consumption was observed by TLC. The reaction was filtered twice on celite and concentrated to yield a yellow oil. The reaction was purified by silica gel column chromatography (35% acetone in hexanes) to afford a colorless oil (59.1 mg, 54% yield), which was used in the next reaction step. $\mathbf{R_f}$ = 0.25 in 35% acetone in hexanes.

To a 100-mL round bottom flask under a N_2 atmosphere was added a stir bar and Boc-L-Phe-OH **(**0.15 mmol, 39.75 mg, 1 equiv) in DCM (0.6 mL). DIPEA (0.57 mmol, 0.1 mL, 3.8 equiv) was then added dropwise to the stirring suspension. The round bottom was cooled to 0° C in an ice bath before successive addition of the colorless oil from the previous step (0.15 mmol, 59.1 mg, 1 equiv) and HOBt (0.18 mmol, 27.5 mg, 1.2 equiv). This was stirred for 15 minutes, followed by addition of EDC (0.195 mmol, 37.3 mg, 1.3 equiv) in one portion. The reaction mixture was allowed to return to room temperature, stirred for 12 h, and concentrated before being taken up in EtOAc and washed with cold 0.1N HCl and a saturated sodium bicarb solution. The organic layer was dried with sodium sulfate and concentrated. The reaction was purified by silica gel column chromatography (25% EtOAc in hexanes) to afford **22** as a colorless solid (48 mg, 51% yield). 28% yield over 2 steps.

$\mathbf{R_f}$ = 0.20 in 25% EtOAc in hexanes.

See characterization data above.

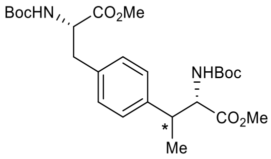

S54-D1 (*S)
S54-D2 (*R)

methyl (2*S*)-2-((tert-butoxycarbonyl)amino)-3-(4-((*S*)-2-((tert-butoxycarbonyl)amino)-3-methoxy-3-oxopropyl)phenyl)butanoate

Prepared according to GP4 using $NiCl_2$•glyme (5 mol%), dtbbpy (5 mol%), K_2CO_3 (2 equiv), Ir[(dF(CF_3)ppy)$_2$dtbbpy]PF_6 (1 mol%), **S5** (1.5 equiv), alkyl bromide **S1** (1.0 equiv), and 1,1,1,3,3,3-hexamethyl-2-(trimethylsilyl)trisilane (1.1 equiv). After 16 hours, the reaction was worked up and purified by silica gel column chromatography (25% EtOAc in hexanes) to afford the title compound as a white foam with 1.7:1 dr (40% yield).

The diastereomers were further separated by preparative HPLC (OD-H column, 5% IPA/hexanes, 1.0 ml/min, t_R=19.8 min (major) and t_R=15.1 min (minor)).

S54-D1 Diastereomer 1 *(*S*):

^{1}H NMR (400 MHz, $CDCl_3$) δ 7.13 – 7.02 (m, 4H), 4.95 (d, J = 8.2 Hz, 1H), 4.77 (d, J = 9.0 Hz, 1H), 4.57 (t, J = 6.8 Hz, 1H), 4.50 (dd, J = 9.0, 5.6 Hz, 1H), 3.70 (s, 3H), 3.68 (s, 3H), 3.32 (p, J = 6.7 Hz, 1H), 3.12 – 2.96 (m, 2H), 1.41 (d, J = 4.6 Hz, 18H), 1.33 (d, J = 7.1 Hz, 3H).

^{13}C NMR (201 MHz, $CDCl_3$) δ 172.5, 172.4, 155.7, 155.2, 139.8, 135.1, 129.6, 127.9, 80.1, 80.1, 58.9, 54.5, 52.3, 52.1, 41.9, 38.1, 28.5, 28.4, 17.6.

mp = 134.6 °C

HRMS (- p APCI) calculated for $C_{25}H_{37}O_8N_2$ [M-H] 493.2555, found 493.2558.

$[\alpha]_D^{22}$ = +31.4 (c = 0.14, $CHCl_3$).

S54-D2 Diastereomer 2 *(*R*):

^{1}H NMR (400 MHz, $CDCl_3$) δ 7.11 (d, J = 7.8 Hz, 2H), 7.05 (d, J = 7.8 Hz, 2H), 5.04 (d, J = 9.3 Hz, 1H), 4.93 (d, J = 8.3 Hz, 1H), 4.56 (dt, J = 7.4, 6.4 Hz, 1H), 4.44 (dd, J = 9.1, 6.8 Hz, 1H), 3.71 (s, 3H), 3.54 (s, 3H), 3.18 – 2.96 (m, 3H), 1.41 (d, J = 3.0 Hz, 17H), 1.33 (d, J = 7.2 Hz, 3H).

^{13}C NMR (151 MHz, $CDCl_3$) δ 172.5, 172.4, 155.3, 155.2, 140.4, 134.9, 129.5, 128.0, 80.1, 80.1, 59.2, 54.5, 52.3, 52.1, 42.8, 38.1, 28.4, 28.4, 16.8.

HRMS (- p APCI) calculated for $C_{25}H_{37}O_8N_2$ [M-H] 493.2555, found 493.2559.

$[\alpha]_D^{22}$ = +31.8 (c = 0.15, $CHCl_3$).

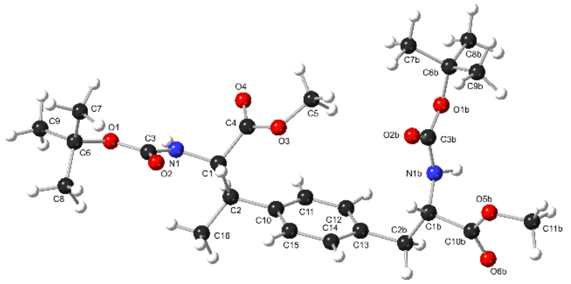

Experimental. Single colorless irregular-shaped crystals of **S54-D2** recrystallised from chloroform by slow evaporation. A suitable crystal with dimensions 0.15 × 0.13 × 0.10 mm^3 was selected and mounted on a loop with paratone on a XtaLAB Synergy, Dualflex, HyPix diffractometer. The crystal was kept at a steady T = 173.00(10) K during data collection. The structure was solved with the ShelXT 2018/2 (Sheldrick, 2015) solution program using iterative methods and by using Olex2 1.5-alpha (Dolomanov et al., 2009) as the graphical interface. The model was refined with ShelXL 2018/3 (Sheldrick, 2015) using full matrix least squares minimisation on $\boldsymbol{F}^2$.

Crystal Data. $C_{25}H_{38}N_2O_8$, M_r = 494.57, orthorhombic, $P2_12_12_1$ (No. 19), a = 9.9469(4) Å, b = 10.3473(5) Å, c = 27.8568(14) Å, $a = b = g = 90^{\circ}$, V = 2867.1(2) Å^3, T = 173.00(10) K, Z = 4, Z' = 1, m(CuK$_a$) = 0.705, 16318 reflections measured, 5198 unique (R_{int} = 0.0450) which were used in all calculations. The final wR_2 was 0.2703 (all data) and R_1 was 0.0872 (I≥2 s(I)).

**Compound**	**S54-D2**
Formula	$C_{25}H_{38}N_2O_8$
$D_{calc.}$/ g cm^{-3}	1.146
m/mm^{-1}	0.705
Formula Weight	494.57
Color	colorless
Shape	irregular-shaped
Size/mm^3	0.15×0.13×0.10
T/K	173.00(10)
Crystal System	orthorhombic
Flack Parameter	-0.06(15)
Hooft Parameter	0.01(13)
Space Group	$P2_12_12_1$
a/Å	9.9469(4)
b/Å	10.3473(5)
c/Å	27.8568(14)
$a/^{\circ}$	90
$b/^{\circ}$	90
$g/^{\circ}$	90
V/Å^3	2867.1(2)
Z	4
Z'	1
Wavelength/Å	1.54184
Radiation type	CuK$_a$
$Q_{min}/^{\circ}$	3.173
$Q_{max}/^{\circ}$	68.239
Measured Refl's.	16318
Indep't Refl's	5198
Refl's I≥2 s(I)	4346
R_{int}	0.0450
Parameters	350
Restraints	740
Largest Peak	0.607
Deepest Hole	-0.595
GooF	1.086
wR_2 (all data)	0.2703
wR_2	0.2577
R_1 (all data)	0.0973
R_1	0.0872

Structure Quality Indicators

**Reflections:**	d min (Cu\a) 2Θ=136.5° 0.83	I/σ(I) 21.7	Rint 4.50%	Full 135.4° 99.4
**Refinement:**	Shift 0.000	Max Peak 0.6	Min Peak -0.6	GooF 1.086	Hooft .01(13)

A [colour ?] irregular-shaped-shaped crystal with dimensions 0.15 × 0.13 × 0.10 mm^3 was mounted on a loop with paratone. Data were collected using a XtaLAB Synergy, Dualflex, HyPix diffractometer equipped with an Oxford Cryosystems low-temperature device operating at T = 173.00(10) K.

Data were measured using w scans with CuK$_a$ radiation. The diffraction pattern was indexed and the total number of runs and images was based on the strategy calculation from the program CrysAlisPro 1.171.42.58a (Rigaku OD, 2022). The maximum resolution that was achieved was Q = 68.239° (0.83 Å).

The unit cell was refined using CrysAlisPro 1.171.42.58a (Rigaku OD, 2022) on 6744 reflections, 41% of the observed reflections.

Data reduction, scaling and absorption corrections were performed using CrysAlisPro 1.171.42.58a (Rigaku OD, 2022). The final completeness is 99.40 % out to 68.239° in Q. A numerical absorption correction based on gaussian integration over a multifaceted crystal model was performed using CrysAlisPro 1.171.42.58a (Rigaku Oxford Diffraction, 2022). An empirical absorption correction using spherical harmonics, implemented in SCALE3 ABSPACK scaling algorithm was also performed. The absorption coefficient m of this material is 0.705 mm^{-1} at this wavelength (l = 1.54184Å) and the minimum and maximum transmissions are 0.831 and 1.000.

The structure was solved and the space group $P2_12_12_1$ (# 19) determined by the ShelXT 2018/2 (Sheldrick, 2015) structure solution program using using iterative methods and refined by full matrix least squares minimisation on $\boldsymbol{F}^2$ using version 2018/3 of ShelXL 2018/3 (Sheldrick, 2015). All non-hydrogen atoms were refined anisotropically. Hydrogen atom positions were calculated geometrically and refined using the riding model. Most hydrogen atom positions were calculated geometrically and refined using the riding model, but some hydrogen atoms were refined freely.

There is a single formula unit in the asymmetric unit, which is represented by the reported sum formula. In other words: Z is 4 and Z' is 1. The moiety formula is C25 H38 N2 O8.

The Flack parameter was refined to -0.06(15). Determination of absolute structure using Bayesian statistics on Bijvoet differences using the Olex2 results in 0.01(13). The chiral atoms in this structure are: C1(S), C1B(S), C2(R). Note: The Flack parameter is used to determine chirality of the crystal studied, the value should be near 0, a value of 1 means that the stereochemistry is wrong and the model should be inverted. A value of 0.5 means that the crystal consists of a racemic mixture of the two enantiomers.

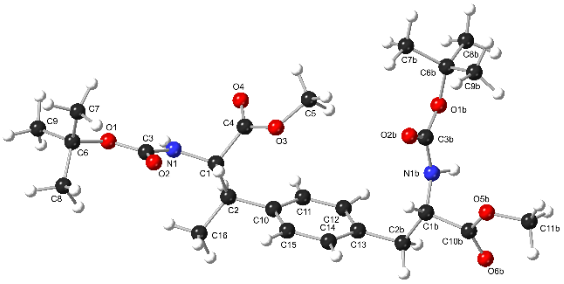

Figure S5.2.1: The ellipses are shown at the 50% probability level.

Data Plots: Diffraction Data

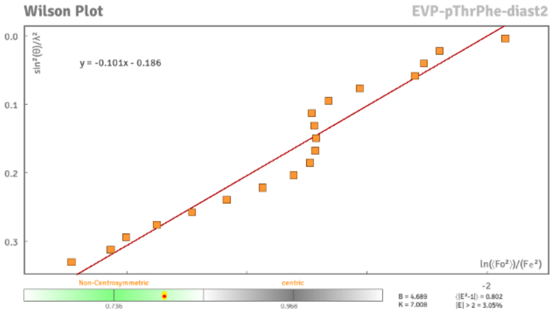

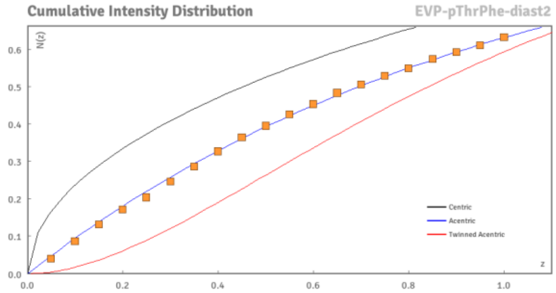

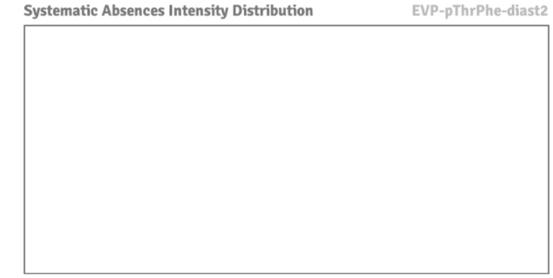

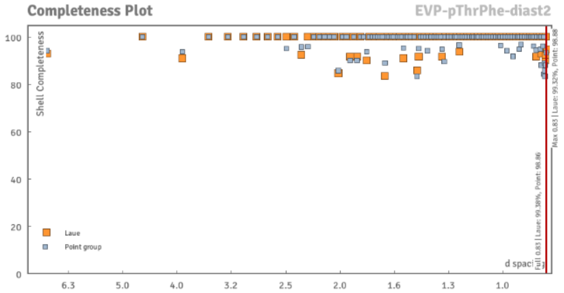

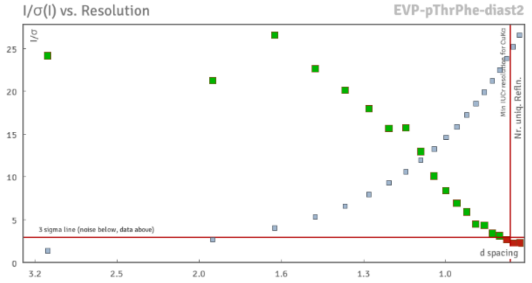

Data Plots: Refinement and Data

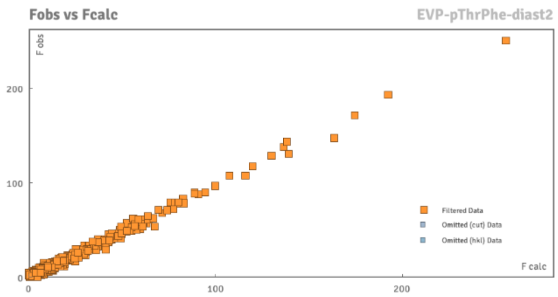

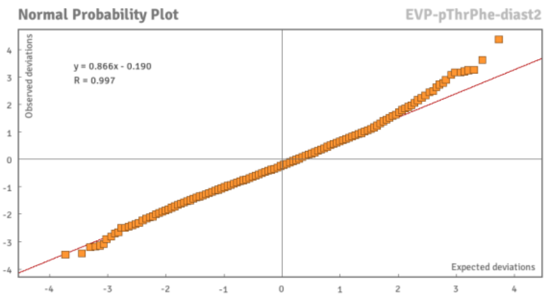

Reflection Statistics

Total reflections (after filtering)	16372	Unique reflections	5198
Completeness	0.989	Mean I/*s*	12.45
hkl_{max} collected	(12, 11, 34)	hkl_{min} collected	(-11, -12, -32)
hkl_{max} used	(11, 12, 33)	hkl_{min} used	(-11, 0, 0)
Lim d_{max} collected	20.0	Lim d_{min} collected	0.83
d_{max} used	13.93	d_{min} used	0.83
Friedel pairs	1942	Friedel pairs merged	0
Inconsistent equivalents	2	R_{int}	0.045
R_{sigma}	0.0462	Intensity transformed	0
Omitted reflections	0	Omitted by user (OMIT hkl)	54
Multiplicity	(5849, 3209, 998, 279, 68, 8)	Maximum multiplicity	10
Removed systematic absences	0	Filtered off (Shel/OMIT)	393

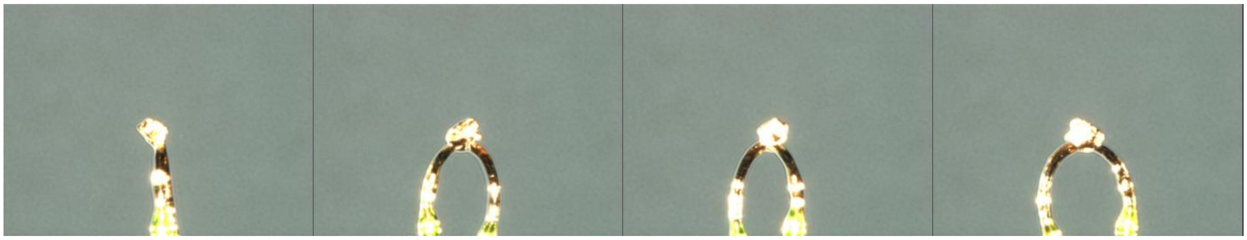

Table S5.2.1: Fractional Atomic Coordinates (×10^4) and Equivalent Isotropic Displacement Parameters (Å^2×10^3) for S54-D2. U_{eq} is defined as 1/3 of the trace of the orthogonalised U_{ij}.

Atom	x	y	z	U_{eq}
O1	5005(5)	950(5)	7352.6(15)	69.8(9)
O1B	3448(5)	5884(5)	4012(2)	74.5(10)
O2	6604(5)	1356(7)	6798.4(17)	82.0(15)
O2B	5518(5)	5153(4)	4214.1(18)	68.7(11)
O3	4845(4)	3460(4)	5645.7(15)	60.1(9)
O4	3014(4)	3326(5)	6117.4(14)	61.2(9)

Atom	x	y	z	U_{eq}
O5B	6201(7)	4068(6)	3049(2)	95.4(18)
O6B	5038(8)	2367(6)	2808.0(19)	98.4(19)
N1	4400(5)	1368(7)	6613.1(19)	69.2(11)
N1B	4051(5)	3945(4)	3773.2(18)	51.1(8)
C1	4581(6)	1575(7)	6102(2)	60.7(8)
C1B	5007(6)	2953(5)	3662(2)	51.4(8)
C2	3868(6)	545(7)	5801(2)	59.7(9)
C2B	4452(6)	1593(5)	3752(2)	50.8(8)
C3	5446(6)	1233(7)	6908(2)	60.8(12)
C3B	4439(6)	5010(5)	4015(2)	53.9(10)
C4	4054(6)	2896(7)	5965(2)	55.6(9)
C5	4348(8)	4630(8)	5429(3)	71.7(16)
C6	5941(7)	705(6)	7749(2)	78.8(11)
C6B	3774(7)	7236(7)	4134(2)	86.9(13)
C7	6758(10)	1906(9)	7845(3)	102.2(19)
C7B	4004(12)	7374(8)	4666(3)	96.2(19)
C8	6758(9)	-485(8)	7639(3)	88.6(16)
C8B	2498(10)	7940(8)	4004(4)	103.1(18)
C9	4991(9)	402(12)	8155(3)	99.4(18)
C9B	4906(10)	7735(8)	3826(4)	97.4(18)
C10	4021(6)	807(5)	5267(2)	51.1(9)
C10B	5420(6)	3080(6)	3128(2)	57.0(10)
C11	2940(6)	1229(6)	4996(2)	53.0(10)
C11B	6419(10)	4393(9)	2550(3)	90(2)
C12	3080(6)	1489(6)	4513(2)	52.0(10)
C13	4295(5)	1342(5)	4282(2)	46.1(8)
C14	5392(6)	931(5)	4558(2)	50.2(10)
C15	5265(6)	684(6)	5046(2)	52.0(10)
C16	4364(11)	-807(8)	5938(3)	87.2(18)

Table S5.2.2: Anisotropic Displacement Parameters (×10^4^) for S54-D2. The anisotropic displacement factor exponent takes the form: $-2p^2[h^2a^{*2} \times U_{11} + ... + 2hka^* \times b^* \times U_{12}]$

Atom	U_{11}	U_{22}	U_{33}	U_{23}	U_{13}	U_{12}
O1	66.0(14)	92(2)	51.4(11)	7.9(13)	3.5(10)	13.1(14)
O1B	76.4(15)	53.7(12)	93(2)	-9.7(13)	-12.6(14)	13.8(11)
O2	57.0(13)	140(5)	49(2)	5(2)	0.4(12)	6.7(18)
O2B	68.5(15)	56(2)	82(3)	-7.7(19)	-18.7(16)	-0.4(14)
O3	53.7(16)	72.6(19)	53.9(18)	2.4(15)	2.4(13)	-0.3(13)
O4	52.5(15)	83(2)	48.4(19)	0.9(17)	-0.4(13)	3.5(15)
O5B	128(4)	94(2)	64(2)	13.9(16)	3.9(18)	-42(3)
O6B	128(4)	103(3)	63.7(18)	-12.0(19)	14(2)	-38(3)
N1	53.1(15)	105(3)	49.4(11)	13.2(13)	7.5(11)	13.4(19)
N1B	52.8(16)	43.6(11)	56.9(18)	2.0(12)	-5.2(12)	1.8(10)
C1	53.5(19)	80.3(15)	48.4(11)	6.7(11)	6.8(11)	3.5(12)
C1B	54.3(19)	44.3(11)	55.7(15)	5.4(10)	-3.0(13)	3.4(11)

Atom	U_{11}	U_{22}	U_{33}	U_{23}	U_{13}	U_{12}
C2	54(2)	68.2(17)	56.8(12)	13.5(12)	7.2(10)	-0.3(15)
C2B	58(2)	43.3(11)	51.0(14)	-1.6(12)	0.4(12)	2.0(14)
C3	57.3(13)	73(3)	51.6(11)	4.1(15)	3.1(9)	14.8(16)
C3B	59.9(14)	44.1(11)	57.7(19)	1.6(12)	-5.5(13)	-0.1(10)
C4	48.1(15)	74.6(16)	44.1(18)	-1.4(13)	-3.9(13)	-2.3(12)
C5	67(3)	79(3)	70(4)	12(2)	3(3)	4(2)
C6	79.0(18)	102(2)	55.6(13)	6.3(16)	-4.9(12)	16.2(15)
C6B	105(3)	49.2(12)	106(3)	-6.9(16)	-8(2)	12.1(14)
C7	107(4)	120(3)	80(4)	-12(3)	-19(3)	2(3)
C7B	126(5)	54(3)	109(3)	-16(2)	-12(3)	-2(4)
C8	88(3)	110(3)	68(3)	12(3)	-5(3)	28(2)
C8B	115(3)	59(3)	135(5)	-6(3)	-10(3)	25(3)
C9	94(3)	147(5)	57(2)	23(3)	4(2)	31(3)
C9B	114(3)	58(3)	120(4)	-5(3)	-3(4)	1(2)
C10	47.1(14)	51(2)	54.7(11)	5.1(13)	4.6(9)	-2.2(14)
C10B	61(2)	53.8(17)	56.5(15)	8.3(11)	0.4(13)	8.1(14)
C11	49.6(15)	54(3)	55.5(13)	0.9(16)	3.1(11)	2.8(17)
C11B	104(4)	98(4)	68(2)	22(2)	7(2)	-16(3)
C12	49.8(15)	51(3)	55.4(13)	0.5(16)	2.0(11)	2.9(15)
C13	49.4(14)	37.5(19)	51.5(13)	-2.4(12)	0.5(10)	0.4(13)
C14	47.8(15)	51(3)	51.8(14)	4.3(15)	3.1(11)	0.6(16)
C15	46.8(14)	57(3)	52.3(14)	5.8(16)	3.0(11)	-1.4(15)
C16	116(5)	73.8(17)	72(3)	20.2(19)	7(3)	16(2)

Table S5.2.1: Bond Lengths in Å for S54-D2.

Atom	Atom	Length/Å
O1	C3	1.346(8)
O1	C6	1.466(8)
O1B	C3B	1.338(8)
O1B	C6B	1.476(9)
O2	C3	1.199(8)
O2B	C3B	1.218(8)
O3	C4	1.323(7)
O3	C5	1.440(9)
O4	C4	1.204(7)
O5B	C10B	1.303(8)
O5B	C11B	1.447(10)
O6B	C10B	1.218(8)
N1	C1	1.452(8)
N1	C3	1.333(8)
N1B	C1B	1.433(7)
N1B	C3B	1.348(8)
C1	C2	1.530(10)
C1	C4	1.512(9)

Atom	Atom	Length/Å
C1B	C2B	1.533(8)
C1B	C10B	1.547(8)
C2	C10	1.519(8)
C2	C16	1.532(10)
C2B	C13	1.507(8)
C6	C7	1.508(5)
C6	C8	1.507(5)
C6	C9	1.507(5)
C6B	C7B	1.506(5)
C6B	C8B	1.507(5)
C6B	C9B	1.507(5)
C10	C11	1.385(8)
C10	C15	1.389(8)
C11	C12	1.377(8)
C12	C13	1.378(8)
C13	C14	1.401(8)
C14	C15	1.388(8)

Table S5.2.2: Bond Angles in ° for S54-D2.

Atom	Atom	Atom	Angle/°
C3	O1	C6	121.5(5)
C3B	O1B	C6B	118.5(5)
C4	O3	C5	116.7(5)
C10B	O5B	C11B	115.7(6)
C3	N1	C1	121.5(5)
C3B	N1B	C1B	120.3(5)
N1	C1	C2	112.1(6)
N1	C1	C4	109.7(5)
C4	C1	C2	109.3(5)
N1B	C1B	C2B	112.5(5)
N1B	C1B	C10B	108.9(4)
C2B	C1B	C10B	109.3(5)
C10	C2	C1	111.4(5)
C10	C2	C16	112.0(6)
C16	C2	C1	110.6(6)
C13	C2B	C1B	110.8(5)
O2	C3	O1	124.8(6)
O2	C3	N1	125.6(6)
N1	C3	O1	109.6(5)
O1B	C3B	N1B	109.8(5)
O2B	C3B	O1B	124.8(5)
O2B	C3B	N1B	125.4(6)
O3	C4	C1	111.2(5)
O4	C4	O3	125.8(6)
O4	C4	C1	122.9(6)

Atom	Atom	Atom	Angle/°
O1	C6	C7	109.4(6)
O1	C6	C8	109.3(6)
O1	C6	C9	101.7(5)
C8	C6	C7	114.8(7)
C9	C6	C7	112.1(8)
C9	C6	C8	108.7(7)
O1B	C6B	C7B	110.5(6)
O1B	C6B	C8B	102.6(6)
O1B	C6B	C9B	110.9(6)
C7B	C6B	C8B	108.6(8)
C7B	C6B	C9B	114.5(8)
C8B	C6B	C9B	109.1(7)
C11	C10	C2	120.9(5)
C11	C10	C15	118.5(5)
C15	C10	C2	120.5(5)
O5B	C10B	C1B	112.7(5)
O6B	C10B	O5B	122.6(6)
O6B	C10B	C1B	124.7(6)
C12	C11	C10	121.1(5)
C11	C12	C13	121.5(6)
C12	C13	C2B	122.0(5)
C12	C13	C14	117.3(5)
C14	C13	C2B	120.6(5)
C15	C14	C13	121.5(5)
C14	C15	C10	119.9(5)

Table S5.2.3: Torsion Angles in ° for S54-D2.

Atom	Atom	Atom	Atom	Angle/°
N1	C1	C2	C10	177.7(5)
N1	C1	C2	C16	-57.1(7)
N1	C1	C4	O3	140.4(5)
N1	C1	C4	O4	-42.6(8)
N1B	C1B	C2B	C13	-70.1(6)
N1B	C1B	C10B	O5B	73.0(7)
N1B	C1B	C10B	O6B	-104.9(8)
C1	N1	C3	O1	-175.1(6)
C1	N1	C3	O2	5.6(12)
C1	C2	C10	C11	-108.4(6)
C1	C2	C10	C15	68.6(7)
C1B	N1B	C3B	O1B	170.3(5)
C1B	N1B	C3B	O2B	-11.1(9)
C1B	C2B	C13	C12	97.5(6)
C1B	C2B	C13	C14	-83.3(6)

Atom	Atom	Atom	Atom	Angle/°
C2	C1	C4	O3	-96.3(6)
C2	C1	C4	O4	80.7(7)
C2	C10	C11	C12	178.9(6)
C2	C10	C15	C14	-179.7(6)
C2B	C1B	C10B	O5B	-163.7(6)
C2B	C1B	C10B	O6B	18.3(9)
C2B	C13	C14	C15	-179.4(5)
C3	O1	C6	C7	62.9(8)
C3	O1	C6	C8	-63.6(8)
C3	O1	C6	C9	-178.4(7)
C3	N1	C1	C2	123.0(7)
C3	N1	C1	C4	-115.4(7)
C3B	O1B	C6B	C7B	-74.5(8)
C3B	O1B	C6B	C8B	169.9(7)
C3B	O1B	C6B	C9B	53.6(8)
C3B	N1B	C1B	C2B	136.6(6)
C3B	N1B	C1B	C10B	-102.0(6)
C4	C1	C2	C10	55.8(6)
C4	C1	C2	C16	-179.0(6)
C5	O3	C4	O4	-6.4(9)
C5	O3	C4	C1	170.4(5)
C6	O1	C3	O2	-2.8(11)
C6	O1	C3	N1	177.9(6)
C6B	O1B	C3B	O2B	18.2(10)
C6B	O1B	C3B	N1B	-163.2(5)
C10	C11	C12	C13	-0.1(9)
C10B	C1B	C2B	C13	168.8(5)
C11	C10	C15	C14	-2.7(9)
C11	C12	C13	C2B	178.5(5)
C11	C12	C13	C14	-0.7(8)
C11B	O5B	C10B	O6B	9.4(11)
C11B	O5B	C10B	C1B	-168.6(7)
C12	C13	C14	C15	-0.2(8)
C13	C14	C15	C10	1.9(9)
C15	C10	C11	C12	1.8(9)
C16	C2	C10	C11	127.3(7)
C16	C2	C10	C15	-55.8(8)

Table S5.2.4: Hydrogen Fractional Atomic Coordinates (×10^4) and Equivalent Isotropic Displacement Parameters (Å^2×10^3) for S54-D2. U_{eq} is defined as 1/3 of the trace of the orthogonalised U_{ij}.

Atom	x	y	z	U_{eq}
H1	3410(30)	1240(90)	6740(30)	104
H1B	3270(50)	4160(70)	3540(20)	70(20)
H1A	5604(13)	1541(7)	6025(2)	73

Atom	x	y	z	U_{eq}
H1BA	5810(40)	3140(70)	3892(17)	62
H2	2848(13)	591(7)	5882(3)	72
H2BA	3485(6)	1494(5)	3577(2)	61
H2BB	5128(6)	884(5)	3599(2)	61
H5A	5145(12)	5140(9)	5278(3)	108
H5B	3884(9)	5197(10)	5689(4)	108
H5C	3656(11)	4399(8)	5162(4)	108
H7A	7539(14)	1965(9)	7597(4)	153
H7B	7153(11)	1865(9)	8190(5)	153
H7C	6145(12)	2716(13)	7813(3)	153
H7BA	3178(15)	7012(9)	4851(4)	144
H7BB	4133(12)	8346(13)	4751(3)	144
H7BC	4861(15)	6860(10)	4764(3)	144
H8A	6269(10)	-1032(10)	7379(4)	133
H8B	6869(9)	-1033(10)	7950(5)	133
H8C	7701(14)	-213(9)	7513(3)	133
H8BA	2569(10)	8898(14)	4114(4)	155
H8BB	1686(13)	7501(10)	4175(4)	155
H8BC	2359(10)	7905(8)	3634(6)	155
H9A	4546(10)	1252(15)	8276(3)	149
H9B	5522(11)	-25(13)	8435(4)	149
H9C	4250(13)	-230(14)	8035(3)	149
H9BA	5821(14)	7434(8)	3969(4)	146
H9BB	4875(10)	8740(14)	3817(4)	146
H9BC	4803(10)	7375(9)	3479(5)	146
H11	2040(70)	1270(60)	5160(30)	64
H11A	6827(11)	3601(13)	2372(4)	135
H11B	7079(13)	5171(13)	2527(3)	135
H11C	5506(15)	4642(10)	2393(4)	135
H12	2320(70)	1900(70)	4340(20)	62
H14	6230(70)	750(70)	4410(20)	60
H15	6020(70)	300(70)	5220(30)	62
H16A	3953(12)	-1485(11)	5704(4)	131
H16B	4068(11)	-1018(8)	6288(5)	131
H16C	5407(16)	-837(8)	5915(3)	131

Table S5.2.5: Hydrogen Bond information for S54-D2.

D	H	A	d(D-H)/Å	d(H-A)/Å	d(D-A)/Å	D-H-A/deg
N1B	H1B	O2[1]	1.04(3)	1.98(5)	2.925(7)	150(6)

[1]-1/2+x,1/2-y,1-z

Citations

CrysAlisPro (Rigaku, V1.171.42.58a, 2022)

CrysAlisPro (ROD), Rigaku Oxford Diffraction, Poland (?).

O.V. Dolomanov and L.J. Bourhis and R.J. Gildea and J.A.K. Howard and H. Puschmann, Olex2: A complete structure solution, refinement and analysis program, *J. Appl. Cryst.*, (2009), **42**, 339-341.

Sheldrick, G.M., Crystal structure refinement with ShelXL, *Acta Cryst.*, (2015), **C71**, 3-8.

Sheldrick, G.M., ShelXT-Integrated space-group and crystal-structure determination, *Acta Cryst.*, (2015), **A71**, 3-8.

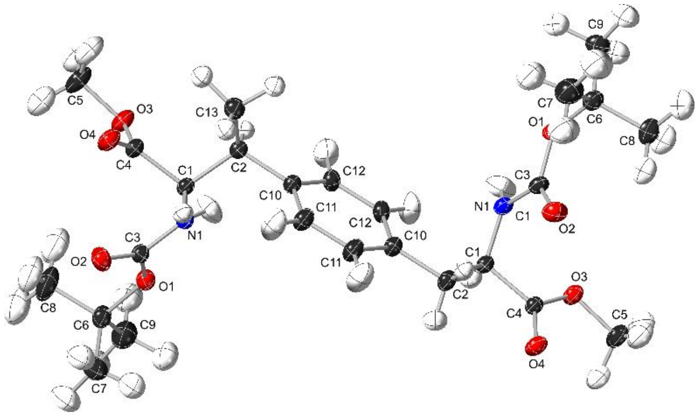

Experimental. Single colorless prism-shaped crystals of **S54-D1** were chosen from the sample as supplied. A suitable crystal with dimensions 0.35 × 0.20 × 0.15 mm^3 was selected and mounted on a loop with paratone on a XtaLAB Synergy-S diffractometer. The crystal was kept at a steady T = 100.01(10) K during data collection. The structure was solved using **ShelXT** (Sheldrick, 2015) using dual-space methods and by using Olex2 1.5-alpha (Dolomanov et al., 2009) as the graphical interface. The model was refined with olex2.refine 1.5-alpha (Bourhis et al., 2015) using full matrix least squares minimisation on $\boldsymbol{F^2}$.

Crystal Data. $C_{25}H_{38}N_2O_8$, M_r = 494.589, monoclinic, *I*2 (No. 5), a = 11.0716(3) Å, b = 9.2054(2) Å, c = 13.5889(3) Å, *b* = 94.839(2)°, *a* = *g* = 90°, *V* = 1380.02(6) Å^3, *T* = 100.01(10) K, *Z* = 2, *Z'* = 0.5, *m*(Cu K$_a$) = 0.732, 16533 reflections measured, 2815 unique (R_{int} = 0.0512) which were used in all calculations. The final wR_2 was 0.0706 (all data) and R_1 was 0.0289 (I≥2 *s*(I)).

**Compound**	**S54-D1**
Formula	$C_{25}H_{38}N_2O_8$
$D_{calc.}$/ g cm^{-3}	1.190
m/mm^{-1}	0.732
Formula Weight	494.589
Colour	colourless
Shape	prism-shaped
Size/mm^3	0.35×0.20×0.15
T/K	100.01(10)
Crystal System	monoclinic
Flack Parameter	-0.10(6)
Hooft Parameter	-0.10(6)
Space Group	*I*2
a/Å	11.0716(3)
b/Å	9.2054(2)
c/Å	13.5889(3)
a/°	90
b/°	94.839(2)
g/°	90
V/Å^3	1380.02(6)
Z	2
Z'	0.5
Wavelength/Å	1.54184
Radiation type	Cu K$_a$
Q_{min}/°	4.95
Q_{max}/°	80.15
Measured Refl's.	16533
Indep't Refl's	2815
Refl's I≥2 *s*(I)	2795
R_{int}	0.0512
Parameters	362
Restraints	612
Largest Peak	0.1088
Deepest Hole	-0.1147
GooF	1.0928
wR_2 (all data)	0.0706
wR_2	0.0703
R_1 (all data)	0.0292
R_1	0.0289

Structure Quality Indicators

**Reflections:**	d min (Cu\a) 2Θ=160.3° 0.78	I/σ(I) 47.2	Rint 5.12%	Full 135.4° 96% to 160.3° 99.9
**Refinement:**	Shift -0.001	Max Peak 0.1	Min Peak -0.1	GooF 1.093
				Hooft -.10(6)

A colourless prism-shaped-shaped crystal with dimensions 0.35 × 0.20 × 0.15 mm^3 was mounted on a loop with paratone. Data were collected using a XtaLAB Synergy, Dualflex, HyPix diffractometer operating at T = 100.01(10) K.

Data were measured using *w* scans with Cu K_a radiation. The diffraction pattern was indexed and the total number of runs and images was based on the strategy calculation from the program CrysAlisPro 1.171.42.58a (Rigaku OD, 2022). The maximum resolution that was achieved was Q = 80.15° (0.78 Å).

The unit cell was refined using CrysAlisPro 1.171.42.58a (Rigaku OD, 2022) on 14337 reflections, 87% of the observed reflections.

Data reduction, scaling and absorption corrections were performed using CrysAlisPro 1.171.42.58a (Rigaku OD, 2022). The final completeness is 99.93 % out to 80.15° in Q. An analytical numeric absorption correction using a multifaceted crystal model based on expressions derived by R.C. Clark & J.S. Reid. (Clark, R. C. & Reid, J. S. (1995). Acta Cryst. A51, 887-897) was performed using CrysAlisPro 1.171.42.58a (Rigaku Oxford Diffraction, 2022). An empirical absorption correction using spherical harmonics, implemented in SCALE3 ABSPACK scaling algorithm was also performed. The absorption coefficient m of this material is 0.732 mm^{-1} at this wavelength (l = 1.54184Å) and the minimum and maximum transmissions are 0.825 and 0.919.

The structure was solved and the space group *I*2 (# 5) determined by **ShelXT** (Sheldrick, 2015) using dual-space methods and refined by full matrix least squares minimisation on $\boldsymbol{F}^2$ using version of olex2.refine 1.5-alpha (Bourhis et al., 2015). All atoms, even hydrogen atoms, were refined anisotropically. Hydrogen atom positions were located from the electron densities and freely refined using Hirshfeld scattering factors. Refinement was by using NoSpherA2, an implementation of non-spherical atom-form-factors (F. Kleemiss, H. Puschmann, O. Dolomanov, S.Grabowsky - https://doi.org/10.1039/D0SC05526C – 2020). NoSpherA2 implementation of HAR makes use of tailor-made aspherical atomic form factors calculated from a Hirshfeld-partitioned electron density (ED) not from spherical-atom form factors. The ED was calculated from a Gaussian basis set single determinant SCF wavefunction from DFT using selected functionals for a fragment of this crystal.

The Flack parameter was refined to -0.1(1). Determination of absolute structure using Bayesian statistics on Bijvoet differences using the Olex2 results in -0.1(1). Note: The Flack parameter is used to determine chirality of the crystal studied, the value should be near 0, a value of 1 means that the stereochemistry is wrong and the model should be inverted. A value of 0.5 means that the crystal consists of a racemic mixture of the two enantiomers.

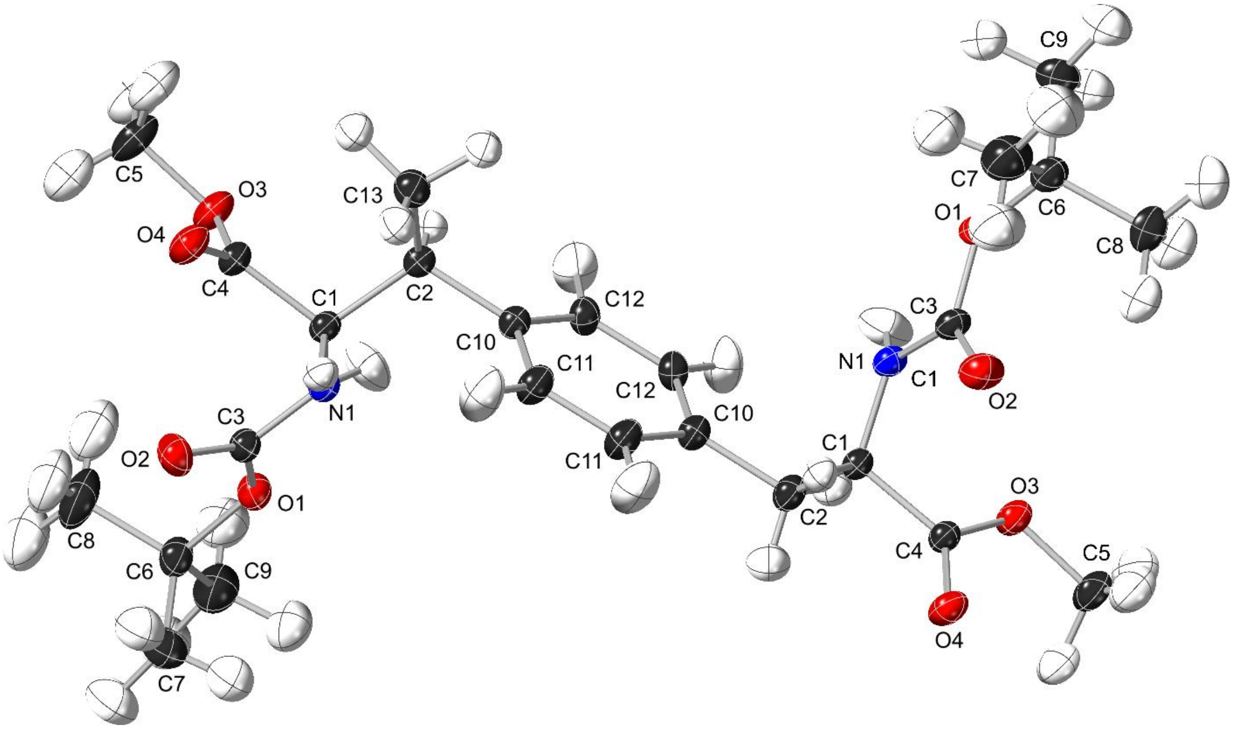

Figure S5.1.2: The molecule lies on a two-fold axis that intersects the mid-point of the phenyl ring and the asymmetric unit contains one half of the atomic positions of the chiral molecular structure; the other half are symmetry generated atoms (Z is 2 and Z' is ½). The two halves are crystallographically identical entities, but the atom labelled C2 corresponds to both the -CH2- group and the -C(Me)(H)- group that both the share the same site. Only one is shown here. However, the methyl group and the hydrogens could be freely refined and their positions accurately determined in this disordered structure (including those for the H atoms). The ellipses are shown at the 50% probability level.

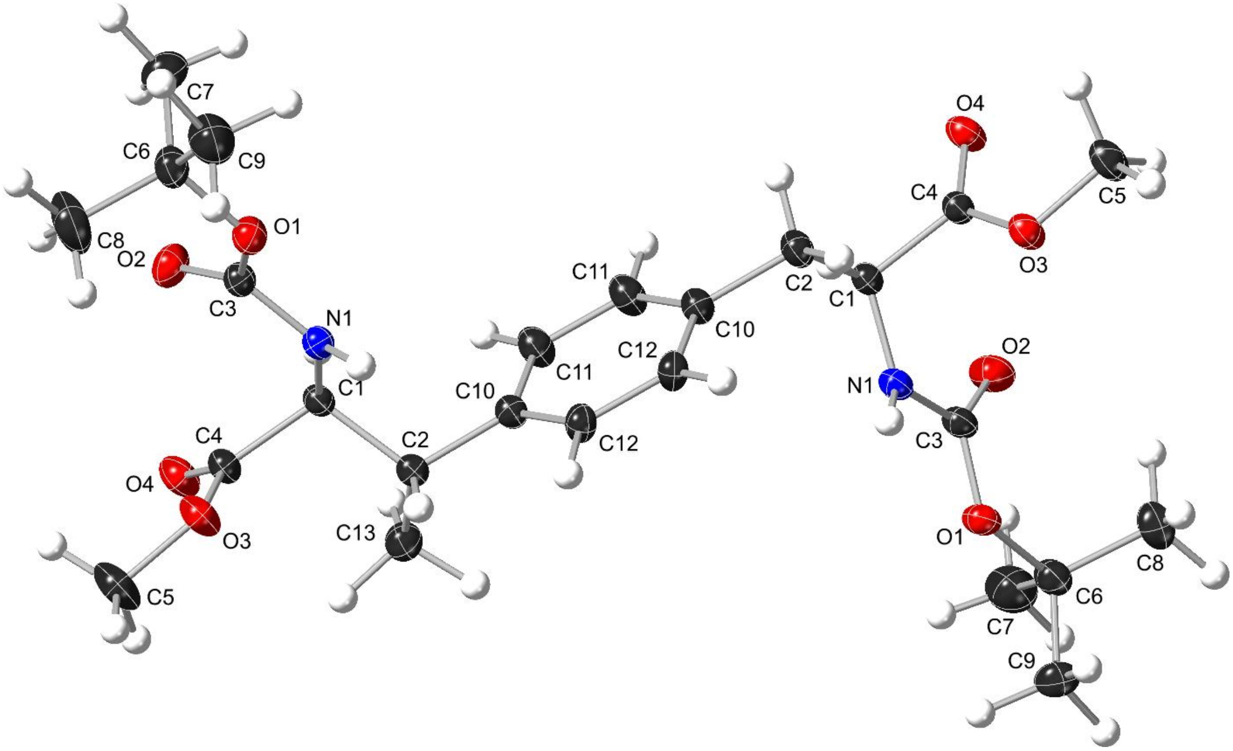

Figure S5.1.2:

Data Plots: Diffraction Data

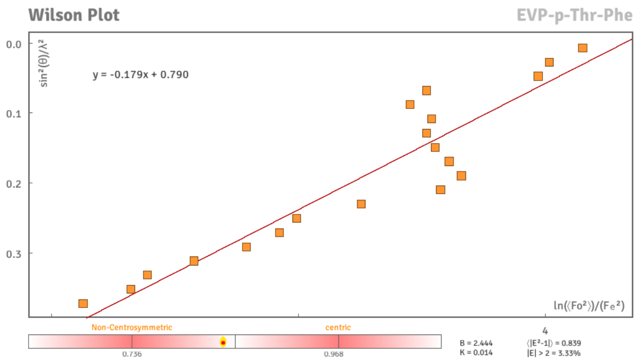

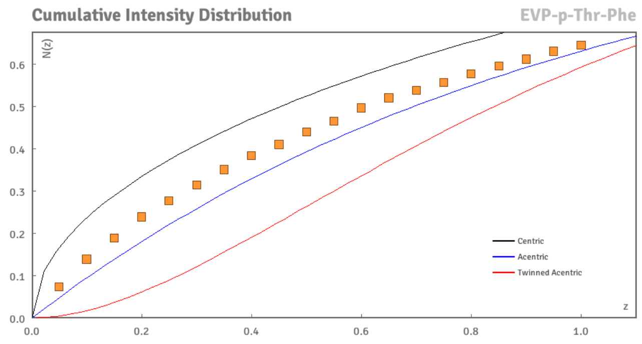

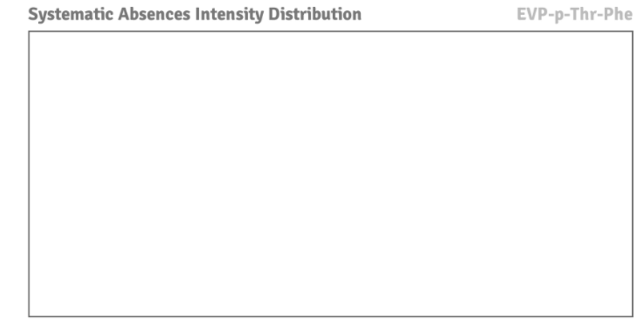

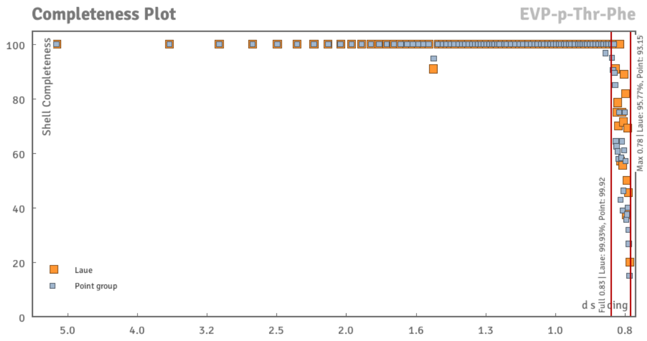

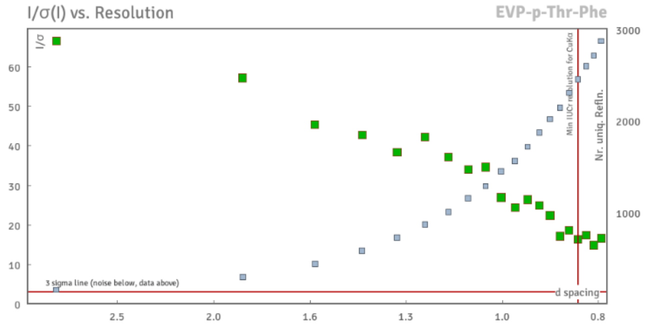

Data Plots: Refinement and Data

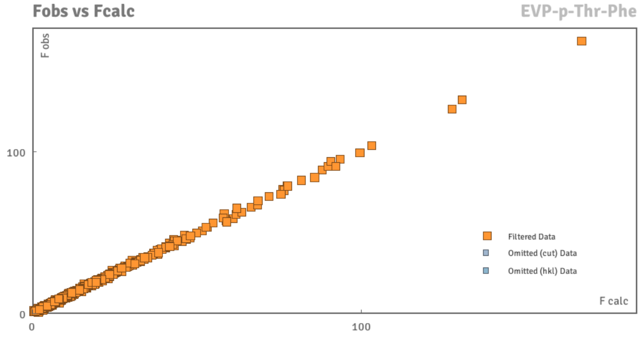

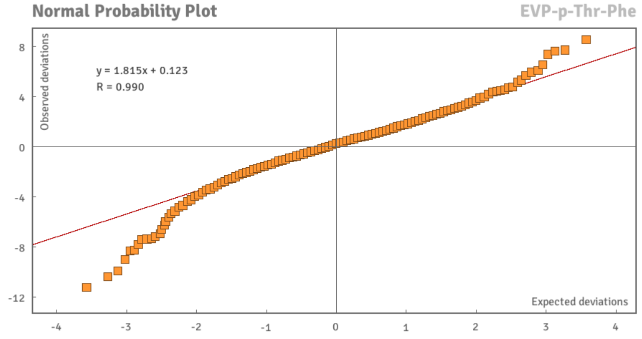

Reflection Statistics

Total reflections (after filtering)	16533	Unique reflections	2815
Completeness	0.932	Mean I/*s*	32.41
hkl_{max} collected	(13, 11, 16)	hkl_{min} collected	(-14, -11, -17)
hkl_{max} used	(14, 11, 17)	hkl_{min} used	(-14, -11, 0)
Lim d_{max} collected	100.0	Lim d_{min} collected	0.77
d_{max} used	8.93	d_{min} used	0.78
Friedel pairs	2104	Friedel pairs merged	0
Inconsistent equivalents	48	R_{int}	0.0512
R_{sigma}	0.0212	Intensity transformed	0
Omitted reflections	0	Omitted by user (OMIT hkl)	15
Multiplicity	(892, 1028, 883, 792, 606, 386, 169, 82, 42, 15, 5)	Maximum multiplicity	16
Removed systematic absences	0	Filtered off (Shel/OMIT)	0

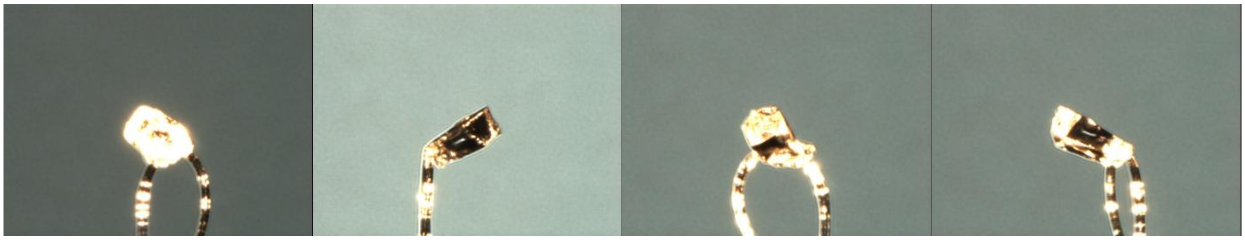

Table S5.6.1: Fractional Atomic Coordinates (×10^4) and Equivalent Isotropic Displacement Parameters (Å^2×10^3) for S54-D1. U_{eq} is defined as 1/3 of the trace of the orthogonalised U_{ij}.

Atom	x	y	z	U_{eq}
O1	5071.4(7)	4310.9(10)	8531.1(6)	26.96(19)
O2	5437.3(8)	6301.2(11)	7600.8(7)	32.1(2)
O3	7209.8(8)	4445.2(10)	6188.6(6)	28.4(2)
O4	7678.2(9)	6800.2(10)	6117.1(6)	28.7(2)
N1	6949.1(9)	4796.0(11)	8190.5(7)	21.3(2)
C1	7842.6(10)	5618.2(13)	7710.0(8)	20.2(2)
C2	9109.9(11)	4931.5(15)	7908.4(9)	24.9(2)
C13	9969(3)	5598(4)	7282(2)	33.4(6)
C3	5778.7(10)	5224.7(13)	8068.7(8)	22.6(2)
C4	7551.4(11)	5709.8(13)	6596.4(9)	21.3(2)
C5	7042.8(15)	4461.1(18)	5125.0(9)	39.3(3)
C6	3755.0(10)	4498.5(18)	8474.2(9)	34.2(3)
C7	3436.7(15)	5916(2)	8970.4(13)	46.2(4)
C8	3238.9(16)	4433(3)	7401.6(12)	58.0(5)
C9	3335.7(15)	3227(2)	9057.4(13)	45.1(4)
C10	9560.3(10)	4949.2(13)	8993.1(8)	22.7(2)
C11	9784.0(12)	6255.0(14)	9505.3(9)	28.6(3)
C12	9783.5(11)	3649.4(14)	9502.0(9)	24.1(3)

Table S5.1.2: Anisotropic Displacement Parameters (×10^4) for S54-D1. The anisotropic displacement factor exponent takes the form: $-2p^2[h^2a^{*2} \times U_{11} + ... + 2hka^* \times b^* \times U_{12}]$

Atom	U_{11}	U_{22}	U_{33}	U_{23}	U_{13}	U_{12}
O1	23.7(4)	34.6(5)	22.9(4)	0.8(3)	4.3(3)	9.1(4)
O2	28.7(4)	34.0(5)	33.7(5)	7.4(4)	4.1(4)	15.1(4)
O3	41.2(5)	25.8(4)	18.0(4)	-8.2(4)	1.8(3)	-1.4(3)
O4	43.8(5)	23.4(4)	19.0(4)	-4.0(3)	3.3(3)	3.3(3)
N1	23.0(4)	23.3(5)	17.8(4)	2.0(3)	2.4(3)	3.4(4)
H1	30(6)	28(2)	60(11)	5.9(14)	10(3)	18(2)
C1	22.6(5)	21.8(5)	16.1(5)	0.2(4)	1.3(3)	-0.4(4)
H1a	27(6)	22.9(16)	22(5)	0.2(11)	2(2)	-2.3(10)
C2	23.6(5)	32.6(6)	18.4(5)	4.1(4)	0.9(4)	-2.2(4)
C13	22.7(11)	50.1(14)	27.5(12)	2.2(7)	3.4(7)	2.6(8)
H13a	24.0(19)	50(4)	27(4)	2.5(12)	3.9(10)	3.4(18)
H13b	31(3)	41(3)	27.7(17)	2.0(13)	2.5(8)	3.8(9)
H13c	26(5)	48.3(19)	30(5)	1.8(10)	2.5(18)	2.3(10)
H2ba	29(4)	34.1(16)	26(3)	2.4(8)	5.8(12)	-3.5(7)
H2bb	31(4)	36(2)	32(3)	1.0(10)	9.2(14)	-0.2(9)

Atom	U_{11}	U_{22}	U_{33}	U_{23}	U_{13}	U_{12}
C3	23.9(5)	25.5(6)	18.5(5)	2.7(4)	2.7(3)	5.4(4)
C4	25.1(5)	21.5(5)	17.2(5)	-2.1(4)	0.9(4)	0.7(3)
C5	56.2(9)	42.8(8)	18.6(6)	-14.6(8)	0.7(5)	-3.6(5)
H5a	70(7)	45.7(19)	34(6)	-17.1(13)	4(3)	-8.0(12)
H5b	63(3)	55(5)	30(6)	-7.1(18)	0.5(17)	1(2)
H5c	60(3)	57(6)	24(5)	-18.2(17)	3.4(15)	-2(2)
C6	22.7(5)	57.1(8)	22.8(6)	0.8(5)	1.7(4)	7.5(5)
C7	34.2(8)	63.2(10)	42.3(9)	13.1(6)	10.3(6)	3.8(7)
H7a	43(6)	67(3)	52(5)	11.2(18)	13(2)	7.3(18)
H7b	36.0(17)	79(7)	64(7)	13.7(11)	12.9(11)	3(3)
H7c	43(5)	67(6)	45(2)	11(2)	6.5(14)	3.2(13)
C8	45.2(9)	97.7(15)	28.8(7)	-17.3(10)	-10.3(6)	11.1(8)
H8a	51(7)	101(4)	36(6)	-18(2)	-9(3)	15.2(19)
H8b	59(6)	101(3)	40(6)	-15(2)	-5(3)	7.0(18)
H8c	46.4(18)	111(7)	48(7)	-18.6(13)	-10.1(10)	11(3)
C9	31.7(8)	68.8(11)	35.6(8)	-12.6(7)	7.6(6)	9.9(7)
H9a	43(6)	71(3)	45(5)	-10.9(17)	11(2)	6.1(17)
H9b	41(5)	62(7)	37(2)	-9(3)	4.4(13)	9.5(14)
H9c	33.4(17)	87(7)	50(6)	-11.3(11)	9.6(11)	10(3)
C10	24.3(5)	24.3(5)	19.3(5)	2.5(4)	0.4(4)	-0.9(4)
C11	41.7(7)	22.6(6)	20.9(6)	1.2(5)	-1.3(5)	-0.1(5)
H11	74(13)	28(3)	37(8)	3(3)	-7(6)	7(2)
C12	23.2(6)	22.9(6)	24.9(6)	-0.1(4)	-4.9(4)	-1.5(4)
H12	51(11)	27(3)	47(8)	-1(3)	-10(6)	-11(2)
H2a	27(4)	33.3(15)	26(3)	3.2(8)	5.1(12)	-3.2(7)

Table S5.1.3: Bond Lengths in Å for S54-D1.

Atom	Atom	Length/Å
O1	C3	1.3412(14)
O1	C6	1.4632(14)
O2	C3	1.2200(14)
O3	C4	1.3304(14)
O3	C5	1.4417(15)
O4	C4	1.2111(15)
N1	C1	1.4445(14)
N1	C3	1.3513(15)
C1	C2	1.5418(16)
C1	C4	1.5228(15)
C2	C13	1.464(3)
C2	C10	1.5158(15)
C6	C7	1.524(2)
C6	C8	1.5212(19)
C6	C9	1.509(2)
C10	C11	1.4006(18)
C10	C12	1.3936(16)
C11	C11[1]	1.389(2)
C12	C12[1]	1.397(2)

—— [1]2-x,+y,2-z

Table S5.1.4: Bond Angles in ° for S54-D1.

Atom	Atom	Atom	Angle/°
C6	O1	C3	121.30(10)
C5	O3	C4	114.62(10)
C3	N1	C1	118.54(9)
C2	C1	N1	110.73(10)
C4	C1	N1	112.49(9)
C4	C1	C2	108.04(9)
C13	C2	C1	110.67(15)
C10	C2	C1	112.67(9)
C10	C2	C13	112.62(15)
O2	C3	O1	125.88(10)
N1	C3	O1	110.56(9)
N1	C3	O2	123.56(11)
O4	C4	O3	122.88(10)
C1	C4	O3	113.23(9)
C1	C4	O4	123.78(10)
C7	C6	O1	110.14(12)
C8	C6	O1	109.75(11)
C8	C6	C7	112.09(16)
C9	C6	O1	103.29(12)
C9	C6	C7	109.82(12)
C9	C6	C8	111.41(15)
C11	C10	C2	121.49(11)
C12	C10	C2	120.22(11)
C12	C10	C11	118.28(10)
C11[1]	C11	C10	120.88(7)
C12[1]	C12	C10	120.84(7)

—— [1]2-x,+y,2-z

Table S5.1.5: Torsion Angles in ° for S54-D1.

Atom	Atom	Atom	Atom	Angle/°
O1	C3	N1	C1	177.57(9)
O2	C3	N1	C1	-2.58(15)
O3	C4	C1	N1	-44.80(11)
O3	C4	C1	C2	77.74(11)
O4	C4	C1	N1	138.98(12)
O4	C4	C1	C2	-98.48(13)
N1	C1	C2	C13	169.67(16)
N1	C1	C2	C10	-63.23(10)
C1	C2	C10	C11	-63.89(13)
C1	C2	C10	C12	117.27(11)
C2	C10	C11	C11[1]	-178.90(15)
C2	C10	C12	C12[1]	179.01(13)
C10	C11	C11[1]	C10[1]	-0.00(13)
C10	C12	C12[1]	C10[1]	-0.19(12)

[1]2-x,+y,2-z

Table S5.1.6: Hydrogen Fractional Atomic Coordinates (×10^4) and Equivalent Isotropic Displacement Parameters (Å^2×10^3) for S54-D2. U_{eq} is defined as 1/3 of the trace of the orthogonalised U_{ij}.

Atom	x	y	z	U_{eq}
H1	7131(15)	3775(18)	8407(14)	39(4)
H1a	7852(12)	6724(16)	7958(10)	24(2)
H13a	10882(11)	5170(30)	7497(19)	33(2)
H13b	9780(20)	5370(30)	6489(6)	33(2)
H13c	10010(20)	6786(6)	7370(20)	35(2)
H2ba	9100(40)	3810(40)	7590(20)	30(2)
H2bb	9670(30)	5580(30)	7580(30)	33(2)
H5a	6909(18)	3320(20)	4909(12)	50(3)
H5b	6315(17)	5130(20)	4912(12)	50(3)
H5c	7878(16)	4840(20)	4844(11)	47(3)
H7a	3756(16)	6840(20)	8590(14)	53(3)
H7b	2467(16)	6010(20)	9000(14)	59(3)
H7c	3876(15)	5980(20)	9630(13)	52(3)
H8a	3516(18)	5420(30)	7011(15)	63(3)
H8b	3550(20)	3410(30)	7063(15)	67(3)
H8c	2276(17)	4290(30)	7341(13)	69(3)
H9a	3568(17)	2250(20)	8685(14)	53(3)
H9b	3776(16)	3260(20)	9820(13)	47(3)
H9c	2355(16)	3280(20)	9108(14)	57(3)
H11	9618(18)	7324(18)	9144(13)	47(5)

Atom	x	y	z	U_{eq}
H12	9665(16)	2602(18)	9147(12)	43(4)
H2a	9000(40)	3760(50)	7790(20)	29(2)

Table S5.1.7: Atomic Occupancies for all atoms that are not fully occupied in S54-D1

Atom	Occupancy
C13	0.500000
H13a	0.500000
H13b	0.500000
H13c	0.500000
H2ba	0.500000
H2bb	0.500000
H2a	0.500000

Table S5.1.8: Selected Bond Lengths in Å for S54-D1.

Atom	Atom	Length/Å
N1	H1	1.000(16)
C1	H1a	1.072(14)
C2	H2ba	1.12(3)
C2	H2bb	1.00(3)
C2	H2a	1.10(4)
C13	H13a	1.101(3)
C13	H13b	1.100(3)
C13	H13c	1.101(3)
C5	H5a	1.095(18)
C5	H5b	1.03(2)
C5	H5c	1.089(17)
C7	H7a	1.07(2)
C7	H7b	1.081(17)
C7	H7c	0.984(18)
C8	H8a	1.11(2)
C8	H8b	1.12(3)
C8	H8c	1.071(18)
C9	H9a	1.07(2)
C9	H9b	1.108(18)
C9	H9c	1.095(17)
C11	H11	1.108(16)
C12	H12	1.081(16)

[1]2-x,+y,2-z

Table S5.1.9: Selected Bond Angles in ° for S54-D1.

Atom	Atom	Atom	Angle/°
C1	N1	H1	119.8(9)
C3	N1	H1	118.5(9)
H1a	C1	N1	110.1(7)
C2	C1	H1a	110.5(7)
C4	C1	H1a	104.8(7)
H2ba	C2	C1	109(2)
H2ba	C2	C13	98.6(16)
H2bb	C2	C1	105(2)
H2bb	C2	C13	13(2)
H2bb	C2	H2ba	112(2)
C10	C2	H2ba	112.3(19)
C10	C2	H2bb	106(2)
H2a	C2	C1	107(2)
H2a	C2	C13	113.3(18)
H2a	C2	H2ba	16(3)
H2a	C2	H2bb	126(3)
H2a	C2	C10	100.0(19)
H13a	C13	C2	108.8(15)

Atom	Atom	Atom	Angle/°
H13b	C13	C2	114.1(15)
H13b	C13	H13a	106.8(16)
H13c	C13	C2	112.1(15)
H13c	C13	H13a	107.7(16)
H13c	C13	H13b	107.1(17)
H5a	C5	O3	105.2(9)
H5b	C5	O3	108.5(9)
H5b	C5	H5a	114.2(14)
H5c	C5	O3	108.3(8)
H5c	C5	H5a	108.3(14)
H5c	C5	H5b	112.0(14)
H7a	C7	C6	111.6(10)
H7b	C7	C6	110.8(12)
H7b	C7	H7a	108.7(15)
H7c	C7	C6	109.7(11)
H7c	C7	H7a	103.7(15)
H7c	C7	H7b	112.1(14)
H8a	C8	C6	109.2(10)
H8b	C8	C6	108.8(11)
H8b	C8	H8a	112.8(15)
H8c	C8	C6	111.7(10)
H8c	C8	H8a	112.0(17)
H8c	C8	H8b	102.1(18)
H9a	C9	C6	107.8(10)
H9b	C9	C6	109.8(10)
H9b	C9	H9a	111.2(15)
H9c	C9	C6	110.3(11)
H9c	C9	H9a	110.3(15)
H9c	C9	H9b	107.5(13)
H11	C11	C10	121.8(9)
H12	C12	C10	122.3(9)

[1]2-x,+y,2-z

Citations

CrysAlisPro (Rigaku, V1.171.42.58a, 2022)

CrysAlisPro (ROD), Rigaku Oxford Diffraction, Poland (?).

L.J. Bourhis and O.V. Dolomanov and R.J. Gildea and J.A.K. Howard and H. Puschmann, The Anatomy of a Comprehensive Constrained, Restrained, Refinement Program for the Modern Computing Environment - Olex2 Disected, *Acta Cryst. A*, (2015), **A71**, 59-71.

O.V. Dolomanov and L.J. Bourhis and R.J. Gildea and J.A.K. Howard and H. Puschmann, Olex2: A complete structure solution, refinement and analysis program, *J. Appl. Cryst.*, (2009), **42**, 339-341.

6. References

(1) Nasir Baig, R. B.; Sai Sudhir, V.; Chandrasekaran, S. Simple and Efficient Synthesis of Allo- and Threo-3,3′-Dimethylcystine Derivatives in Enantiomerically Pure Form. *Tetrahedron Asymmetry* **2008**, *19* (12), 1425–1429. https://doi.org/10.1016/j.tetasy.2008.06.001.

(2) Carson, W. P.; Sarver, P. J.; Goudy, N. S.; MacMillan, D. W. C. Photoredox Catalysis-Enabled Sulfination of Alcohols and Bromides. *J. Am. Chem. Soc.* **2023**, *145* (38), 20767–20774. https://doi.org/10.1021/jacs.3c08216.

(3) Liu, S.; Cai, C.; Bai, Z.; Sheng, W.; Tan, J.; Wang, H. Late-Stage Macrocyclization of Bioactive Peptides with Internal Oxazole Motifs via Palladium-Catalyzed C-H Olefination. *Org. Lett.* **2021**, *23* (8), 2933–2937. https://doi.org/10.1021/acs.orglett.1c00580.

(4) Garg, N. K.; Sarpong, R.; Stoltz, B. M. The First Total Synthesis of Dragmacidin D. *J. Am. Chem. Soc.* **2002**, *124* (44), 13179–13184. https://doi.org/10.1021/ja027822b.

(5) Hoffer, L.; Garcia, M.; Leblanc, R.; Feracci, M.; Betzi, S.; Ben Yaala, K.; Daulat, A. M.; Zimmermann, P.; Roche, P.; Barral, K.; Morelli, X. Discovery of a PDZ Domain Inhibitor Targeting the Syndecan/Syntenin Protein-Protein Interaction: A Semi-Automated "Hit Identification-to-Optimization" Approach. *J. Med. Chem.* **2023**, *66* (7), 4633–4658. https://doi.org/10.1021/acs.jmedchem.2c01569.

(6) Liu, N. N.; Zhao, S. M.; Zhao, J. F.; Zeng, G. Z.; Tan, N. H.; Liu, J. P. Synthesis and Conformation Studies of Rubiyunnanin B Analogs. *Tetrahedron* **2014**, *70* (37), 6630–6640. https://doi.org/10.1016/j.tet.2014.06.108.

(7) Bartoccini, F.; Fanini, F.; Retini, M.; Piersanti, G. General Synthesis of Unnatural 4-, 5-, 6-, and 7-Bromo-D-Tryptophans by Means of a Regioselective Indole Alkylation. *Tetrahedron Lett.* **2020**, *61* (22), 151923. https://doi.org/10.1016/j.tetlet.2020.151923.

(8) Zwick, C. R.; Sosa, M. B.; Renata, H. Modular Chemoenzymatic Synthesis of GE81112 B1 and Related Analogs Enables Elucidation of Its Key Pharmacophores. *J. Am. Chem. Soc.* **2021**, *143* (3), 1673–1679. https://doi.org/10.1021/jacs.0c13424.Modular.

(9) Hibi, M.; Mori, R.; Miyake, R.; Kawabata, H.; Kozono, S.; Takahashi, S.; Ogawa, J. Novel Enzyme Family Found in Filamentous Fungi Catalyzing Trans-4-Hydroxylation of L-Pipecolic Acid. *Appl. Environ. Microbiol.* **2016**, *82* (7), 2070–2077. https://doi.org/10.1128/AEM.03764-15.

(10) Ju, J.; Ozanick, S. G.; Shen, B.; Thomas, M. G. Conversion of (2S)-Arginine to (2S,3R)-Capreomycidine by VioC and VioD from the Viomycin Biosynthetic Pathway of Streptomyces Sp. Strain ATCC11861. *ChemBioChem* **2004**, *5* (9), 1281–1285. https://doi.org/10.1002/cbic.200400136.

(11) Yin, X.; Zabriskie, T. M. VioC Is a Non-Heme Iron, α-Ketoglutarate-Dependent Oxygenase That Catalyzes the Formation of 3S-Hydroxy-L-Arginine during Viomycin Biosynthesis. *ChemBioChem* **2004**, *5* (9), 1274–1277. https://doi.org/10.1002/cbic.200400082.

(12) Sakai, A.; Xiang, D. F.; Xu, C.; Song, L.; Yew, W. S.; Raushel, F. M.; Gerlt, J. A. Evolution of Enzymatic Activities in the Enolase Superfamily: N-Succinylamino Acid Racemase and a New Pathway for the Irreversible Conversion of D-to L-Amino Acids. *Biochemistry* **2006**, *45*, 4455–4462. https://doi.org/https://doi.org/10.1021/bi060230b.

(13) Amatuni, A.; Shuster, A.; Abegg, D.; Adibekian, A.; Renata, H. Comprehensive Structure−Activity Relationship Studies of Cepafungin Enabled by Biocatalytic C−H Oxidations. *ACS Cent. Sci.* **2023**, *9* (2), 239–251. https://doi.org/10.1021/acscentsci.2c01219.

(14) Vitale, R.; Ottonello, G.; Petracca, R.; Bertozzi, S. M.; Ponzano, S.; Armirotti, A.; Berteotti, A.; Dionisi, M.; Cavalli, A.; Piomelli, D.; Bandiera, T.; Bertozzi, F. Synthesis, Structure-Activity, and Structure-Stability Relationships of 2-Substituted-n-(4-Oxo-3-Oxetanyl) n-Acylethanolamine Acid Amidase (NAAA) Inhibitors. *ChemMedChem* **2014**, *9* (2), 323–336. https://doi.org/10.1002/cmdc.201300416.

(15) Allouche, E. M. D.; Simonet-Davin, R.; Waser, J. N-Terminal Selective C−H Azidation of Proline-Containing Peptides: A Platform for Late-Stage Diversification. *Chem. - A Eur. J.* **2022**, *28* (17), 1–6. https://doi.org/10.1002/chem.202200368.

(16) Zhang, X.; King-Smith, E.; Renata, H. Total Synthesis of Tambromycin by Combining Chemocatalytic and Biocatalytic C−H Functionalization. *Angew. Chemie - Int. Ed.* **2018**, *57* (18), 5037–5041. https://doi.org/10.1002/anie.201801165.

(17) Brown, A. D.; Bagal, S. K.; Blackwell, P.; Blakemore, D. C.; Brown, B.; Bungay, P. J.; Corless, M.; Crawforth, J.; Fengas, D.; Fenwick, D. R.; Gray, V.; Kemp, M.; Klute, W.; Malet Sanz, L.; Miller, D.; Murata, Y.; Payne, C. E.; Skerratt, S.; Stevens, E. B.; Warmus, J. S. The Discovery and Optimization of Benzimidazoles as Selective NaV1.8 Blockers for the Treatment of Pain. *Bioorganic Med. Chem.* **2019**, *27* (1), 230–239. https://doi.org/10.1016/j.bmc.2018.12.002.

(18) Bollu, A.; Sharma, N. K. Synthesis and Conformational Analysis of Aminopyrazolonyl Amino Acid (APA)/Peptides. *European J. Org. Chem.* **2019**, *2019* (6), 1286–1292. https://doi.org/10.1002/ejoc.201801640.

(19) Meyer, F. M.; Liras, S.; Guzman-Perez, A.; Perreault, C.; Bian, J.; James, K. Functionalization of Aromatic Amino Acids via Direct C-H Activation: Generation of Versatile Building Blocks for Accessing Novel Peptide Space. *Org. Lett.* **2010**, *12* (17), 3870–3873. https://doi.org/10.1021/ol1015674.

(20) Zhang, Q.; Ding, Q.; Song, C.; Chang, J. Asymmetric Synthesis of Methyl N-(Tert-Butoxycarbonyl)Indoline-2-Carboxylates. *Chinese J. Org. Chem.* **2018**, *38* (1), 221–227. https://doi.org/10.6023/cjoc201708002.

(21) Chiu, K. W.; Tseng, Y. H.; Li, Y. X.; Chein, R. J. Biomimetic Total Synthesis of Clavicipitic Acid: A DDQ-Mediated Intramolecular Cross-Dehydrogenative Coupling Approach. *Org. Lett.* **2023**, *25* (19), 3456–3460. https://doi.org/10.1021/acs.orglett.3c01029.

(22) Shendage, D. M.; Fröhlich, R.; Haufe, G. Highly Efficient Stereoconservative Amidation and Deamidation of α-Amino Acids. *Org. Lett.* **2004**, *6* (21), 3675–3678.

https://doi.org/10.1021/ol048771l.

(23) Reddy, M. Compounds and Methods for Treating Bacterial Infections. US 2014/0206677 A1, 2014.

(24) Wang, X.; Corcilius, L.; Premdjee, B.; Payne, R. J. Synthesis and Utility of β-Selenophenylalanine and β-Selenoleucine in Diselenide-Selenoester Ligation. *J. Org. Chem.* **2020**, *85* (3), 1567–1578. https://doi.org/10.1021/acs.joc.9b02665.

(25) Ghosh, K. C.; Duttagupta, I.; Bose, C.; Banerjee, P.; Gayen, A. K.; Sinha, S. Synthesis and Anticancer Activities of Proline-Containing Cyclic Peptides and Their Linear Analogs and Congeners. *Synth. Commun.* **2019**, *49* (2), 221–236. https://doi.org/10.1080/00397911.2018.1550201.

(26) Ranganathan, D.; Vaish, N. K.; Shah, K. Protein Backbone Modification by Novel Ca-C Side-Chain Scission. *J. Am. Chem. Soc.* **1994**, *116* (15), 6545–6557. https://doi.org/10.1021/ja00094a008.

(27) Manfrin, A.; Borduas-Dedekind, N.; Lau, K.; McNeill, K. Singlet Oxygen Photooxidation of Peptidic Oxazoles and Thiazoles. *J. Org. Chem.* **2019**, *84* (5), 2439–2447. https://doi.org/10.1021/acs.joc.8b02684.

(28) Liu, Y.; Zhao, X.; Wang, H.; Liu, H.; Sui, Z.; Yan, B.; Du, Y. Total Synthesis of the Proposed Microcyclamides MZ602 and MZ568. *J. Org. Chem.* **2021**, *86* (1), 1065–1073. https://doi.org/10.1021/acs.joc.0c02541.

(29) Delgado, O.; Martin Müller, H.; Bach, T. Concise Total Synthesis of the Thiazolyl Peptide Antibiotic GE2270 A. *Chem. - A Eur. J.* **2008**, *14* (8), 2322–2339. https://doi.org/10.1002/chem.200701823.

(30) Moskal, J. R.; Meltzer, H. Subacute Administration of NMDA Modulators Alone or in Combination. US 2018/0325893 A1, 2018.

(31) Tang, Z.; Yang, Z. H.; Cun, L. F.; Gong, L. Z.; Mi, A. Q.; Jiang, Y. Z. Small Peptides Catalyze Highly Enantioselective Direct Aldol Reactions of Aldehydes with Hydroxyacetone: Unprecedented Regiocontrol in Aqueous Media. *Org. Lett.* **2004**, *6* (13), 2285–2287. https://doi.org/10.1021/ol049141m.

(32) Bentley, D. J.; Moody, C. J. Asymmetric Synthesis of the Central Tryptophan Residue of Stephanotic Acid. *Org. Biomol. Chem.* **2004**, *2* (24), 3545–3547. https://doi.org/10.1039/b414996c.

7. NMR Spectra

¹H NMR (600 MHz, $CDCl_3$) of compound **S12**

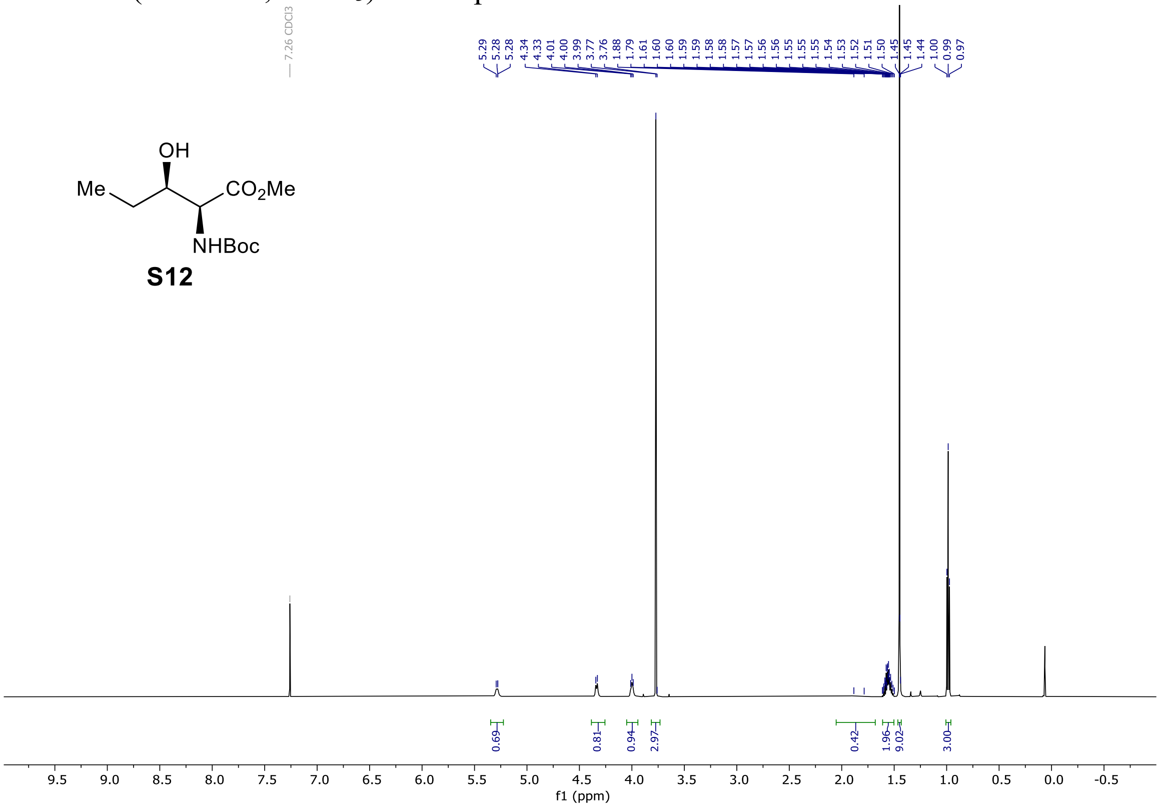

¹³C NMR (151 MHz, $CDCl_3$) of compound **S12**

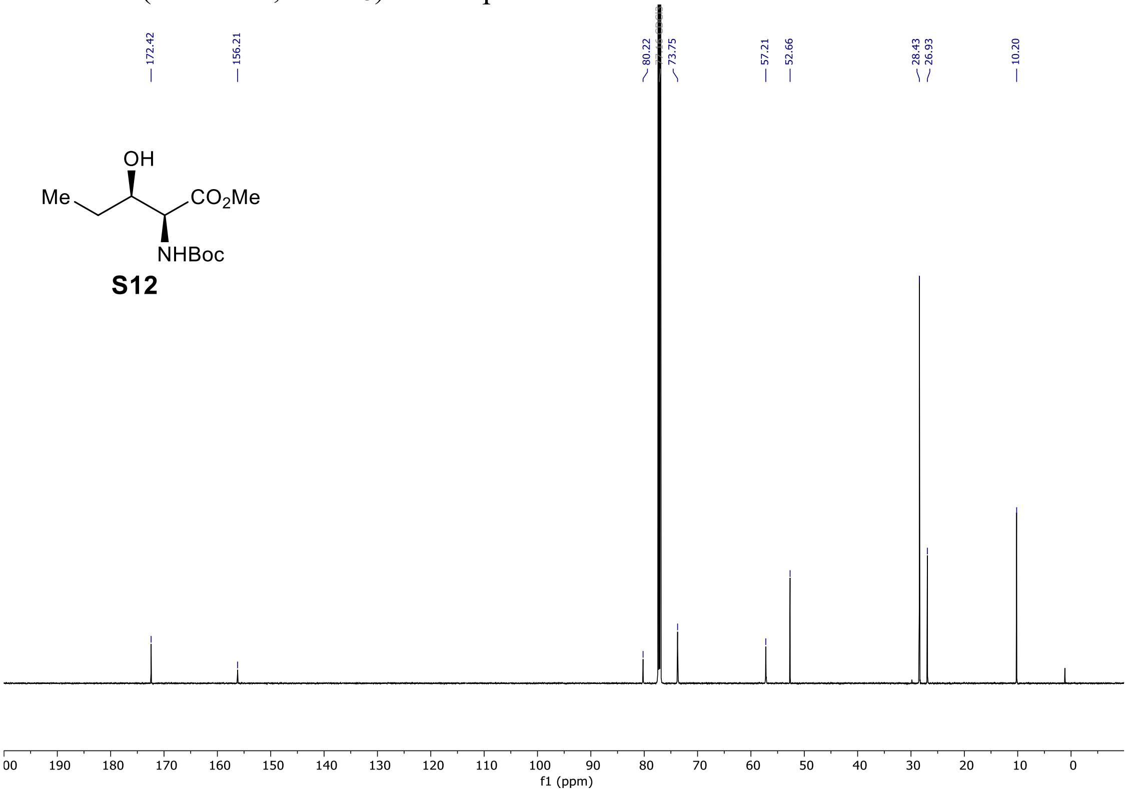

^{1}H NMR (600 MHz, $CDCl_3$) of compound **S14**

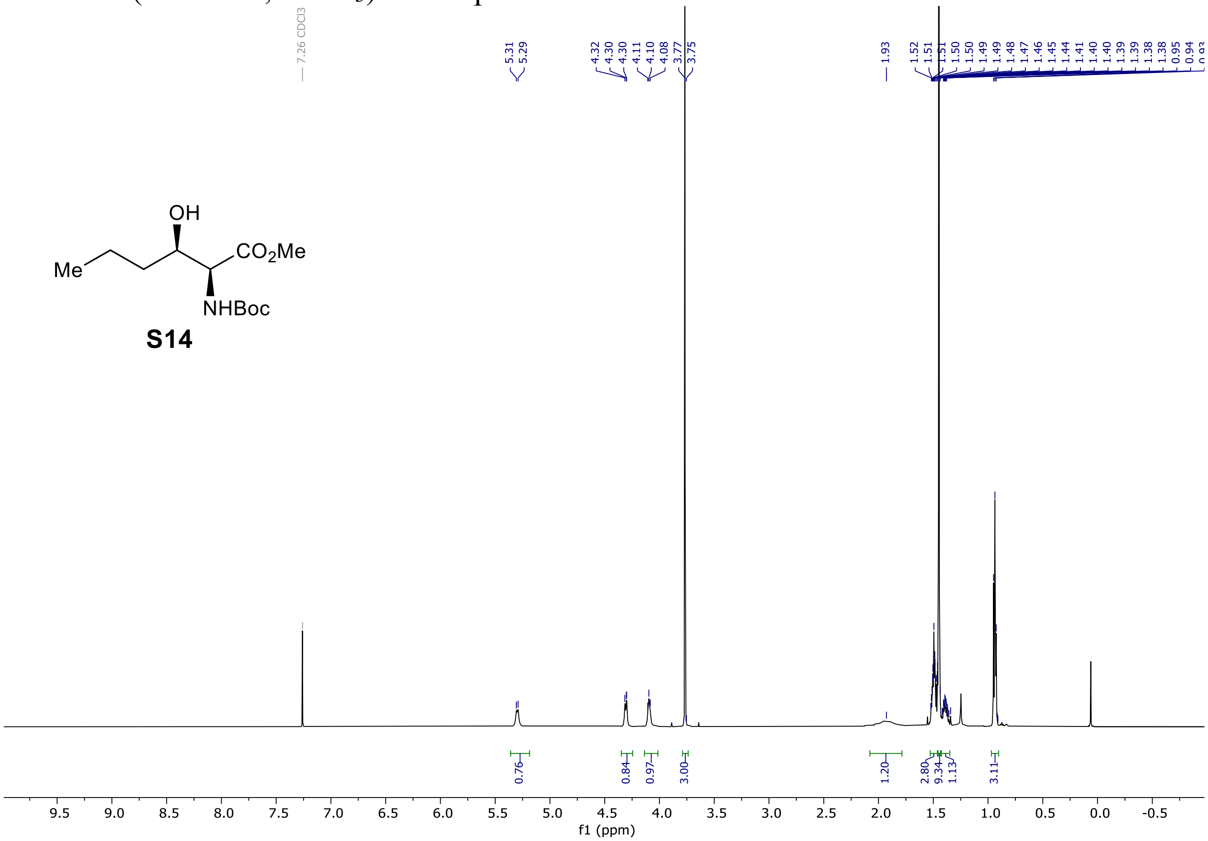

^{13}C NMR (151 MHz, $CDCl_3$) of compound **S14**

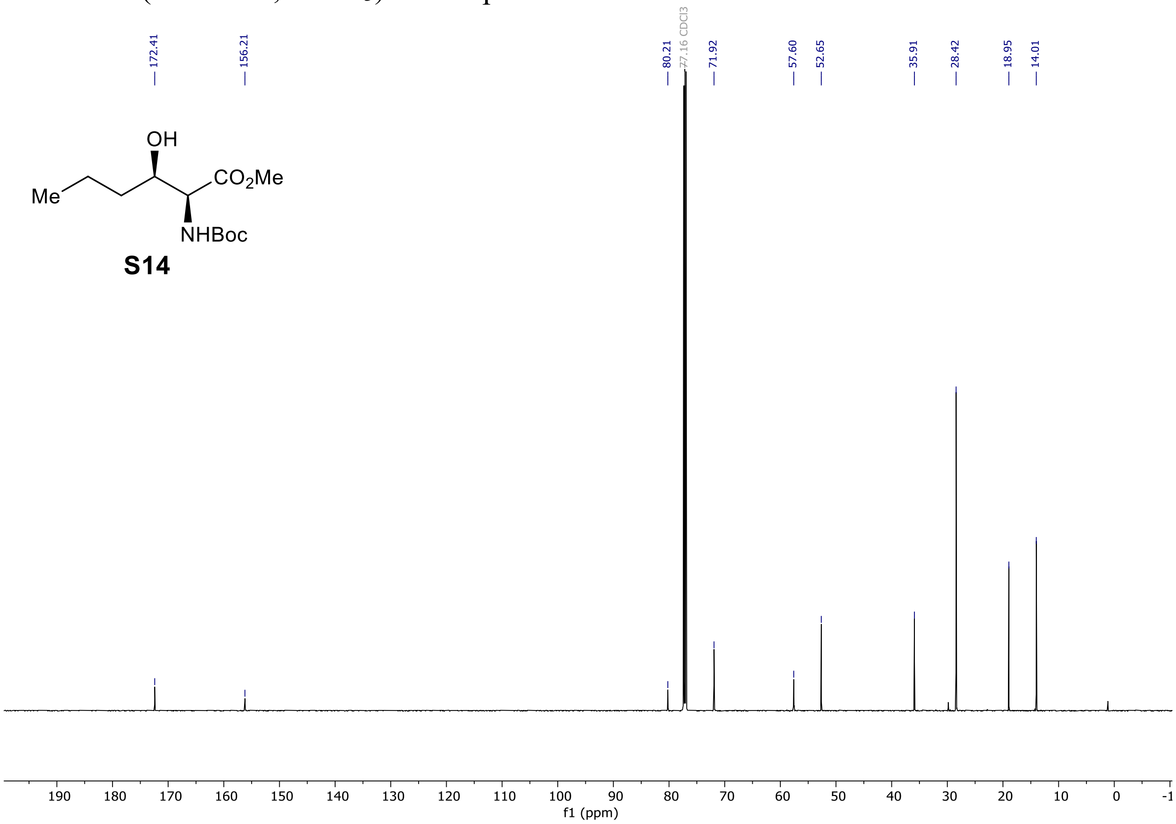

^{1}H NMR (600 MHz, $CDCl_3$) of compound **S16**

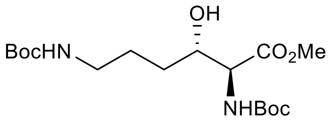

S16

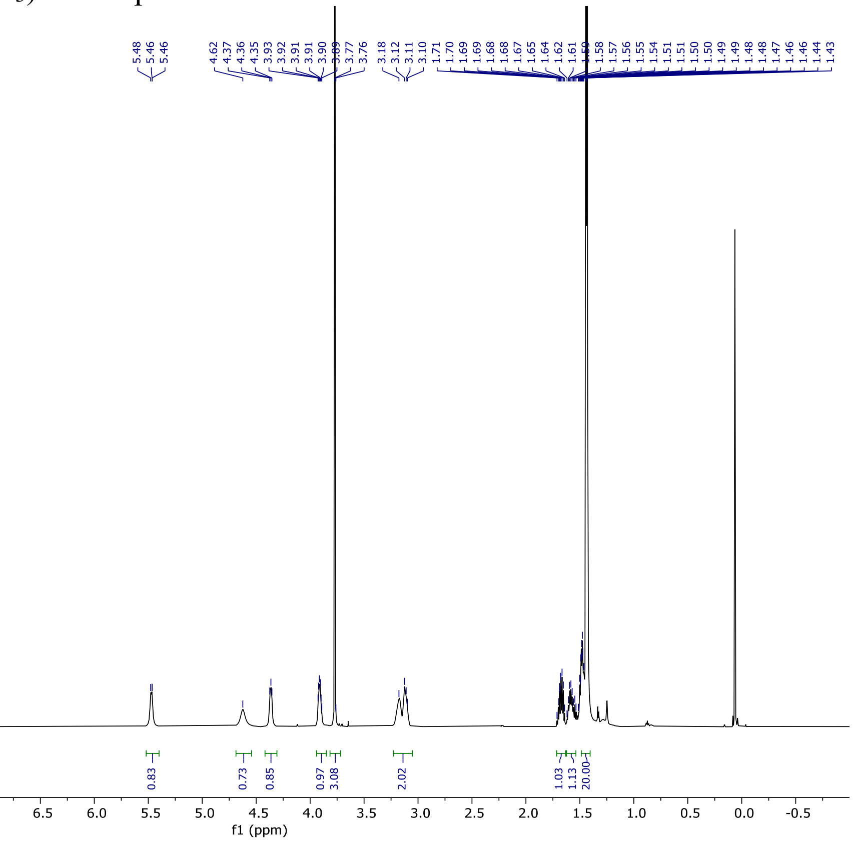

^{13}C NMR (151 MHz, $CDCl_3$) of compound **S16**

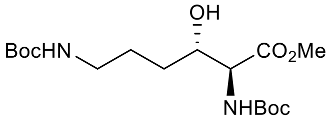

S16

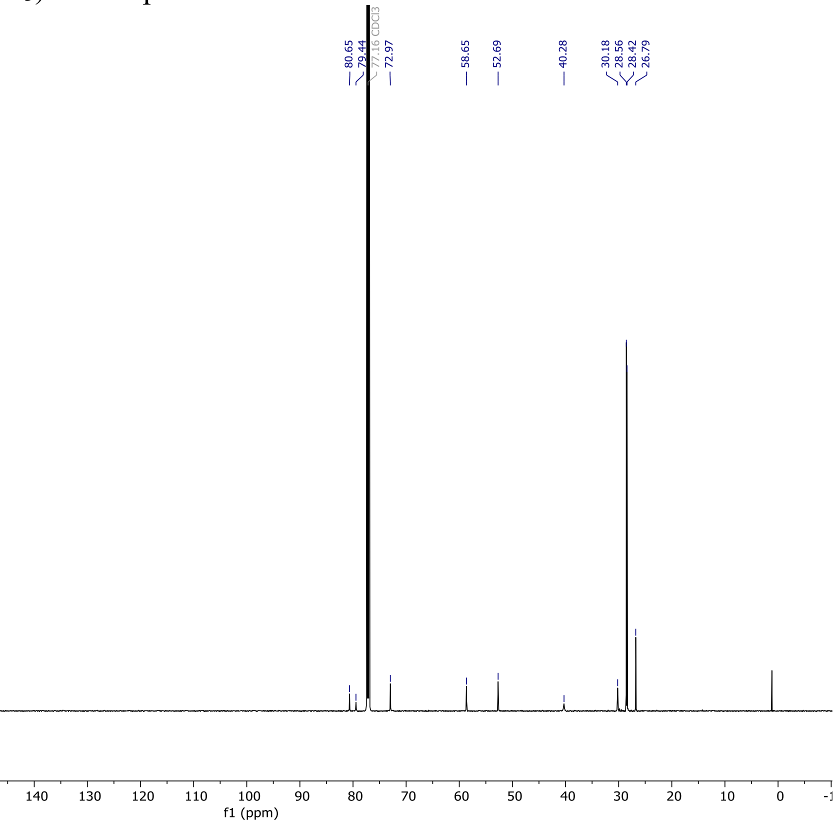

^{1}H NMR (600 MHz, $CDCl_3$) of compound **S18**

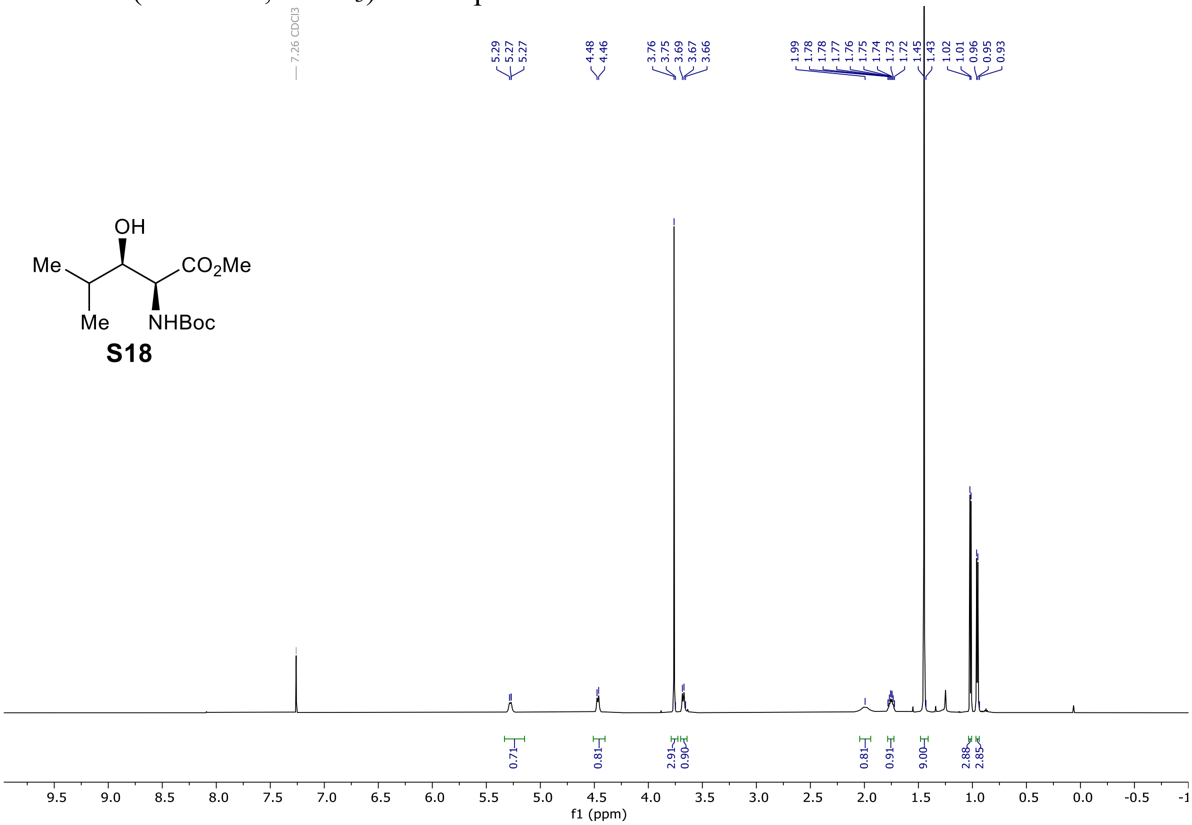

^{13}C NMR (151 MHz, $CDCl_3$) of compound **S18**

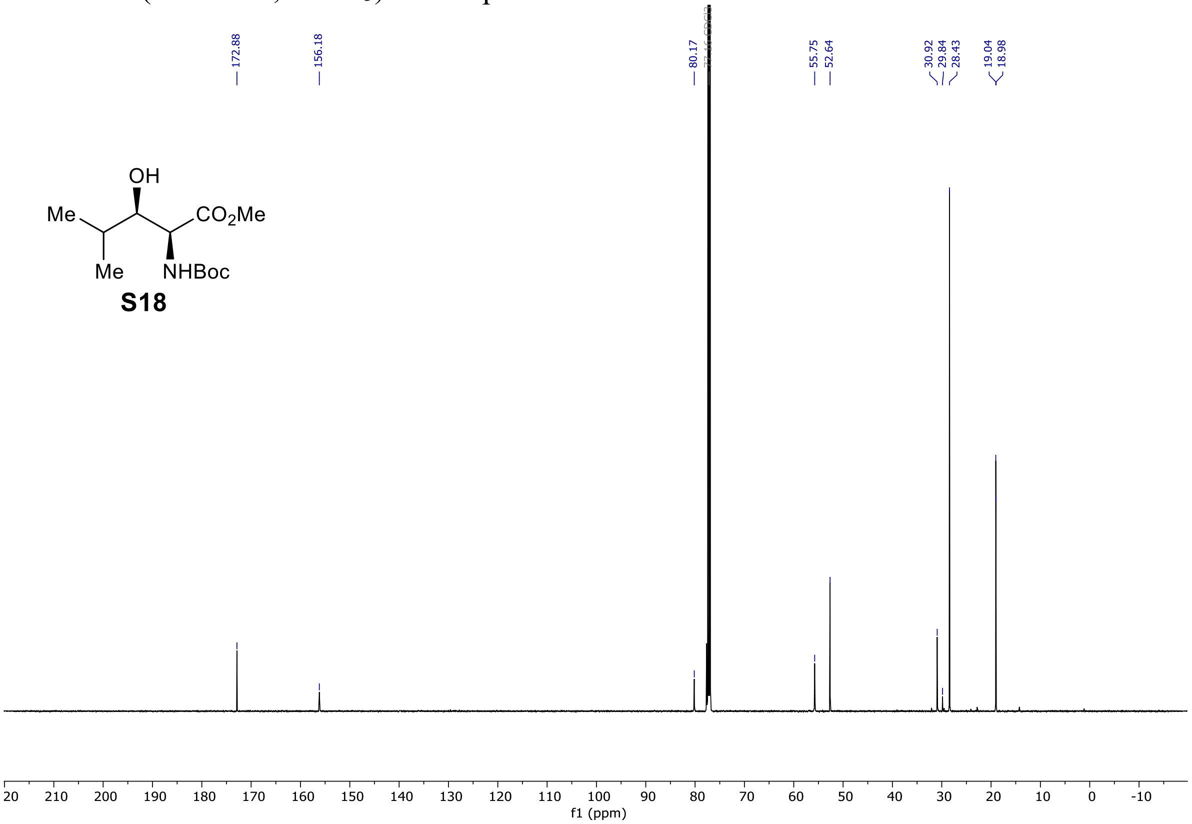

^{1}**H NMR** (400 MHz, $CDCl_3$) of compound **S19**

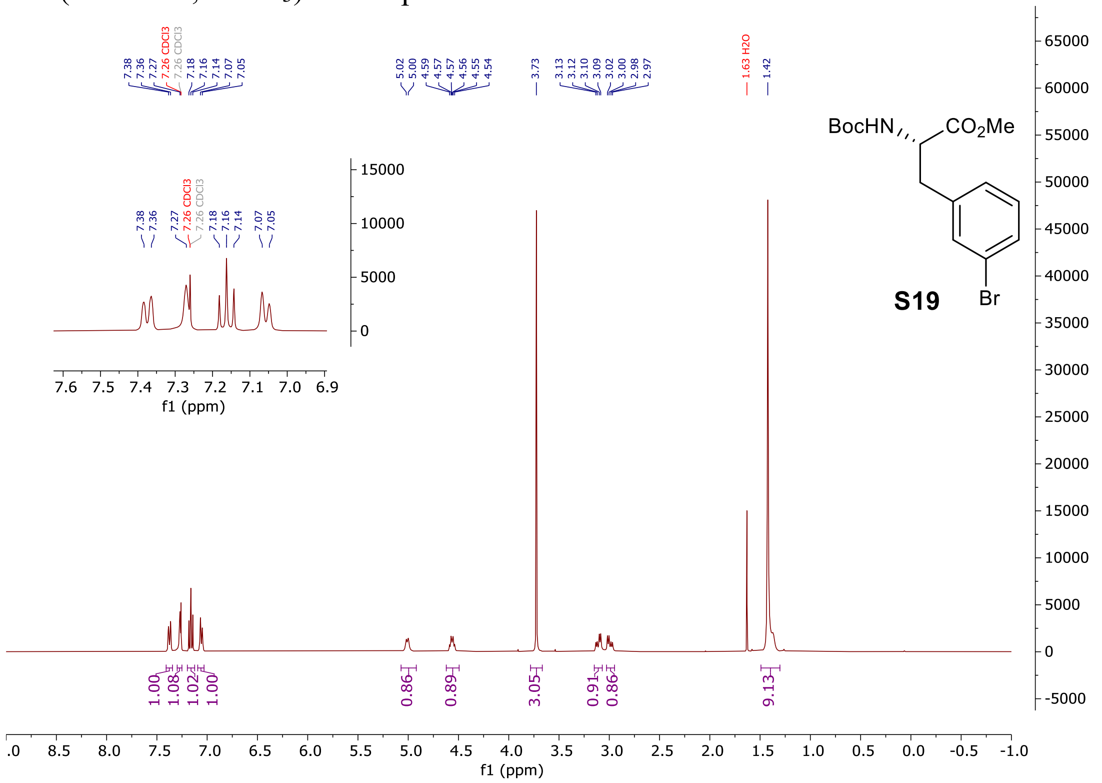

^{13}**C NMR** (101 MHz, $CDCl_3$) of compound **S19**

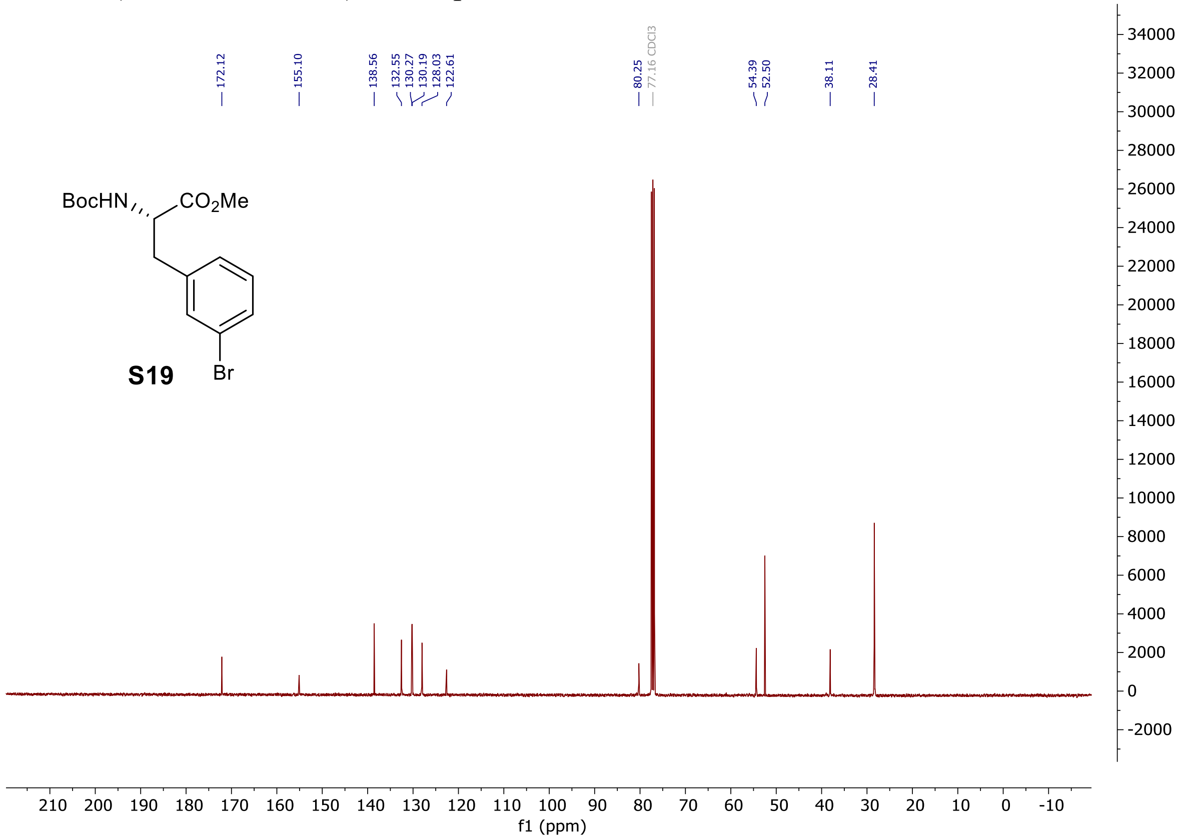

^{1}H NMR (400 MHz, $CDCl_3$) of compound **S20**

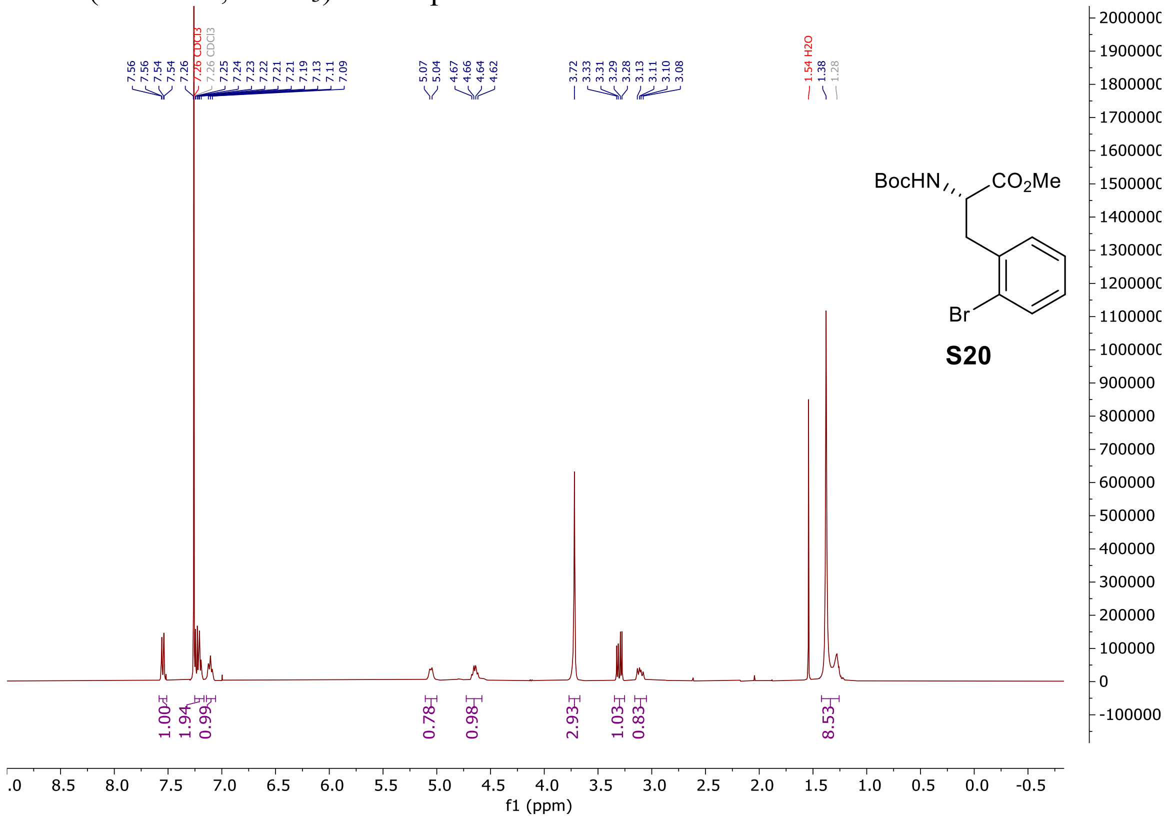

^{1}H NMR (400 MHz, $CDCl_3$) of compound **S21**

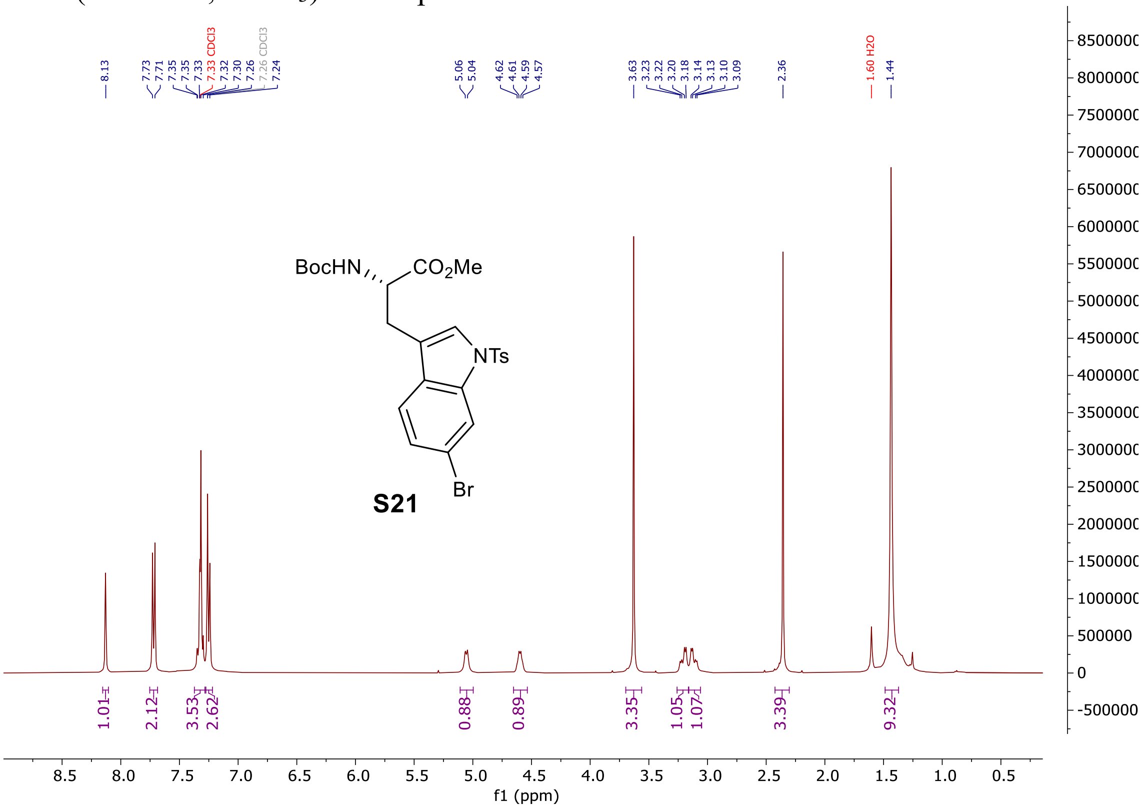

^{13}C NMR (101 MHz, $CDCl_3$) of compound **S21**

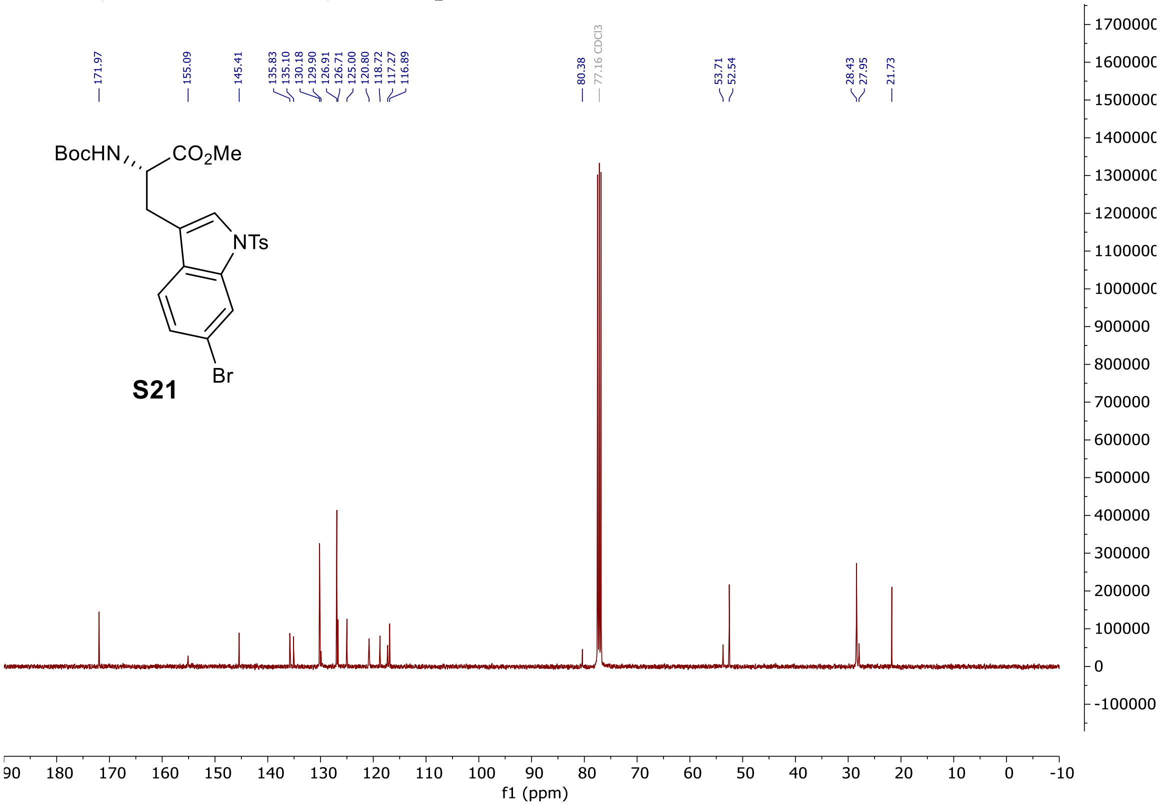

[1]**H NMR** (400 MHz, $CDCl_3$) of compound **S22**

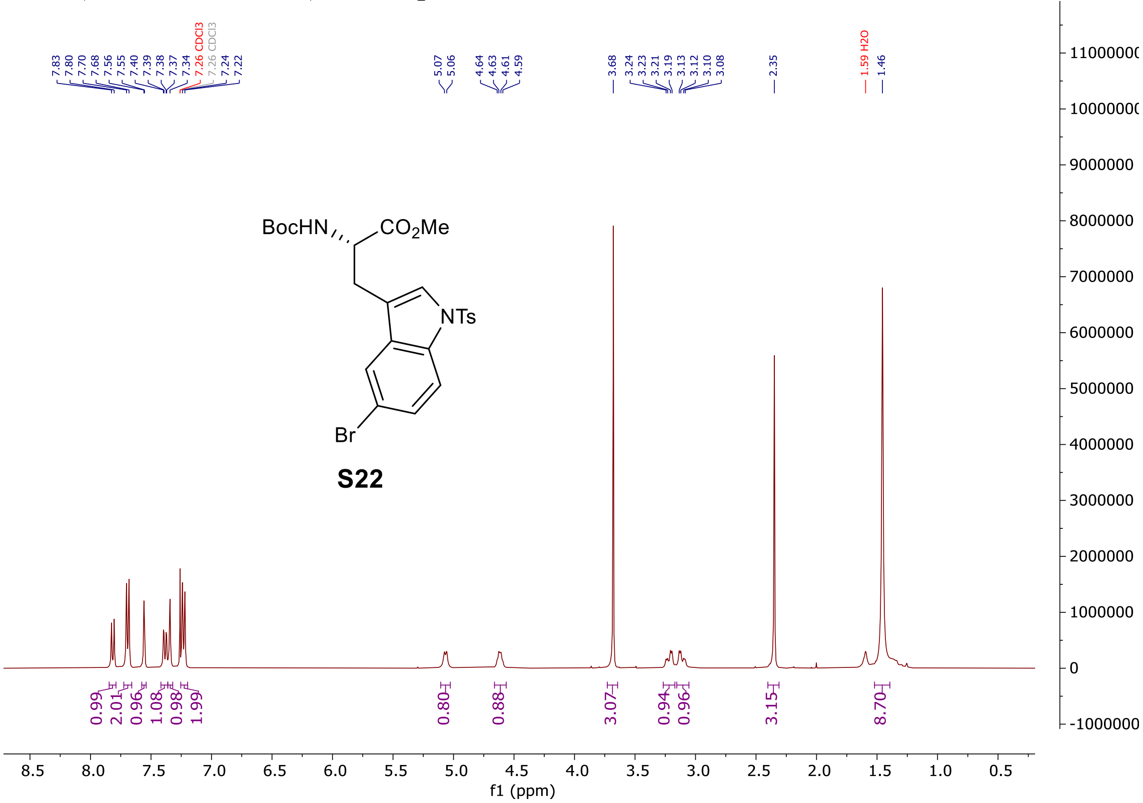

[13]**C NMR** (101 MHz, $CDCl_3$) of compound **S22**

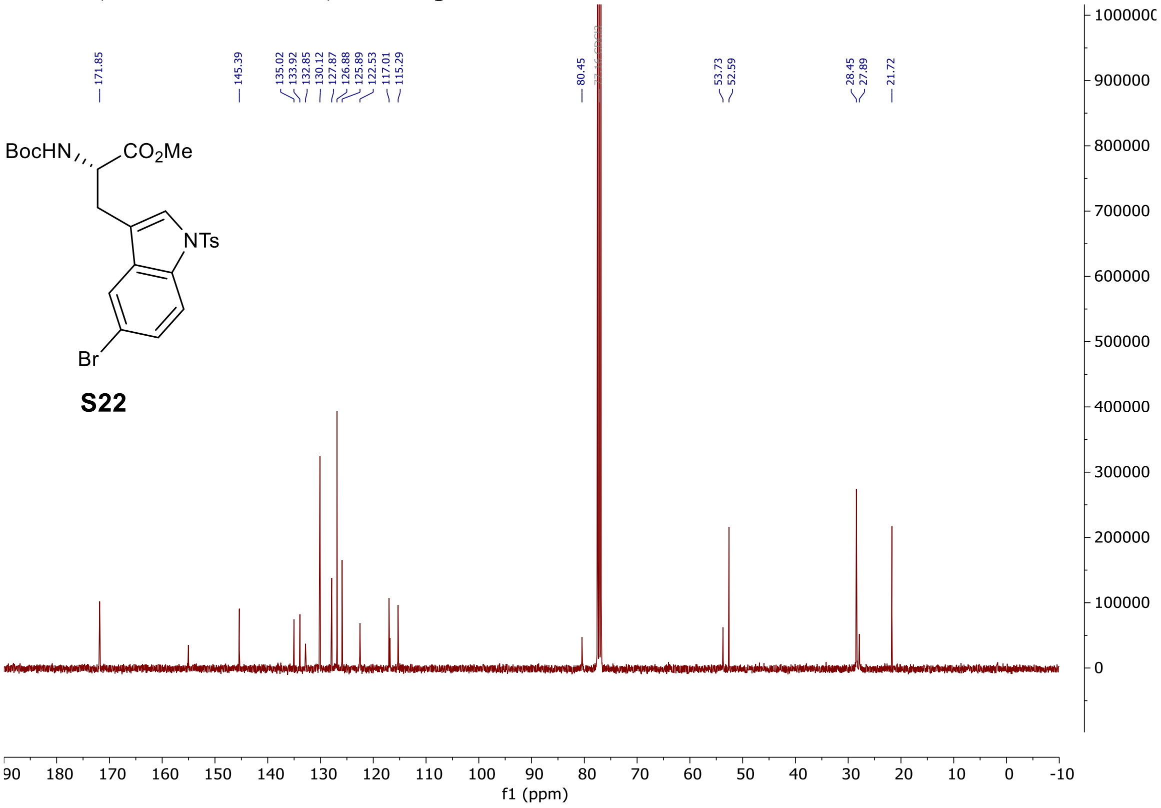

^{1}H NMR (400 MHz, $CDCl_3$) of compound **S23**

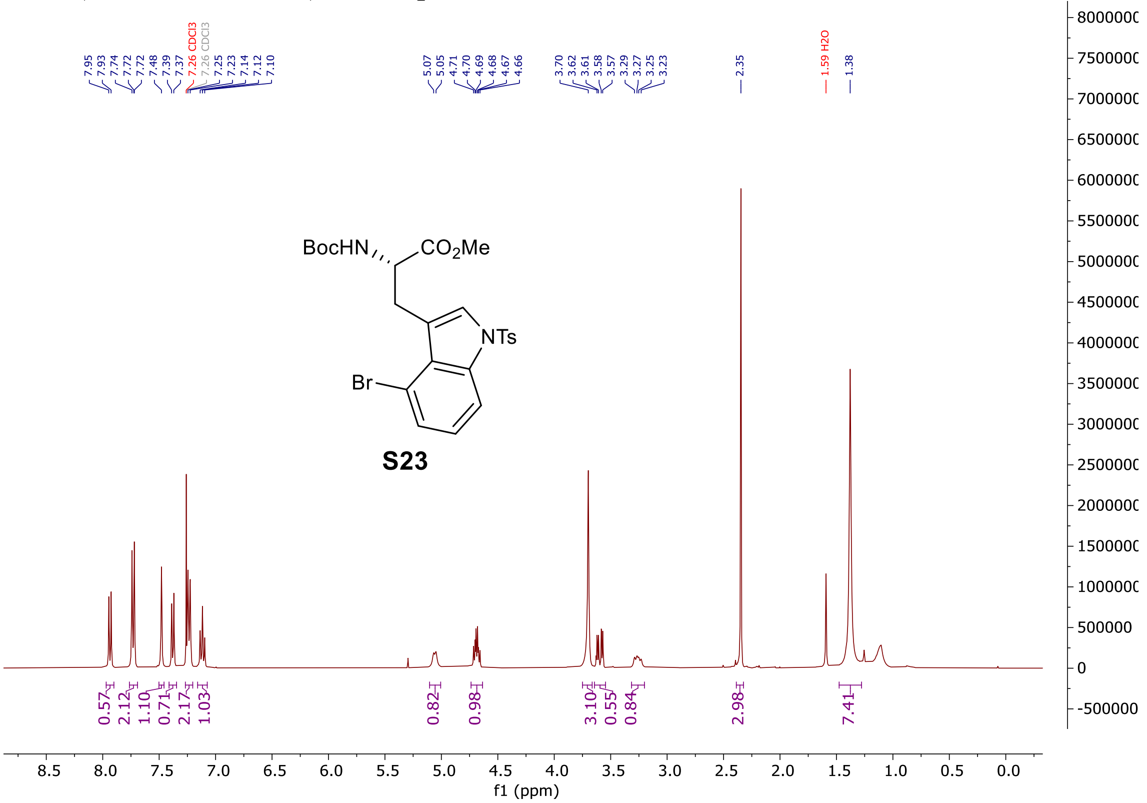

^{13}C NMR (101 MHz, $CDCl_3$) of compound **S23**

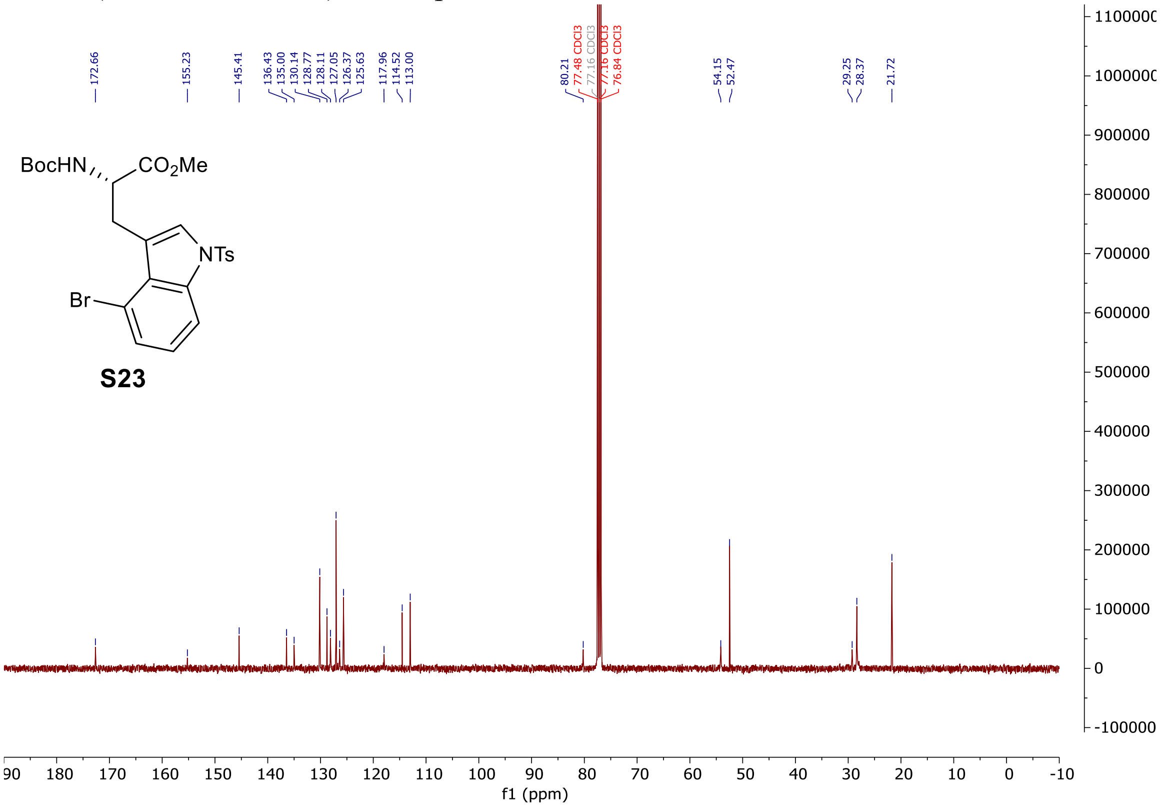

^{1}H NMR (400 MHz, $CDCl_3$) of compound **S24**

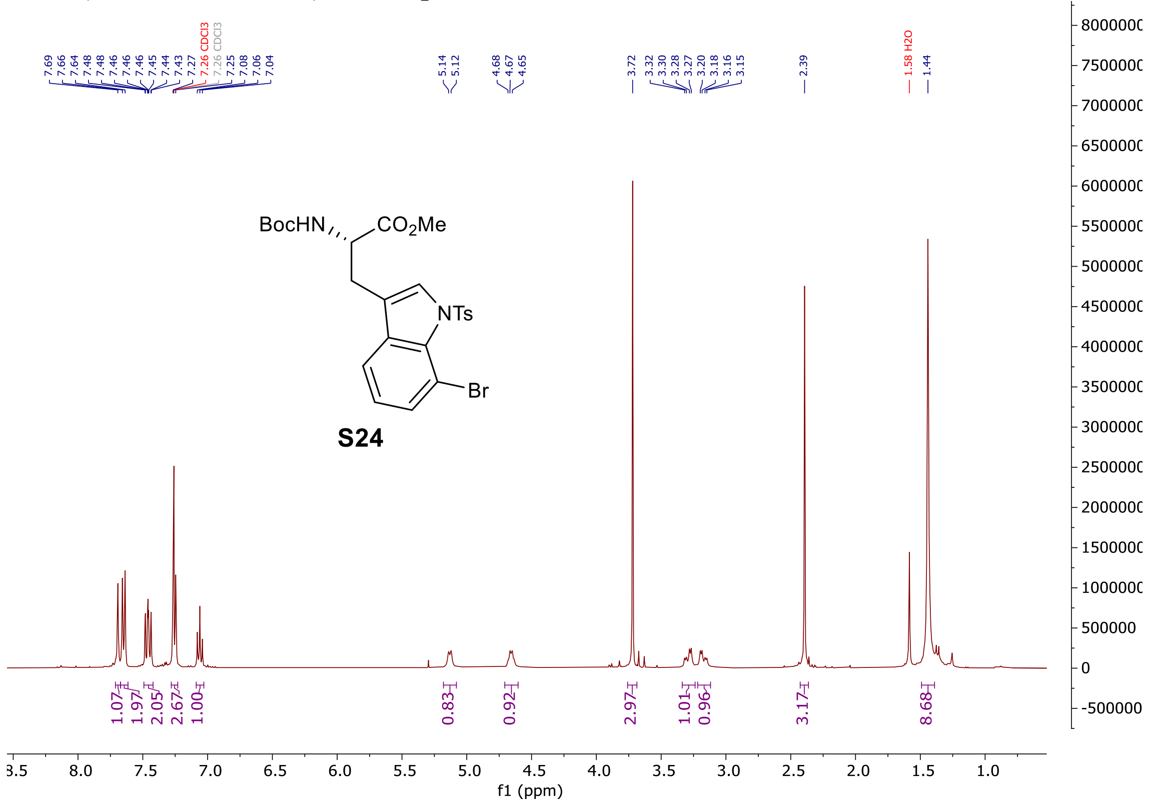

^{13}C NMR (101 MHz, $CDCl_3$) of compound **S24**

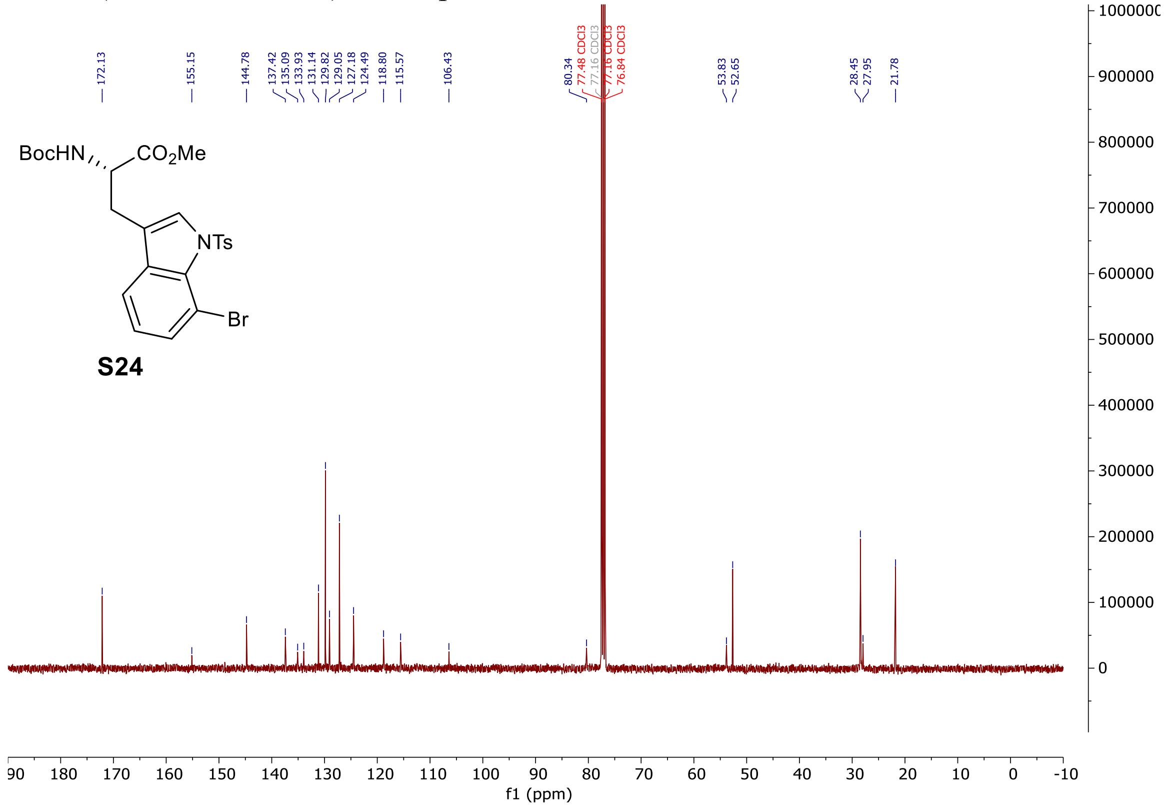

^{1}H NMR (400 MHz, DMSO) of compound **S25**

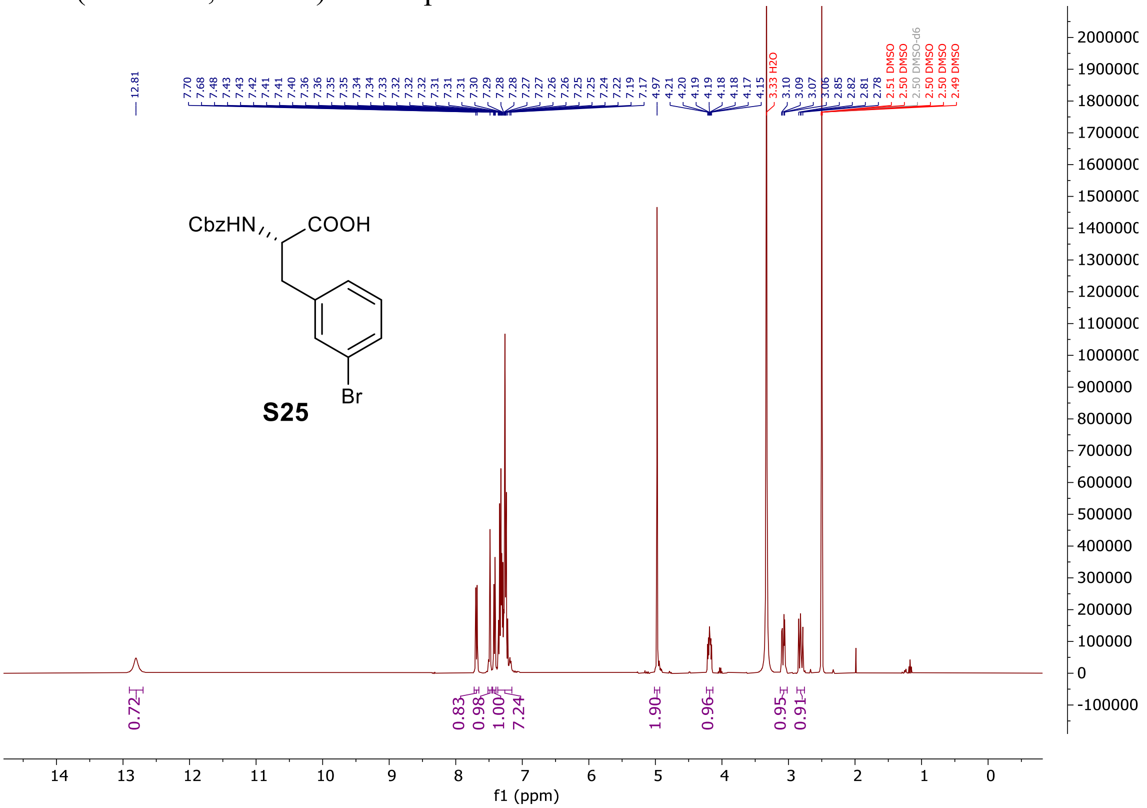

^{13}C NMR (101 MHz, DMSO) of compound **S25**

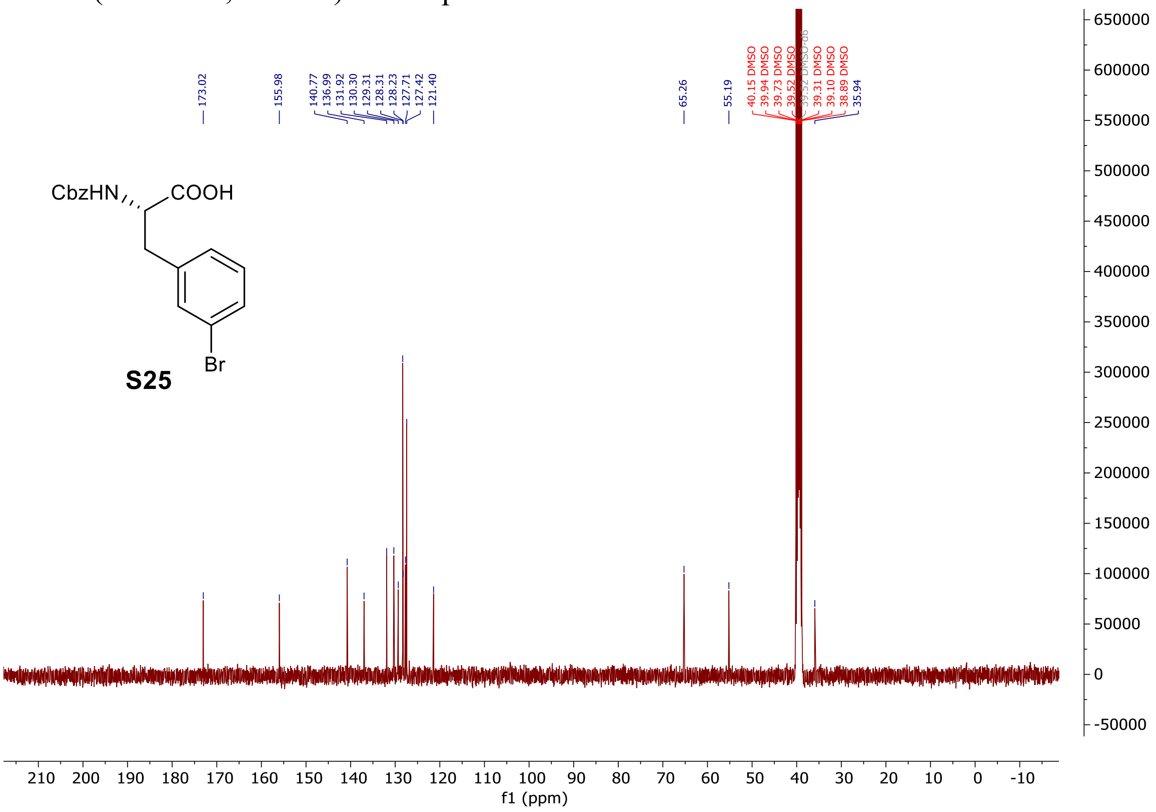

^{1}H NMR (400 MHz, $CDCl_3$) of compound **S26**

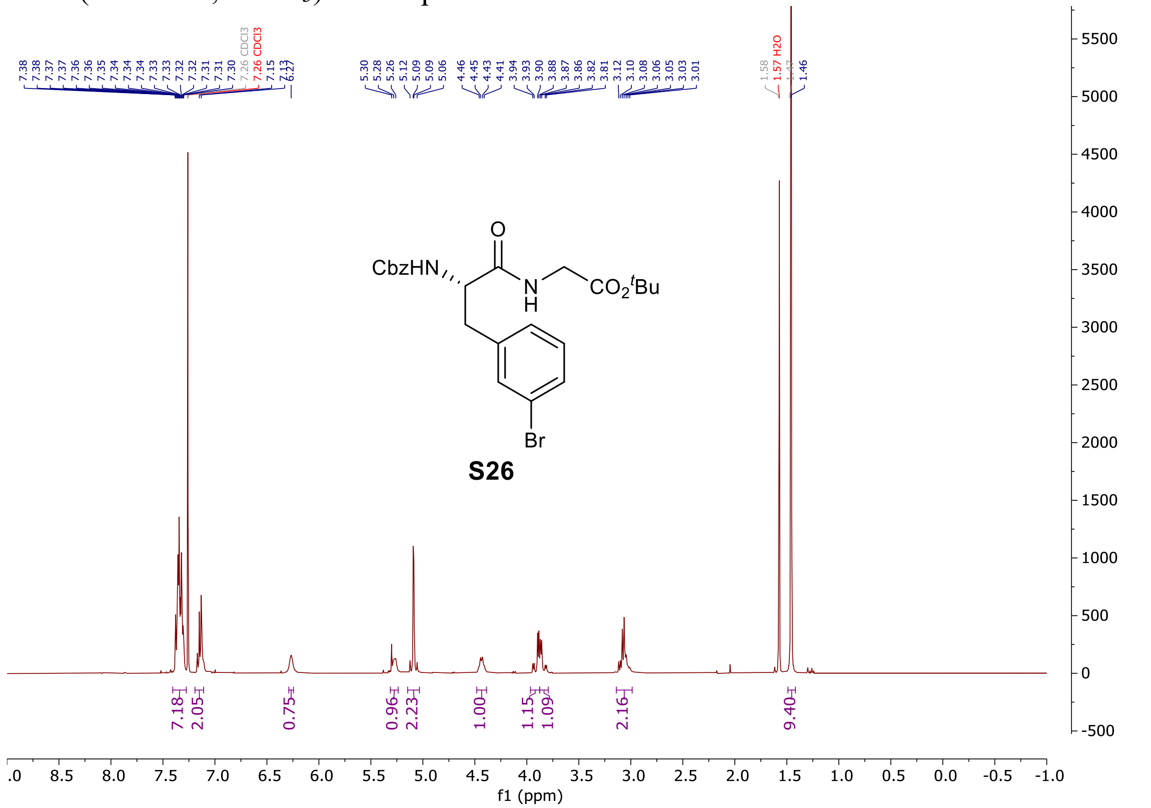

^{13}C NMR (101 MHz, $CDCl_3$) of compound **S26**

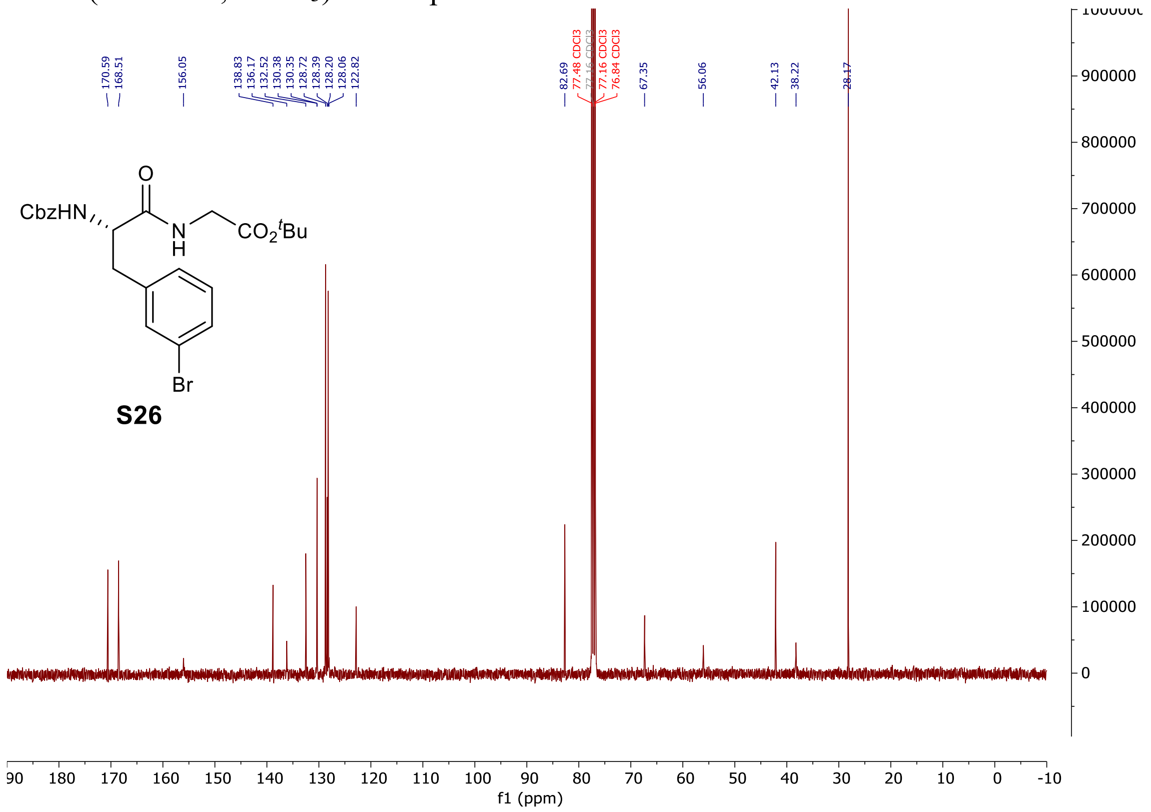

^{1}H NMR (400 MHz, $CDCl_3$) of compound **25**

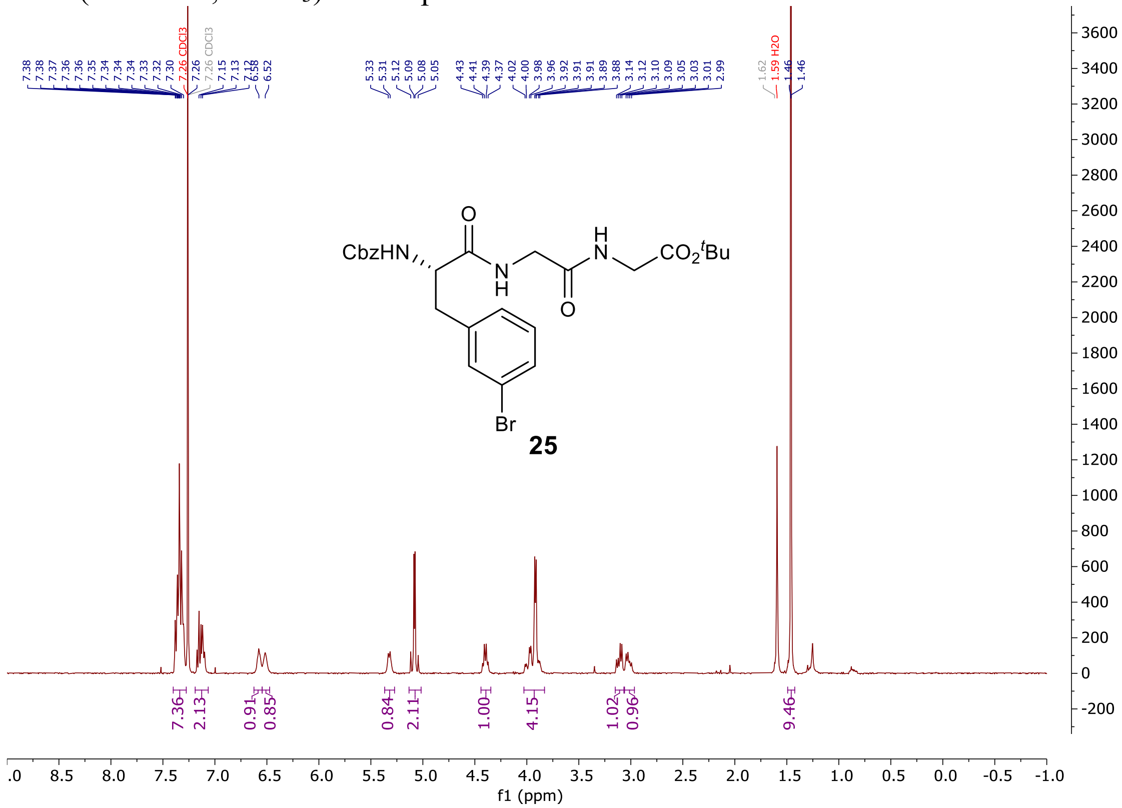

^{13}C NMR (101 MHz, $CDCl_3$) of compound **25**

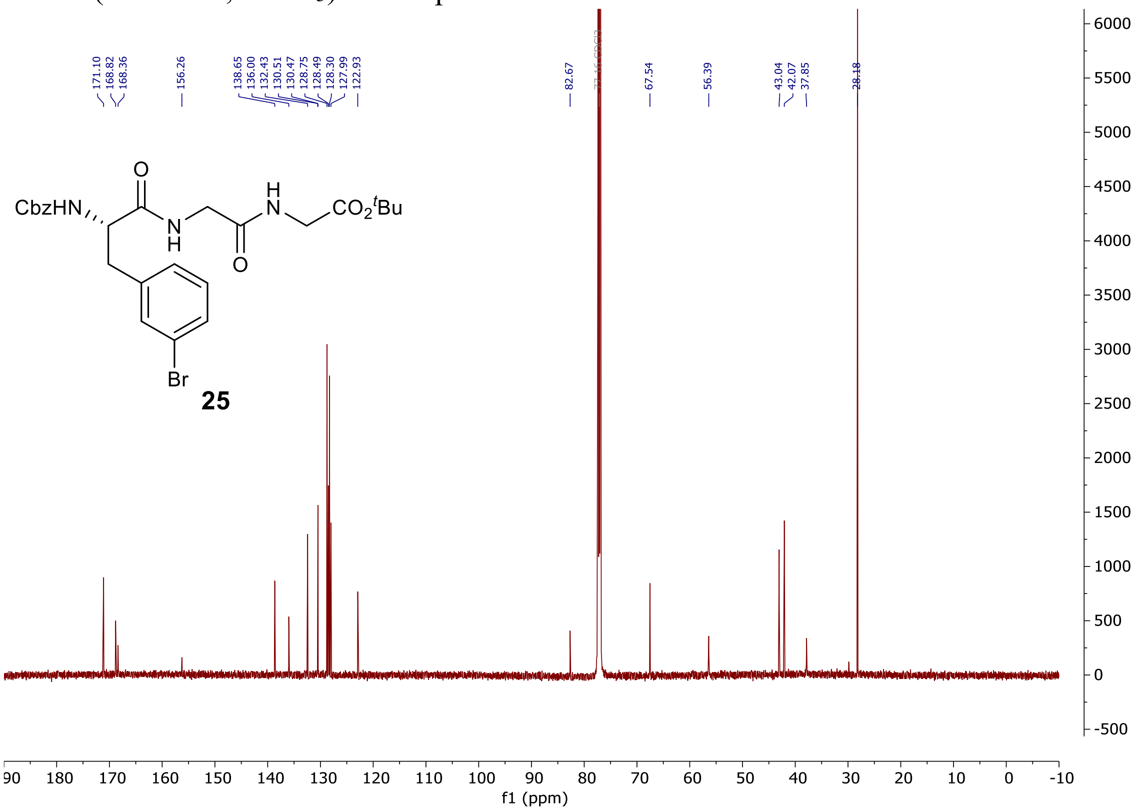

^{1}H NMR (800 MHz, $CDCl_3$) of compound **S27**

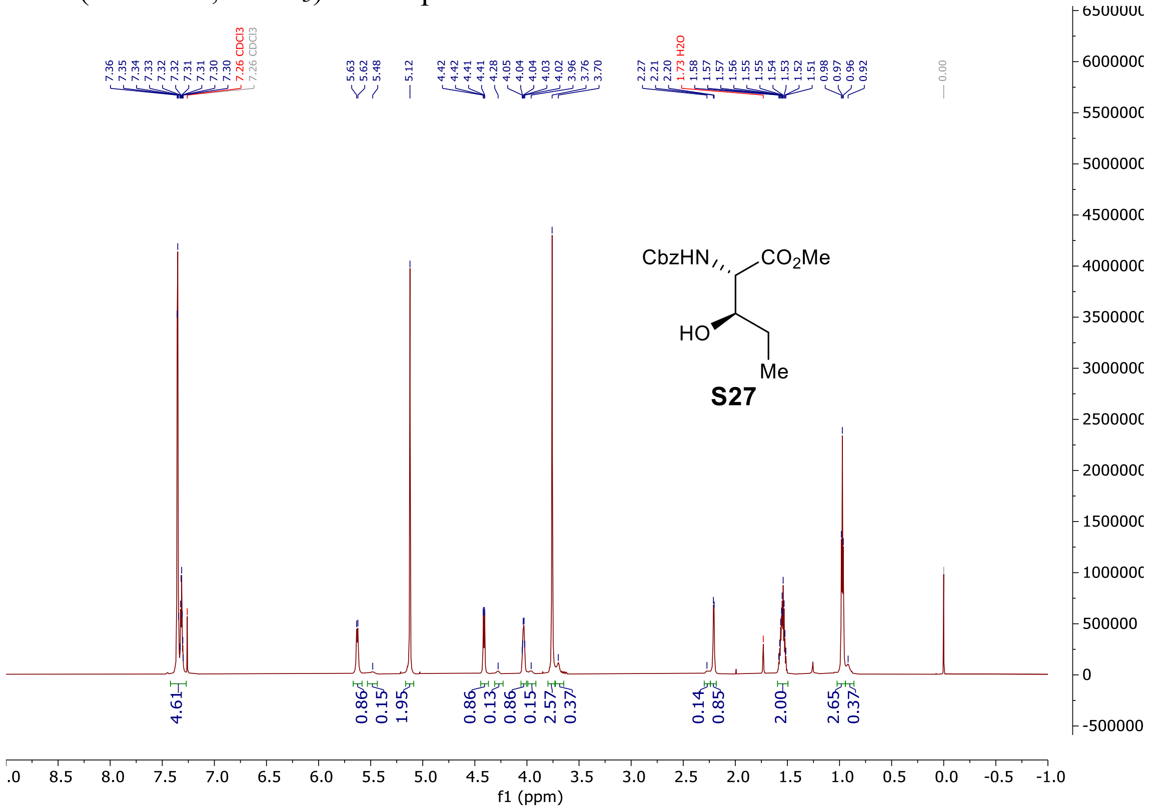

^{13}C NMR (201 MHz, $CDCl_3$) of compound **S27**

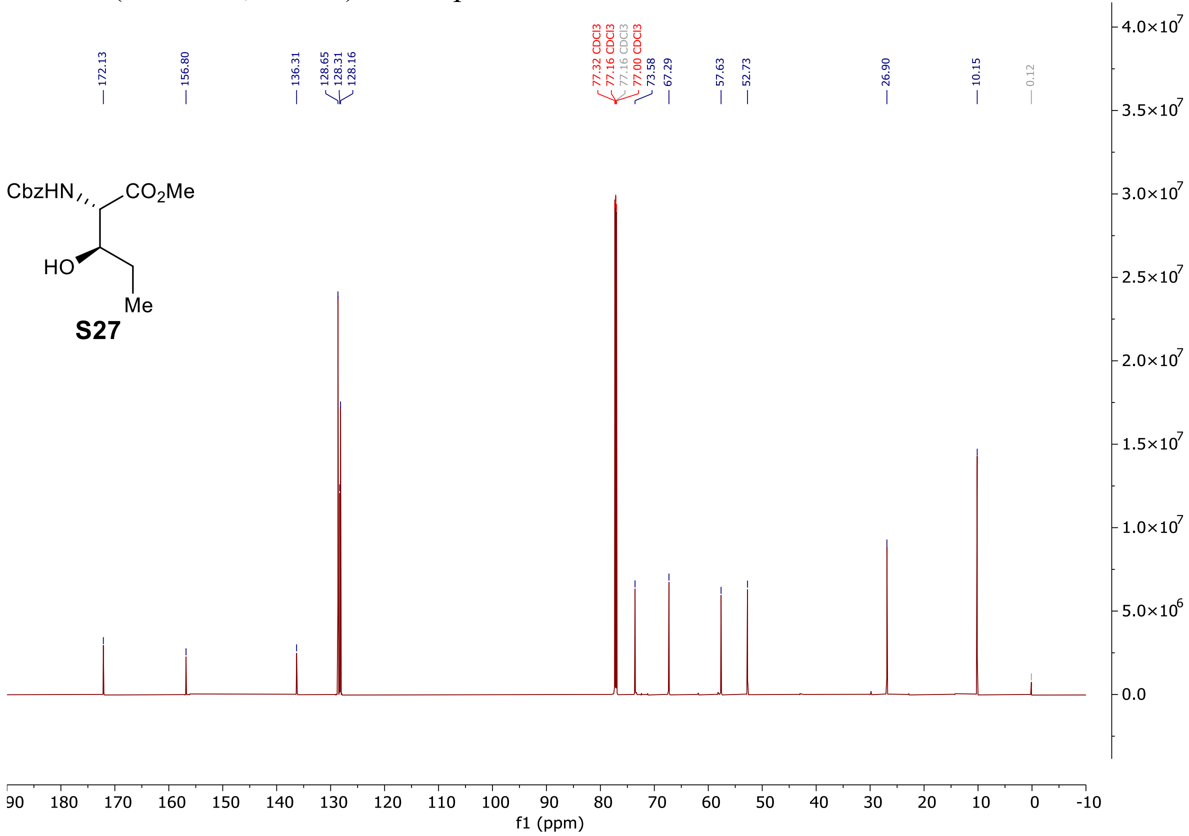

^{1}H NMR (400 MHz, $CDCl_3$) of compound **S28**

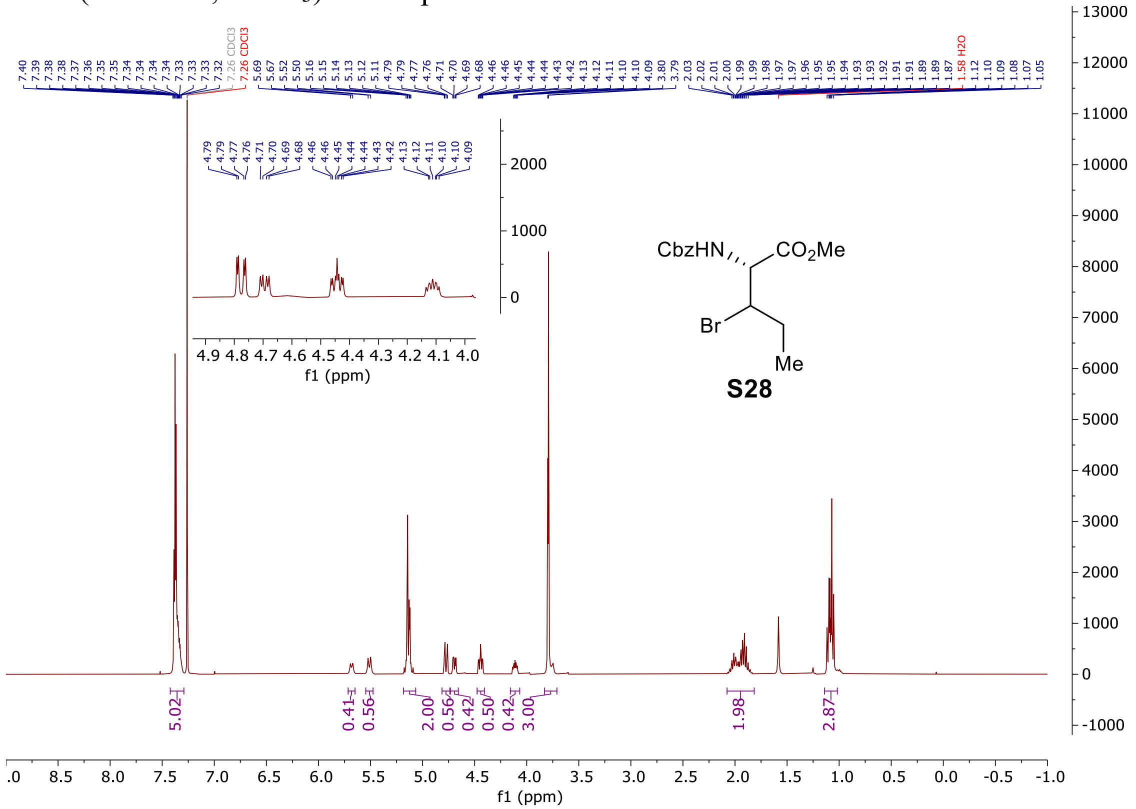

^{13}C NMR (101 MHz, $CDCl_3$) of compound **S28**

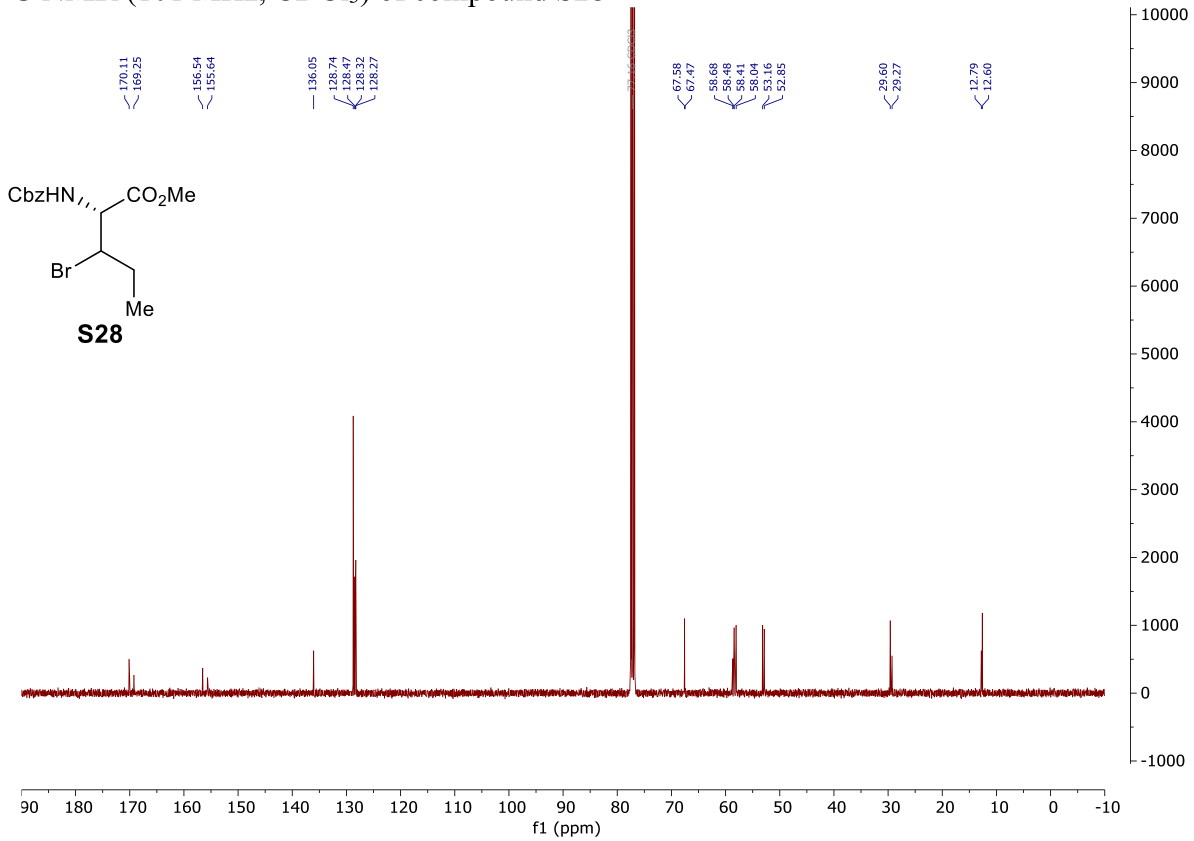

^{1}H NMR (400 MHz, $CDCl_3$) of compound **S29**

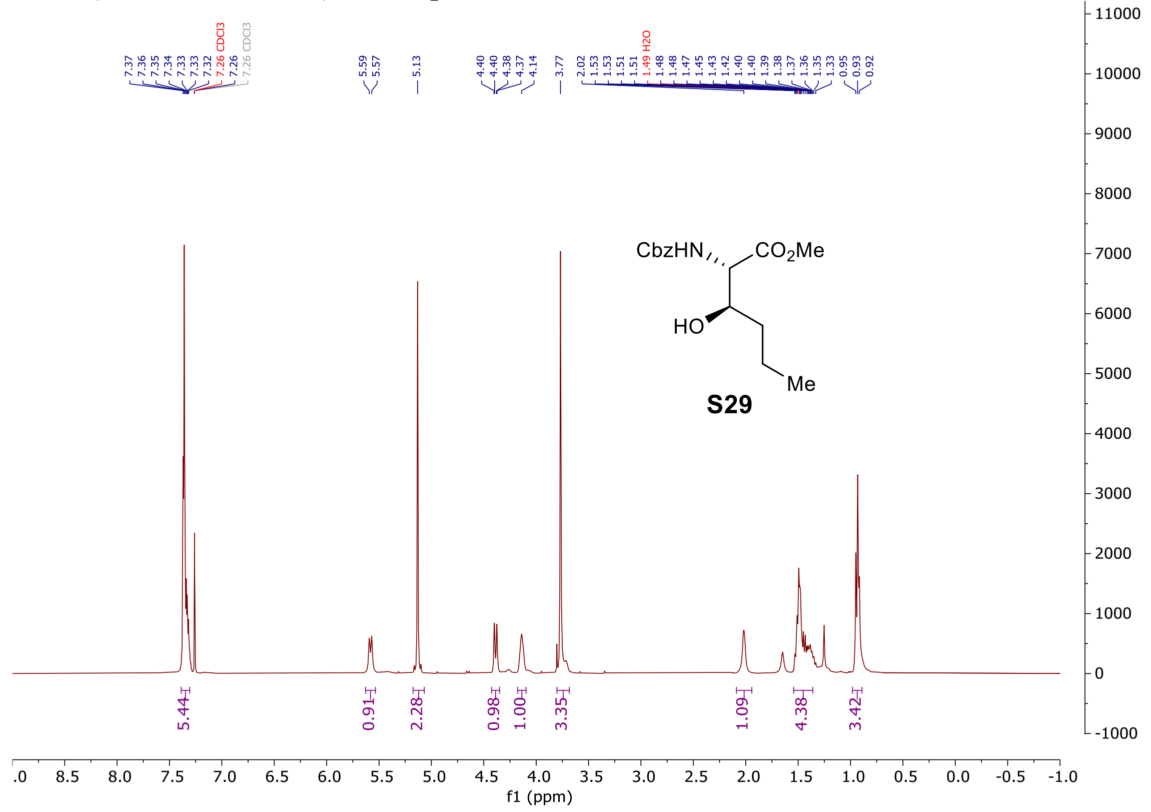

^{13}C NMR (101 MHz, $CDCl_3$) of compound **S29**

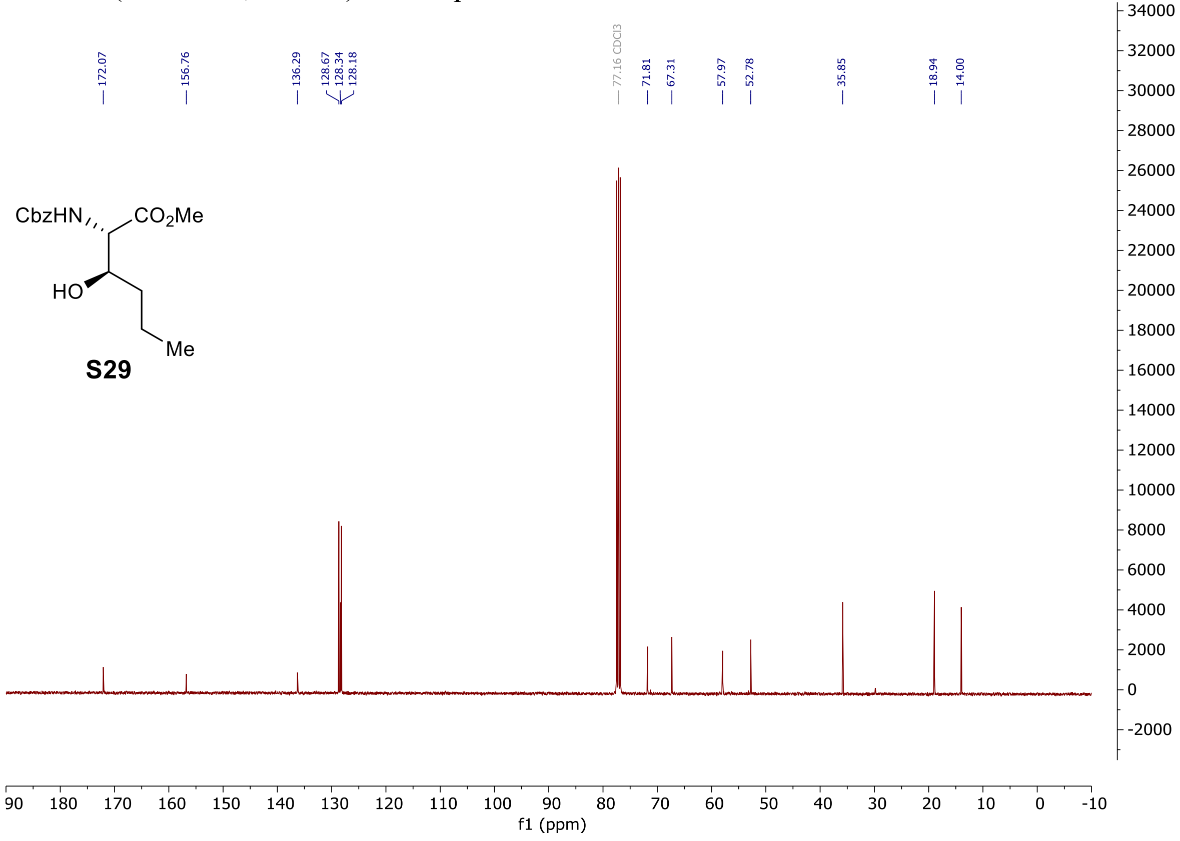

^{1}H NMR (400 MHz, $CDCl_3$) of compound **S30**

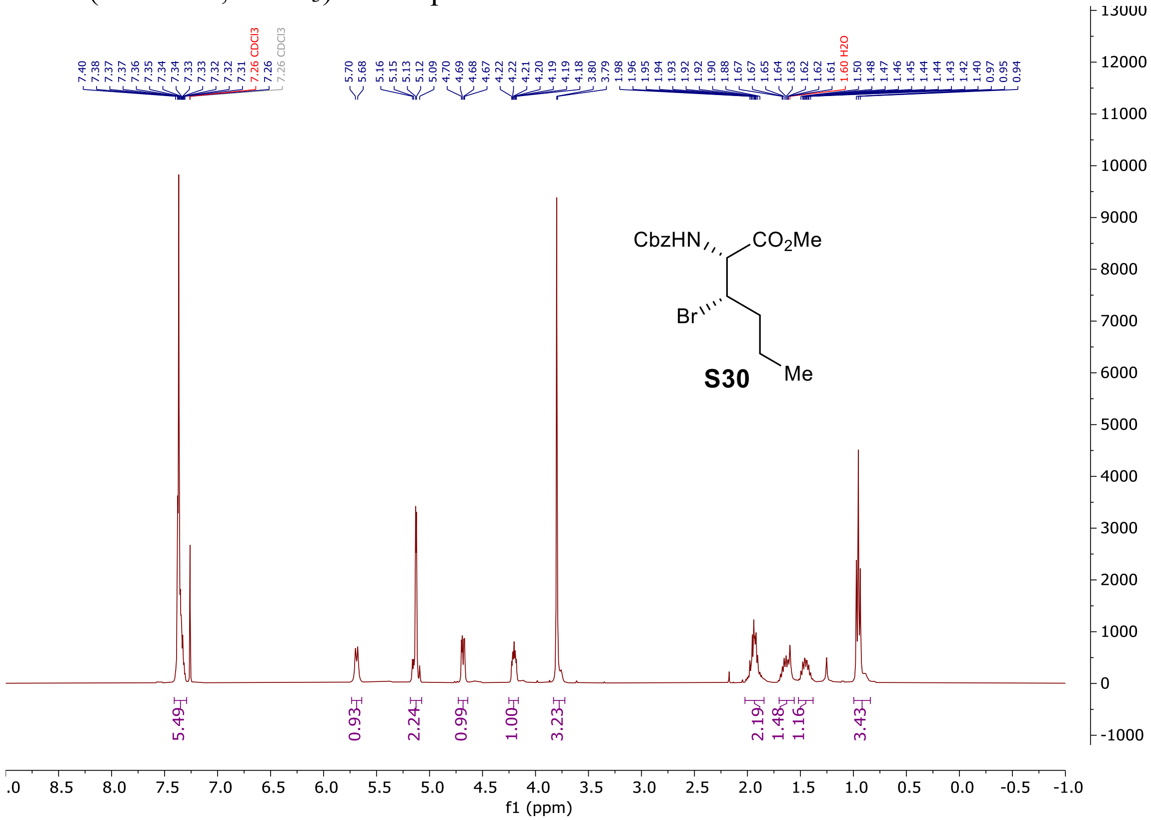

^{13}C NMR (101 MHz, $CDCl_3$) of compound **S30**

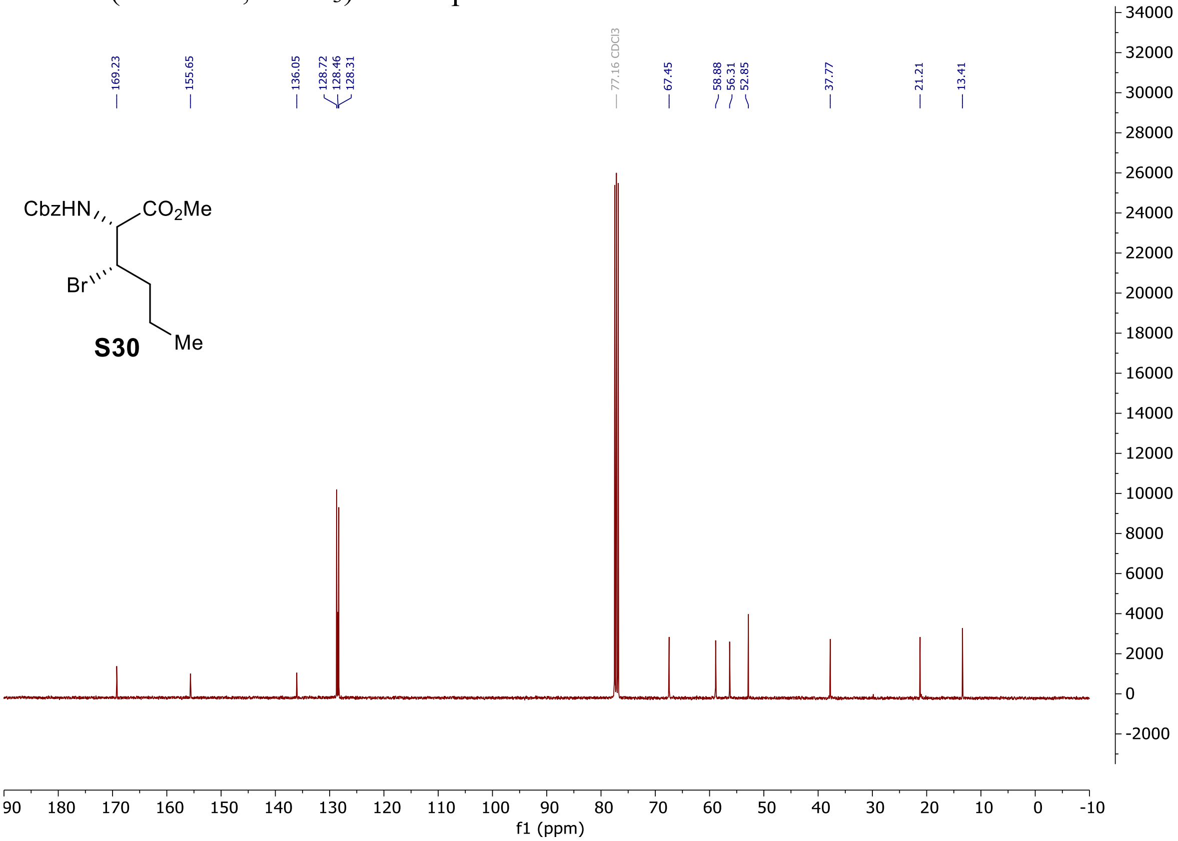

^{1}H NMR (400 MHz, $CDCl_3$) of compound **S31**

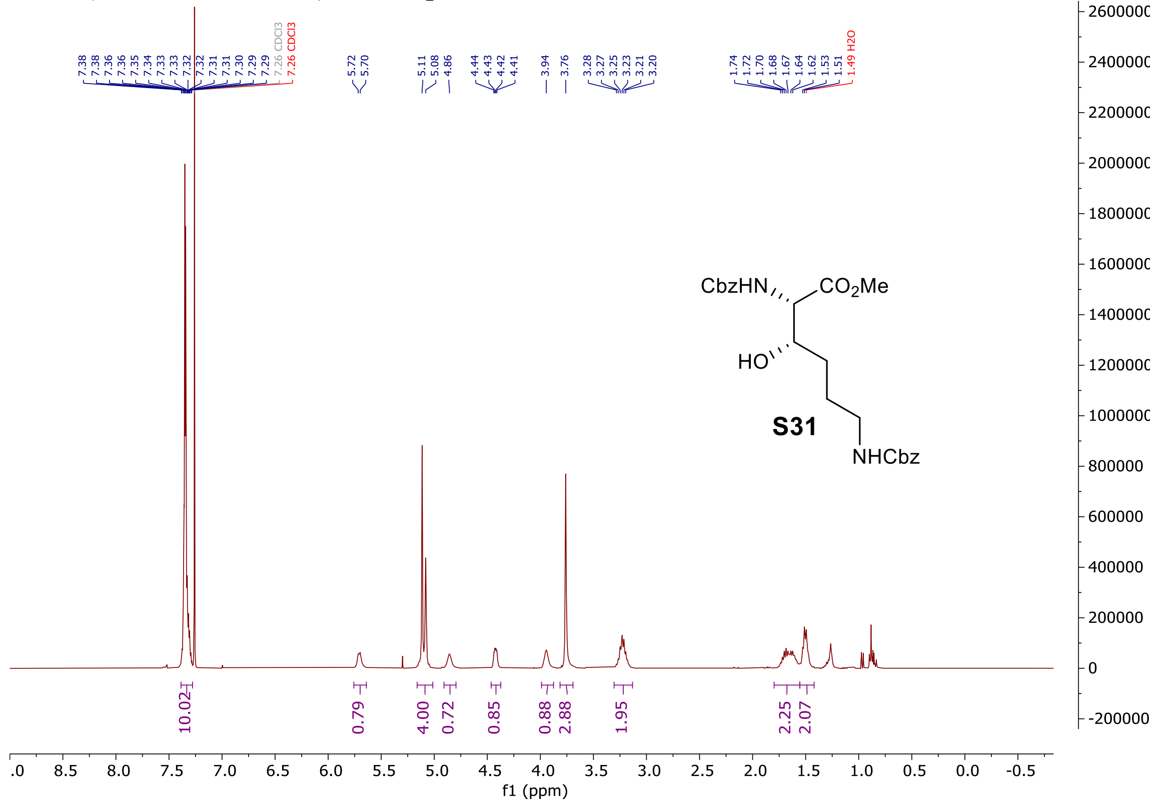

^{13}C NMR (101 MHz, $CDCl_3$) of compound **S31**

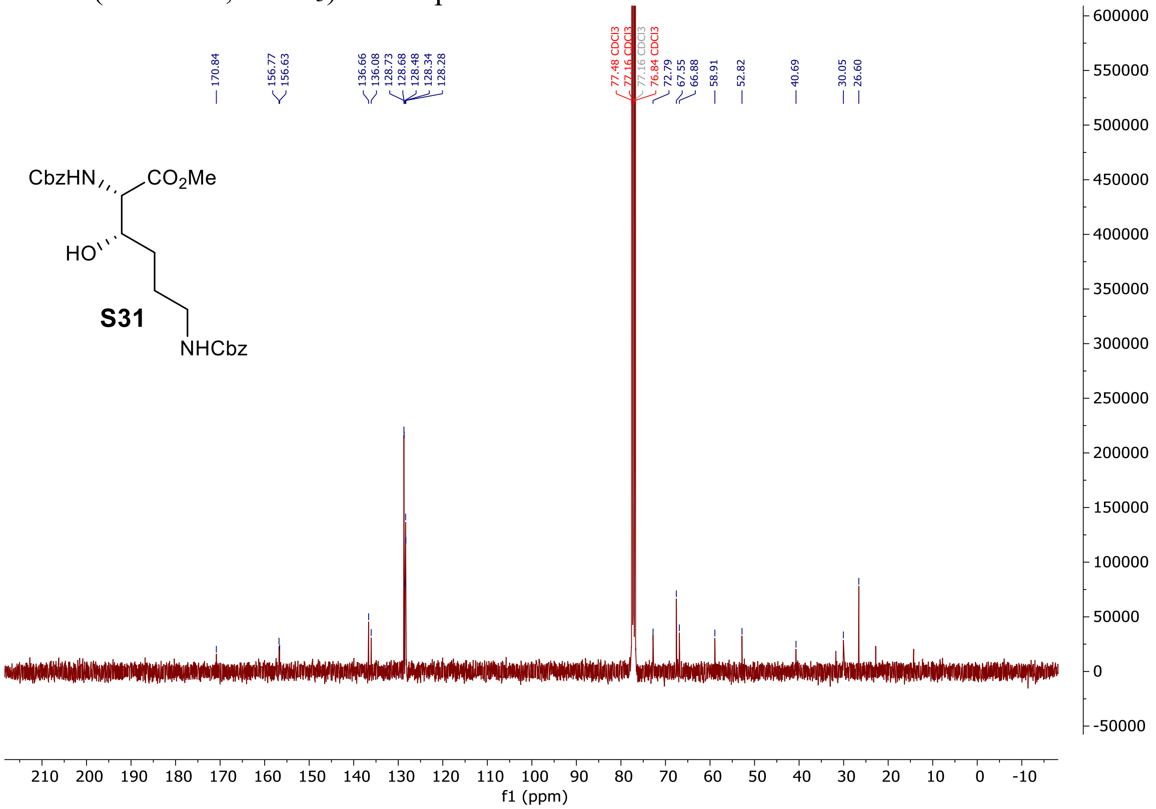

^{1}H NMR (400 MHz, $CDCl_3$) of compound **S32**

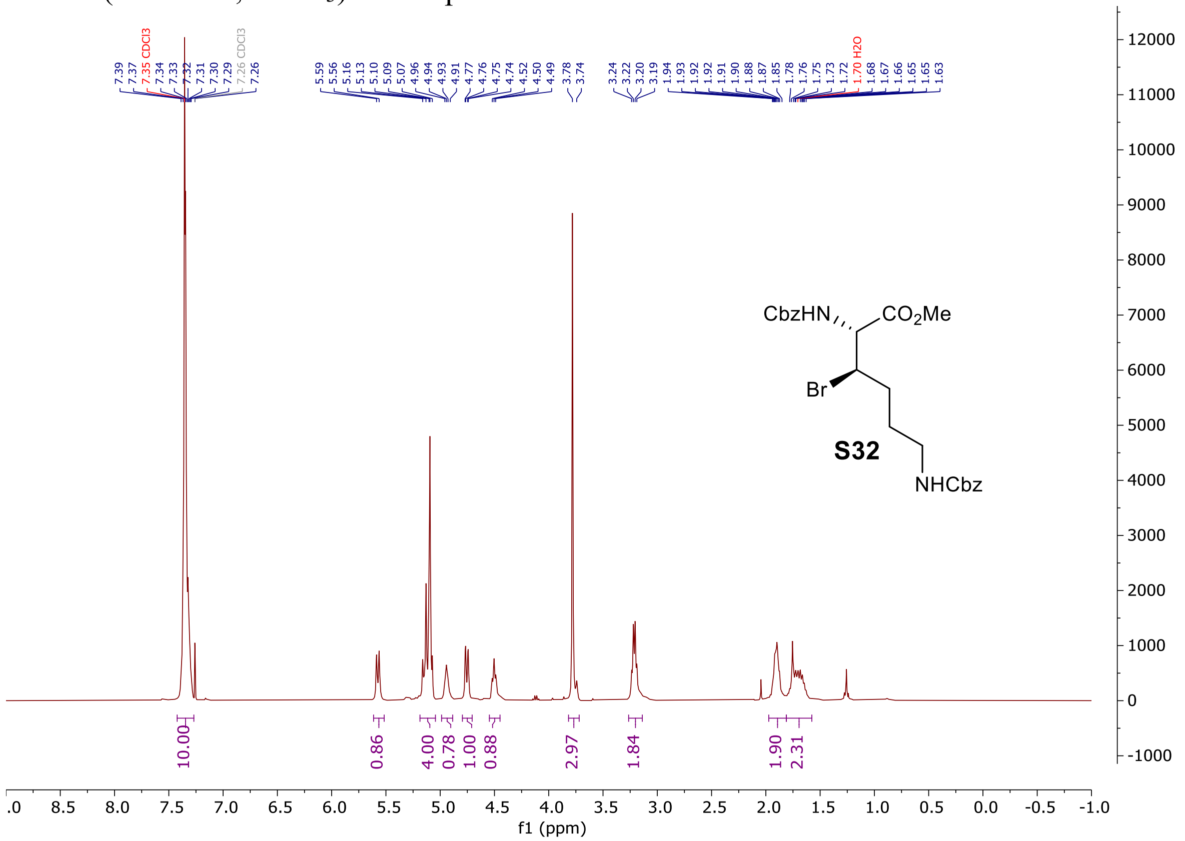

^{13}C NMR (101 MHz, $CDCl_3$) of compound **S32**

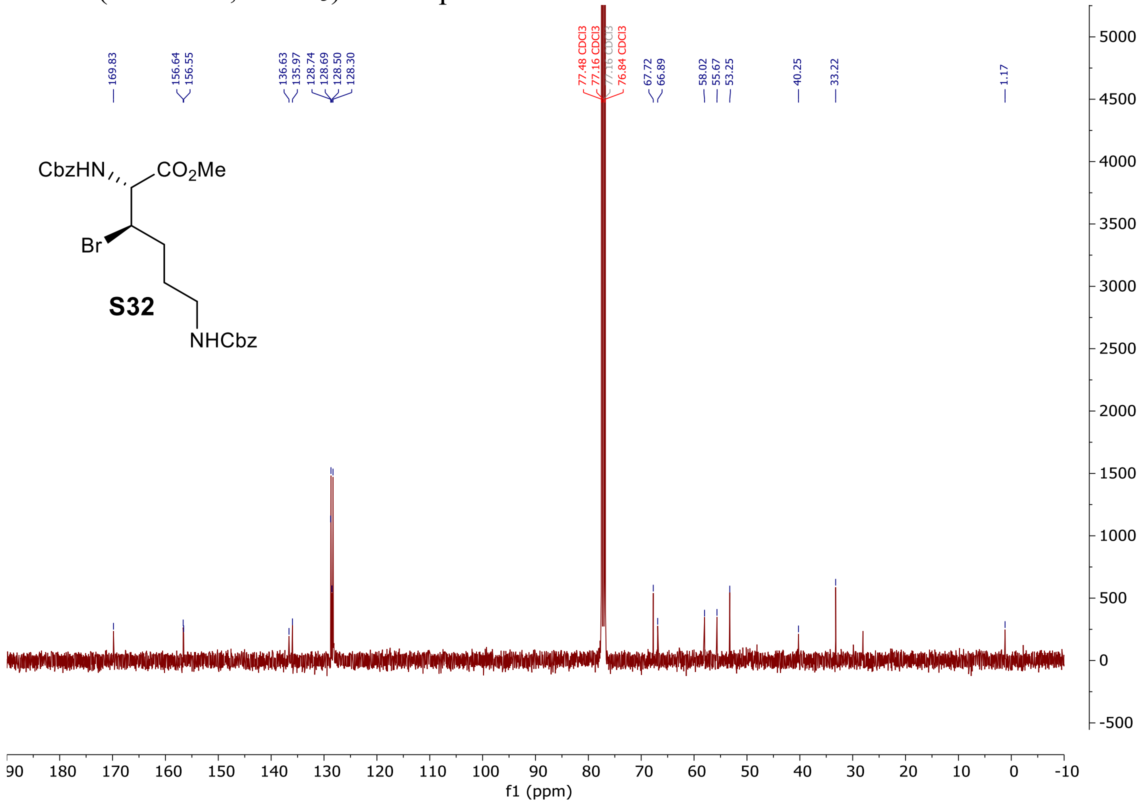

^{1}**H NMR** (400 MHz, $CDCl_3$) of compound **S35**

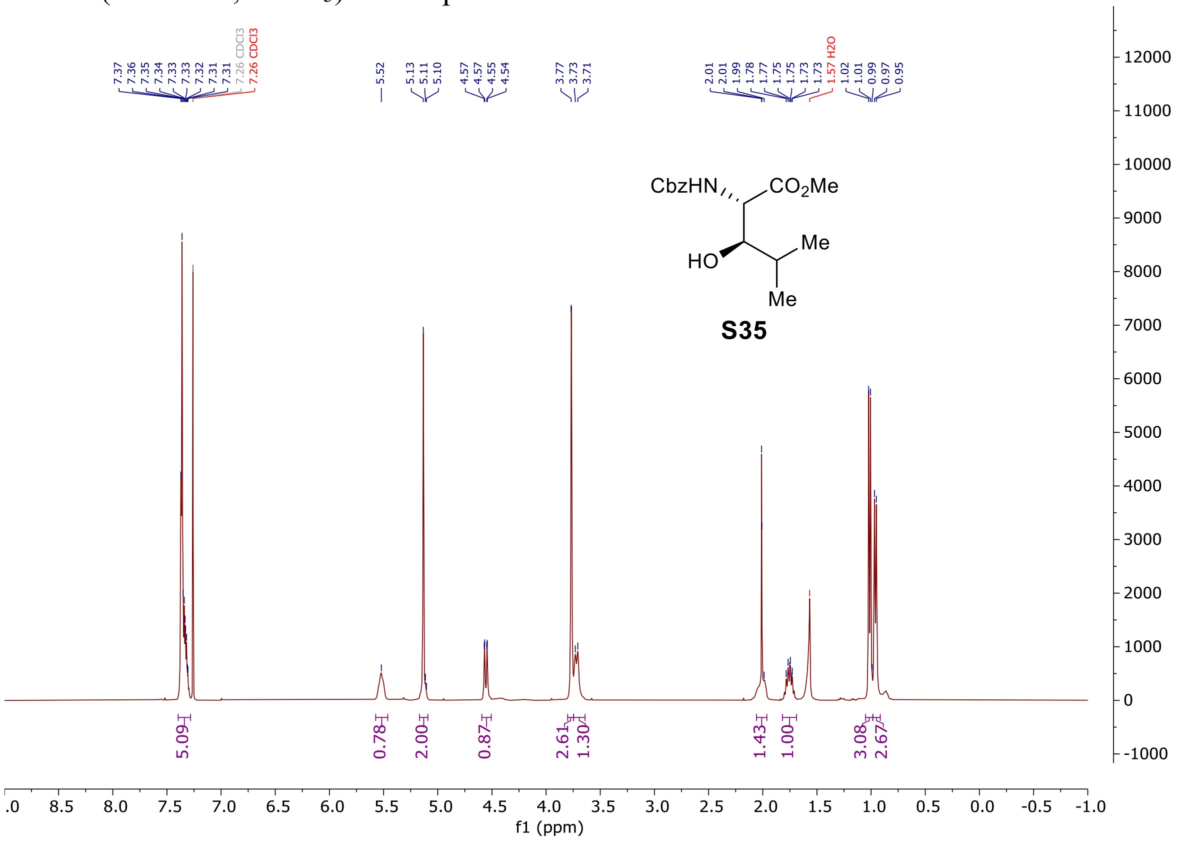

^{13}**C NMR** (101 MHz, $CDCl_3$) of compound **S35**

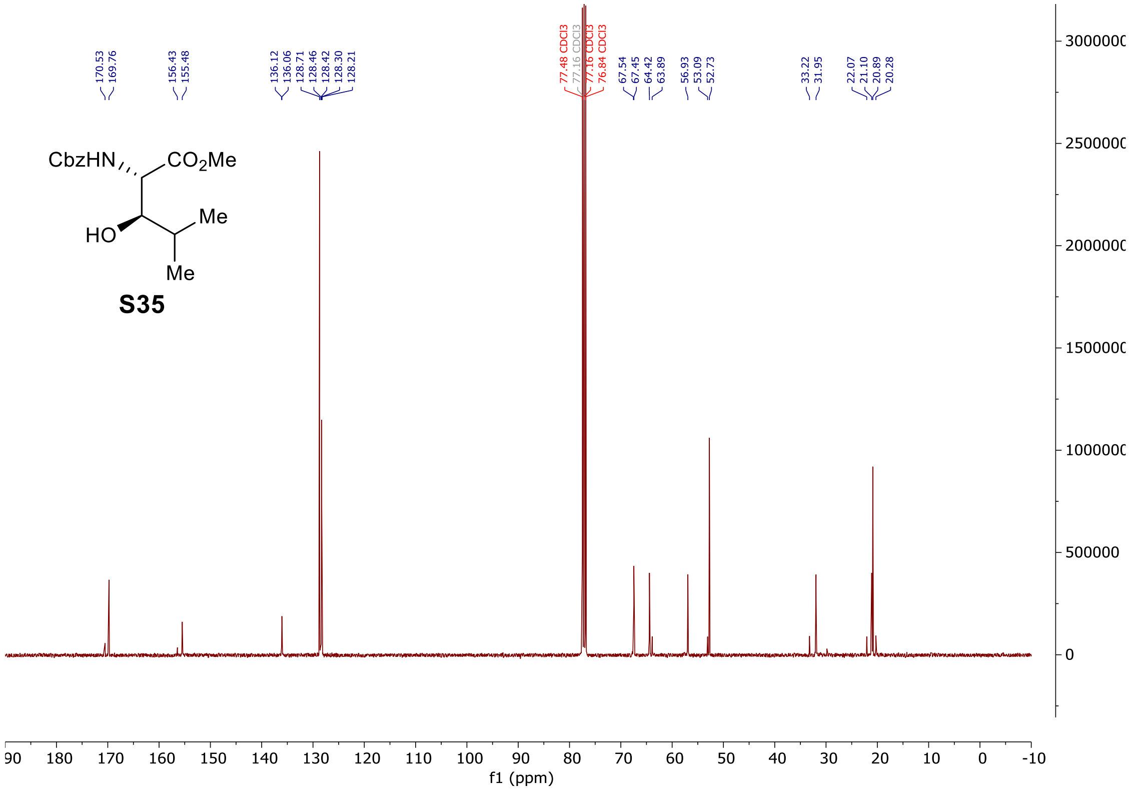

^{1}**H NMR** (400 MHz, $CDCl_3$) of compound **S36**

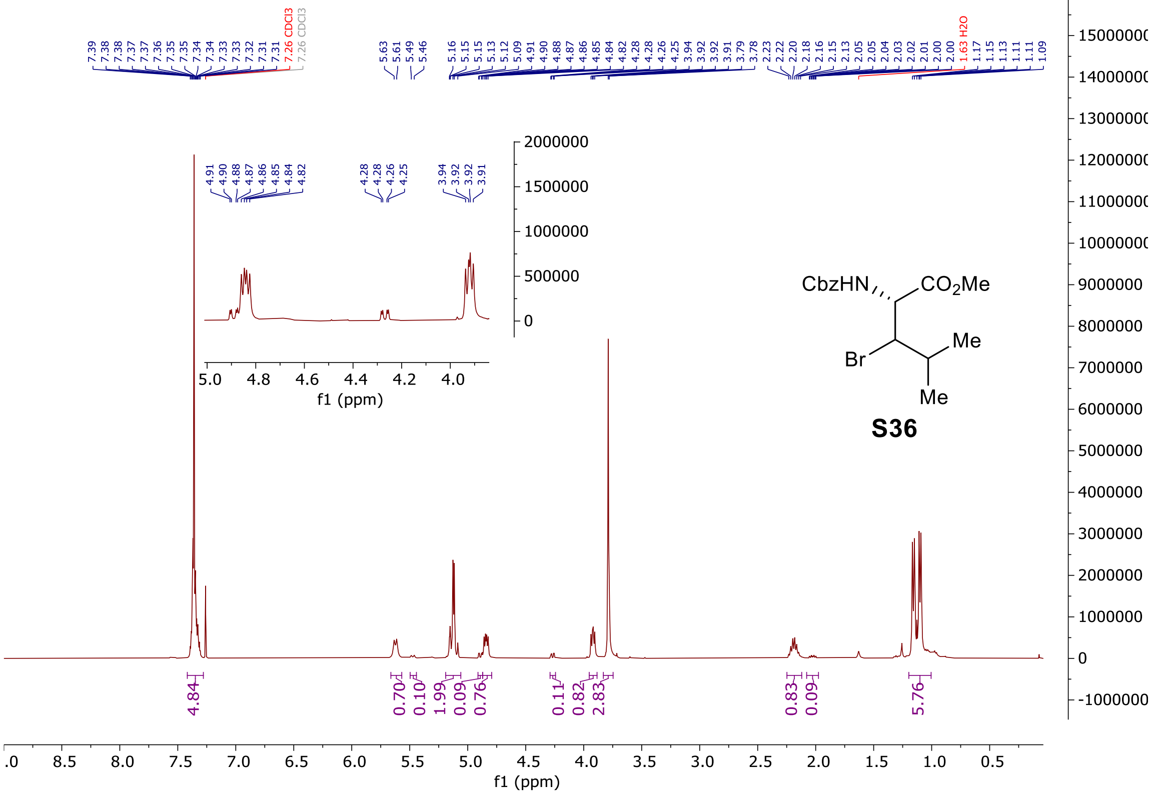

^{13}**C NMR** (101 MHz, $CDCl_3$) of compound **S36**

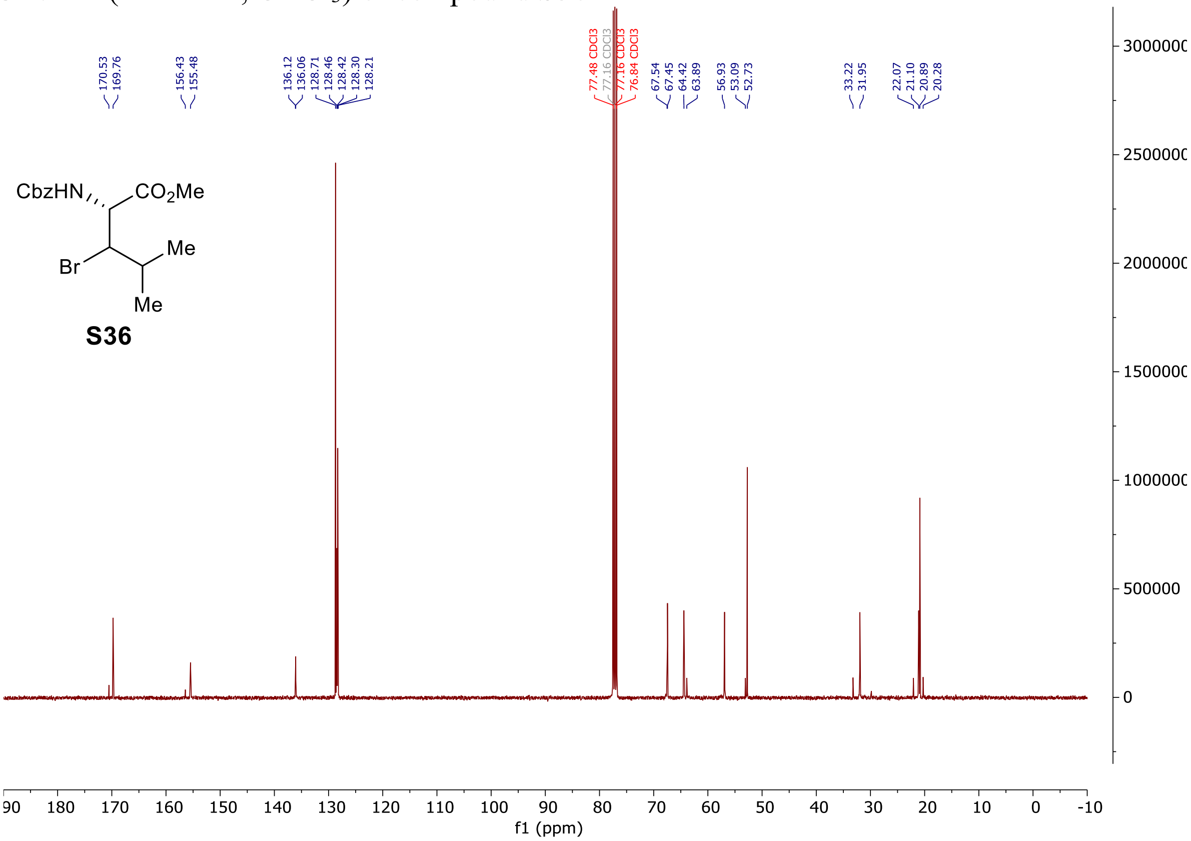

^{1}H NMR (400 MHz, $CDCl_3$) of compound **S37**

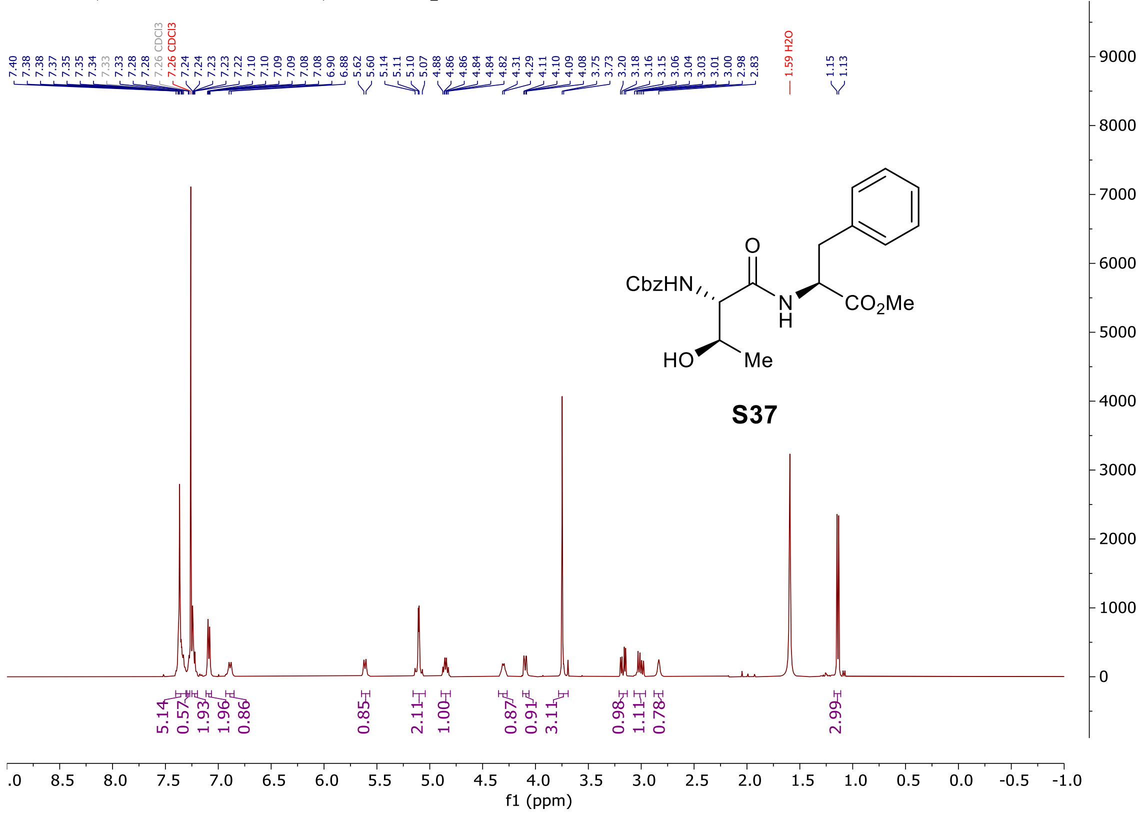

^{1}H NMR (800 MHz, $CDCl_3$) of compound **S38**

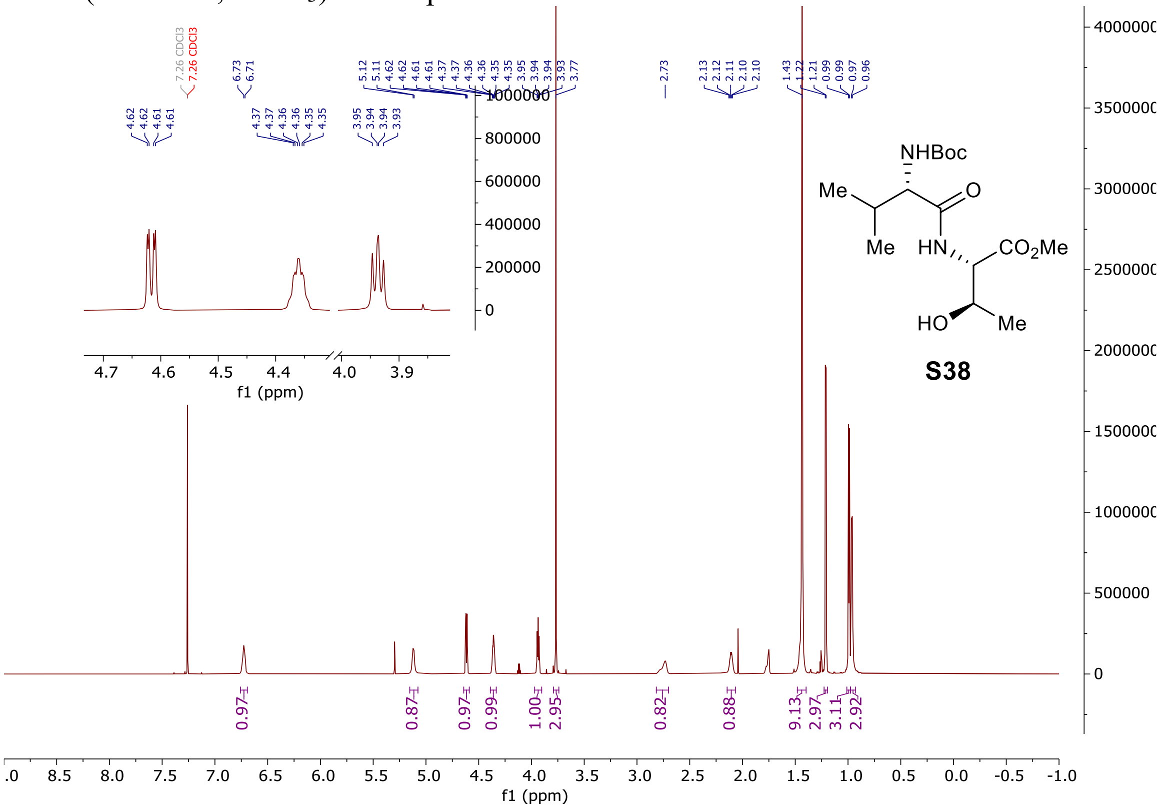

^{13}C NMR (201 MHz, $CDCl_3$) of compound **S38**

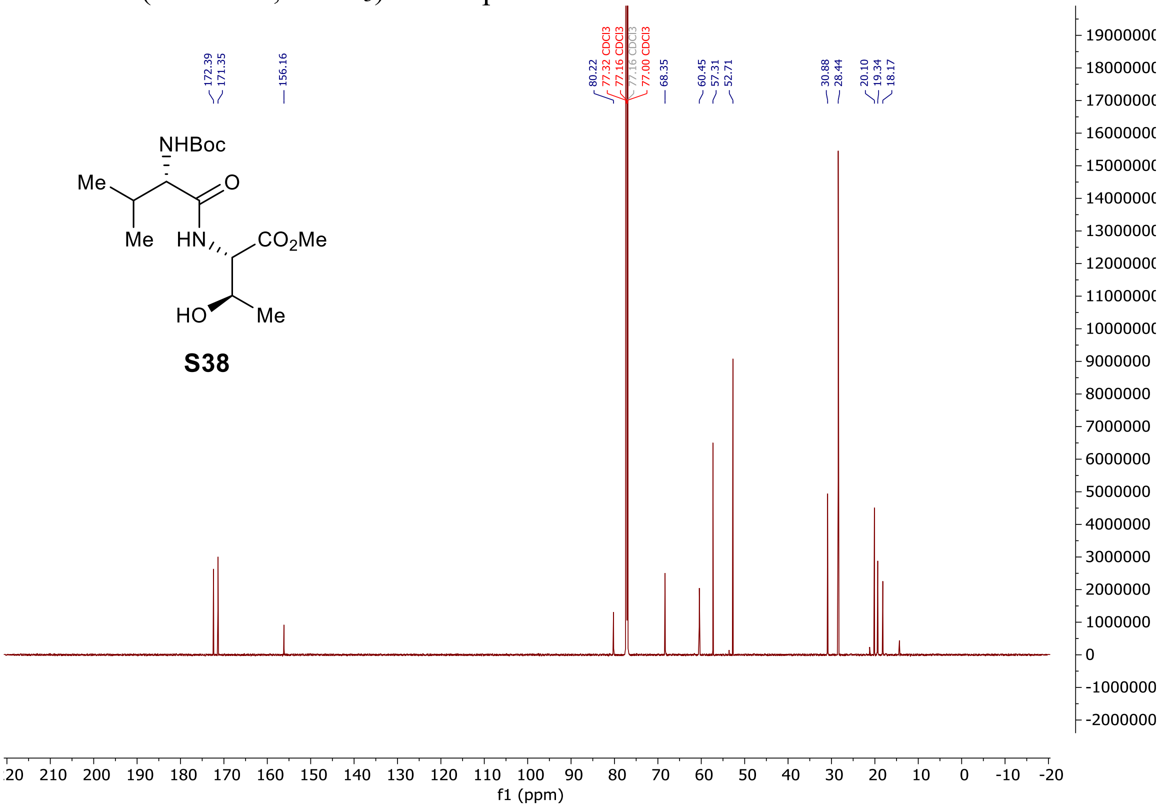

^{1}H NMR (400 MHz, $CDCl_3$) of compound **S39**

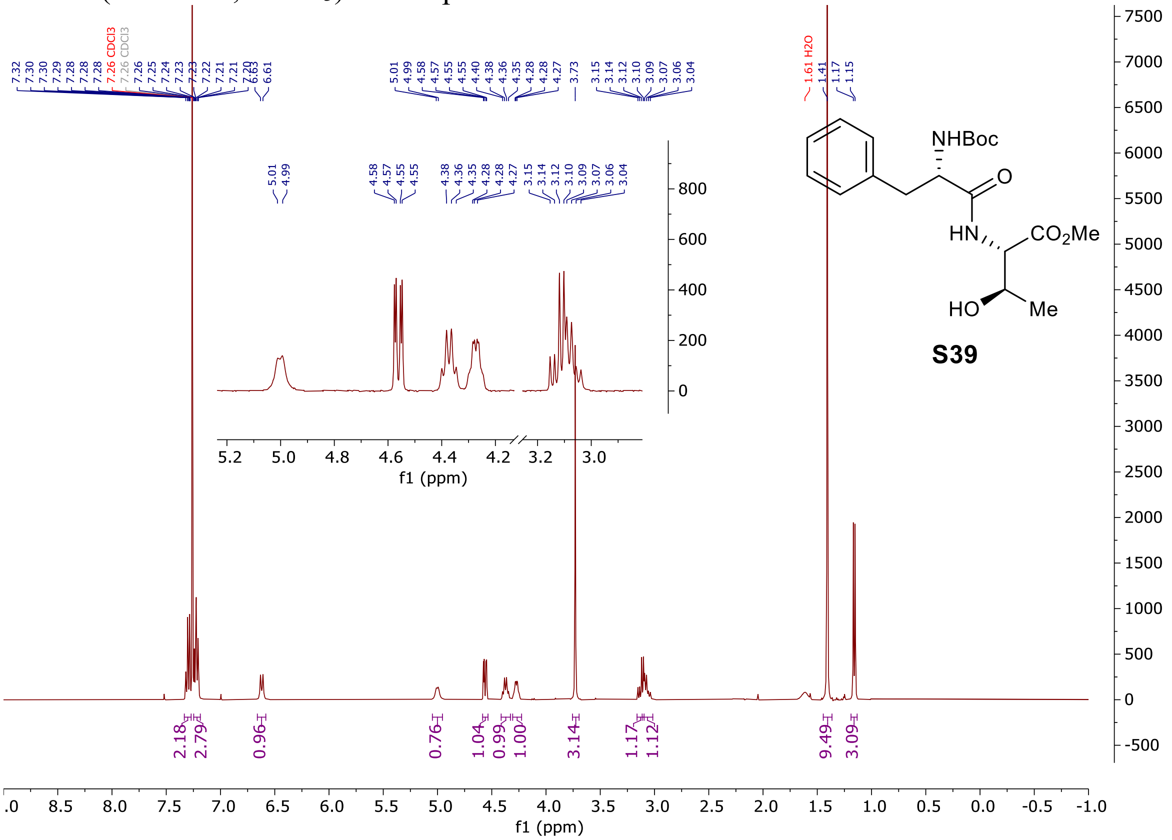

^{1}H NMR (400 MHz, $CDCl_3$) of compound **S40**

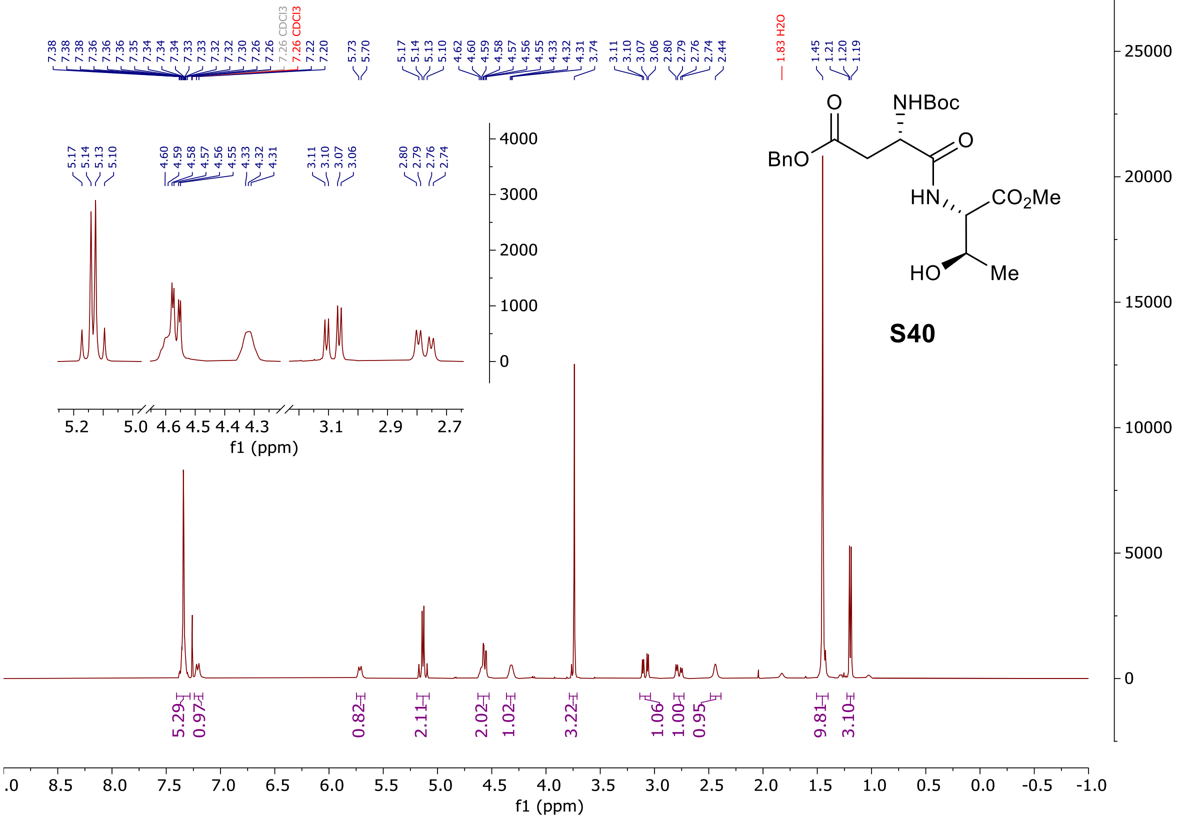

^{1}H NMR (800 MHz, $CDCl_3$) of compound **S41**

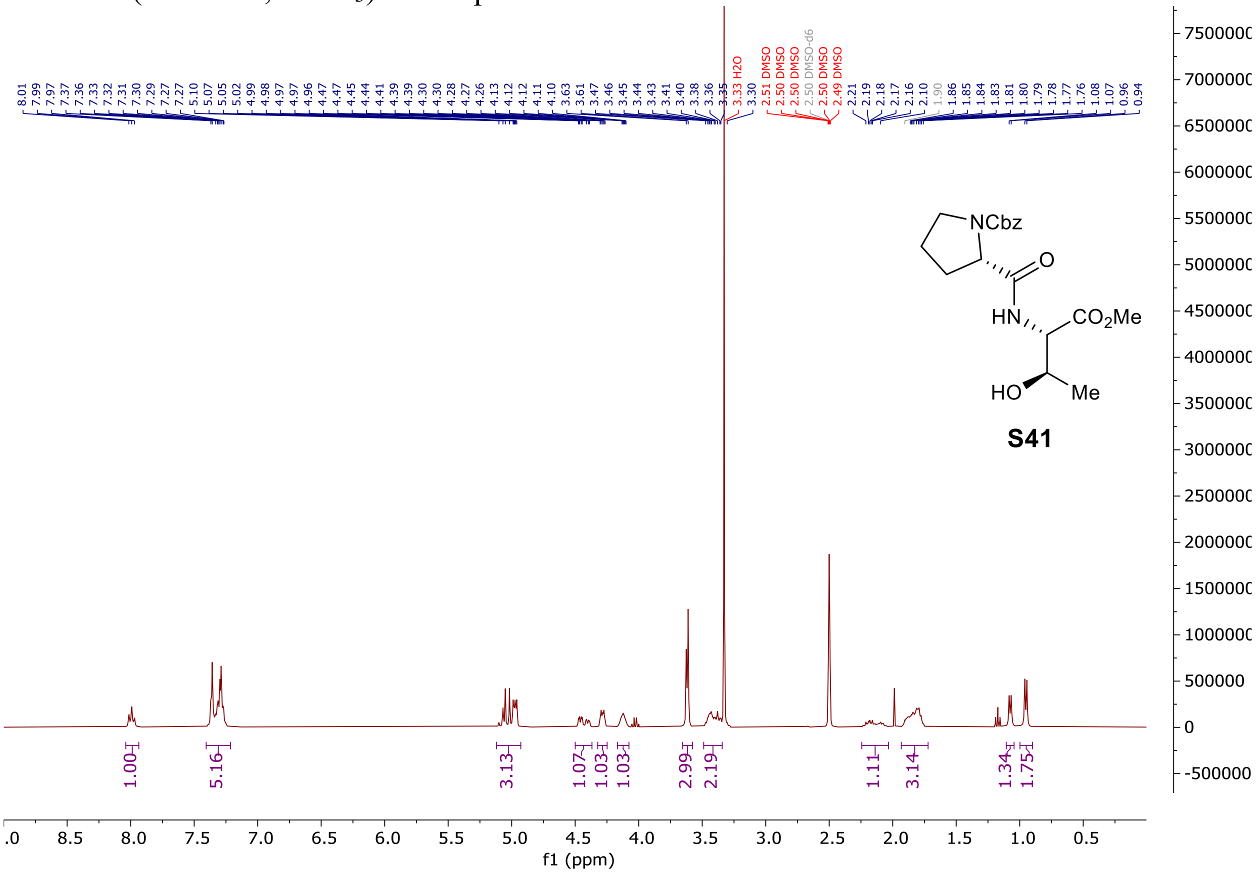

^{1}H NMR (400 MHz, $CDCl_3$) of compound **S42**

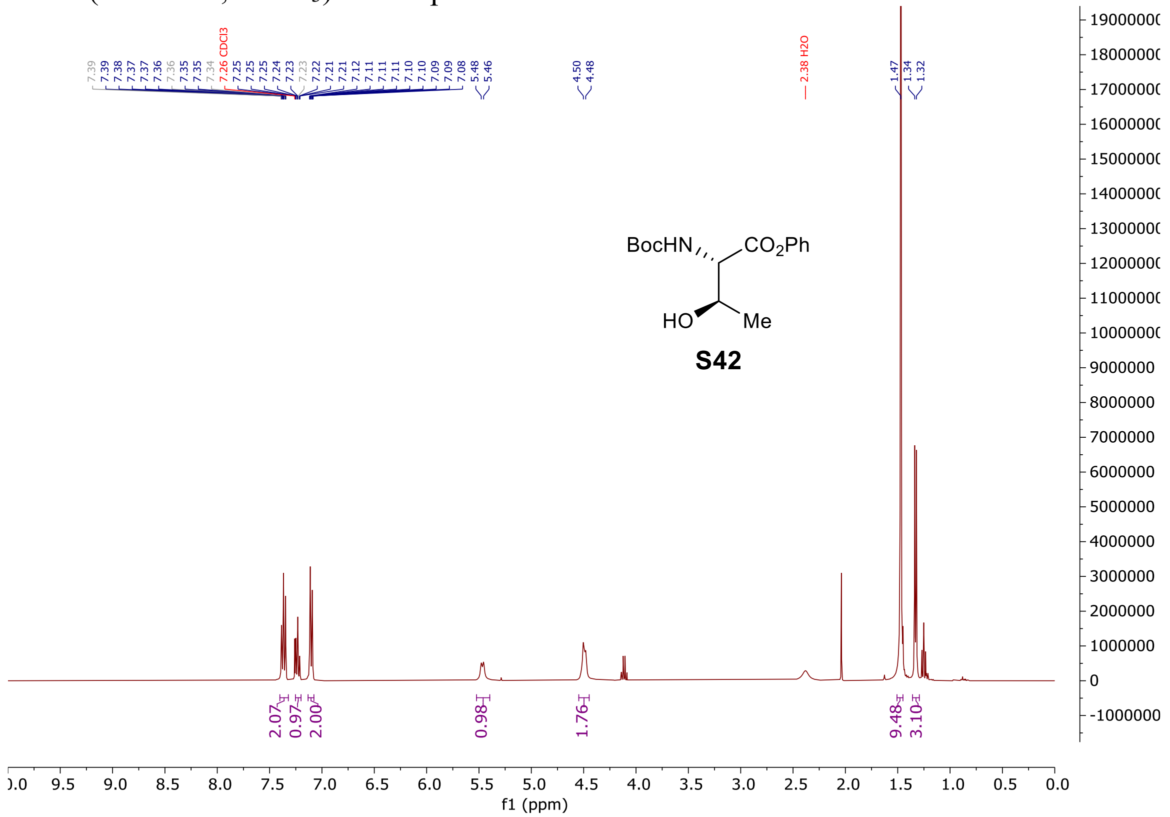

^{13}C NMR (101 MHz, $CDCl_3$) of compound **S42**

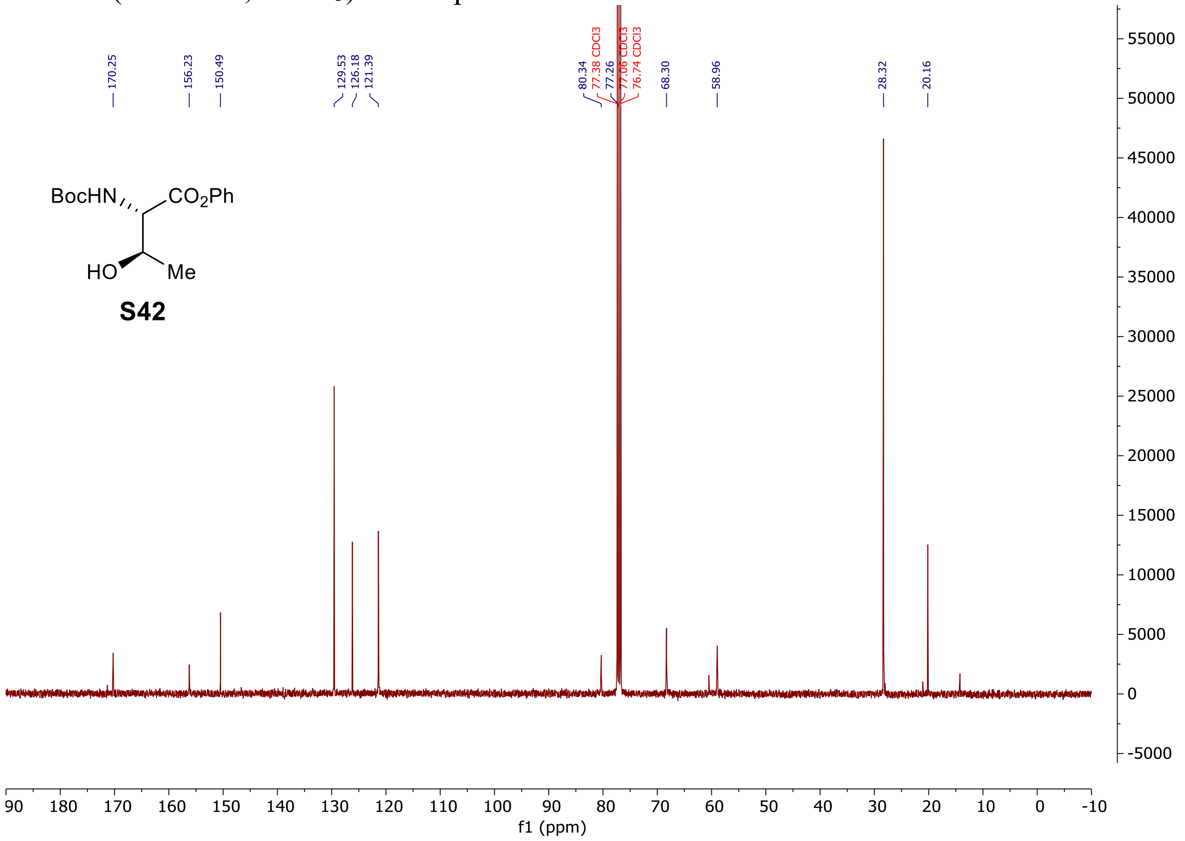

^{1}**H NMR** (400 MHz, $CDCl_3$) of compound **S43**

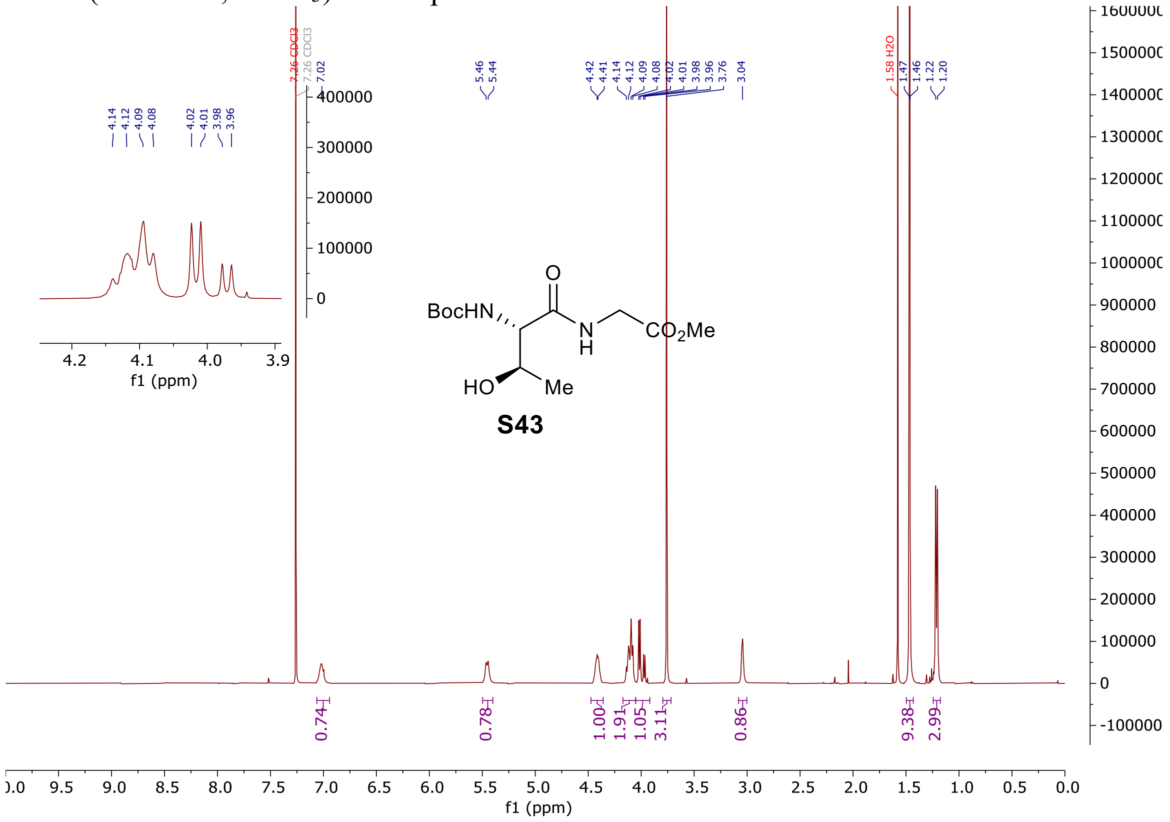

^{13}**C NMR** (101 MHz, $CDCl_3$) of compound **S43**

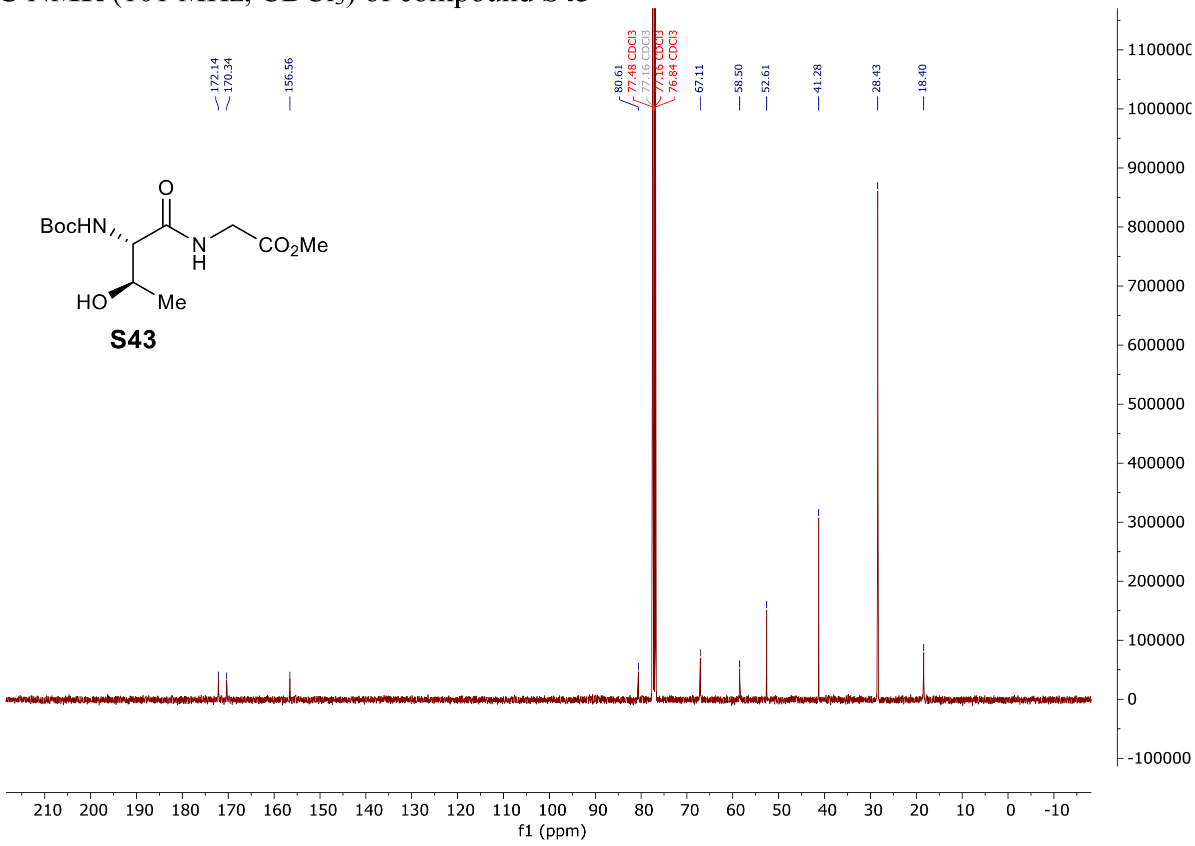

^{1}H NMR (600 MHz, $CDCl_3$) of compound **S44**

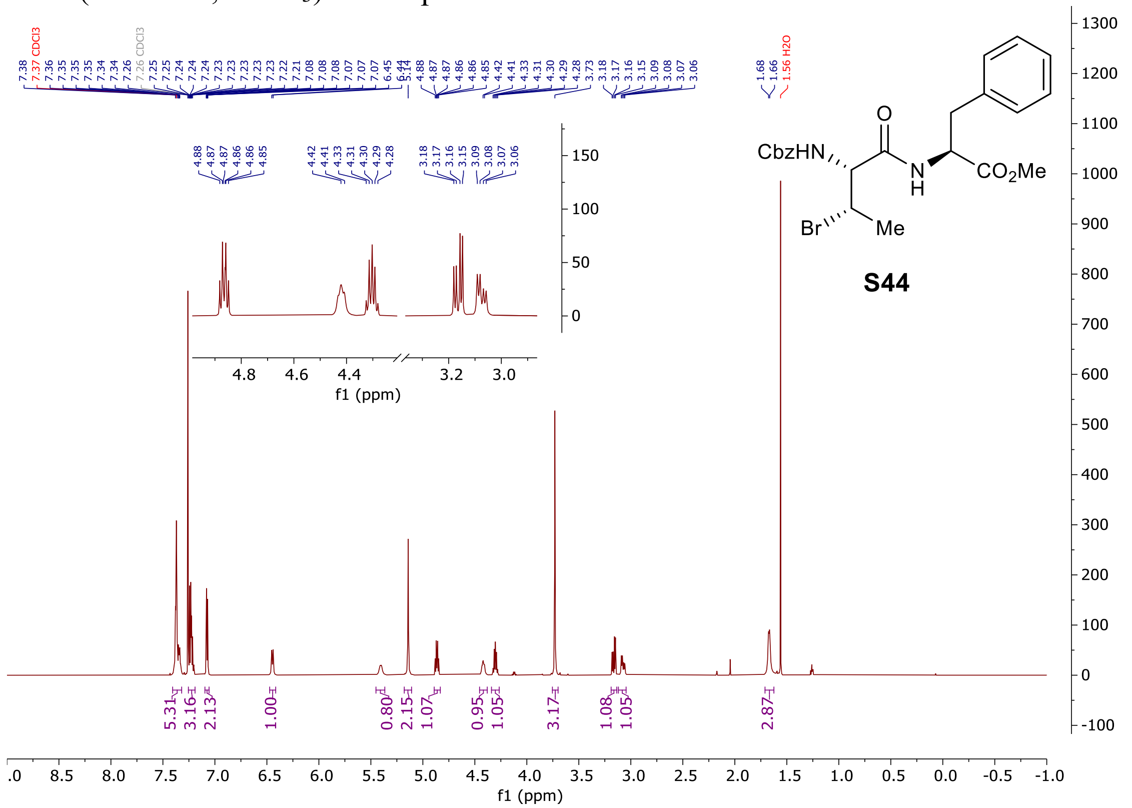

^{13}C NMR (151 MHz, $CDCl_3$) of compound **S44**

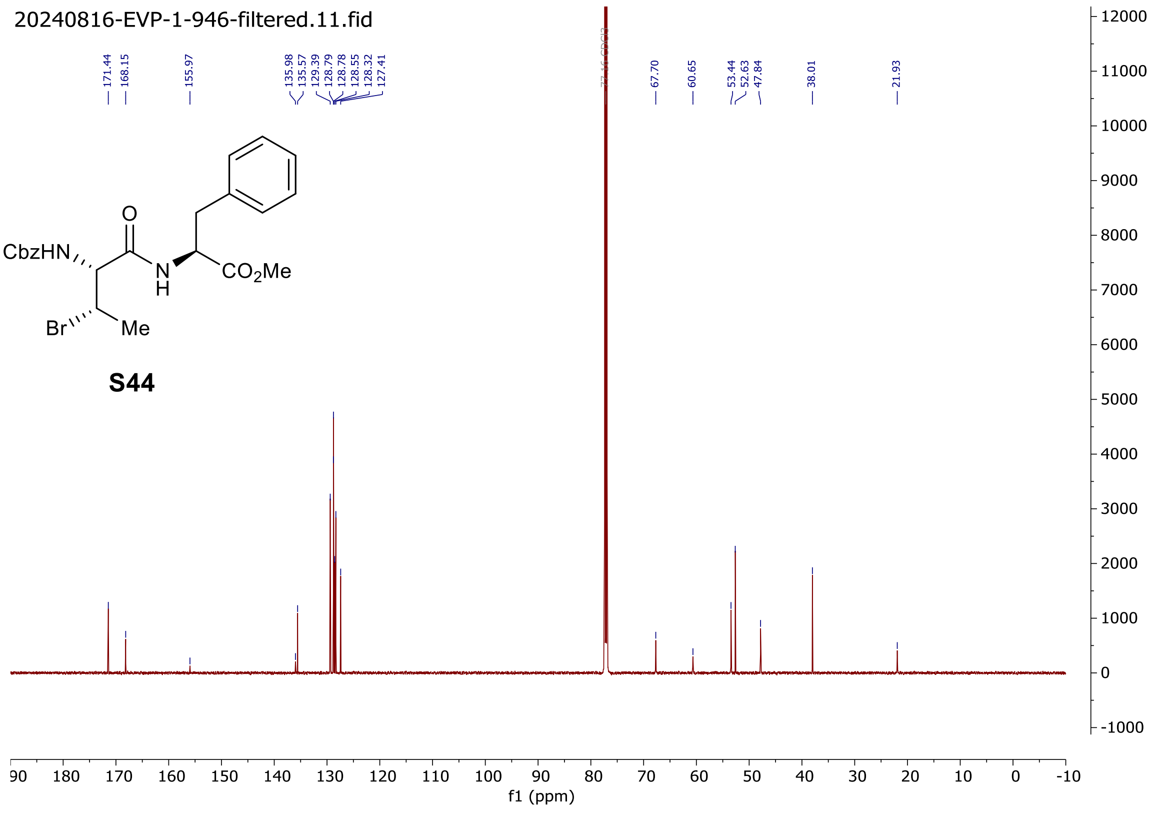

^{1}H NMR (400 MHz, $CDCl_3$) of compound **24**

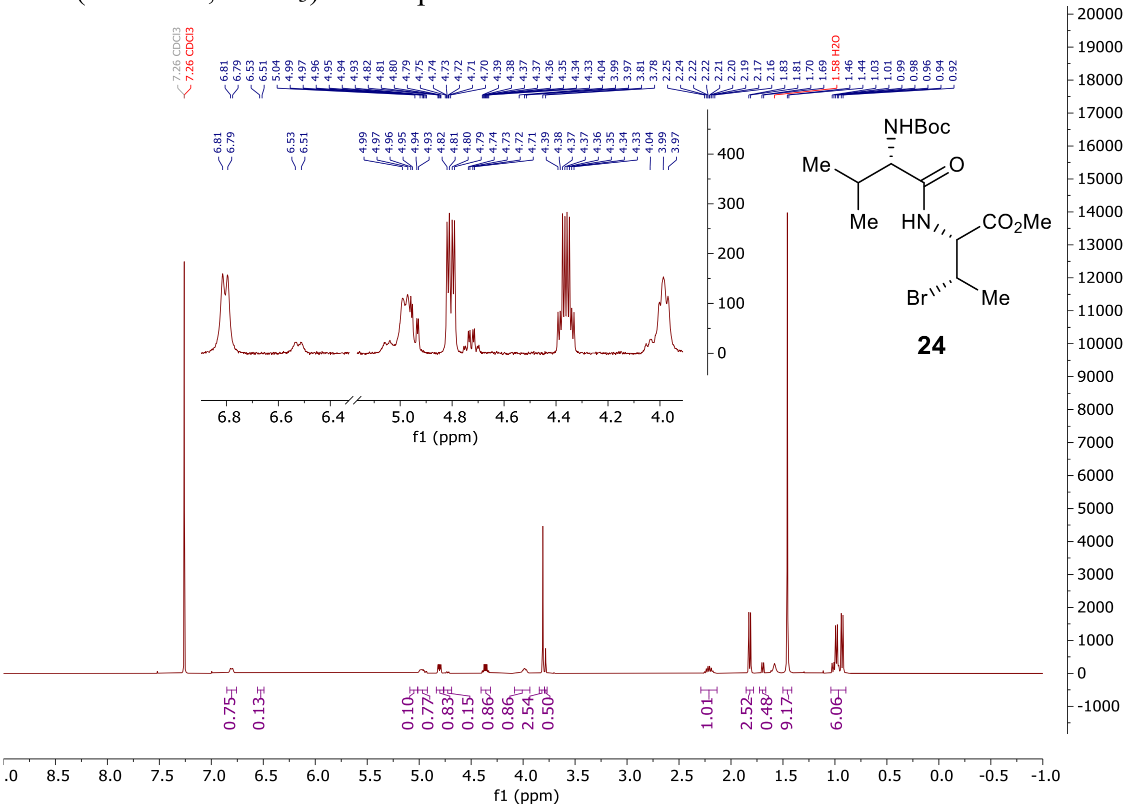

^{13}C NMR (101 MHz, $CDCl_3$) of compound **24**

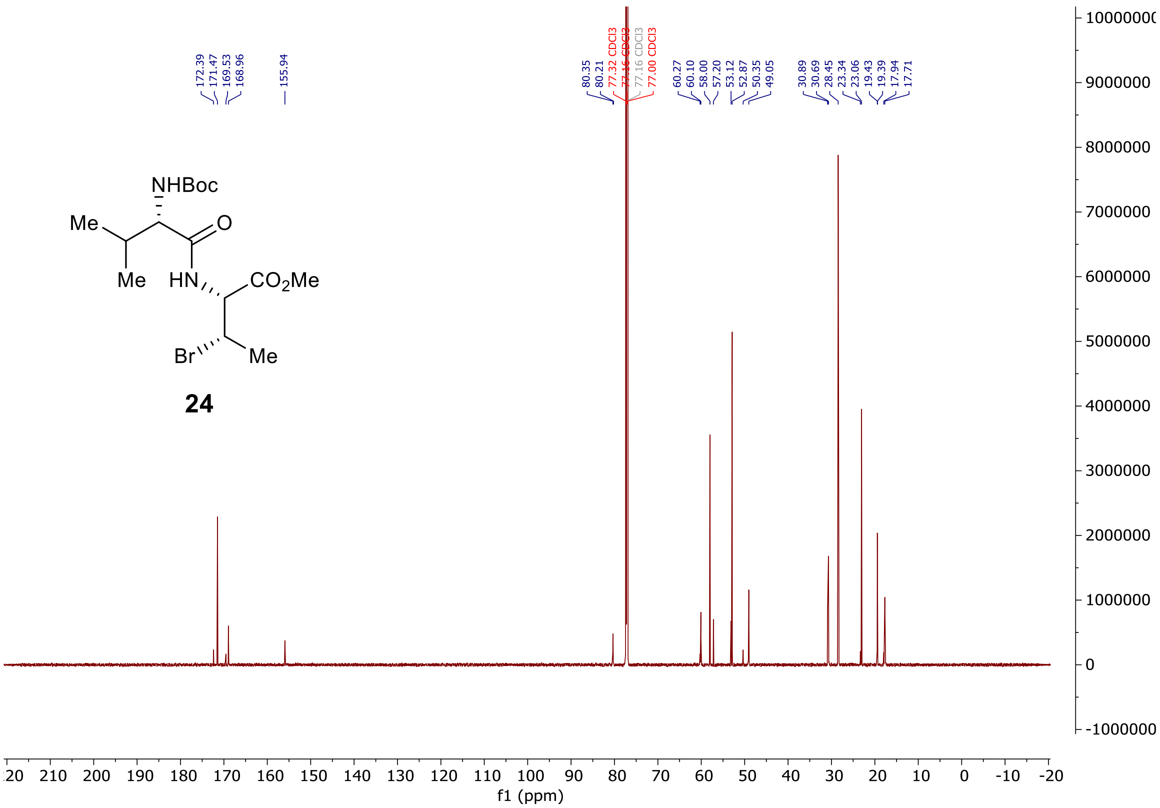

¹H NMR (800 MHz, $CDCl_3$) of compound **S45**

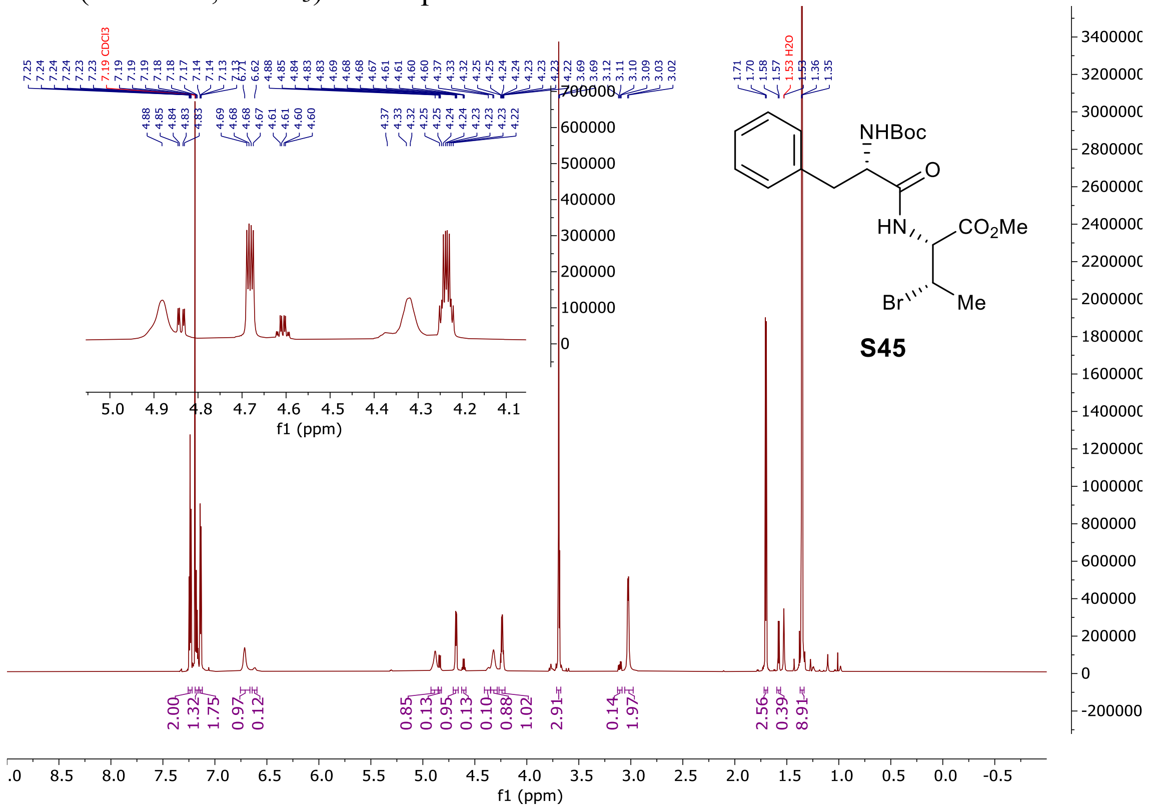

¹³C NMR (201 MHz, $CDCl_3$) of compound **S45**

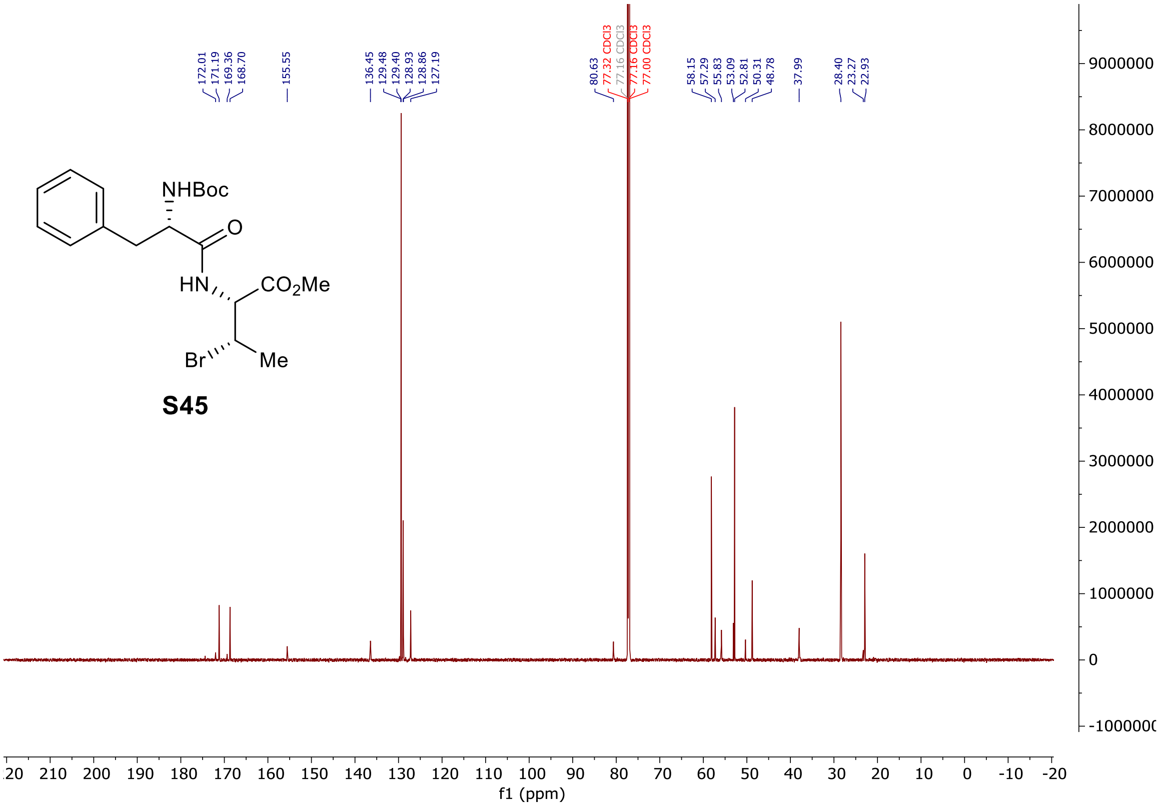

^{1}H NMR (400 MHz, $CDCl_3$) of compound **S46**

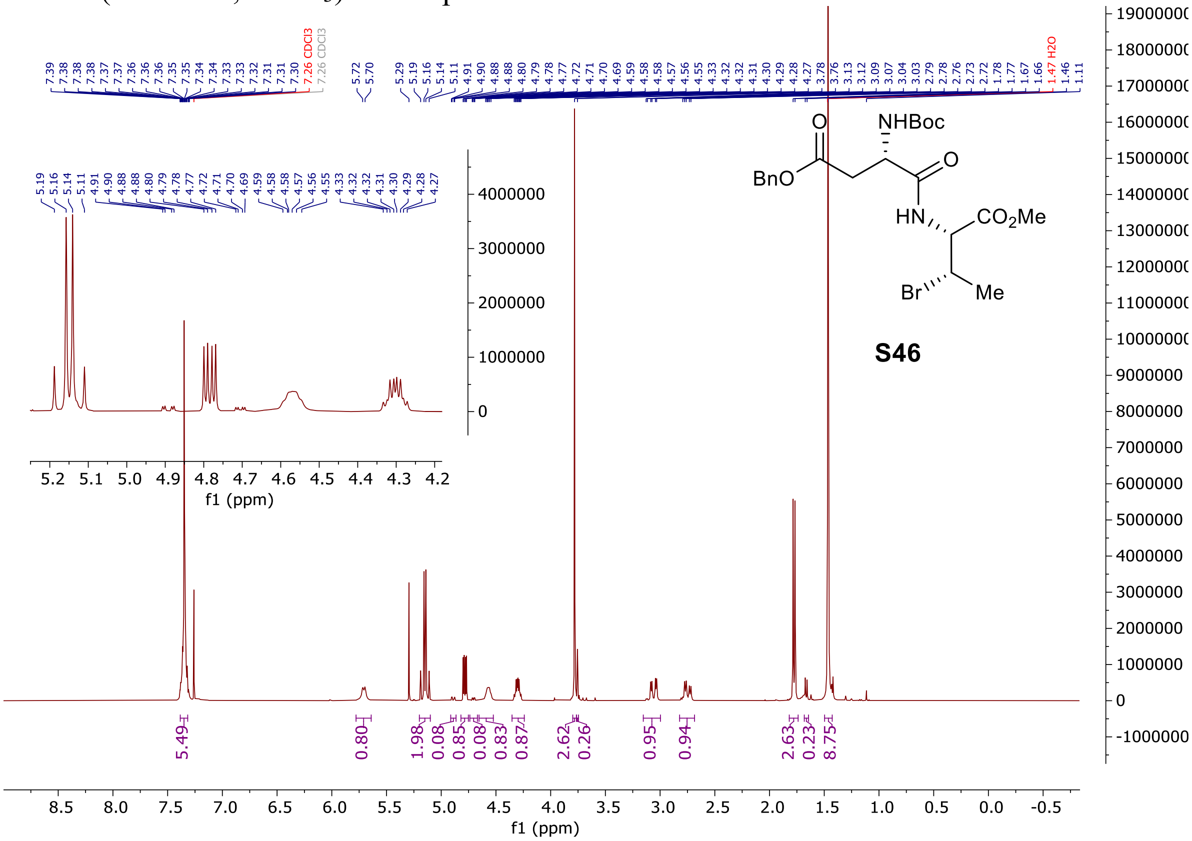

^{13}C NMR (151 MHz, $CDCl_3$) of compound **S46**

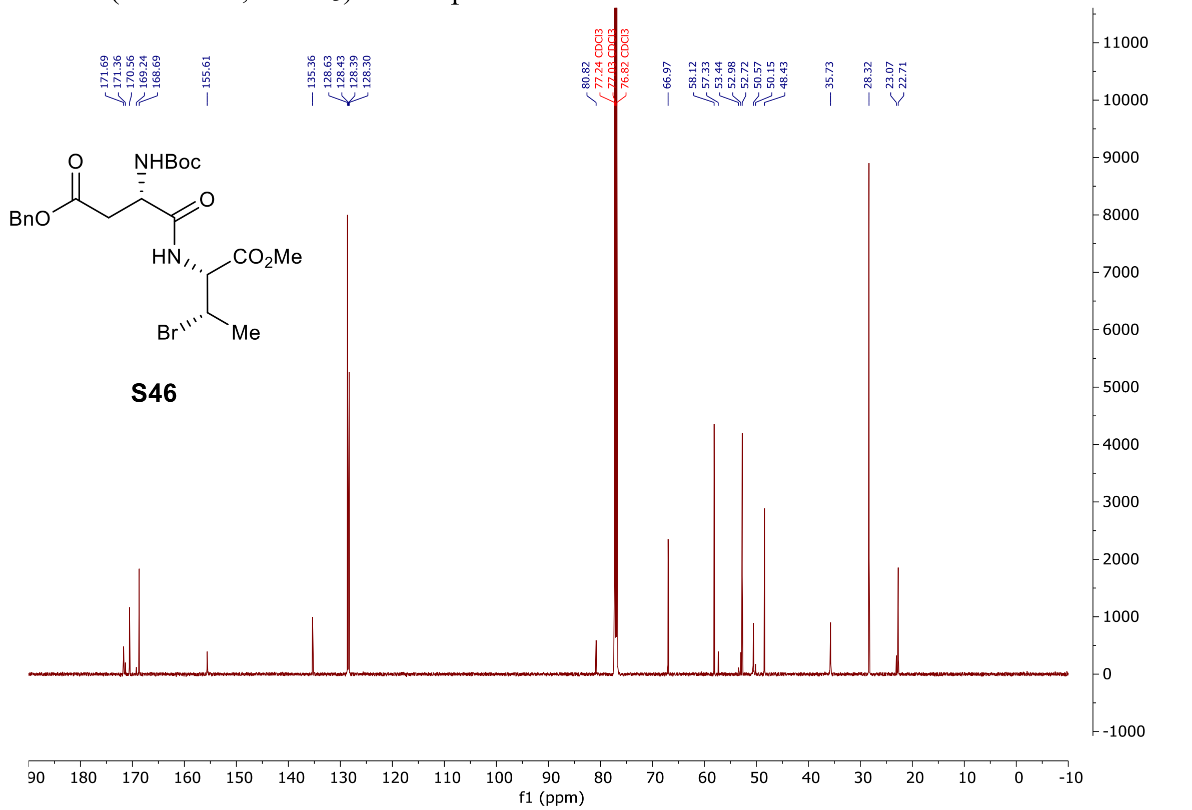

^{1}H NMR (400 MHz, $CDCl_3$) of compound **S47**

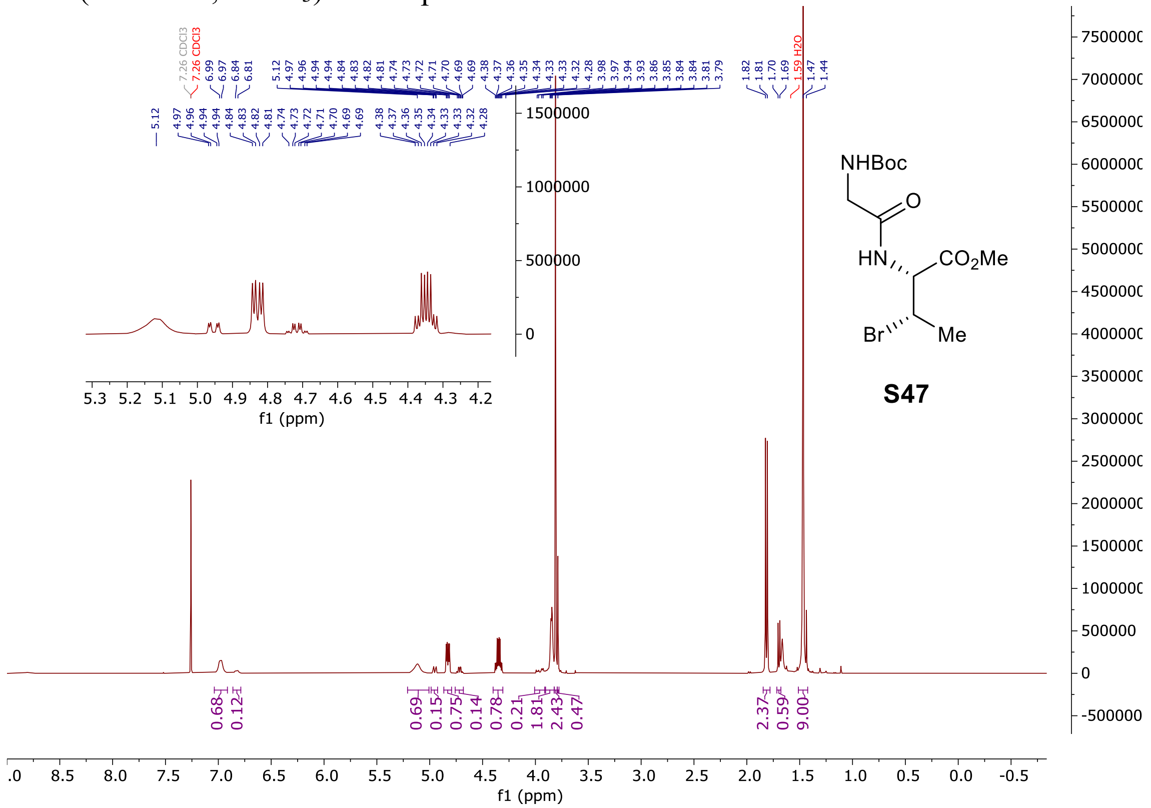

^{13}C NMR (151 MHz, $CDCl_3$) of compound **S47**

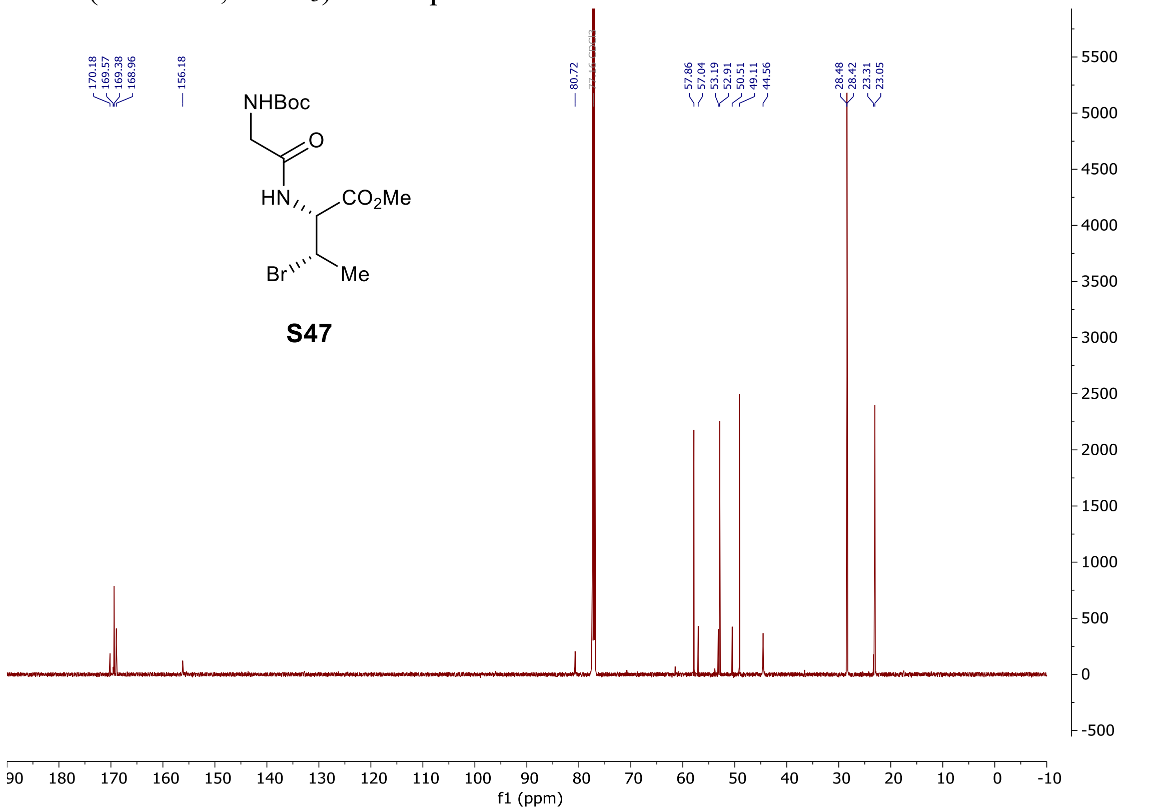

^{1}H NMR (400 MHz, $CDCl_3$) of compound **S48**

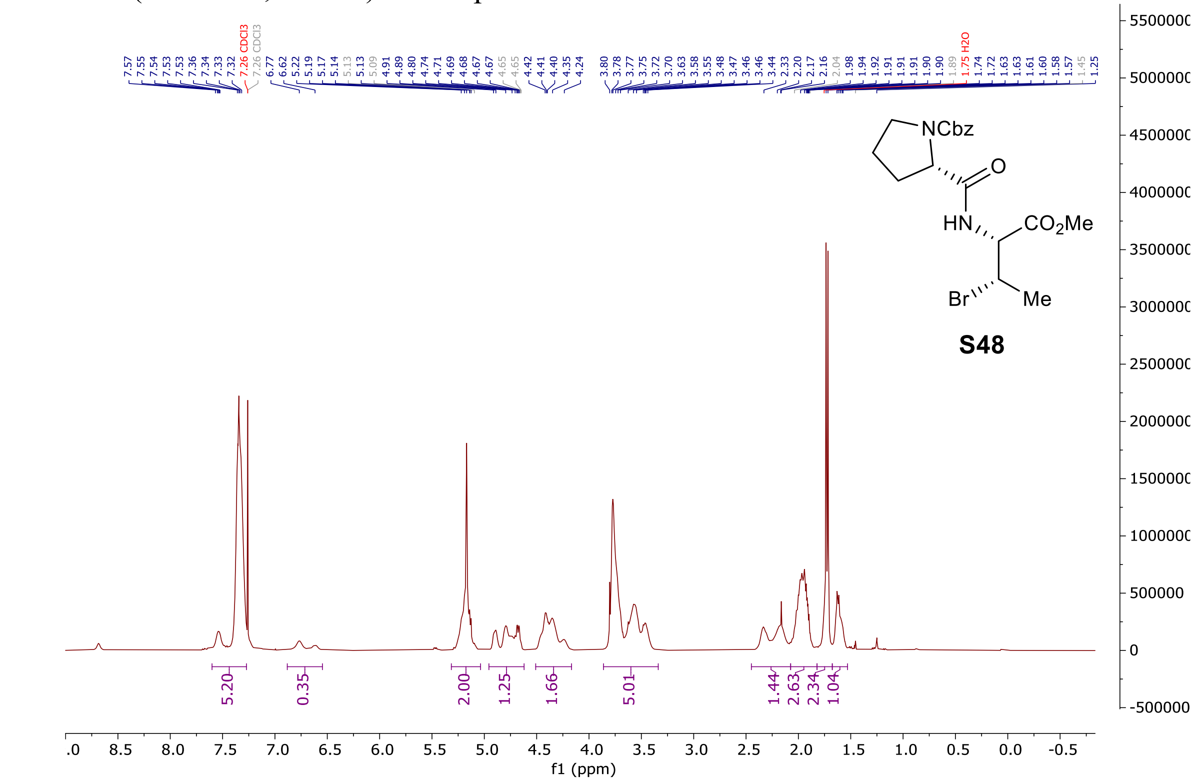

^{13}C NMR (101 MHz, $CDCl_3$) of compound **S48**

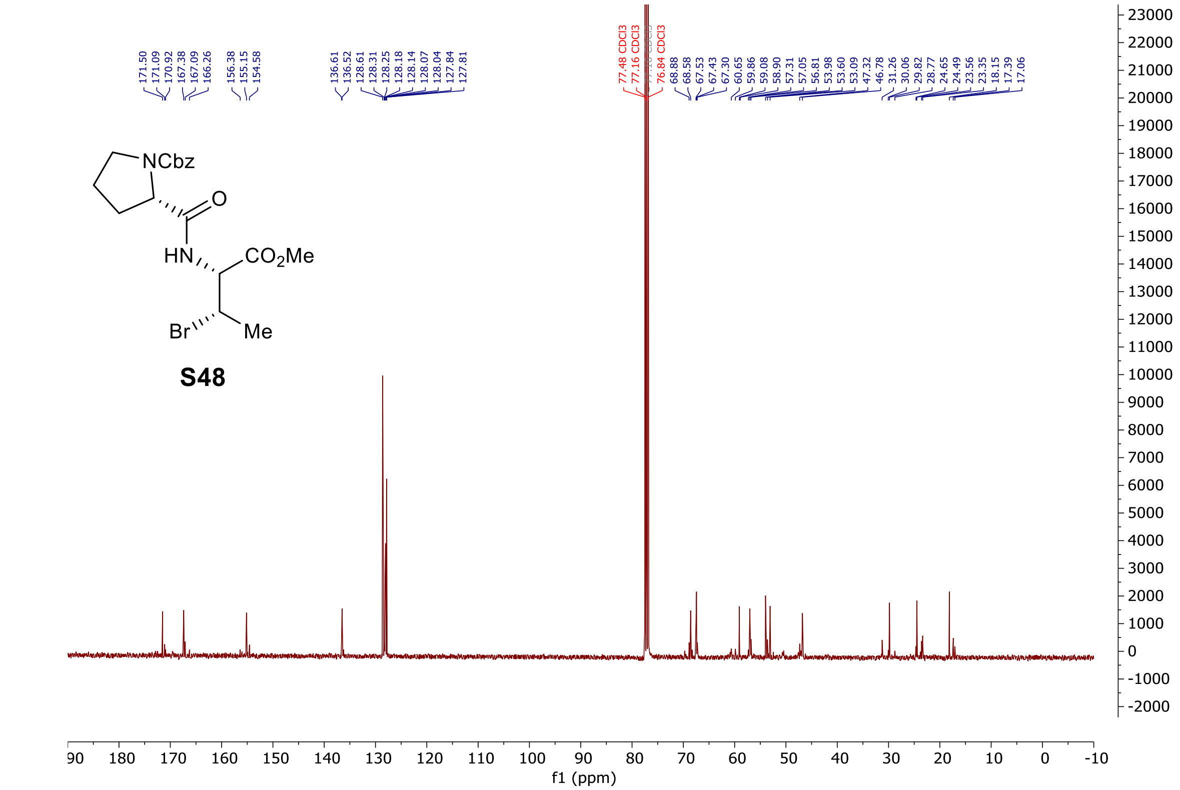

^{1}H NMR (400 MHz, $CDCl_3$) of compound **S49**

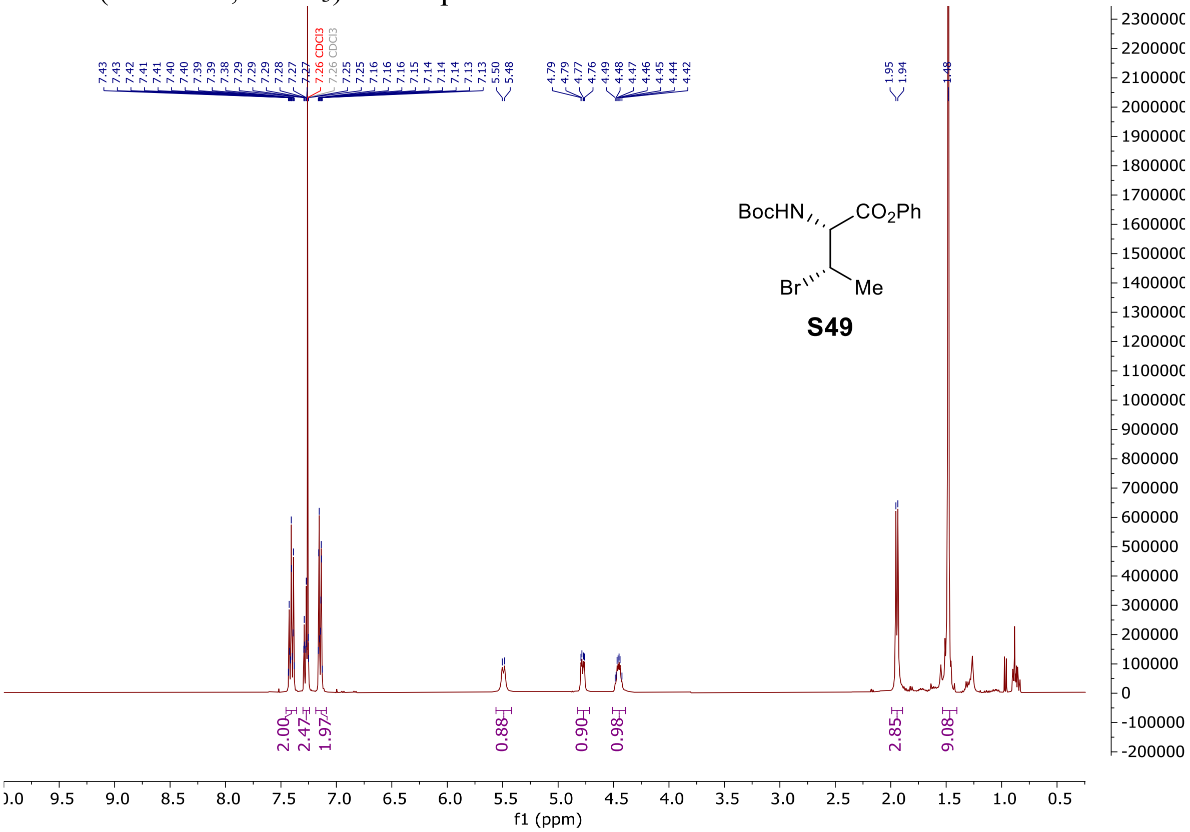

^{13}C NMR (101 MHz, $CDCl_3$) of compound **S49**

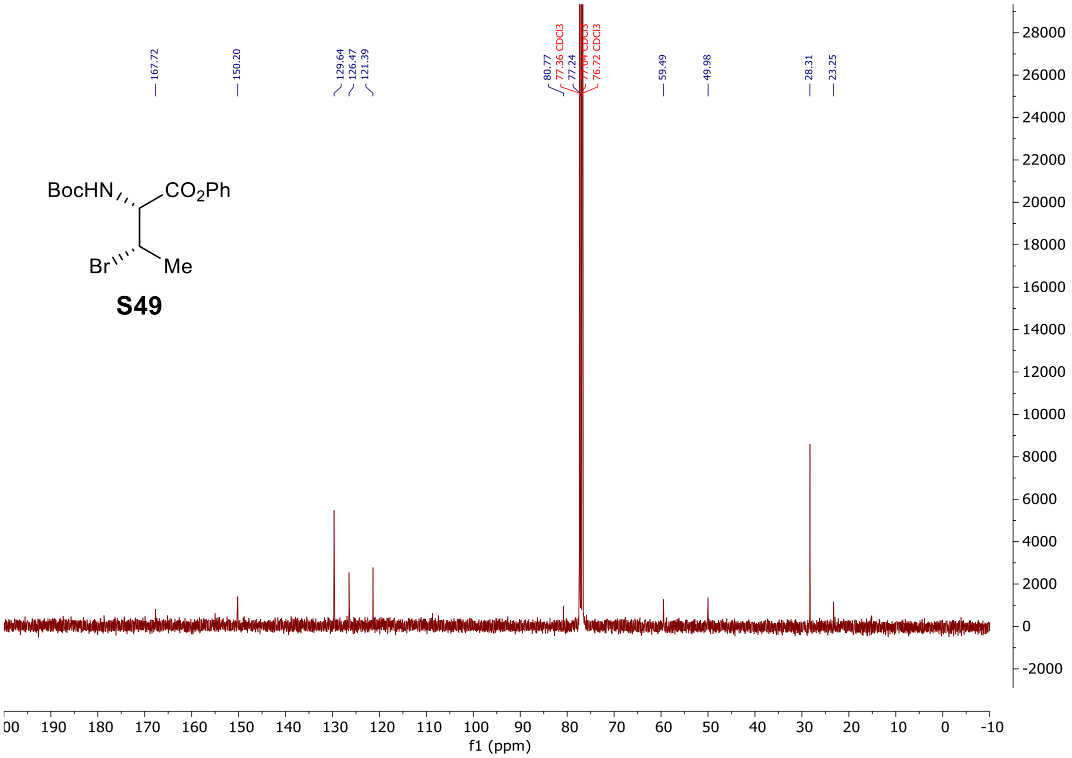

^{1}H NMR (400 MHz, $CDCl_3$) of compound **S50**

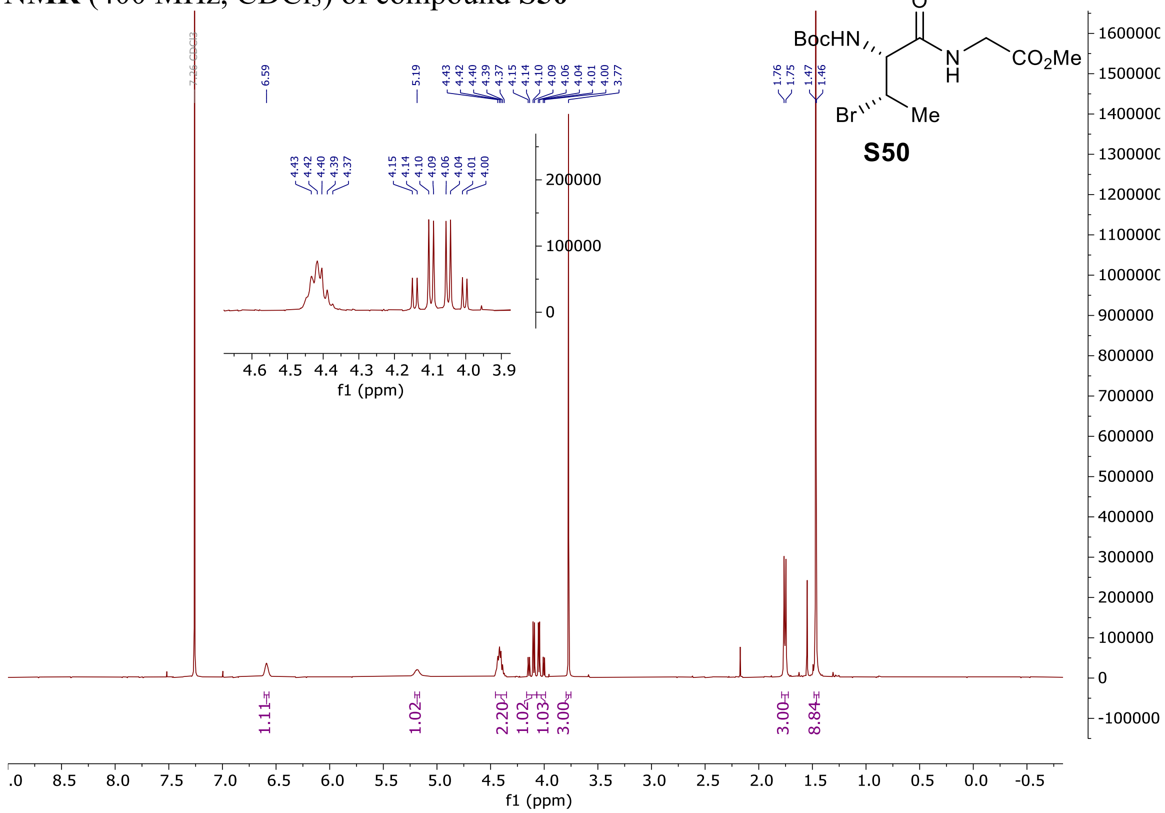

^{13}C NMR (101 MHz, $CDCl_3$) of compound **S50**

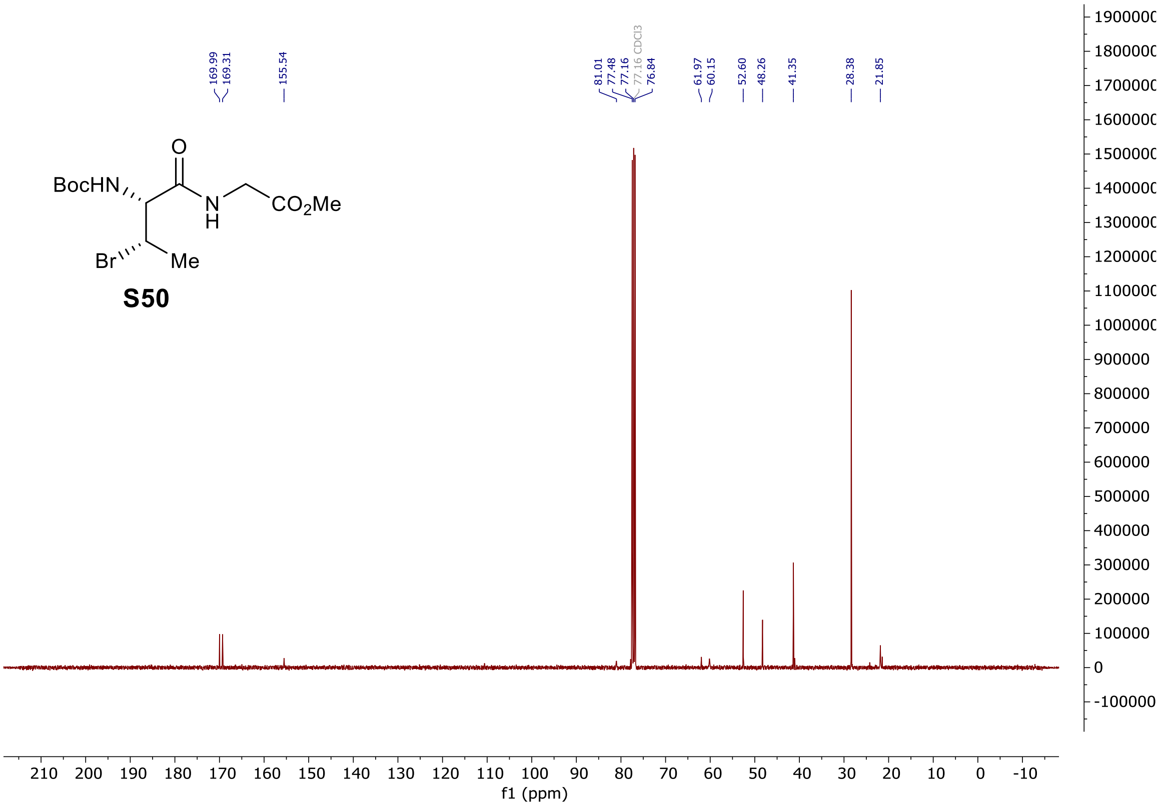

^{1}H NMR (800 MHz, $CDCl_3$, 25 °C) of compound **6** (rotamers visible, 12:1 isolated dr)

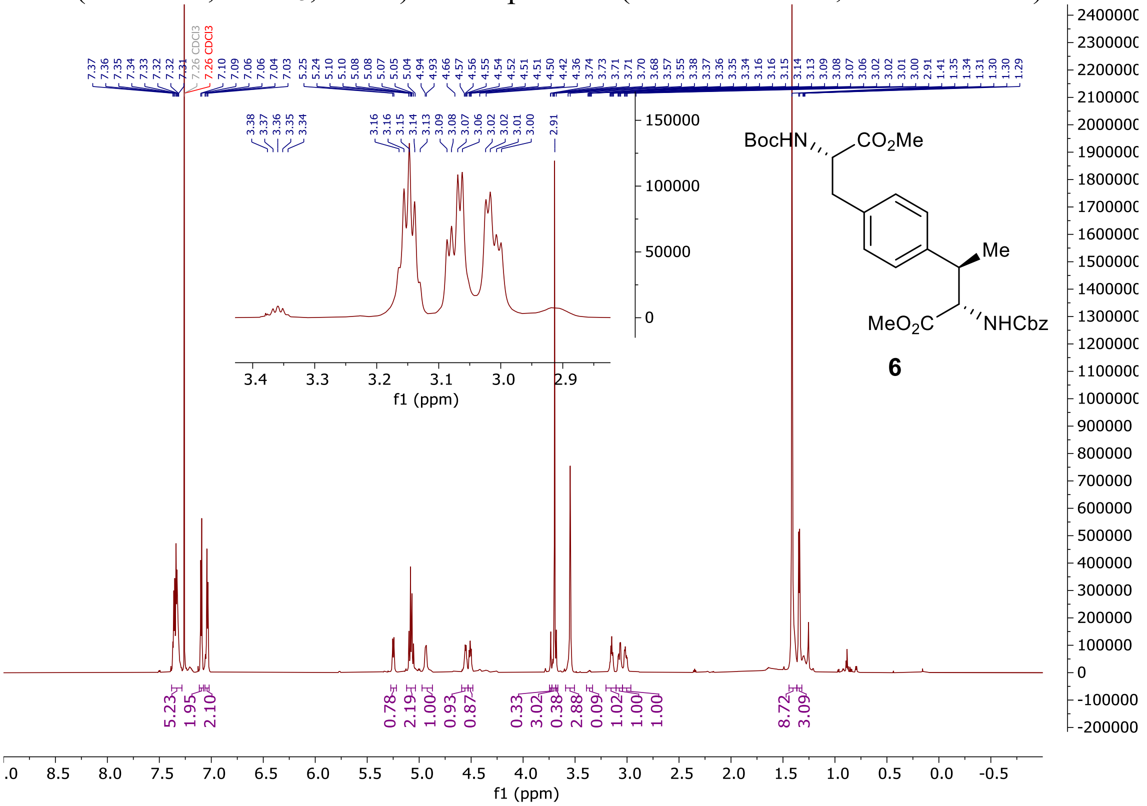

^{13}C NMR (201 MHz, $CDCl_3$) of compound **6**

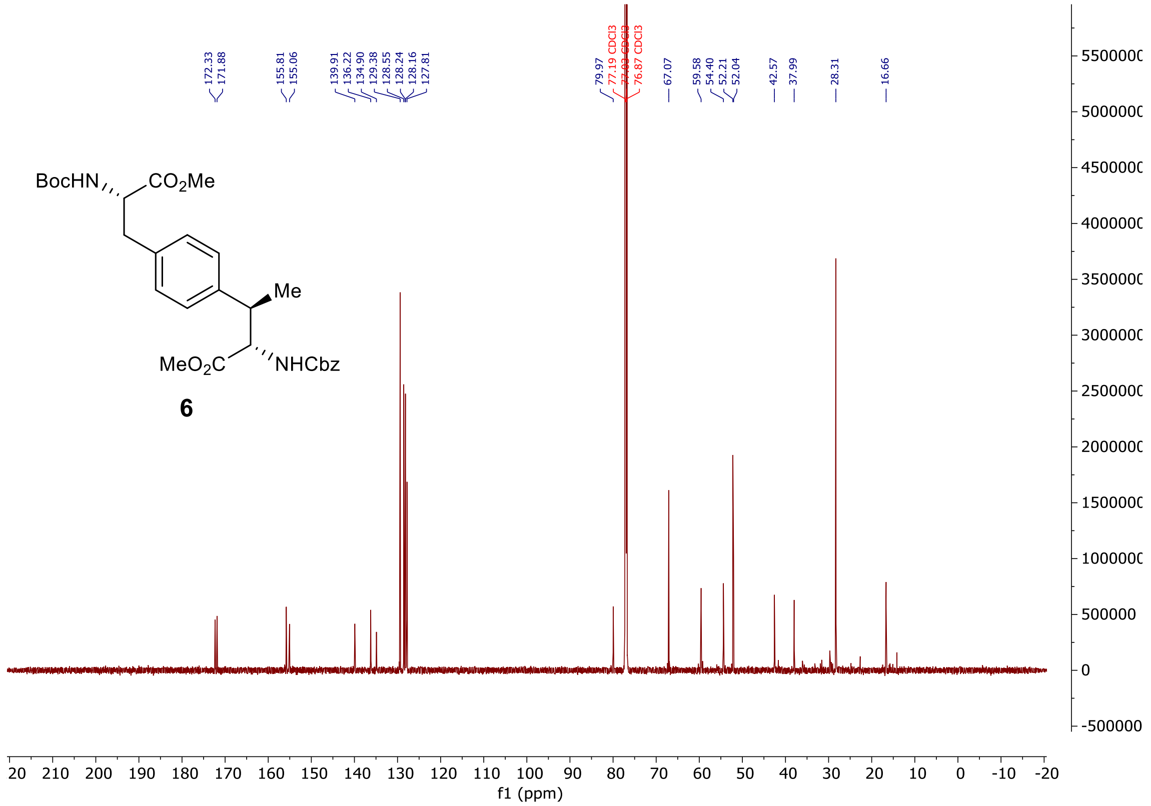

NOESY (800 MHz, $CDCl_3$) of compound **6**

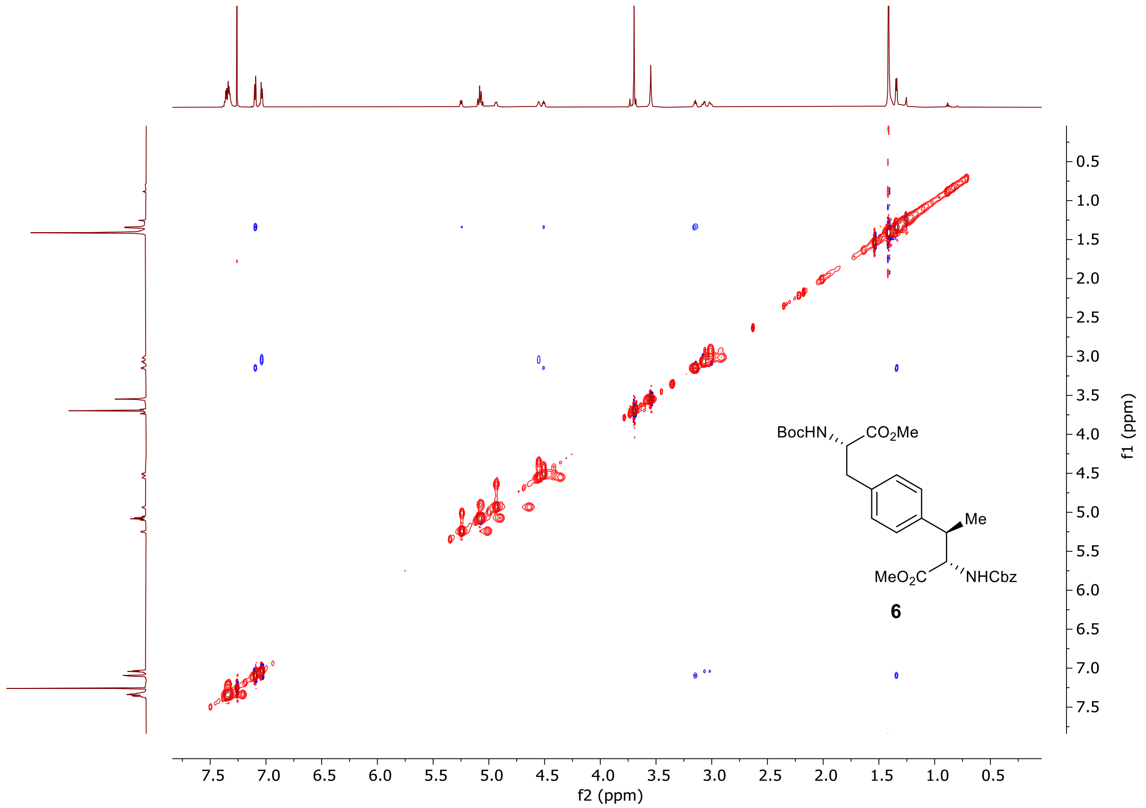

COSY (800 MHz, $CDCl_3$) of compound **6**

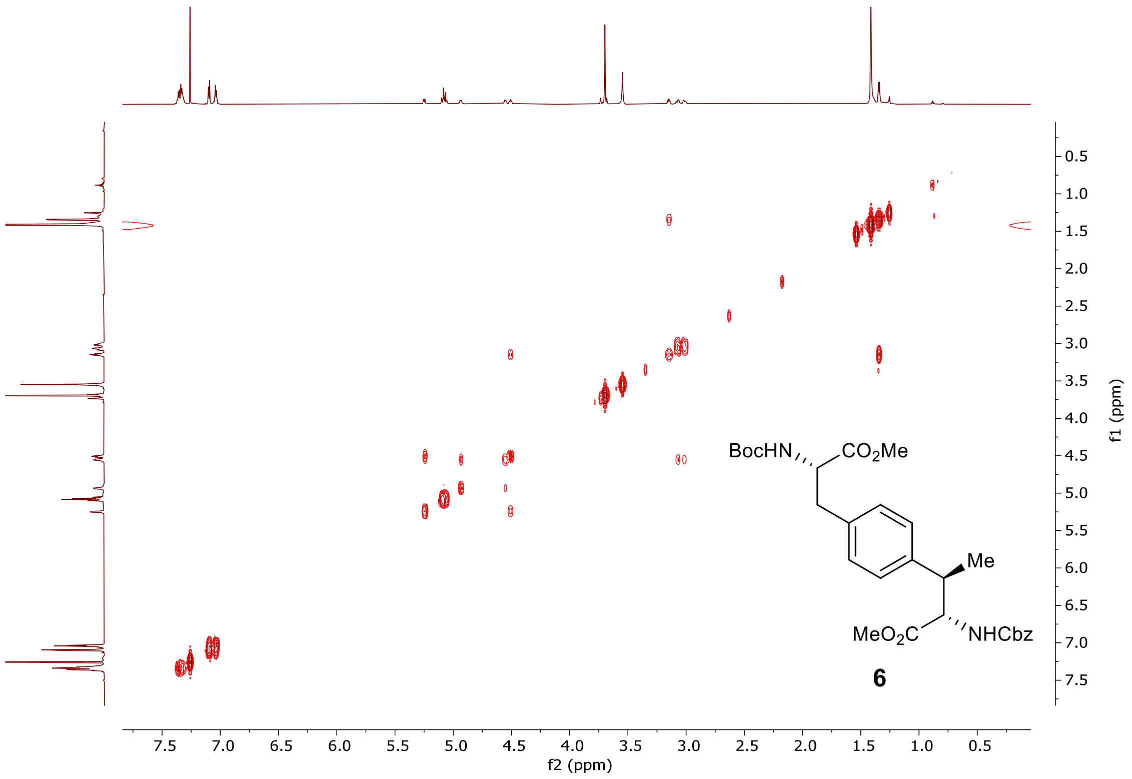

^{1}H NMR (600 MHz, $CDCl_3$, 50 °C) of compound **7** (5:1 isolated dr)

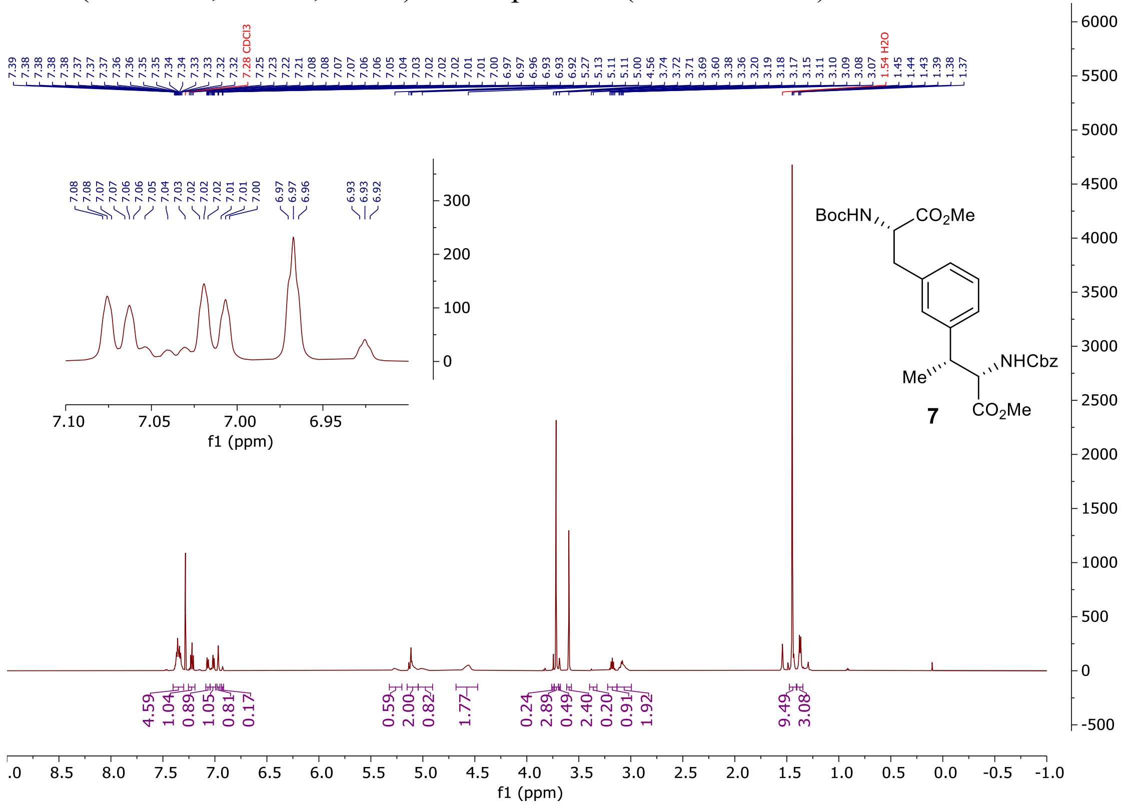

^{1}H NMR (600 MHz, $CDCl_3$, 25 °C) of compound **7** (rotamers visible)

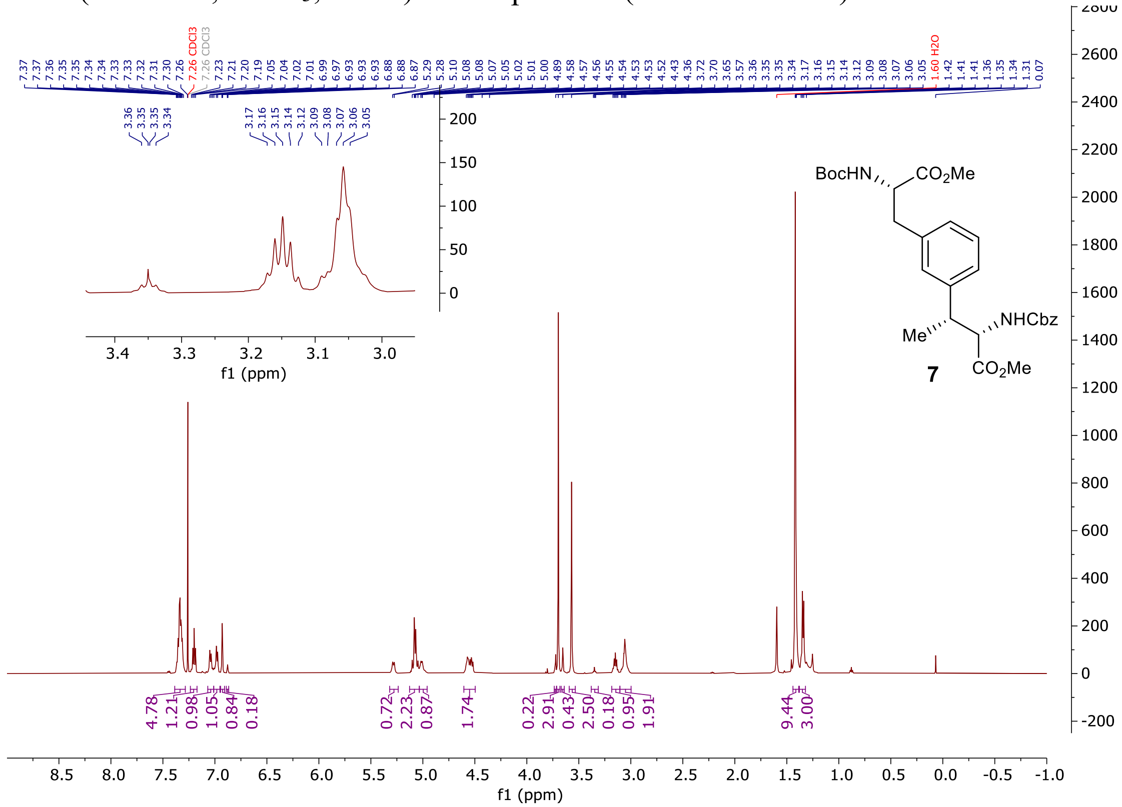

^{13}C NMR (151 MHz, $CDCl_3$) of compound **7**

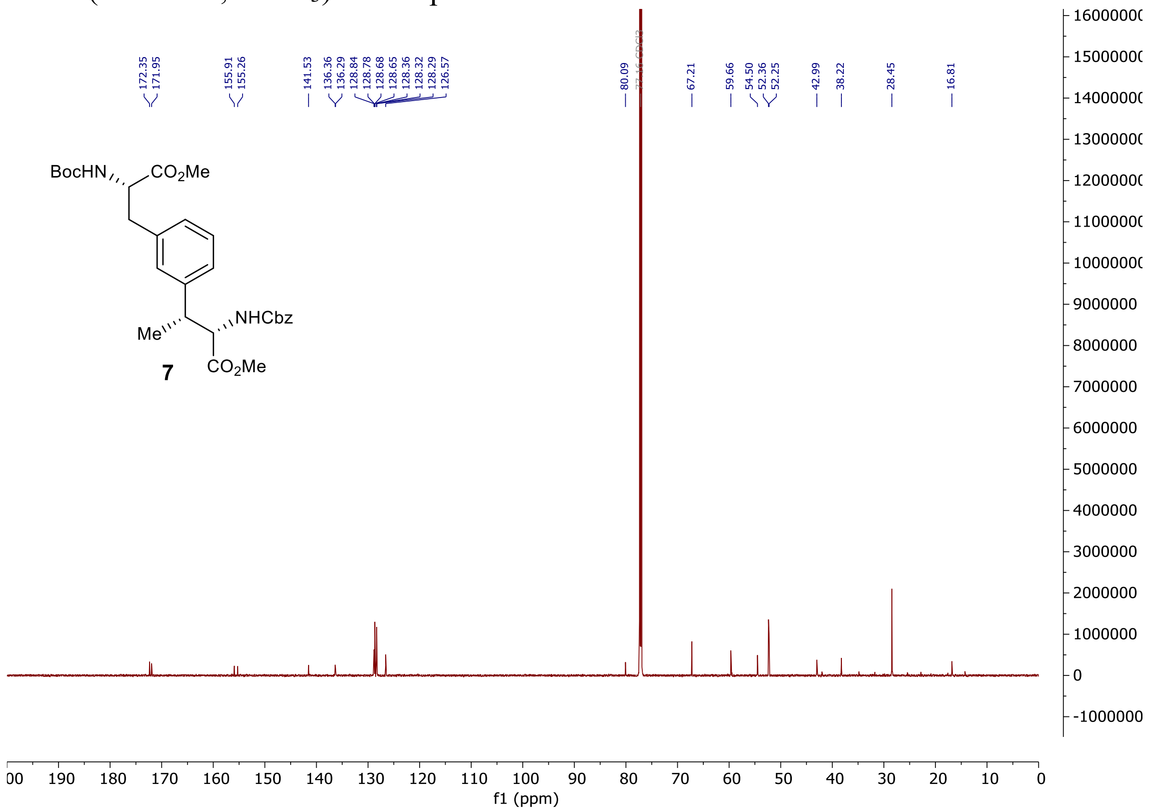

NOESY (600 MHz, $CDCl_3$) of compound **7**

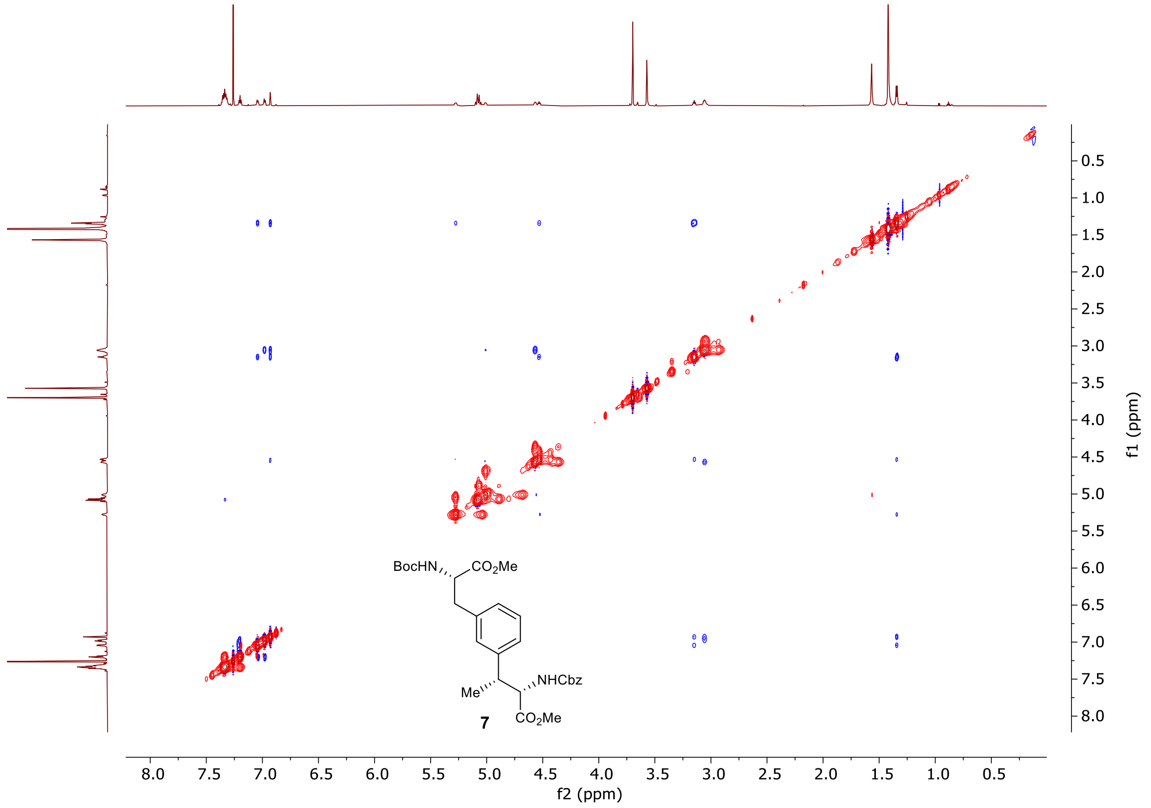

^{1}H NMR (400 MHz, $CDCl_3$, 25 °C) of compound 8b (rotamers visible)

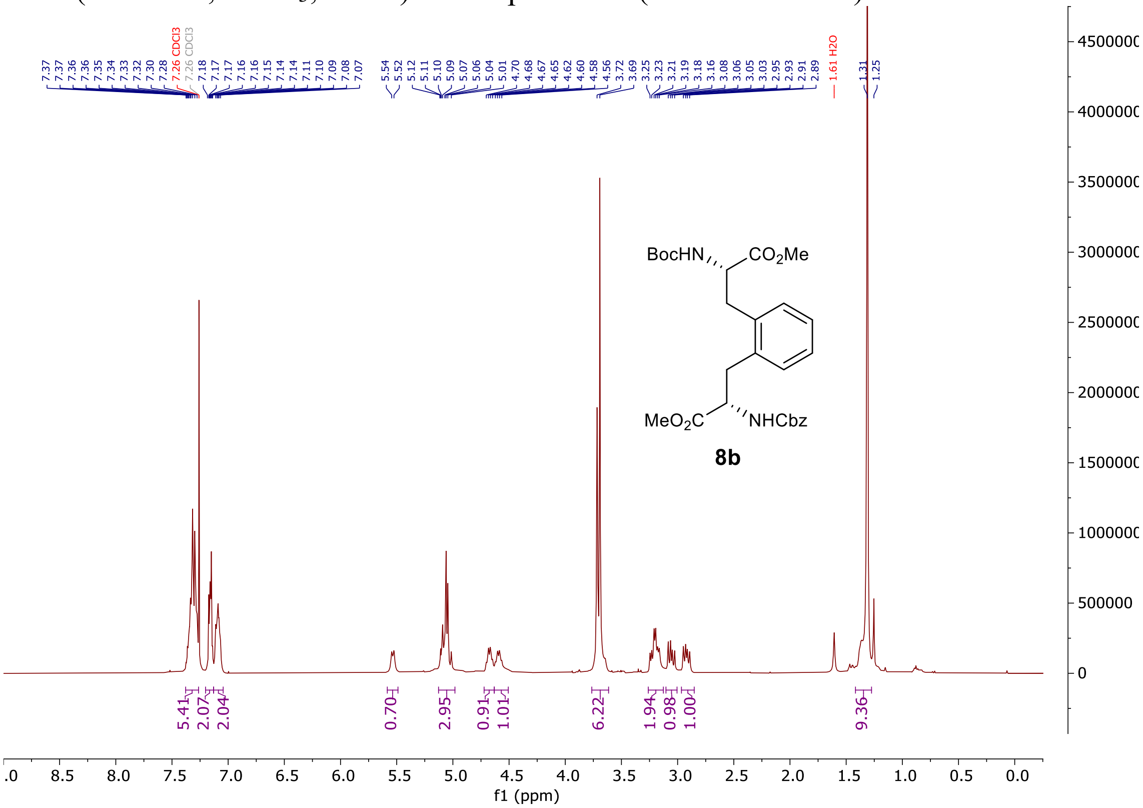

^{1}H NMR (800 MHz, $CDCl_3$, 50 °C) of compound 8b

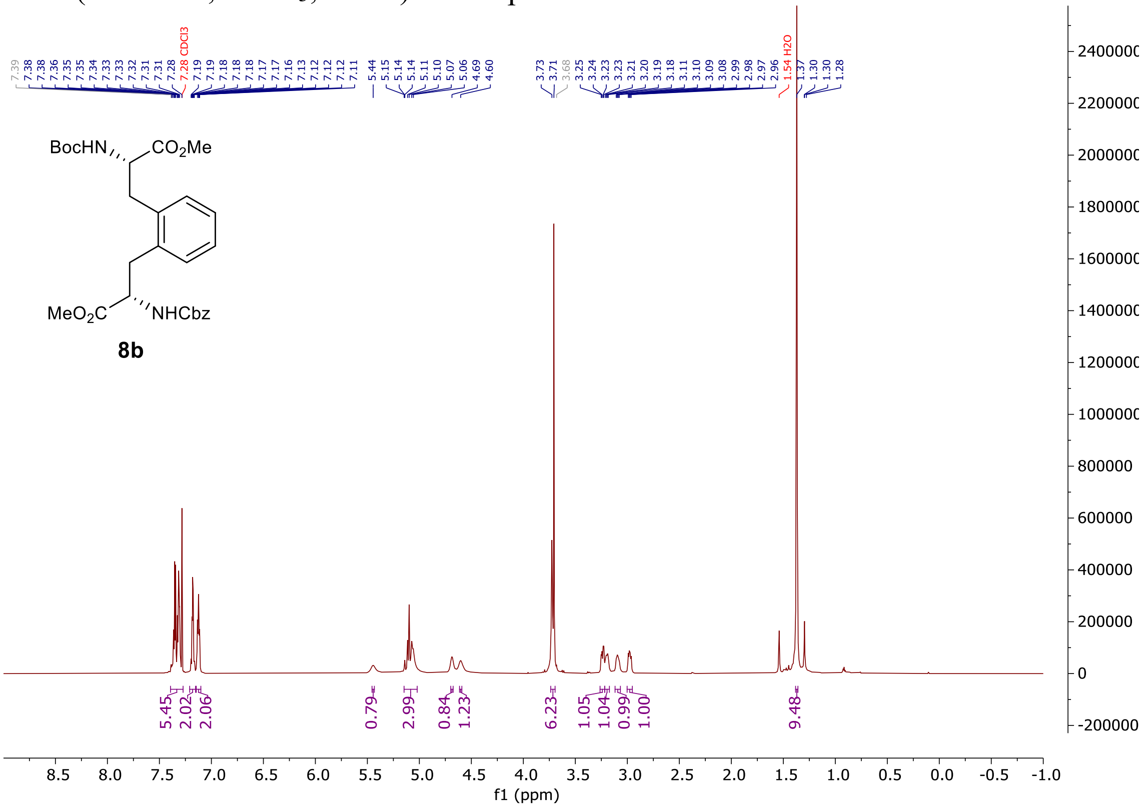

^{13}C NMR (151 MHz, $CDCl_3$) of compound **8b**

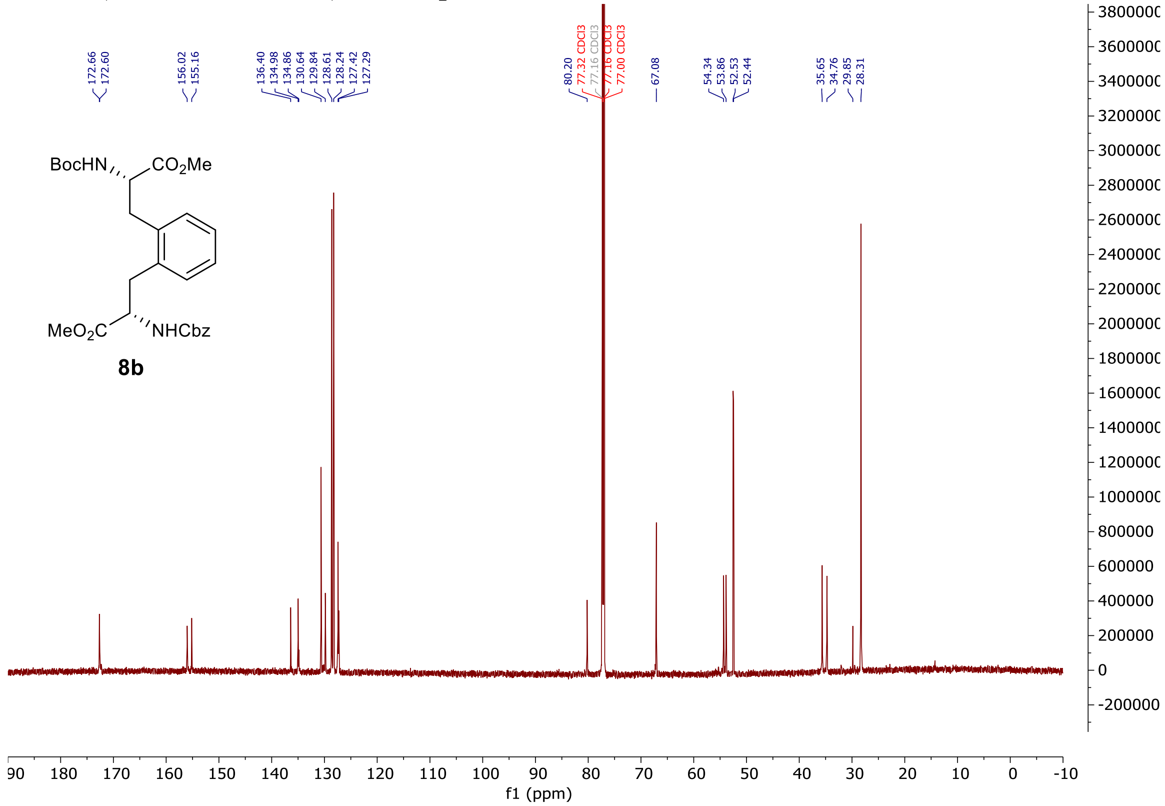

NOESY (800 MHz, $CDCl_3$) of compound **8b**

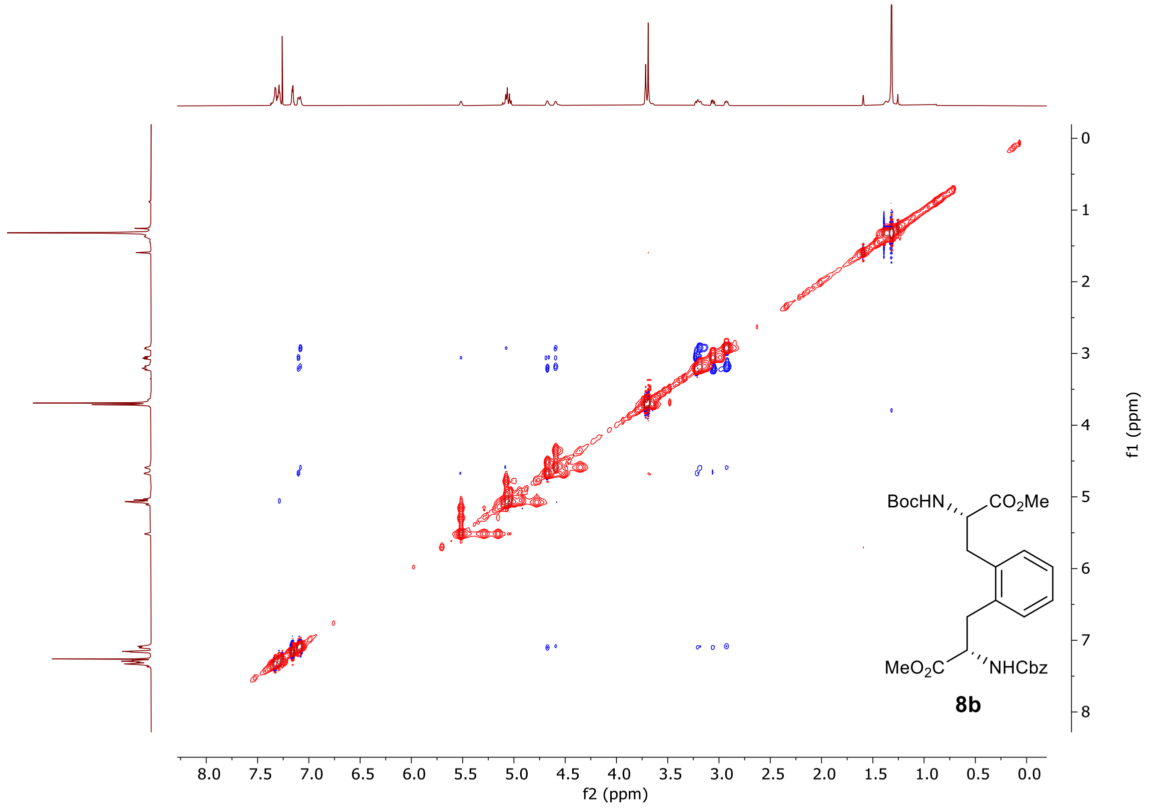

COSY (800 MHz, CDCl3) of compound **8b**

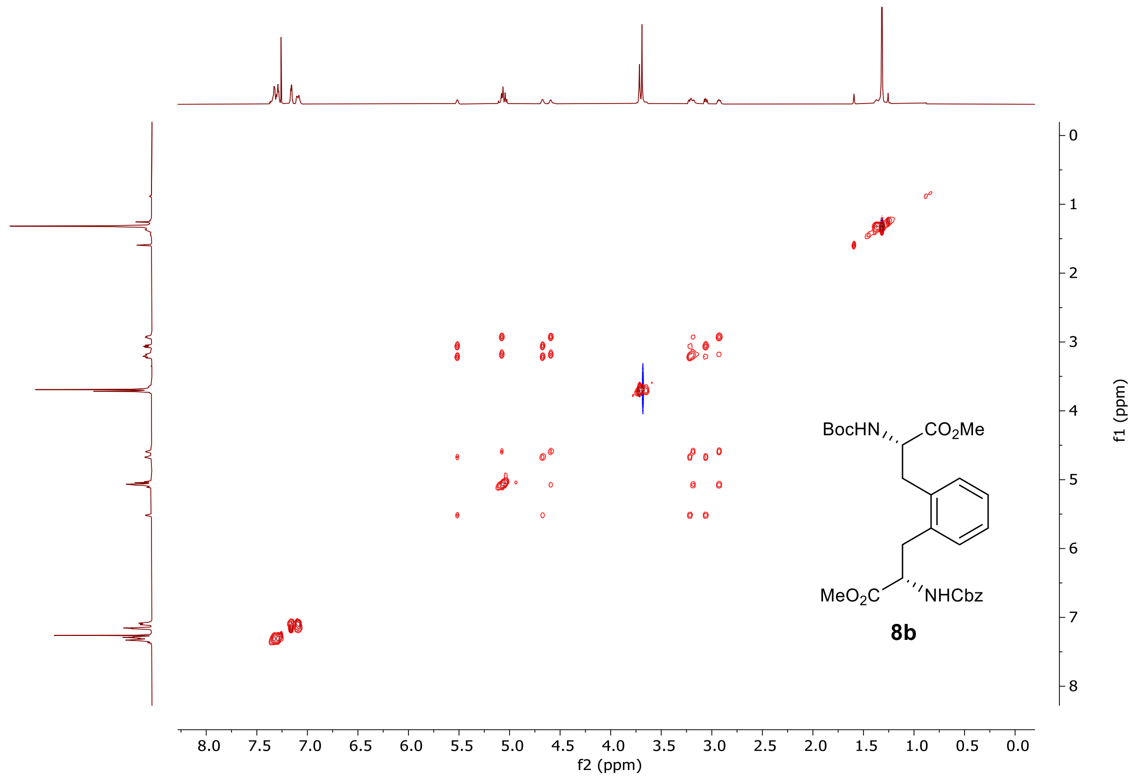

HMBC (800 MHz, CDCl3) of compound **8b**

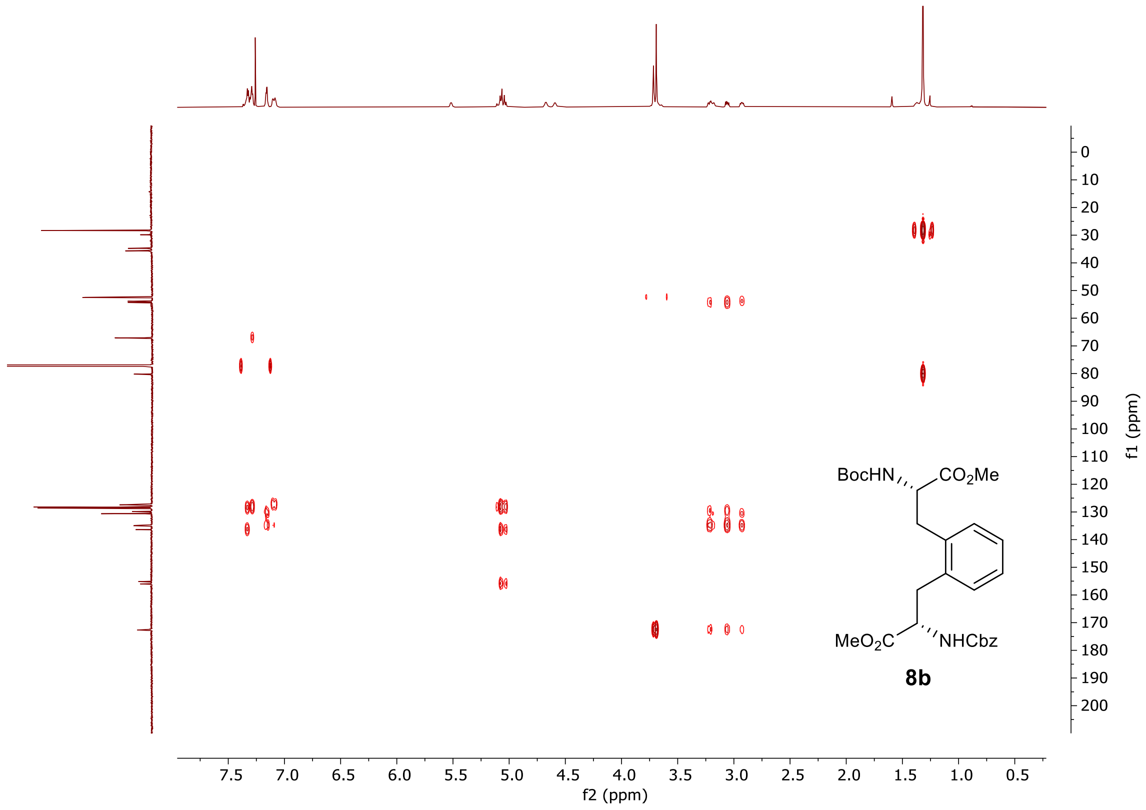

^{1}**H NMR** (800 MHz, $CDCl_3$, 25 °C) of compound **9a** (rotamers visible, 9:1 isolated dr)

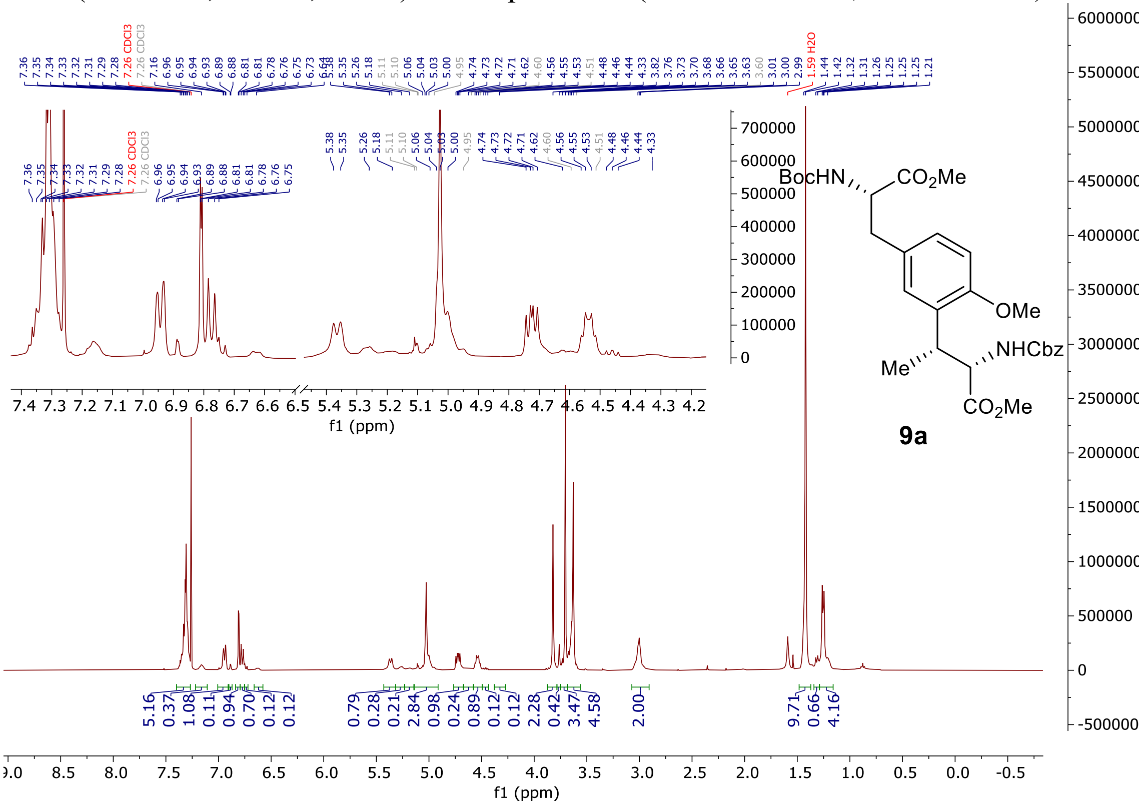

^{1}**H NMR** (800 MHz, $CDCl_3$, 50 °C) of compound **9a**

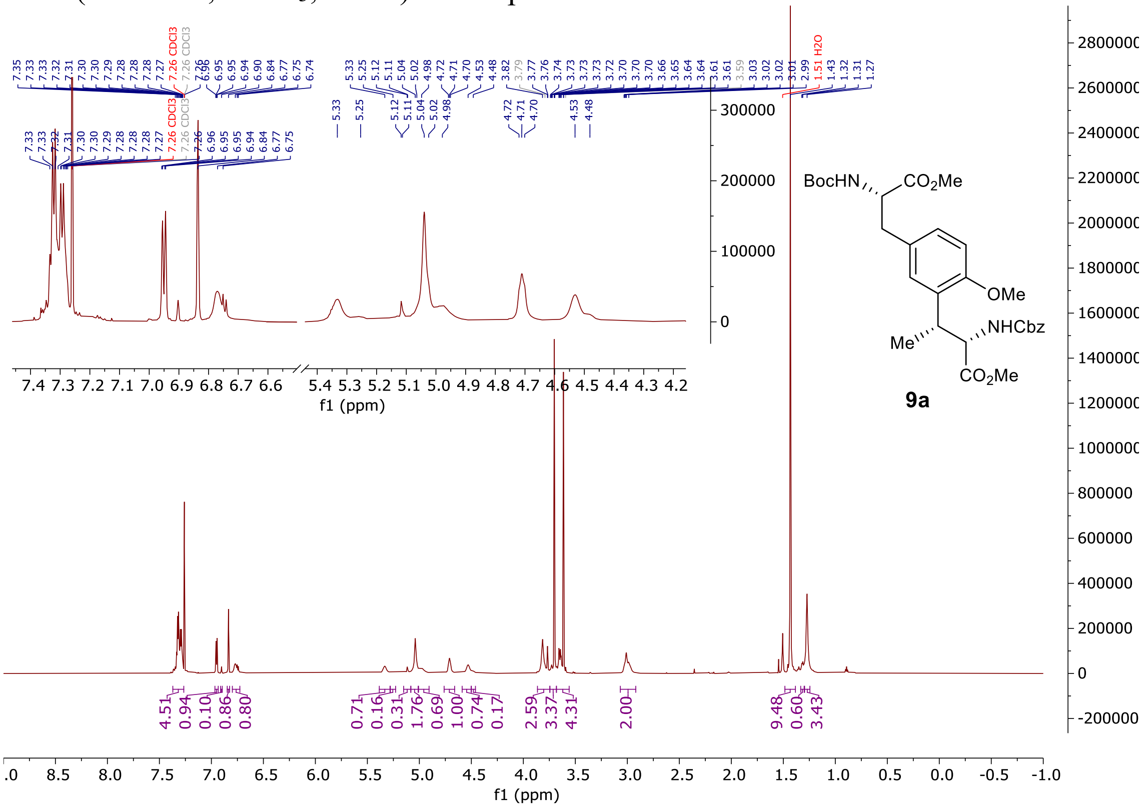

^{13}C NMR (201 MHz, $CDCl_3$) of compound **9a**

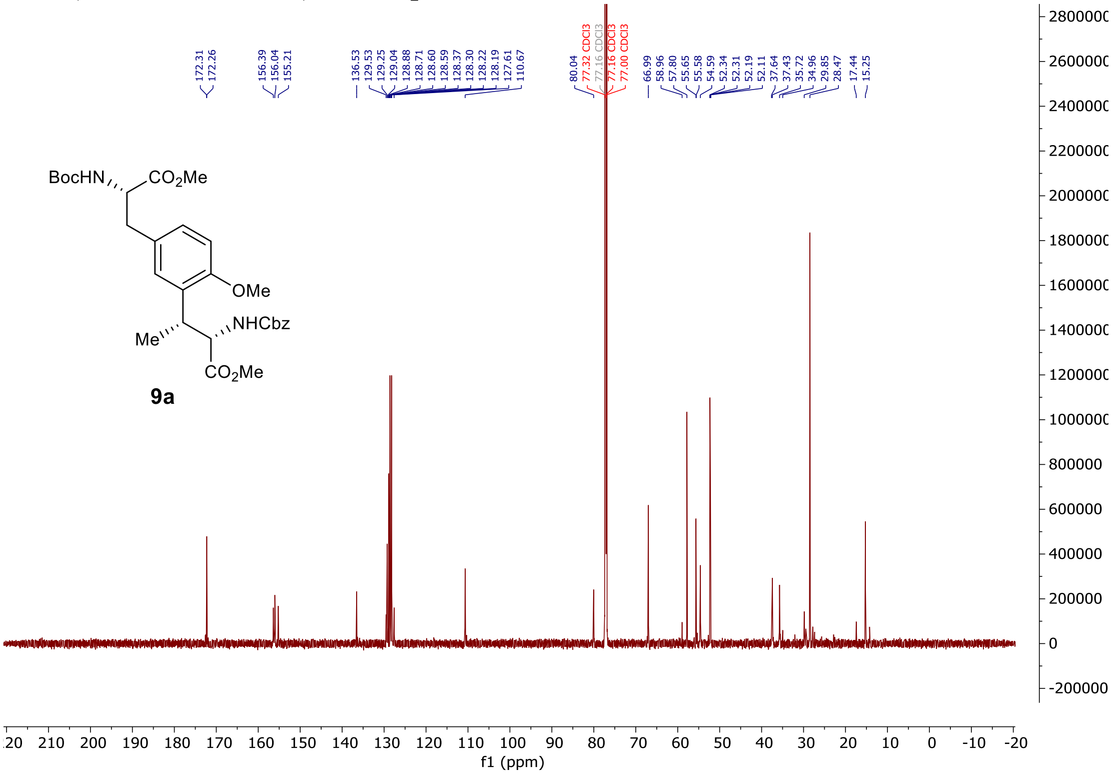

^{1}H NMR (600 MHz, $CDCl_3$, 25 °C) of compound **9b**

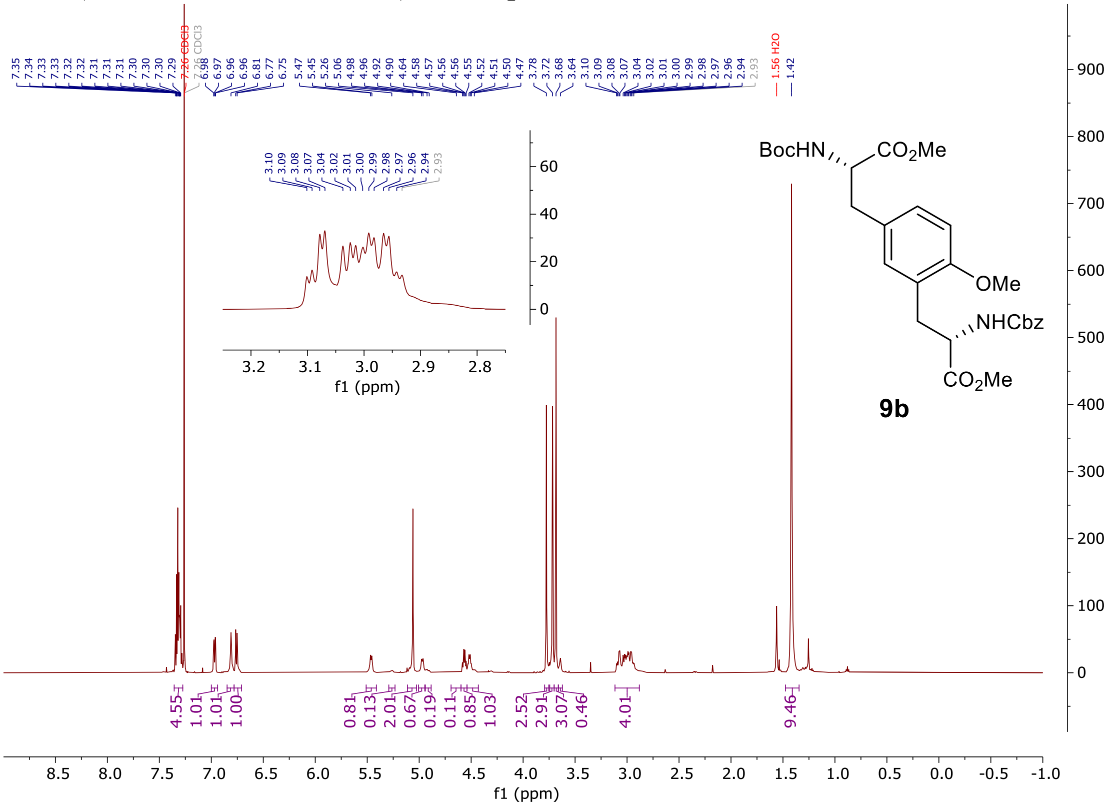

^{1}H NMR (600 MHz, $CDCl_3$, 50 °C) of compound **9b**

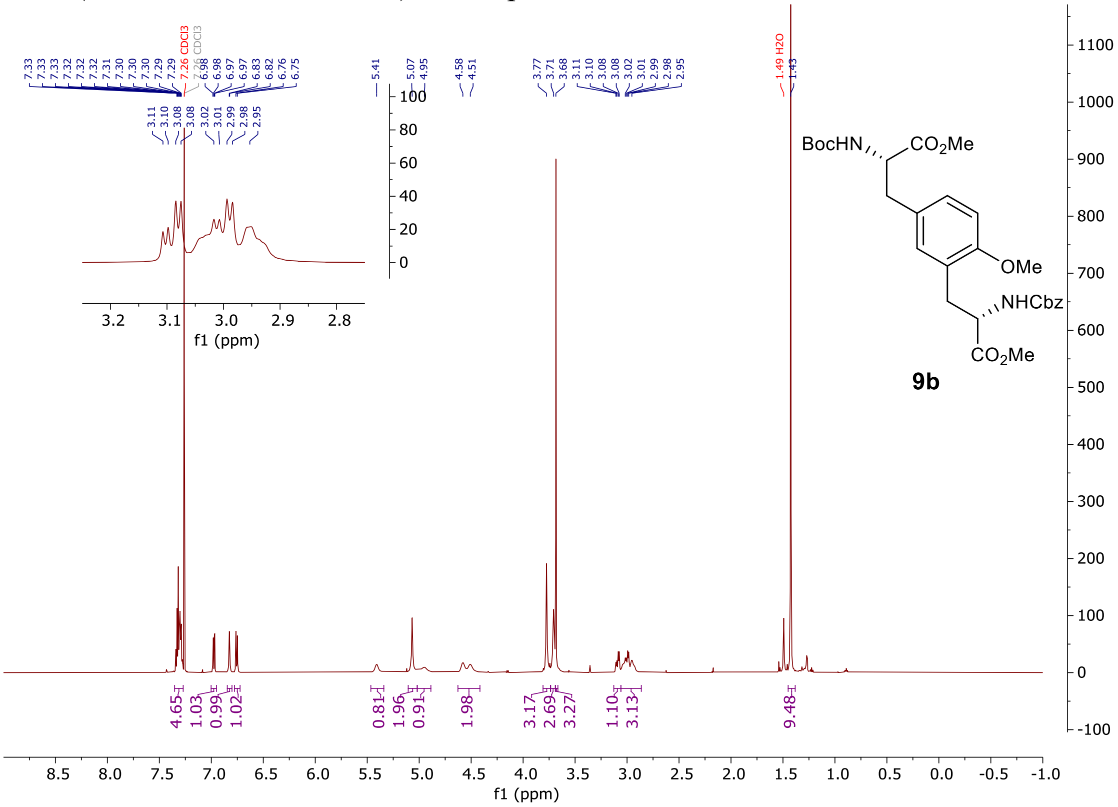

^{13}C NMR (201 MHz, $CDCl_3$) of compound **9b**

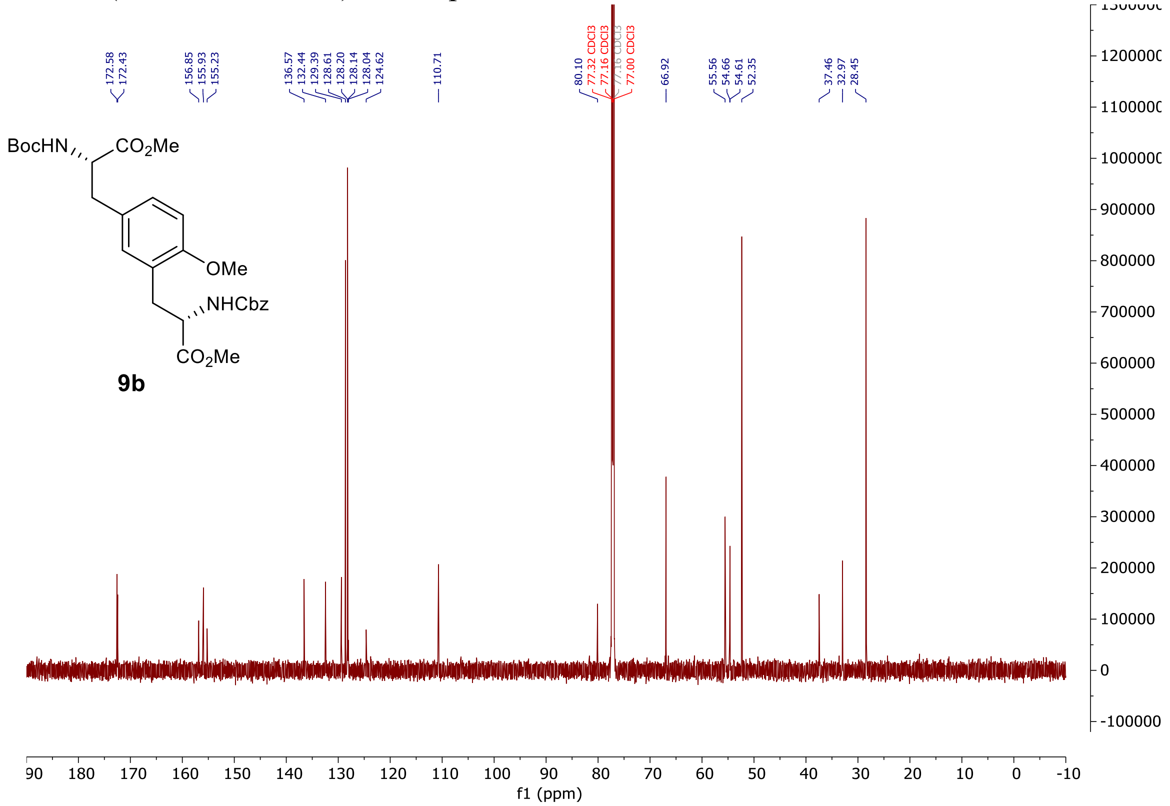

NOESY (800 MHz, $CDCl_3$) of compound **9b**

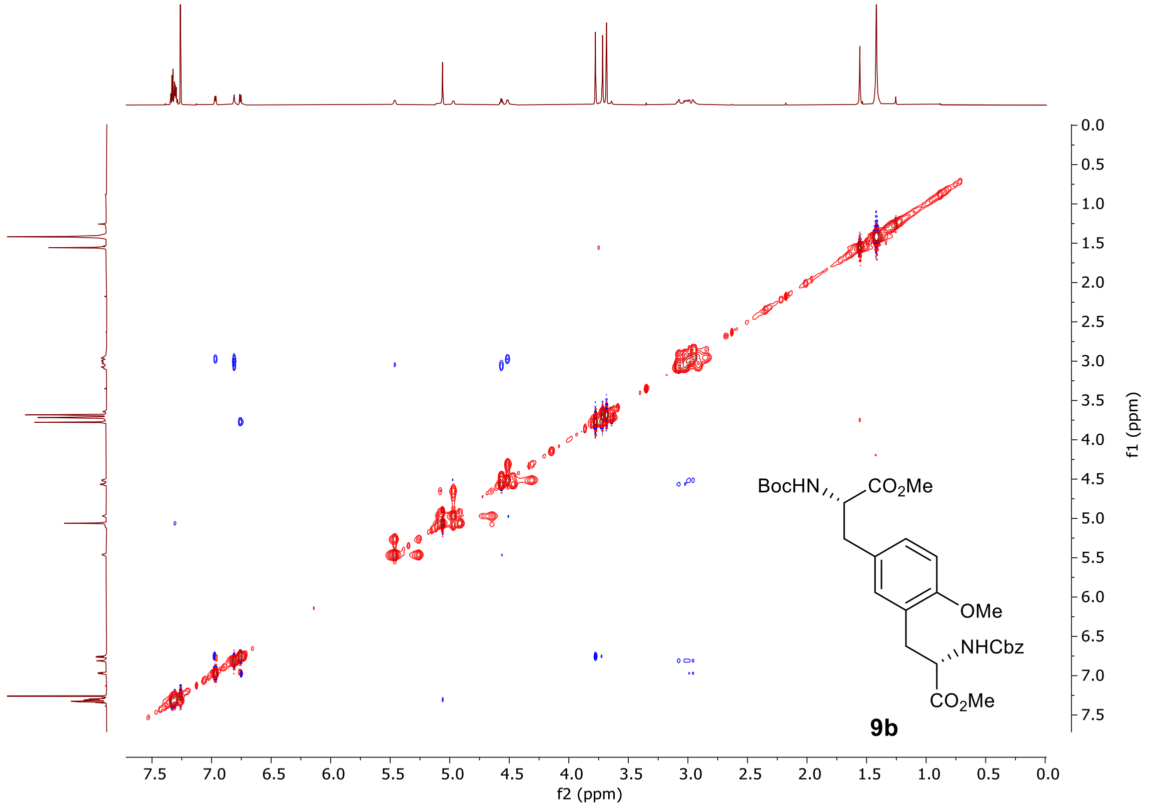

HMBC (800 MHz, $CDCl_3$) of compound **9b**

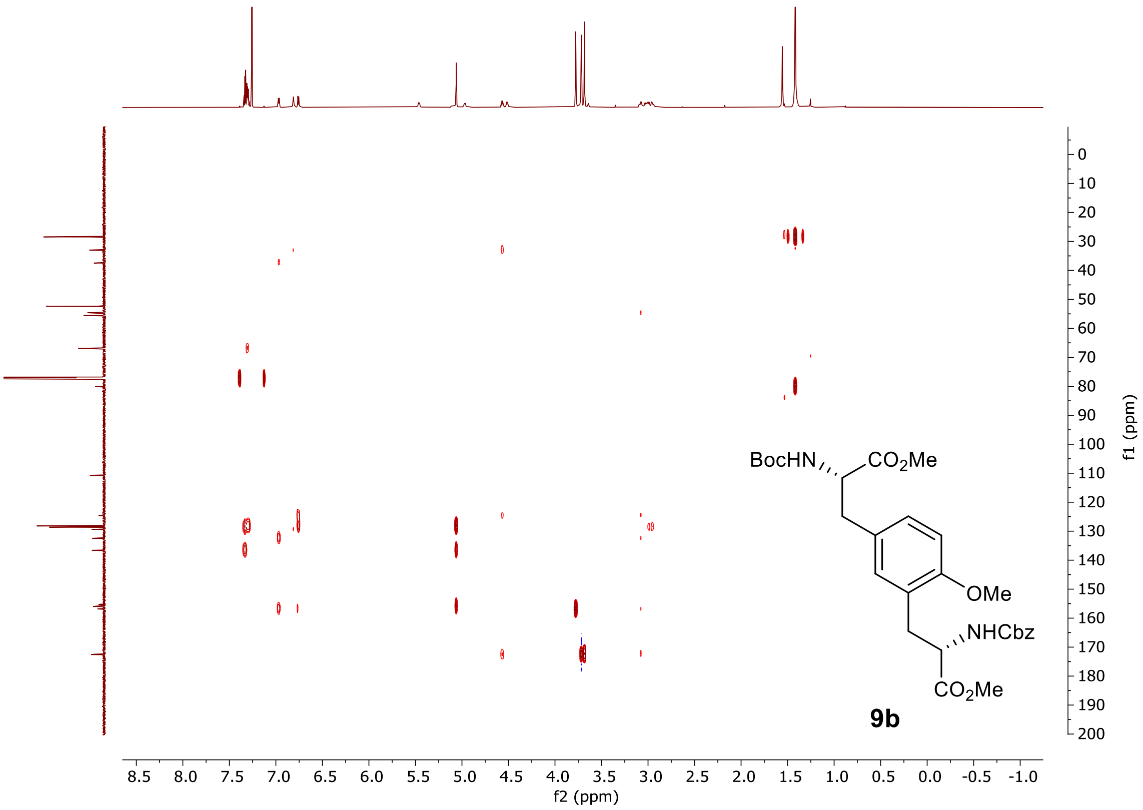

^{1}H NMR (800 MHz, $CDCl_3$, 25 °C) of compound **10** (rotamers visible, 15:1 isolated dr)

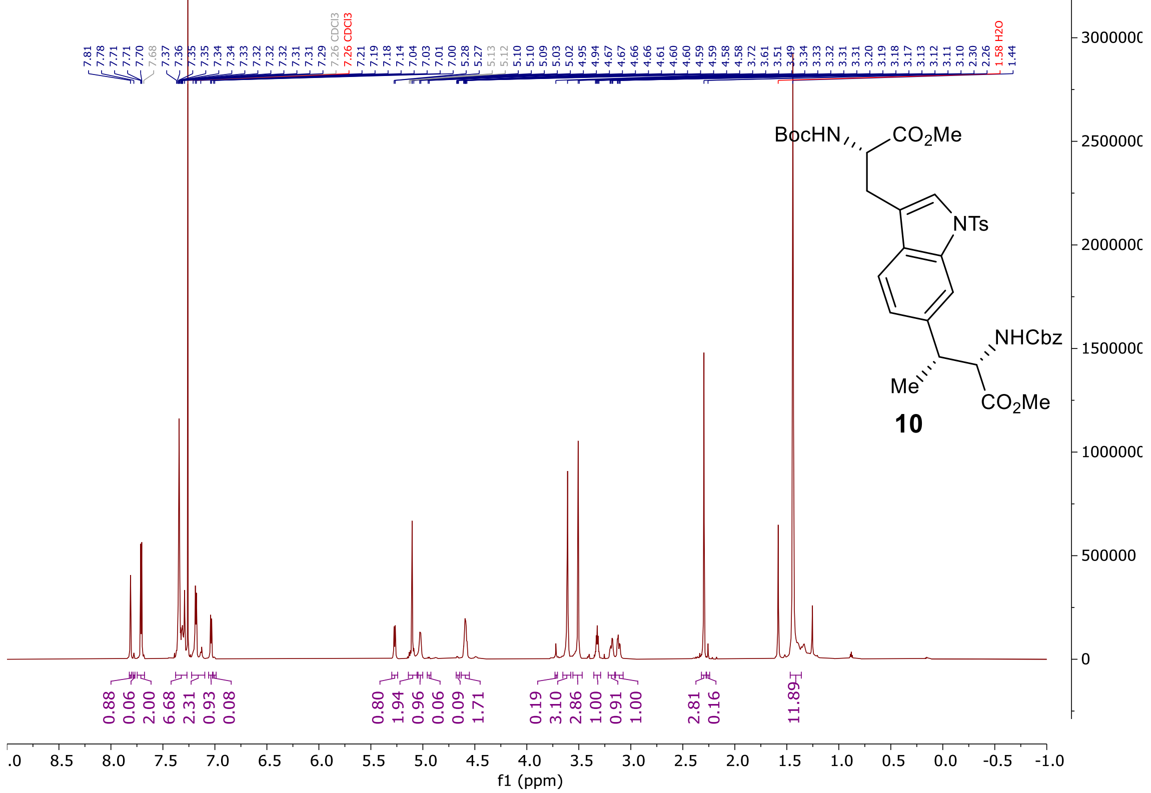

^{13}C NMR (201 MHz, $CDCl_3$) of compound **10**

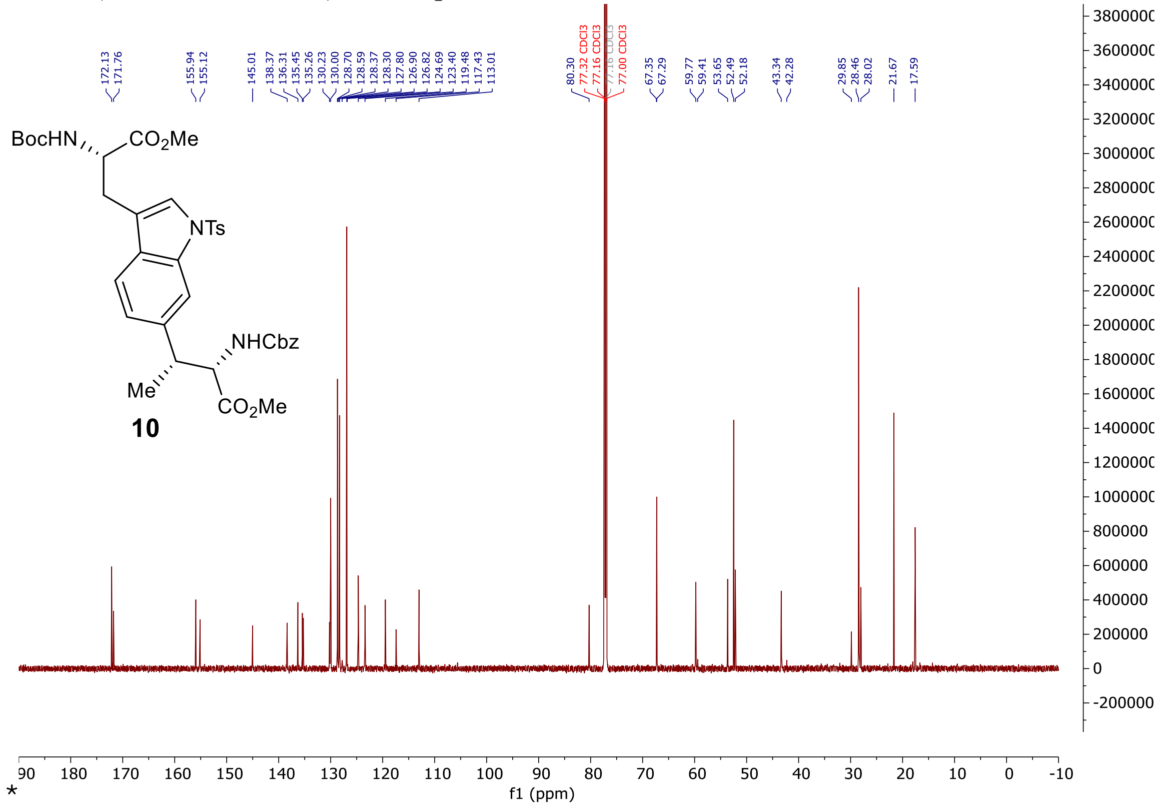

COSY (800 MHz, $CDCl_3$) of compound **10**

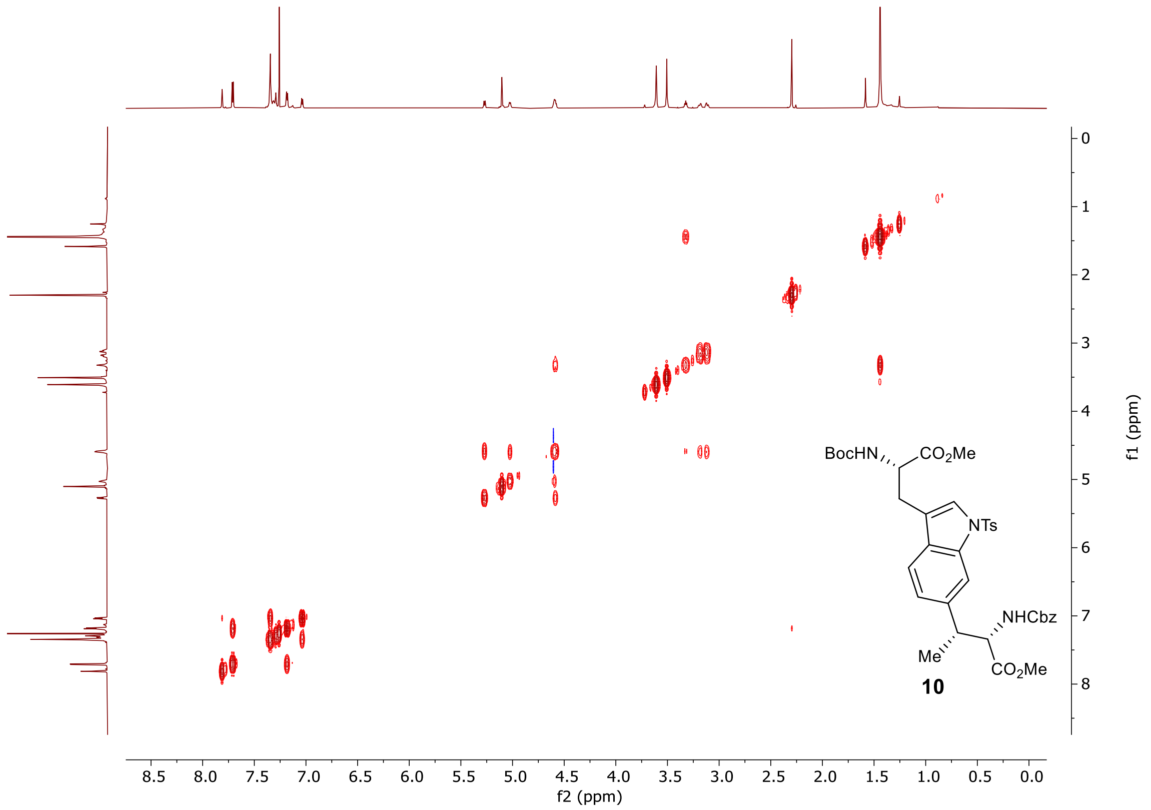

NOESY (800 MHz, $CDCl_3$) of compound **10**

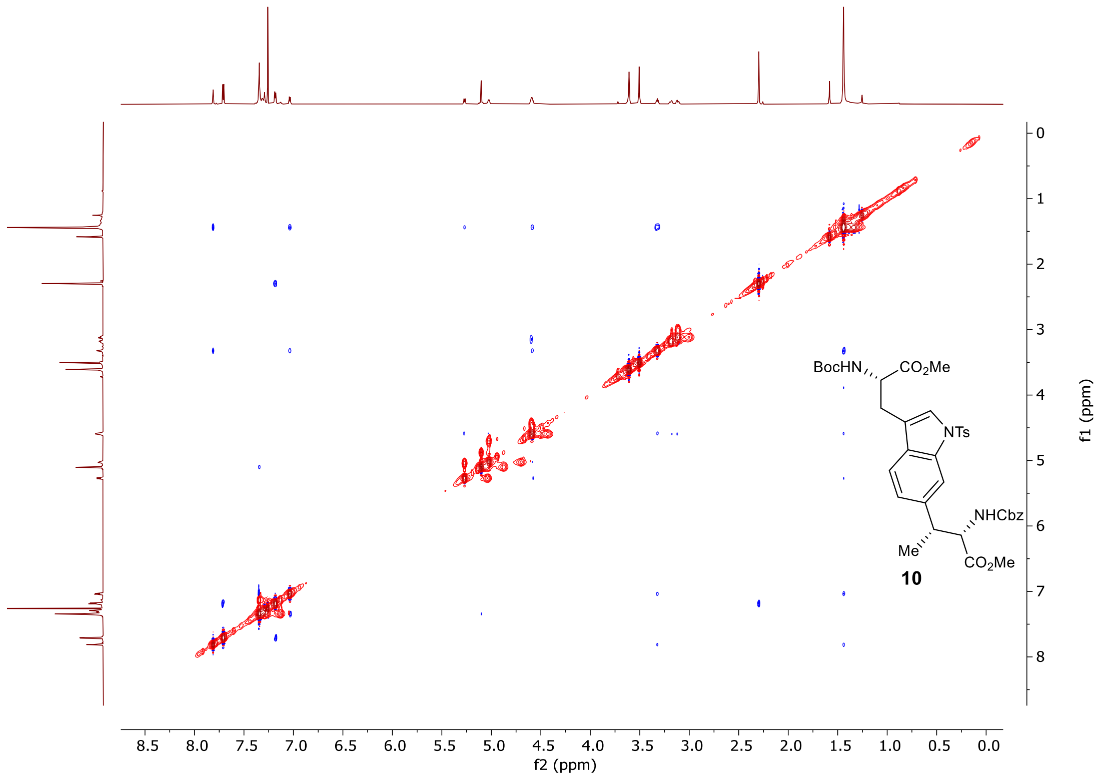

HMBC (800 MHz, CDCl$_3$) of compound **10**

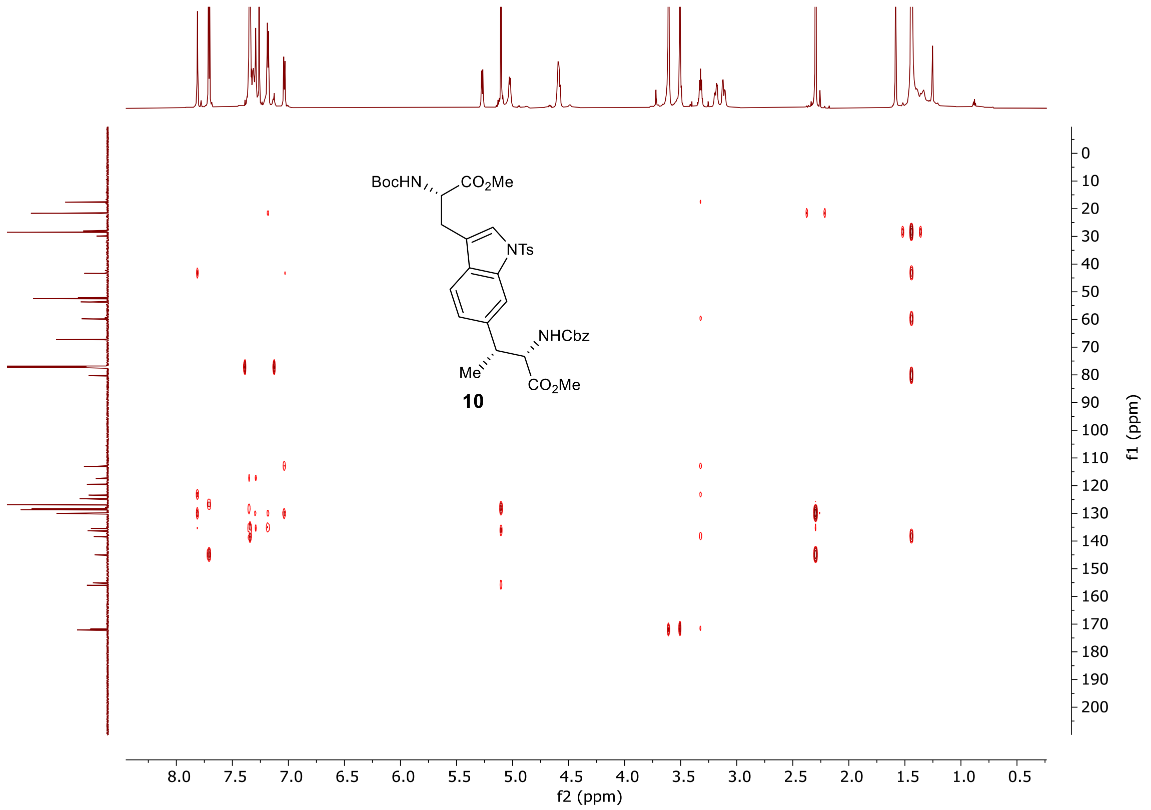

^{1}**H NMR** (400 MHz, $CDCl_3$, 25 °C) of compound **11** (rotamers visible, 6:1 isolated dr)

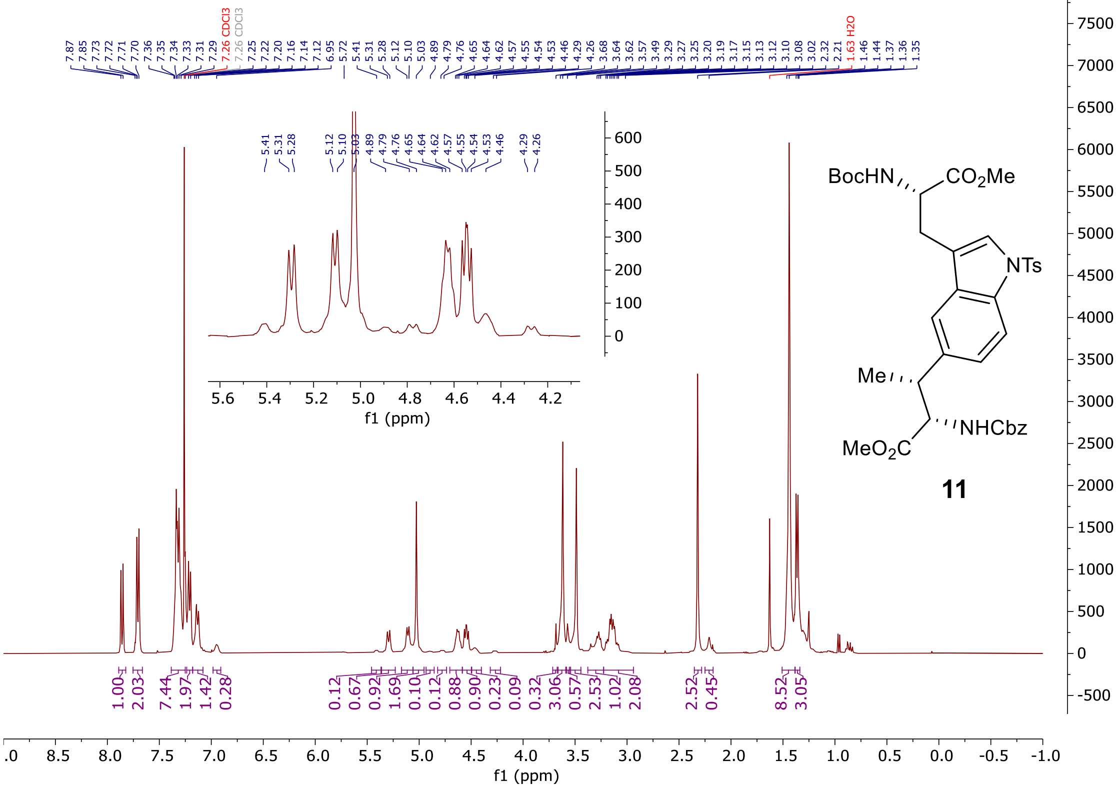

^{1}**H NMR** (600 MHz, $CDCl_3$, 50 °C) of compound **11**

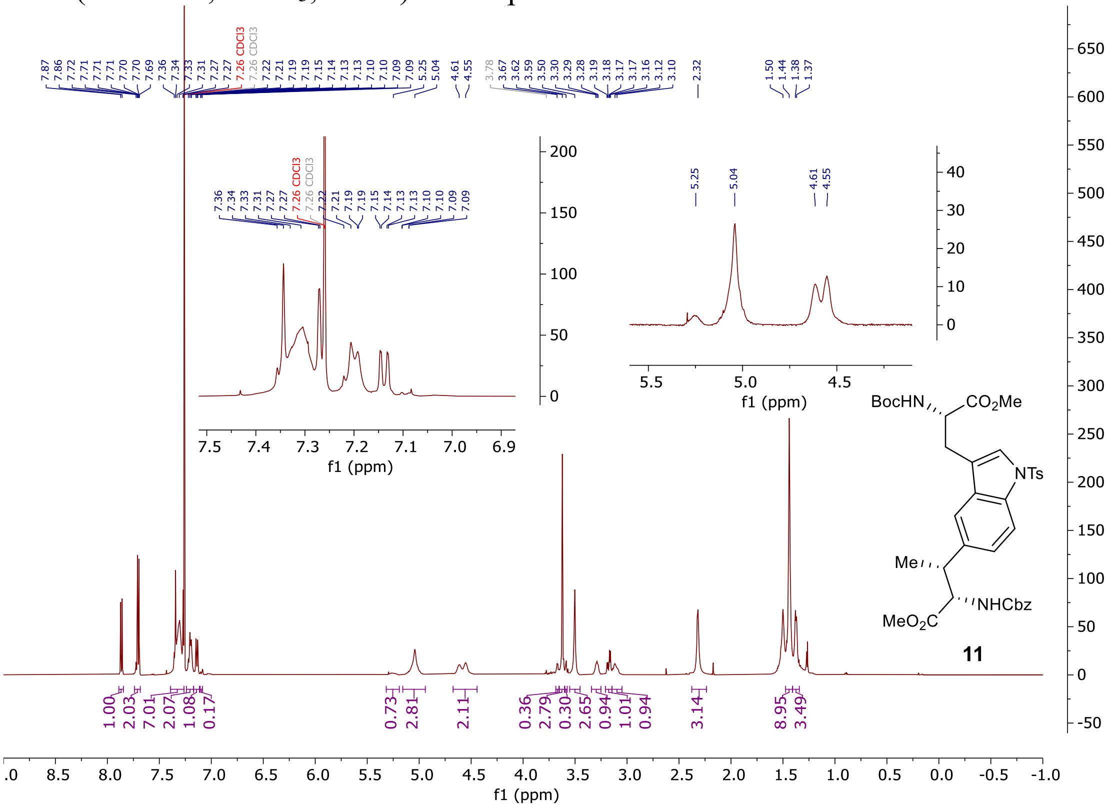

^{13}C NMR (201 MHz, $CDCl_3$) of compound **11**

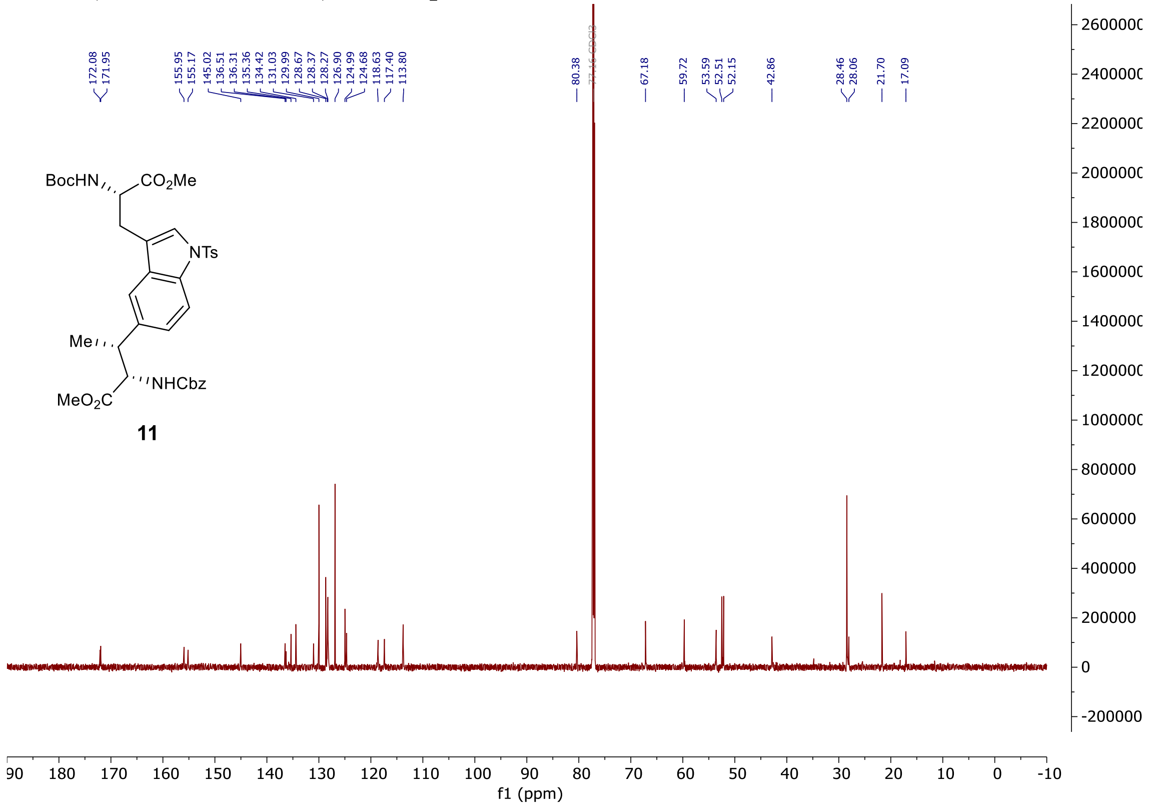

COSY (800 MHz, $CDCl_3$) of compound **11**

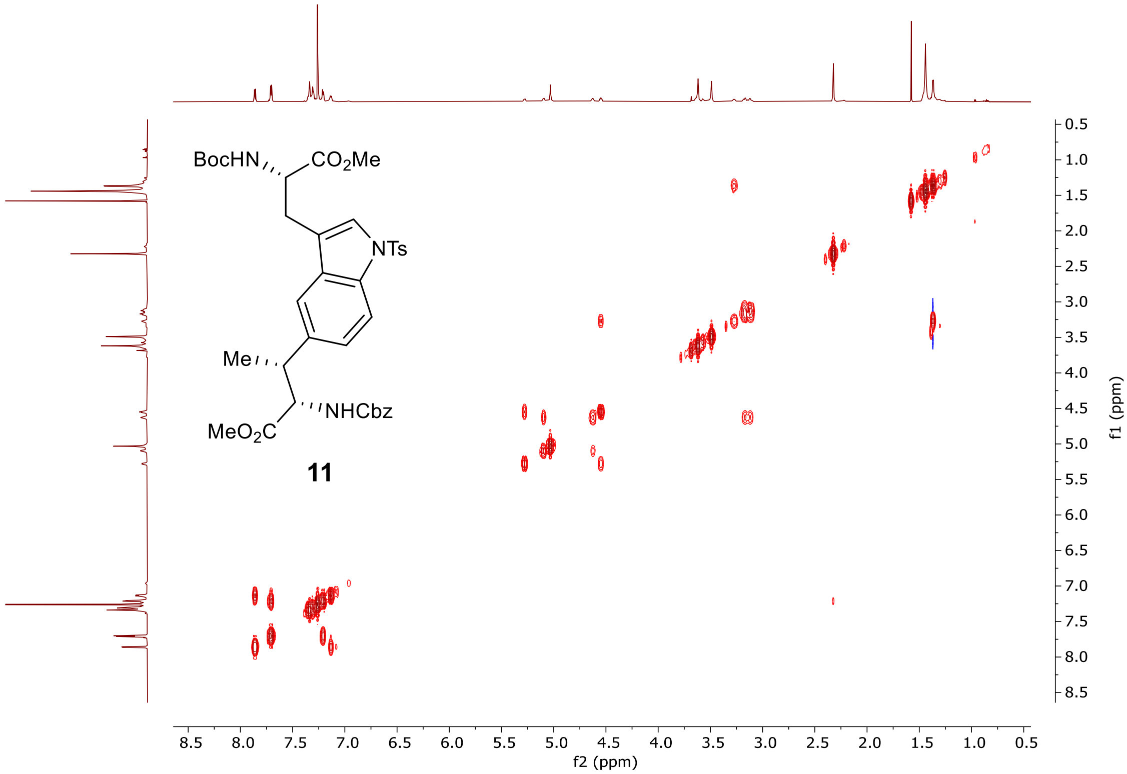

NOESY (800 MHz, $CDCl_3$) of compound **11**

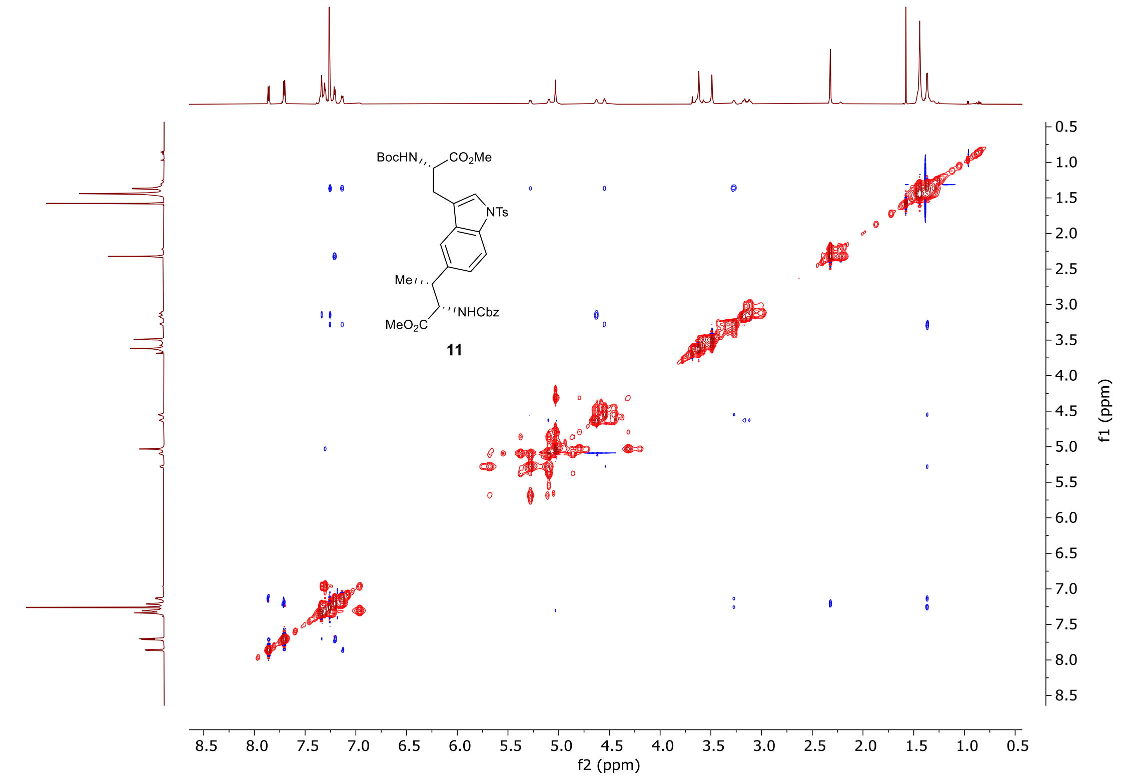

HMBC (800 MHz, $CDCl_3$) of compound **11**

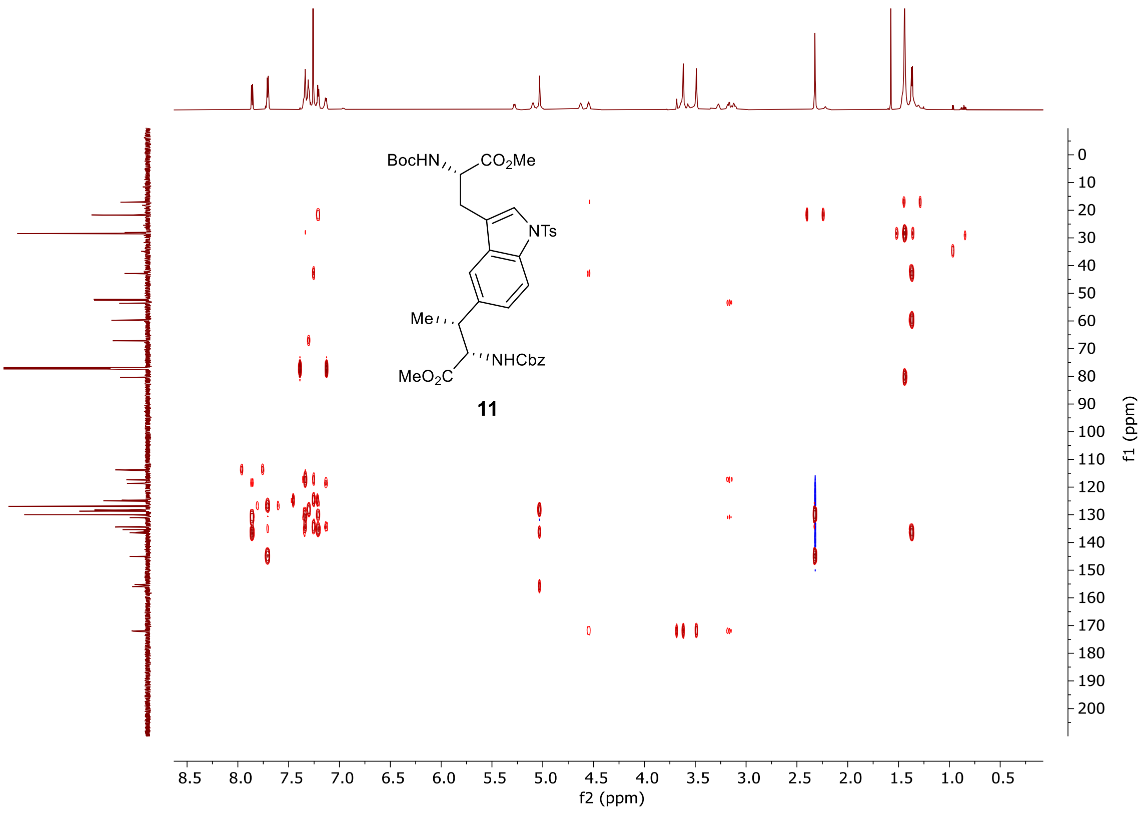

^{1}**H NMR** (400 MHz, $CDCl_3$, 25 °C) of compound **12** (14:1 isolated dr)

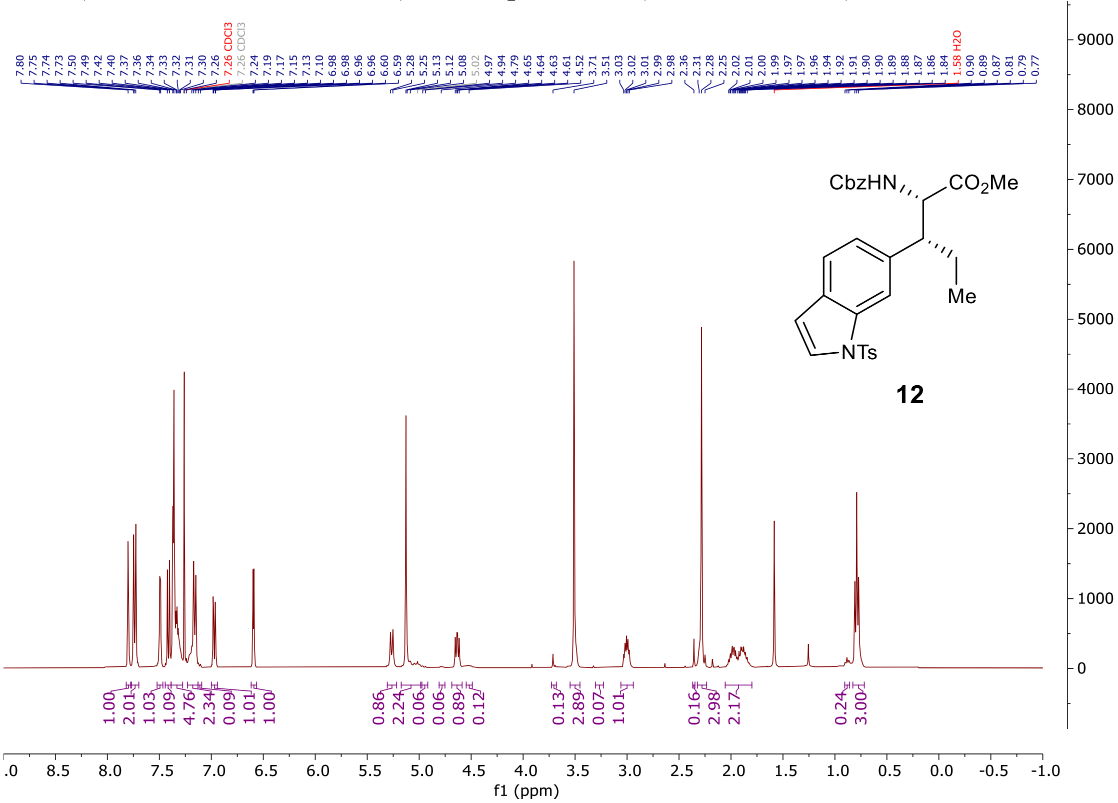

^{13}**C NMR** (201 MHz, $CDCl_3$) of compound **12**

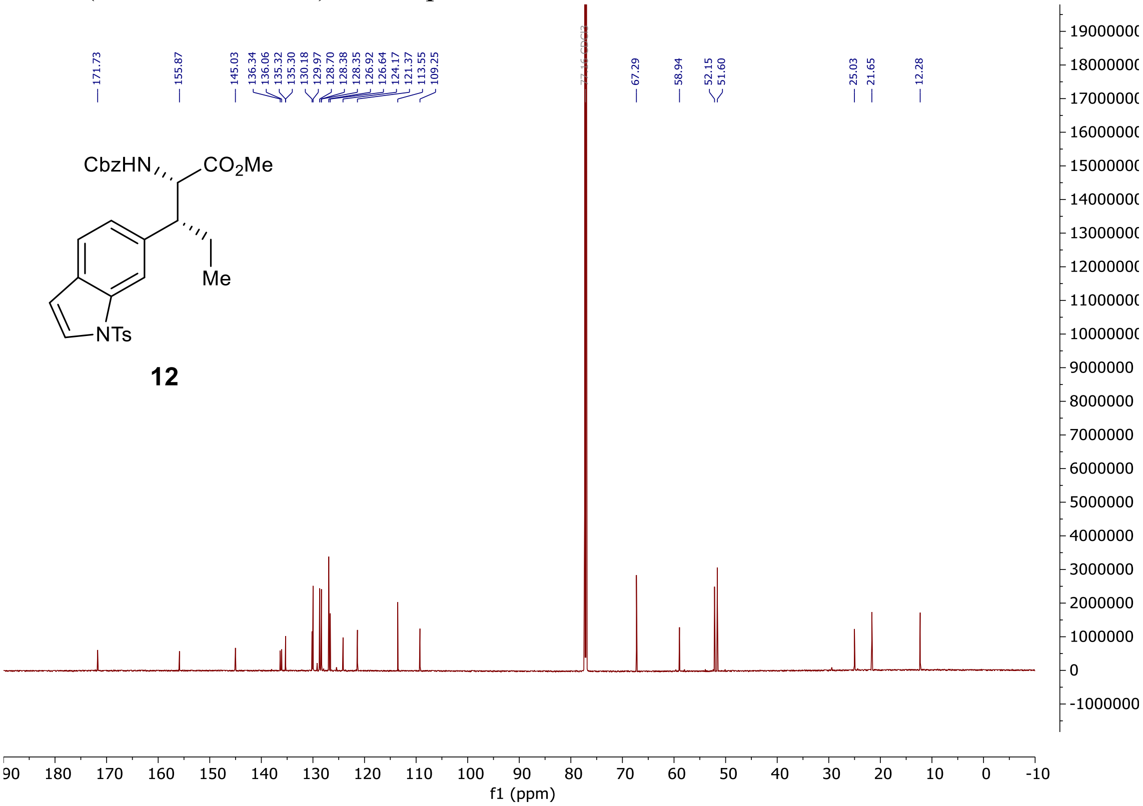

NOESY (800 MHz, $CDCl_3$) of compound **12**

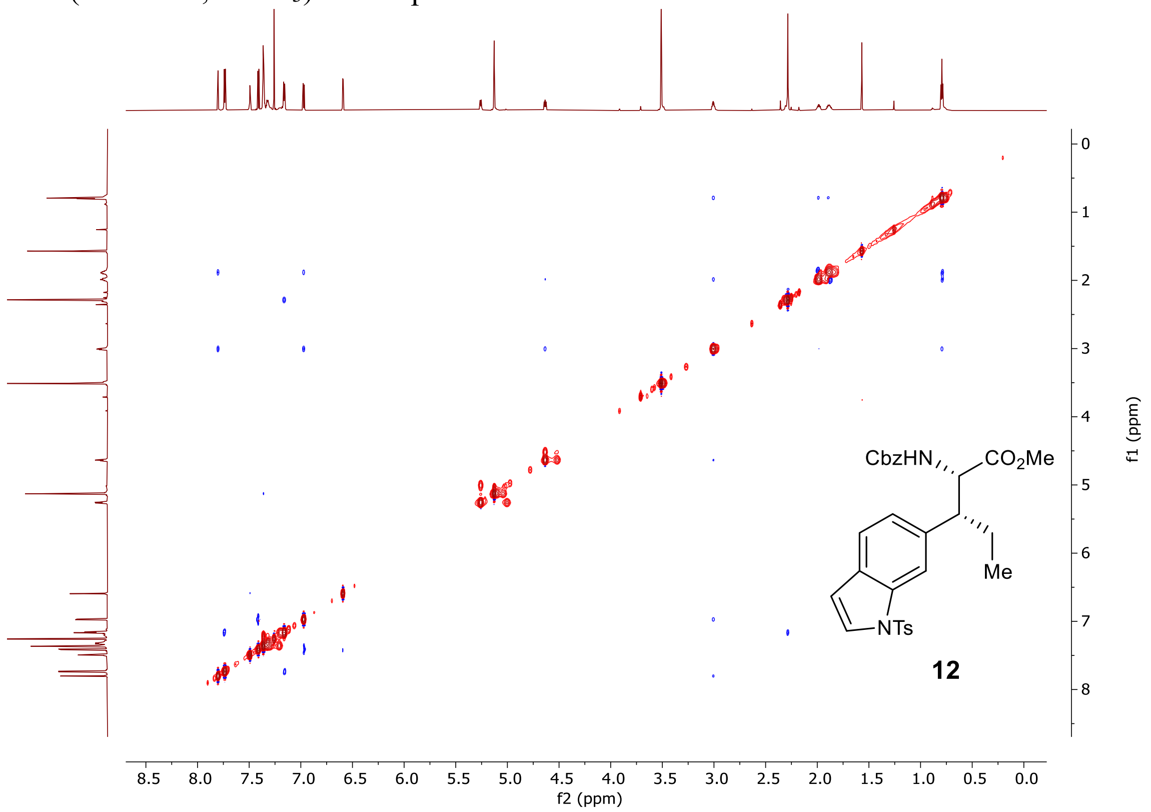

HMBC (800 MHz, $CDCl_3$) of compound **12**

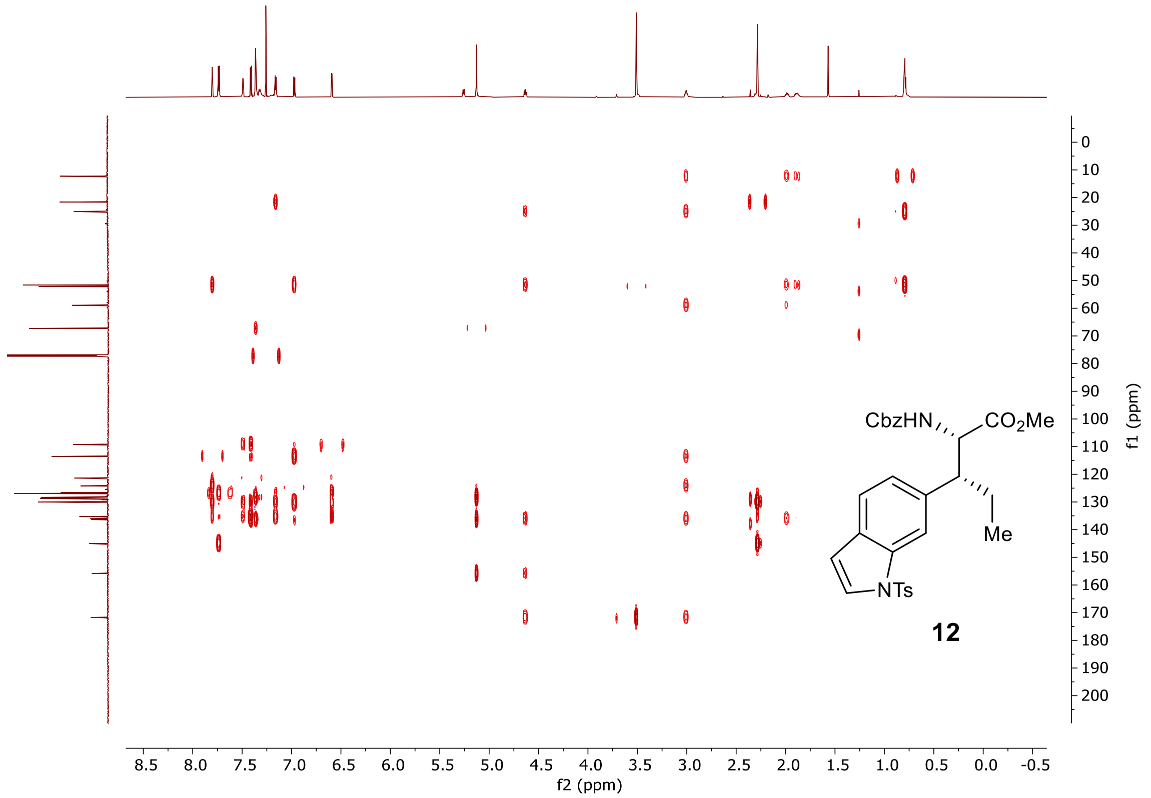

^{1}H NMR (600 MHz, $CDCl_3$, 25 °C) of compound **13** (14:1 isolated dr)

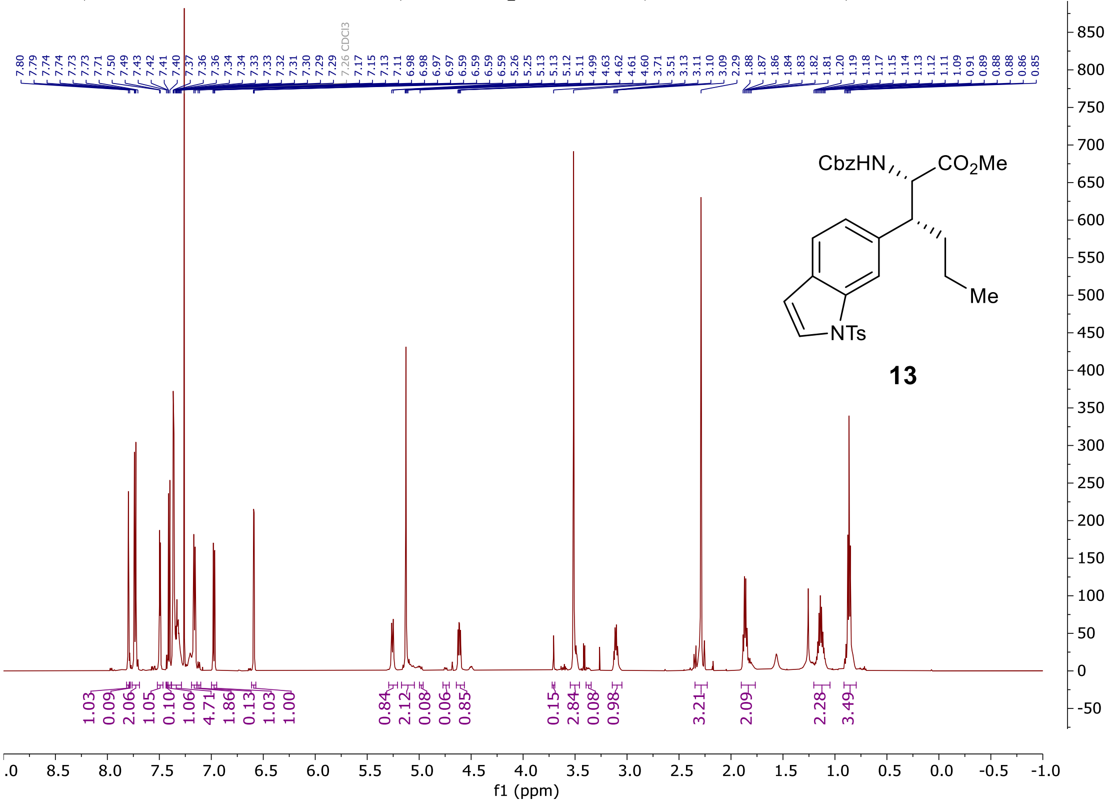

^{13}C NMR (151 MHz, $CDCl_3$) of compound **13**

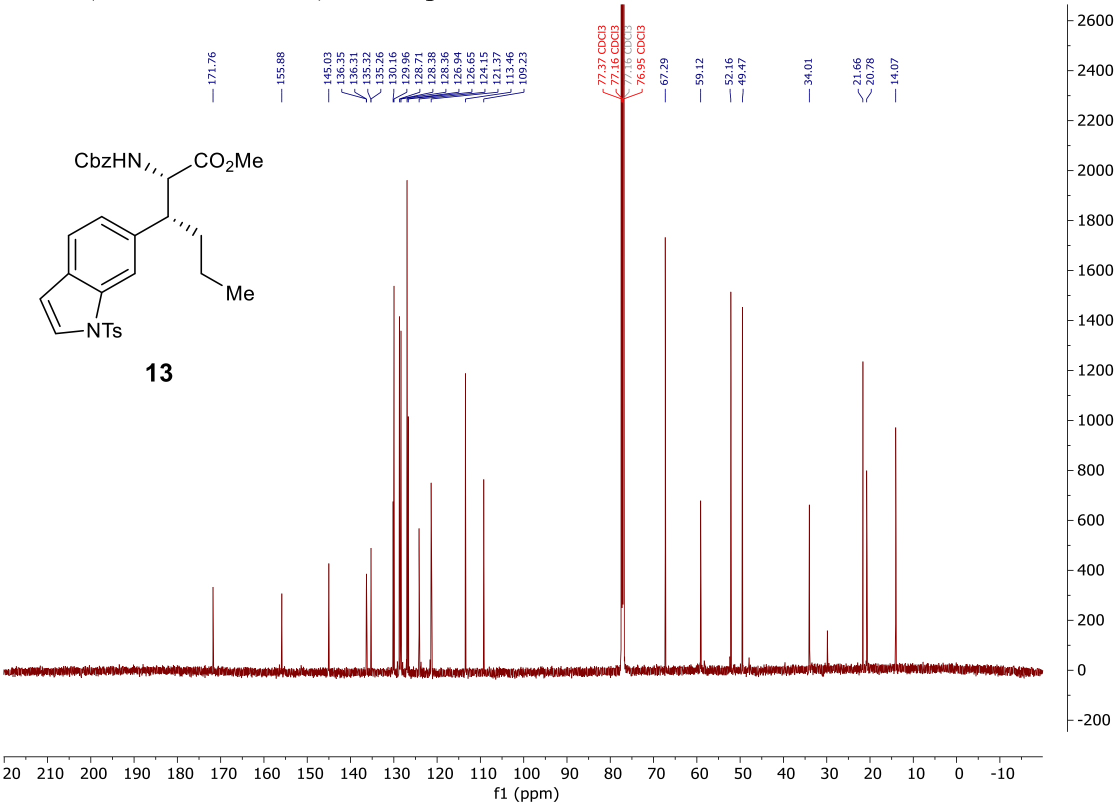

^{1}H NMR (800 MHz, $CDCl_3$, 25 °C) of compound **14** (7:1 isolated dr)

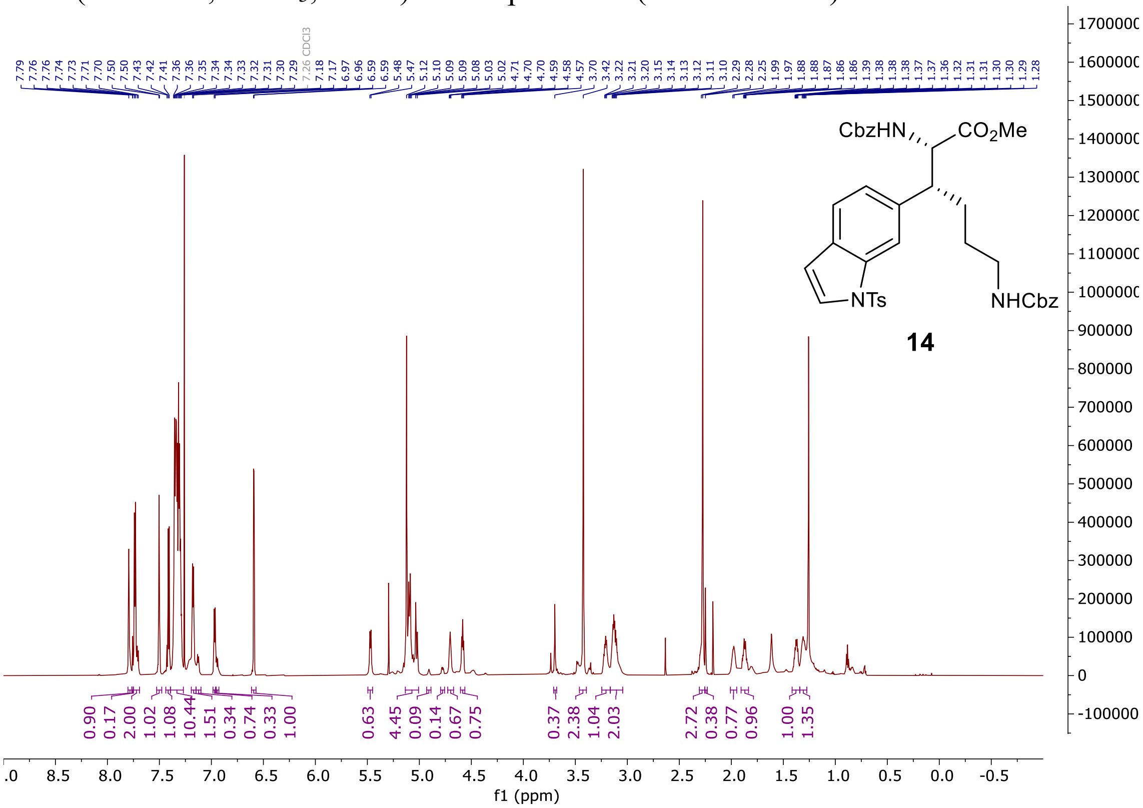

^{13}C NMR (201 MHz, $CDCl_3$) of compound **14**

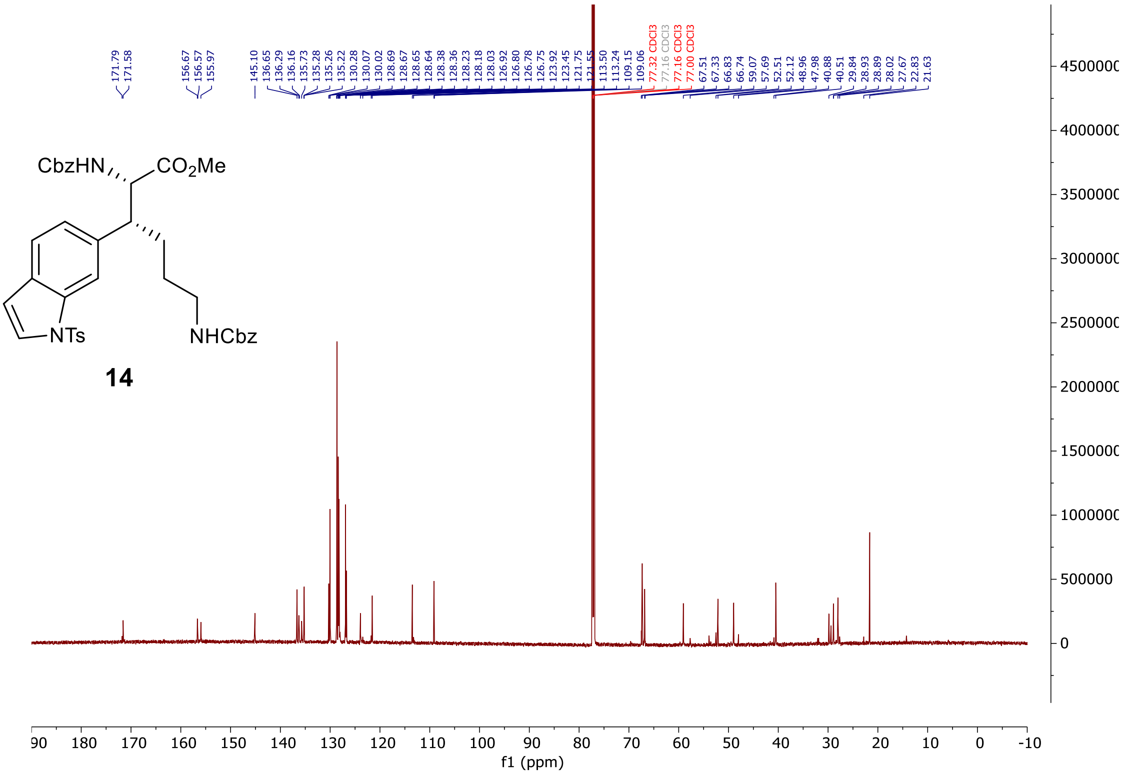

COSY (800 MHz, $CDCl_3$) of compound **14**

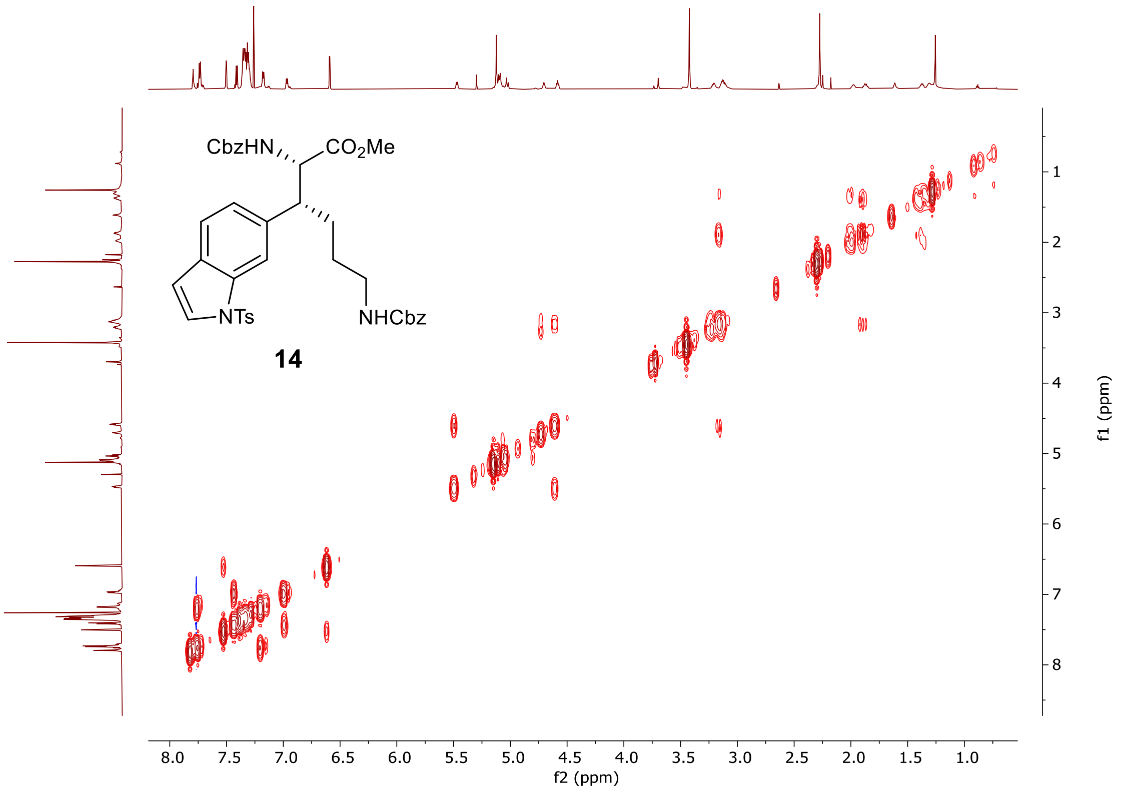

NOESY (800 MHz, $CDCl_3$) of compound **14**

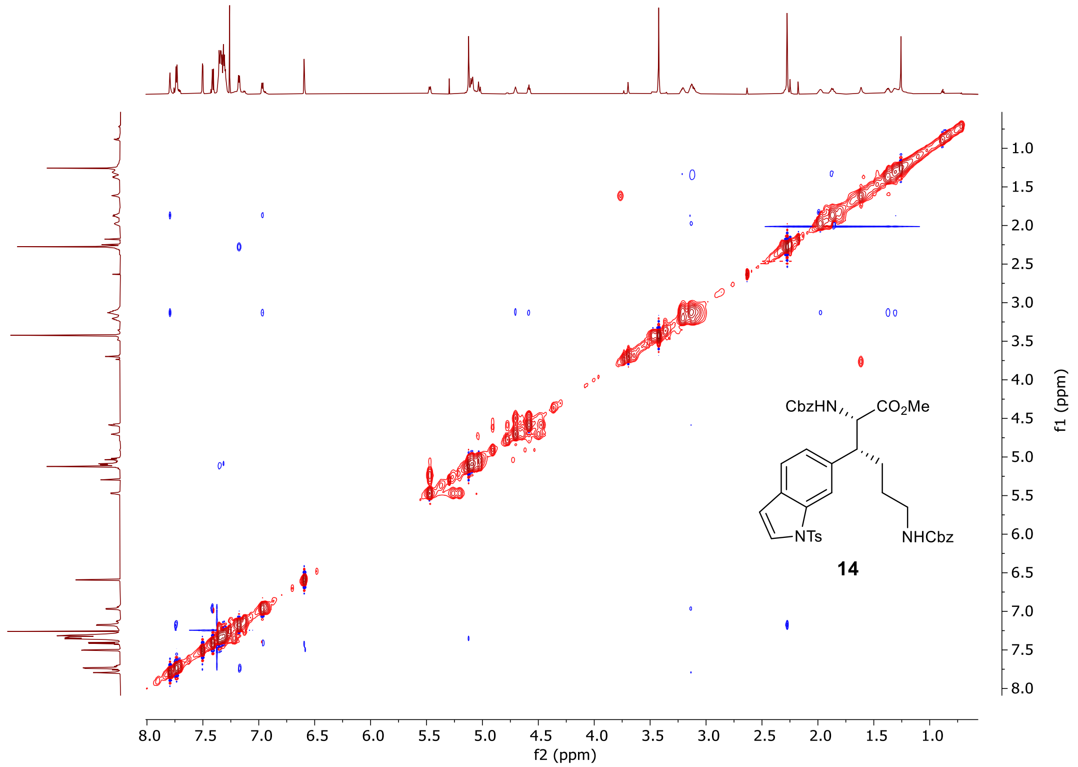

HMBC (800 MHz, $CDCl_3$) of compound **14**

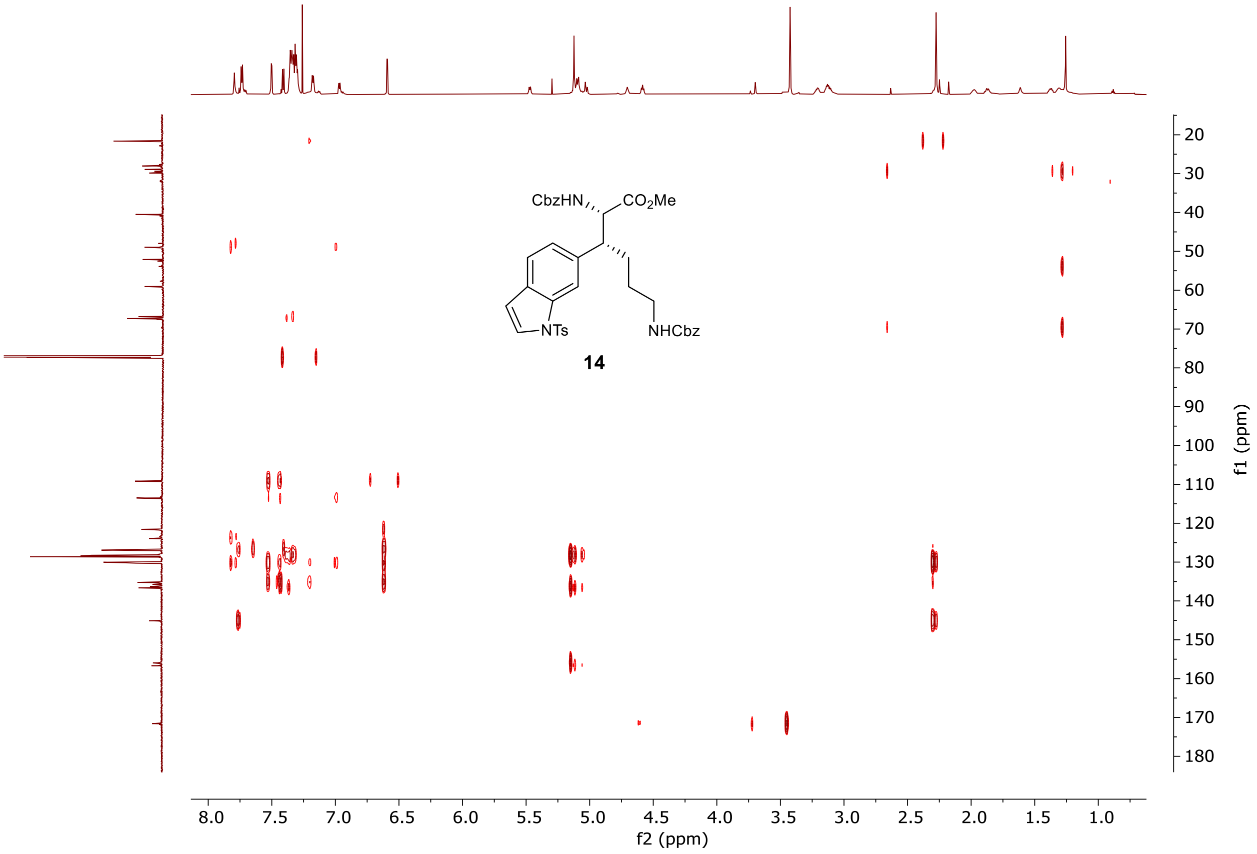

^{1}H NMR (400 MHz, $CDCl_3$, 25 °C) of compound **16** (1.4 isolated dr)

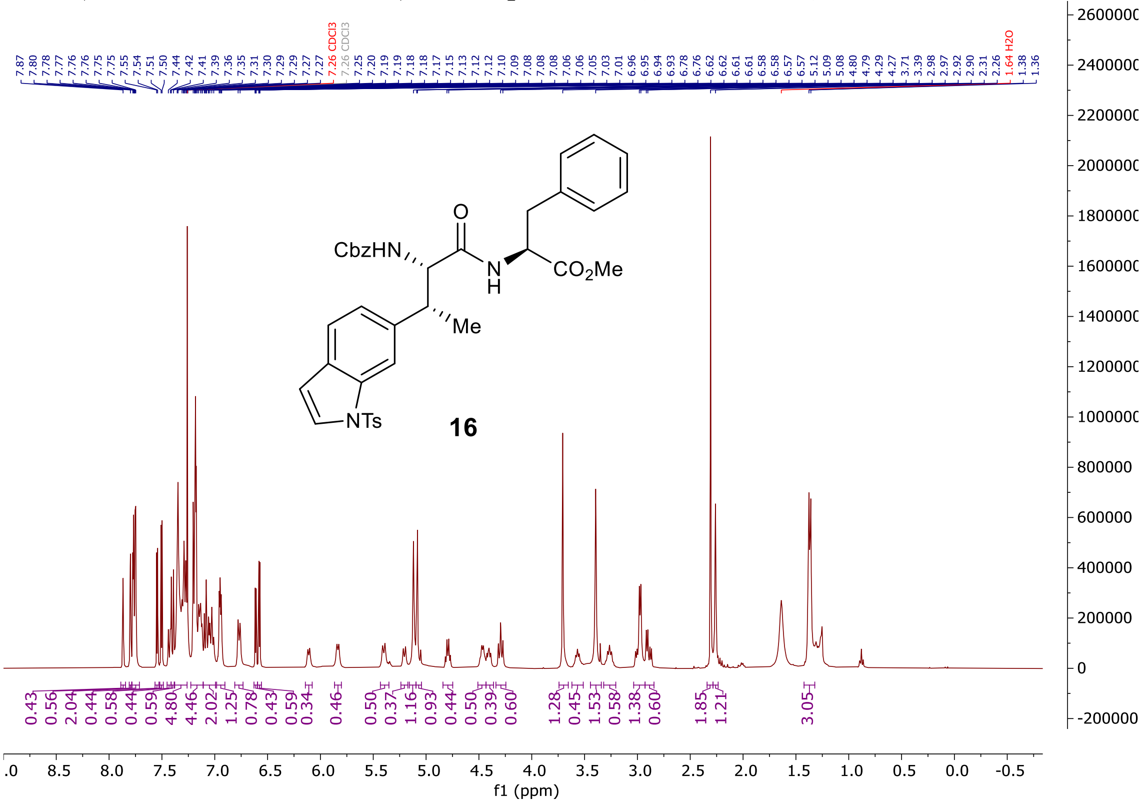

^{13}C NMR (201 MHz, $CDCl_3$) of compound **16**

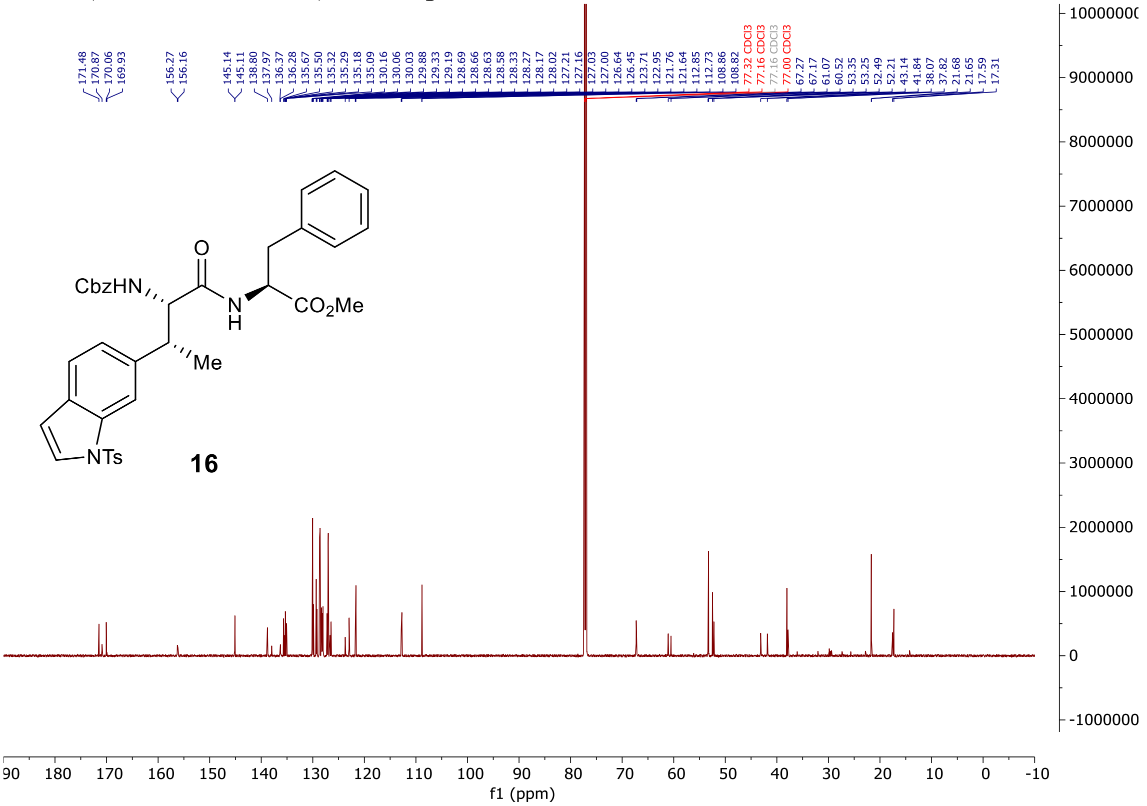

COSY (800 MHz, $CDCl_3$) of compound **16**

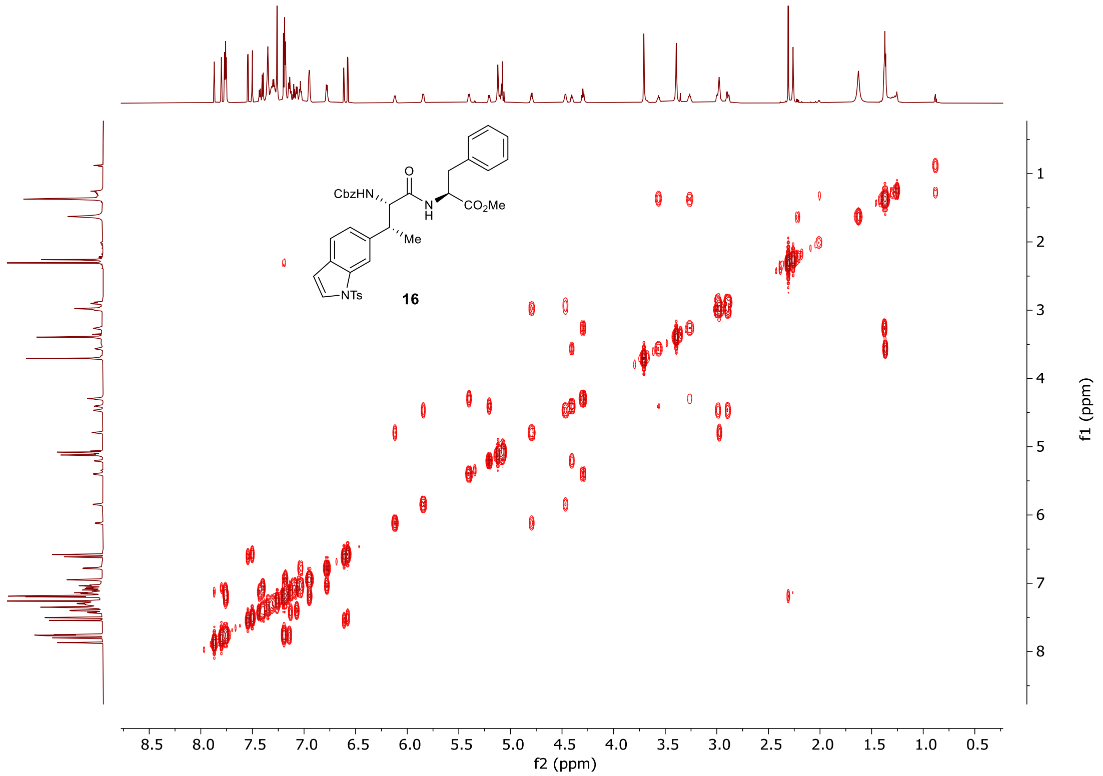

NOESY (800 MHz, $CDCl_3$) of compound **16**

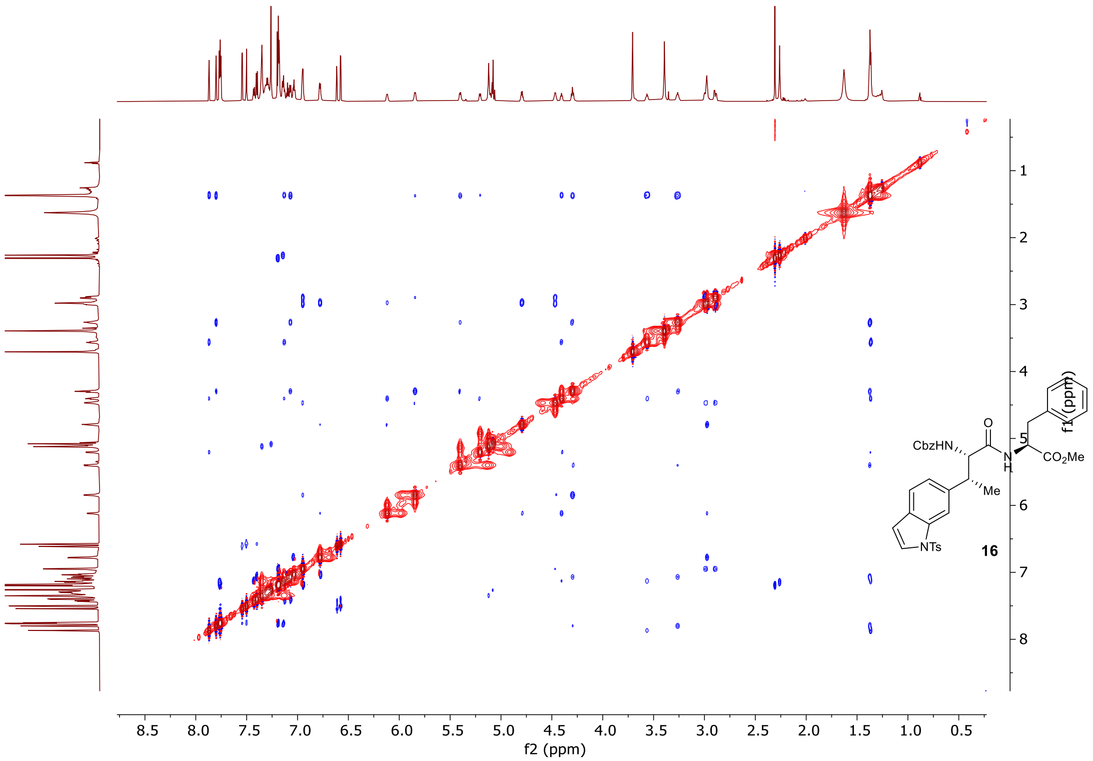

HMBC (800 MHz, $CDCl_3$) of compound **16**

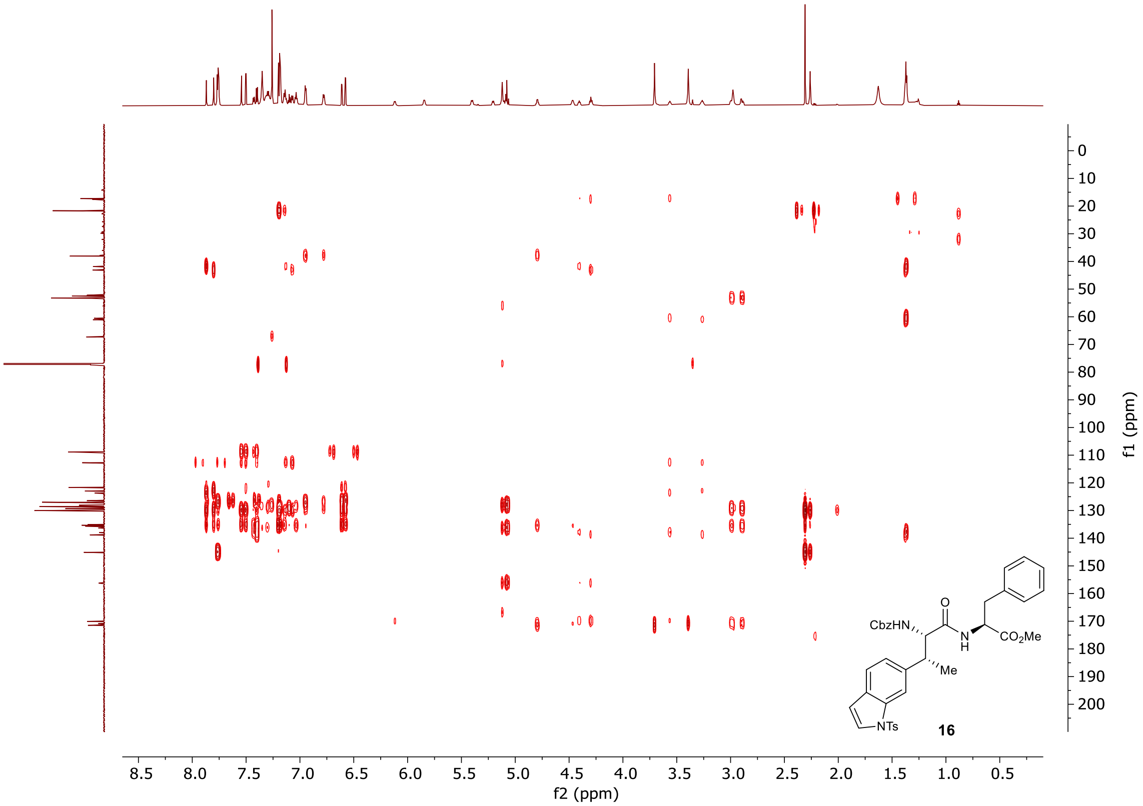

^{1}H NMR (800 MHz, $CDCl_3$, 25 °C) of compound **17** (6:1 isolated dr)

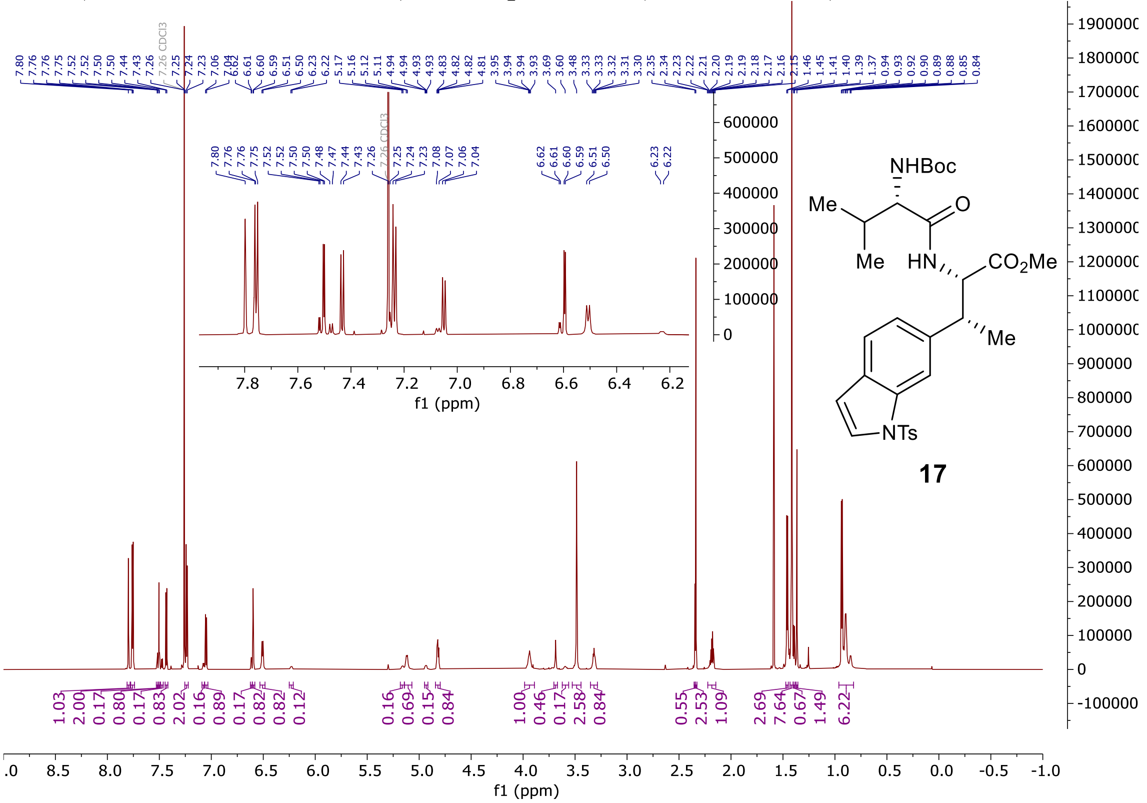

^{13}C NMR (201 MHz, $CDCl_3$) of compound **17**

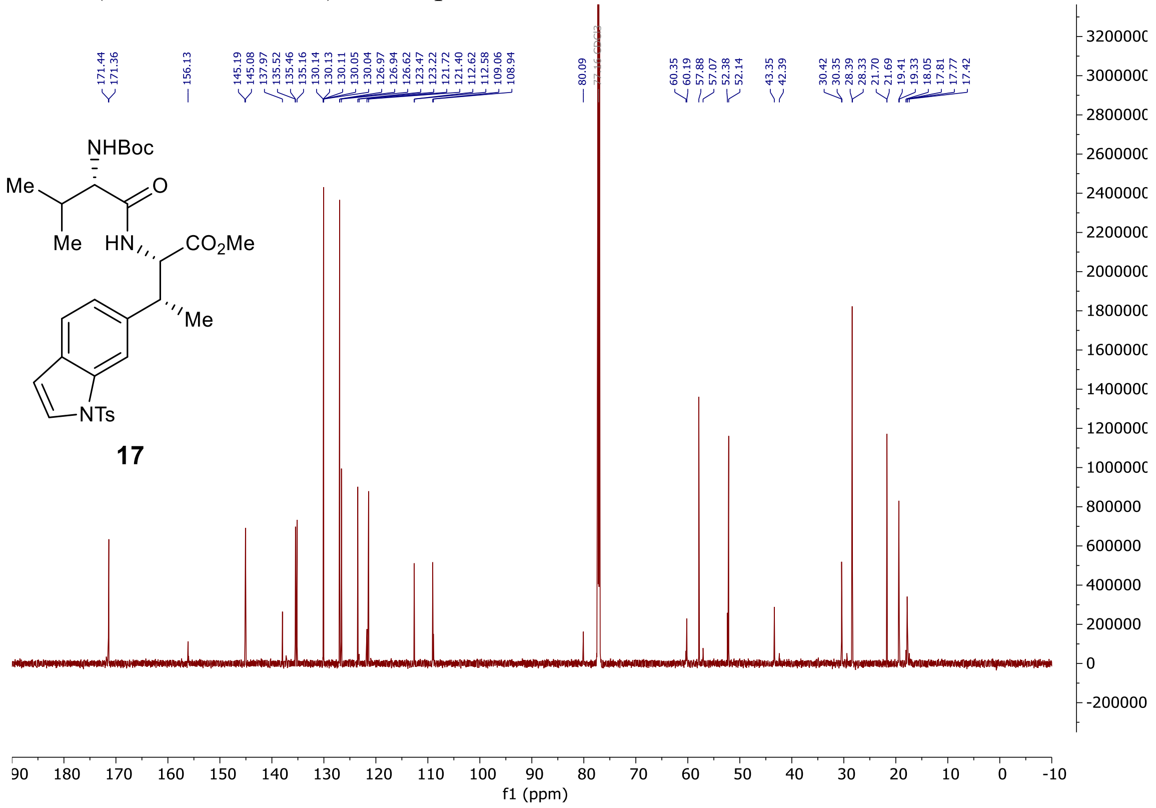

COSY (800 MHz, $CDCl_3$) of compound **17**

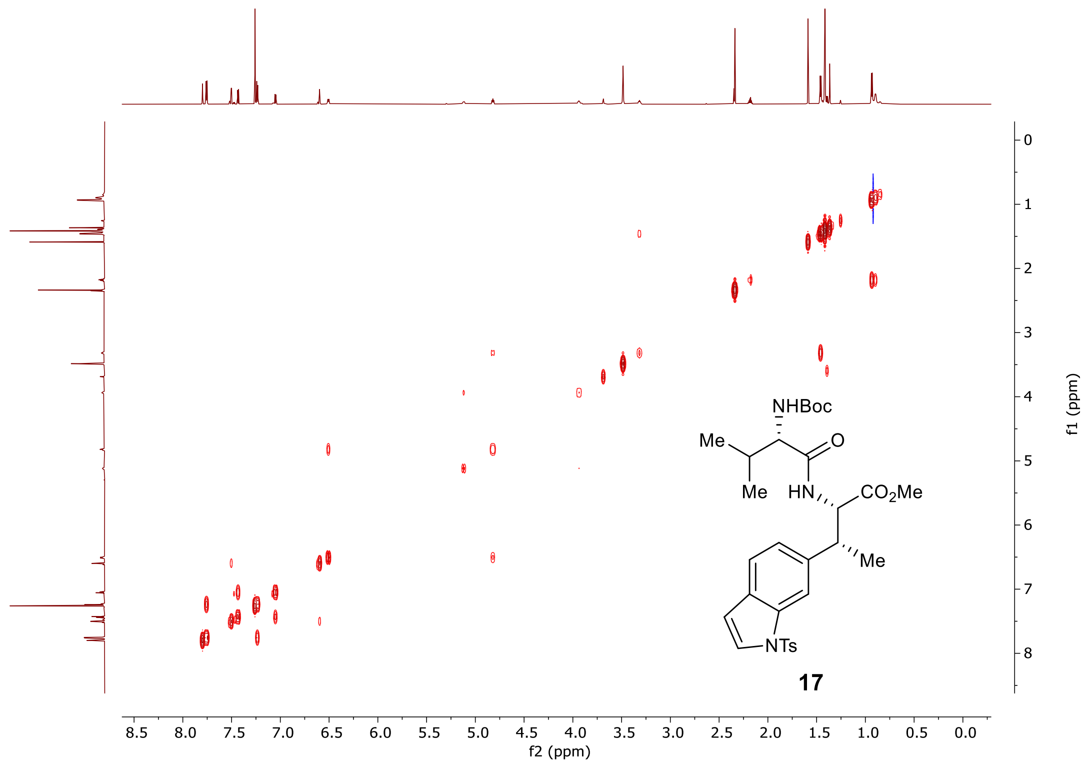

NOESY (800 MHz, $CDCl_3$) of compound **17**

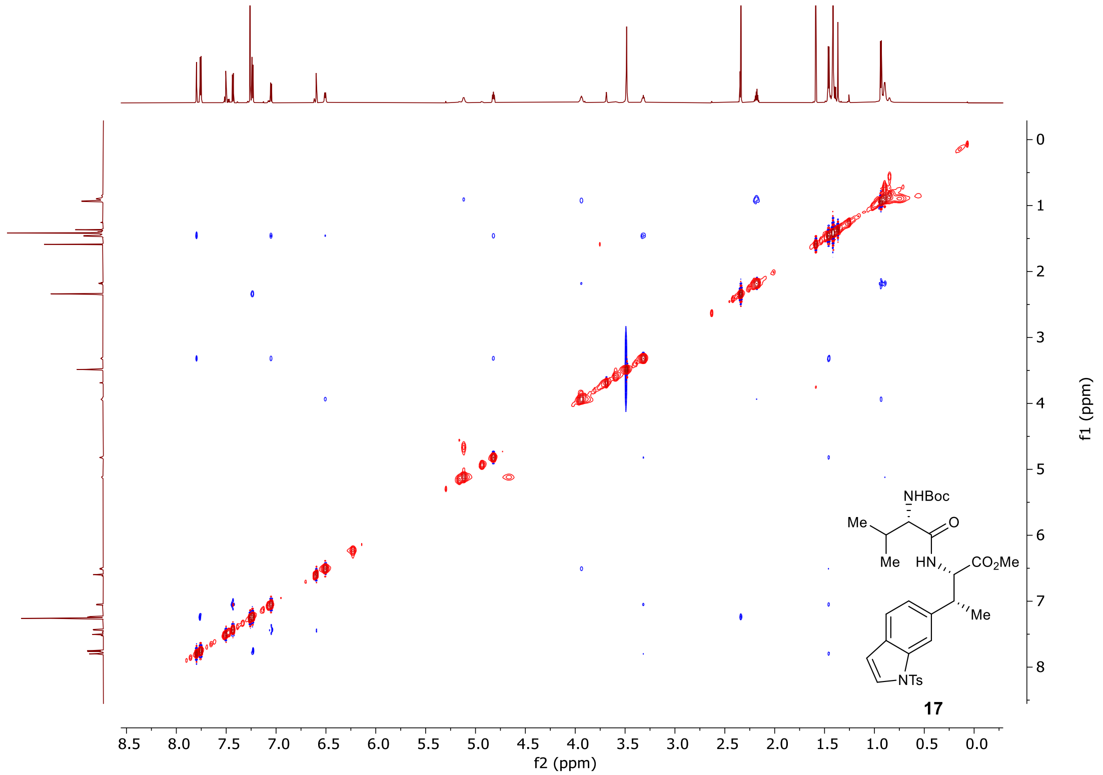

HMBC (800 MHz, $CDCl_3$) of compound **17**

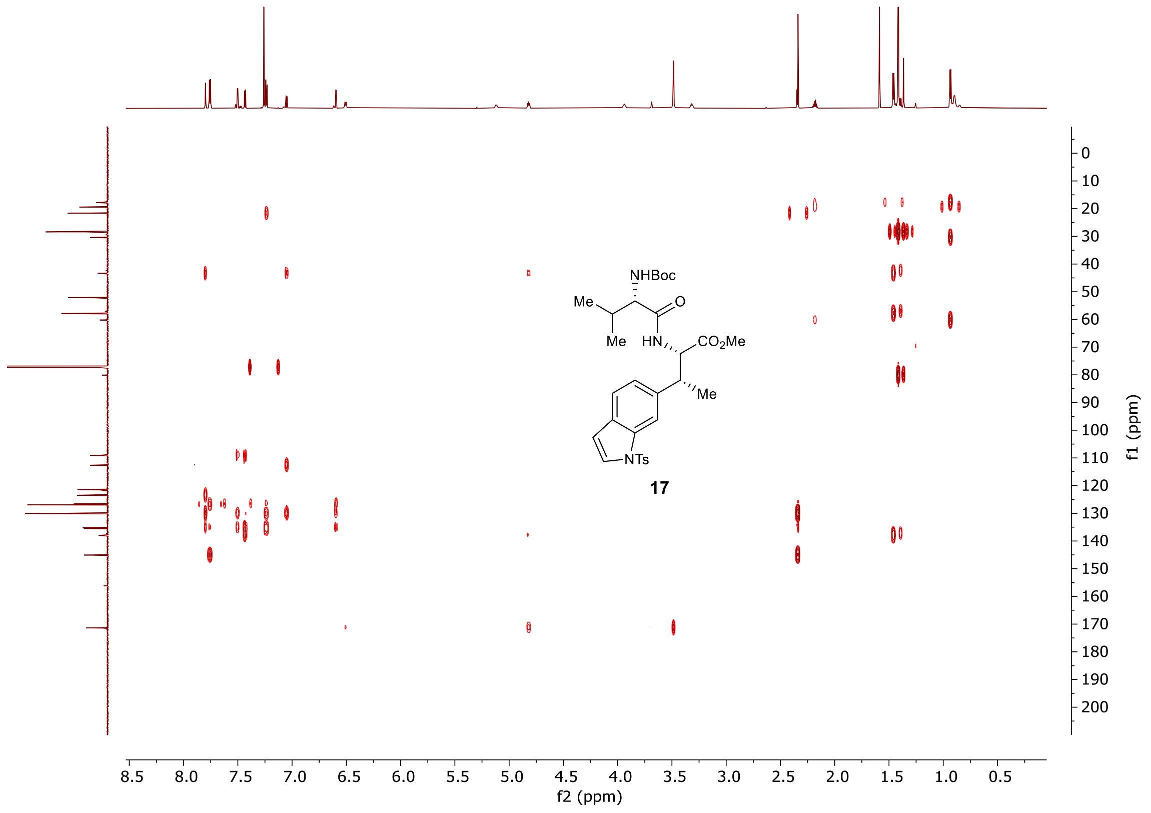

^{1}H NMR (400 MHz, $CDCl_3$) of compound **18** (9:1 isolated dr)

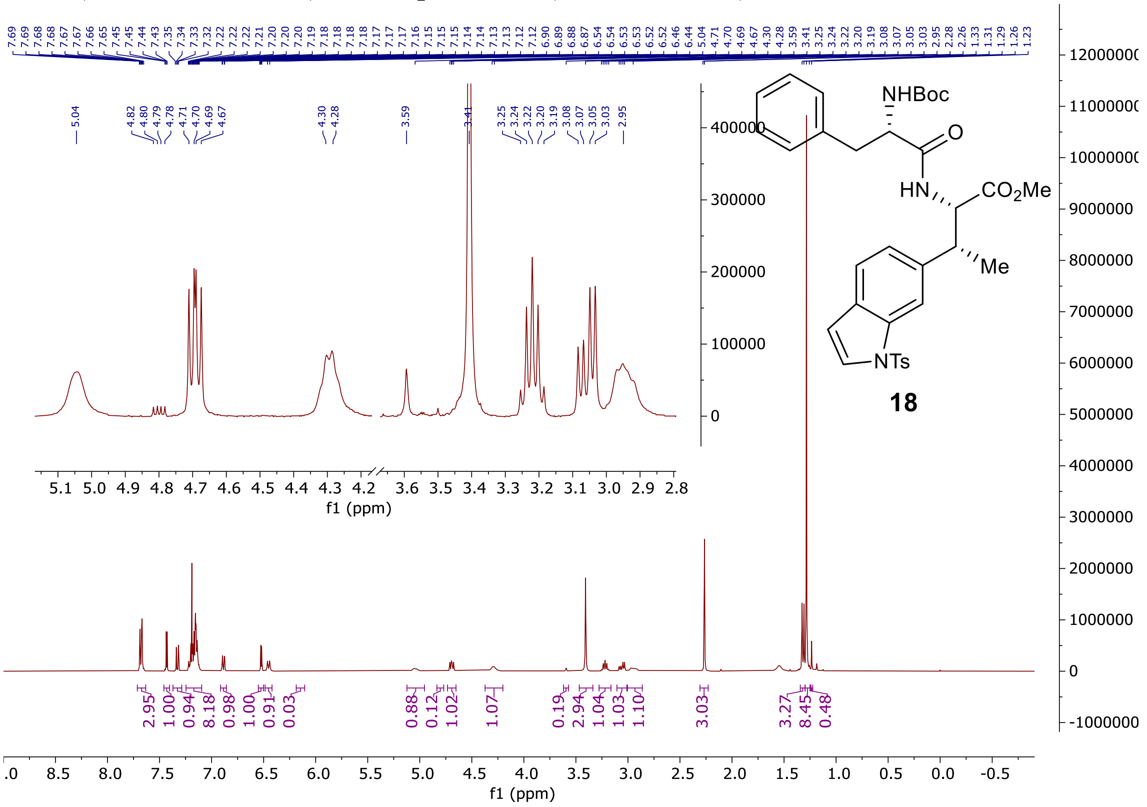

^{13}C NMR (201 MHz, $CDCl_3$) of compound **18**

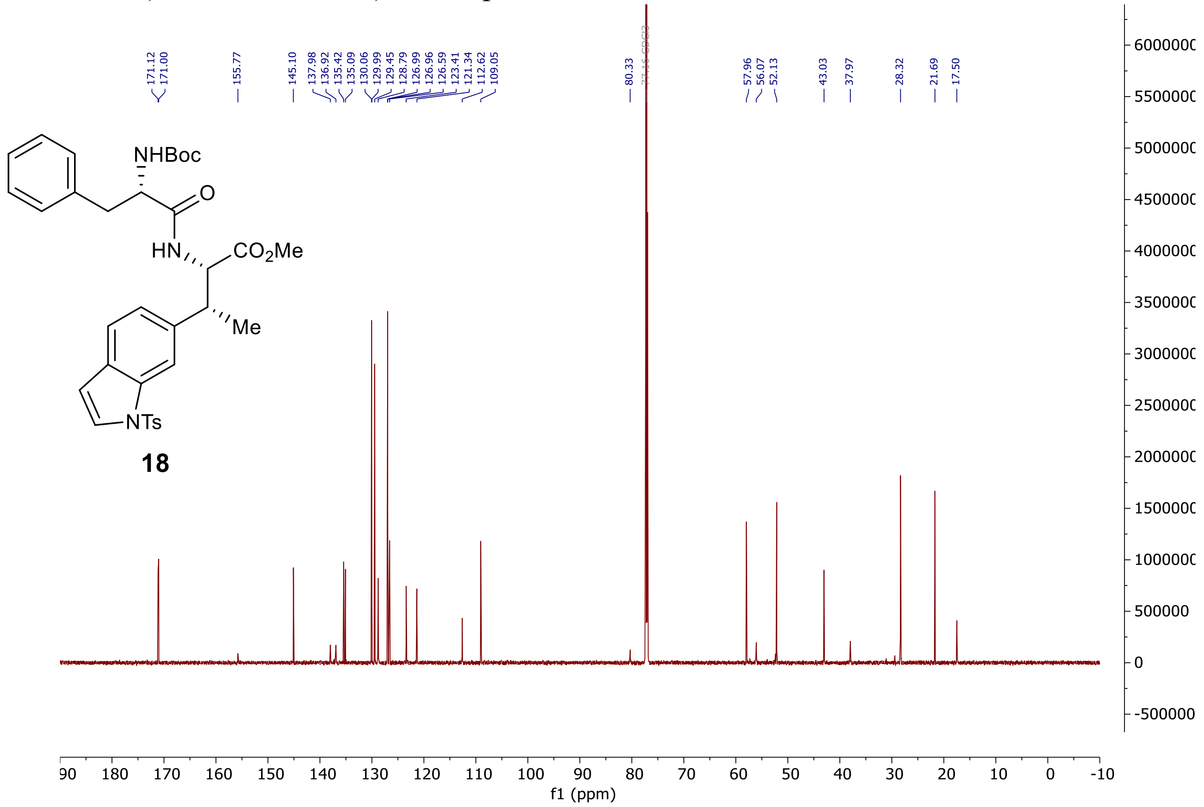

COSY (800 MHz, $CDCl_3$) of compound **18**

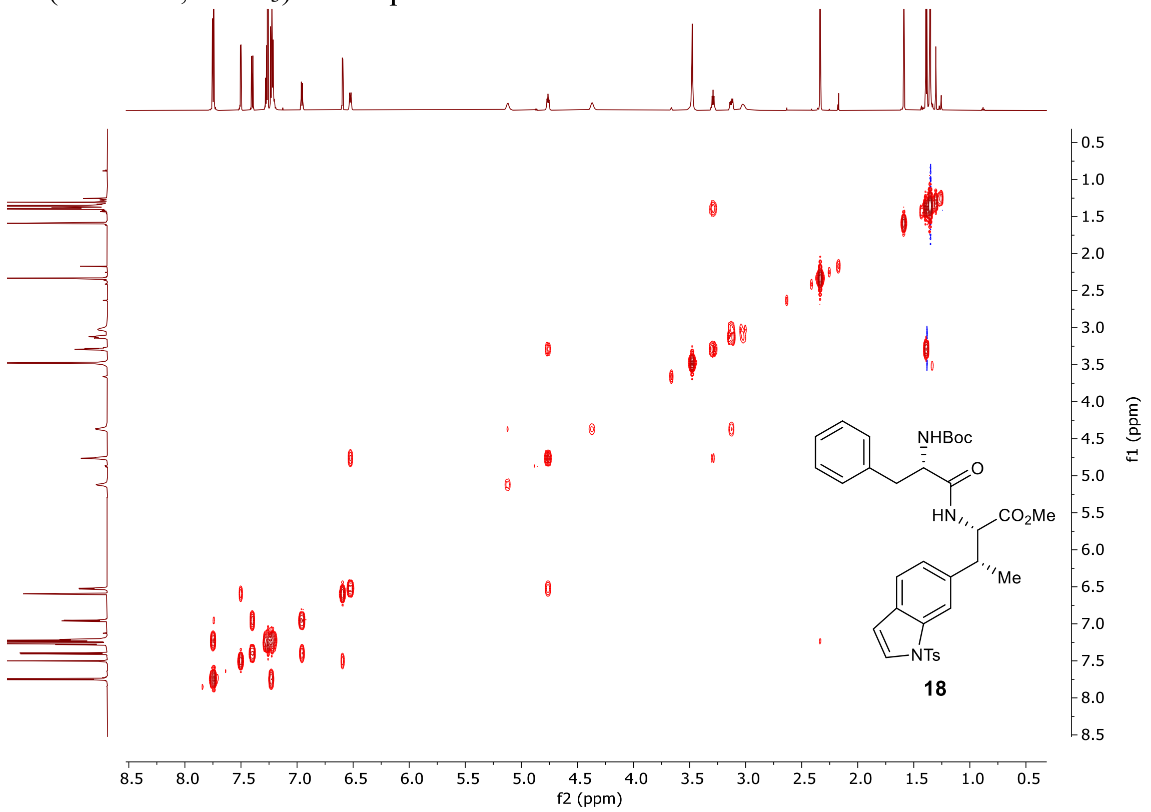

HMBC (800 MHz, $CDCl_3$) of compound **18**

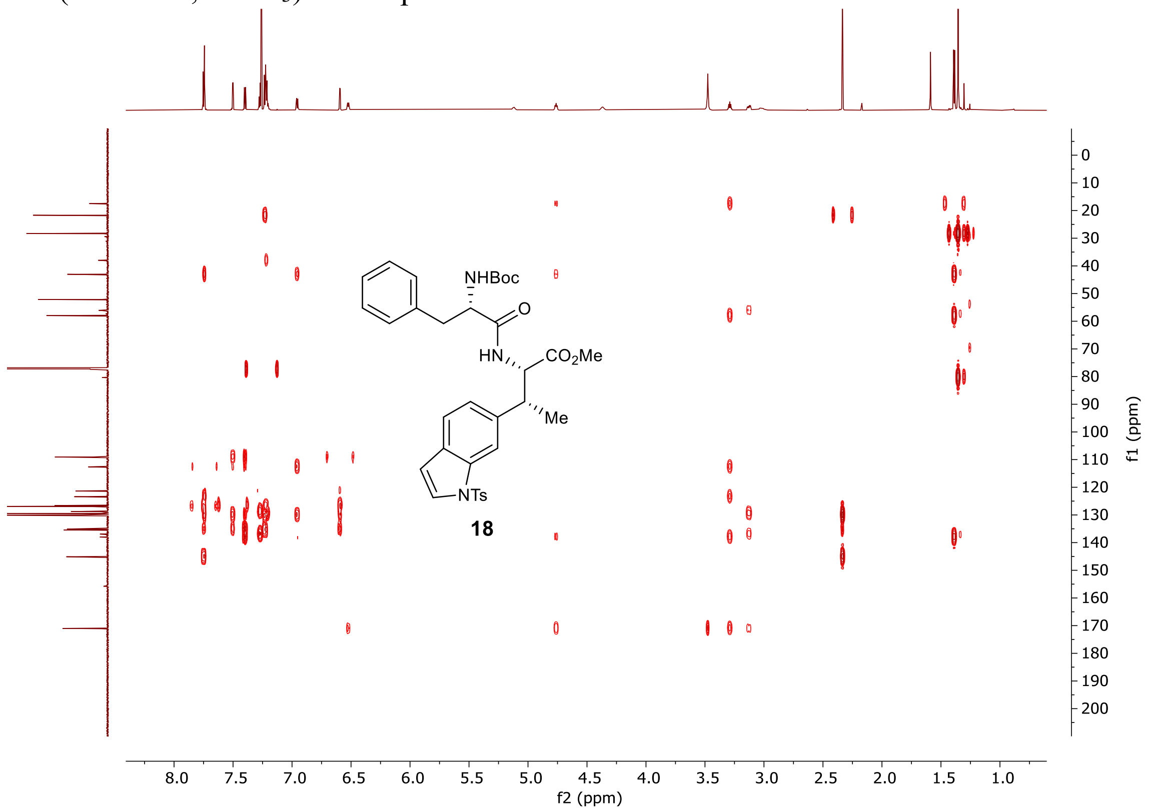

NOESY (800 MHz, $CDCl_3$) of compound **18**

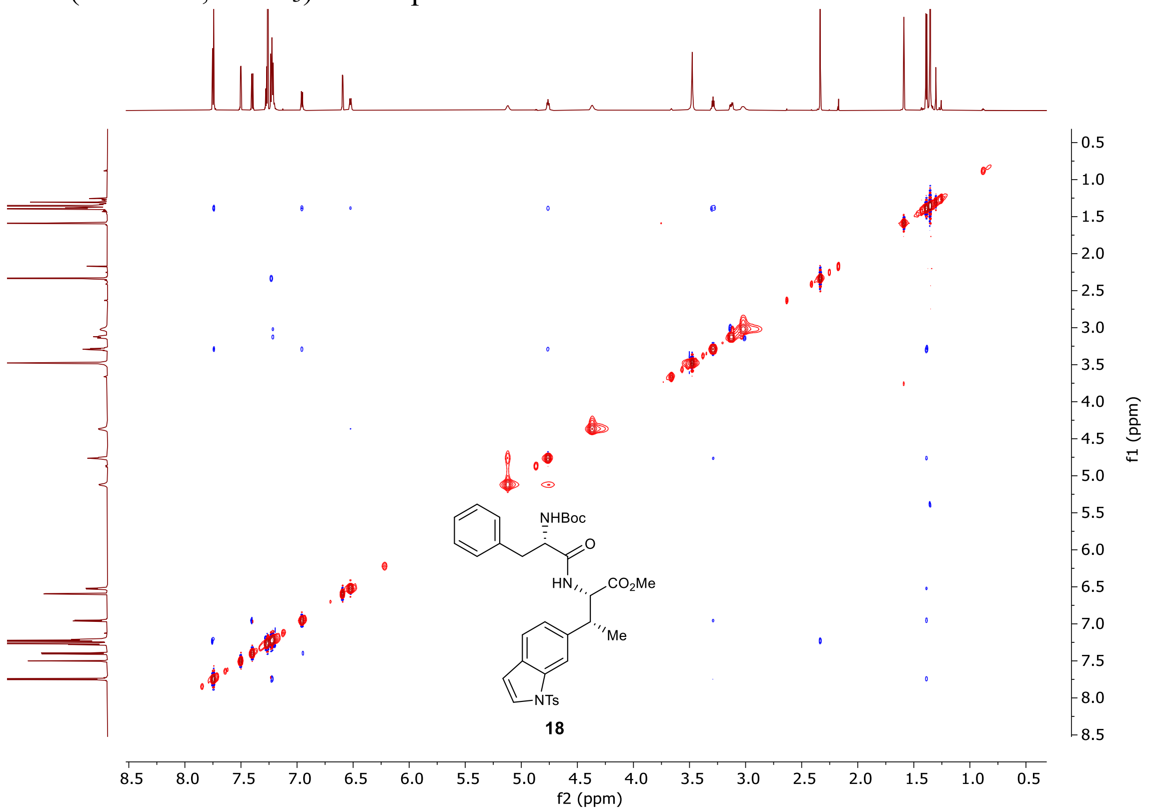

^{1}H NMR (600 MHz, $CDCl_3$) of compound **19** (>20:1 isolated dr)

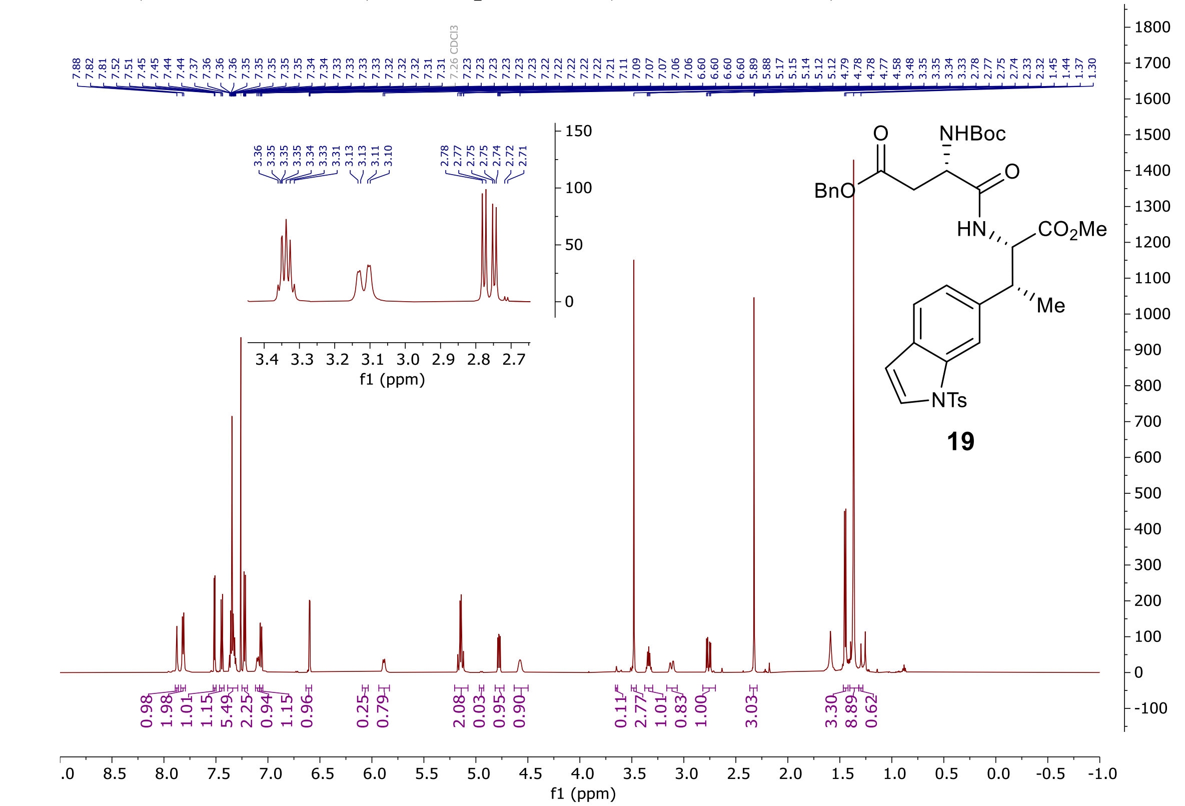

^{13}C NMR (151 MHz, $CDCl_3$) of compound **19**

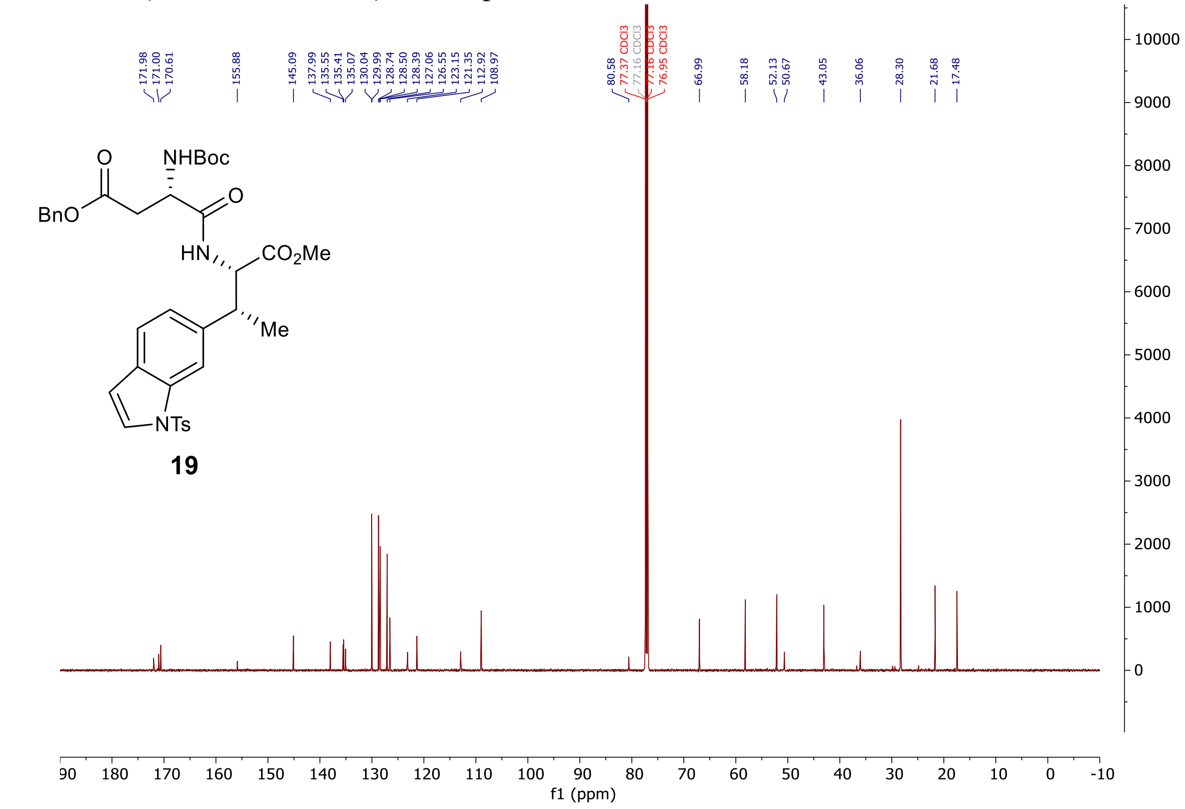

COSY (600 MHz, $CDCl_3$) of compound **19**

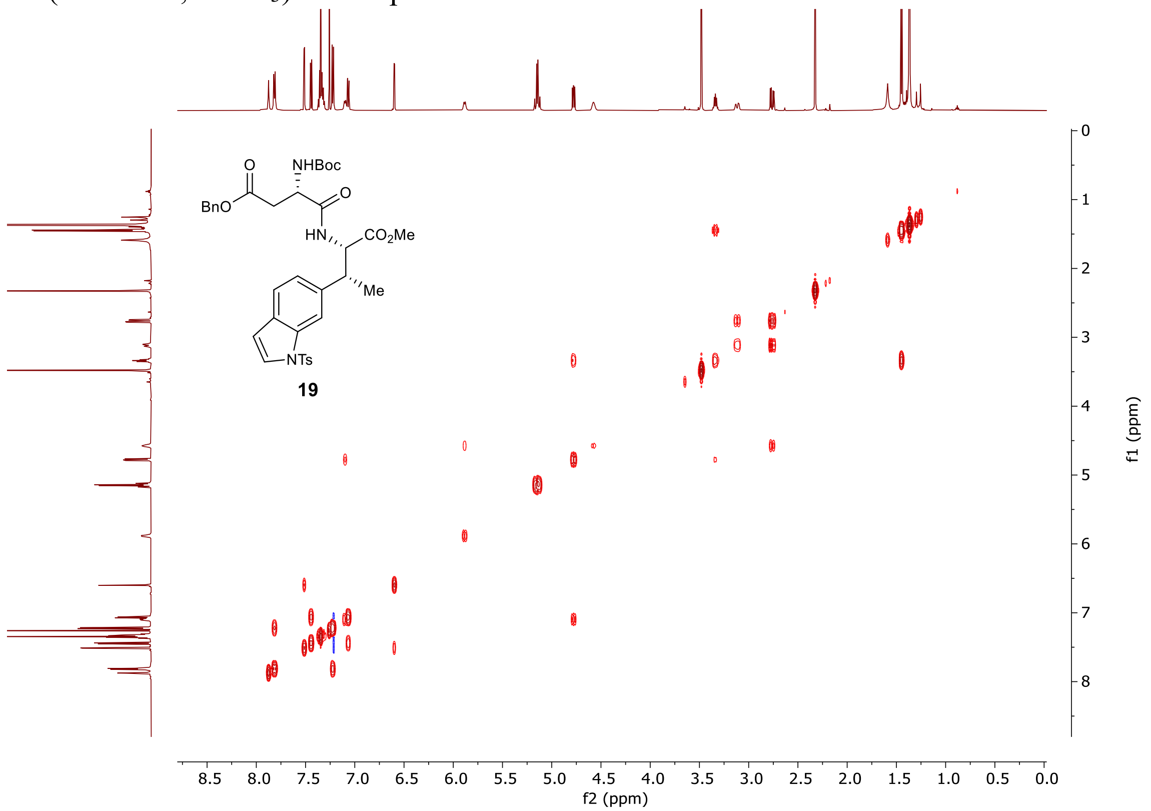

NOESY (600 MHz, $CDCl_3$) of compound **19**

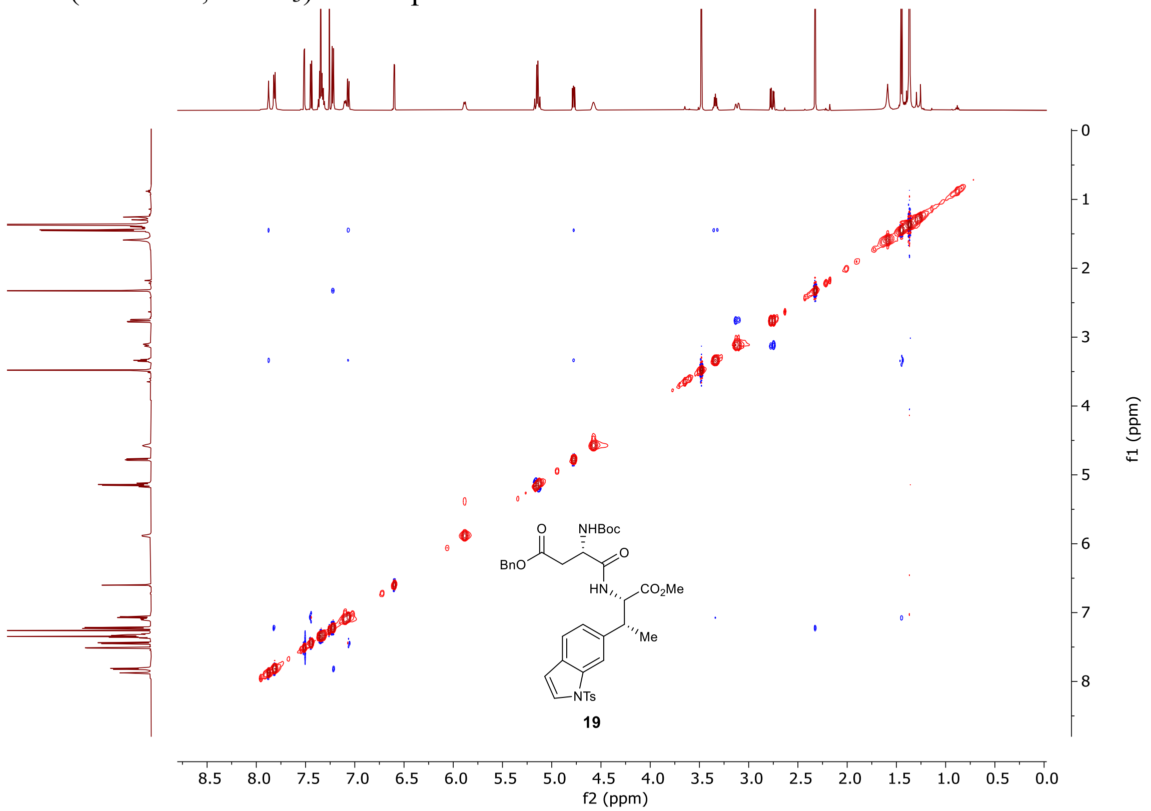

HMBC (600 MHz, $CDCl_3$) of compound **19**

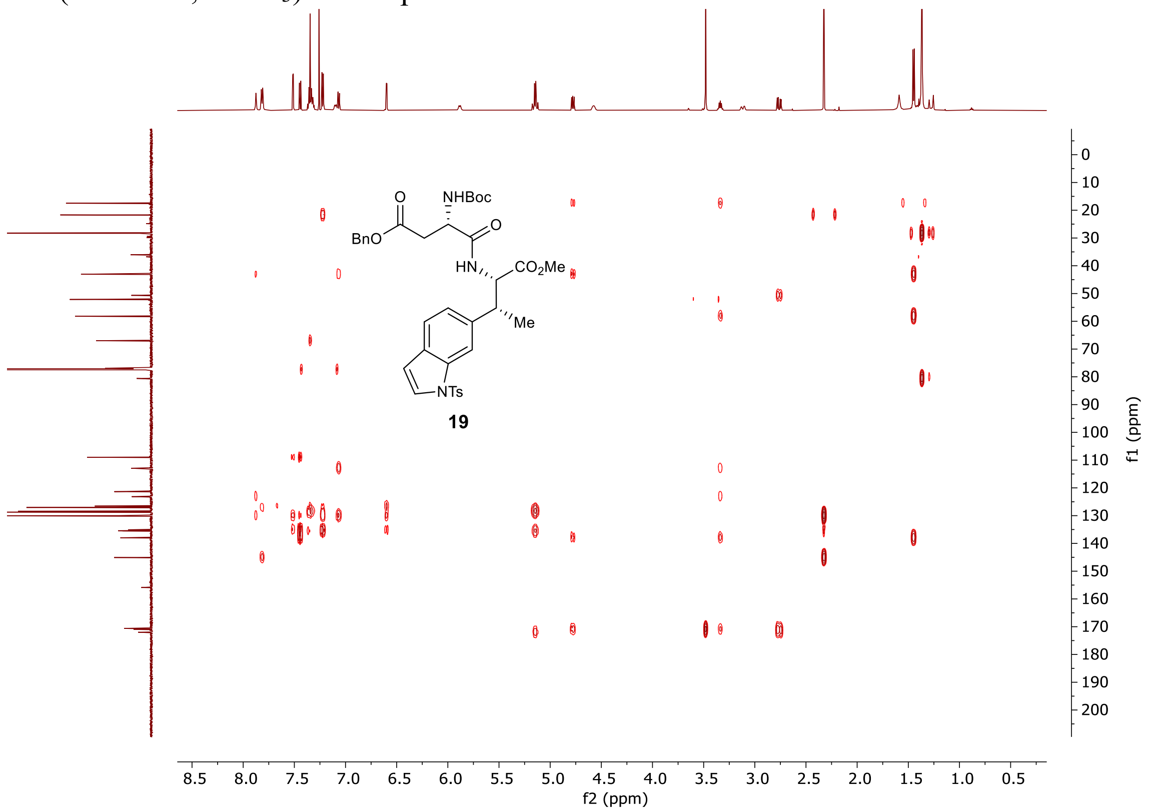

^{1}H NMR (600 MHz, $CDCl_3$) of compound **20** (12:1 isolated dr)

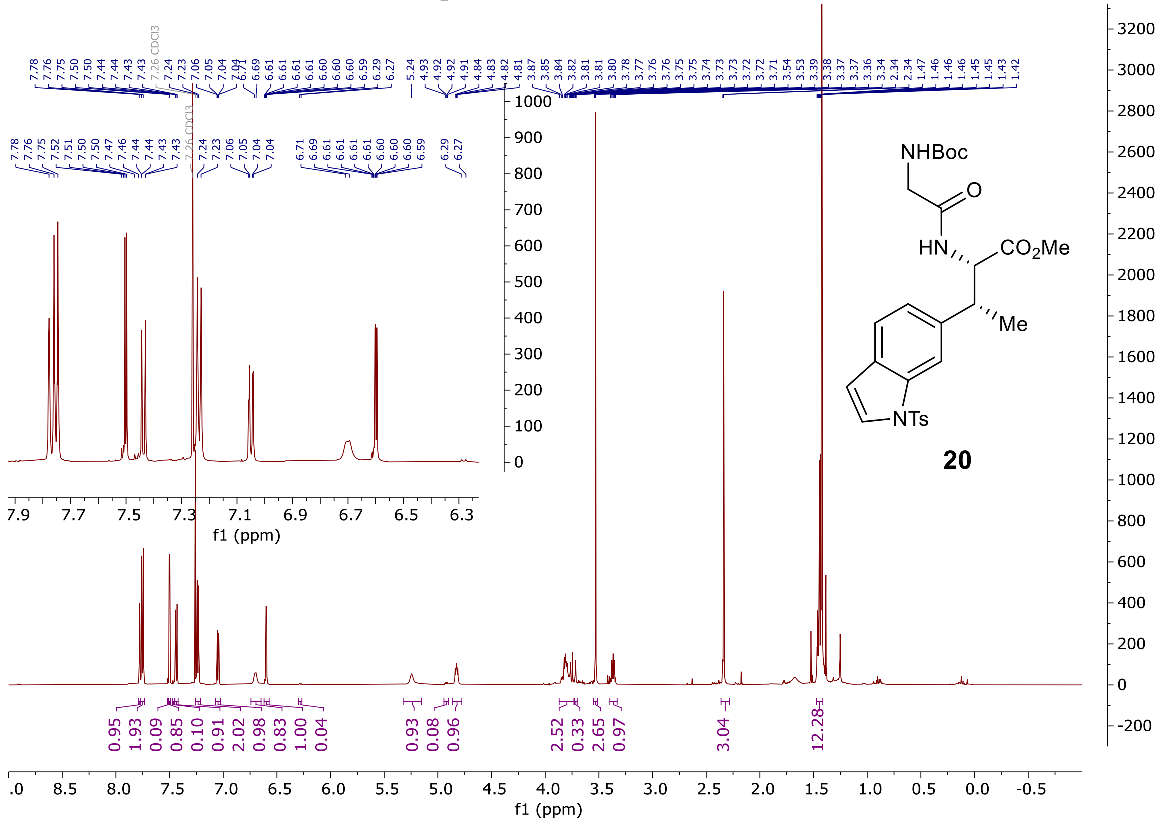

^{13}C NMR (151 MHz, $CDCl_3$) of compound **20**

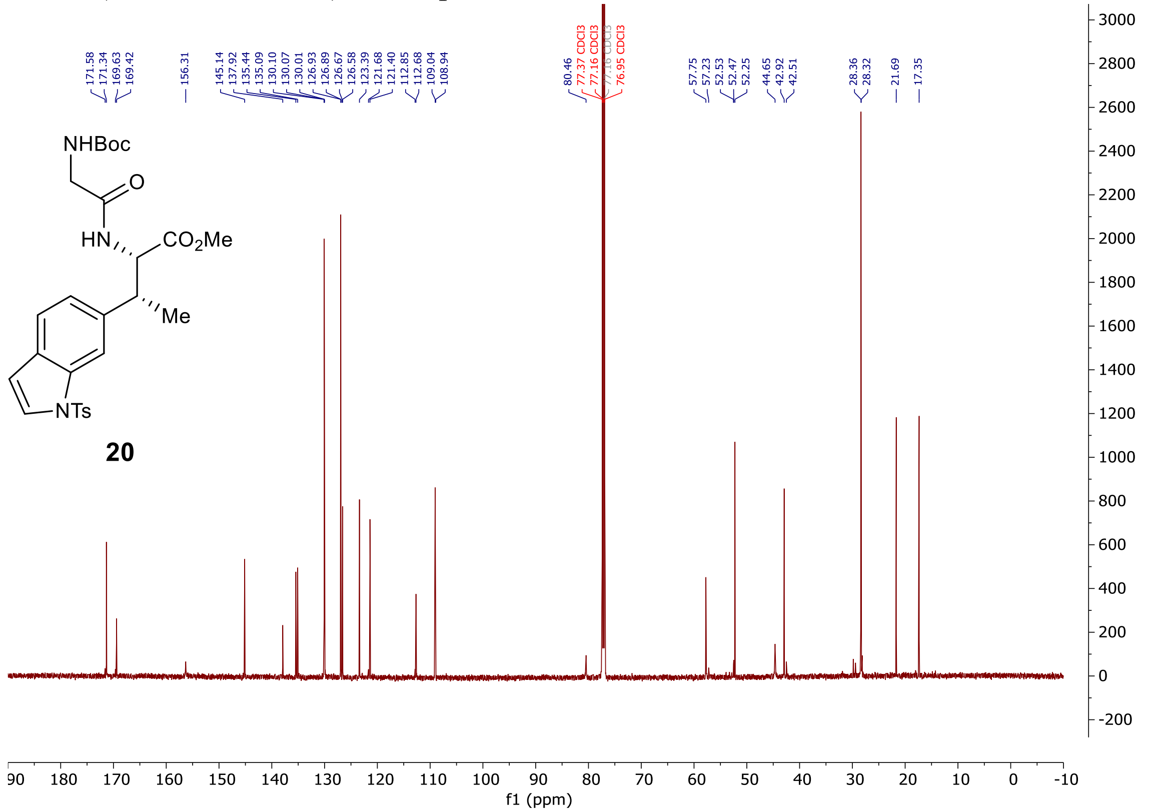

COSY (600 MHz, $CDCl_3$) of compound **20**

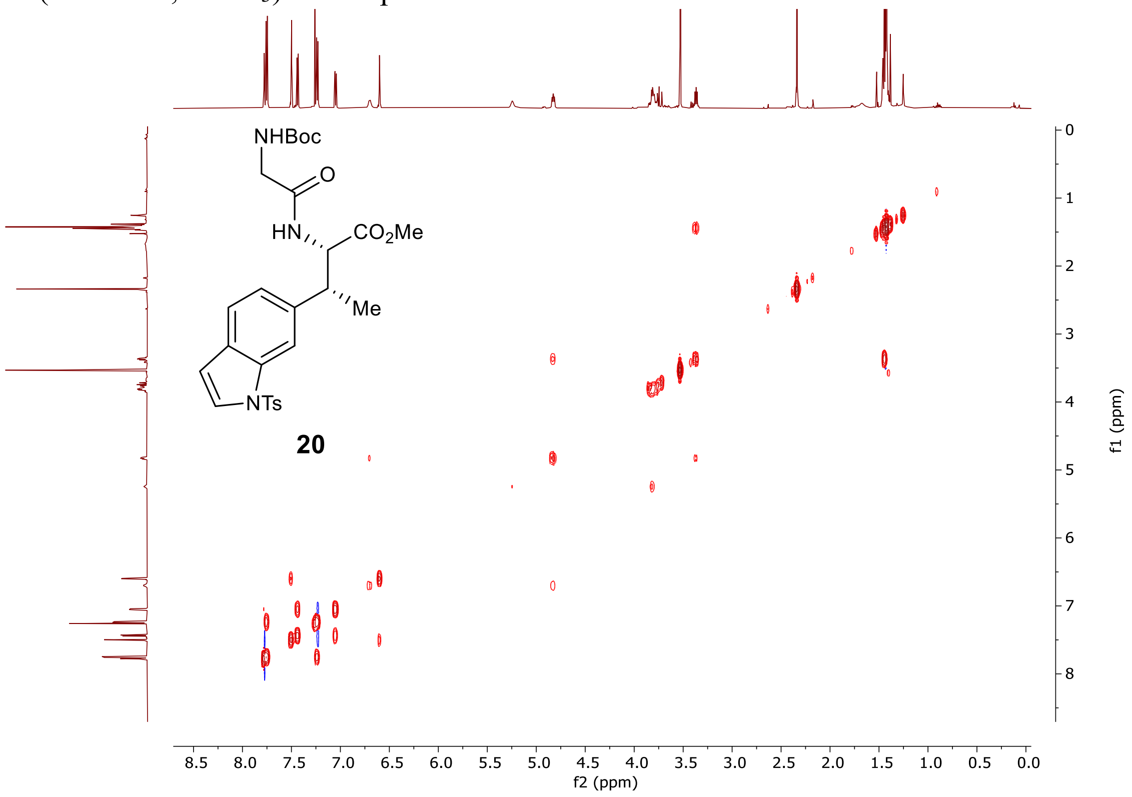

NOESY (600 MHz, $CDCl_3$) of compound **20**

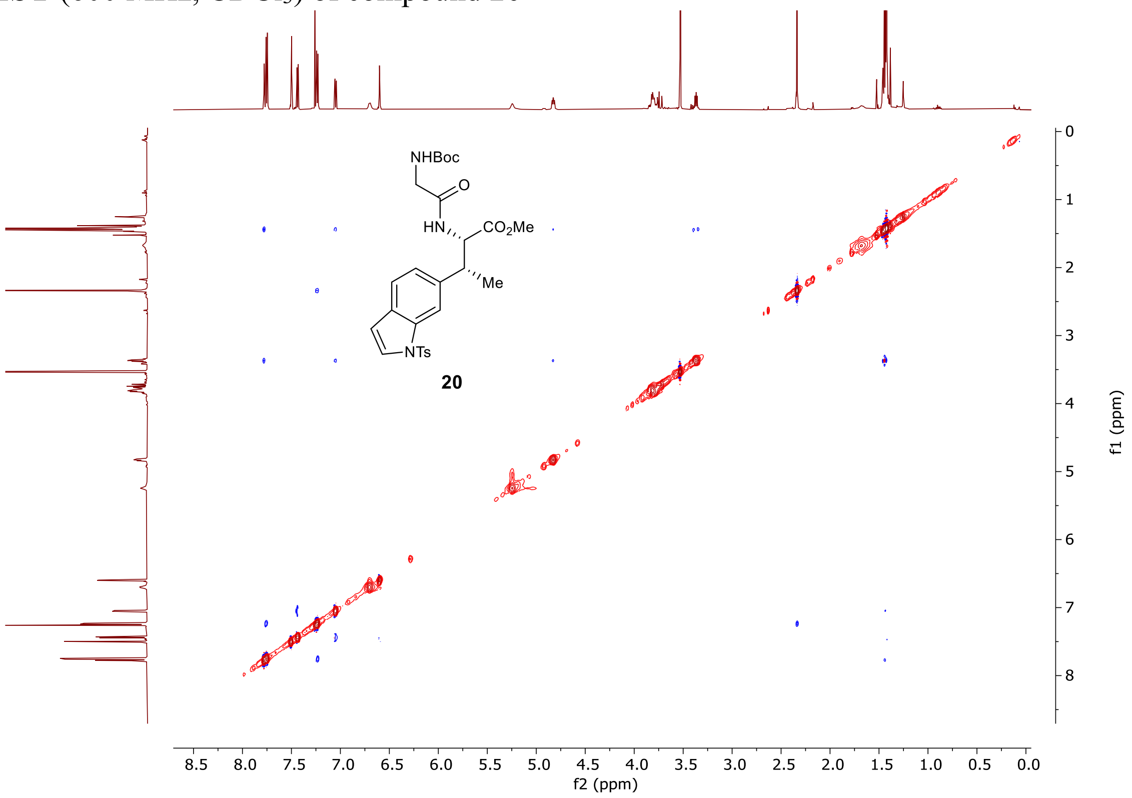

HMBC (600 MHz, $CDCl_3$) of compound **20**

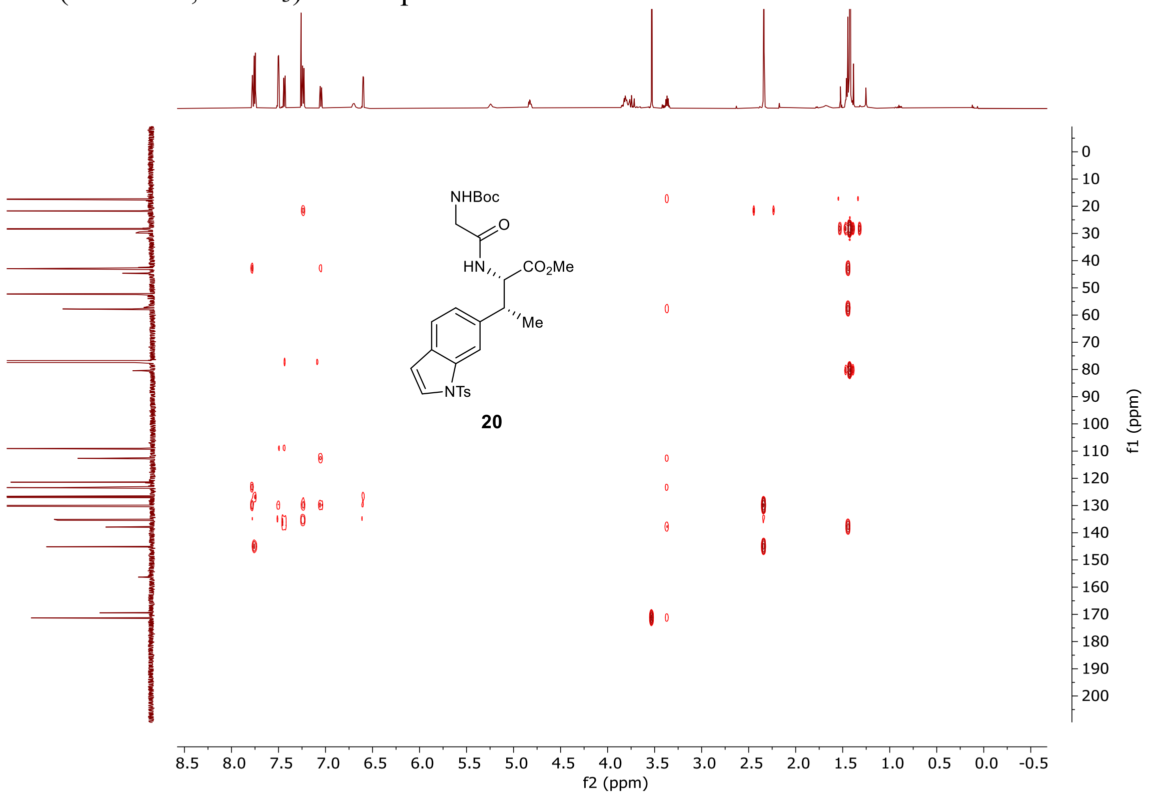

^{1}H NMR (400 MHz, $CDCl_3$) of compound **21** (13:1 isolated dr)

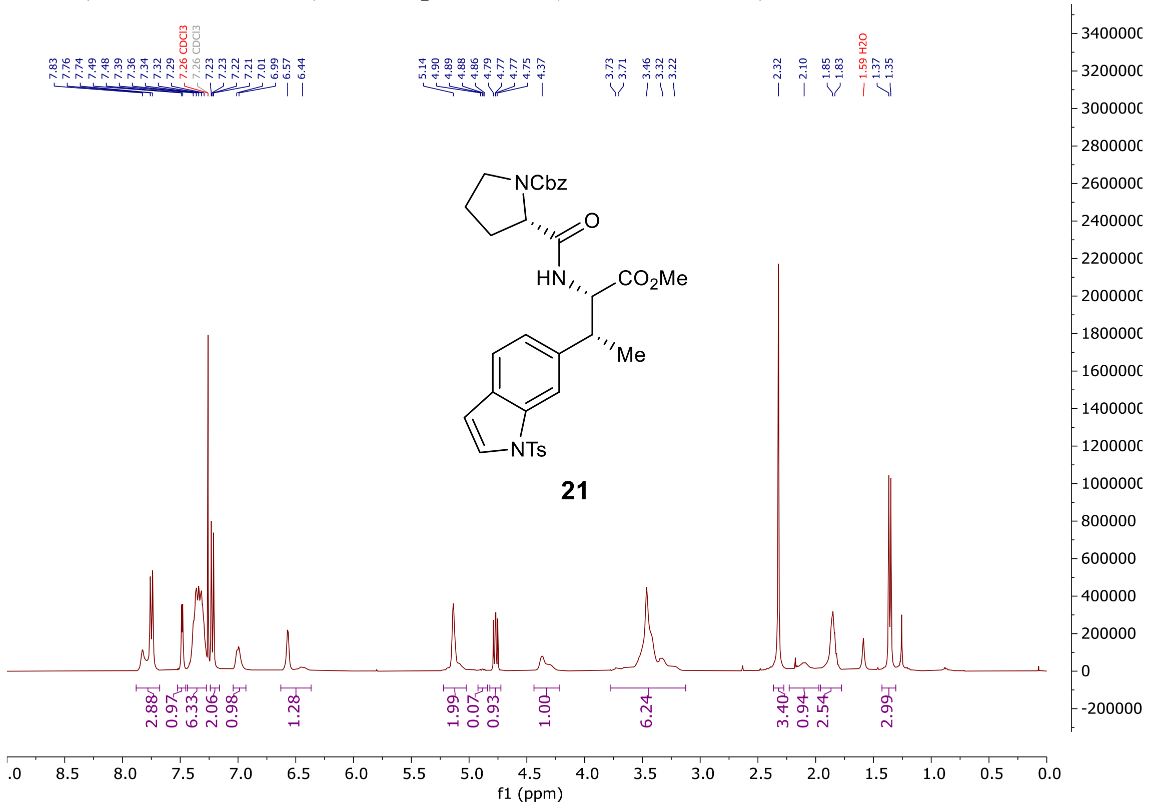

^{13}C NMR (151 MHz, $CDCl_3$) of compound **21**

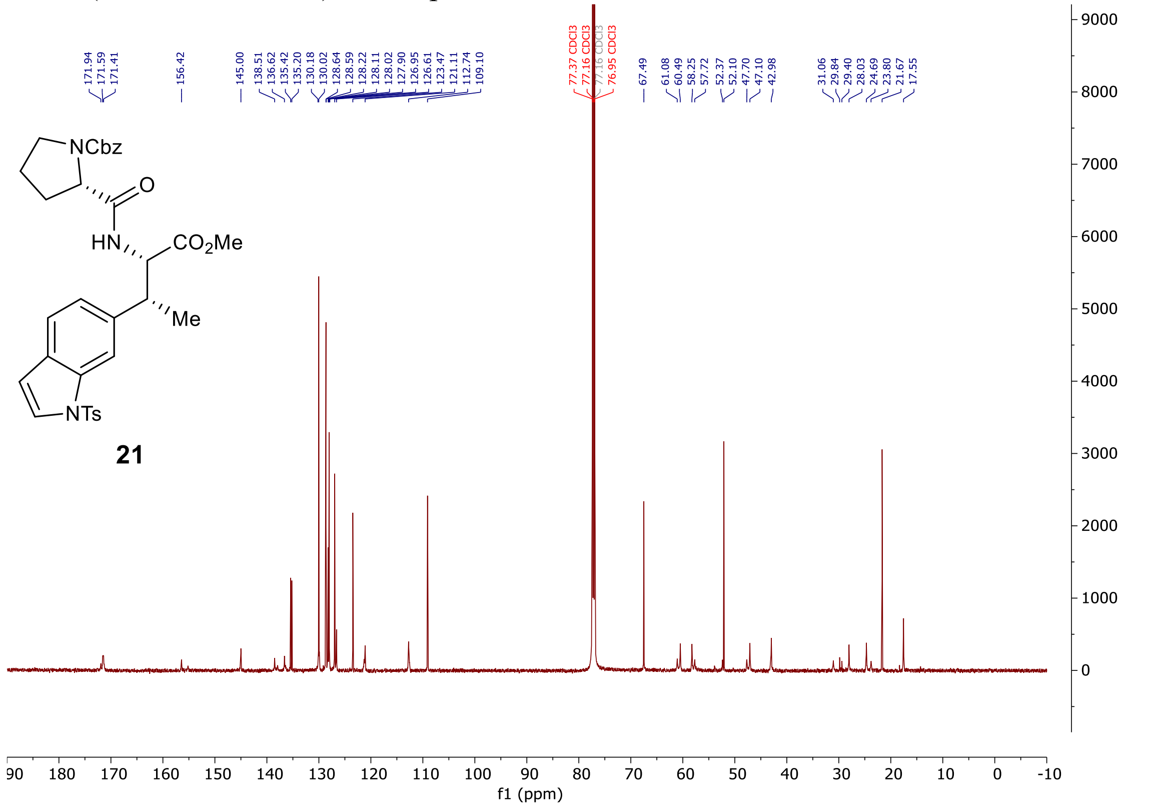

COSY (600 MHz, $CDCl_3$) of compound **21**

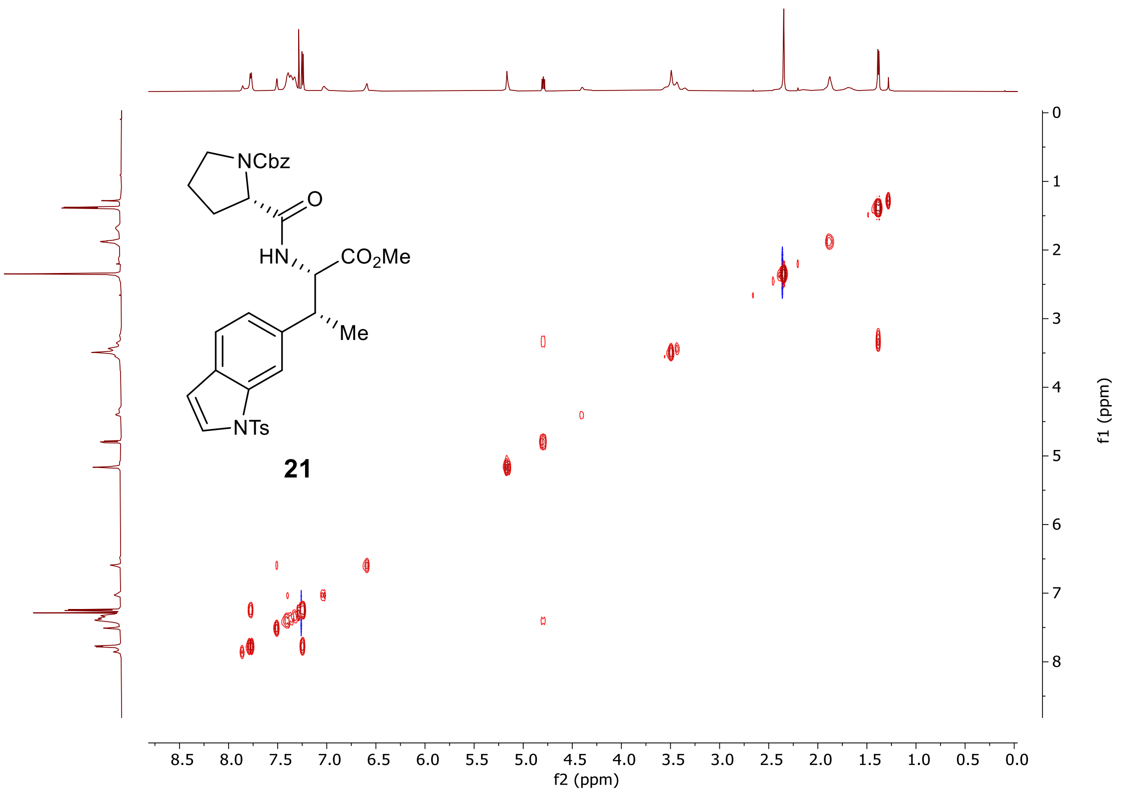

NOESY (600 MHz, $CDCl_3$) of compound **21**

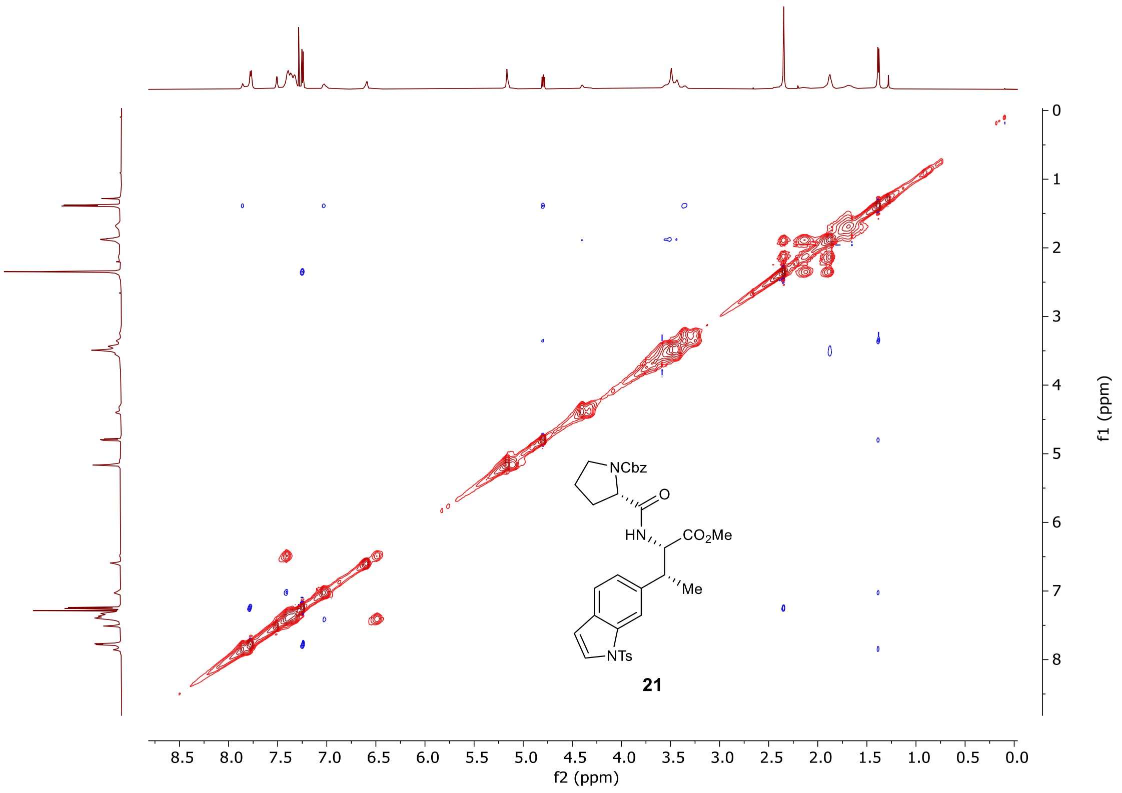

HMBC (600 MHz, $CDCl_3$) of compound **21**

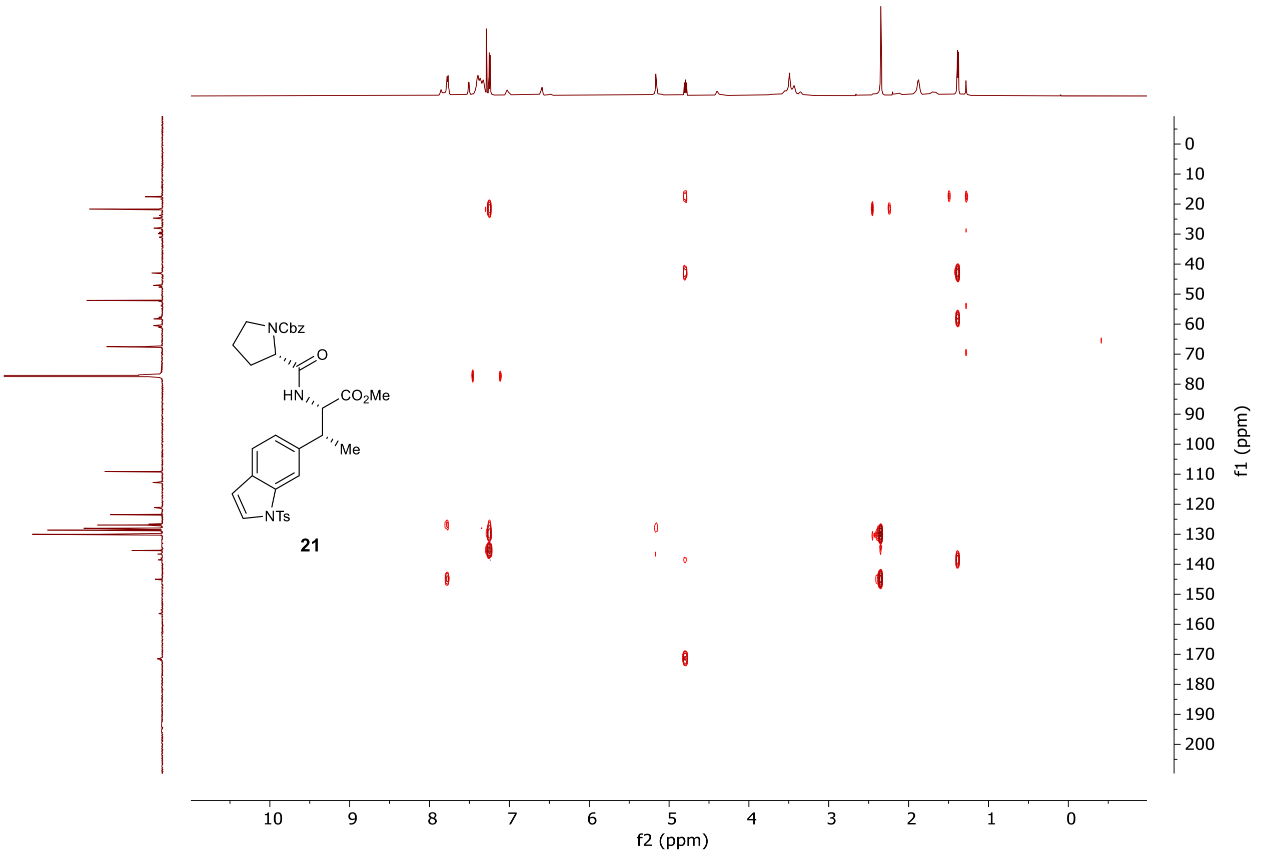

^{1}**H NMR** (800 MHz, $CDCl_3$, 25 °C) of compound **22** (7:1 isolated dr)

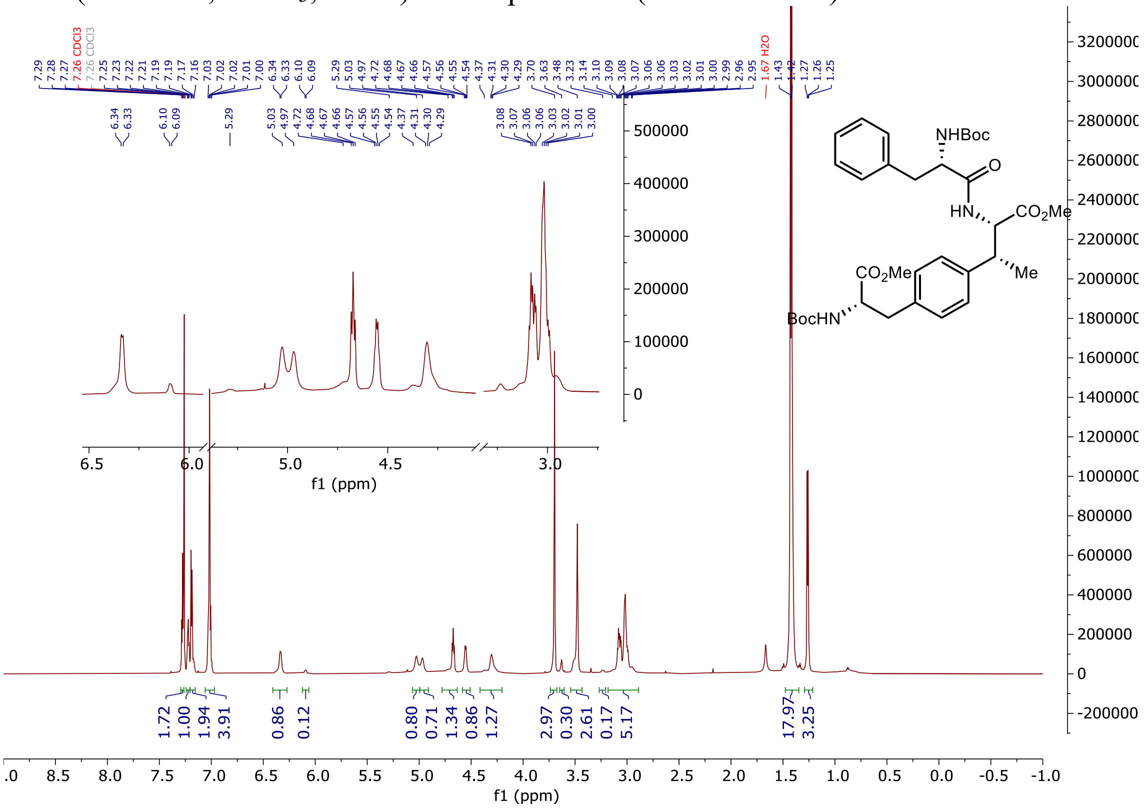

^{1}**H NMR** (600 MHz, $CDCl_3$, 50 °C) of compound **22**

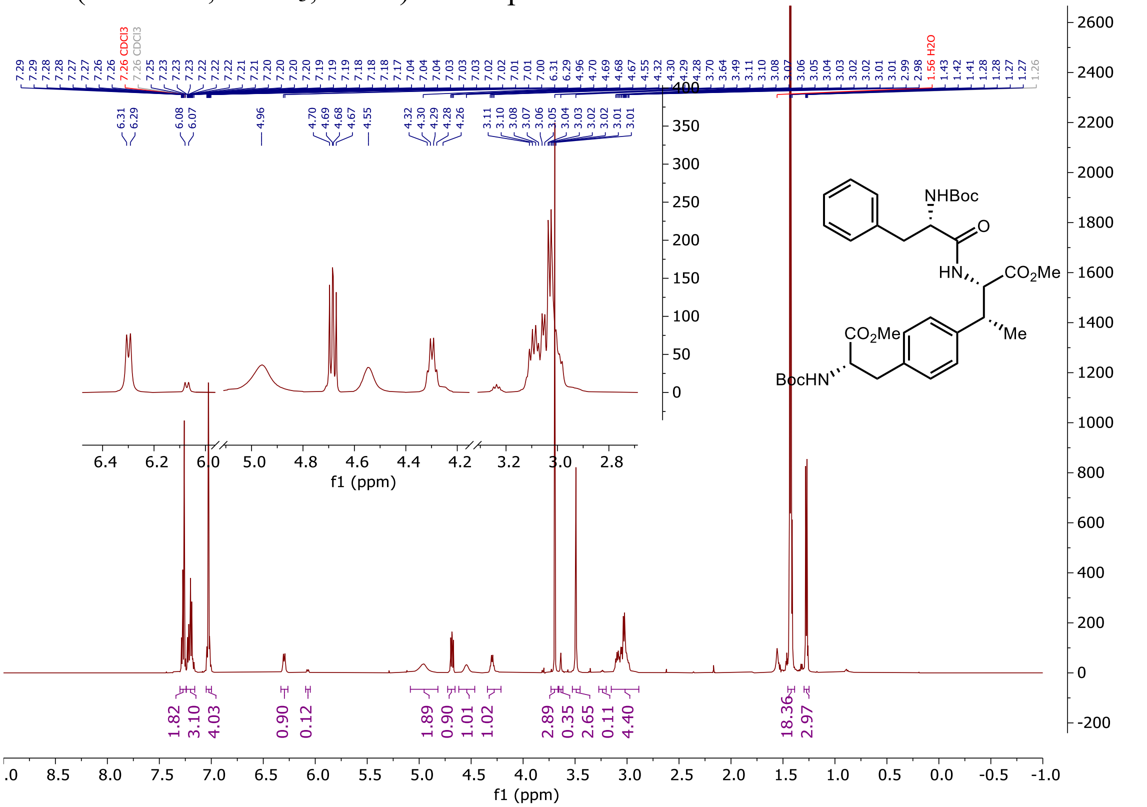

^{13}C NMR (201 MHz, $CDCl_3$) of compound **22**

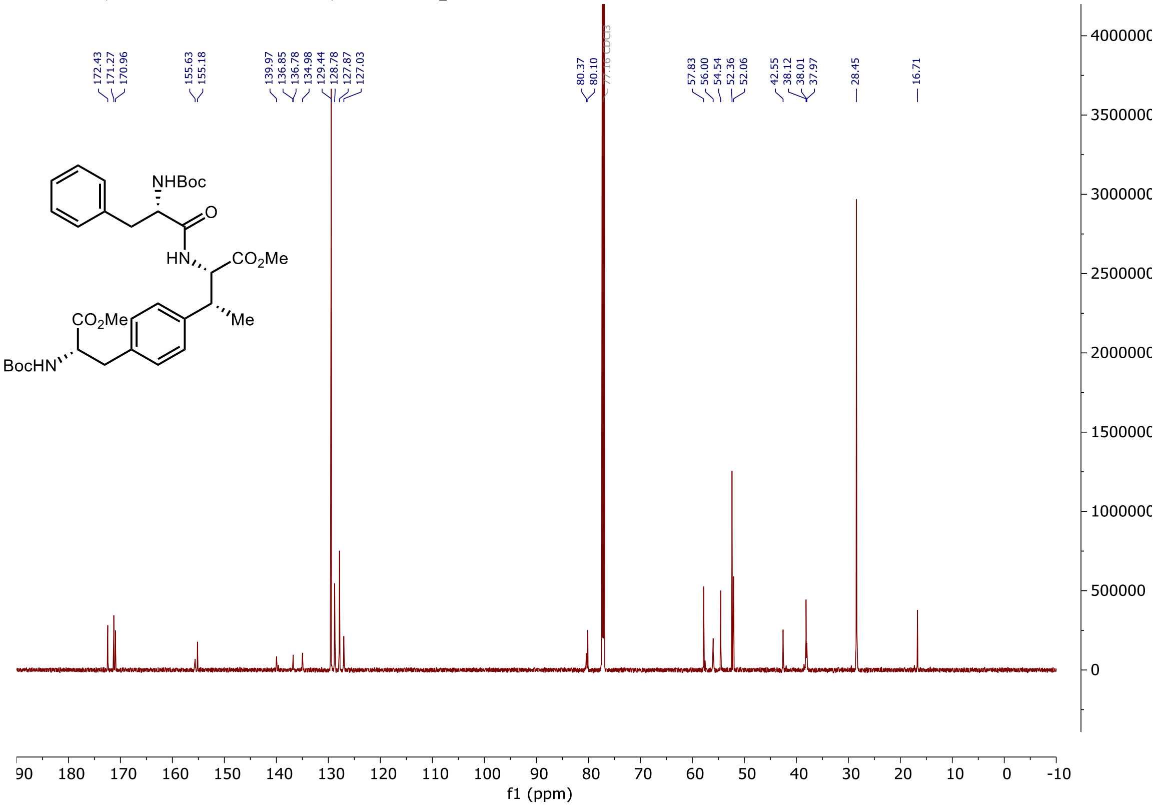

COSY (800 MHz, $CDCl_3$) of compound **22**

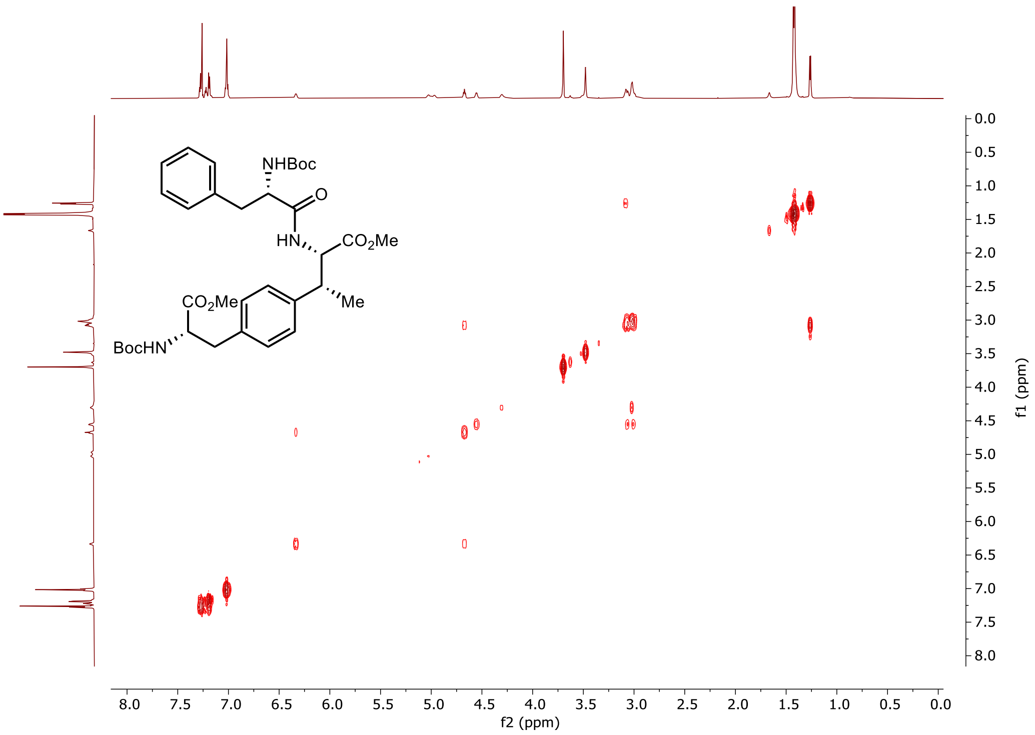

NOESY (800 MHz, $CDCl_3$) of compound **22**

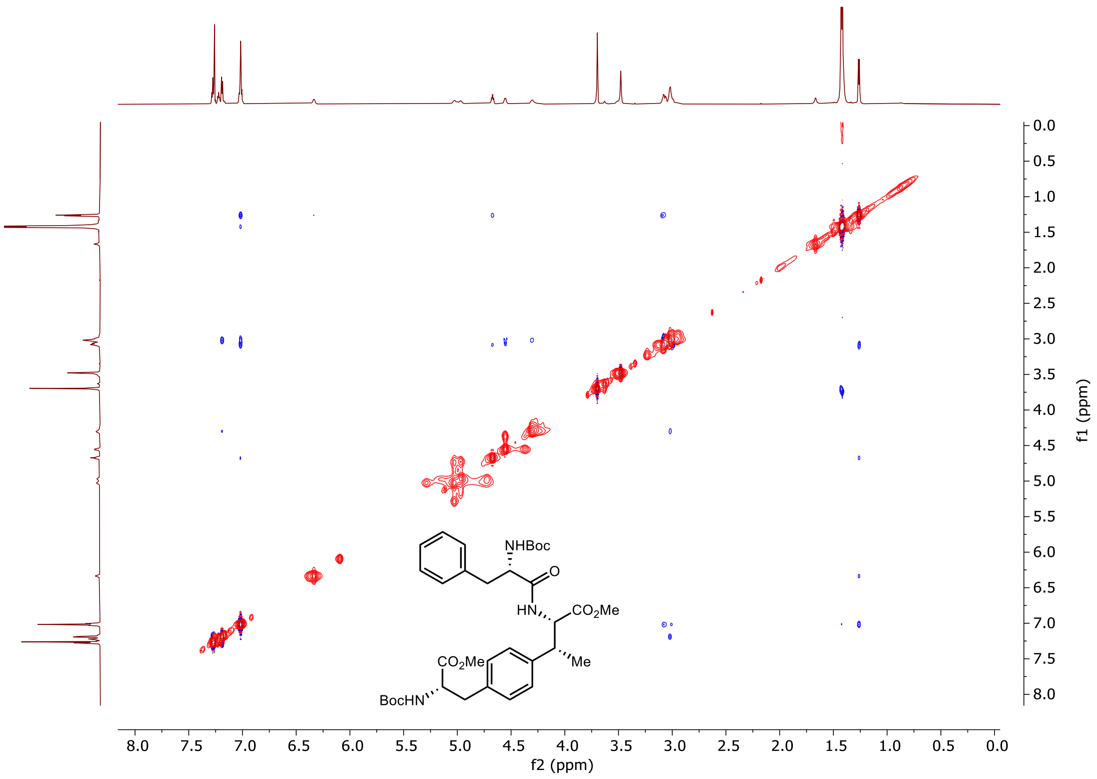

HMBC (800 MHz, $CDCl_3$) of compound **22**

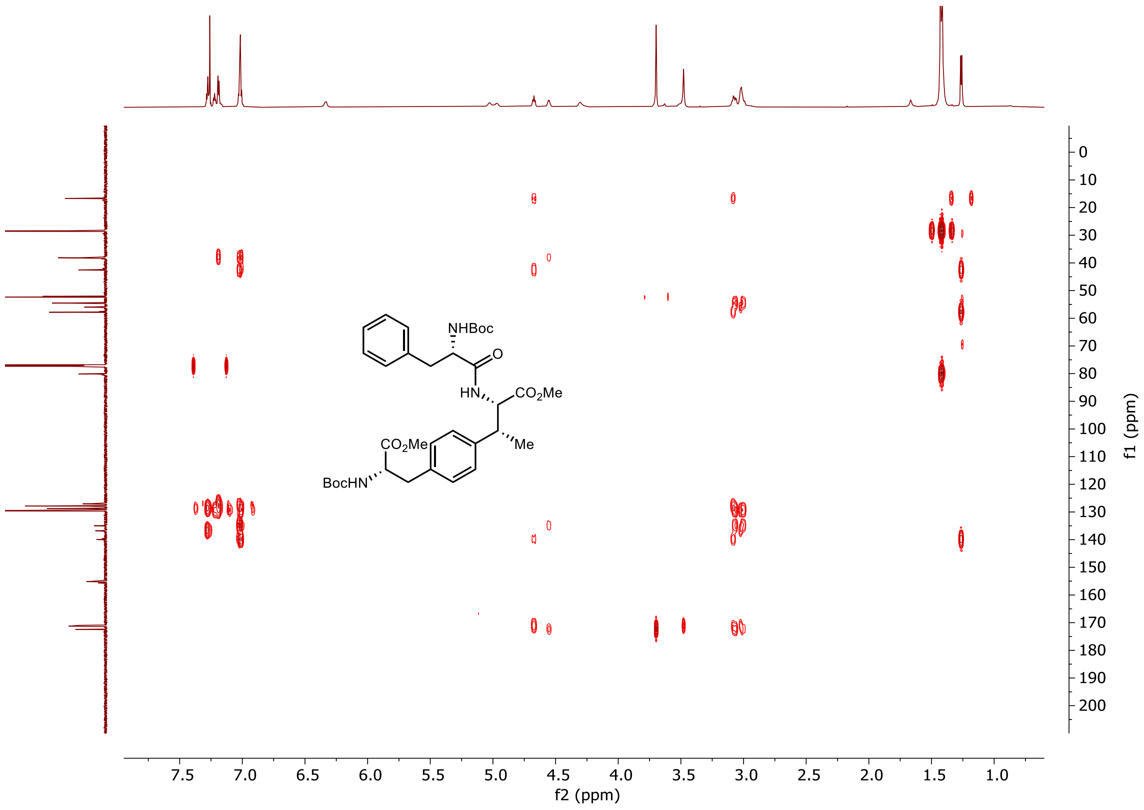

^{1}H NMR (600 MHz, $CDCl_3$, 55 °C) of compound **23** (12:1 isolated dr)

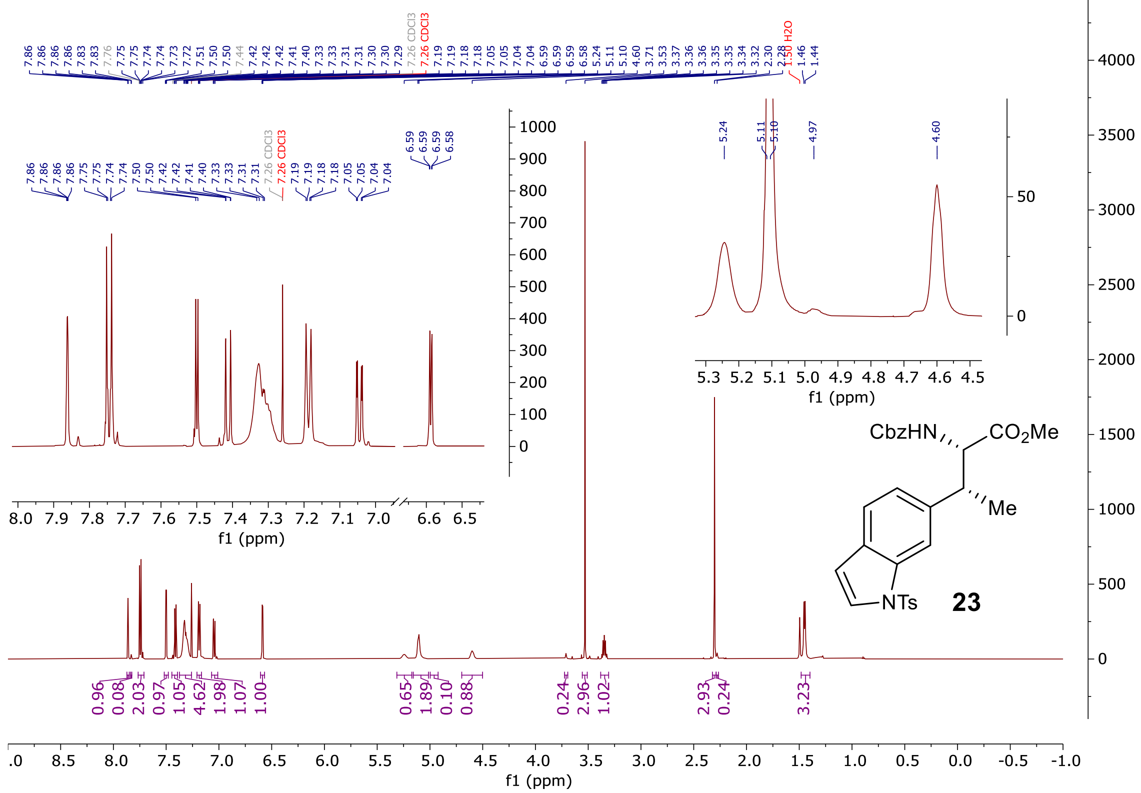

^{1}H NMR (600 MHz, $CDCl_3$, 25 °C) of compound **23** (rotamers visible)

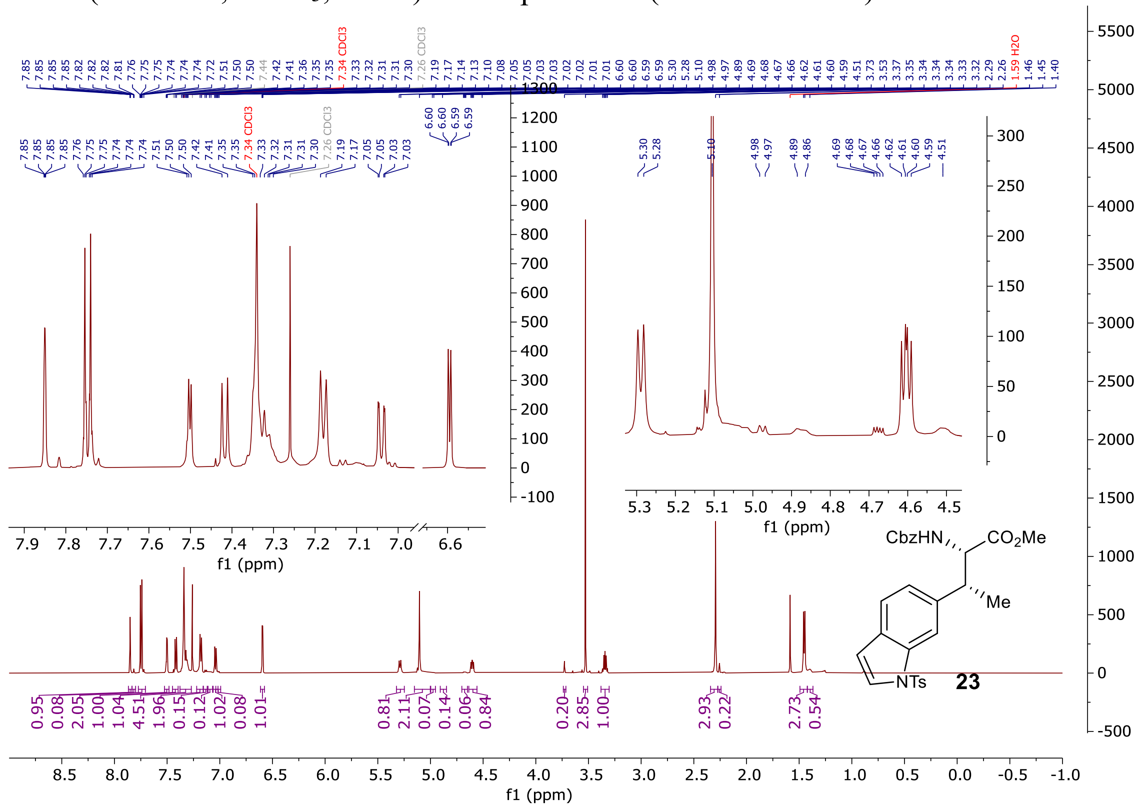

^{13}C NMR (151 MHz, $CDCl_3$) of compound **23**

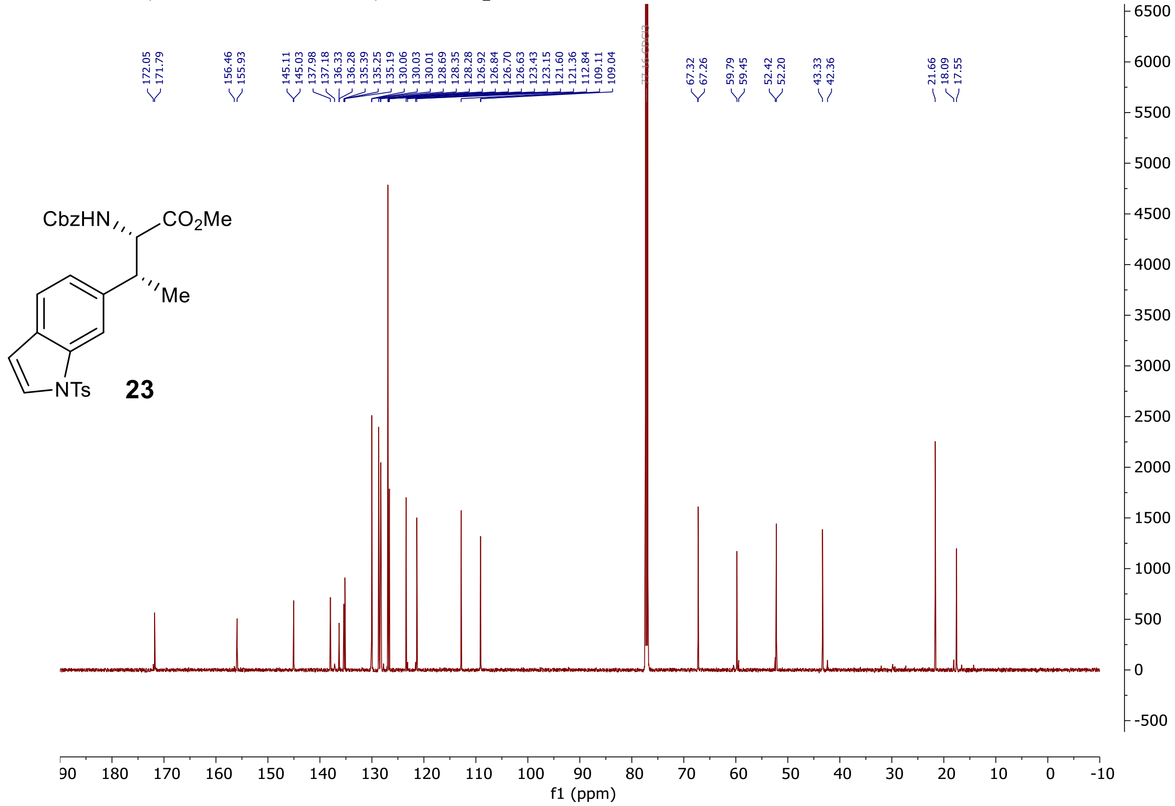

NOESY (600 MHz, $CDCl_3$) of compound **23**

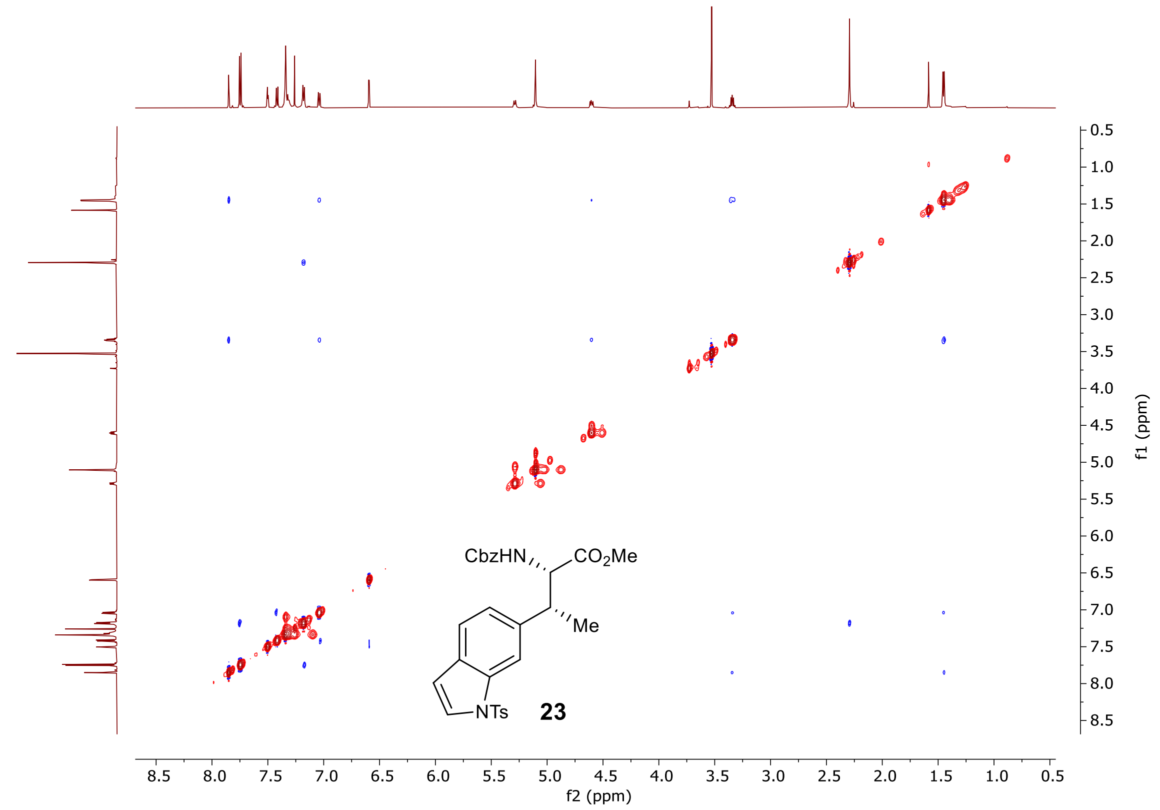

HMBC (600 MHz, $CDCl_3$) of compound **23**

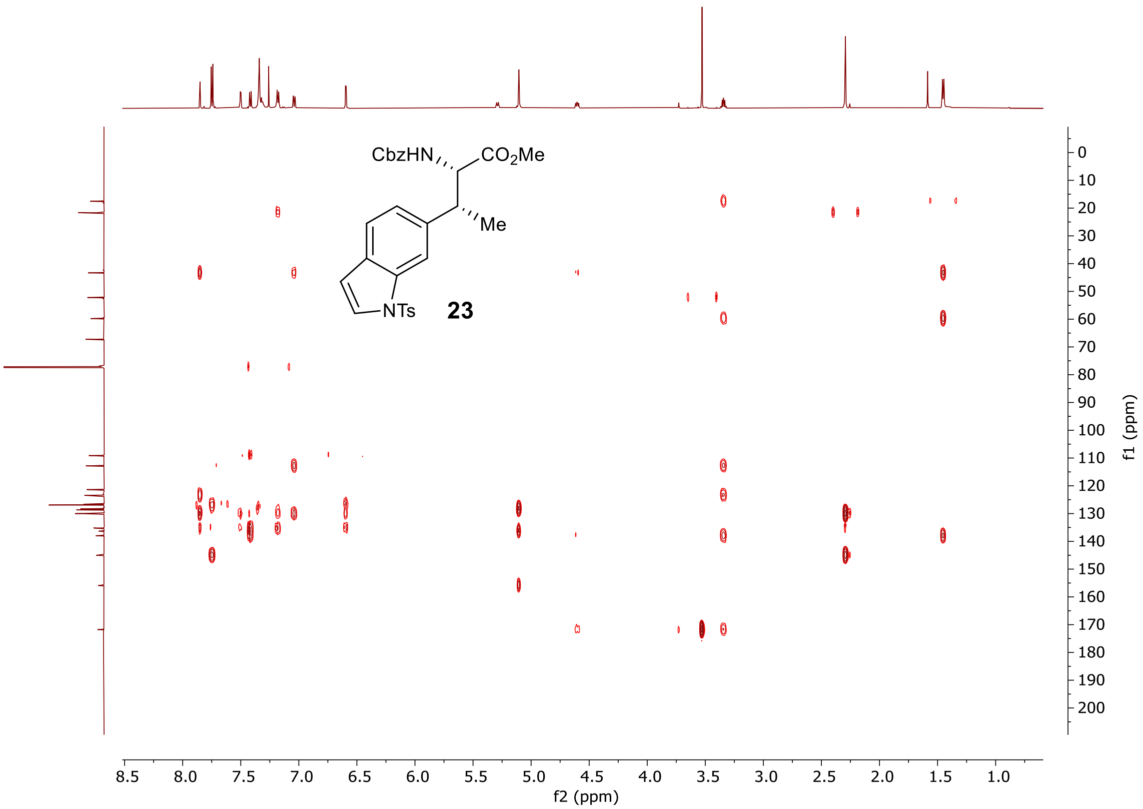

COSY (600 MHz, $CDCl_3$) of compound **23**

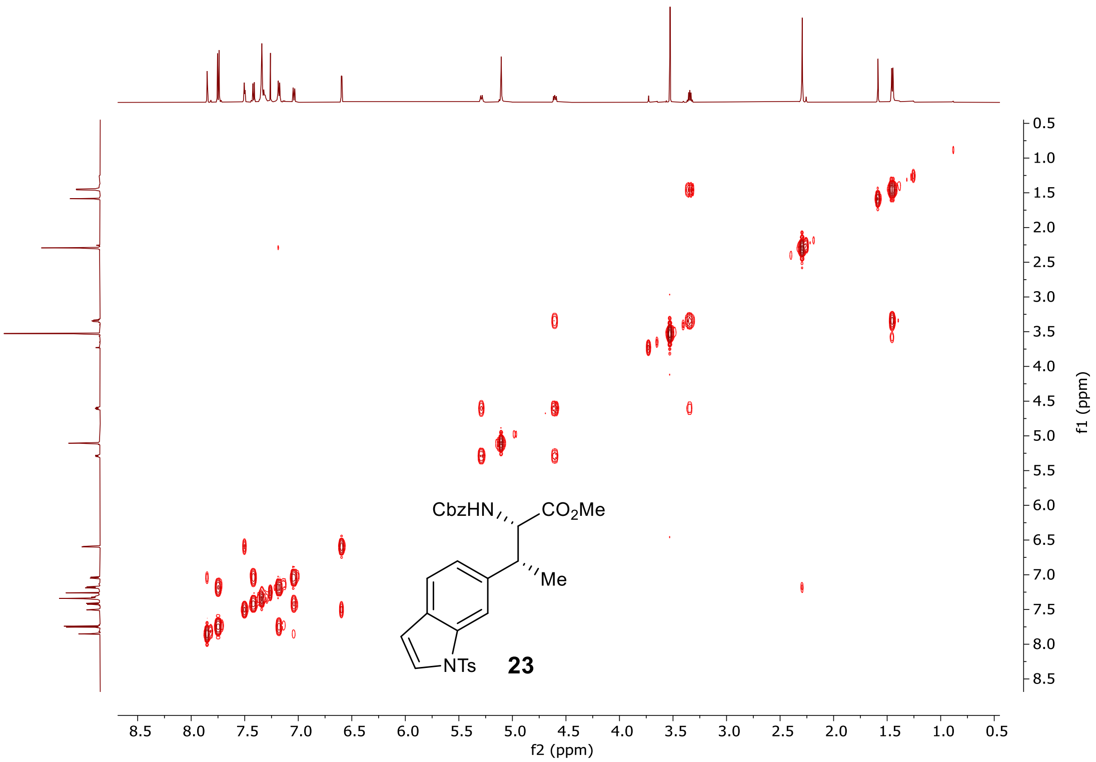

^{1}H NMR (400 MHz, $CDCl_3$, 25 °C) of compound **S51** Diastereomer 1

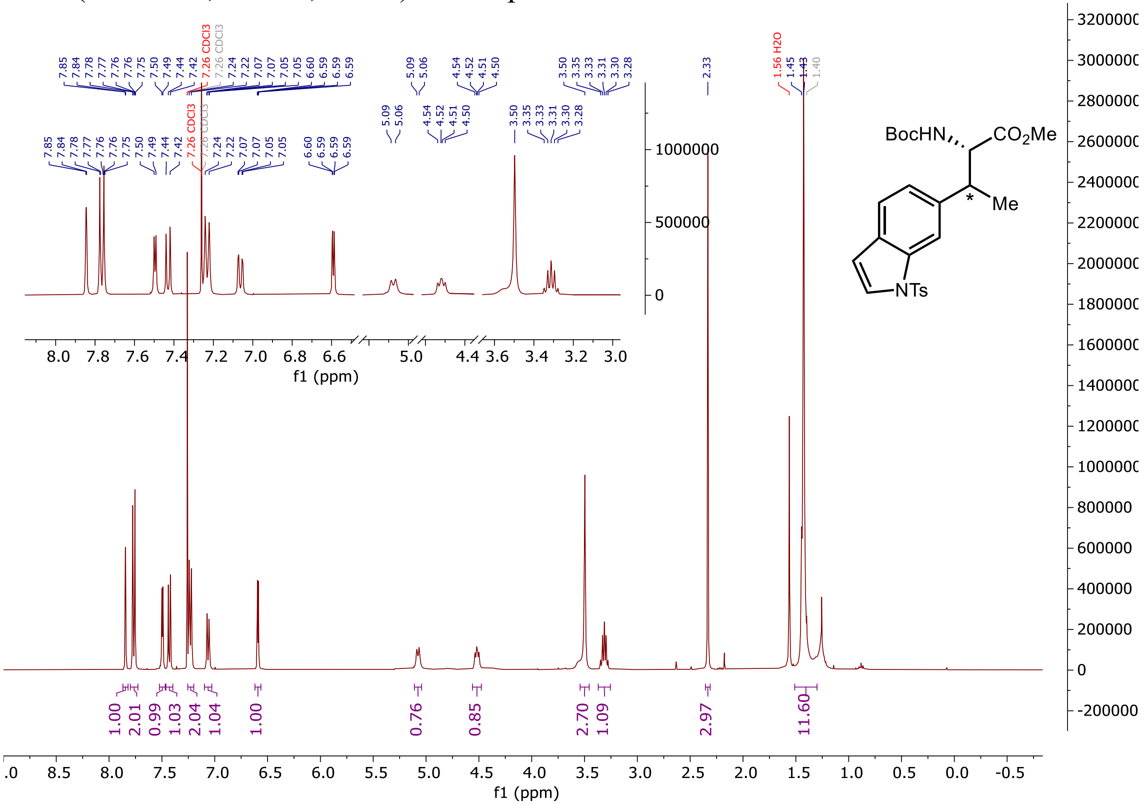

^{13}C NMR (151 MHz, $CDCl_3$) of compound **S51-D1**

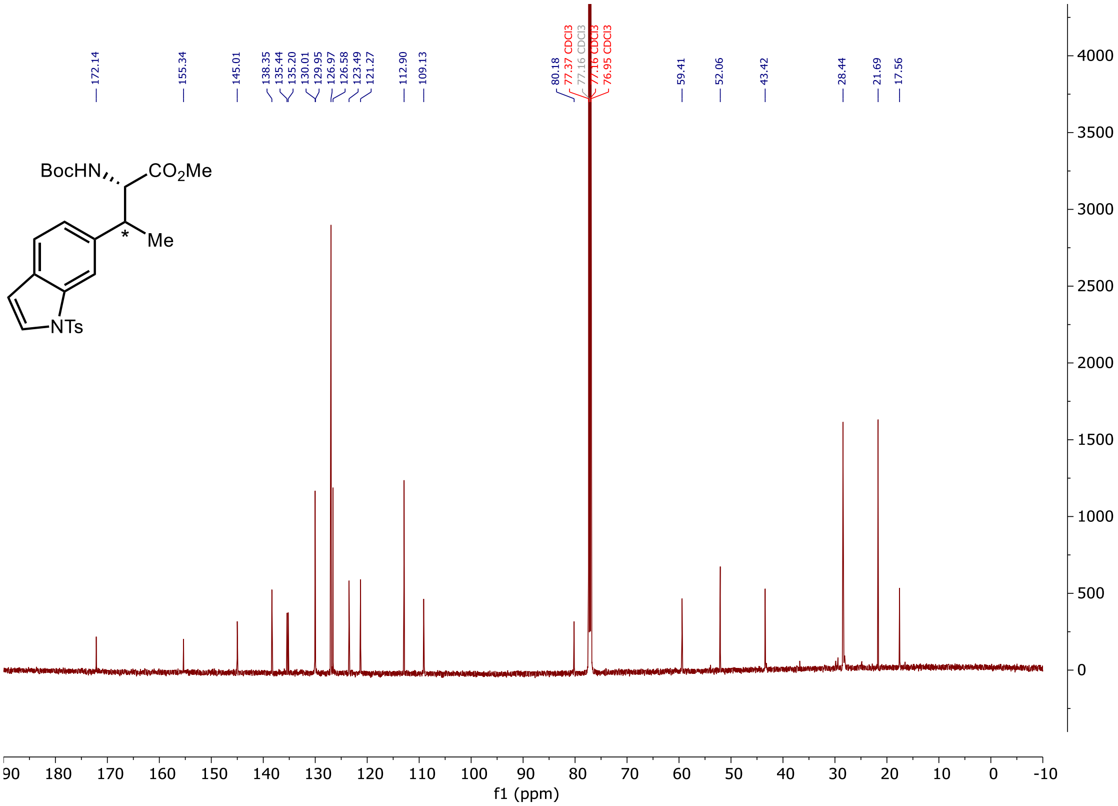

COSY (600 MHz, $CDCl_3$) of compound **S51** Diastereomer 1

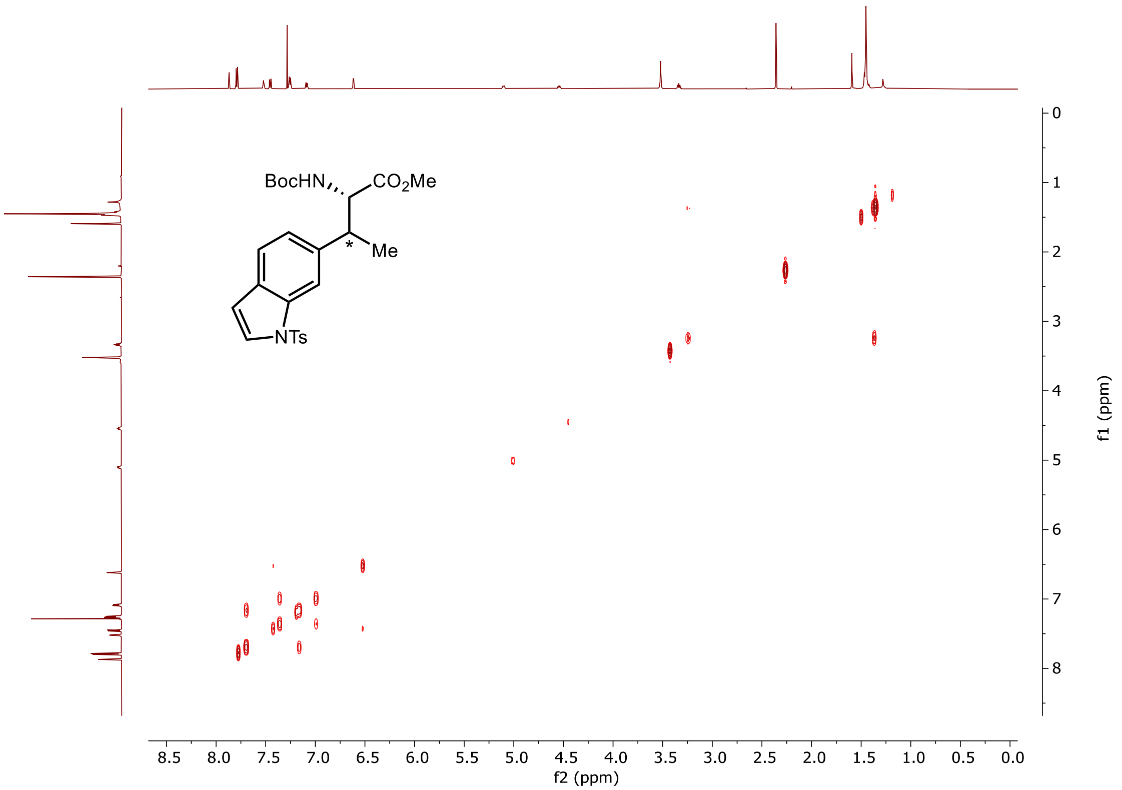

HMBC (600 MHz, $CDCl_3$) of compound **S51** Diastereomer 1

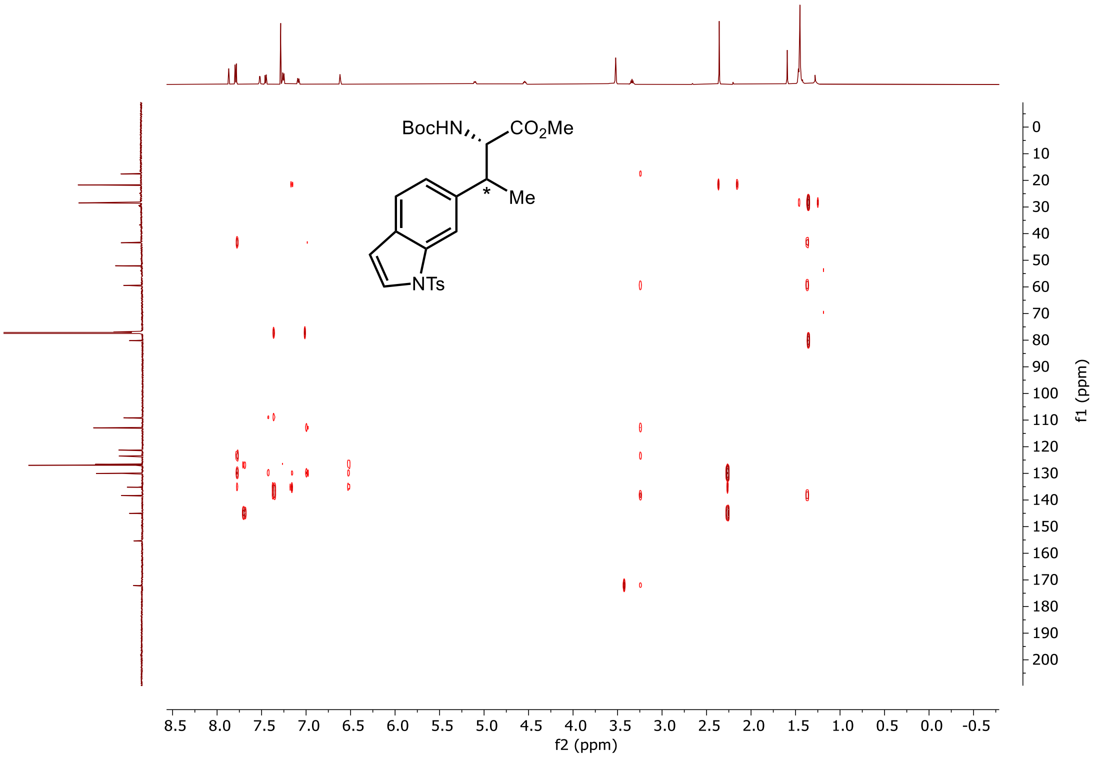

^{1}H NMR (400 MHz, $CDCl_3$, 25 °C) of compound **S51** Diastereomer 2

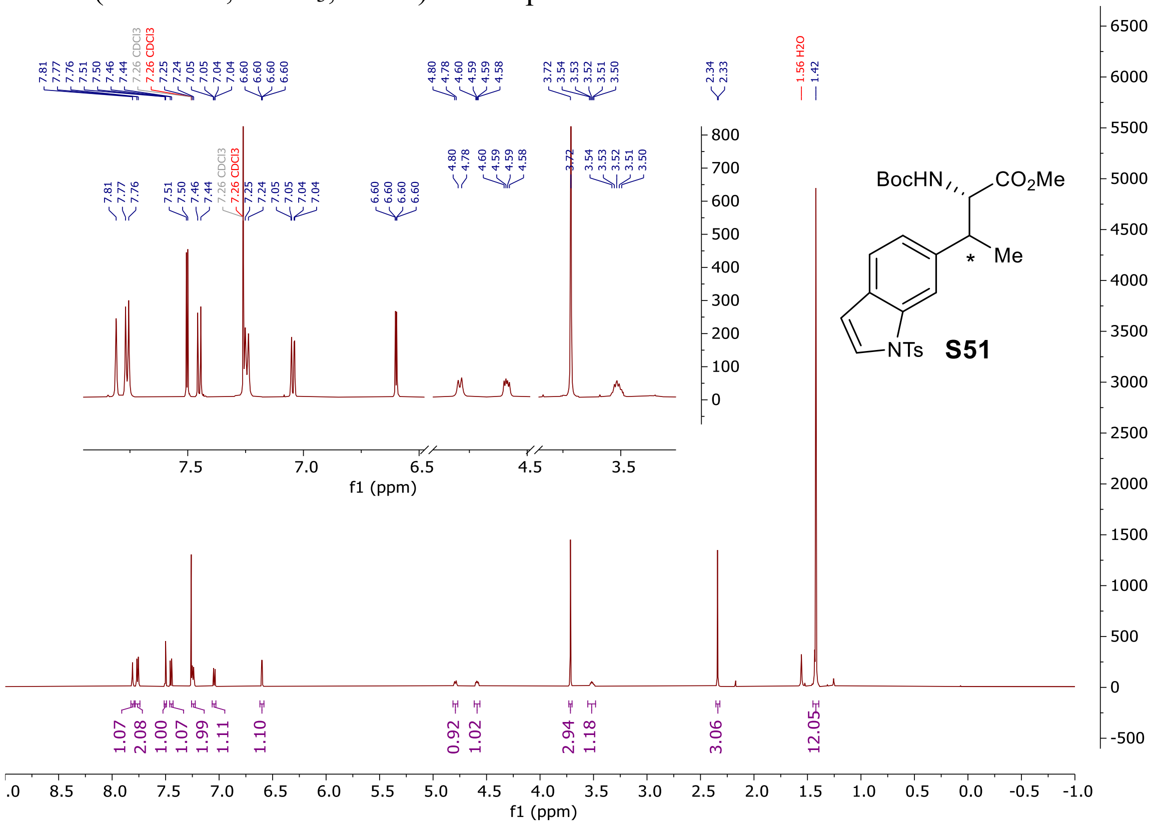

^{13}C NMR (151 MHz, $CDCl_3$) of compound **S51** Diastereomer 2

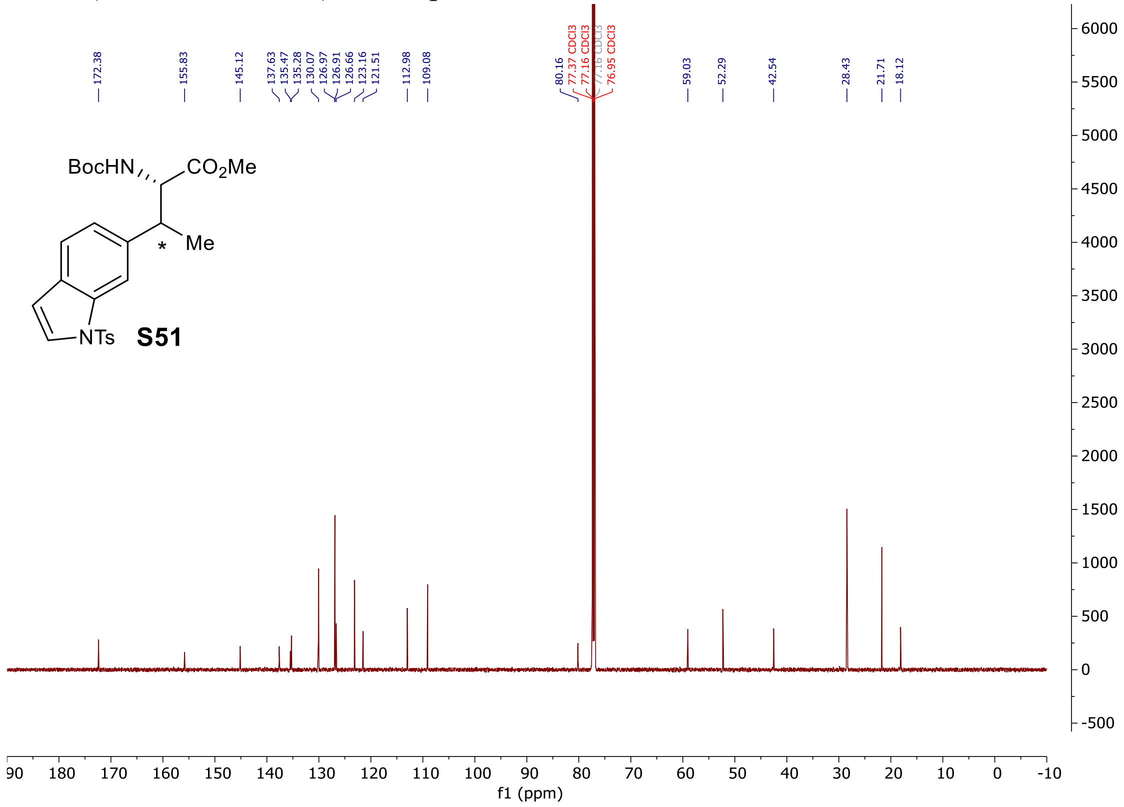

COSY (600 MHz, $CDCl_3$) of compound **S51** Diastereomer 2

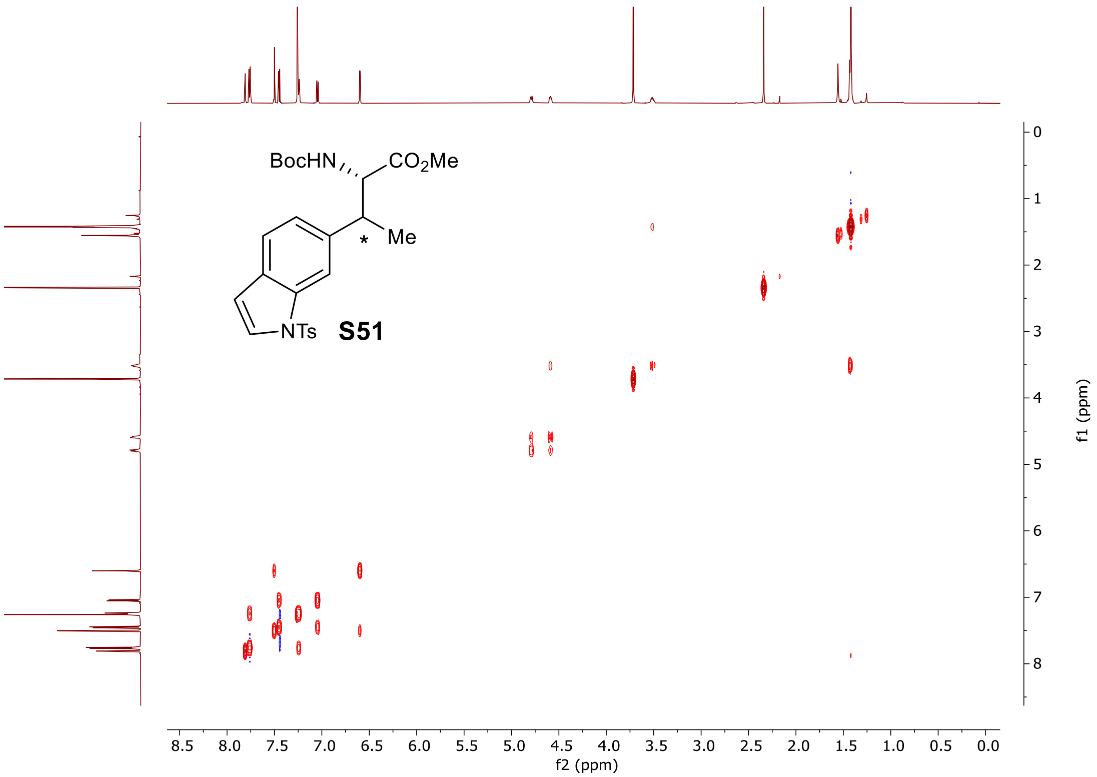

NOESY (600 MHz, $CDCl_3$) of compound **S51** Diastereomer 2

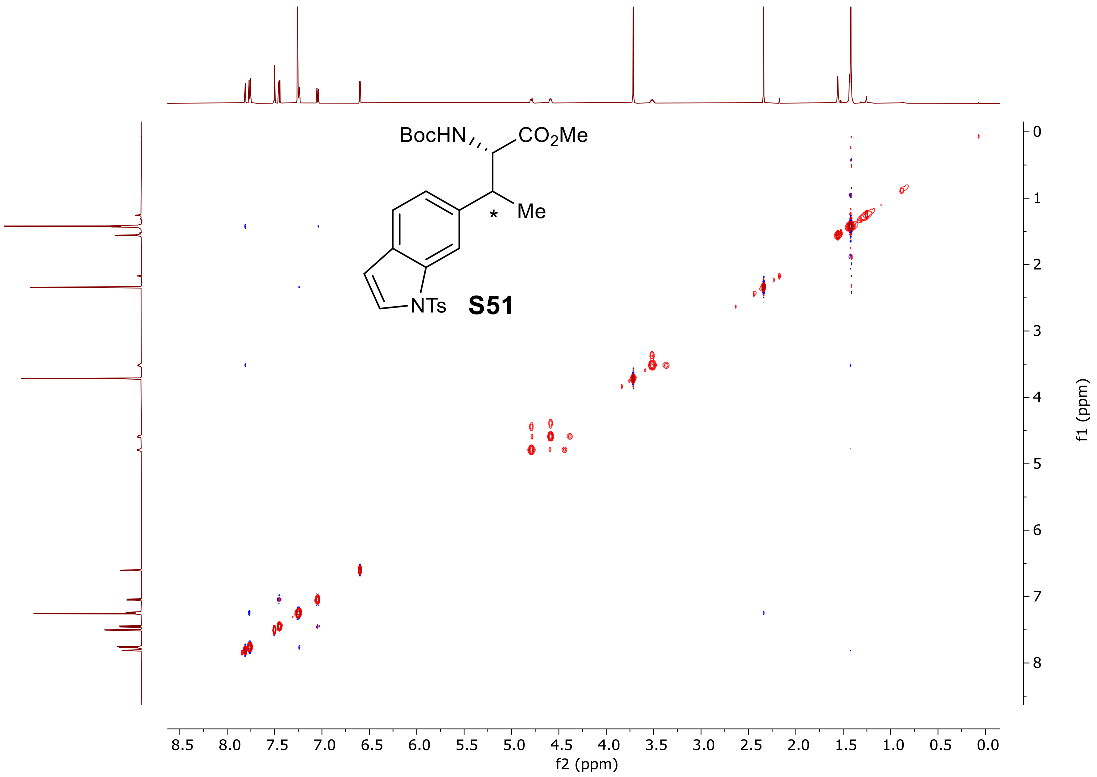

HMBC (600 MHz, $CDCl_3$) of compound **S51** Diastereomer 2

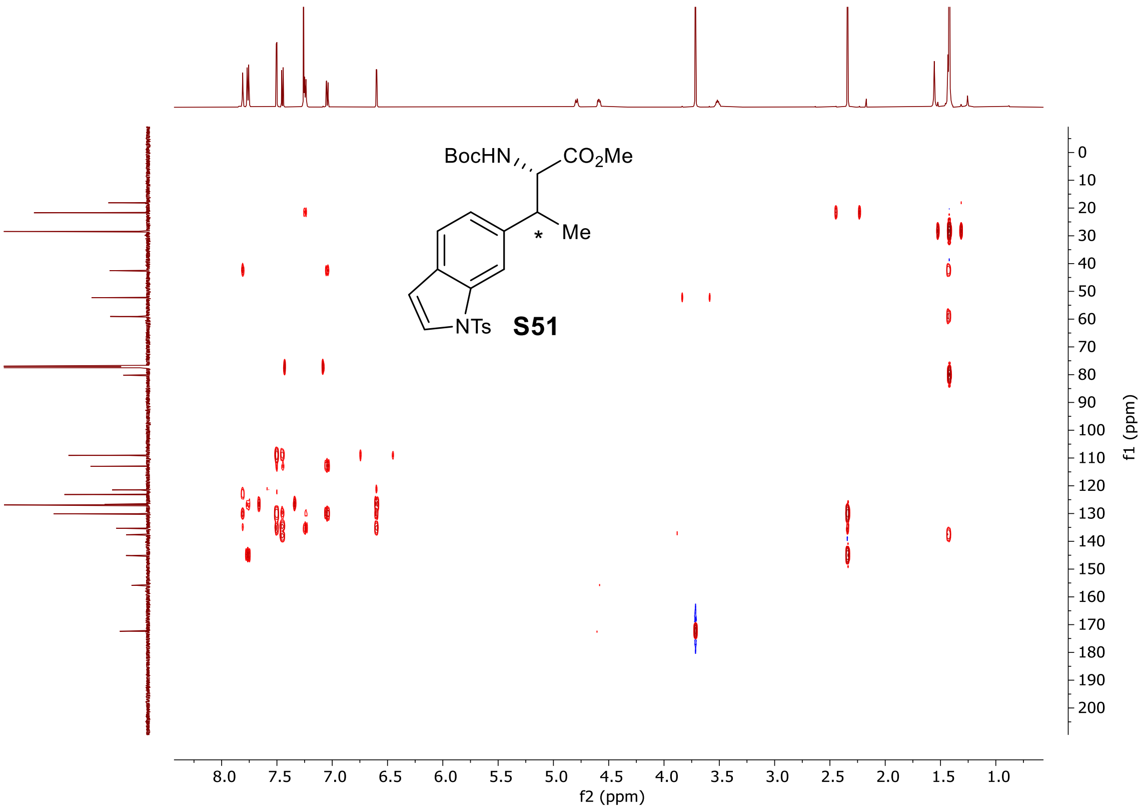

^{1}H NMR (800 MHz, $CDCl_3$, 25 °C) of compound **S52** (3:1 isolated dr)

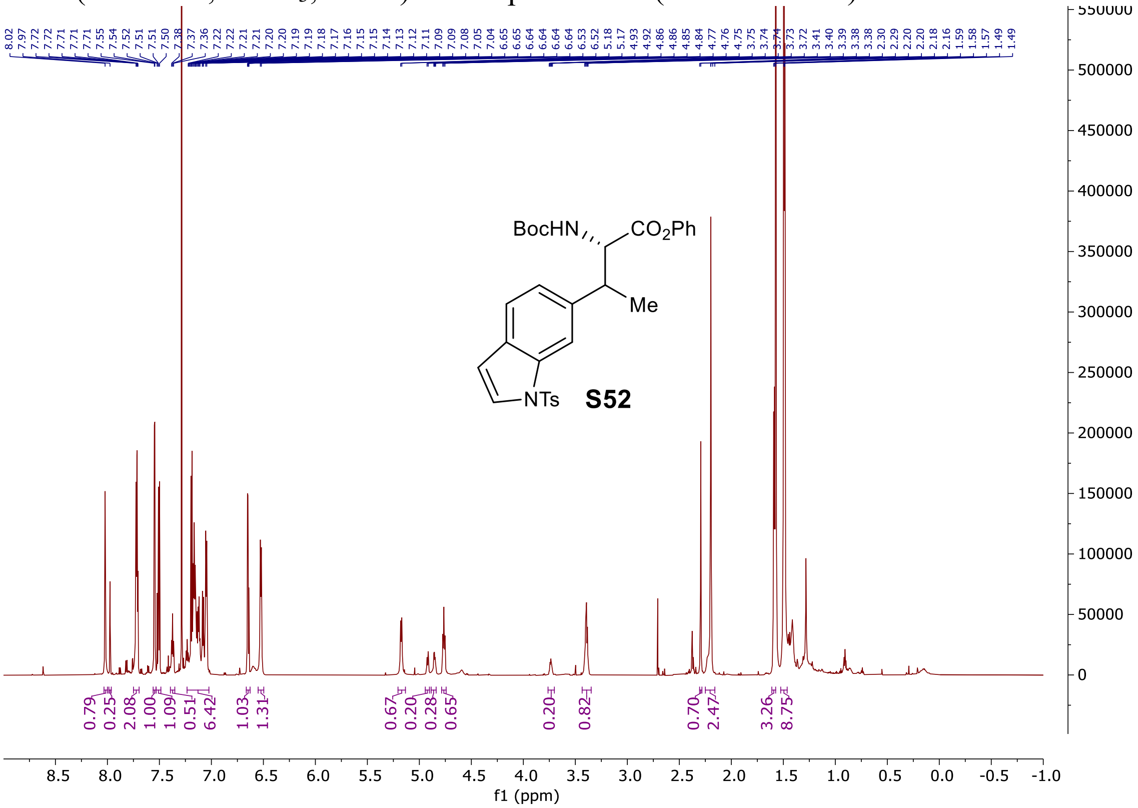

^{13}C NMR (201 MHz, $CDCl_3$) of compound **S52**

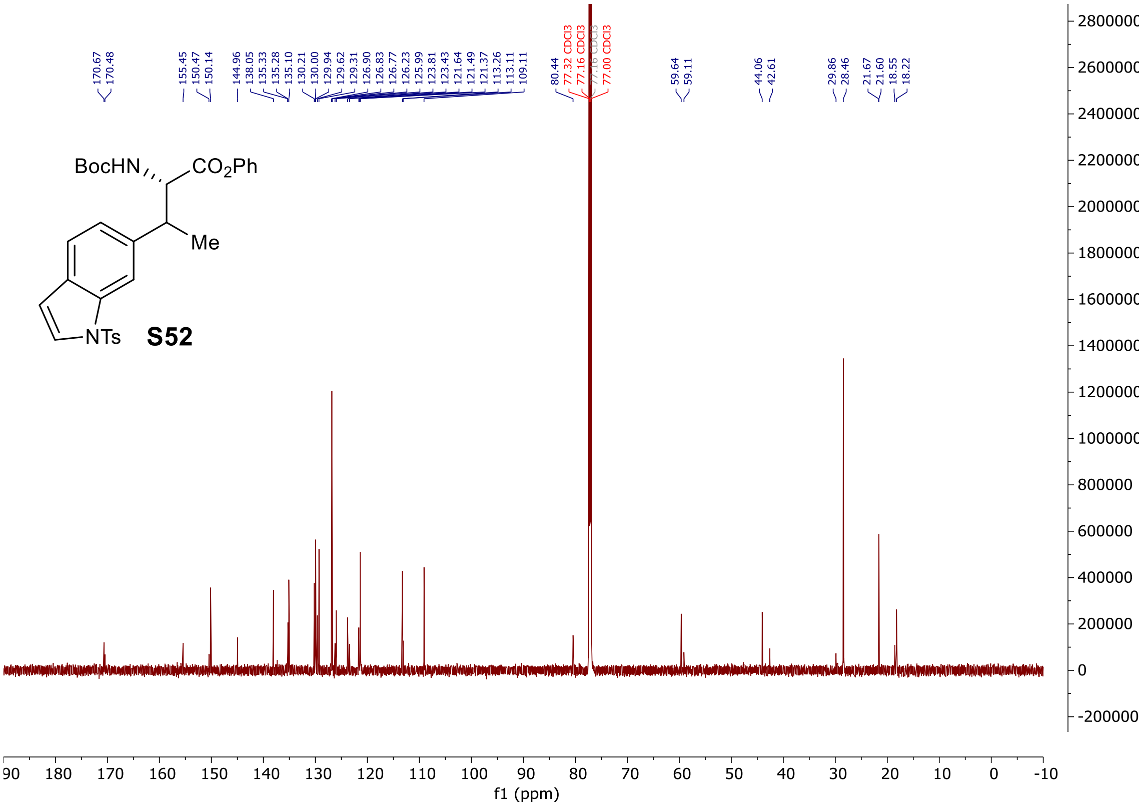

COSY (800 MHz, $CDCl_3$) of compound **S52**

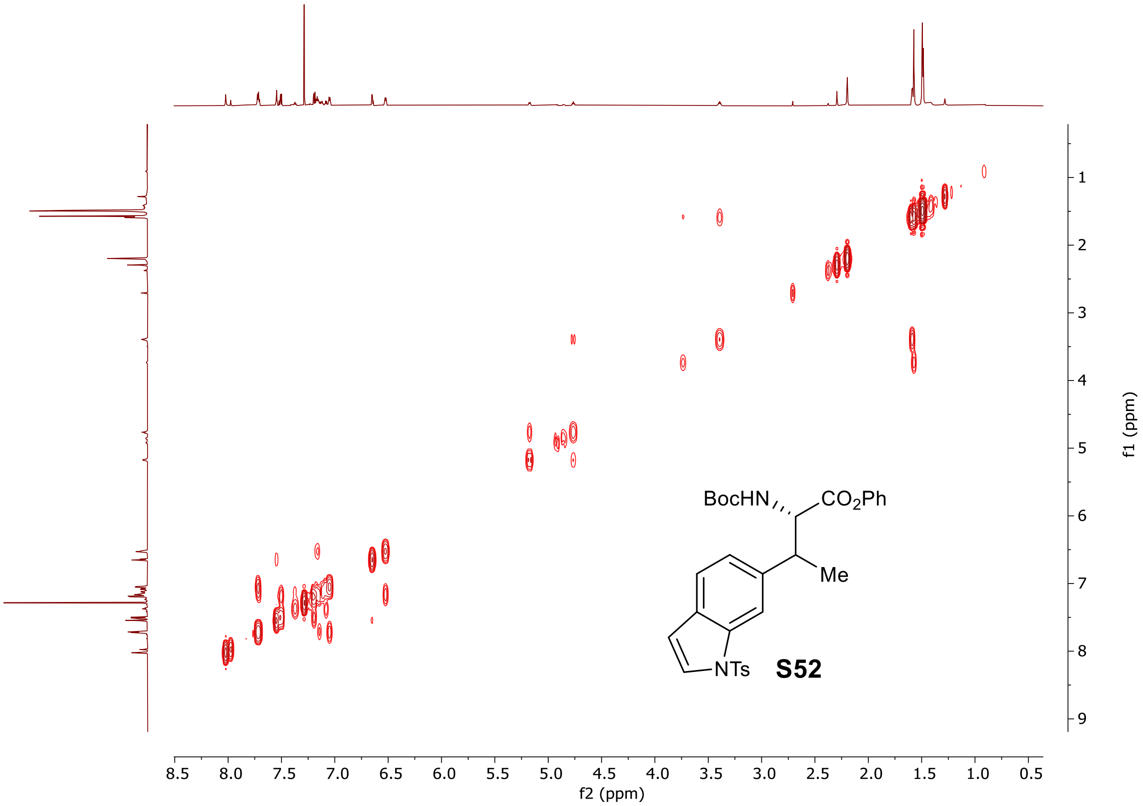

HMBC (800 MHz, $CDCl_3$) of compound **S52**

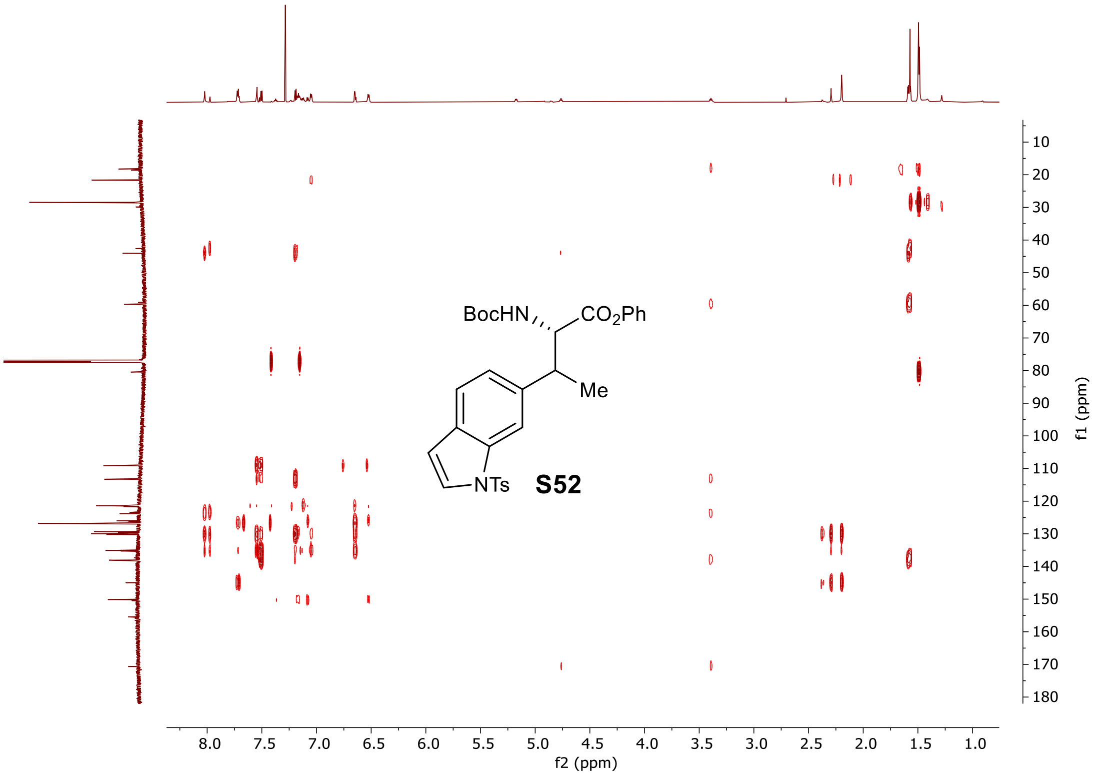

NOESY (800 MHz, $CDCl_3$) of compound **S52**

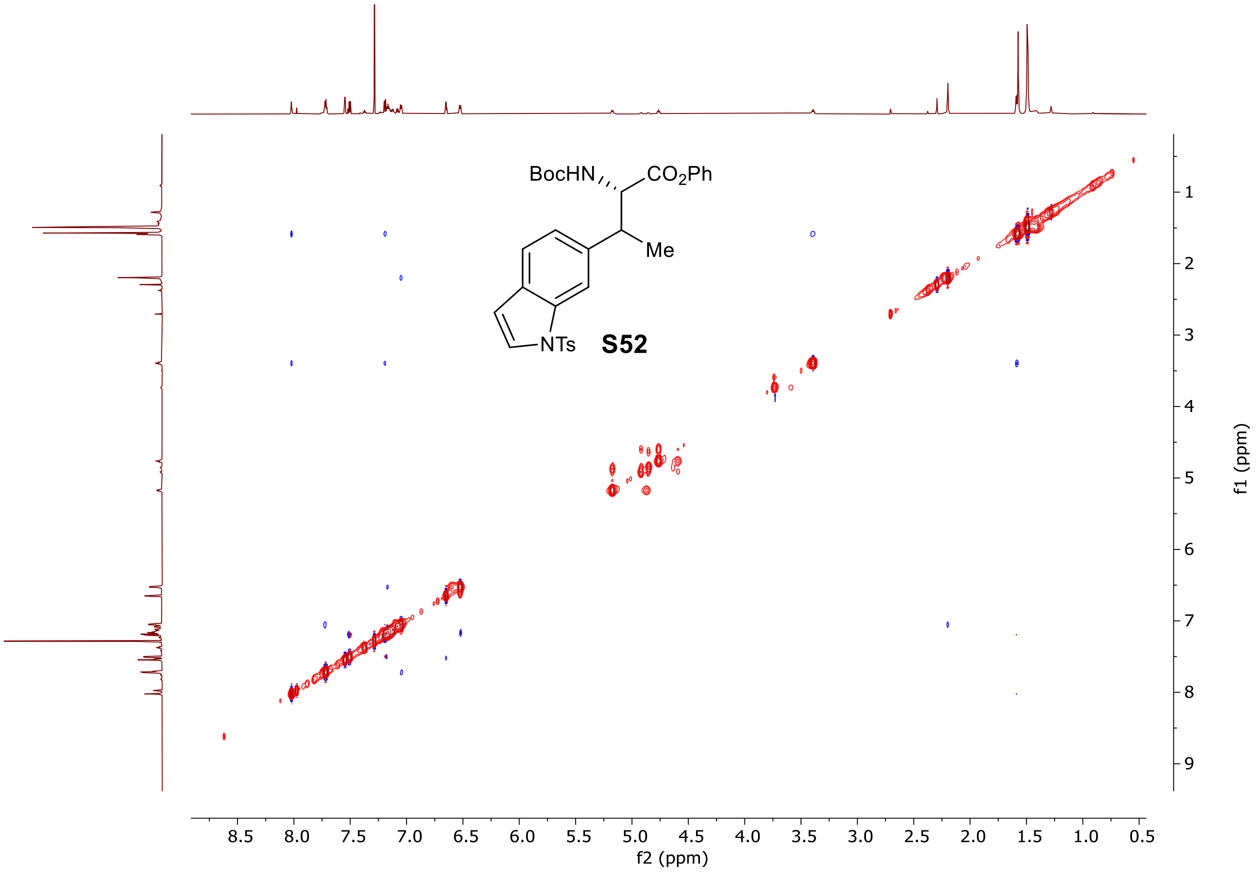

^{1}H NMR (800 MHz, $CDCl_3$, 25 °C) of compound **S53** (1:1 isolated dr)

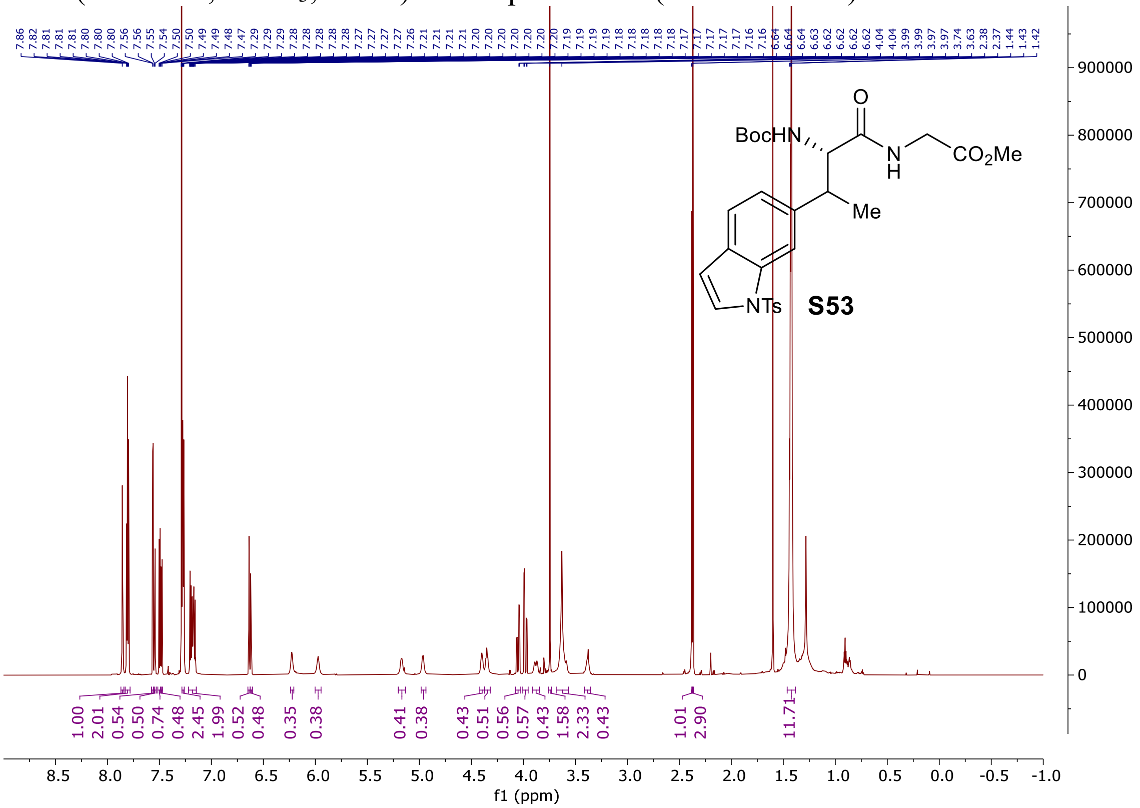

^{13}C NMR (201 MHz, $CDCl_3$) of compound **S53**

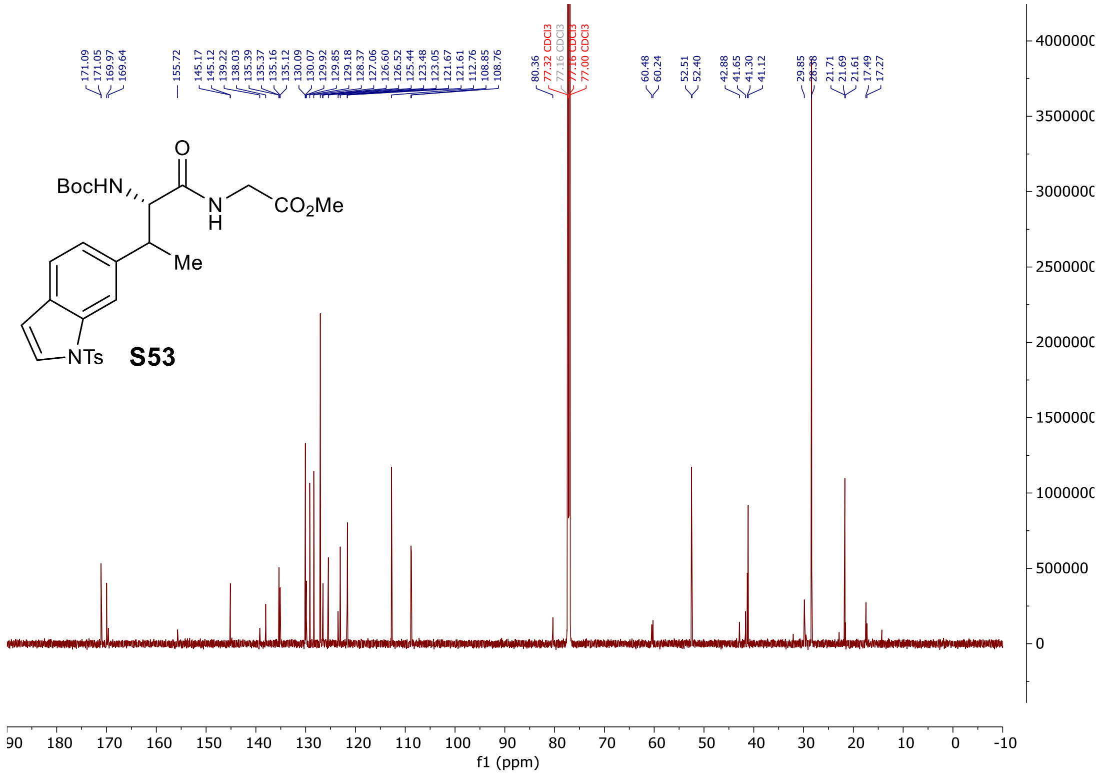

COSY (800 MHz, $CDCl_3$) of compound **S53**

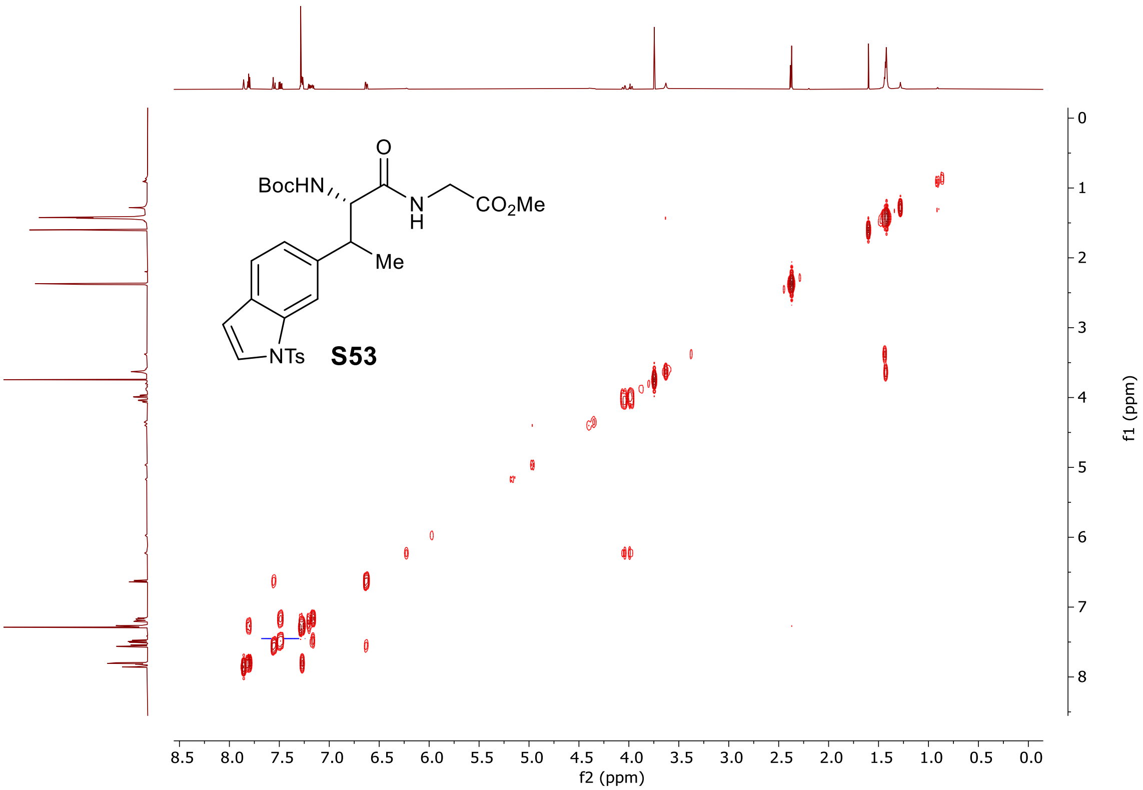

NOESY (800 MHz, $CDCl_3$) of compound **S53**

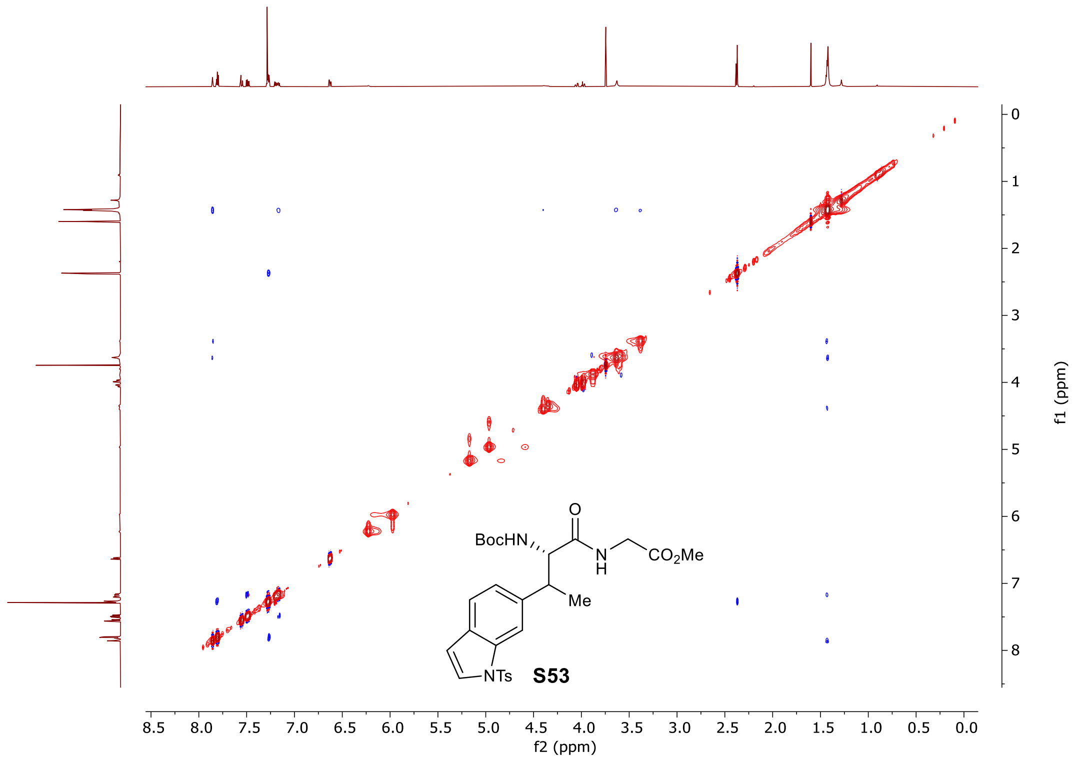

HMBC (800 MHz, $CDCl_3$) of compound **S53**

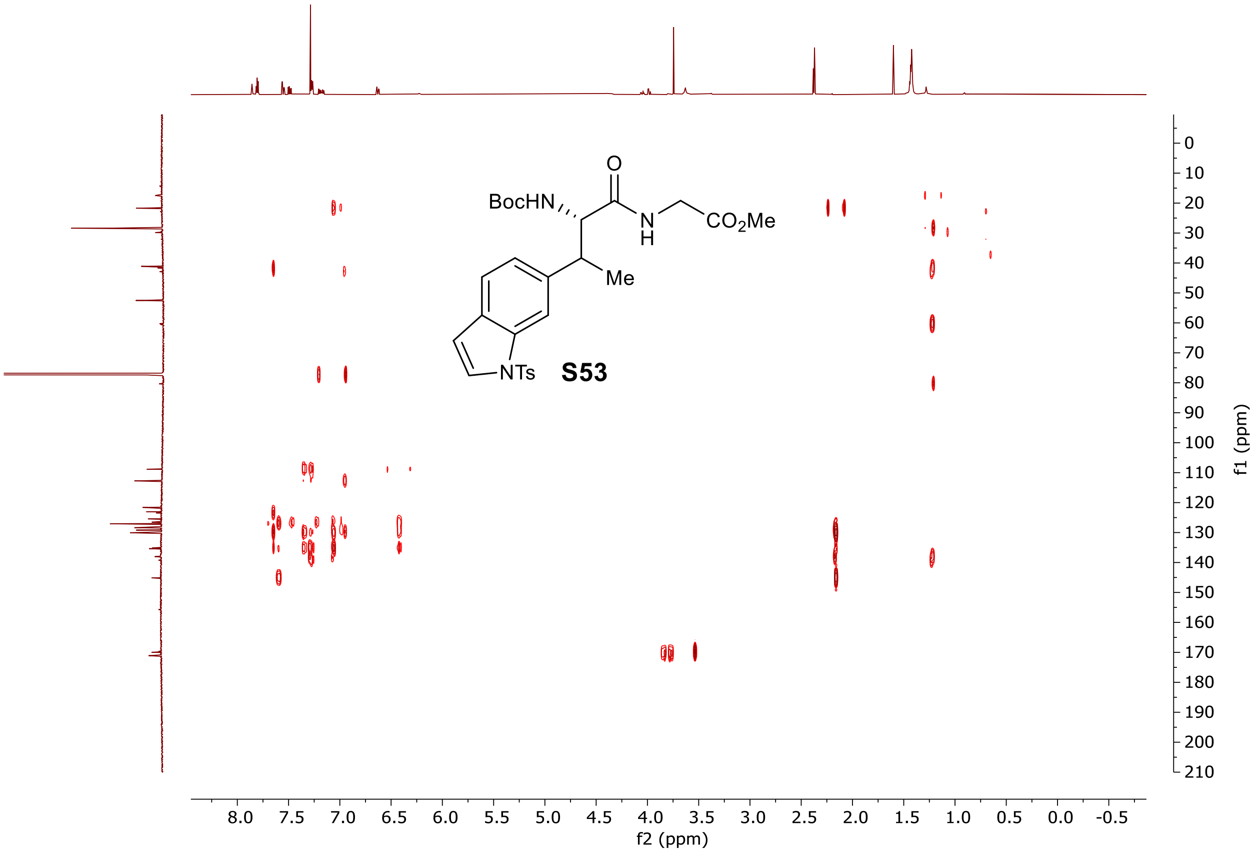

^{1}H NMR (400 MHz, $CDCl_3$, 25 °C) of compound **S54-D1**

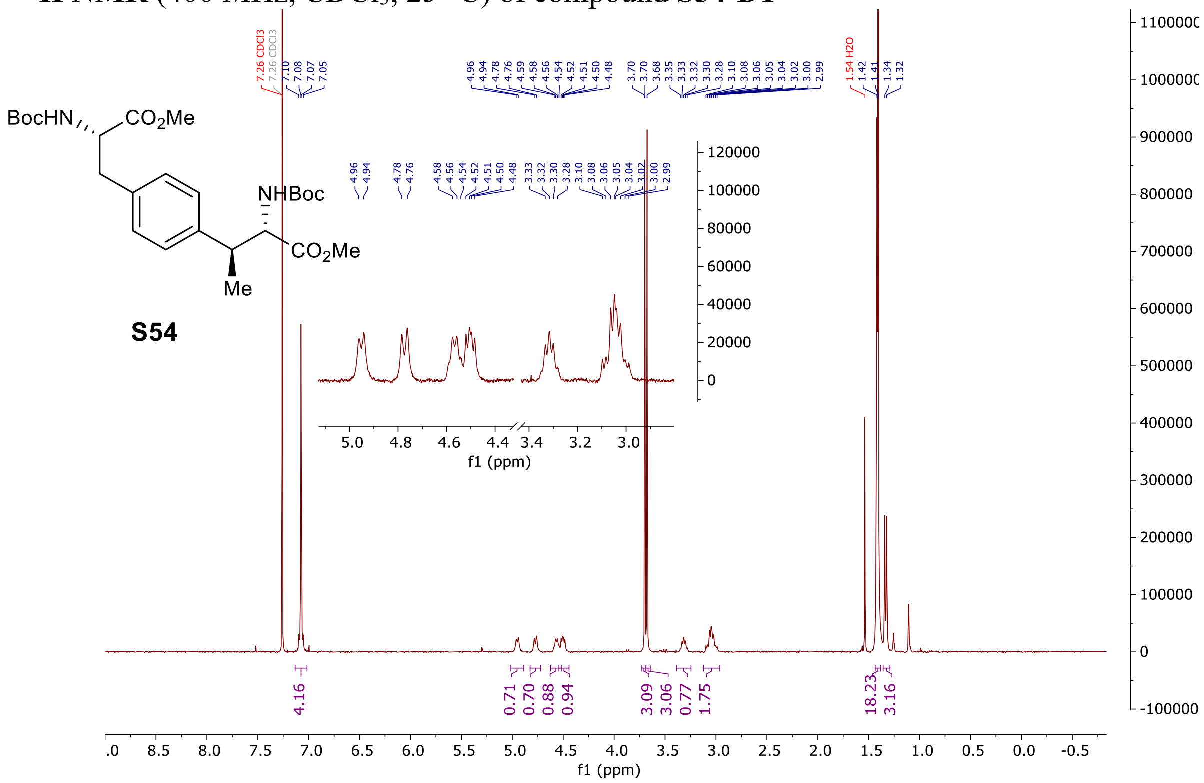

^{13}C NMR (201 MHz, $CDCl_3$) of compound **S54-D1**

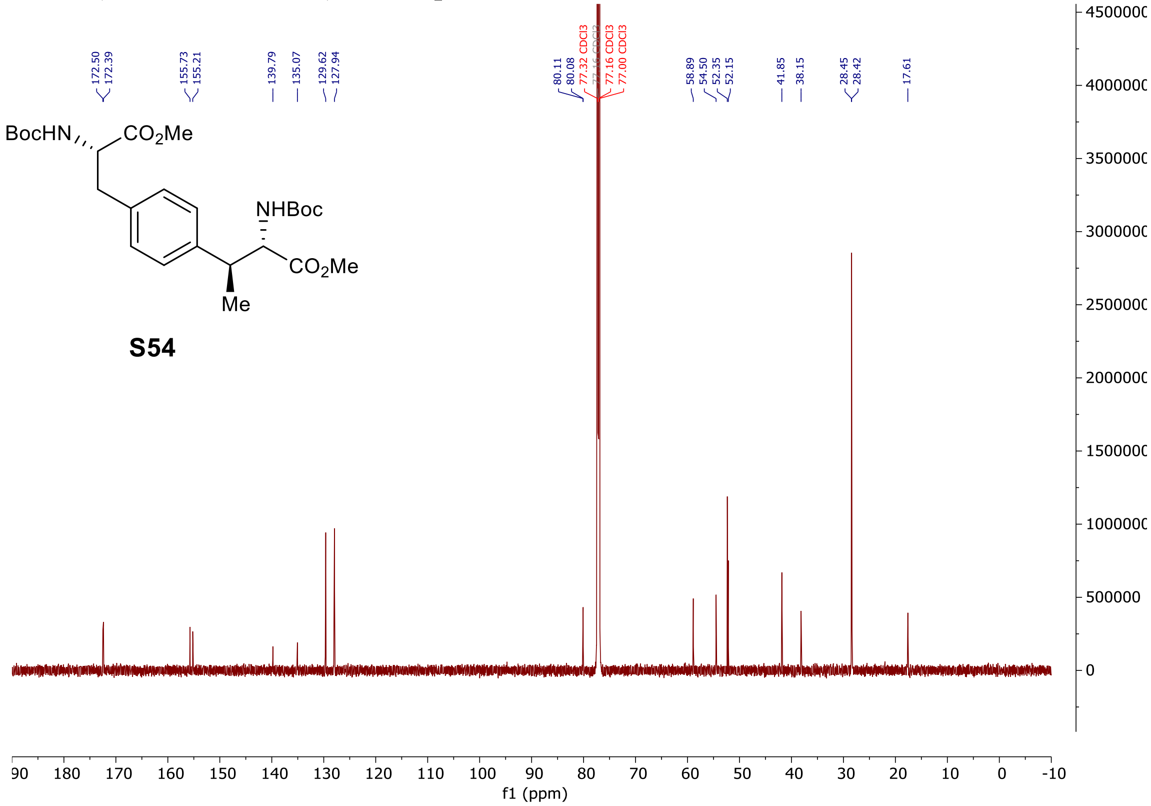

NOESY (800 MHz, $CDCl_3$) of compound **S54-D1**

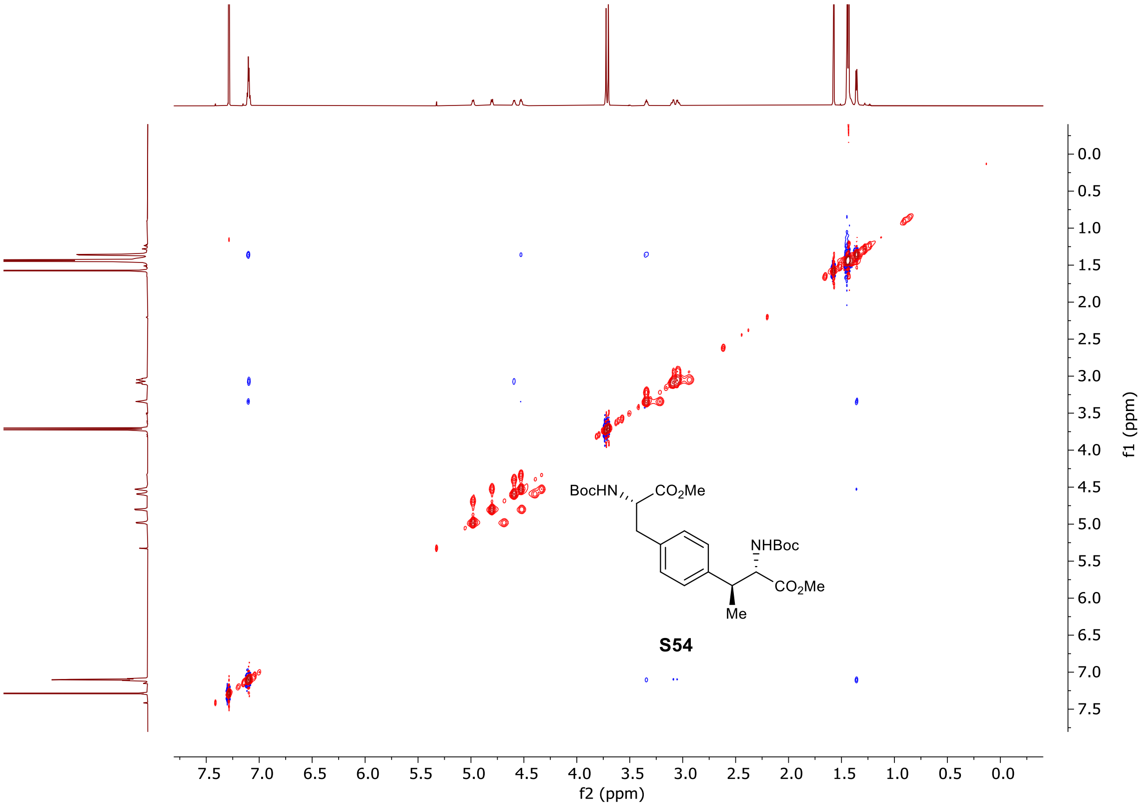

COSY (800 MHz, $CDCl_3$) of compound **S54-D1**

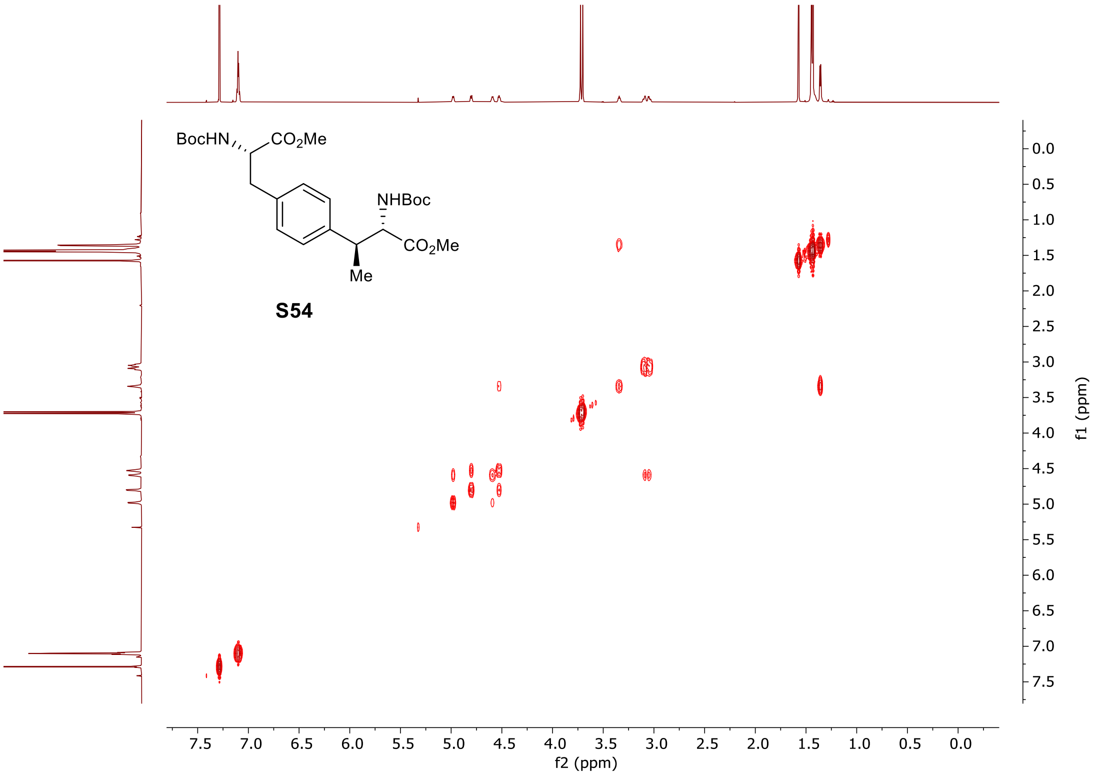

HMBC (800 MHz, $CDCl_3$) of compound **S54-D1**

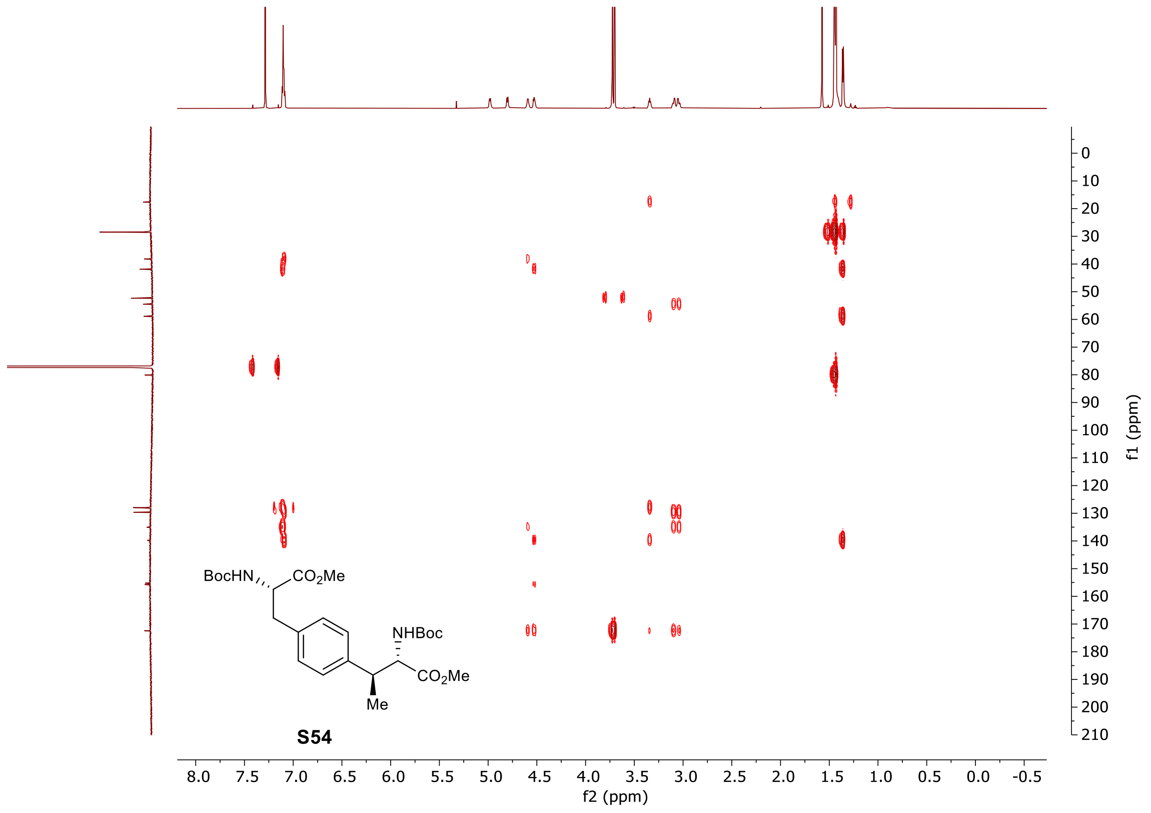

TOCSY (800 MHz, $CDCl_3$) of compound **S54-D1**

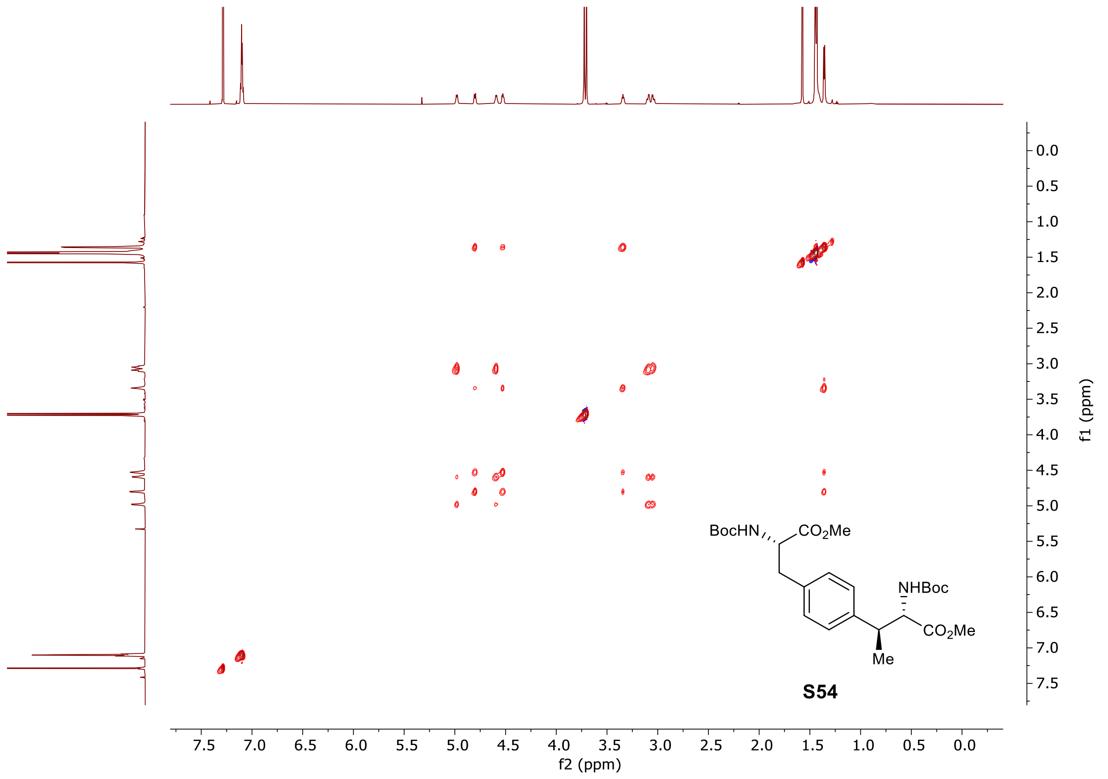

^{1}H NMR (400 MHz, $CDCl_3$, 25 °C) of compound **S54-D2**

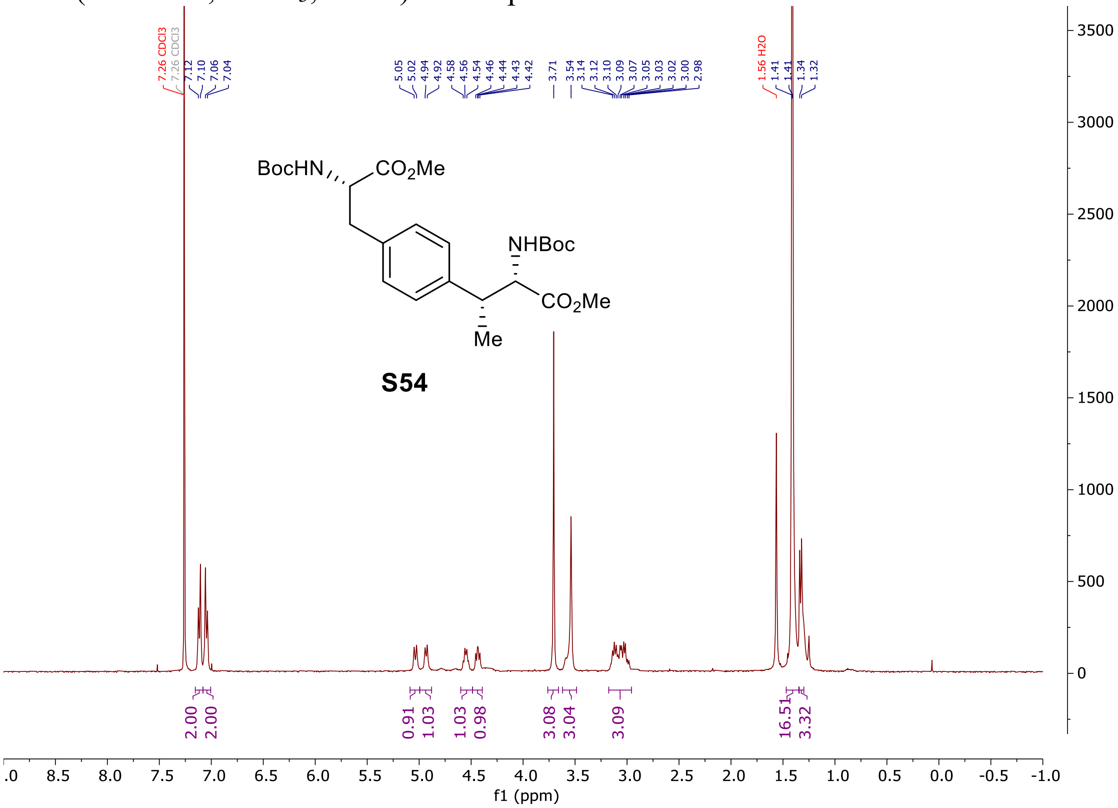

^{13}C NMR (151 MHz, $CDCl_3$) of compound **S54-D2**

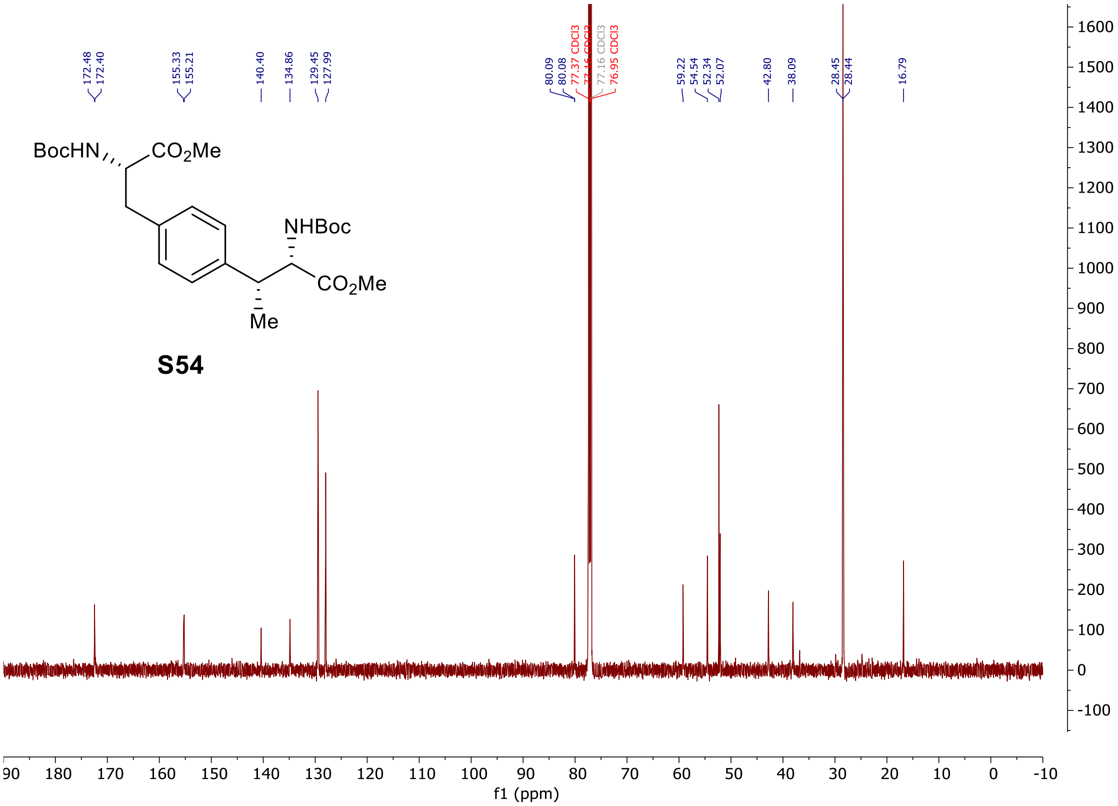

NOESY (800 MHz, $CDCl_3$) of compound **S54-D2**

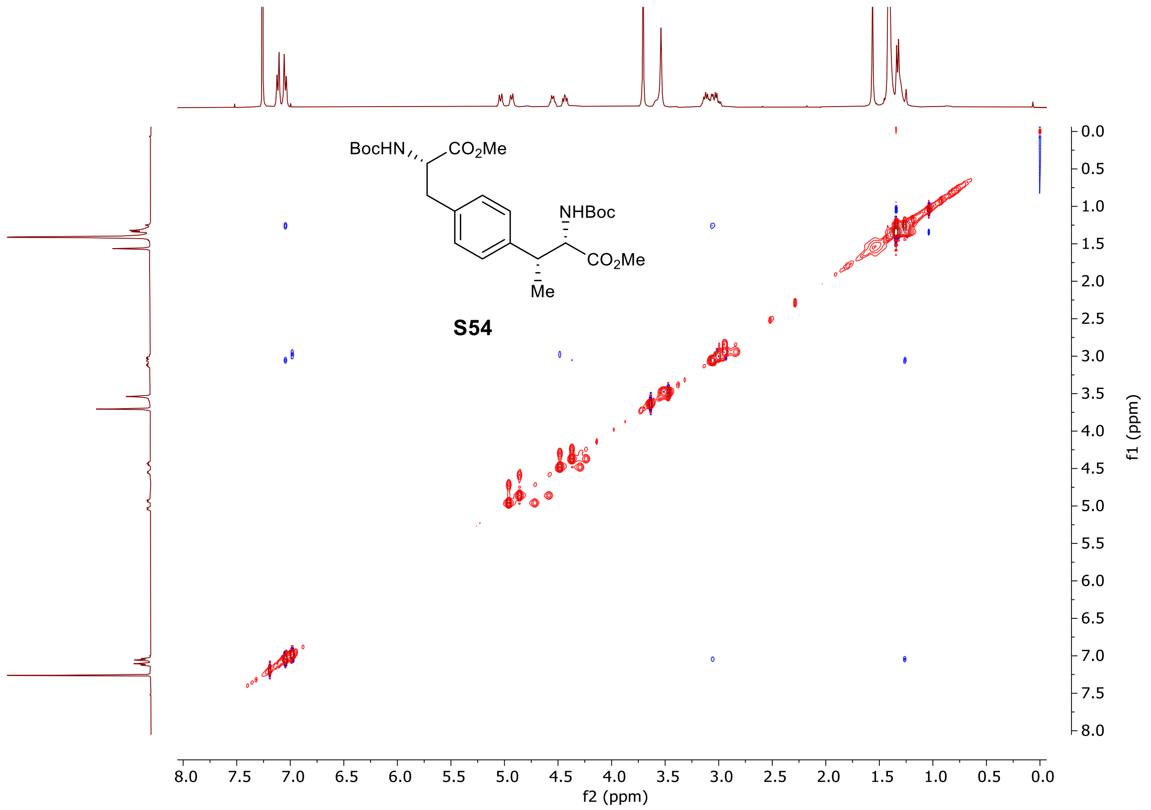

COSY (800 MHz, $CDCl_3$) of compound **S54-D2**

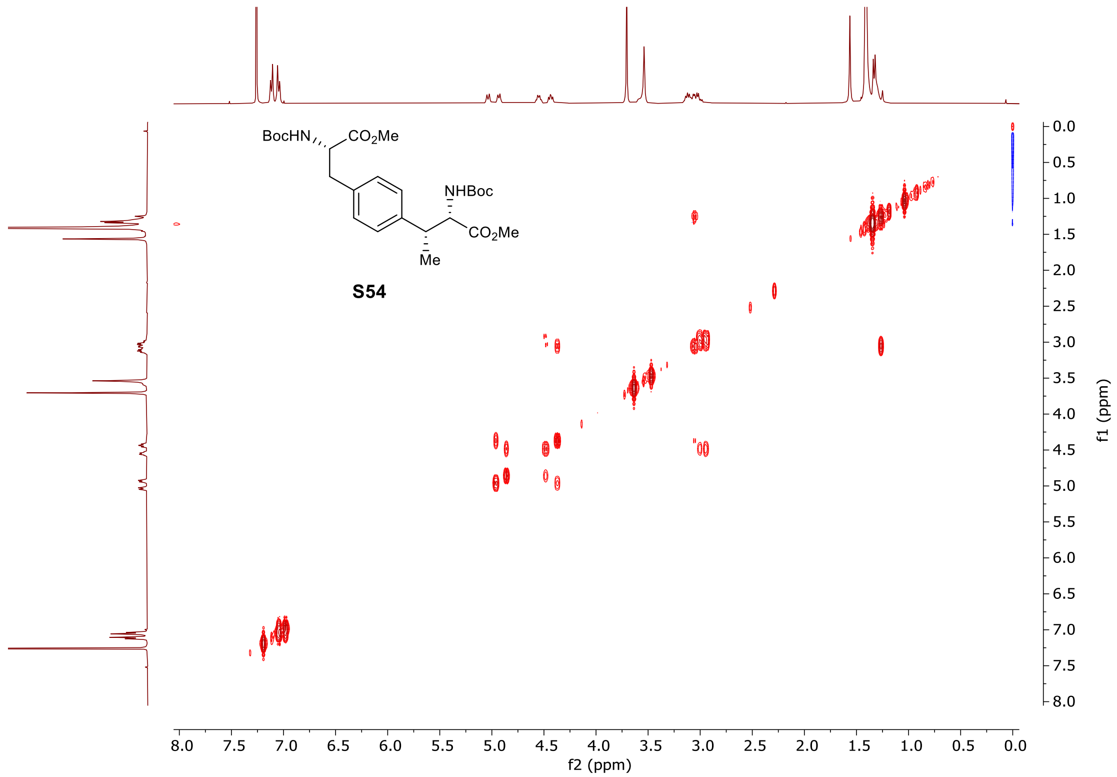

TOCSY (800 MHz, $CDCl_3$) of compound **S54-D2**

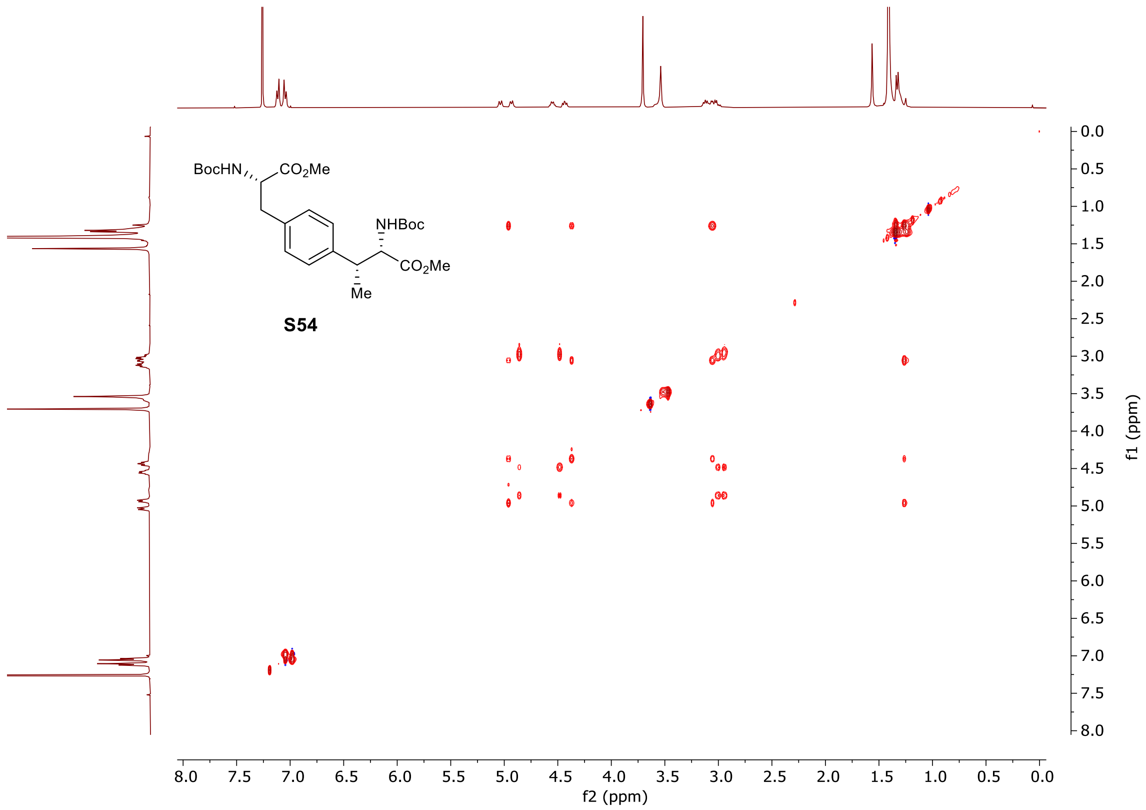

^{1}H NMR (800 MHz, MeOD, 25 °C) of compound **26** (15:1 isolated dr)

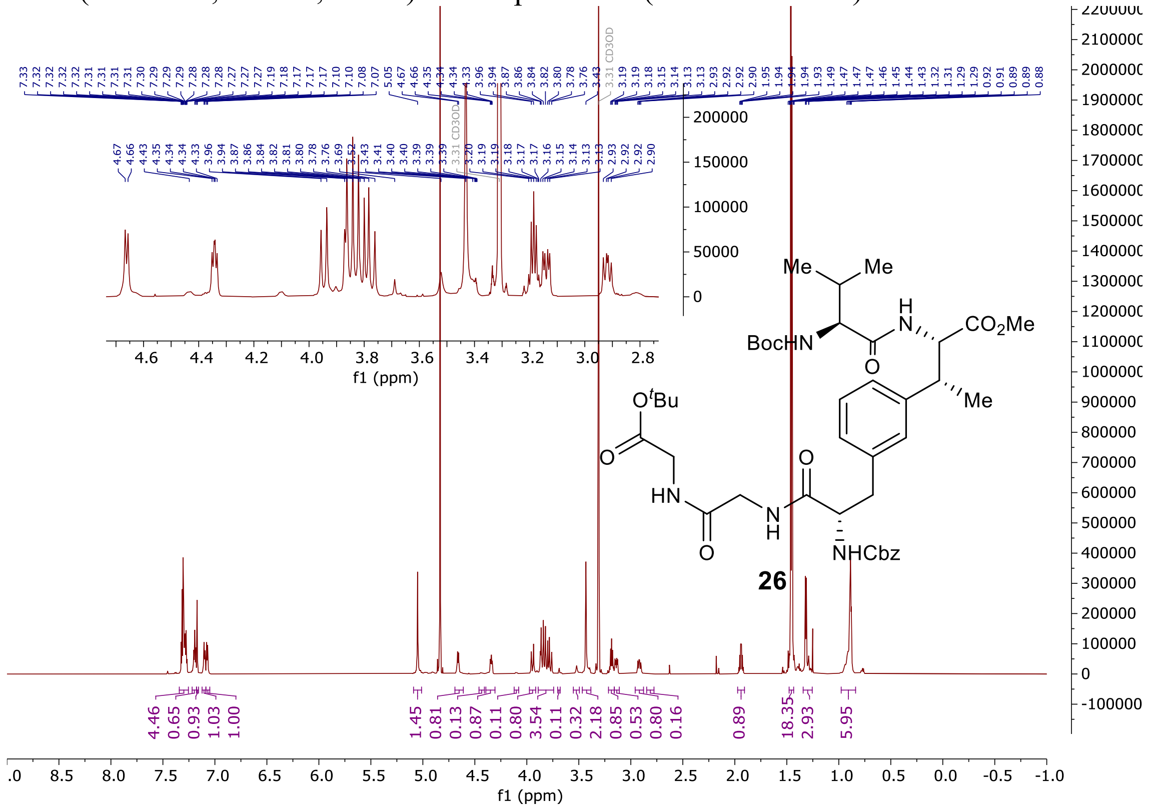

^{1}H NMR (800 MHz, MeOD, 50 °C) of compound **26**

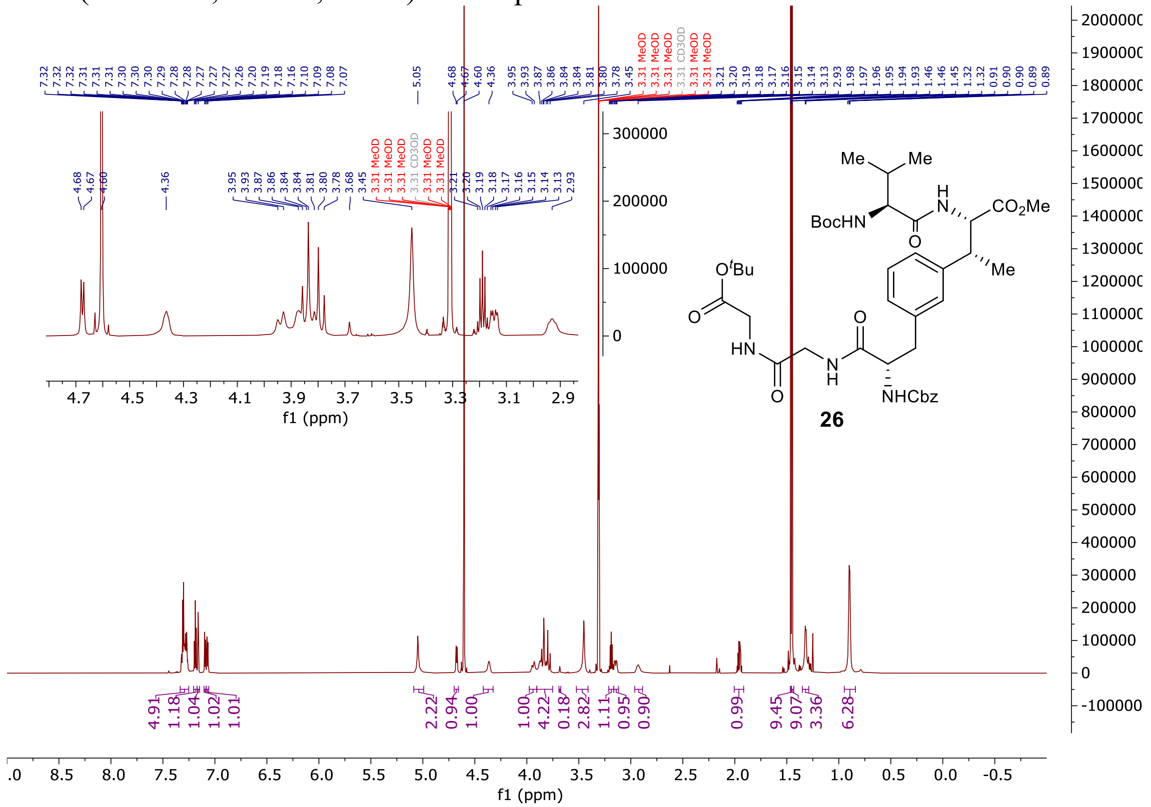

^{13}C NMR (201 MHz, MeOD) of compound **26**

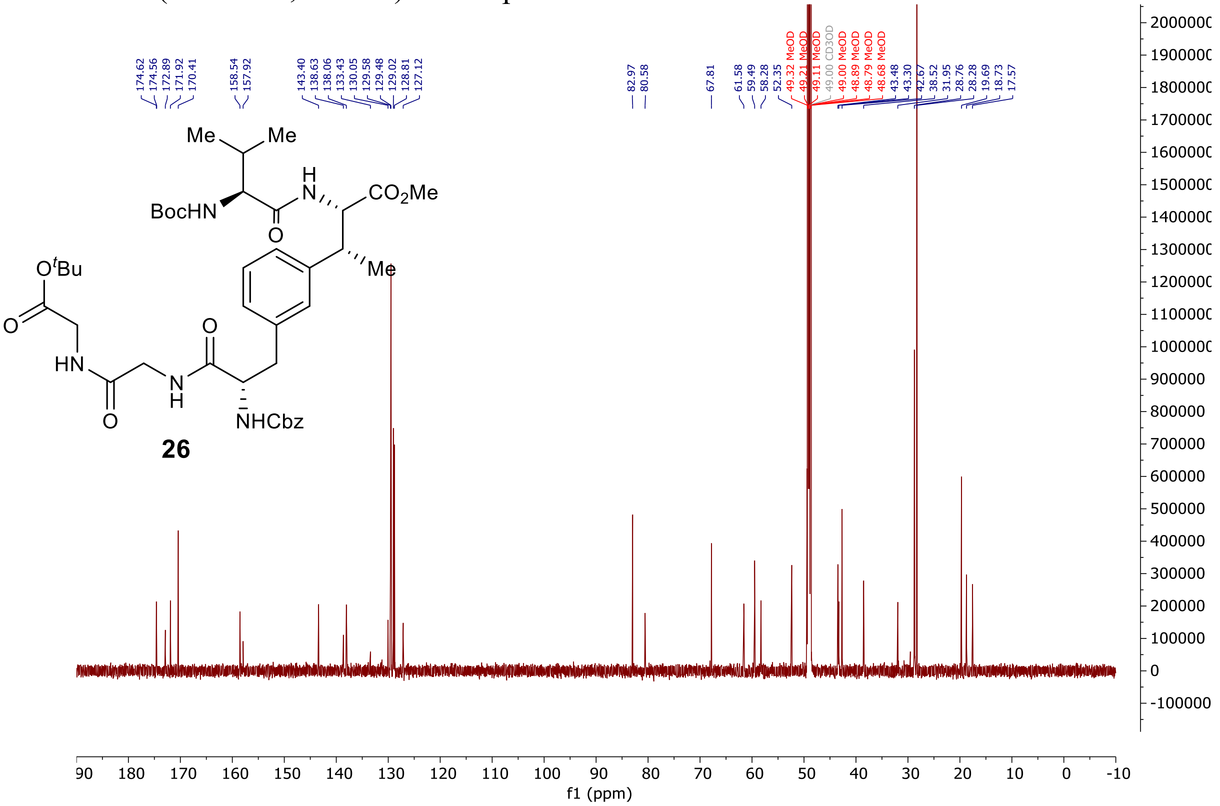

COSY (800 MHz, MeOD) of compound **26**

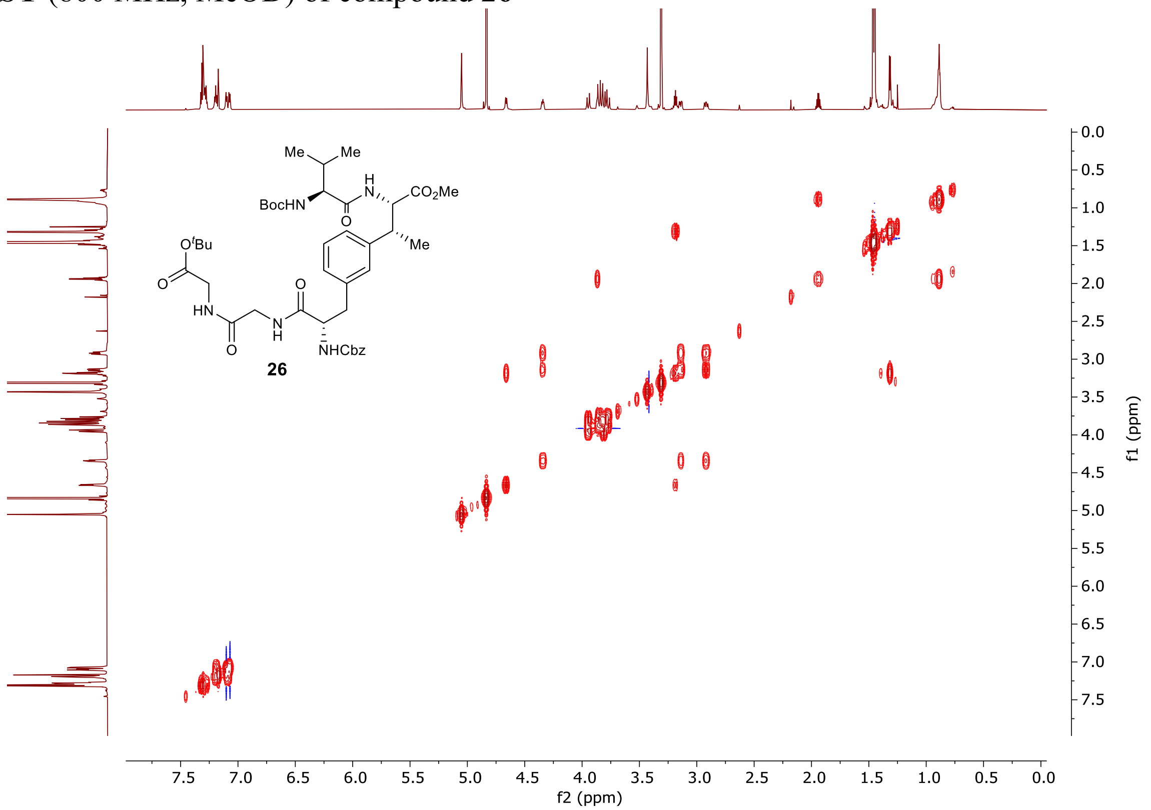

HMBC (800 MHz, MeOD) of compound **26**

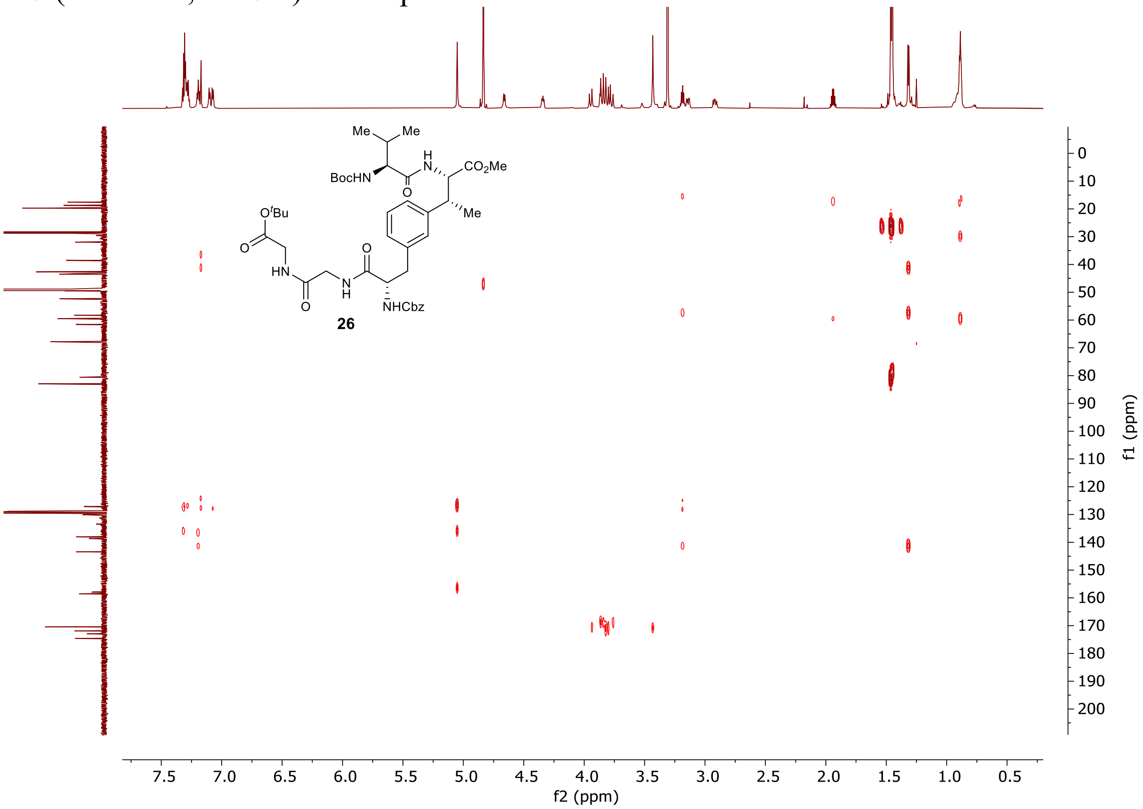

^{1}H NMR (800 MHz, MeOD, 25 °C) of compound **27**

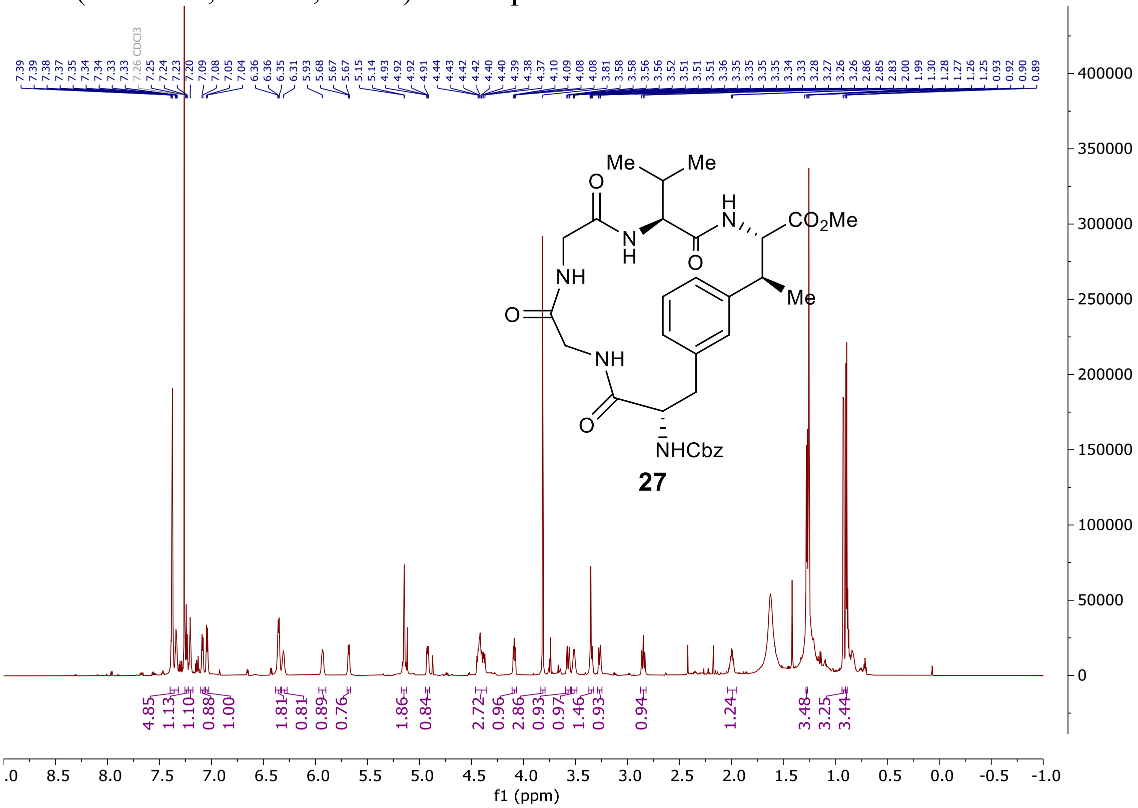

^{13}C NMR (201 MHz, MeOD) of compound **27**

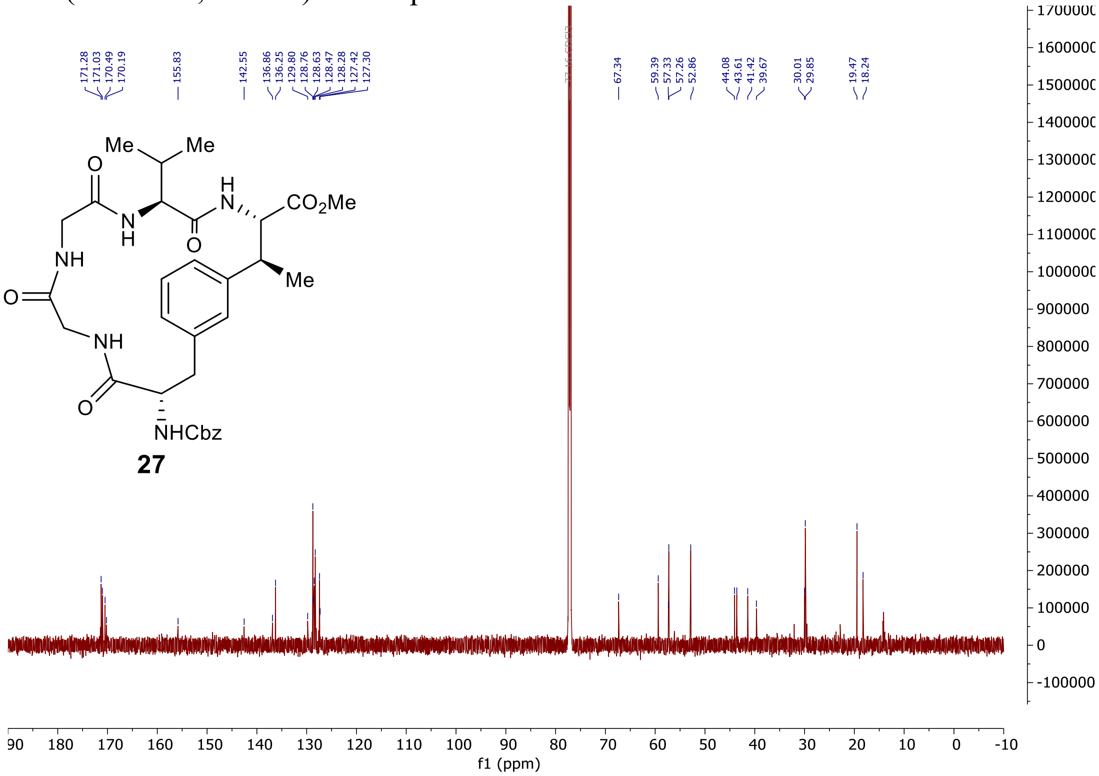

COSY (800 MHz, MeOD) of compound **27**

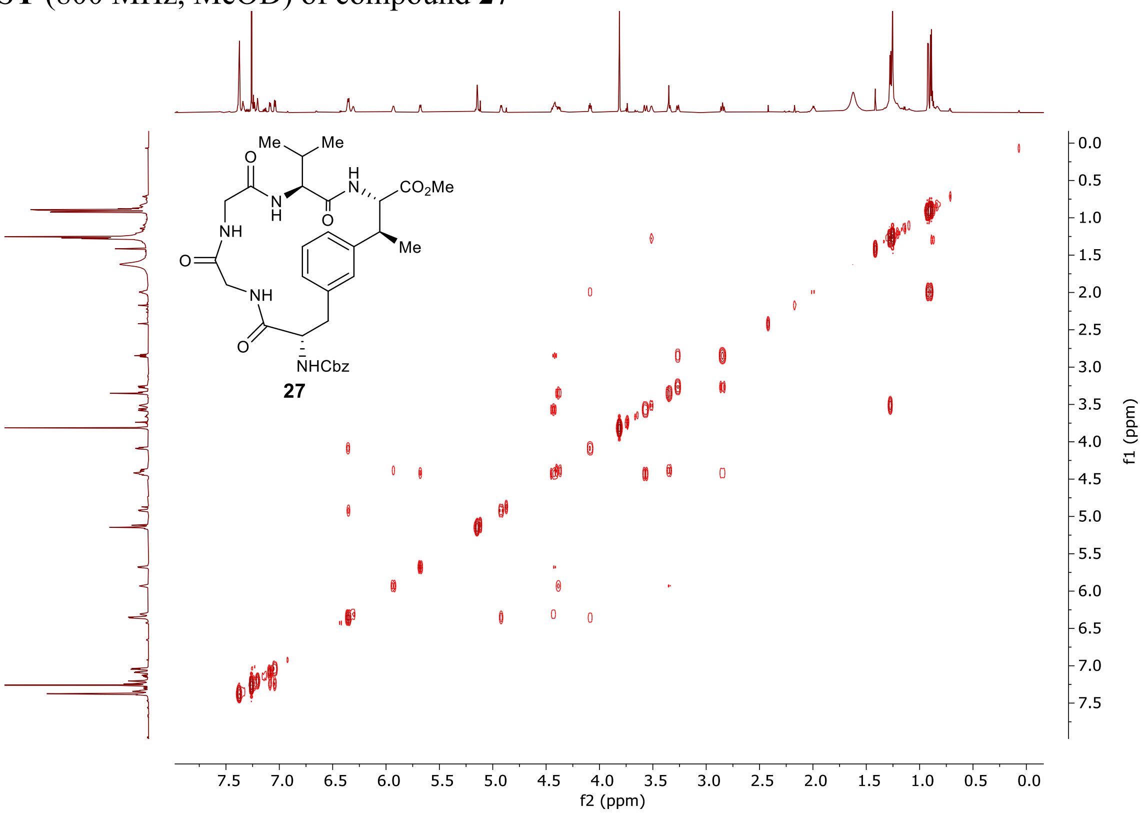

HMBC (800 MHz, MeOD) of compound **27**

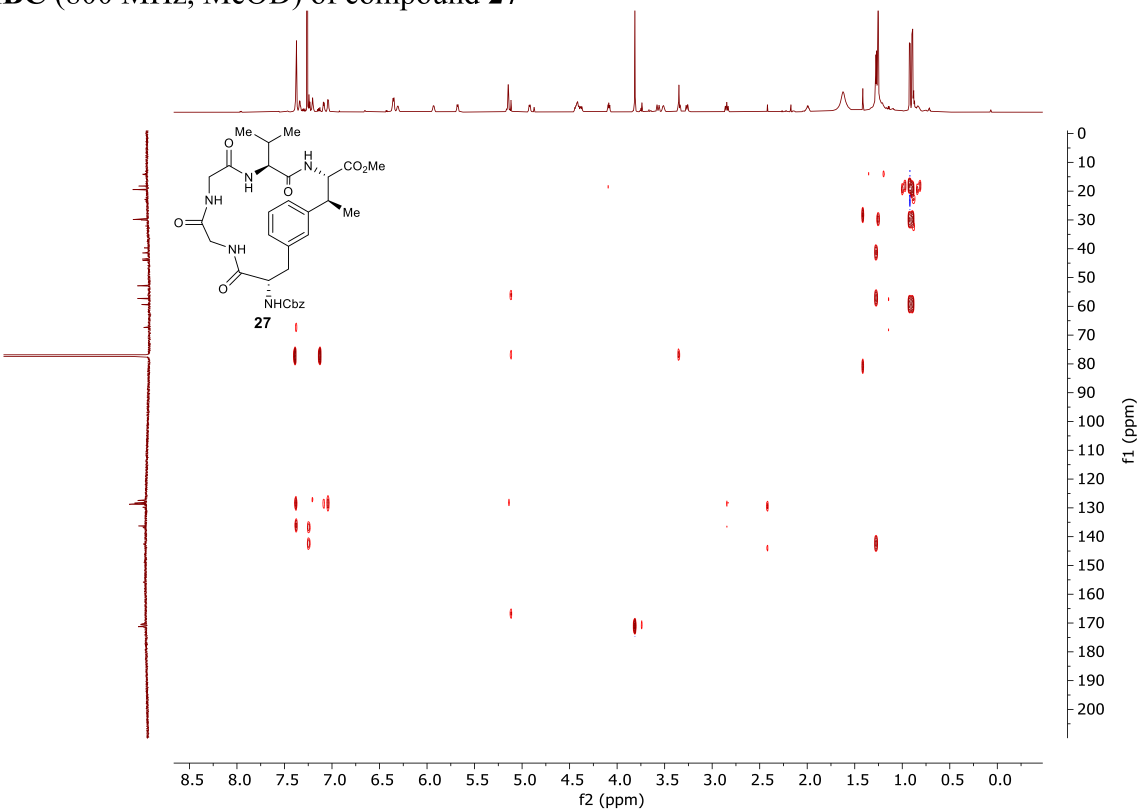

NOESY (800 MHz, MeOD) of compound **27**

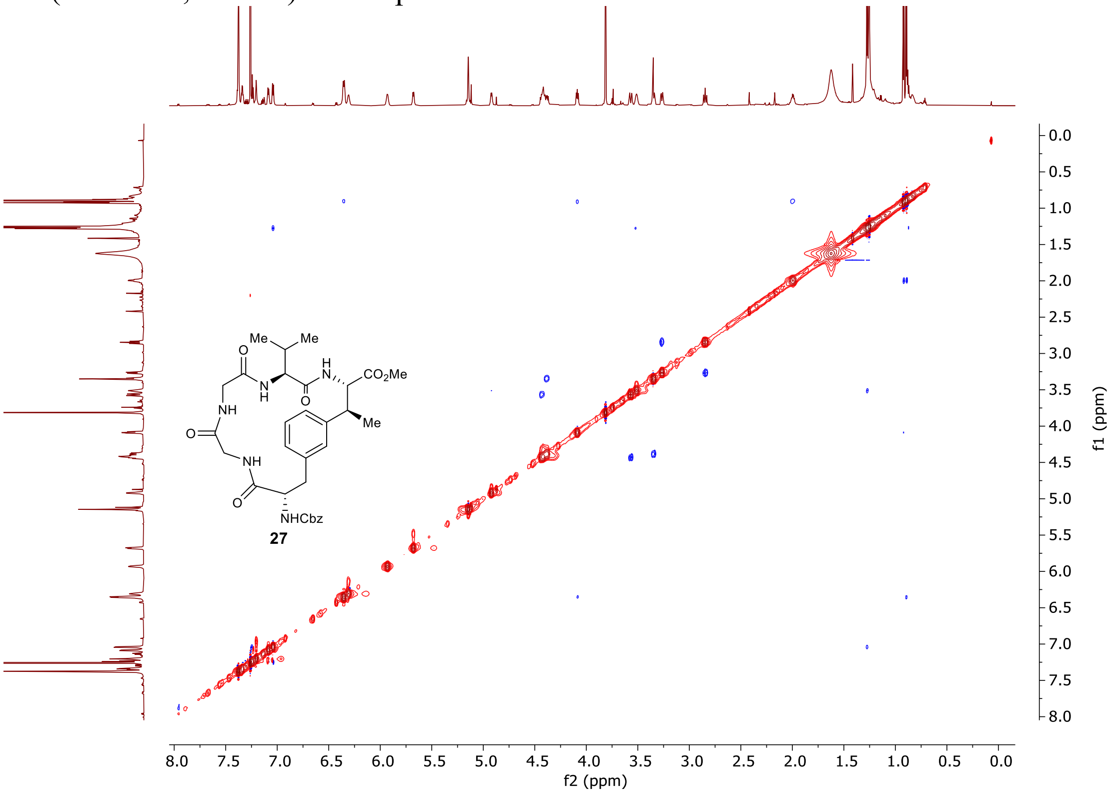

Supplement: Supplementary file 1 [file ol5c02198_si_001.pdf]
